# Exploring the Transcriptome of Ciliated Cells Using In Silico Dissection of Human Tissues

**DOI:** 10.1371/journal.pone.0035618

**Published:** 2012-04-25

**Authors:** Alexander E. Ivliev, Peter A. C. 't Hoen, Willeke M. C. van Roon-Mom, Dorien J. M. Peters, Marina G. Sergeeva

**Affiliations:** 1 A. N. Belozersky Institute of Physico-Chemical Biology, Moscow State University, Moscow, Russia; 2 Center for Human and Clinical Genetics, Leiden University Medical Center, Leiden, The Netherlands; AC Camargo Cancer Hospital, Brazil

## Abstract

Cilia are cell organelles that play important roles in cell motility, sensory and developmental functions and are involved in a range of human diseases, known as ciliopathies. Here, we search for novel human genes related to cilia using a strategy that exploits the previously reported tendency of cell type-specific genes to be coexpressed in the transcriptome of complex tissues. Gene coexpression networks were constructed using the noise-resistant WGCNA algorithm in 12 publicly available microarray datasets from human tissues rich in motile cilia: airways, fallopian tubes and brain. A cilia-related coexpression module was detected in 10 out of the 12 datasets. A consensus analysis of this module's gene composition recapitulated 297 known and predicted 74 novel cilia-related genes. 82% of the novel candidates were supported by tissue-specificity expression data from GEO and/or proteomic data from the Human Protein Atlas. The novel findings included a set of genes (DCDC2, DYX1C1, KIAA0319) related to a neurological disease dyslexia suggesting their potential involvement in ciliary functions. Furthermore, we searched for differences in gene composition of the ciliary module between the tissues. A multidrug-and-toxin extrusion transporter MATE2 (SLC47A2) was found as a brain-specific central gene in the ciliary module. We confirm the localization of MATE2 in cilia by immunofluorescence staining using MDCK cells as a model. While MATE2 has previously gained attention as a pharmacologically relevant transporter, its potential relation to cilia is suggested for the first time. Taken together, our large-scale analysis of gene coexpression networks identifies novel genes related to human cell cilia.

## Introduction

Cilia are microtubule-rich organelles which protrude from cell surface and play important roles in motility, sensory perception [1] and development [2] in a wide range of eukaryotes including human. In the adult human body, epithelial cells with motile cilia are highly abundant in airways, reproductive tracts and specific brain regions [3]. In these tissues, motile cilia are important for clearance of mucosa (airways), transport of oocytes (fallopian tubes) and circulation of cerebrospinal fluid (brain) [4]. Although many human tissues contain cells with a single non-motile cilium (called a ‘primary’ cilium) [5], airways, fallopian tubes and specific brain regions are peculiar in containing epithelial cells with numerous and motile cilia [4]. Mutations leading to defects in motile cilia cause ciliopathies involving symptoms such as hydrocephalus, chronic airway infections, infertility and developmental abnormalities, including situs inversus and congenital heart defects [3], [6]. Identification of proteins that are involved in cilia biogenesis and motion is important for understanding how cilia function in health and disease [3], [7].

Motile cilia have a highly ordered inner structure formed by doublets of microtubules that are interconnected with a number of multiprotein complexes, e.g. radial spokes, nexin links, central sheath and dynein arms [4]. Although a subset of cilia-related proteins are known, the complete range of proteins required for biogenesis and functioning of cilia remains to be determined [4], [7]. Several high-throughput studies explored the ciliome in various organisms using analysis of gene sequence [8], [9], [10], transcript [11], [12], [13] and protein abundances [14], [15], [16]. These studies resulted in identification of novel cilia-related genes, as summarized in the Ciliary Proteome database [14] and Ciliome DB [7]. Mutations in some of these genes were subsequently found to be associated with human ciliopathies [15], [16].

We hypothesized that a novel approach involving a large-scale meta-analysis of gene coexpression networks will provide a new insight into biology of cells with motile cilia. Analysis of gene coexpression networks represents a powerful methodology that allows to reveal modules of coordinately expressed genes in an unsupervised manner, each module corresponding to a specific biological driving factor [17], [18]. It was recently found that gene coexpression networks generated from tissue-level data include not only modules related to universal cellular functions (protein synthesis, energy metabolism, etc.) but also those corresponding to individual cell types [19]. This is potentially explained by the fact that relative abundances of cell types are expected to vary from sample to sample, leading to coordinate changes in expression levels of genes transcribed specifically in each cell type [19]. Such a variation provides opportunity to identify cell type-specific genes based on expression data from physically undissected tissues [19], [20]. We applied this *in silico* tissue dissection approach to characterize the transcriptome of cells with motile cilia.

In the present study, coexpression networks were constructed based on a large set of published microarray data from the tissues rich in motile cilia: airways, fallopian tubes and brain (a total of 1,615 samples from 12 independent datasets). The analysis revealed a highly reproducible coexpression module pertaining to cells with motile cilia. This module was further searched for genes shared and unique across the tissues. The analysis predicted novel potential cilia-related genes, including those involved in a neurological disorder dyslexia (DCDC2, DYX1C1, KIAA0319) and a pharmacologically relevant small molecule transporter MATE2 (SLC47A2). These findings provide novel insights into the human ciliome.

## Results

### Detection of the ciliary coexpression module in multiple tissue types

**Figure 1:**
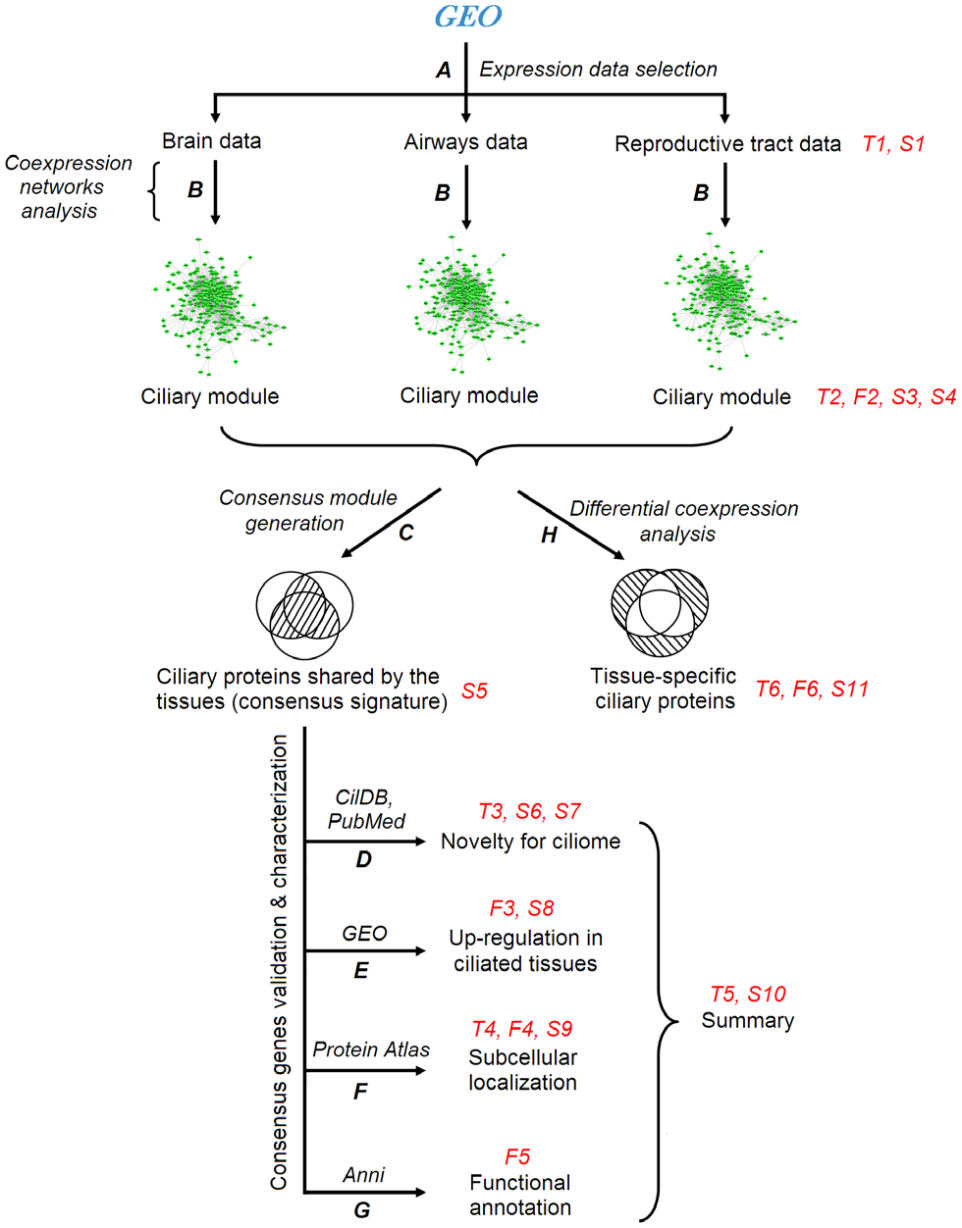
Flowchart of analysis. Marks in italics - databases, programs and analysis types. Red marks - tables (“T”), figures (“F”) and supplementary tables (“S”). **A.** Search in the GEO database: selection of data pertaining to brain, airways and reproductive tracts. **B.** Construction of coexpression networks in each dataset using WGCNA algorithm: identification of coexpression modules. **C.** Generation of a consensus ciliary module: identification of genes shared by the tissues. D to G: validation and characterization of genes in the consensus signature. **D.** Discrimination between known, candidate and novel ciliary genes (CilDB and PubMed databases). **E.** Determination which genes from the consensus signature are up-regulated in ‘ciliated’ tissues compared to ‘non-ciliated’ tissues (GEO database). **F.** Determination which proteins from the consensus signature have characteristic patterns of subcellular localization in ciliated cells (Protein Atlas database). **G.** Linking genes to cellular functions and human diseases using literature mining (Anni 2.1 program). **H.** Differential coexpression analysis: identification of genes which represent members of the ciliary module in only a subset of ciliated tissues.

To assemble a biologically and technically broad sampling of data, we searched the gene expression repository Gene Expression Omnibus [21] for microarray datasets that quantify gene expression in brain, airways and reproductive tracts – Fig. 1. The search resulted in 12 large datasets (5 – for brain, 5 – for airways and 2 – for reproductive tracts) (Table 1). Each dataset described expression levels of at least 15,000 genes in at least 24 samples (Table 1). For each brain dataset, we additionally created sub-datasets composed of samples from individual anatomical regions (cortex, pons, cerebellum and others, see Table S1) to account for the fact that brain regions strongly differ from each other transcriptionally and histologically [19], [22].

**Table 1:** Gene expression datasets.

Tissue type	Dataset GEO ID	Description	Platform	Normal samples
I	GSE15745	Brain tissue	Illumina HumanRef-8 v2.0	584
	GSE11882	Brain tissue	Affymetrix U133Plus	173
	GSE15222	Brain tissue	Illumina HumanRef-8	363
	GSE13344	Brain tissue	Affymetrix 1.0 ST Array	95
	GSE5281	Brain tissue	Affymetrix U133Plus	74
II	GSE13933	Airway brushing	Affymetrix U133Plus	87
	GSE19188	Lung tissue	Affymetrix U133Plus	65
	GSE18842	Lung tissue	Affymetrix U133Plus	45
	GSE4302	Airway brushing	Affymetrix U133Plus	44
	GSE5264	Airway epithelial cells	Affymetrix U133Plus	30
III	GSE12446	Endometrium	Affymetrix U133Plus	31
	GSE10971	Fallopian tubes	Affymetrix U133Plus	24

A coexpression network was constructed in each dataset (Fig. 1A) using an advanced algorithm – Weighted Gene Coexpression Network Analysis (WGCNA) [18], [23] – with our previously described procedure for genome-wide analysis [24] (Methods). The analysis revealed from 11 to 46 coexpression modules in the different datasets. The modules contained approximately 400 genes on average. By definition, genes in each module showed highly similar expression profiles.

To infer biological factors driving formation of each module, we tested each module for an enrichment in genes with shared functional annotations using DAVID [25]. The modules were found to be related to a wide range of biological processes (e.g. cell division, ATP synthesis, cell adhesion, extracellular matrix remodeling) and cell types (immune cells, neurons and others) (see Table S2 for details). While certain functions were represented in all the tissues, others were limited to a single tissue type (e.g. synaptic transmission in brain). The observed diversity of the modules is consistent with the previous studies of the human transcriptome [17], [19].

To determine whether any of these modules are related to ciliated cells, we tested each module for overlap with an established set of 75 known ciliary proteins – a collection compiled by Gherman and colleagues based on the previous ciliome studies [14]. 10 out of the 12 datasets were found to include a module significantly enriched in these golden-standard ciliary proteins (Table 2; P<0.001, Fisher's exact test). Furthermore, annotations of these modules produced by DAVID [25] were also related to cilia (Table S2).

**Table 2:** Detection of ciliary coexpression modules.

Tissue type	Dataset GEO ID	Description	Total number of modules	Size of ciliary module	Ciliary markers*	Further analysis
I	GSE15745	Brain (all regions)	20	195	4×10^−9^	No
		Brain (pons)	28	350	5×10^−11^	***Yes***
		Brain (cerebellum)	19	0	>10^−3^	No
		Brain (cerebral cortex)	19	0	>10^−3^	No
	GSE11882	Brain (all regions)	13	287	9×10^−5^	No
		Brain (hippocampus)	38	205	1×10^−11^	***Yes***
		Brain (cerebral cortex)	11	0	>10^−3^	No
	GSE15222	Brain (all regions)	22	67	3×10^−7^	***Yes***
	GSE13344	Brain (all regions)	32	393	5×10^−10^	***Yes***
	GSE5281	Brain (all regions)	30	0	>10^−3^	No
II	GSE13933	Airways	18	1285	2×10^−12^	***Yes***
	GSE19188	Lung tissue	28	609	2×10^−22^	***Yes***
	GSE18842	Lung tissue	38	357	6×10^−20^	***Yes***
	GSE4302	Airways	28	0	>10^−3^	No
	GSE5264	Airway cells	46	1330	5×10^−32^	***Yes***
III	GSE12446	Endometrium	30	681	2×10^−22^	***Yes***
	GSE10971	Fallopian tubes	25	524	6×10^−11^	***Yes***

* – enrichment of the ciliary module with ciliary markers from the Gherman's list (Fisher's exact test P-value). For datasets, where no ciliary module was detected, a P-value “>10^−3^” is specified because no module reached this threshold of statistical significance.

In the brain datasets where anatomical region-specific networks were available (GSE15745 and GSE11882), the ciliary module was detected in pons (GSE15745) and hippocampus (GSE11882). This is consistent with the literature since pons and hippocampus are located in a close proximity to ventricles typically lined with ciliated epithelium [26]. Because enrichment of the module with ciliary markers was stronger in these region-specific networks than in the multi-region networks in these datasets (Table 2), we used the respective region specific ciliary modules for further analysis in these datasets. In the other brain datasets, only whole-dataset networks were available. Therefore, ciliary modules from whole-dataset networks were used for further analysis in these datasets (Table 2).

Gene composition of the ciliary module in brain, airways and reproductive tracts is provided in a dataset-by-dataset form in the Table S3. The gene composition results for the 10 datasets provide a robust basis for transcriptional characterization of cells with motile cilia. Based on these data, we sought to identify transcripts shared by ciliated cells from the different tissues (Fig. 1C–G) and those expressed in a tissue-specific way (Fig. 1H).

### Determination of a consensus ciliary signature

Over-representation of genes with the same biological function in a set of modules across datasets does not guarantee that these modules are highly similar in their gene composition. To assess mutual similarity of the ciliary modules, we compared them with each other and with the rest of the modules across the datasets. The ciliary modules were found to share on average more than 50% of genes with each other, this overlap statistically highly significant (P<10^−20^ for the least significant pair of the datasets; see Table S4 for details on the cross-dataset overlap of the modules). Furthermore, the ciliary modules were more similar to each other than to any other modules, which allows them to be viewed as variants of the same module in the different functional contexts.

**Figure 2:**
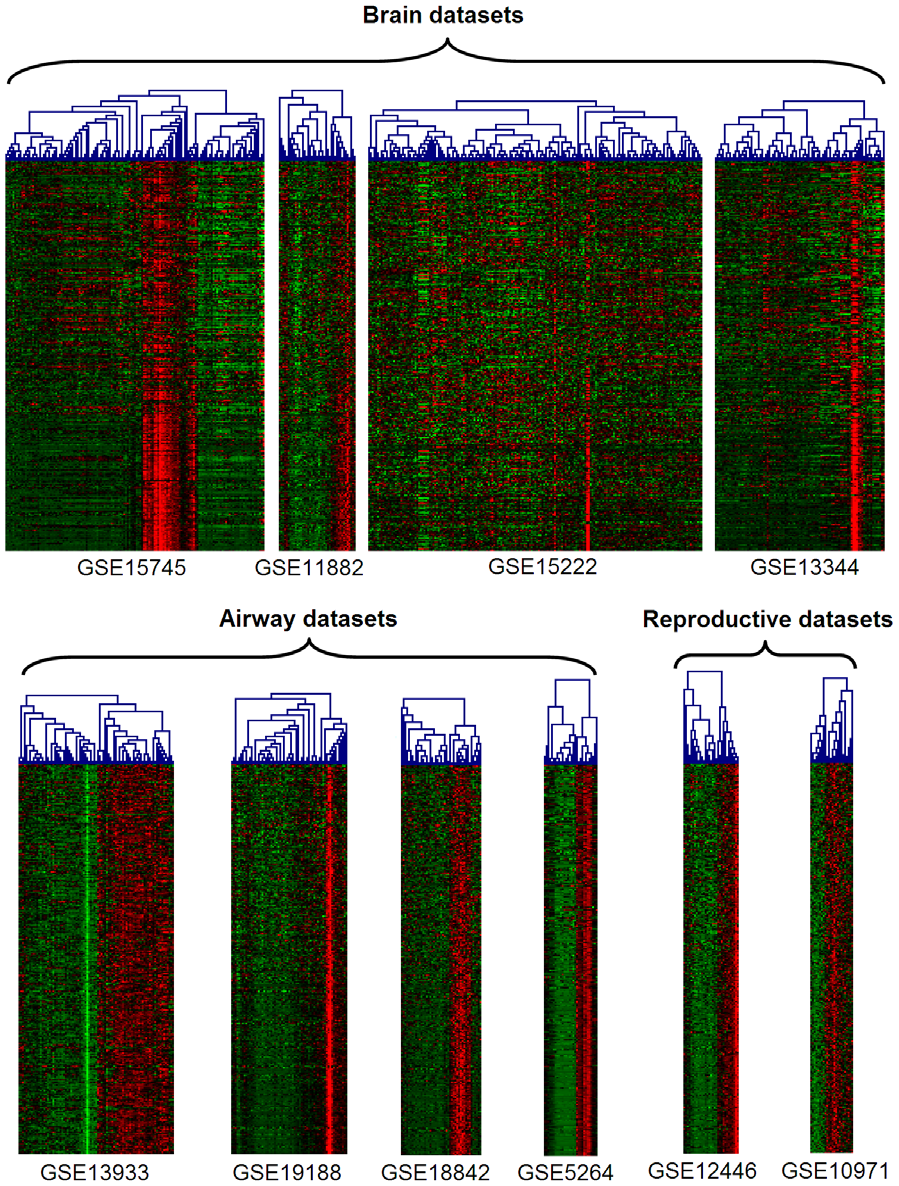
Heatmaps of consensus ciliary signature in 10 contributing datasets. Red - high, green - low level of expression. Columns – samples, rows – genes. Samples were clustered separately in each dataset. Genes were ordered by the number of datasets in which they belonged to the ciliary module: the gene order is constant across the datasets. Genes, that lacked measurements in a subset of the experiments, were excluded from the heatmaps.

The cross-dataset consistency of the ciliary module enabled summarization of the modules' gene composition into a consensus ciliary signature. The signature was compiled of genes belonging to the ciliary module in at least 2 of the 3 tissues (brain, airways and reproductive tracts) and, simultaneously, in at least 4 of the 10 datasets (FDR<0.5%, permutation-based test, Methods). Joint application of the two requirements produced a consensus signature composed of 371 genes (Table S5). Expression patterns of the consensus genes in the original datasets were plotted as heatmaps (Fig. 2). The visualization confirmed consistent coexpression of the identified genes in the analyzed tissues.

### Consensus signature predicts novel cilia-related genes

We compared our consensus signature with the ciliary gene lists from the earlier studies. For this purpose, the largest ciliome resource – CilDB [27] – was used. This database provides an extension of the key earlier resources – Ciliary Proteome Database [14] and Ciliome Database [7]. Studies in CilDB can be grouped into 4 categories based on the underlying approach: comparative genomics [9], regulatory genomics (i.e. identification of cilia-related motifs in gene promoters) [28], gene expression analyses [13] and proteomics [29]. Since many studies were performed on model organisms, for each gene from the signature we determined its orthologs in the model organisms (see Table S6 for the gene-by-gene orthology information). Conservation rate of the signature genes was consistent with that previously reported [29], [30]. The consensus signature orthologs were next compared with the ciliary gene lists from the CilDB studies. Despite the methodological diversity of the studies, a highly significant overlap between the gene list from our study and those from the previous studies was observed (see Table 3 for the best matching studies per methodological category; also see Table S6 for data on the complete set of the studies).

**Table 3:** Comparison of the consensus signature with lists of ciliary genes from the previous studies.

Study	Organism	Genes reported as ciliary*	Signature genes shared by genomes§	Overlap with signature (absolute)†	Overlap with signature (percent)‡	P-value**
*Comparative genomics*						
[9]	Green alga	605	138	78	57%	3×10^−35^
[10]	Green alga	332	138	63	46%	5×10^−38^
[8]	Fruit fly	343	164	41	25%	2×10^−25^
*Regulatory genomics (X-box in promoters)*						
[56]	Worm	2429	98	42	43%	6×10^−5^
[57]	Friut fly	631	164	26	16%	5×10^−6^
[28]	Worm	860	98	17	17%	5×10^−3^
*Gene expression analyses*						
[12]	Human	1305	371	220	59%	4×10^−179^
[30]	Paramecium	677	154	97	63%	9×10^−48^
[11]	Mouse	116	360	61	17%	4×10^−75^
*Proteomics*						
[29]	Green alga	1003	138	96	70%	8×10^−35^
[27]	Paramecium	736	154	90	58%	5×10^−38^
[58]	Human	248	371	84	23%	1×10^−88^

For each category, the table shows 3 studies that exhibited the largest overlap with the consensus signature; results for a broader range of studies are presented in the Table S6. Furthermore, for each individual gene from the signature, orthologs in each model organism are also provided in the Table S6.

* - Number of human orthologs that correspond to the list of ciliary genes in a given study.

§ - Number of human genes in the consensus signature with orthologs in the respective model organism.

† - Number of genes shared by the reported gene list and our consensus signature.

‡ - Percentage of shared genes from the total number of signature genes that have orthologs in the respective model organism.

** - Statistical significance of the overlap (Fisher's exact test). Organisms: ‘Fruit fly’ – D. melanogaster, ‘Green alga’ – C. reinhardtii, ‘Mouse’ – M. musculus, ‘Paramecium’ – P. tetraurelia, ‘Worm’ – C. elegans.

To distinguish between known genes and novel predictions, we characterized each gene in the signature by strength of experimental support in CilDB and whether the gene was mentioned as cilia-related in MEDLINE. This resulted in stratification of the signature genes into 3 categories: (I) 237 genes with strong evidence from the previous studies (“known ciliary genes”), (II) 60 genes with weak evidence from the studies (“previously proposed candidates”) and (III) 74 genes with no evidence from the previous studies (“novel candidates”). The gene-to-category mapping is provided in the Table S7.

### Tissue-specificity analysis supports the novel predictions

**Figure 3:**
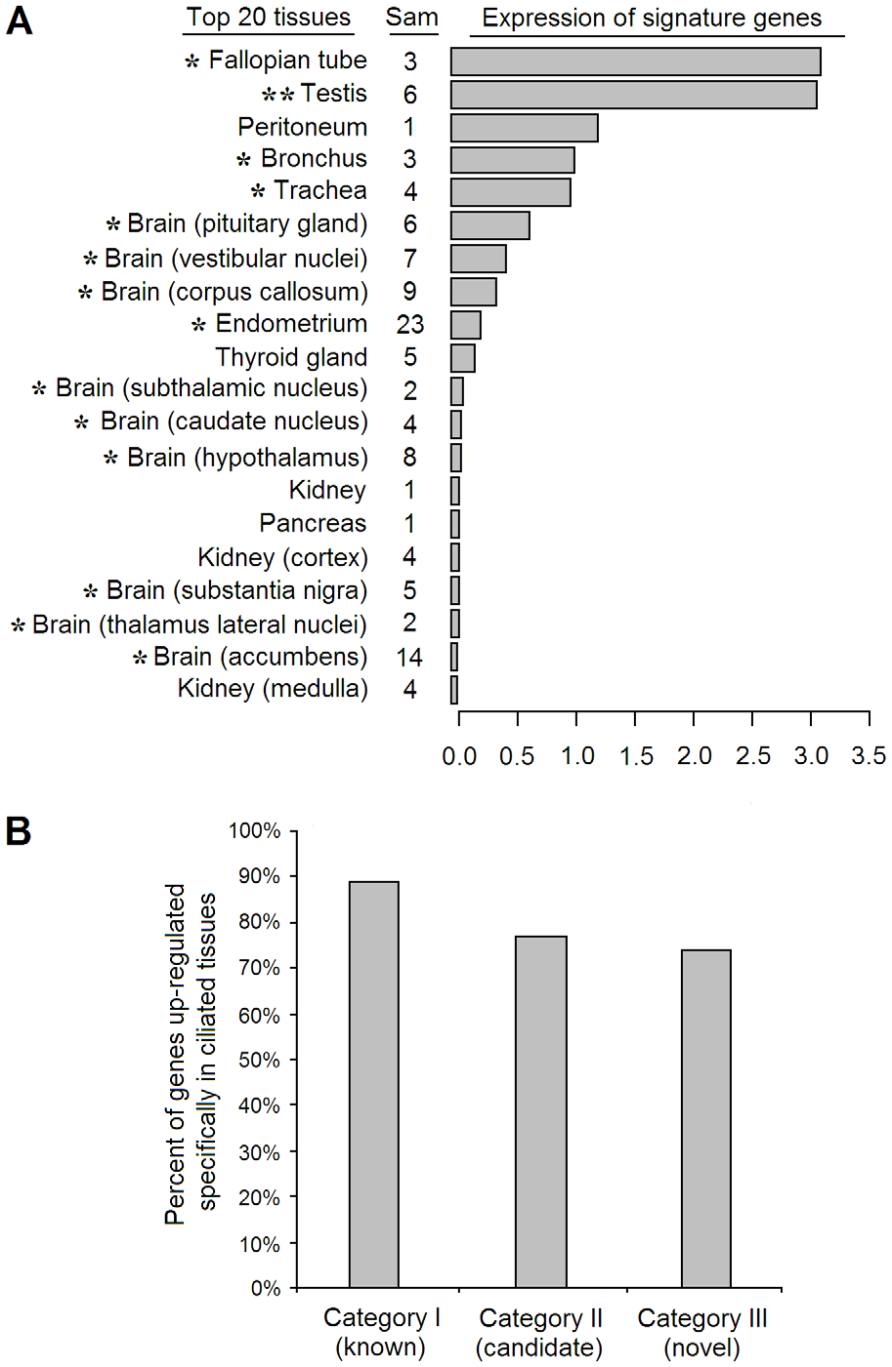
Tissue specificity analysis of the signature genes. (**A**) Top 20 tissues (out of the total 104 human tissues available in GSE7307) with highest mean expression level of the 326 signature genes. The “Sam” column describes the number of samples available for each tissue in the dataset. The bars show mean Z-score expression level of the signature genes in the respective tissues. An asterisk marks tissues that were previously reported to contain cells with motile cilia. A double asterisk marks testis that contains spermatozoa with motile flagellum (an organelle related to motile cilia). (**B**) Percentage of signature genes up-regulated in ciliated tissues – by literature-based categories. Up-regulated genes were detected using a permutation-based test that compared expression level of a given gene in the union of trachea, bronchus, lung, fallopian tube, endometrium and testis versus the rest of the tissues in the GSE7307 dataset (P<0.05, Methods).

To validate the predictions, we explored tissue-specificity of the signature genes in the human body. The largest publicly available microarray dataset (GSE7307) measuring gene expression levels across 104 normal human tissues was analyzed. Consistent with a high content of ciliary genes in the signature, average expression level of the complete signature was highest in tissues containing cells with motile cilia – out of a broad range of human tissues (Fig. 3A). Furthermore, for each gene, we separately calculated a gene-specific P-value that estimated whether the gene is preferentially up-regulated in tissues rich in motile cilia/flagella (trachea, bronchus, lung, fallopian tubes, endometrium, testis), using a permutation-based test (Methods). The tissue-specificity P-values are provided in the Table S8. 89% of the known ciliary genes, 77% of the previously proposed and 74% of the novel ciliary candidates were found to be significantly up-regulated in the ciliated tissues (Fig. 3B). For the ciliary candidates, these data support them as functionally related to motile cilia.

### Characterizing the signature at the protein level using Human Protein Atlas data

To further validate and characterize our predictions, we analyzed protein-level immunostaining data from the Human Protein Atlas – a large-scale antibody-based resource on protein expression in human tissues [31]. 218 of the signature proteins were available in the database. For each protein, immunostaining images from airways and fallopian tubes were analyzed (brain ciliated epithelium data were unavailable). Each protein was attributed to a class: (A) proteins preferentially expressed in ciliated cells and localized in the ciliary compartment, (B) proteins preferentially expressed in ciliated cells and localized in other subcellular compartments, (C) proteins with no evidence for an association with ciliated cells (i.e. expressed in both ciliated and non-ciliated cells, or not expressed in ciliated cells at all). A summary of this analysis is provided in the Table 4.

**Table 4:** Localization patterns of signature proteins from distinct novelty categories.

	Staining is strongest in cilia	Staining is strongest in non-ciliary compartments of ciliated cells	Staining is not associated with ciliated cells
Known ciliary genes (cat. I)	76 (56%)	34 (25%)	26 (19%)
Previously proposed (cat. II)	13 (38%)	8 (24%)	13 (38%)
Novel candidates (cat. III)	20 (42%)	5 (10%)	23 (48%)
All signature genes (total)	109 (50%)	47 (22%)	62 (28%)

The table describes how many proteins with different localization types were present in each novelty category. Percentages in brackets describe the protein numbers as fractions from the total number of proteins available in the Protein Atlas for a given category (the sum of the percentages in each row equals 100%). ‘Cat.’ is an abbreviation for ‘category’.

Among the known ciliary genes, 56% belonged to the class A, 25% – to the class B and 19% – to the class C. Presence of known ciliary genes in the class C may be explained by imperfect specificity of antibodies to their target proteins. The fact that 81% of the known ciliary genes belong to the classes A and B shows that the Human Protein Atlas data are largely consistent with those from the previous studies.

Among the ciliary candidates, a substantial fraction also belonged to the classes A and B (62% of the previously proposed and 52% of the novel ciliary candidates) (Table 4). This suggests that, although not all, the majority of the candidates are indeed functionally related to motile cilia, thus providing a validation of the transcriptomic predictions.

**Figure 4:**
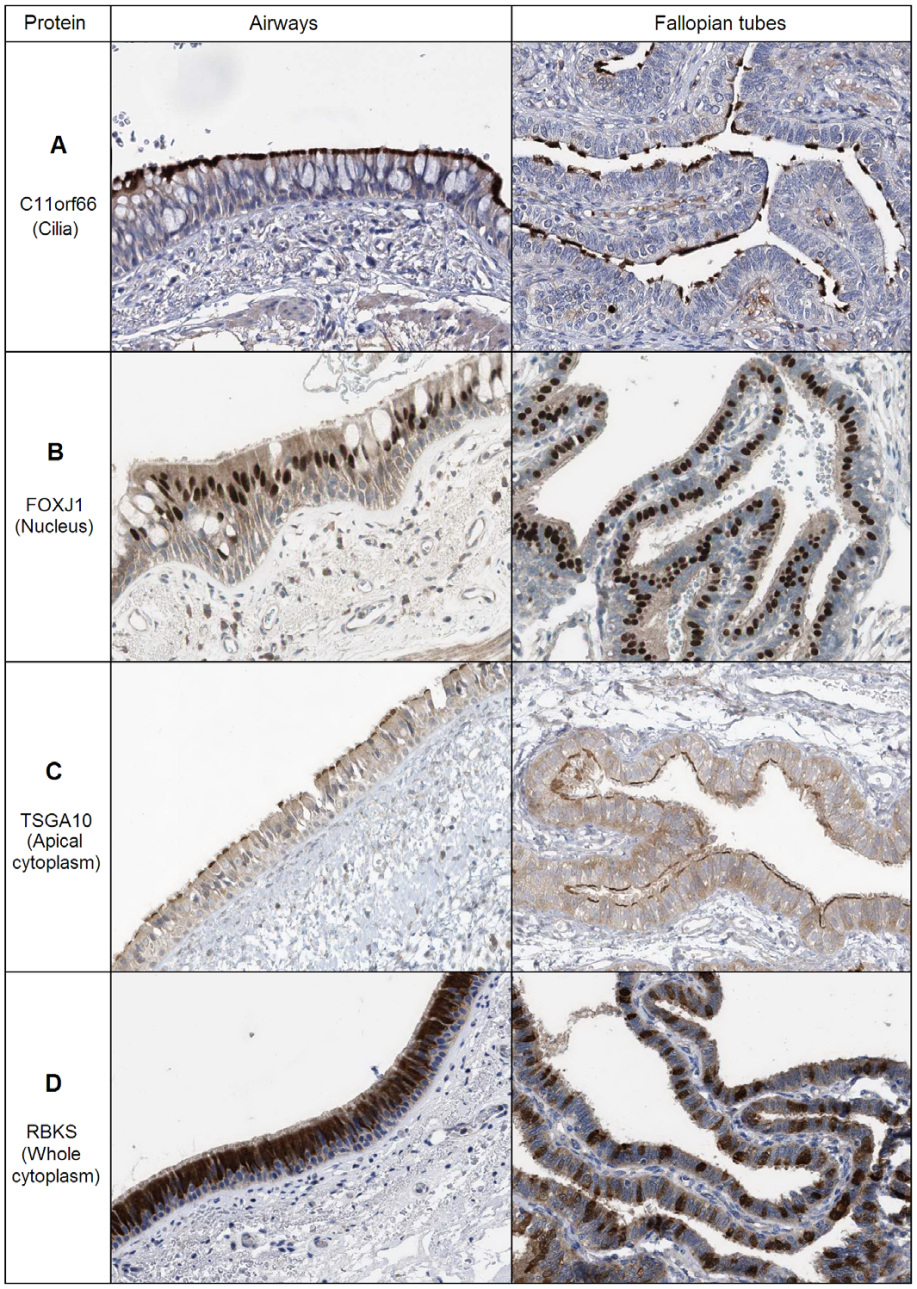
Subcellular localization types of the signature proteins. The images were obtained by immunohistochemical staining of airways and fallopian tubes with protein-specific antibodies in the Protein Atlas project [31]. The antibodies were targeted at the following proteins: (A) C11orf66 (a protein with unknown function), (B) FOXJ1 (a transcription factor known to regulate cilium biogenesis), (C) TSGA10 (a sperm tail protein), (D) RBKS (ribokinase, a ribose metabolism enzyme). Brown corresponds to the antibody-based staining, blue – to staining of nuclei with DAPI. Note that in airways ciliated cells form a continuous layer, while in fallopian tubes they are separated from each other by non-ciliated epithelial cells.

At the subcellular level, the examined proteins included those restricted to cilia (C11orf66, Fig. 4A), nuclei (FOXJ1, Fig. 4B), apical cytoplasm (TSGA10, Fig. 4C) and whole cytoplasm of ciliated cells (RBKS, Fig. 4D). Thus, the signature includes not only direct ciliary components, but also proteins from other subcellular compartments of ciliated cells.

A collection of the immunochemistry images for the 218 signature proteins is provided in the Table S9. This collection provides hypotheses of protein functions [31] and may facilitate selection of ciliary candidates for further investigation (see Discussion).

In addition to the protein-level data, a broader summary of the protein-level and expression-level results is provided in the Table 5 (supported ciliary candidates). The complete summary (for all the signature proteins) is provided in the Table S10.

**Table 5:** Summary for ciliary candidates supported by the Protein Atlas data.

Symbol	Novelty category	Number of datasets in which the gene belongs to the ciliary module	Validation	
			Tissue specificity (P-value)	Protein Atlas
ACYP1	III	5	1.0E-06	Class B (Mixed localization)
ARMC2	III	8	1.7E-06	Class A (Cilia)
BAIAP3	III	4	NS	Class A (Cilia)
C1orf87	II	9	1.0E-06	Class A (Cilia)
C1orf92	III	6	1.0E-06	Class A (Cilia)
C1orf129	III	4	NS	Class B (Apical cytoplasm)
C1orf222	II	4	NS	Class A (Cilia)
C6orf103	II	5	1.0E-06	Class B (Mixed localization)
C9orf9	II	4	1.1E-03	Class A (Cilia)
C10orf92	III	6	8.2E-06	Class A (Cilia)
C11orf63	III	4	1.7E-06	Class A (Cilia)
C11orf66	II	5	NS	Class A (Cilia)
C14orf179	II	4	1.0E-06	Class A (Cilia)
C21orf58	III	7	1.0E-06	Class A (Cilia)
C22orf23	III	7	NS	Class A (Cilia)
CCDC89	III	5	1.6E-05	Class A (Cilia)
CIB1	III	4	1.0E-06	Class A (Cilia)
DCDC5	III	4	1.0E-06	Class B (Apical cytoplasm)
DZIP3	II	6	1.1E-05	Class A (Cilia)
FANK1	II	9	1.0E-06	Class B (Cilia & cytoplasm)
FLJ16686	III	4	2.7E-04	Class B (Apical cytoplasm)
FSD1L	II	4	NS	Class A (Cilia)
IQCK	III	6	1.0E-06	Class A (Cilia)
KIAA0319	III	5	NS	Class A (Cilia)
KCNRG	II	6	1.0E-06	Class A (Cilia)
LPAR3	III	5	1.0E-06	Class A (Cilia)
LRGUK	III	6	5.0E-06	Class A (Cilia)
LRP2BP	II	7	7.9E-05	Class A (Cilia)
LRRC6	II	6	1.0E-06	Class B (Mixed localization)
LRRC18	III	6	7.8E-05	Class A (Cilia)
LRRIQ3	III	4	6.3E-04	Class A (Cilia)
MIPEP	II	5	3.6E-02	Class A (Cilia)
NEK10	II	5	1.9E-02	Class B (Mixed localization)
NUP62CL	III	7	1.0E-06	Class A (Cilia)
PLCH1	II	4	1.7E-06	Class A (Cilia)
PPM1E	III	6	NS	Class A (Cilia)
PPP1R16A	II	4	3.1E-02	Class B (Apical cytoplasm)
RBKS	II	4	1.2E-04	Class B (Cilia & cytoplasm)
RBM20	III	4	NS	Class A (Cilia)
RGS22	III	8	1.0E-06	Class A (Cilia)
SPAG17	II	7	1.0E-06	Class A (Cilia)
SPATA18	II	10	1.0E-06	Class B (Apical cytoplasm)
SYTL3	III	4	7.6E-04	Class B (Apical cytoplasm)
UBXN10	II	8	1.0E-06	Class A (Cilia)
UFC1	III	5	1.0E-06	Class A (Cilia)
WDR49	II	9	8.5E-05	Class B (Apical cytoplasm)

The table includes 46 signature genes that belong to the novelty categories II or III (proteins with weak or no evidence from the previous studies, respectively) and are supported as cilia-related by the Human Protein Atlas data. Summary for all the signature genes is provided in the Table S10. ‘NS’ – non-significant.

### Functional annotation of ciliary genes using literature mining

To gain a deeper insight into functions of the signature genes, we determined biological context in which these genes are studied in the literature. Large-scale mining of MEDLINE complements manually curated gene-annotation databases such as Gene Ontology by providing a broader and, often, more up-to-date description of genes [32].

**Figure 5:**
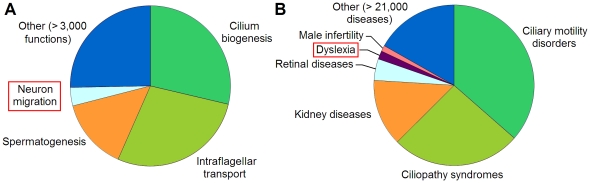
Associations of signature genes with cell functions and diseases suggested by literature mining. **A**. Cell functions. **B**. Diseases and syndromes. The plots depict contributions (%) of each biological term (a specific cell function or disease) into the overall similarity between contexts in which the signature genes are mentioned in the literature [32]. A high contribution value for a given term implies that multiple genes from the signature co-occur with this term in a large number of literature abstracts. The disease categories include the following individual diseases: ‘Ciliary motility disorders’ - ciliary dyskinesias (diseases that manifest mainly in dysfunctions of motile cilia), ‘Ciliopathy syndromes’ - Bardet-Biedl syndrome, Meckel syndrome, Joubert syndrome (diseases that manifest in a broader range of dysfunctions, including those related to non-motile cilia); ‘Kidney diseases’ - nephronophthisis, polycystic kidney disease; ‘Retinal diseases’ - retinitis pigmentosa, retinal dystrophy, Leber amaurosis.

We searched for cell functions (Fig. 5A) and diseases (Fig. 5B) that most frequently co-occur with the signature gene names across a broad collection of MEDLINE abstracts. This was performed using Anni – a biomedical literature mining tool [32]. ‘Cilium biogenesis’, ‘intraflagellar transport’ and ‘spermatogenesis’ were the top-scoring cell functions. They collectively contributed 71% to the overall literature-based cohesion score that measures average similarity between the input genes (Fig. 5A). In the complementary analysis based on disease terms, the top scoring diseases were well-known ciliopathies (Fig. 5B) including kidney and retinal diseases [3].

Surprisingly, the results also included the following terms: ‘neuron migration’ (Fig. 5A) and ‘dyslexia’ (Fig. 5B). These terms corresponded to a group of shared genes: DCDC2, DYX1C1 and KIAA0319. Mutations in these genes are associated with a cognitive neurological disorder, dyslexia, that is thought to be caused by impairments in migration of neurons during embryogenesis [33]. DCDC2, DYX1C1 and KIAA0319 are known to be expressed in neurons and their brain-specific knock-down leads to a decrease in neuron migration in rat embryonic brain [33]. Meanwhile, little is known about the molecular functions of these proteins [33]. Our results suggest that DCDC2, DYX1C1 and KIAA0319 may be involved in biology of cilia (see Discussion).

### Link of the drug-and-toxin transporter MATE2 with the ciliome

In the previous steps, we focused on analysis of the consensus signature – the list of genes apparently shared by ciliated cells across the different tissues. Meanwhile, little is known about how transcriptional signatures of ciliated cells differ between tissues [7]. Differential coexpression analysis [34], [35] provides a tool to identify tissue-specific features of ciliated cells.

The differential coexpression analysis was performed by searching for genes that consistently occupied a central (i.e. hub) position [36] in the ciliary module in one tissue while being absent from the module in the other tissues – followed by testing for statistical significance (P<0.05, see Methods). The search identified 8 differentially coexpressed genes: 1 brain-specific (SLC47A2), 6 airway-specific (SIX1, CDH26, C1orf114, CCDC148, LRRC49, NAT1) and 1 fallopian tube-specific (HOXC4) (Table 6). The differential coexpression can be caused by differences in the transcriptome of ciliated cells or, alternatively, in the transcriptomes of other (“background”) cell types present in the samples [34]. To focus on the first component, we compared the differential coexpression results with changes in absolute expression levels of these genes between the tissues (Student's t-test between brain, airways and fallopian tubes based on the GSE7307 dataset, see Methods). 4 of the 8 genes showed a consistent tissue specificity profile: SLC47A2 was highly expressed in brain, SIX1 and CDH26 – in airways, HOXC4 – in fallopian tubes (Table 6, see also Table S11 for details). Additionally, among 3 of these genes present in the Human Protein Atlas, 2 were consistently differentially stained in ciliated cells between the tissues (SIX1 and CDH26, Table 6, see also Table S11). This suggests that, although modest, tissue-specific features may exist in the transcriptome of ciliated cells.

**Table 6:** Differentially coexpressed genes in the ciliary module.

Gene	Differential coexpression analysis				Validation	
	Brain	Airways	Fallopian tubes	P-value	Tissue specificity	Protein Atlas
SLC47A2	Hub (0.98)	-	-	6.6E-4	+	NA
SIX1	-	Hub (0.88)	-	3.4E-3	+	+
CDH26	-	Hub (0.76)	-	3.4E-3	+	+
C1orf114	-	Hub (0.87)	-	3.3E-3	−	−
CCDC148	-	Hub (0.89)	-	3.3E-3	−	NA
LRRC49	-	Hub (0.86)	-	1.7E-2	−	NA
NAT1	-	Hub (0.80)	-	1.0E-2	−	NA
HOXC4	-	-	Hub (0.84)	1.8E-2	+	NA

Mark ‘hub’ denotes that the gene represents a hub in the ciliary module in more than a half of the datasets for this tissue; mark ‘−’ indicates that the gene belongs to the ciliary module in none of the datasets pertaining to this tissue. Figures in brackets represent membership of a gene in the ciliary module (MM_ciliary_, see Methods), averaged across datasets of the tissue. P-values describe statistical significance of difference in MM_ciliary_ values for a gene between the tissues (ANOVA, Benjamini-Hochberg correction). In the validation columns, “+” indicates that the gene is supported as tissue specific by the respective analysis, “−” indicates that the results were non-supportive.

Among the identified genes, we further focused on SLC47A2. This gene is also known as MATE2 (multidrug and toxin extrusion transporter 2) [37], [38] and represents a transporter of small molecules across the plasma membrane [37]. MATE2 is highly expressed in kidney where it is considered to facilitate extrusion of drugs and metabolites from blood into urine [38]. Our data suggest that MATE2 is physically or functionally associated with cilia.

**Figure 6:**
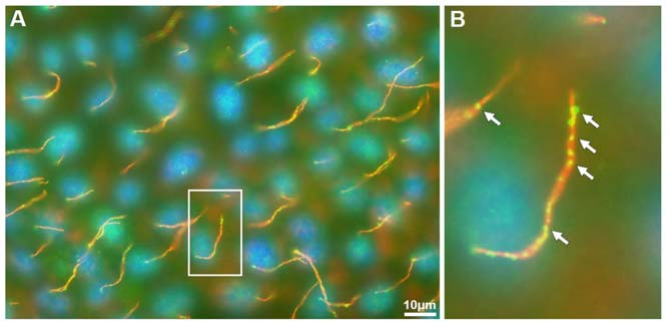
MATE2 co-localizes with acetylated α-tubulin in the primary cilia of MDCK cells. Immunofluorescence staining was performed with antibodies against MATE2 (green) and acetylated α-tubulin (red). Nuclei were stained with DAPI (blue). Co-localization of the antibody signals in the primary cilia is observed. **A** – MDCK immunofluorescence image; **B** - enlarged fragment of the image (marked with a white box in A). Arrows in B mark fluorescence from the antibodies specific to MATE2.

To explore this possibility, we performed an immunofluorescent staining of renal epithelial MDCK cells, an established model for studying cilia [39] with MATE2-specific antibodies. Figure 6 shows the co-staining of MDCK cells with antibodies against MATE2 (green) and acetylated α-tubulin, an established ciliary marker (red) [40]. A delicate signal for MATE2 overlapped with the signal of acetylated α-tubulin, specifically in the primary cilium compartment (Fig. 6). This supports our bioinformatics-based prediction that MATE2 has a previously unrecognized relation to cilia and suggests that this function is shared by primary and motile cilia.

## Discussion

### The consensus ciliary signature

Gene coexpression is well known to indicate functional cooperation between genes [36], [41] which is commonly used to search for genes with specific functions [17], [42]. Transcriptomic modules in tissue-level data were recently shown to include those corresponding to individual cell types [19]. This provides insight into quantitative variation of cellular composition across tissue specimen, thus performing an ‘*in silico* dissection’ of the tissue [19], [20]. Based on this approach, we explored the transcriptomes of brain, airways and fallopian tubes, and identified a coexpression module related to cells with motile cilia in these tissues. The module contained genes specifically expressed in such cells and likely to be functionally related to motile cilia.

Although the analyzed tissues consist of several cell types and are therefore expected to contain also primary cilia, the observed ciliary module is most likely to be driven by motile rather than primary cilia for several reasons. 1) Motile cilia are expected to be much more abundant in the analyzed tissues than primary cilia [5]. Therefore cross-sample variation in expression levels of genes associated with them are expected to be more robust that those associated with primary cilia. 2) Motile cilia cluster together in many copies on cells of a specific type (ciliated epithelial cells), while primary cilia are distributed in a single copy over a broad range of cells [5]. It is more likely that coordinate changes in expression are caused by variation related to a single cilia-rich cell type (ciliated epithelial cells) than by coordinate differences in biogenesis of the primary cilium over the whole tissue. 3) By definition, each coexpression module corresponds to a specific driving factor [18], [23]. Meanwhile, in all the datasets, the ciliary module was observed to contain markers associated with motility (e.g. radial spoke protein RSPH1, axonemal dyneins DNAH9, DNAH12, DNALI1, and others, Table S5). This suggests that the driving factor was related to motile rather than to primary cilia.

Although the identified consensus signature thus represents a signature of cells with motile cilia, this does not imply that all genes in this signature are specific to this type of cilia. Indeed, certain proteins are known to be shared by motile and primary cilia [5]. For example, the consensus signature contained IFT88 and IFT172 (Table S10) that mediate intraflagellar transport in both cilia types [5]. Furthermore, the signature included genes whose mutations are known to cause ciliopathies with dysfunctions of primary cilia (e.g. ARL6, LCA5, TMEM67) [43]. Despite their known relation to primary cilia, these proteins were confirmed as also linked to motile cilia by the Human Protein Atlas data (Table S9). Taken together, genes in the consensus signature tend to be functionally related to motile cilia – regardless of their potential additional roles in the other types of cilia and other cellular functions.

Although the consensus ciliary signature contained a broad range of known cilia-related genes, it did not cover the ciliome completely. Out of the 75 genes compiled by Gherman and colleagues as a representative set of established ciliary genes [14], 20 belonged to the signature. 30 other genes were absent from the signature but still were members of the ciliary module in at least one dataset. The remaining 25 genes were constitutively absent from the module, possibly due to their multifunctional nature (e.g. ubiquitous cytoskeletal proteins tubulins [14]), specificity to non-motile sensory cilia (e.g. serotonin receptors 1B and 2C [14]) or functional expression below the microarray detection limit. Overall, the ciliome coverage provided by the signature was similar to those obtained in the other ciliome studies.

The results of the tissue-specificity and Human Protein Atlas analyses suggest that the signature includes false positives. This is suggested by the fact that the percentage of genes in the classes A and B were lower among novel (52%) and predicted (62%) candidates than among known ciliary genes (81%) (Table 4). Interestingly, the main type of false-positives appears to be genes that are markers of epithelial cells regardless of whether these cells carry motile cilia or not (Table S9). This is potentially explained by certain correlation in abundance of all epithelial cells and ciliated epithelial cells across tissue samples. In total, approximately 28% of the signature proteins are estimated to belong to the class C (Table 4). Although this provides a false-positive rate estimate, some of these proteins may actually represent false negatives. Such proteins could fall into the class C due to insufficient specificity of antibodies to their target proteins [31]. This is supported by the fact that 62% of the class C proteins are suggested as cilia-related by the tissue specificity analysis (Table S10). Thus, the true false-positive rate is likely to be lower than the proportion of class C proteins in the Human Protein Atlas data.

Comparison of the consensus signature with the previous studies involved mapping of the human signature genes to their orthologs in model organisms (Table 3). These CilDB data allowed estimation of the conservation level of the signature genes. In this analysis, 45% of signature genes had a detectable ortholog in Drosophila, 42% – in Paramecium, and 37% – in Chlamydomonas (Table S6). These data are similar to those obtained in the previous studies [27], [29], [30] and support the view that, while key ciliary proteins (e.g. dyneins, Table S6) may be ubiquitous among ciliated organisms, the broader ciliome significantly differs in gene composition across species.

### Known and novel ciliary genes

The consensus signature included known ciliary genes from a wide range of functional classes (see Table S10 for the complete list of the signature genes): ciliary motor proteins (dyneins and kinesins), microtubule organization proteins (MAP6, MAP9, TPPP3, TTLL9), radial spoke components (RSPH1, RSPH4A, RSPH9), intraflagellar transport proteins (IFT172, IFT88), basal body-associated proteins (CEP97, CETN2, CSPP1), cilium biogenesis transcription factors (FOXJ1, RFX3), metabolic enzymes (AK7), signaling proteins (MAK, PROM1), and others. It also contained 60 genes that were previously reported as ciliary candidates with weak experimental evidence, and 74 novel genes, which are, to the best of our knowledge, suggested as related to cilia for the first time.

Literature data provide indications towards potential functions of the novel candidates in the cilium. For instance, CIB1 is known to be important for microtubule organization during cell division [44] and thus could be expected to play a role in the formation of microtubular cytoskeleton within cilia. BAIAP3 is related to exocytosis [45] and could be involved in transport of macromolecules between cytoplasm and the ciliary compartment. Phospholipase PLCH1 represents an inositol-1,3,5-phosphate (IP3)-producing enzyme [46]. Since IP(3) is known to affect frequency of the ciliary beat [47], the PLCH1 enzyme might be involved in the regulation of this process.

Human Protein Atlas described localization patterns for several ciliary candidates that were insufficiently characterized in the earlier studies. For instance, metabolic enzymes adenylate kinase 1 (AK1) and ribokinase (RBKS) are observed to have a strong cytoplasmic staining in cells with motile cilia and lack staining in non-ciliated cells (Table S9). The cytoplasmic staining restricted to ciliated cells, combined with the general functions of AK1 and RBKS, suggests involvement of these proteins in energy metabolism of cells with motile cilia. Several other proteins (B9D1, CCDC41, CLUAP1, TSGA10 and others, Table S9) were stained specifically in the apical region of cytoplasm in ciliated cells, suggesting their association with the basal region of cilia. An unusual localization pattern was demonstrated by a zinc finger-containing protein DZIP1L that was simultaneously stained in the ciliary compartment and nuclei of ciliated cells (Table S9). While this protein, related to Hedgehog signaling, is considered to be localized in the basal bodies of cilia [40], the Human Protein Atlas data indicate that DZIP1L might have broader functions that currently presumed.

### Dyslexia related genes in the signature

The literature mining approach revealed the consensus ciliary signature to include a group of genes related to the neurological disorder dyslexia (DCDC2, DYX1C1 and KIAA0319). There is also support for these proteins to be related to motile cilia by the tissue specificity and Human Protein Atlas data (Table S10). This finding is unexpected since no role for cilia in dyslexia is known. Similarly, the proteins themselves (DCDC2, DYX1C1 and KIAA0319) were not known to be related to cilia [33]. Nevertheless, since an overlap exists in protein composition of motile and primary cilia [5], proteins DCDC2, DYX1C1 and KIAA0319 might be related not only to motile (as suggested by the consensus signature, Table S10) but also to primary cilia which are present on a wide range of cell types, including neurons. At the time of this article submission, an independent study has been published demonstrating that overexpression of DCDC2 in neurons influences morphology and function of the primary cilium and the protein itself is localized to the primary cilium in neurons [48]. These data combined with the demonstration that DCDC2, DYX1C1 and KIAA0319 belong to the consensus ciliary signature (Table S10) raise the possibility that dysfunction of ciliary proteins may underlie dyslexia.

### MATE2 in the ciliome

Identification of MATE2 (SLC47A2) as a hub gene in the ciliary module in brain as well as the immunofluorescence results for MATE2 in the MDCK cell line suggest an association of MATE2 with cilia. MATE2 is known as a transporter protein involved in extrusion of positively charged small molecules (including drugs, such as metformin) from blood into the urine [49]. MATE2 was previously shown to be predominantly localized in the brush border of kidney proximal tubules which is an epithelial cell layer carrying microvilli [37]. While microvilli increase the surface of cellular plasma membrane, our data suggest that, at least in certain cell types, MATE2 can be localized in cilia, which also represent a cell protrusion organelle. The transport functions of MATE2 could potentially favor the cell protrusion localization. Our data also broaden the current view of the MATE2 tissue specificity. Thus, while MATE2 was previously proposed as a kidney-specific protein [37], our data suggest MATE2 to be also expressed in brain, specifically at the interface between the tissue and cerebrospinal fluid.

## Methods

### Expression data acquisition

The Gene Expression Omnibus repository [21] was searched for microarray expression datasets from brain, airways and reproductive tracts (human tissues containing the largest number of cells with motile cilia). The search was restricted to normal samples. Pathological samples, if present in the datasets, were excluded from the analysis. The search criteria ensured genome coverage and data robustness: each dataset contained measurements for at least 15,000 genes and at least 15 samples. If several of the found datasets shared a laboratory of origin, only the largest dataset was considered. After the filtering, 5 largest datasets were selected for each tissue. We downloaded the datasets from GEO using the Microarray Retriever web-tool [50]. For each brain dataset, we additionally created sub-datasets (with a minimum size of 15 samples) composed of samples from individual anatomical regions in order to control for biological differences between the regions [19], [22].

### Expression data normalization

Since most of the datasets shared their platform (Affymetrix U133Plus 2.0), we normalized them using an identical procedure. The normalization was based on custom CDF files (http://masker.nci.nih.gov/ev/) where non-specific and mis-targeted probes are masked [51]. The normalization was performed in R (http://cran.r-project.org/) using MAS5 algorithm (package “affy”) [52] followed by quantile normalization (package “DNAMR”). For other datasets (generated on Affymetrix 1.0 ST Array, Amersham Bioarray, Illumina HumanRef-8 and Illumina HumanRef-8 v2.0), we used the already normalized data from GEO since each of these platforms had been used to generate only one dataset used in this study.

### Coexpression networks analysis

In each dataset, gene coexpression networks analysis was performed independently from the other datasets. Because construction of coexpression networks at a genome scale is computationally intensive, in each dataset we randomly selected 4,000 genes for network analysis [22], [53] and next expanded the identified modules to the genome scale using a previously described heuristic procedure [24].

Gene coexpression networks were constructed using Weighted Gene Coexpression Networks Analysis (WGCNA) which is robust to noise and highlights consistent gene coexpression relationships [18], [23]. We started WGCNA by calculating Pearson correlations for all possible pairs of gene expression profiles. To mask weak correlations, the Pearson network was ‘weighted’ by raising each correlation to a power (the power value was chosen individually for each network according to the scale-free topology criterion) [18]. The weighting procedure strongly down-sizes low correlations, while only mildly affecting high-value correlations – a robust way of applying a soft threshold to the network [18]. According to WGCNA, the weighted network was next transformed into a network of Topological Overlap (TO) [18]. TO measures the extent to which two genes share their neighbours in the original network. Since this takes into account not only the correlation between the two genes themselves but also their correlations across the entire network, TO represents a more robust coexpression measure than pairwise correlation alone [18], [54]. The TO network was hierarchically clustered. The cluster dendrogram was split into modules using the Dynamic Tree Cut algorithm (minimal module size – 10 genes, the “deepSplit” mode – enabled) [55].

### Expanding modules to the genome scale

To expand the obtained modules from the 4,000 genes up to the genome-scale (>15,000 genes), we used a previously described procedure [24]. For each module we obtained a ‘*module eigengene*’ (ME) – a representative expression profile of the module [18]. Each ME was calculated by summarizing expression profiles of 10 genes with highest connectivity in the respective module (the top hub genes) [18], [24]. Such an ME described the module's dominating expression trend. The MEs were next used to assign genes to modules at a genome scale. Specifically, for each gene in the dataset we calculated Pearson correlation between its expression profile and MEs of the modules. These correlations measure the gene-to-module association strengths and are known as ‘*module membership*’ values (MM value for a gene with respect to a module) [18]. Finally, each gene was assigned to the module whose ME was most highly correlated with the gene's expression profile. Genes weakly correlated with all the MEs (all MM values<0.5) were assigned to none of the modules.

### FDR assessment in generation of the consensus signature

To estimate the number of genes expected to be members of the consensus signature by chance, we used a false-discovery rate (FDR) measure. In each dataset, we replaced genes in the ciliary module with randomly chosen genes while keeping the module sizes preserved. Using these permuted gene lists, we generated the respective consensus signatures (1000 iterations). The FDR was calculated as average size of the permutation-based signatures divided by the true consensus signature size.

### Analysis of the CilDB

To compare our consensus signature with the ciliary gene lists from the previous studies, we used CilDB version 2.0 (30 studies: http://cildb.cgm.cnrs-gif.fr/v2/page/ciliary_studies) [27]. From this database we extracted lists of ciliary genes determined in each study. The human signature genes were converted into lists of orthologs in the respective model organisms using the CilDB orthology maps (‘Inparanoid and Filtered Best Hit’ option) [27]. For each study, the list of signature orthologs was compared to the list of genes determined in the study using Fisher's exact test. Specifically, we evaluated the significance of the overlap taking the set of genes shared by the human's and model organism's genomes as a background.

To distinguish between known and novel ciliary genes in the signature, we characterized each gene by the number of previous studies in which it was detected with high, medium and low confidence according to CilDB. Genes in the signature were stratified into 3 categories: (1) known ciliary genes (high confidence in at least 1 study or medium confidence in at least 2 studies), (2) low-evidence ciliary candidates (medium confidence in one study or weak confidence in any number of studies) (3) novel ciliary candidates.

### Tissue specificity analysis

The multi-tissue human expression dataset GSE7307 was downloaded from the GEO database [21] (CEL files, Affymetrix U133 Plus 2.0 microarrays). From the total of 677 samples, we selected those representing normal tissues whereas samples corresponding to cell lines or disease-affected tissues were discarded. This resulted in selection of 502 samples for 104 tissues. The samples were normalized as described above. For each gene, expression levels were further transformed into Z-scores. To determine tissues in which the signature genes are highly expressed, we calculated mean expression level of the signature genes in each tissue and ranked the 104 tissues based on this value.

To obtain gene-specific estimates of tissue specificity, the 502 samples were ranked based on expression profiles of each individual gene. To evaluate a shift of samples obtained from tissues with motile cilia towards the top of the rankings, a permutation test was used. In this test, we calculated mean rank of such samples based on the real ranking and compared it to the null distribution of this quantity based on randomized rankings (10^6^ permutations). P-value was estimated as the fraction of values from the null distribution that were larger than the real value. Based on literature data, the following tissues were considered as containing cells with motile cilia: trachea, bronchus, lung, fallopian tubes, endometrium and testis. Since presence of cells with motile cilia in brain highly depends on the brain region, we excluded all brain samples from the analysis to avoid potential bias in P-value estimation. P-values were finally corrected for multiple testing (Bejnamini-Hochberg method, R package “multtest”).

### Protein localization

For each protein from the consensus signature, we searched the Protein Atlas database (http://www.proteinatlas.org/) (version 7.0) for images obtained by immunohistochemical staining of human tissues with protein-specific antibodies [31]. We downloaded images for 2 tissues with motile cilia where the staining results were most informative: airways (bronchus/nasopharynx) and fallopian tube. For airways, we used data from bronchus, while data from nasopharynx were used only if bronchus data were unavailable (nasopharynx images are marked with an asterisk in the Table S9). When several antibodies were available for a protein, we selected the antibody which best stained cilia in the tissues (the selection was kept constant for all tissues). Each protein was assigned to a class based on its staining in airways and fallopian tubes according to the following criteria. Class 1 (protein localized in cilia): in both tissues the staining is restricted to ciliated cells, and at least in one tissue is focused in the ciliary compartment. Class 2 (protein expressed in ciliated cells): in both tissues the staining is restricted to ciliated cells but not specifically to the ciliary compartment. Proteins of this class were further classified according to their subcellular localization: ‘Cilia and cytoplasm’, ‘Cytoplasm’, ‘Apical cytoplasm’, ‘Nucleus’, ‘Uncertain subcellular localization’. Class 3 (proteins expressed in both ciliated and non-ciliated cells, as well as proteins not expressed in ciliated cells at all): staining in at least one tissue is not specific to ciliated cells or absent in the tissue. Representative fragments from the full-size images were combined into a large collection (Table S9). Full size images are available in Protein Atlas online [31].

### Literature mining

From the consensus signature we selected 328 genes supported as ciliary by tissue specificity and/or Protein Atlas analyses. The gene list was analyzed in Anni (version 2.1, http://www.biosemantics.org/index.php?page=software) [32]. In this gene set, literature profiles were available for 103 genes, whereas the others were mentioned in less than 5 abstracts in MEDLINE and did not have a literature profile (the Anni database of abstracts was last updated on April 1, 2010). A literature profile of a given gene represents a list of terms (cell functions, diseases, drugs, etc.) that co-occur with name of this gene in abstracts. Terms in the profile are weighted to signify their importance based on normalized frequency of term co-occurrence with gene name [32]. Using Anni, we annotated the 103 genes list based on two alternative categories of terms: ‘Cell functions’ (3043 terms) and ‘Diseases and Syndromes’ (21,892 terms). The annotation procedure ranked terms from a given category by their contribution into overall correlation between the literature profiles, thus identifying prevailing features shared by the literature contexts of the genes [32]. In the two rankings (‘Cell functions’ and ‘Diseases and Syndromes’) we selected terms with contribution values above a threshold (>2% for cell function and >1% for disease terms). Terms representing ambiguous abbreviations (e.g. ‘PCD’ that can be recognized as ‘premature centromere division’ and ‘primary ciliary dyskinesia’) were removed.

### Differential coexpression analysis

Hub genes of the ciliary module were defined as genes that belong to this module and show an expression profile highly correlated with the ciliary module eigengene (MM_ciliary_≥0.75, see “Expanding modules to the genome scale”). To identify genes differentially coexpressed between brain, airways and fallopian tubes, we first selected genes that represented ciliary module hubs in one tissue (specifically, in more than half of datasets from a tissue) but did not belong to the ciliary module in any of the other tissue datasets. To ensure that the coexpression differences were statistically significant, for each gene from this list we compared MM_ciliary_ values between the tissues (ANOVA test based on Fisher-transformed MM_ciliary_ values). The correction for multiple testing across genes was performed using Benjamini-Hochberg method.

### Validation of differentially coexpressed genes

Genes identified as differentially coexpressed were tested using (1) expression tissue specificity and (2) Protein Atlas data. To test expression tissue specificity, we compared expression levels of the genes between brain (10 samples, see below), airways (7 samples) and fallopian tubes (3 samples) using Student's t-test based on microarray data from the GSE7307 dataset (Z-score normalized data, see “Tissue specificity analysis”). Because ependymal cells are known to be present in only a subset of brain regions [26], we selected an “ependyma-positive” subgroup of samples from the total of 193 brain samples available in the dataset: 10 samples with highest mean expression level of ciliary markers (genes from the signature that belonged to the ciliary module in all the 10 datasets were used as ciliary markers, Table S5, - all of them had been reported as ciliary in the previous studies). Using Student's t-test, we compared expression level of a given target gene between the candidate tissue and the union of the two other tissues (P<0.05, Table 6). Additionally, Protein Atlas was searched for immunostaining data corresponding to the differentially coexpressed genes [31].

### Immunofluorescence

MDCK cells were mildly fixed for 5 minutes with 0.4% paraformaldehyde at 37°C, subsequently treated with 0.5% TX-100 in PHEM buffer (50 mM PIPES, 50 mM HEPES, 10 mM EGTA and 10 mM MgCl2, pH 6.9) for 2 minutes at 37°C, followed by the fixation with methanol:aceton (1∶2) for 10 minutes at 4°C. Immunostaining for MATE2 was performed using the SLC47A2 antibody (rabbit, Abcam, Ab105050, 1∶500) and goat anti-rabbit IgG (H+L) (Alexa Fluor® 488, Invitrogen, A11008, 1∶400). The cilium was immunostained with the antibody specific for acetylated α tubulin (mouse, Sigma-Aldrich T6793, clone 6-11B-1, 1∶200) and goat anti-mouse IgG (H+L) (Alexa Fluor® 594, Invitrogen, A11005, 1∶1000). Nuclei were stained with DAPI.

## Supporting Information

Table S1 **Data description.** The table contains descriptions of microarray samples from the GEO database. Each spread sheet corresponds to one experiment from the GEO database. The first 5 spread sheets correspond to brain, the next 5 – to airways, the final 2 – to female reproductive tracts (column “Tissue type”). The spread sheets mention all samples from the respective datasets, including pathological samples (column “Pathological state”). The “Inclusion Status” column specifies which samples were included into a given network. Since pathological samples were excluded from the networks construction process, they are marked as “Removed”. Note that, since brain datasets GSE15745 and GSE11882 contained more than 15 samples per brain anatomical region, the spread sheets corresponding to these datasets additionally mark samples that were used for construction of region-specific networks (column “Inclusion Status”).
(XLS)

Table S2 **Functional annotation of all modules.** Column “Module size” describes numbers of genes in each module (after extension of the modules' gene composition from the 4,000 seed genes to the genome scale). Column “DAVID annotation” specifies top scoring annotation term (as measured by enrichment P-value) that functionally characterizes a given module according to the DAVID web tool. Original P-values are shown in brackets. P-values that remain≤0.05 after the Benjamini-Hochberg correction are marked with an asterisk. Column “Ciliary markers” provides P-values that measure enrichment of modules with the golden-standard ciliary markers from the Gherman's list (Fisher's exact test). Modules significantly enriched with the ciliary markers are marked green. “NS” stands for “non-significant” (P>0.05).
(XLS)

Table S3 **Gene composition of the ciliary modules.** For each network a list of genes in the ciliary module is provided (genome scale analysis). The “Module membership” column provides Pearson correlations between expression profile of a given gene and integrated eigengene of the ciliary module (see Methods). This measure ranks genes based on their proximity to the center of the ciliary module (hub position).
(XLS)

Table S4 **Cross-networks modules similarity.** The first table describes similarity of the ciliary module in each dataset to the ciliary modules in the other datasets. The other 3 tables describe similarity of the ciliary module in each dataset to non-ciliary modules in the other datasets. Because each dataset contains many non-ciliary modules, the second table provides median similarity values, the third table – highest similarity values, and the fourth table – lowest similarity values (across all non-ciliary modules within a given dataset). In each of the four tables, the top-right corner of the matrix provides Fisher's exact test P-values describing significance of gene overlap between the modules. The bottom-left corner provides corresponding percentages of gene overlap (100% stands for the size of the smaller module in each pair).
(XLS)

Table S5 **Number of datasets that support each gene as belonging to the ciliary module.** The “Consensus signature genes” spread sheet provides data for genes from the consensus signature. The “Rest of the genes” spread sheet provides data for genes that belonged to the ciliary module in at least one dataset but were not included to the consensus signature. For each gene-dataset pair, “1” denotes membership of the gene in the ciliary module, “0” – absence of the gene in the ciliary module. The “Total positive datasets” column describes number of datasets that support the gene as belonging to the ciliary module (sum across the previous columns). The “Tissues” column provides number of tissues (brain, airways, reproductive tracts) in which the gene belongs to the ciliary module in at least one dataset.
(XLS)

Table S6 **Orthology information and overlap of the consensus signature with the previous studies.** Spreadsheet “Orthology information”: for each gene, orthologs in the model organisms are specified. The orthology information was extracted from the CilDB database. Spreadsheet “Comparison with prev. studies”: overlap of the consensus signature with ciliary genes lists from the previous studies. Columns in this table correspond to those in the Table 3 of the main text (see Results).
(XLS)

Table S7 **Stratification of the signature genes into novelty categories.** For each gene, the table specifies number of ciliome studies in which the gene was detected with a strong/medium/weak evidence (according to the CilDB evidence codes). The “MEDLINE” column specifies whether the gene is described as potentially related to cilia in MEDLINE abstracts. The “Category” column provides the resulting assignment of the gene to a novelty category: strong evidence from the previous studies, weak evidence from the previous studies, no evidence from the previous studies.
(XLS)

Table S8 **Tissue specificity of the signature genes.** For each gene, a p-value is provided estimating significance of the gene's up-regulation in ciliated tissues compared to the rest of the tissues in the human body (see Methods). The “Top 15 tissue samples” column specifies the 15 samples with highest expression levels of the gene. The samples are listed in a decreasing order of the gene's expression level and were selected from the total of 309 samples in GSE7307 (see Methods). Some of the tissues are represented by more than one sample since GSE7307 contained biological replicates.
(XLS)

Table S9 **Immunostaining images from Human Protein Atlas.** Proteins are grouped by novelty categories: known ciliary proteins (category I), previously predicted candidates (category II) and novel candidates (category III). The summary at the top of the document briefly describes the staining pattern of each protein in the tissues and specifies the resulting staining class. The immunohistochemical images underlying the protein classification are provided below the summary table. The “Protein” column specifies protein name, antibody ID and novelty category of each protein. The “Airways” and “Fallopian tubes” columns provide Protein Atlas images for the respective tissues. In the “Airways” column, images marked with an asterisk correspond to nasopharynx, while the rest of the images in this column – to bronchus. All images in the table have been cropped out from larger images in the Protein Atlas in order to enable their compilation into a collection. The full size images can be found in the Human Protein Atlas database.
(PDF)

Table S10 **Data summary for the signature genes.** For each signature gene, this table summarizes data from the Tables S5, S7, S8 and S9.
(XLS)

Table S11 **Differential coexpression analysis between the tissues.** (1) The “Differential coexpression” spread sheet contains only the differentially coexpressed genes. For each gene, the table specifies its membership (MM) in the ciliary module in each dataset. Module membership was calculated as Pearson correlation between expression profile of a gene and integrated expression profile of the ciliary module (see Methods, section “Expanding modules to the genome scale”). Green indicates that the gene belongs to the ciliary module in a given dataset. Genes that belong to the ciliary module and have MM value >0.75 were considered hubs in the respective dataset. For a given gene, statistical significance of MM difference between the tissues is described by ANOVA P-value. (2) The “Tissue specificity” spread sheet describes validation of the differentially coexpressed genes based on the GSE7307 dataset. The table shows expression levels of the genes in 10 “ependyma-positive” brain samples, 7 airway samples and 3 fallopian tube samples. For each gene, mean expression level in the tissues is provided. Tissue with the highest expression level of each gene is colored based on centrality status of the gene in this tissue. Specifically, green denotes that the gene represents a hub in this tissue's ciliary module, grey denotes that the gene represents a hub in a different tissue's ciliary module. For each gene up-regulated in the same tissue where the gene represents a ciliary hub, statistical significance of up-regulation is provided (Student's t-test P-value after a Benjamini-Hochberg correction). For genes supported as tissue-specific markers of ciliated cells, the underlying tissue specificity profile is visualized as a histogram. (3) The “Protein Atlas” spread sheet describes validation of the differentially coexpressed genes with immunostaining data. Human Protein Atlas contained data for 3 of the 8 differentially coexpressed genes. Since ependyma is absent from Protein Atlas, we compared the immunostaining data between airways (bronchus and nasopharynx) and fallopian tubes.
(XLS)
