# Supplementary material for: Exploring the Transcriptome of Ciliated Cells Using In Silico Dissection of Human Tissues
Source: PLoS One. 2012 Apr 25;7(4):e35618. doi: 10.1371/journal.pone.0035618 (PMC3338421; doi:10.1371/journal.pone.0035618)
Supplement: Table S9 — Immunostaining images from Human Protein Atlas. Proteins are grouped by novelty categories: known ciliary proteins (category I), previously predicted candidates (category II) and novel candidates (category III). The summary at the top of the document briefly describes the staining pattern of each protein in the tissues and specifies the resulting staining class. The immunohistochemical images underlying the protein classification are provided below the summary table. The “Protein” column specifies protein name, antibody ID and novelty category of each protein. The “Airways” and “Fallopian tubes” columns provide Protein Atlas images for the respective tissues. In the “Airways” column, images marked with an asterisk correspond to nasopharynx, while the rest of the images in this column – to bronchus. All images in the table have been cropped out from larger images in the Protein Atlas in order to enable their compilation into a collection. The full size images can be found in the Human Protein Atlas database. (PDF) [file pone.0035618.s009.pdf]

Analysis of protein localization

Summary

Symbol	Airways	Fallopian tubes	Staining Class
			Category 1
AGBL2	Cilia and cytoplasm	Cilia	(1) Staining is specific to cilia
AK7	Cilia	Cilia	(1) Staining is specific to cilia
ANKRD45	Cilia	Cilia	(1) Staining is specific to cilia
ARMC3	Cilia	Cilia	(1) Staining is specific to cilia
ARMC4	Cilia	Cilia	(1) Staining is specific to cilia
ARL6	Cilia	Cilia	(1) Staining is specific to cilia
C1orf158	Cilia	Cilia	(1) Staining is specific to cilia
C10orf63	Cilia	Cilia	(1) Staining is specific to cilia
C20orf26	Cilia	Cilia	(1) Staining is specific to cilia
C21orf59	Cilia and cytoplasm	Cilia	(1) Staining is specific to cilia
C3orf15	Cilia and cytoplasm	Cilia	(1) Staining is specific to cilia
C7orf57	Cilia	Cilia	(1) Staining is specific to cilia
C7orf67	Cilia	Cilia	(1) Staining is specific to cilia
C8orf47	Cilia	Cilia	(1) Staining is specific to cilia
C9orf116	Cilia	Cilia	(1) Staining is specific to cilia
C9orf117	Cilia	Cilia	(1) Staining is specific to cilia
C9orf98	Cilia	Cilia	(1) Staining is specific to cilia
CCDC104	Cilia	Cilia	(1) Staining is specific to cilia
CCDC146	Cilia and cytoplasm	Cilia	(1) Staining is specific to cilia
CCDC147	Cilia	Cilia	(1) Staining is specific to cilia
CCDC39	Cilia	Cilia	(1) Staining is specific to cilia
CCDC40	Cilia	Cilia	(1) Staining is specific to cilia
CETN2	Cilia and cytoplasm	Cilia	(1) Staining is specific to cilia
CYB5D1	Cilia and cytoplasm	Cilia	(1) Staining is specific to cilia
DCDC2	Apical cytoplasm	Cilia	(1) Staining is specific to cilia
DLEC1	Cilia	Cilia	(1) Staining is specific to cilia
DNAH5	Cilia	Cilia	(1) Staining is specific to cilia
DNAH7	Cilia	Cilia	(1) Staining is specific to cilia
DNAH9	Cilia	Cilia	(1) Staining is specific to cilia
DNAH12	Cilia	Cilia	(1) Staining is specific to cilia
DNAI1	Cilia	Cilia	(1) Staining is specific to cilia
DNAI2	Cilia	Cilia	(1) Staining is specific to cilia
DNALI1	Cilia	Cilia	(1) Staining is specific to cilia
DYDC2	Cilia	Cilia	(1) Staining is specific to cilia
EFCAB1	Cilia	Cilia	(1) Staining is specific to cilia
EFCAB6	Cilia	Cilia	(1) Staining is specific to cilia
EFHB	Cilia	Cilia	(1) Staining is specific to cilia
EFHC1	Cilia and cytoplasm	Cilia	(1) Staining is specific to cilia
EFHC2	Cilia	Cilia	(1) Staining is specific to cilia
FLJ23834	Cilia	Cilia	(1) Staining is specific to cilia

IQUB	Apical cytoplasm	Cilia	(1) Staining is specific to cilia
KIF9	Cilia	Cilia	(1) Staining is specific to cilia
LCA5	Cilia	Cilia	(1) Staining is specific to cilia
LRRC23	Cilia	Cilia	(1) Staining is specific to cilia
LRRC34	Cilia	Cilia	(1) Staining is specific to cilia
LRRC48	Cilia	Cilia	(1) Staining is specific to cilia
MAP6	Cytoplasm	Cilia	(1) Staining is specific to cilia
MDH1B	Cilia	Cilia	(1) Staining is specific to cilia
MLF1	Cilia	Cilia	(1) Staining is specific to cilia
MORN5	Cilia	Cilia	(1) Staining is specific to cilia
MS4A8B	Cilia	Cilia	(1) Staining is specific to cilia
PPIL6	Cilia	Cilia	(1) Staining is specific to cilia
PROM1	Cilia	Cilia	(1) Staining is specific to cilia
RIBC2	Cilia	Cilia	(1) Staining is specific to cilia
ROPN1L	Cytoplasm	Cilia	(1) Staining is specific to cilia
RPGR	Cilia	Cilia	(1) Staining is specific to cilia
RSPH1	Cilia	Cilia	(1) Staining is specific to cilia
RSPH4A	Cilia	Cilia	(1) Staining is specific to cilia
RSPH9	Cilia	Cilia	(1) Staining is specific to cilia
RTDR1	Cilia	Cilia	(1) Staining is specific to cilia
SPA17	Cilia	Cilia	(1) Staining is specific to cilia
SPAG6	Cilia	Cilia	(1) Staining is specific to cilia
SPATA17	Cilia	Cilia	(1) Staining is specific to cilia
SPEF2	Cilia	Cilia	(1) Staining is specific to cilia
SRGAP3	Cytoplasm	Cilia	(1) Staining is specific to cilia
STOML3	Cilia	Cilia	(1) Staining is specific to cilia
TCTEX1D1	Cilia	Cilia	(1) Staining is specific to cilia
TEKT2	Cilia	Cilia	(1) Staining is specific to cilia
TEX9	Cilia	Cilia	(1) Staining is specific to cilia
TTC18	Cilia	Cilia	(1) Staining is specific to cilia
TTC25	Cilia	Cilia	(1) Staining is specific to cilia
TTC26	Apical cytoplasm	Cilia	(1) Staining is specific to cilia
TTC29	Cilia and cytoplasm	Cilia	(1) Staining is specific to cilia
WDR16	Cilia	Cilia	(1) Staining is specific to cilia
YSK4	Cilia	Cilia	(1) Staining is specific to cilia
ZBBX	Cilia	Cilia	(1) Staining is specific to cilia
AK1	No staining	Cilia and cytoplasm	(2) Staining is specific to ciliated cells (cilia and cytoplasm)
B9D1	Apical cytoplasm	Apical cytoplasm	(2) Staining is specific to ciliated cells (apical region of cytoplasm)
C1orf102	Cytoplasm	Cytoplasm	(2) Staining is specific to ciliated cells (cytoplasm)
C11orf60	Apical cytoplasm	Apical cytoplasm	(2) Staining is specific to ciliated cells (apical region of cytoplasm)
C13orf30	Apical cytoplasm	Apical cytoplasm	(2) Staining is specific to ciliated cells (apical region of cytoplasm)
C6orf118	Cytoplasm	Apical cytoplasm	(2) Staining is specific to ciliated cells (uncertain subcellular localization)
C9orf135	Cytoplasm	Apical cytoplasm	(2) Staining is specific to ciliated cells (uncertain subcellular localization)
C2CD3	Apical cytoplasm	Apical cytoplasm	(2) Staining is specific to ciliated cells (apical cytoplasm)
CCDC41	Apical cytoplasm	Apical cytoplasm	(2) Staining is specific to ciliated cells (apical region of cytoplasm)
CDS1	Cytoplasm	Cytoplasm	(2) Staining is specific to ciliated cells (cytoplasm)
CEP97	Apical cytoplasm	Apical cytoplasm	(2) Staining is specific to ciliated cells (apical region of cytoplasm)

CLGN	Cytoplasm	Cytoplasm	(2) Staining is specific to ciliated cells (cytoplasm)
CLUAP1	Apical cytoplasm	Apical cytoplasm	(2) Staining is specific to ciliated cells (apical region of cytoplasm)
DPCD	Cytoplasm	Cytoplasm	(2) Staining is specific to ciliated cells (cytoplasm)
DZIP1L	Cilia and nucleus	Cilia and nucleus	(2) Staining is specific to ciliated cells (cilia and nucleus)
FOXJ1	Nucleus	Nucleus	(2) Staining is specific to ciliated cells (nucleus)
HSP90AA1	Cytoplasm	Cytoplasm	(2) Staining is specific to ciliated cells (cytoplasm)
HSPB11	Cilia & apical cytopl.	Cilia & apical cytopl.	(2) Staining is specific to ciliated cells (cilia and apical cytoplasm)
MAK	Cilia and cytoplasm	Cilia and cytoplasm	(2) Staining is specific to ciliated cells (cilia and cytoplasm)
MAP9	Apical cytoplasm	Apical cytoplasm	(2) Staining is specific to ciliated cells (apical cytoplasm)
PTGES3	Cytoplasm	Cytoplasm	(2) Staining is specific to ciliated cells (cytoplasm)
RFX3	Nucleus	Nucleus	(2) Staining is specific to ciliated cells (nucleus)
SCGB2A1	Cytoplasm	Cilia and cytoplasm	(2) Staining is specific to ciliated cells (uncertain subcellular localization)
SLC22A4	Cilia and cytoplasm	Cilia and cytoplasm	(2) Staining is specific to ciliated cells (cilia and cytoplasm)
SLC27A2	Cilia and cytoplasm	Cytoplasm	(2) Staining is specific to ciliated cells (uncertain subcellular localization)
SPAG16	Cytoplasm	Cytoplasm	(2) Staining is specific to ciliated cells (cytoplasm)
SPATA6	Apical cytoplasm	Apical cytoplasm	(2) Staining is specific to ciliated cells (apical region of cytoplasm)
STK33	Apical cytoplasm	Cytoplasm	(2) Staining is specific to ciliated cells (uncertain subcellular localization)
TMEM67	Apical cytoplasm	Apical cytoplasm	(2) Staining is specific to ciliated cells (apical region of cytoplasm)
TRAF3IP1	Apical cytoplasm	Apical cytoplasm	(2) Staining is specific to ciliated cells (apical region of cytoplasm)
TSGA10	Apical cytoplasm	Apical cytoplasm	(2) Staining is specific to ciliated cells (apical region of cytoplasm)
TTLL6	Apical cytoplasm	Apical cytoplasm	(2) Staining is specific to ciliated cells (apical region of cytoplasm)
VWA3B	Nucleus & apical cyto	Nucleus & apical cyto	(2) Staining is specific to ciliated cells (nucleus & apical region of cytoplasm)
ZNF474	Apical cytoplasm	Apical cytoplasm	(2) Staining is specific to ciliated cells (apical region of cytoplasm)
CAT	Non-specific	Non-specific	(3) Staining is non-specific to ciliated cells or absent
CCDC17	Cytoplasm	Non-specific	(3) Staining is non-specific to ciliated cells or absent
CCDC65	Cilia and cytoplasm	Non-specific	(3) Staining is non-specific to ciliated cells or absent
C14orf45	Cytoplasm	Non-specific	(3) Staining is non-specific to ciliated cells or absent
C14orf50	Cytoplasm	Non-specific	(3) Staining is non-specific to ciliated cells or absent
C2orf40	Non-specific	Non-specific	(3) Staining is non-specific to ciliated cells or absent
CHST9	Non-specific	Non-specific	(3) Staining is non-specific to ciliated cells or absent
DNAJA4	Cytoplasm	Non-specific	(3) Staining is non-specific to ciliated cells or absent
GMPR	Cytoplasm	Non-specific	(3) Staining is non-specific to ciliated cells or absent
HKDC1	Non-specific	Non-specific	(3) Staining is non-specific to ciliated cells or absent
IK	Cytoplasm	Non-specific	(3) Staining is non-specific to ciliated cells or absent
IQCH	Non-specific	Non-specific	(3) Staining is non-specific to ciliated cells or absent
KIF21A	Non-specific	Non-specific	(3) Staining is non-specific to ciliated cells or absent
LRRC46	Cytoplasm	Non-specific	(3) Staining is non-specific to ciliated cells or absent
MYB	Non-specific	Non-specific	(3) Staining is non-specific to ciliated cells or absent
NME7	Cytoplasm	Non-specific	(3) Staining is non-specific to ciliated cells or absent
NSUN7	Cytoplasm	Non-specific	(3) Staining is non-specific to ciliated cells or absent
PECR	Non-specific	Non-specific	(3) Staining is non-specific to ciliated cells or absent
PTPRT	Cytoplasm	Non-specific	(3) Staining is non-specific to ciliated cells or absent
RAGE	Cytoplasm	Non-specific	(3) Staining is non-specific to ciliated cells or absent
RIBC1	Cytoplasm	Non-specific	(3) Staining is non-specific to ciliated cells or absent
SERPINI2	Cytoplasm	Non-specific	(3) Staining is non-specific to ciliated cells or absent
TMEM190	Non-specific	Non-specific	(3) Staining is non-specific to ciliated cells or absent
TTC21A	Non-specific	Non-specific	(3) Staining is non-specific to ciliated cells or absent

WDR63	Non-specific	Non-specific	(3) Staining is non-specific to ciliated cells or absent
ZMYND10	Cytoplasm	Non-specific	(3) Staining is non-specific to ciliated cells or absent
**Category 2**			
C1orf87	Cilia	Cilia	(1) Staining is specific to cilia
C1orf222	Cilia	Cilia	(1) Staining is specific to cilia
C11orf66	Cilia	Cilia	(1) Staining is specific to cilia
C14orf179	Cilia	Cilia	(1) Staining is specific to cilia
C9orf9	Cilia	Cilia	(1) Staining is specific to cilia
DZIP3	Cilia	Cilia	(1) Staining is specific to cilia
FSD1L	Cilia	Cilia	(1) Staining is specific to cilia
KCNRG	Cilia	Cilia	(1) Staining is specific to cilia
LRP2BP	Cilia	Cilia	(1) Staining is specific to cilia
MIPEP	Cilia	Cilia	(1) Staining is specific to cilia
PLCH1	Cilia and cytoplasm	Cilia	(1) Staining is specific to cilia
SPAG17	Cilia	Cilia	(1) Staining is specific to cilia
UBXN10	Cilia and cytoplasm	Cilia	(1) Staining is specific to cilia
C6orf103	Apical cytoplasm	Cytoplasm	(2) Staining is specific to ciliated cells (uncertain subcellular localization)
FANK1	Cilia and cytoplasm	Cilia and cytoplasm	(2) Staining is specific to ciliated cells (cilia and cytoplasm)
LRRC6	Cilia and cytoplasm	Cytoplasm	(2) Staining is specific to ciliated cells (uncertain subcellular localization)
NEK10	Cytoplasm	Apical cytoplasm	(2) Staining is specific to ciliated cells (uncertain subcellular localization)
PPP1R16A	Apical cytoplasm	Apical cytoplasm	(2) Staining is specific to ciliated cells (apical cytoplasm)
RBKS	Cilia and cytoplasm	Cilia and cytoplasm	(2) Staining is specific to ciliated cells (cilia and cytoplasm)
SPATA18	Apical cytoplasm	Apical cytoplasm	(2) Staining is specific to ciliated cells (apical cytoplasm)
WDR49	Apical cytoplasm	Apical cytoplasm	(2) Staining is specific to ciliated cells (apical cytoplasm)
APOBEC4	Apical cytoplasm	Non-specific	(3) Staining is non-specific to ciliated cells or absent
C3orf25	Cytoplasm	Non-specific	(3) Staining is non-specific to ciliated cells or absent
CCDC60	Non-specific	Non-specific	(3) Staining is non-specific to ciliated cells or absent
FAM179A	Cytoplasm	Non-specific	(3) Staining is non-specific to ciliated cells or absent
KIAA1377	Cytoplasm	Non-specific	(3) Staining is non-specific to ciliated cells or absent
KLHDC9	Cilia and cytoplasm	Non-specific	(3) Staining is non-specific to ciliated cells or absent
NEK11	Non-specific	Non-specific	(3) Staining is non-specific to ciliated cells or absent
SLFN13	Non-specific	Non-specific	(3) Staining is non-specific to ciliated cells or absent
SPATA4	Cytoplasm	Non-specific	(3) Staining is non-specific to ciliated cells or absent
STOX1	Cytoplasm	Non-specific	(3) Staining is non-specific to ciliated cells or absent
TMEM17	Non-specific	Non-specific	(3) Staining is non-specific to ciliated cells or absent
WDR31	Cytoplasm	Non-specific	(3) Staining is non-specific to ciliated cells or absent
ZNF20	Cytoplasm	Non-specific	(3) Staining is non-specific to ciliated cells or absent
**Category 3**			
ARMC2	Cilia and cytoplasm	Cilia	(1) Staining is specific to cilia
BAIAP3	Cilia and cytoplasm	Cilia	(1) Staining is specific to cilia
C1orf92	Cilia and cytoplasm	Cilia	(1) Staining is specific to cilia
C10orf92	Cilia	Cilia	(1) Staining is specific to cilia
C11orf63	Cilia	Cilia	(1) Staining is specific to cilia
C21orf58	Apical cytoplasm	Cilia	(1) Staining is specific to cilia
C22orf23	Cilia	Cilia	(1) Staining is specific to cilia
CCDC89	Cilia	Cilia	(1) Staining is specific to cilia
CIB1	Cilia	Apical cytoplasm	(1) Staining is specific to cilia

IQCK	Cilia	Cilia	(1) Staining is specific to cilia
KIAA0319	Cilia	Cilia	(1) Staining is specific to cilia
LPAR3	Cilia	Cilia	(1) Staining is specific to cilia
LRGUK	Cilia	Cilia	(1) Staining is specific to cilia
LRRC18	Cilia	Cilia	(1) Staining is specific to cilia
LRRIQ3	Apical cytoplasm	Cilia	(1) Staining is specific to cilia
NUP62CL	Cilia and cytoplasm	Cilia	(1) Staining is specific to cilia
PPM1E	Cilia	Cilia	(1) Staining is specific to cilia
RBM20	Cilia	Cilia	(1) Staining is specific to cilia
RGS22	Cytoplasm	Cilia	(1) Staining is specific to cilia
UFC1	Cytoplasm and cilia	Cilia	(1) Staining is specific to cilia
ACYP1	Apical cytoplasm	Cilia and cytoplasm	(2) Staining is specific to ciliated cells (uncertain subcellular localization)
C1orf129	Apical cytoplasm	Apical cytoplasm	(2) Staining is specific to ciliated cells (apical cytoplasm)
DCDC5	Apical cytoplasm	Apical cytoplasm	(2) Staining is specific to ciliated cells (apical cytoplasm)
FLJ16686	Apical cytoplasm	Apical cytoplasm	(2) Staining is specific to ciliated cells (apical cytoplasm)
SYTL3	Apical cytoplasm	Apical cytoplasm	(2) Staining is specific to ciliated cells (apical cytoplasm)
ALS2CR12	Non-specific	Non-specific	(3) Staining is non-specific to ciliated cells or absent
ANKFN1	Cytoplasm	Non-specific	(3) Staining is non-specific to ciliated cells or absent
CCDC67	Cytoplasm	Non-specific	(3) Staining is non-specific to ciliated cells or absent
C9orf103	Non-specific	Non-specific	(3) Staining is non-specific to ciliated cells or absent
C10orf57	Non-specific	Non-specific	(3) Staining is non-specific to ciliated cells or absent
FABP6	Non-specific	Non-specific	(3) Staining is non-specific to ciliated cells or absent
FAM174A	Non-specific	Non-specific	(3) Staining is non-specific to ciliated cells or absent
GLT8D1	Cytoplasm	Non-specific	(3) Staining is non-specific to ciliated cells or absent
GRAMD2	Non-specific	Non-specific	(3) Staining is non-specific to ciliated cells or absent
IL5RA	Cytoplasm	Non-specific	(3) Staining is non-specific to ciliated cells or absent
KCNMB2	Non-specific	Non-specific	(3) Staining is non-specific to ciliated cells or absent
KIAA0556	Non-specific	Non-specific	(3) Staining is non-specific to ciliated cells or absent
LCA5L	Non-specific	Non-specific	(3) Staining is non-specific to ciliated cells or absent
MARCH10	Non-specific	Non-specific	(3) Staining is non-specific to ciliated cells or absent
NELL2	Non-specific	Non-specific	(3) Staining is non-specific to ciliated cells or absent
PERP	Non-specific	Non-specific	(3) Staining is non-specific to ciliated cells or absent
SNCAIP	Cytoplasm	Non-specific	(3) Staining is non-specific to ciliated cells or absent
TIGD4	Non-specific	Non-specific	(3) Staining is non-specific to ciliated cells or absent
TMEM146	Non-specific	Non-specific	(3) Staining is non-specific to ciliated cells or absent
TSPAN6	Cytoplasm	Non-specific	(3) Staining is non-specific to ciliated cells or absent
ULK4	Non-specific	Non-specific	(3) Staining is non-specific to ciliated cells or absent
WRB	Non-specific	Non-specific	(3) Staining is non-specific to ciliated cells or absent
XK	Non-specific	Non-specific	(3) Staining is non-specific to ciliated cells or absent

Airway images marked with an asterisk (below) were taken from nasopharynx.
The rest of the airway images were taken from bronchus.

Category 1

(genes with strong evidence

for ciliary function from the *literature*)

Coverage: 136 proteins were available in Protein Atlas
from the total of 237 proteins in the category

Protein	Airways	Fallopian tubes	Summary
AGBL2 (antibody HPA007718)<br><br>Category 1	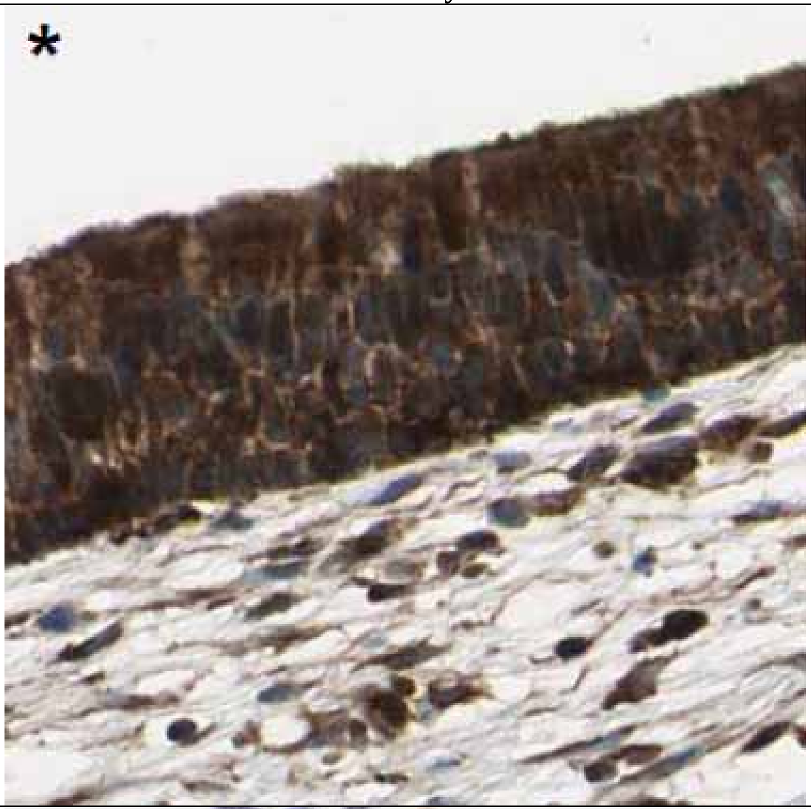	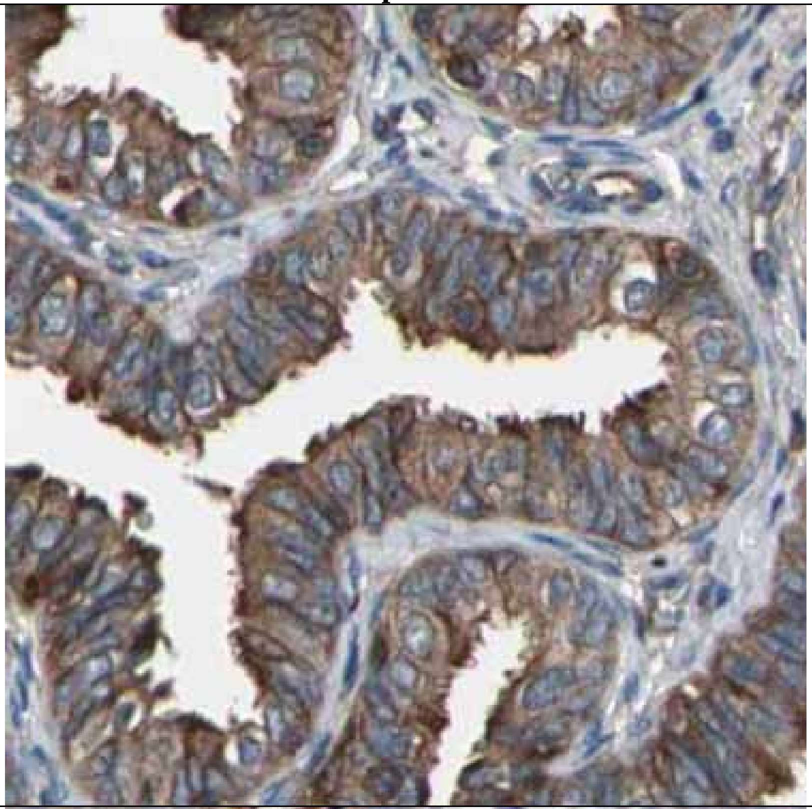	Staining is specific to *cilia*
AK7 (antibody HPA003543)<br><br>Category 1	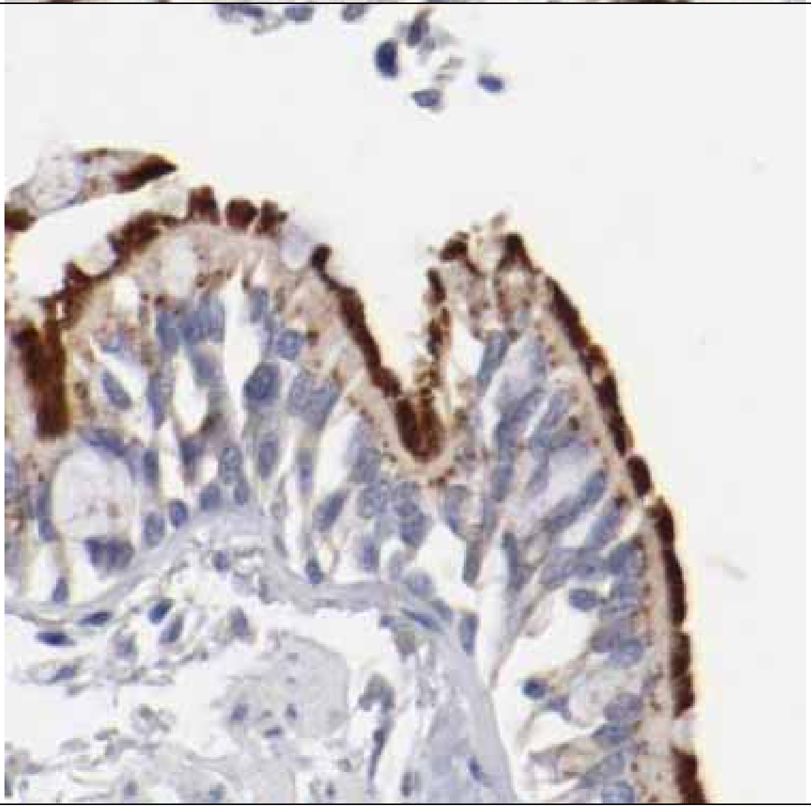	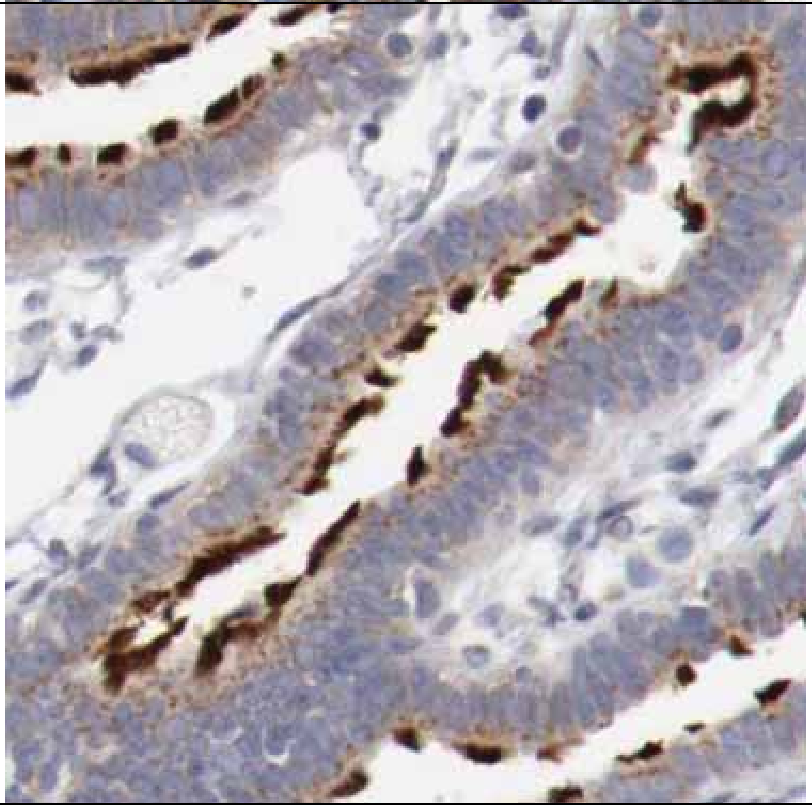	Staining is specific to *cilia*

ANKRD45 (antibody HPA031657)<br><br>Category 1	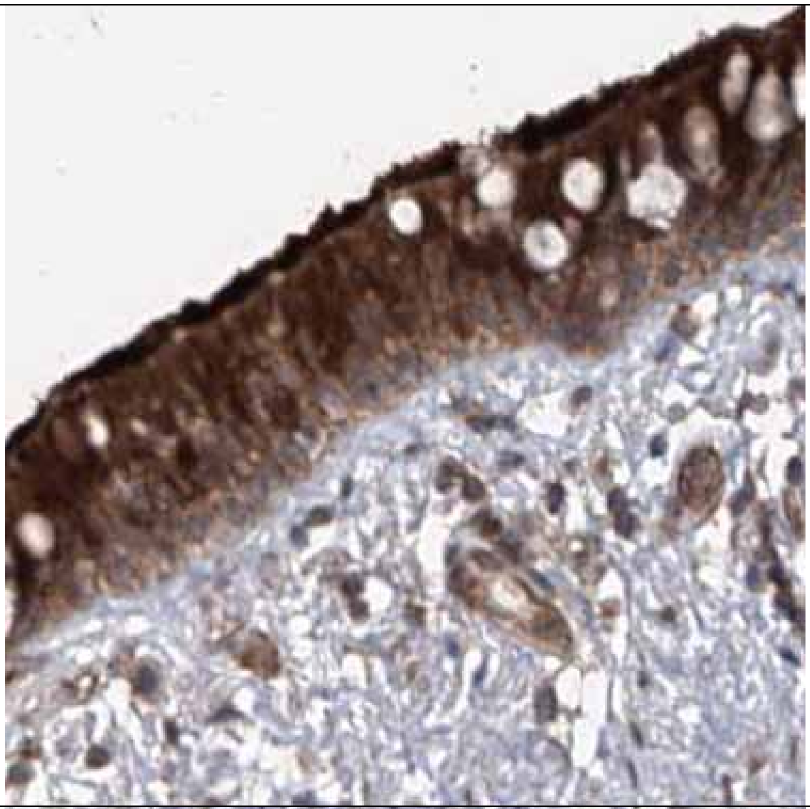	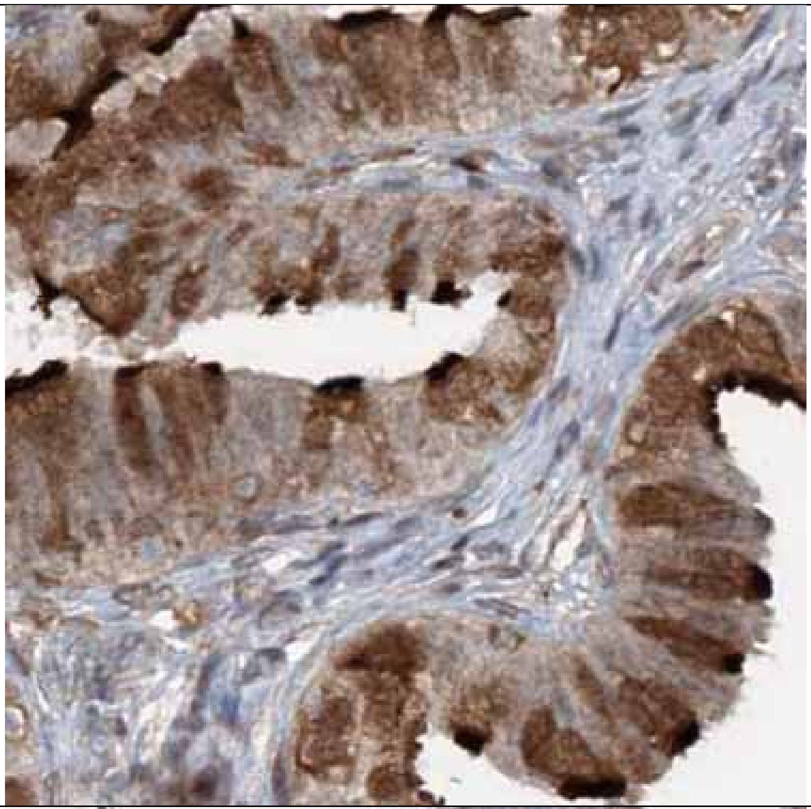	Staining is specific to *cilia*
ARMC3 (antibody HPA037824)<br><br>Category 1	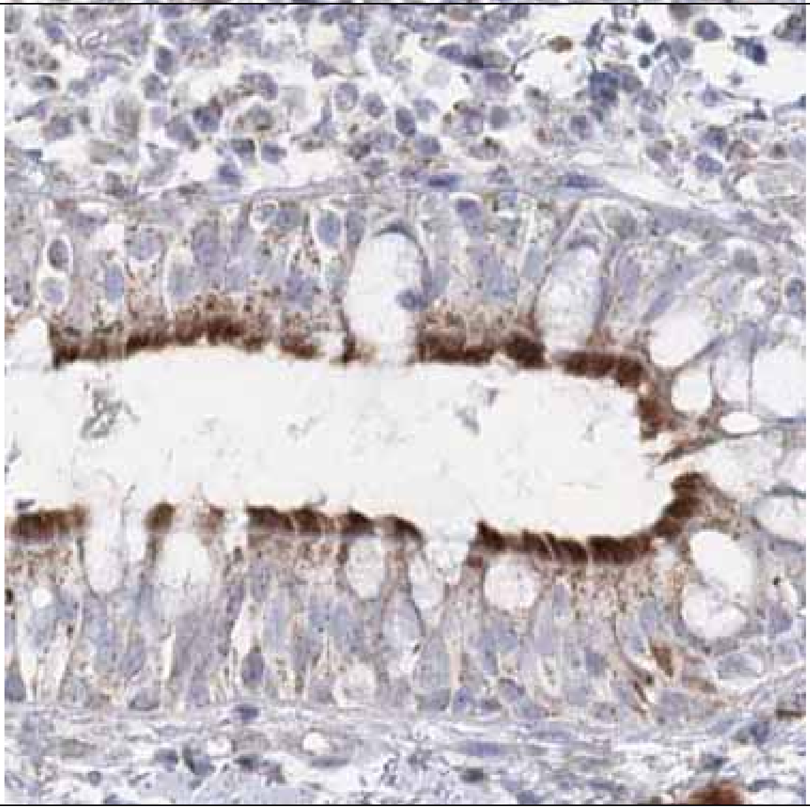	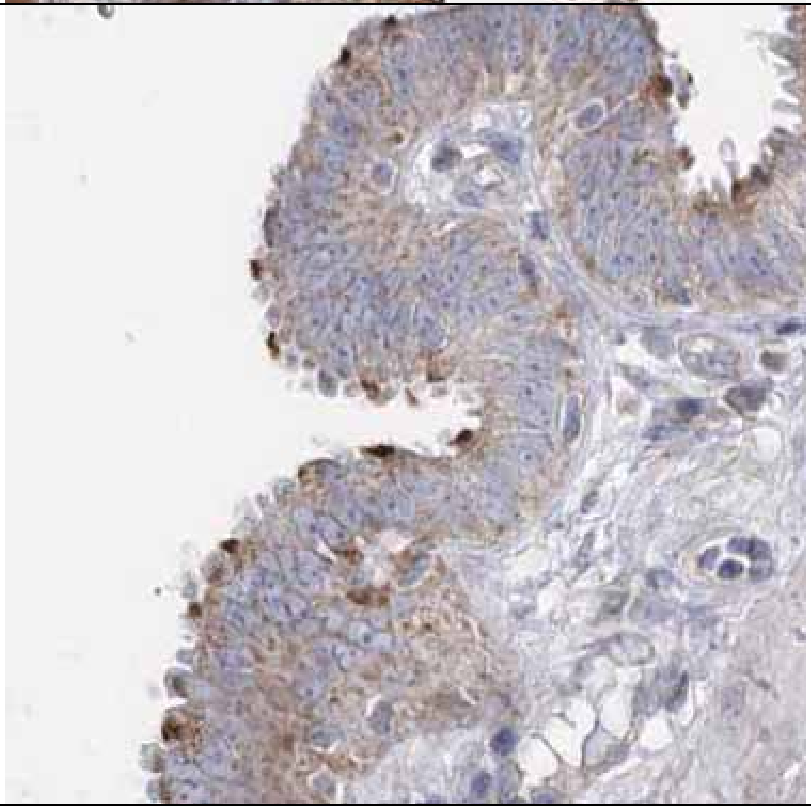	Staining is specific to *cilia*

ARMC4<br>(antibody<br>HPA037829)<br><br>Category 1	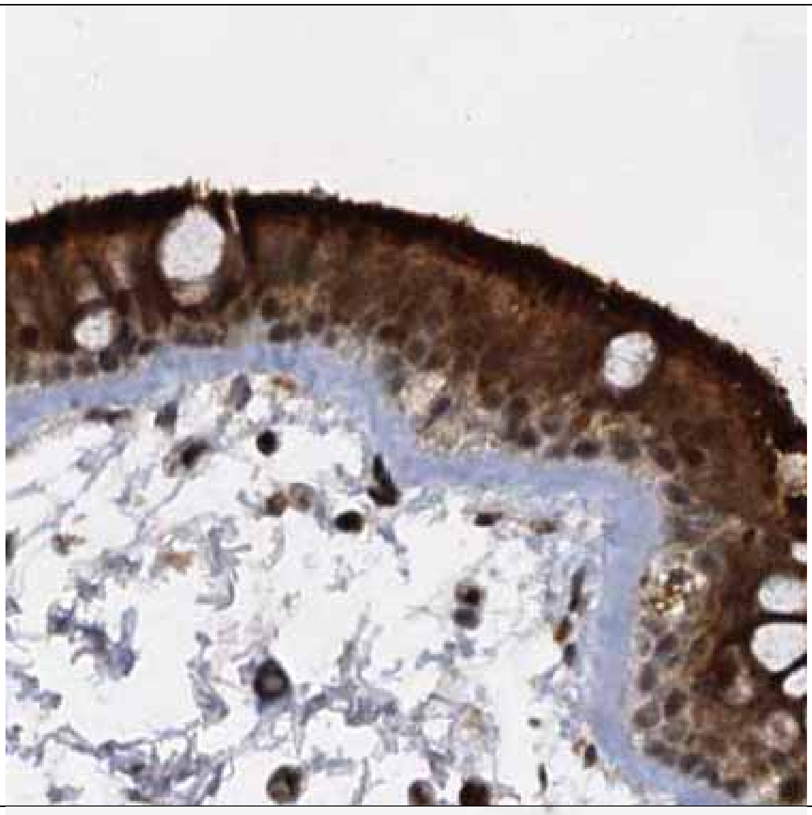	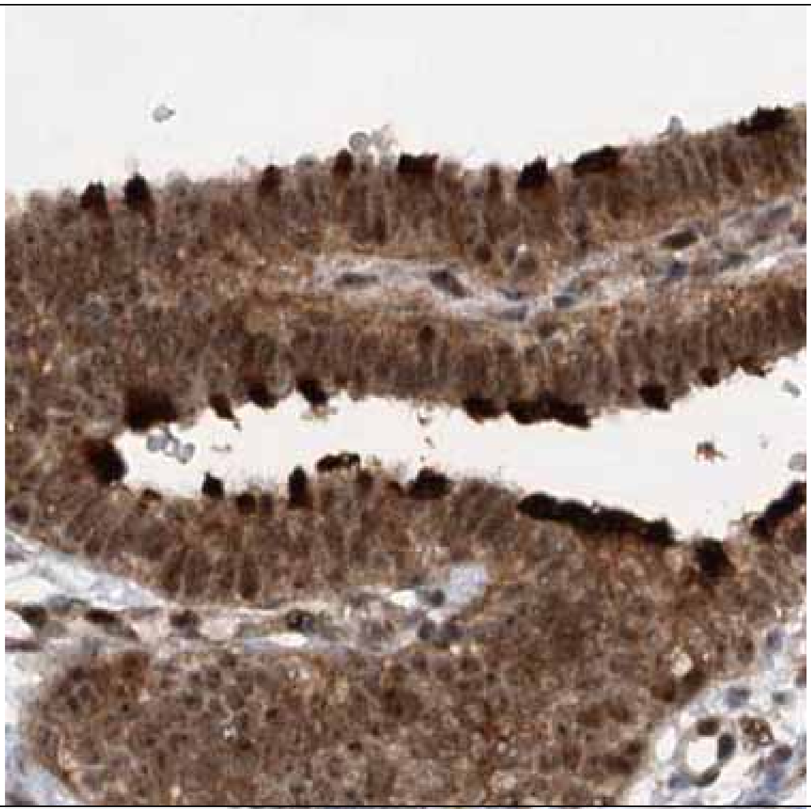	Staining is specific to *cilia*
ARL6<br>(antibody<br>HPA019361)<br><br>Category 1	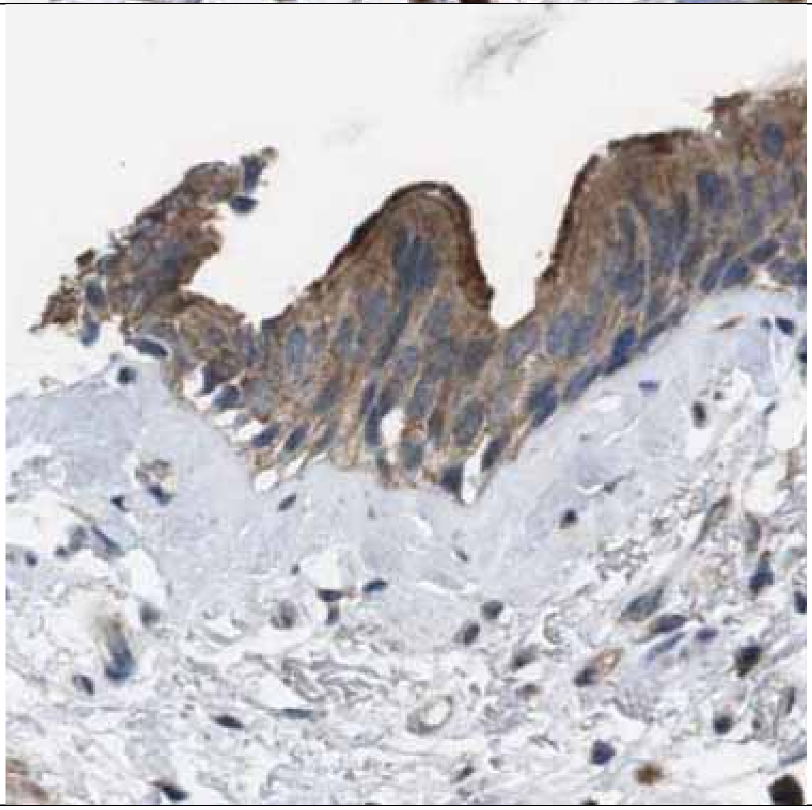	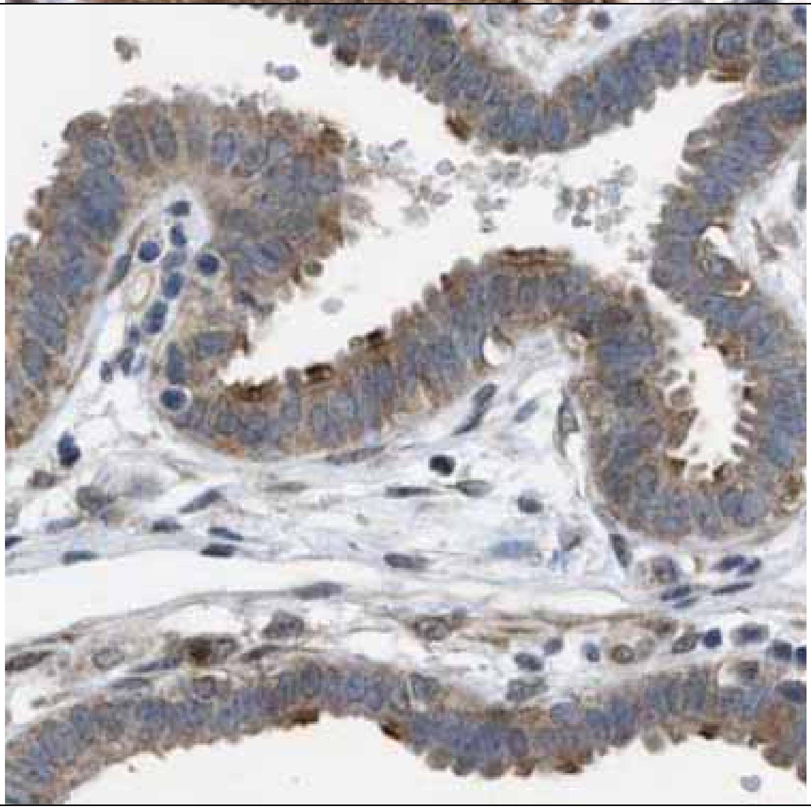	Staining is specific to *cilia*

C1orf158 (antibody HPA028396)<br><br>Category 1	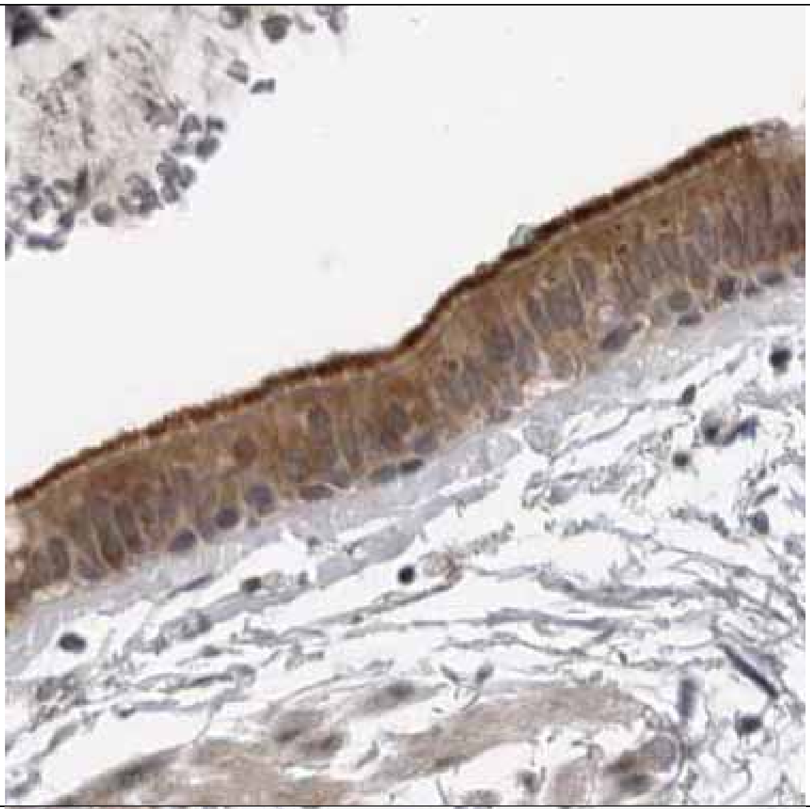	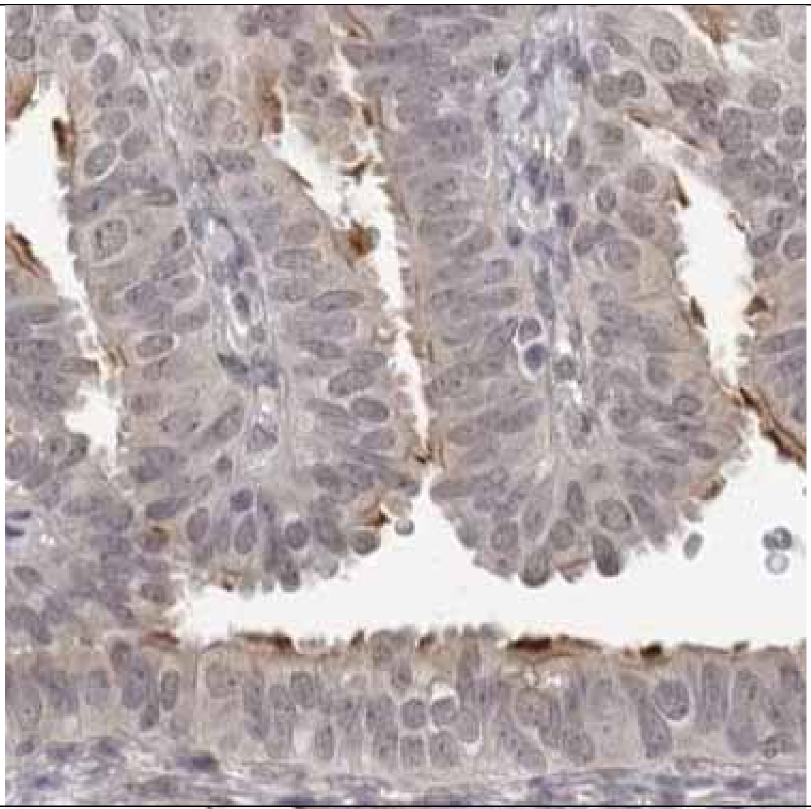	Staining is specific to *cilia*
C10orf63 (antibody HPA037593)<br><br>Category 1	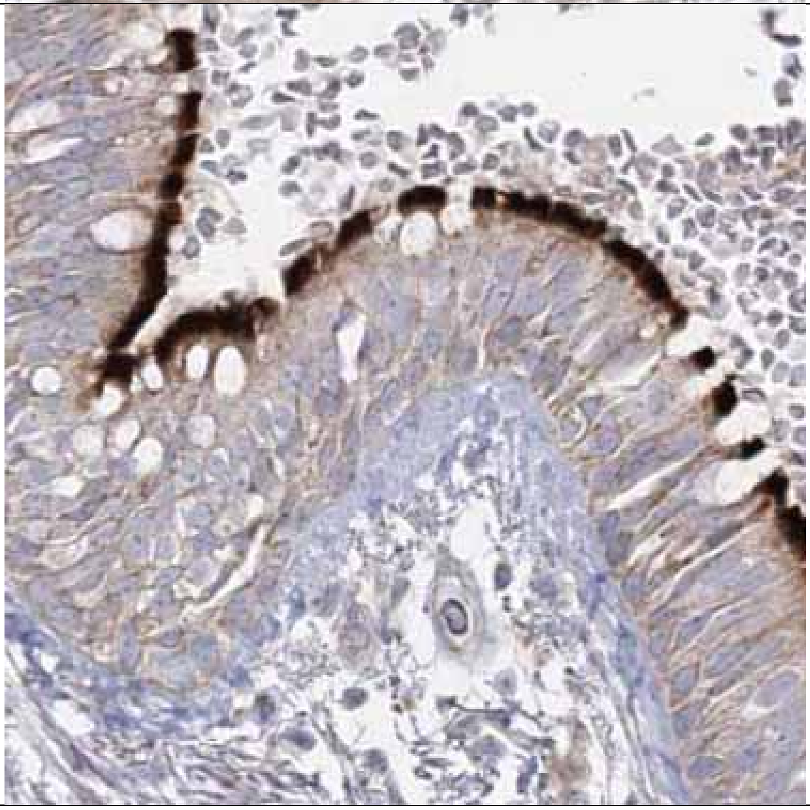	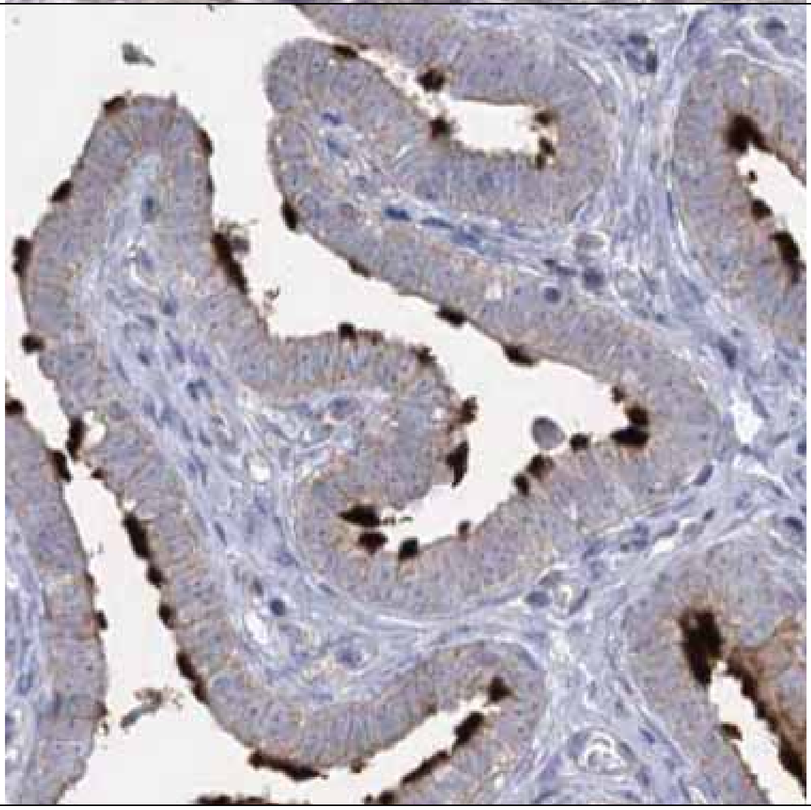	Staining is specific to *cilia*

C20orf26<br>(antibody<br>HPA009079)<br><br>Category 1	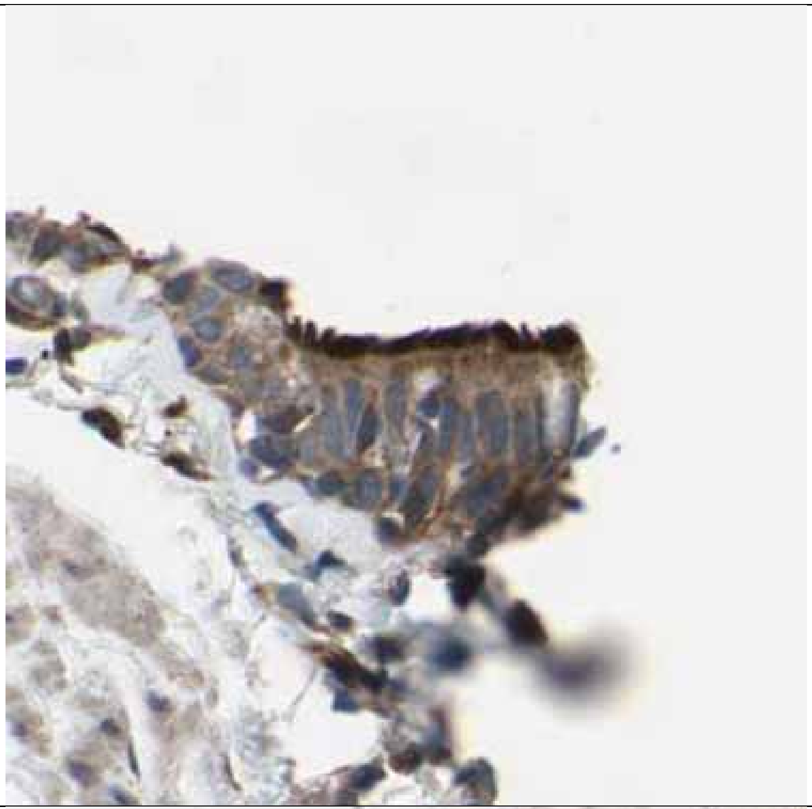	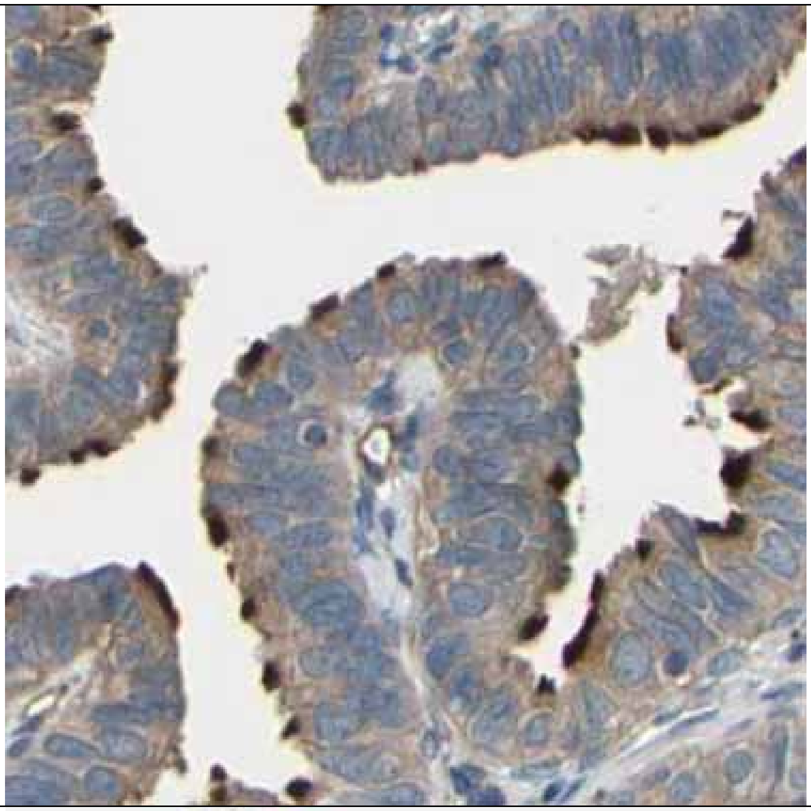	Staining is specific to *cilia*
C21orf59<br>(antibody<br>HPA028849)<br><br>Category 1	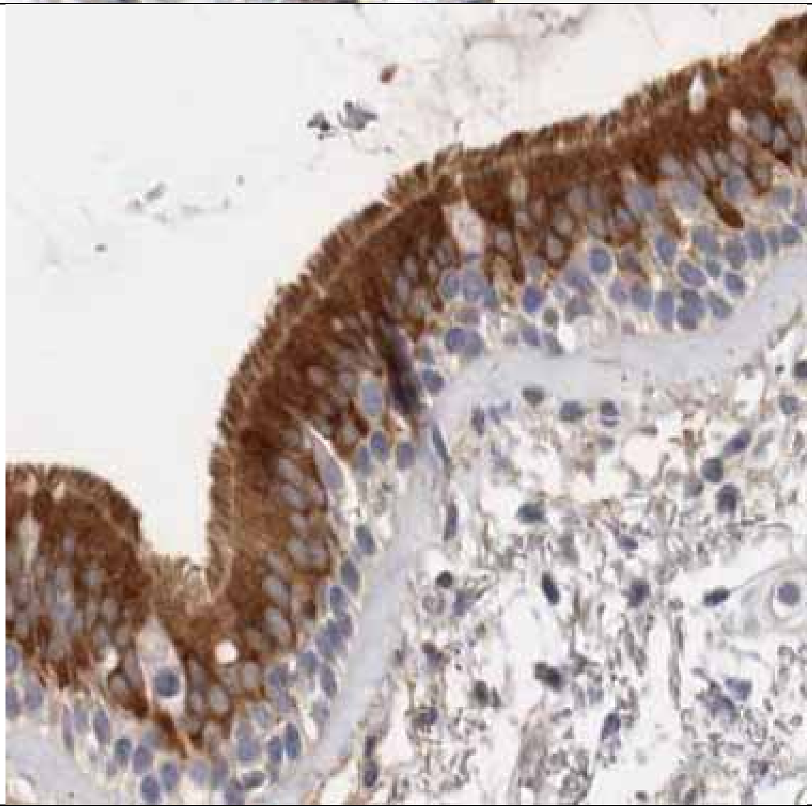	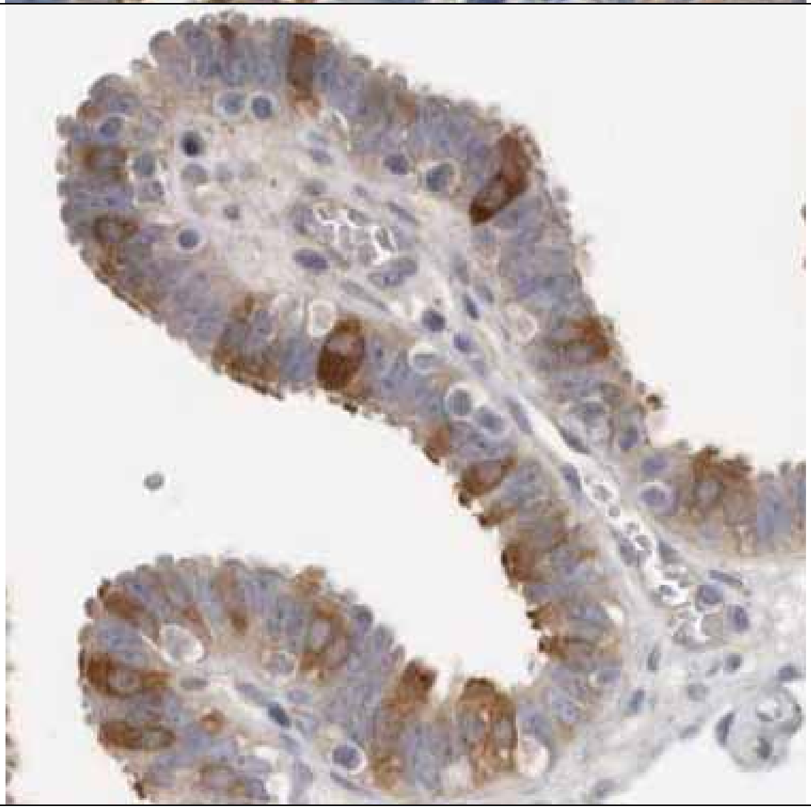	Staining is specific to *cilia*

C3orf15 (antibody HPA035610)<br><br>Category 1	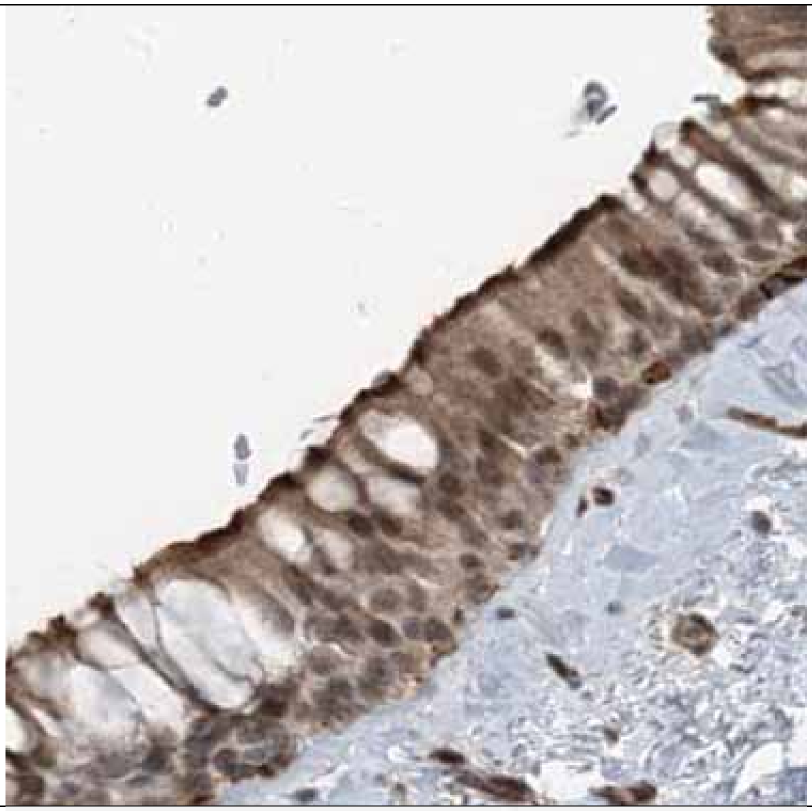	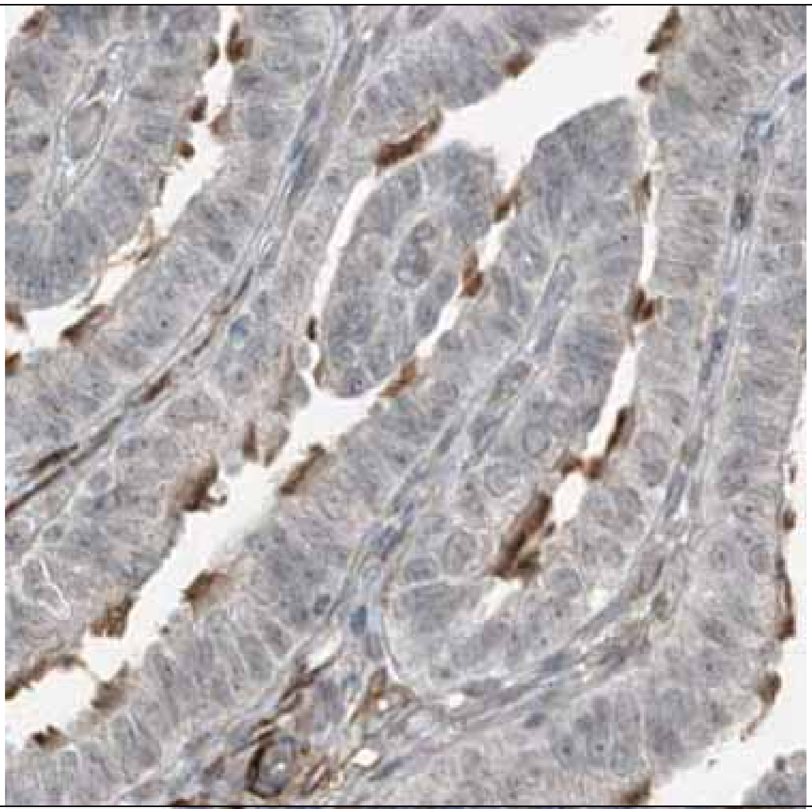	Staining is specific to *cilia*
C7orf57 (antibody HPA021286)<br><br>Category 1	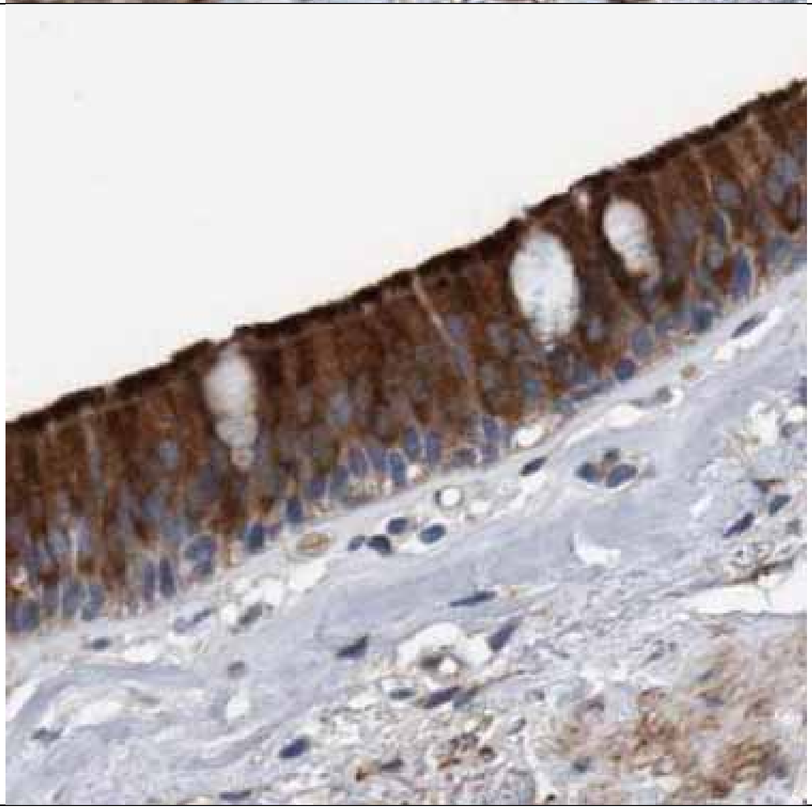	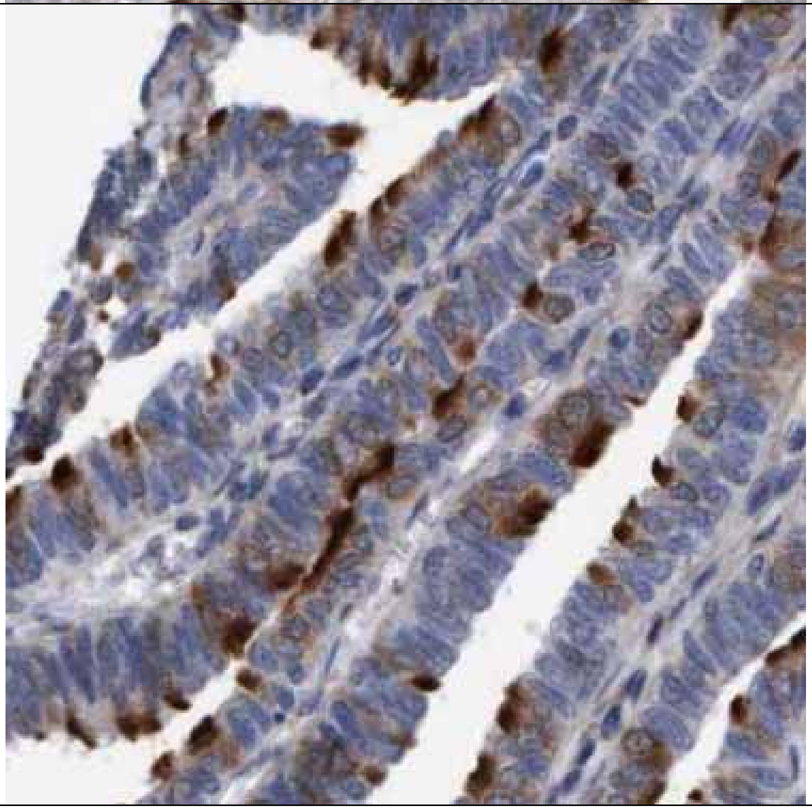	Staining is specific to *cilia*

C7orf67<br>(antibody<br>HPA030130)<br><br>Category 1	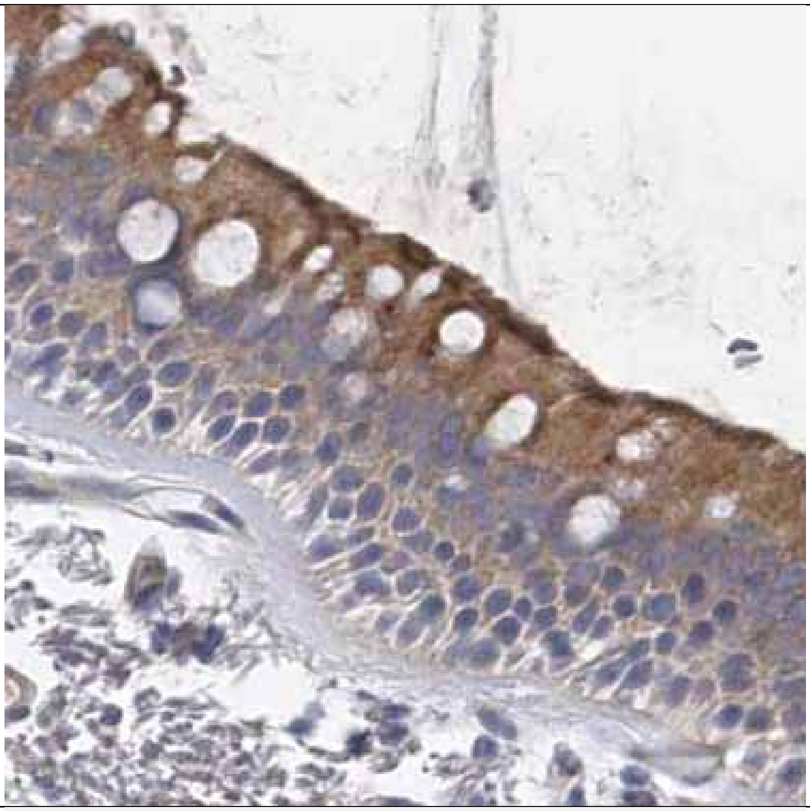	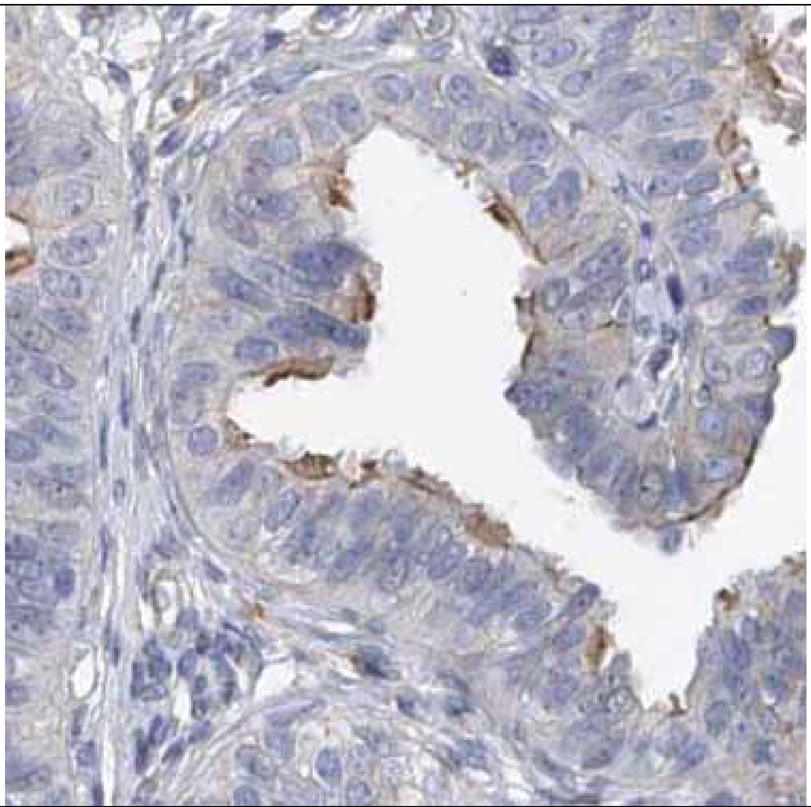	Staining is specific to *cilia*
C8orf47<br>(antibody<br>HPA025070)<br><br>Category 1	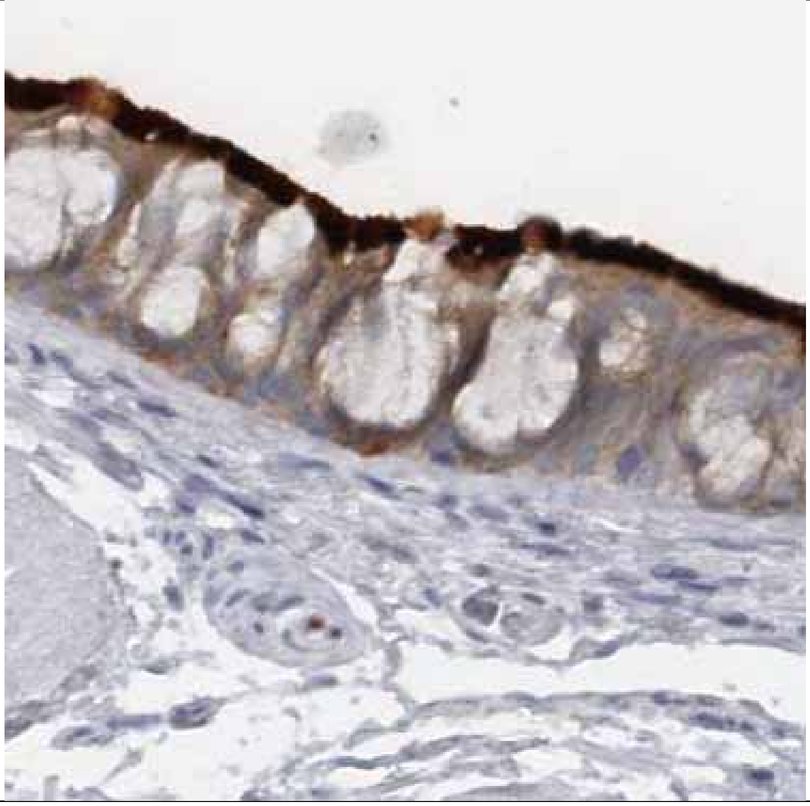	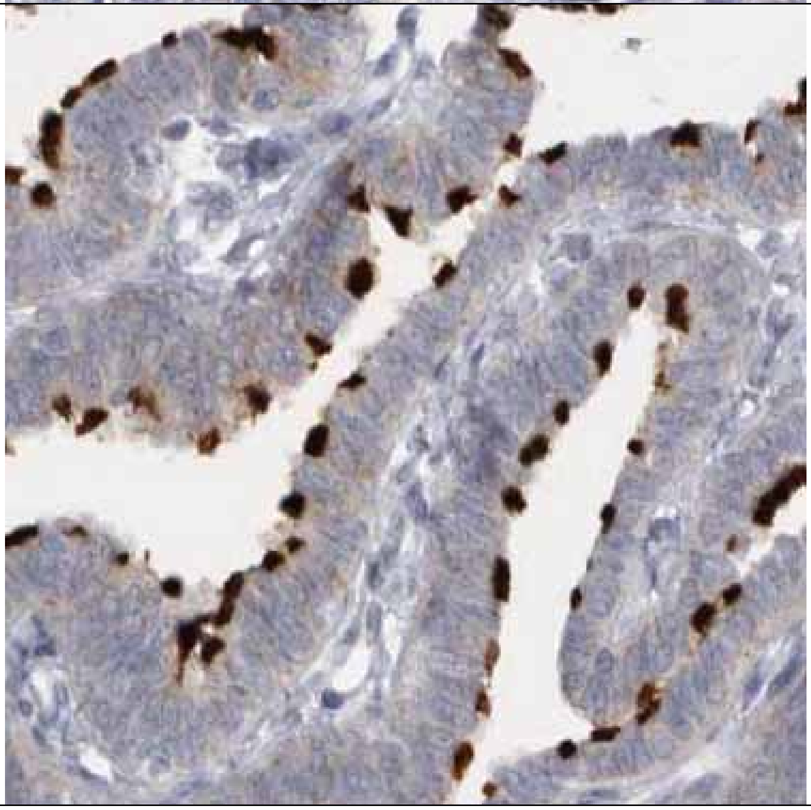	Staining is specific to *cilia*

C9orf116 (antibody HPA021439)<br><br>Category 1	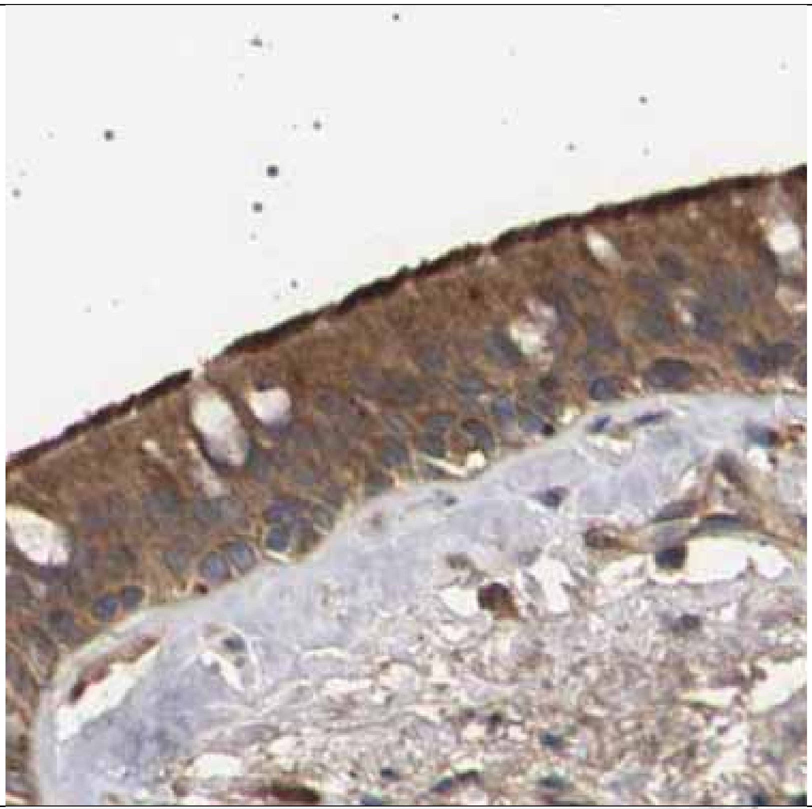	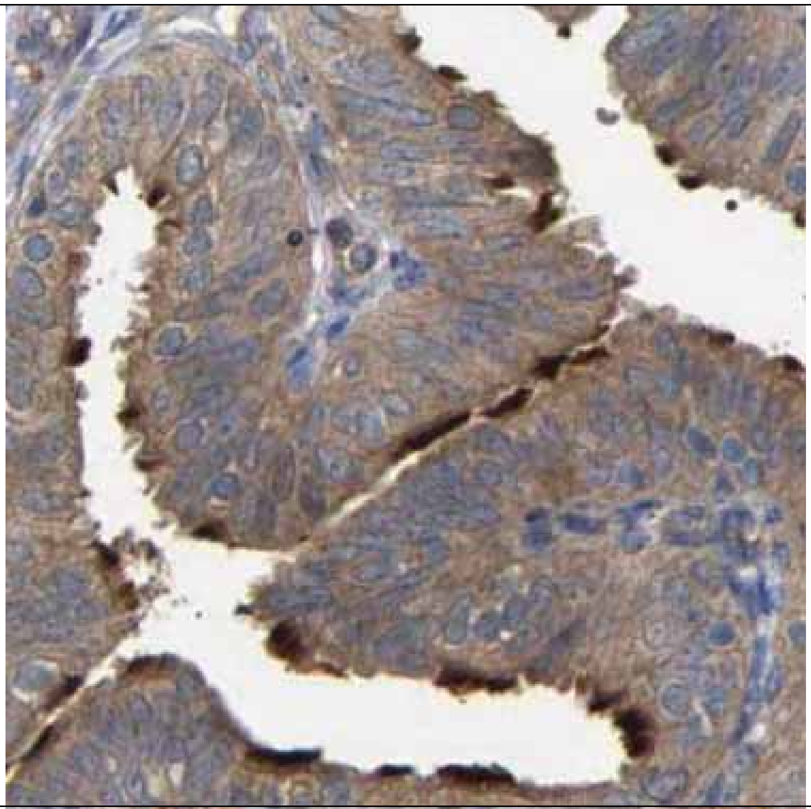	Staining is specific to *cilia*
C9orf117 (antibody HPA021786)<br><br>Category 1	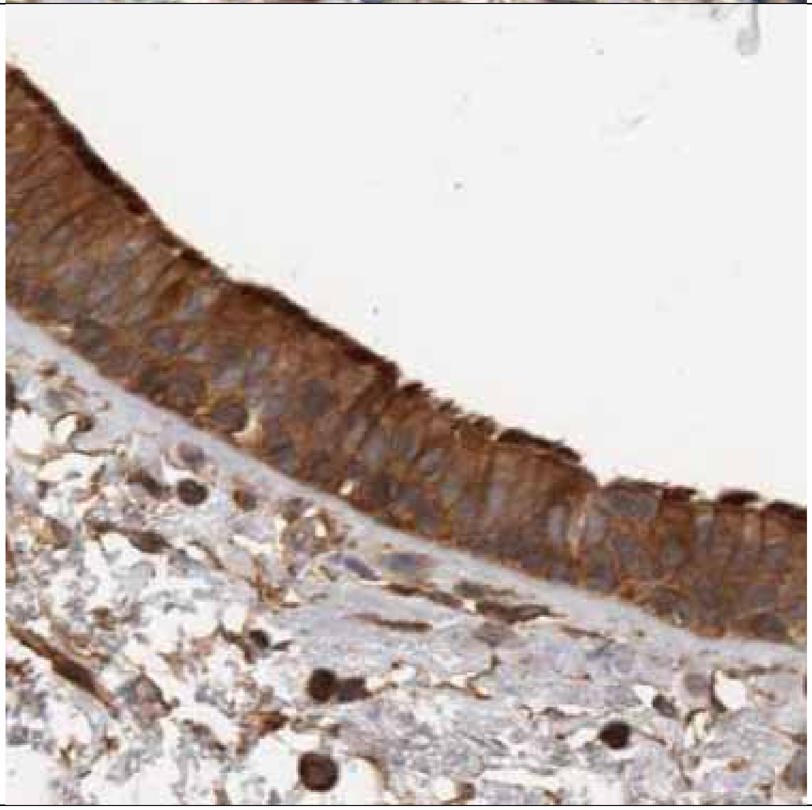	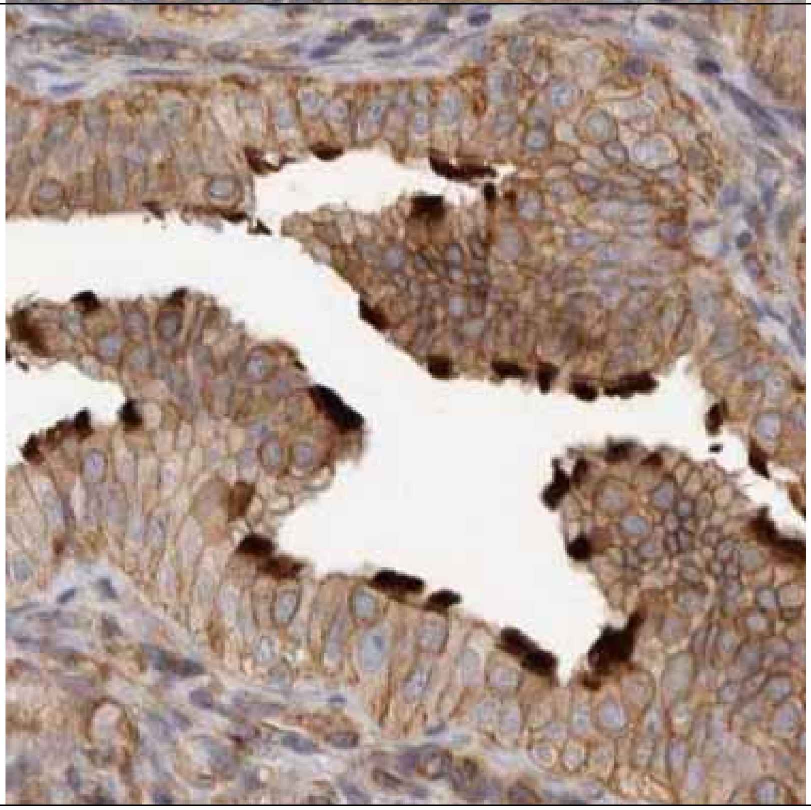	Staining is specific to *cilia*

C9orf98 (antibody HPA023894)<br><br>Category 1	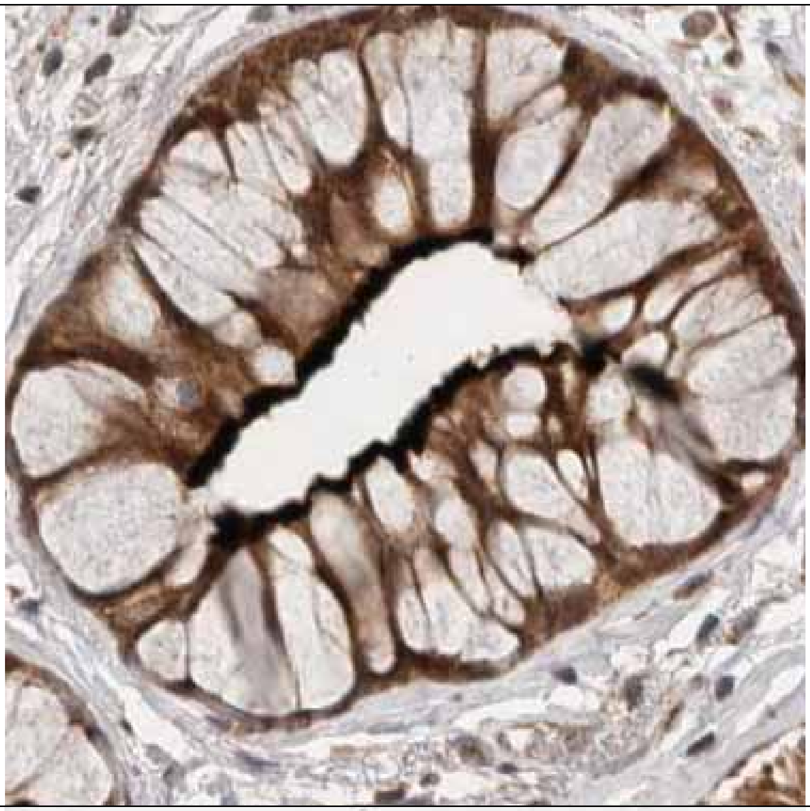	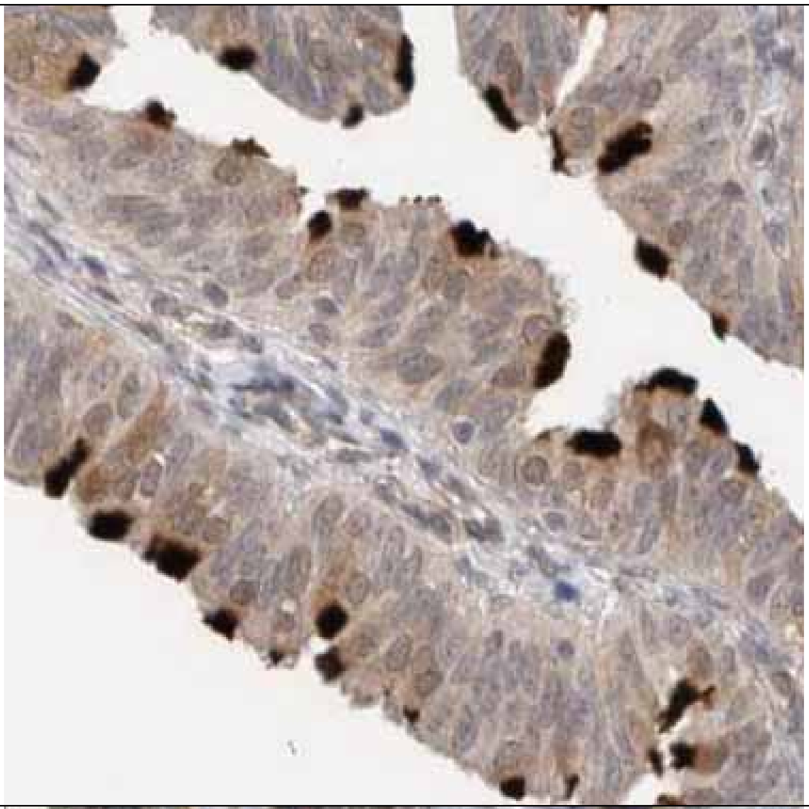	Staining is specific to *cilia*
CCDC104 (antibody HPA017061)<br><br>Category 1	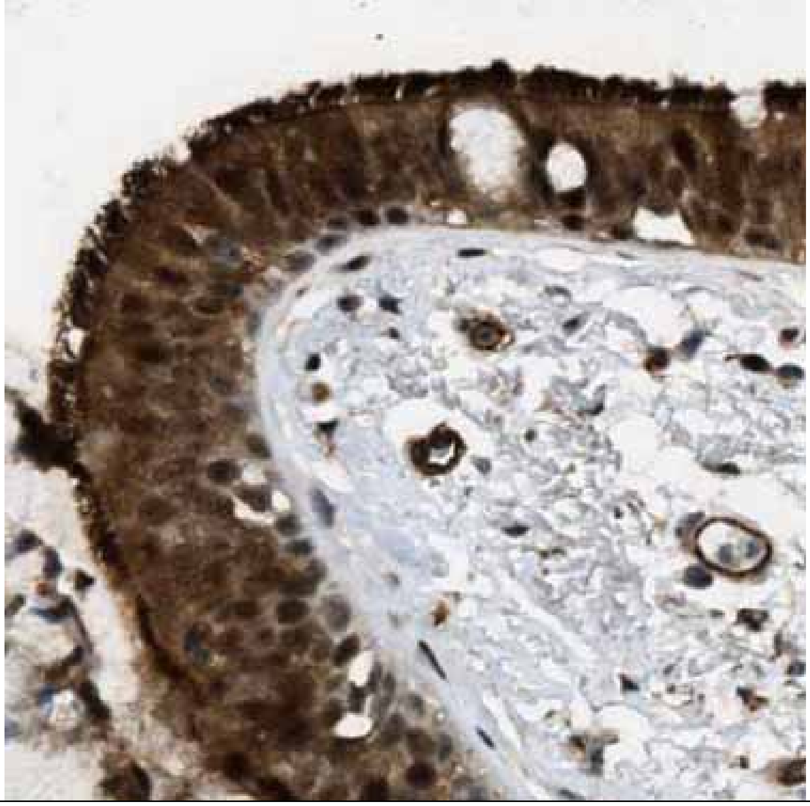	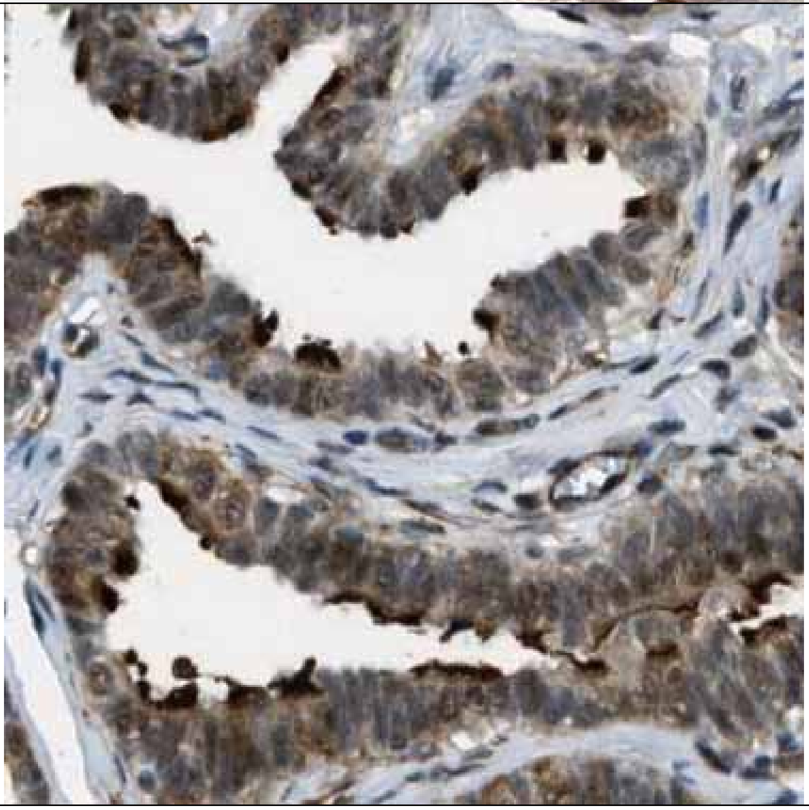	Staining is specific to *cilia*

CCDC146 (antibody HPA020082)<br><br>Category 1	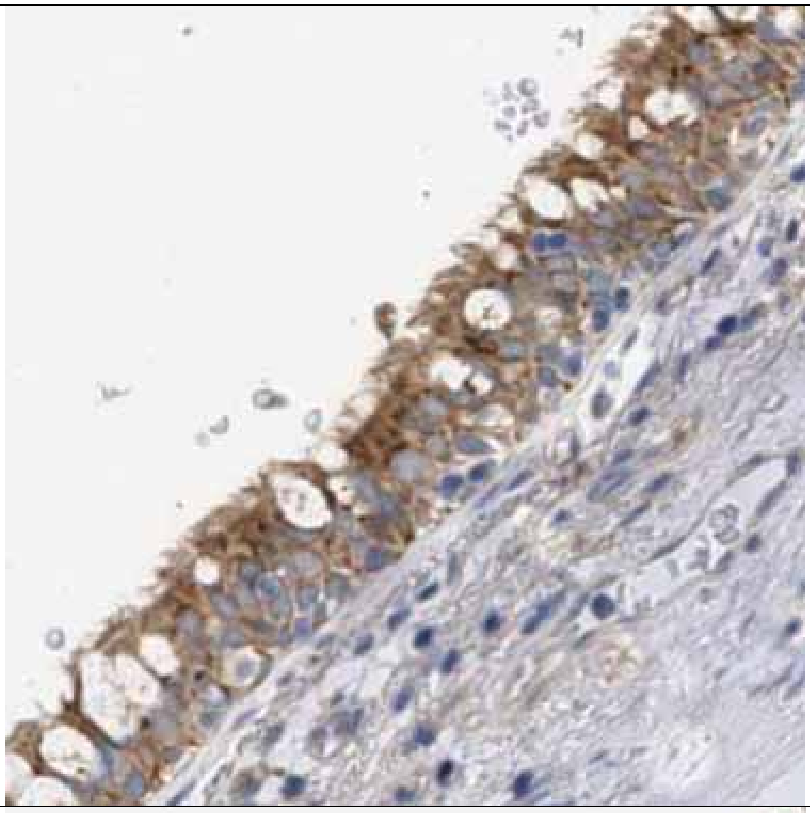	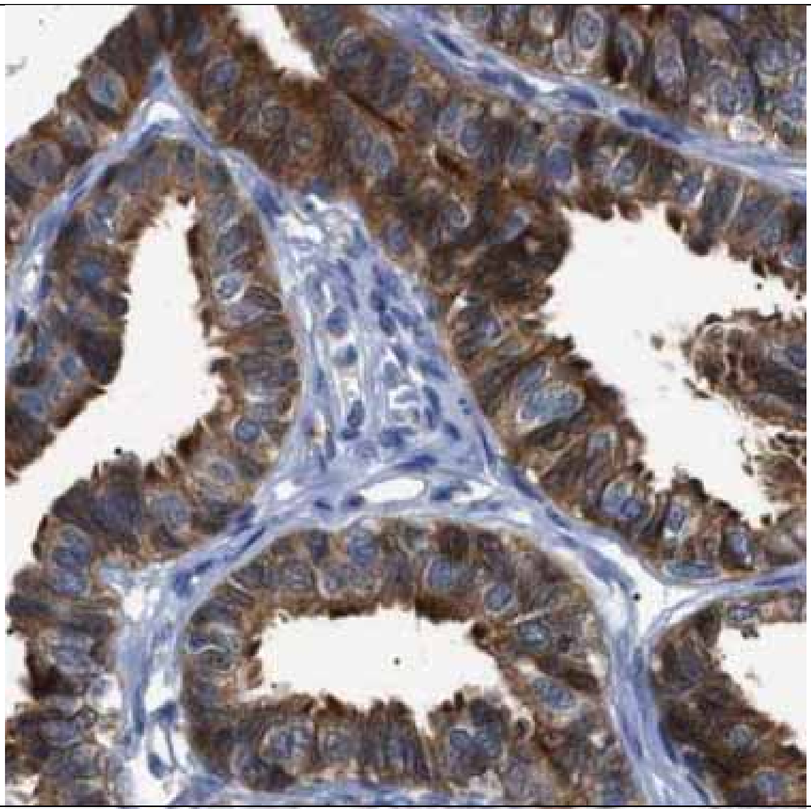	Staining is specific to *cilia*
CCDC147 (antibody HPA036555)<br><br>Category 1	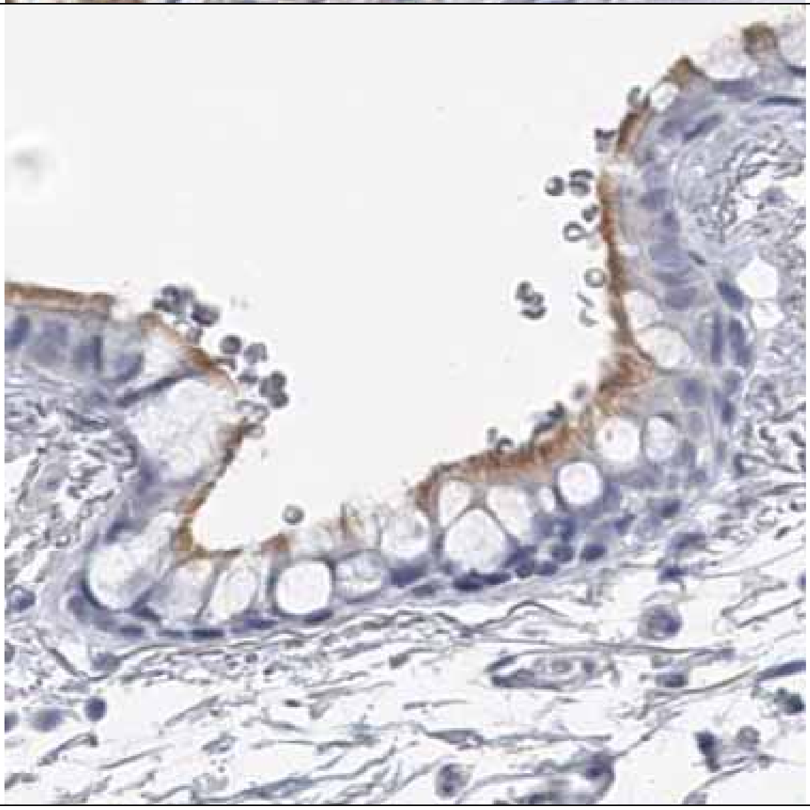	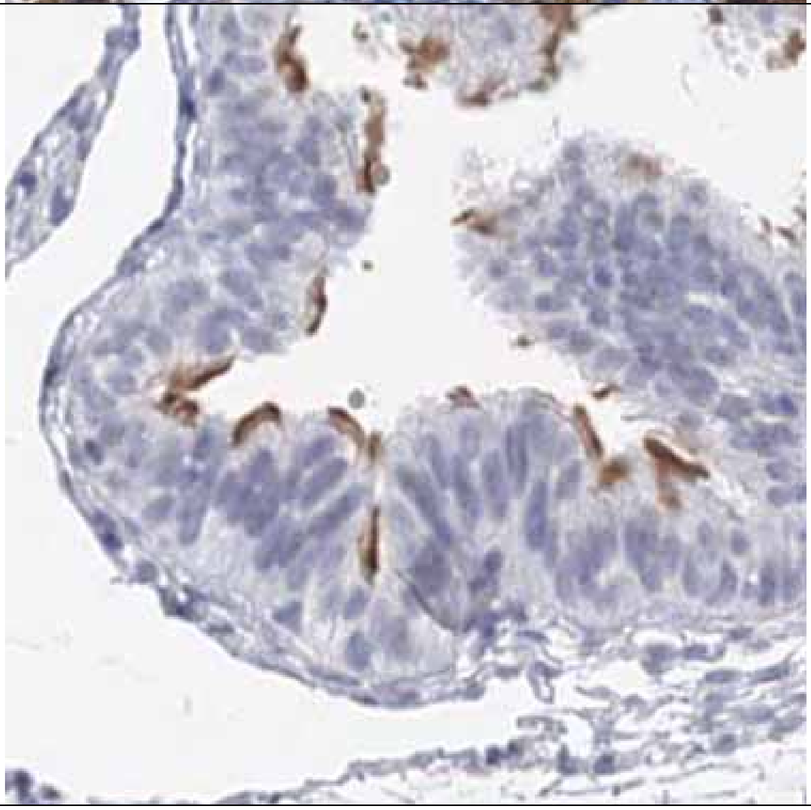	Staining is specific to *cilia*

CCDC39 (antibody HPA035364)<br><br>Category 1	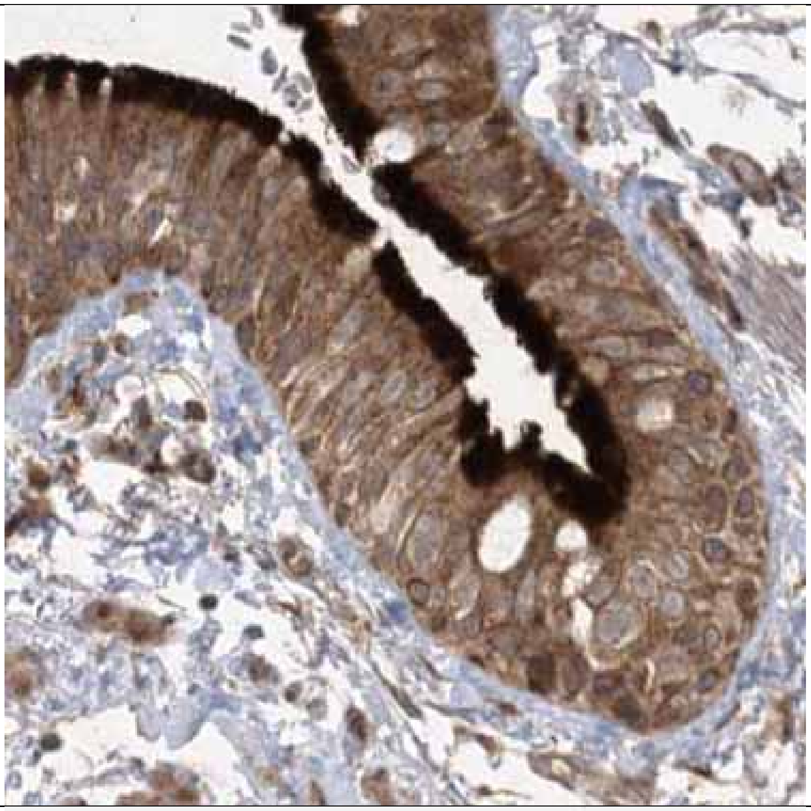	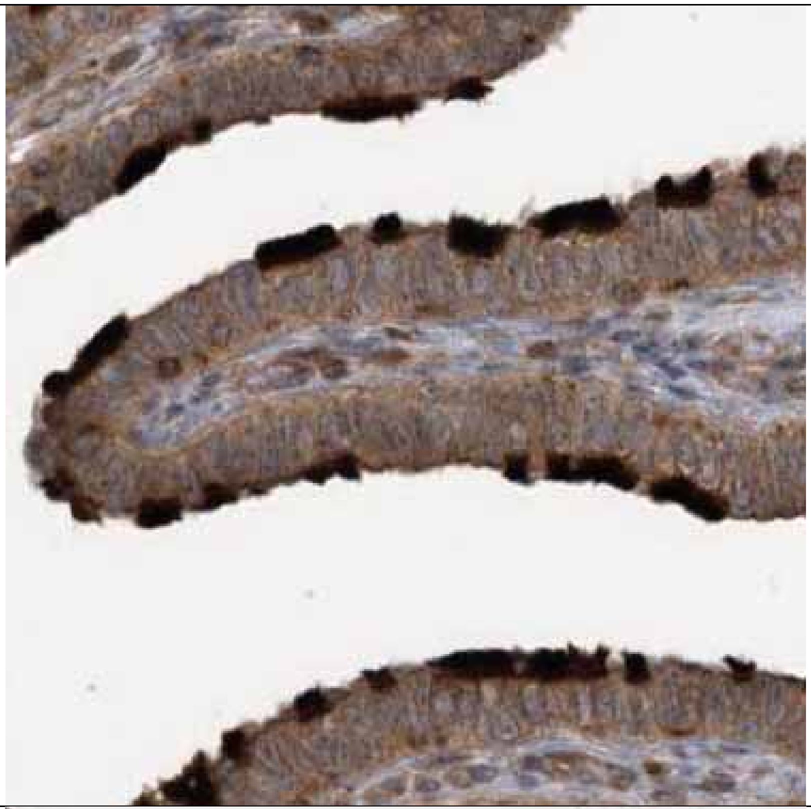	Staining is specific to *cilia*
CCDC40 (antibody HPA022974)<br><br>Category 1	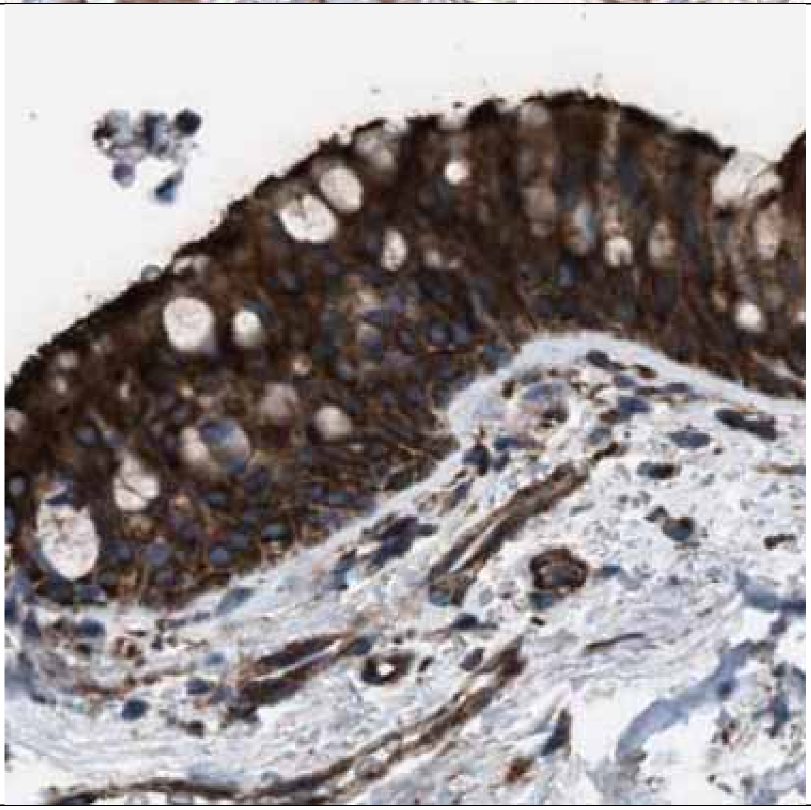	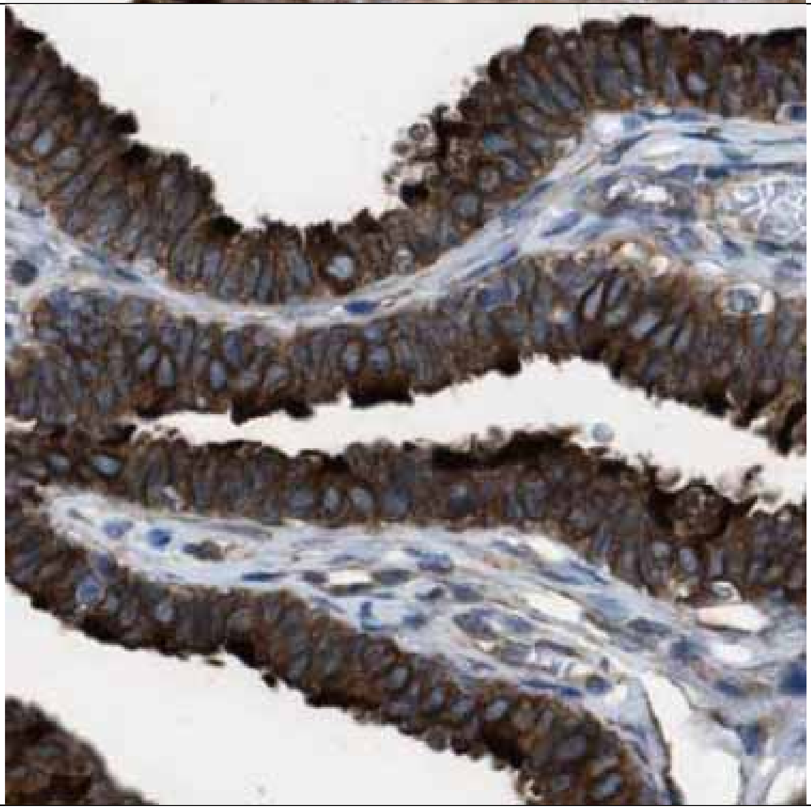	Staining is specific to *cilia*

CETN2<br>(antibody<br>HPA028956)<br><br>Category 1	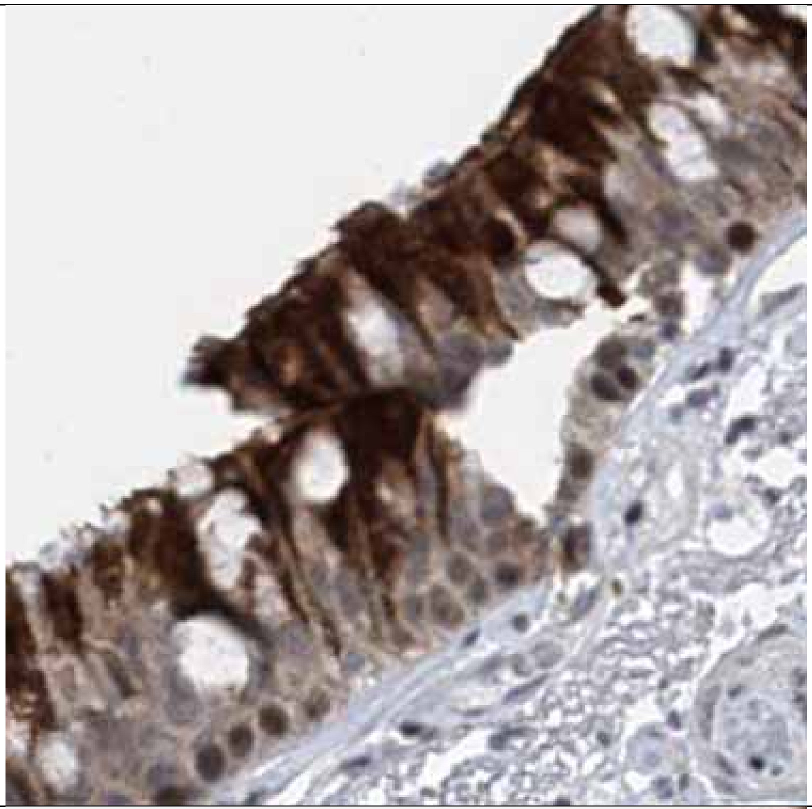	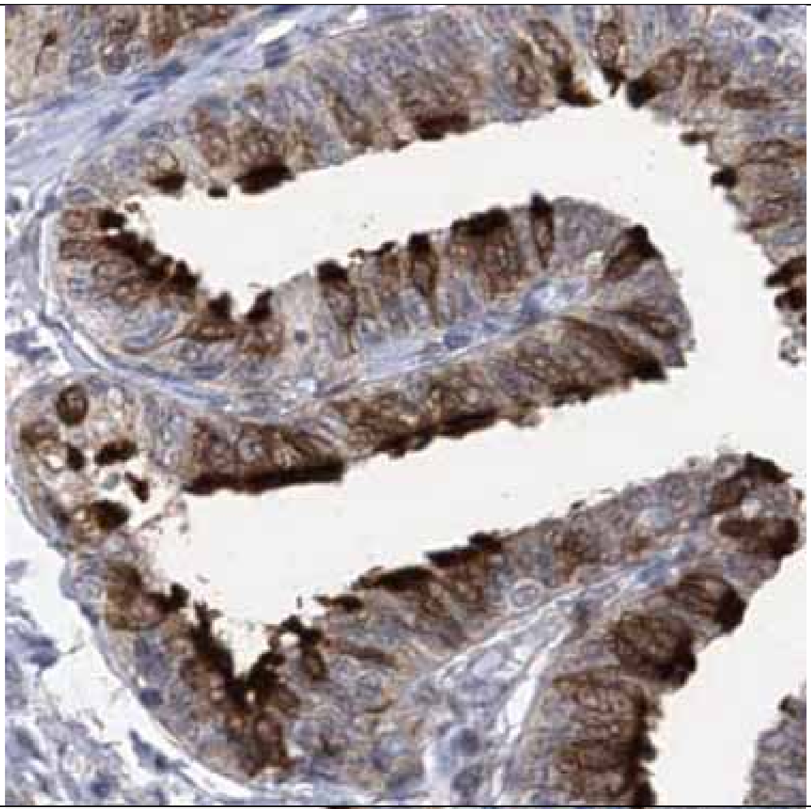	Staining is specific to *cilia*
CYB5D1<br>(antibody<br>HPA021632)<br><br>Category 1	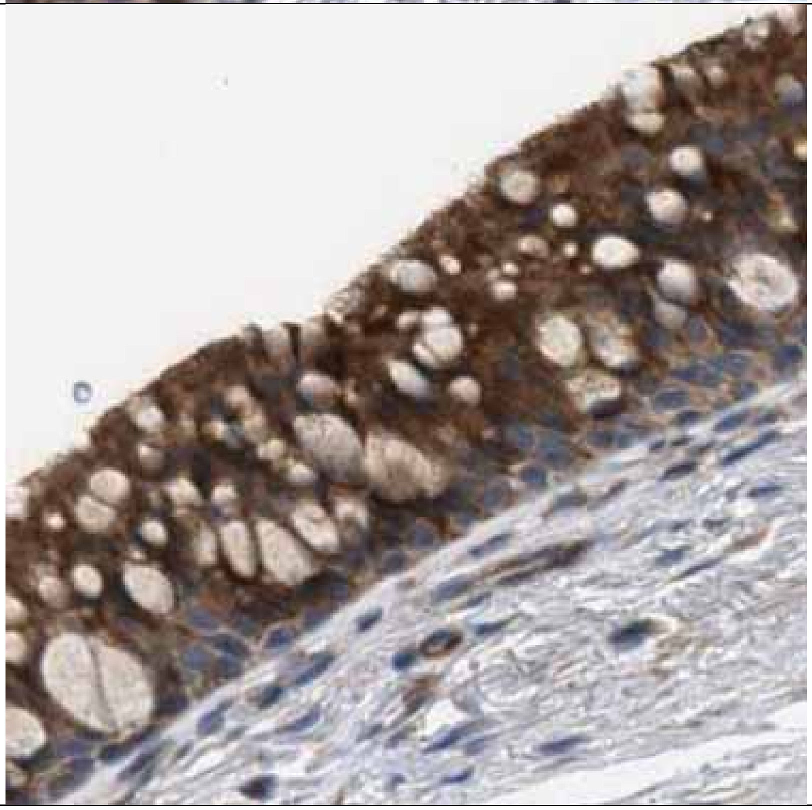	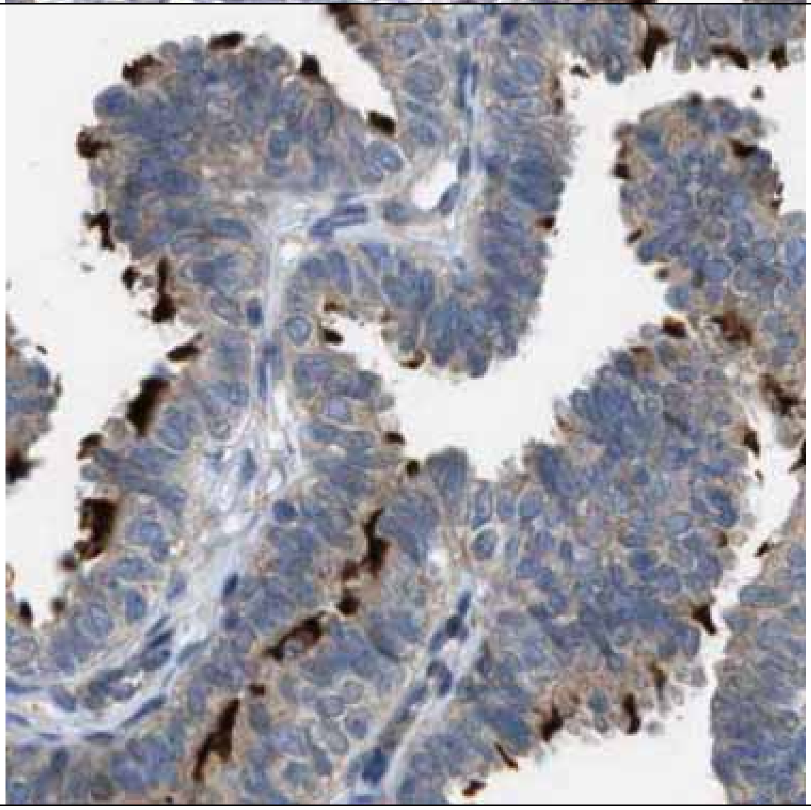	Staining is specific to *cilia*

DCDC2 (antibody HPA031582)<br><br>Category 1	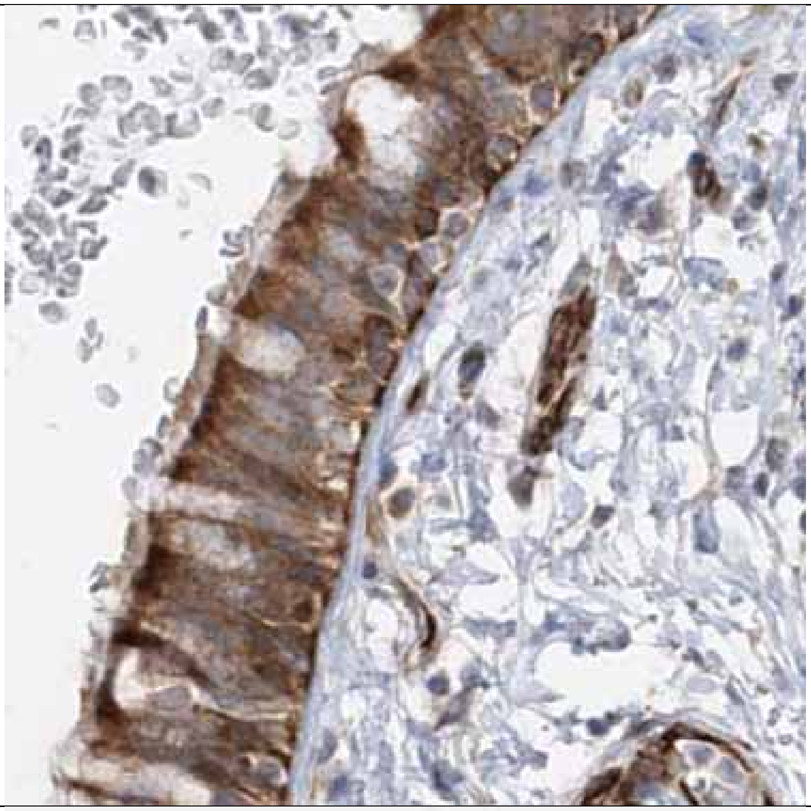	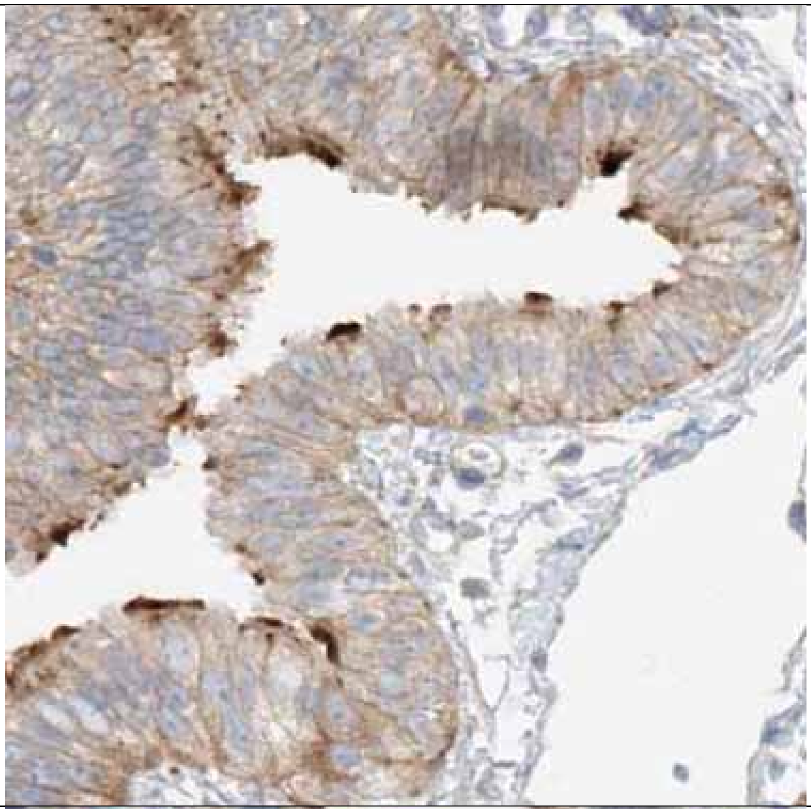	Staining is specific to *cilia*
DLEC1 (antibody HPA019077)<br><br>Category 1	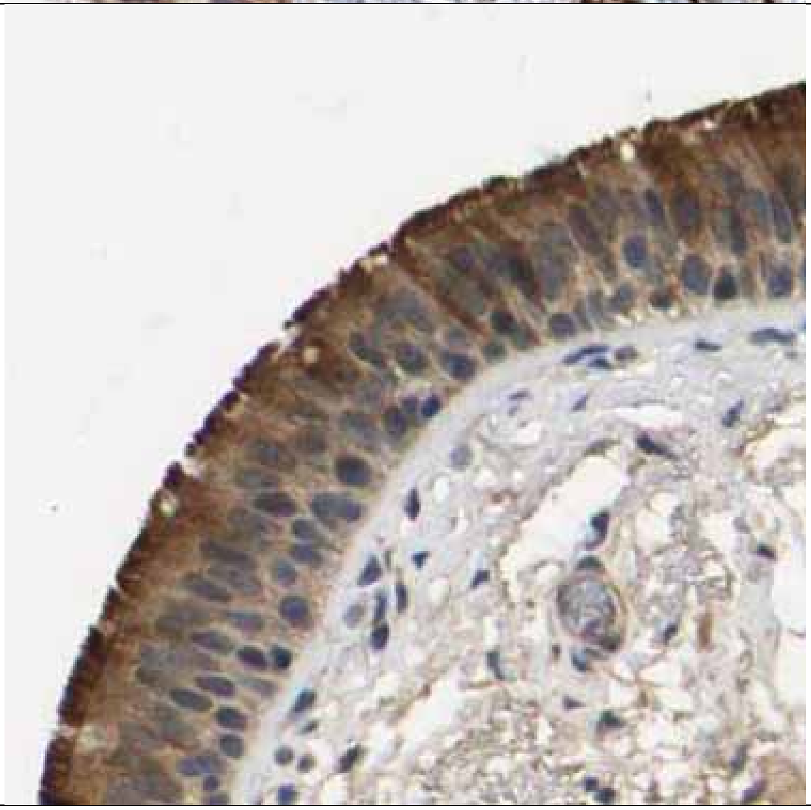	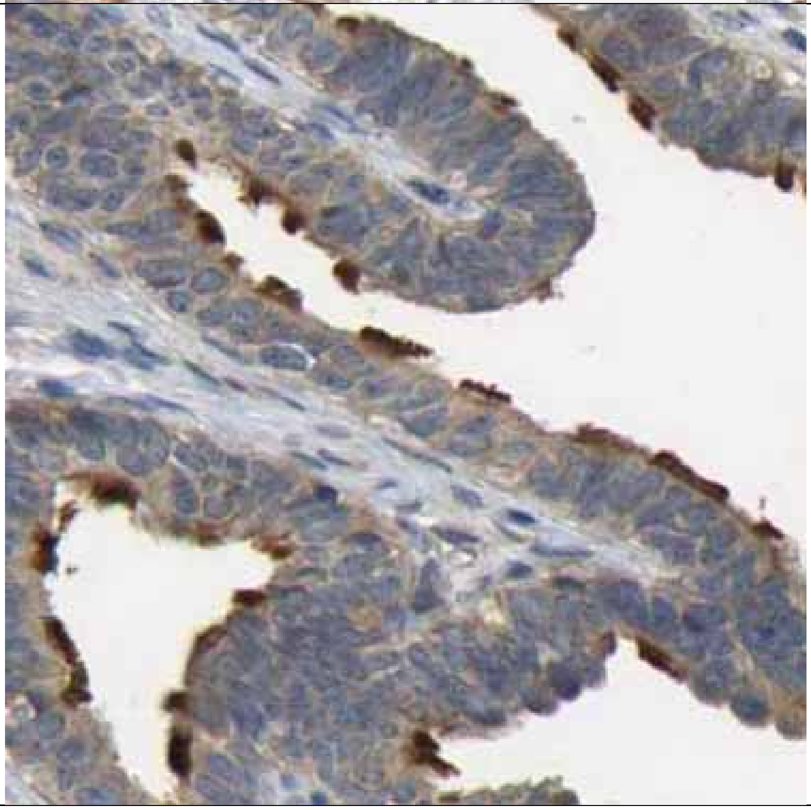	Staining is specific to *cilia*

DNAH5 (antibody HPA037469)<br><br>Category 1	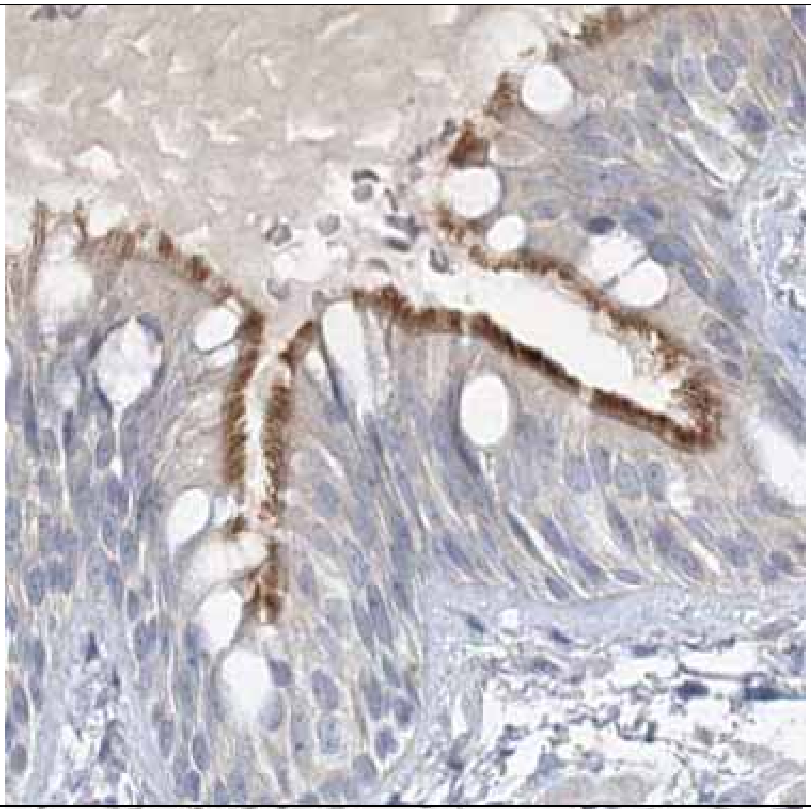	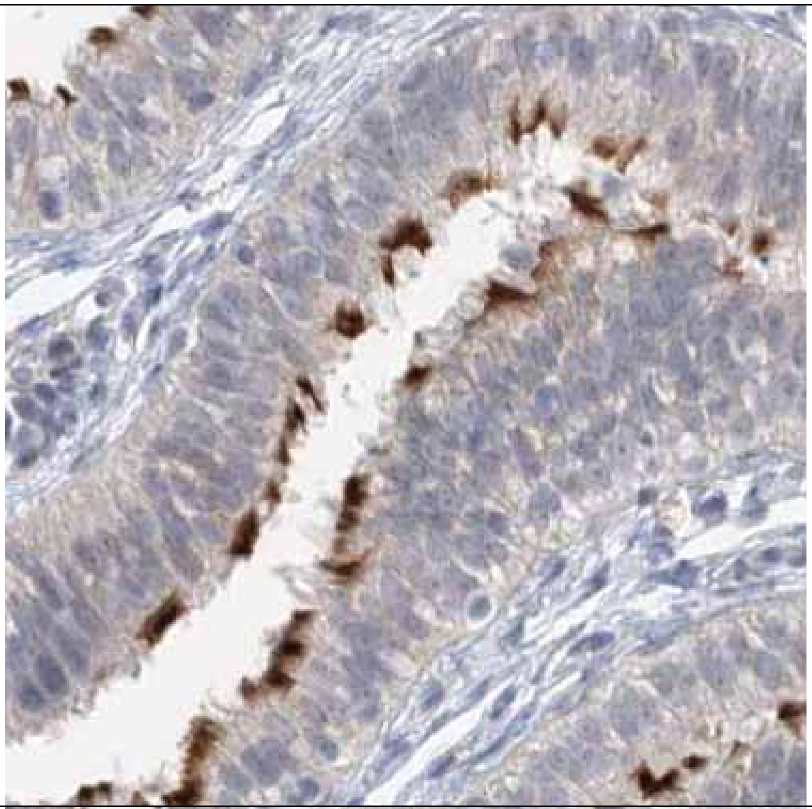	Staining is specific to *cilia*
DNAH7 (antibody HPA034724)<br><br>Category 1	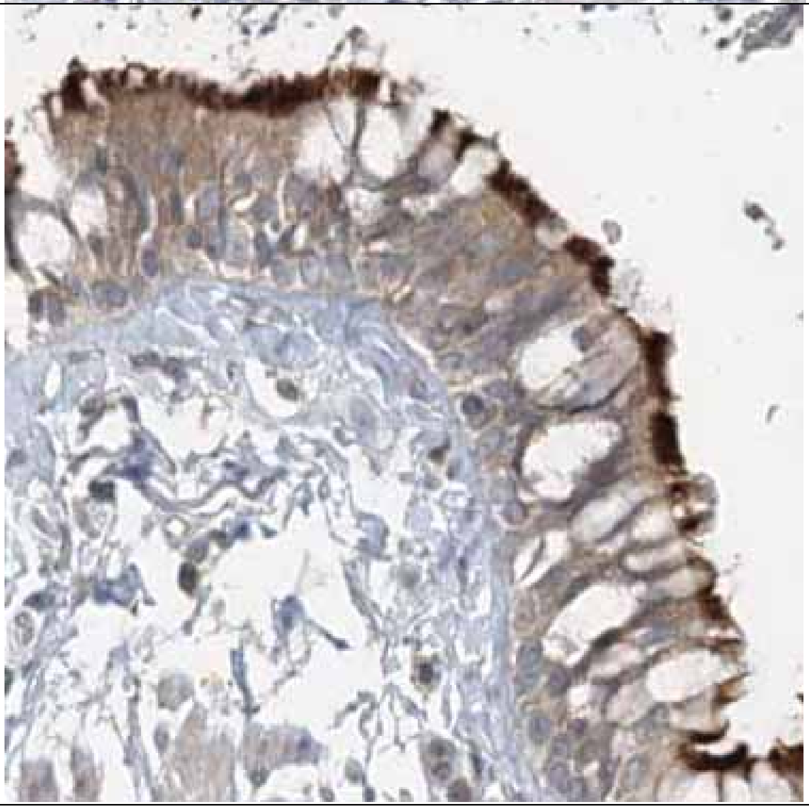	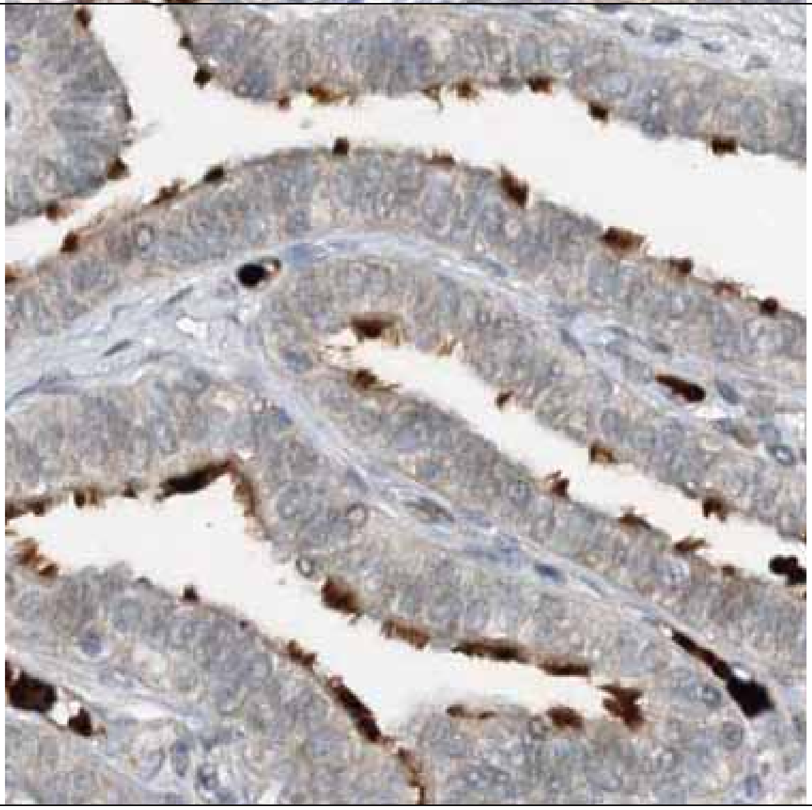	Staining is specific to *cilia*

DNAH9 (antibody HPA020639)<br><br>Category 1	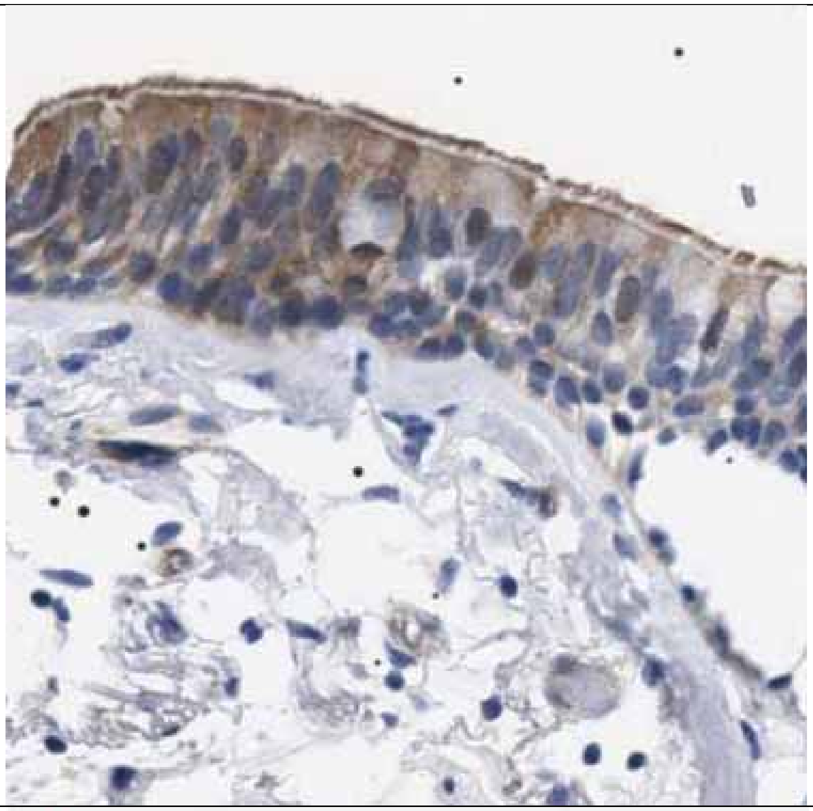	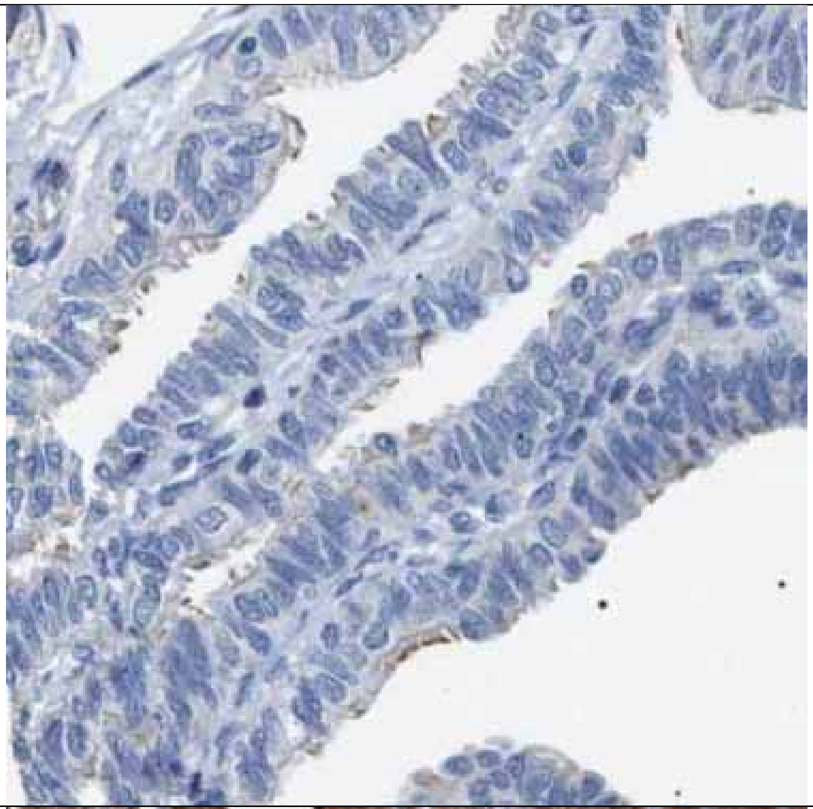	Staining is specific to *cilia*
DNAH12 (antibody HPA037493)<br><br>Category 1	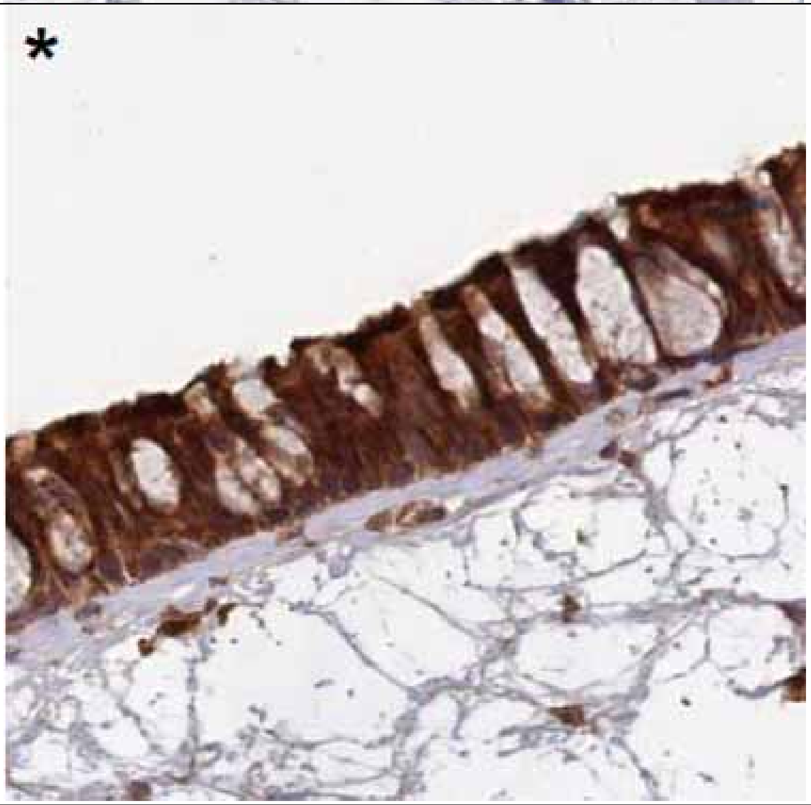	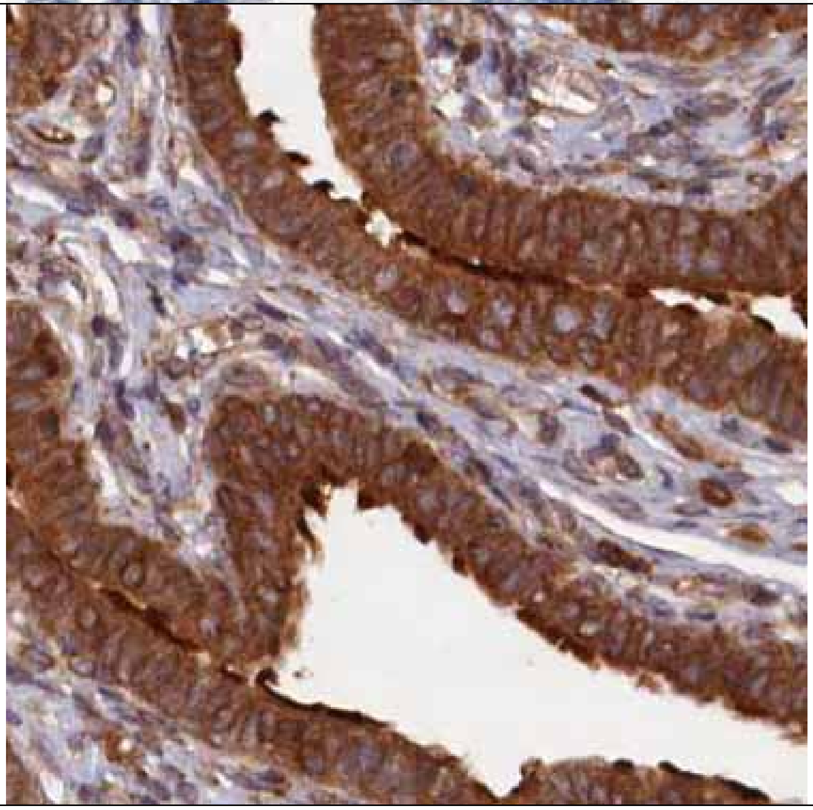	Staining is specific to *cilia*

DNAI1 (antibody HPA021649)<br><br>Category 1	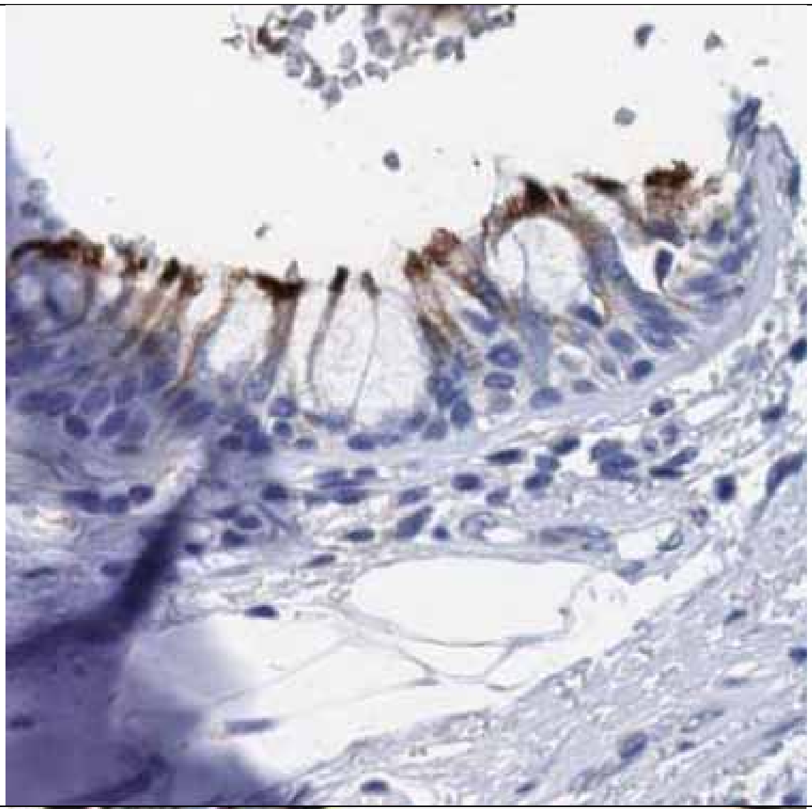	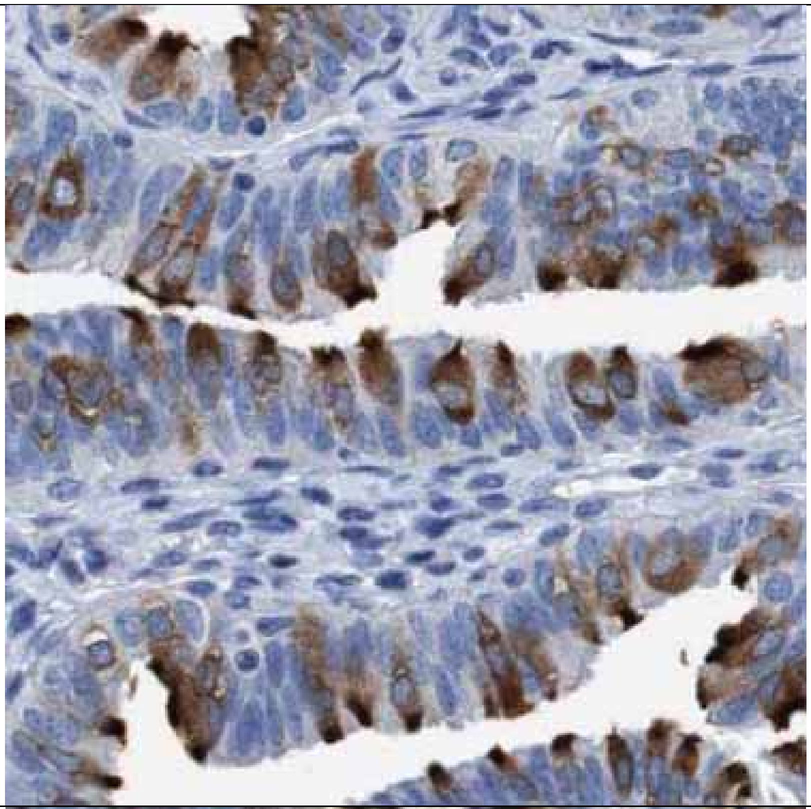	Staining is specific to *cilia*
DNAI2 (antibody CAB006245)<br><br>Category 1	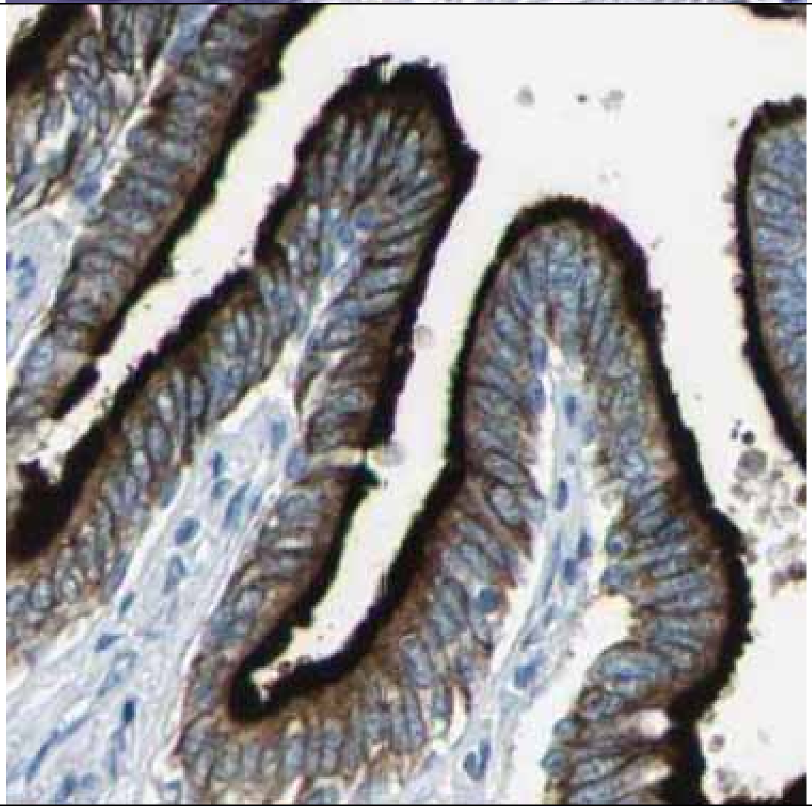	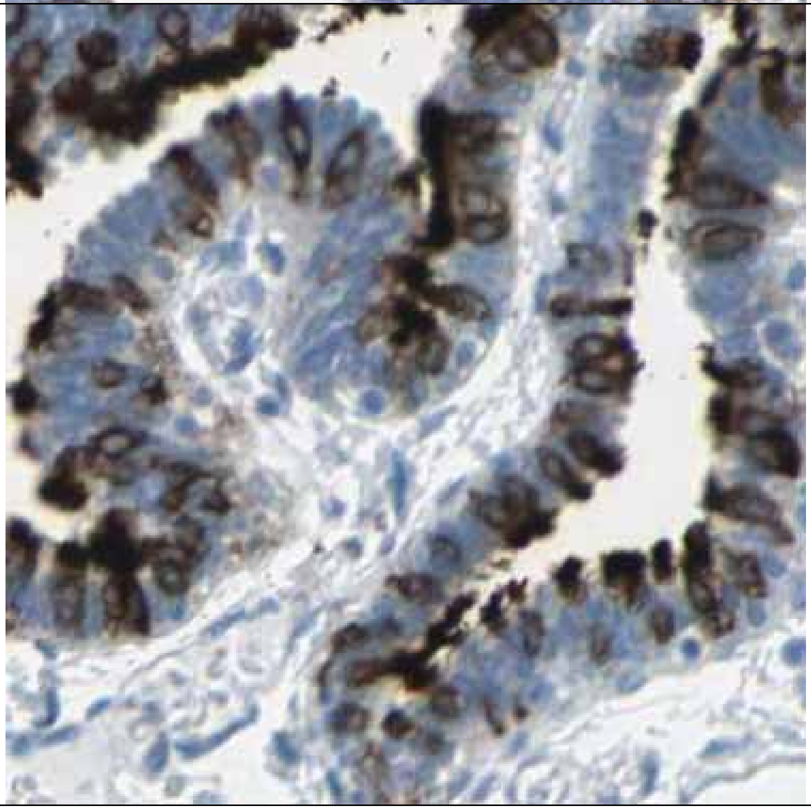	Staining is specific to *cilia*

DNALI1 (antibody HPA028305)<br><br>Category 1	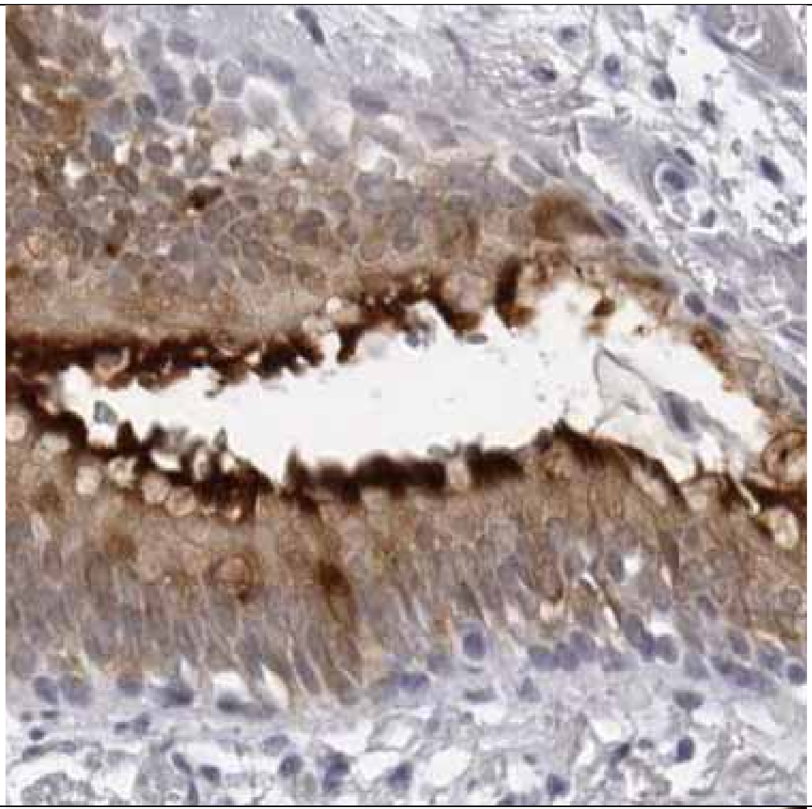	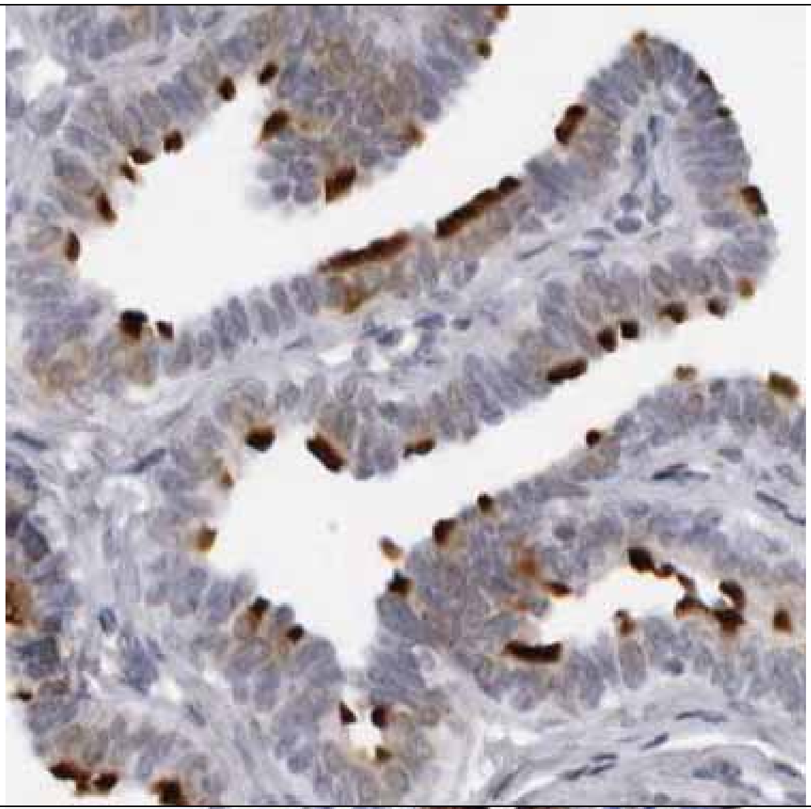	Staining is specific to *cilia*
DYDC2 (antibody HPA038006)<br><br>Category 1	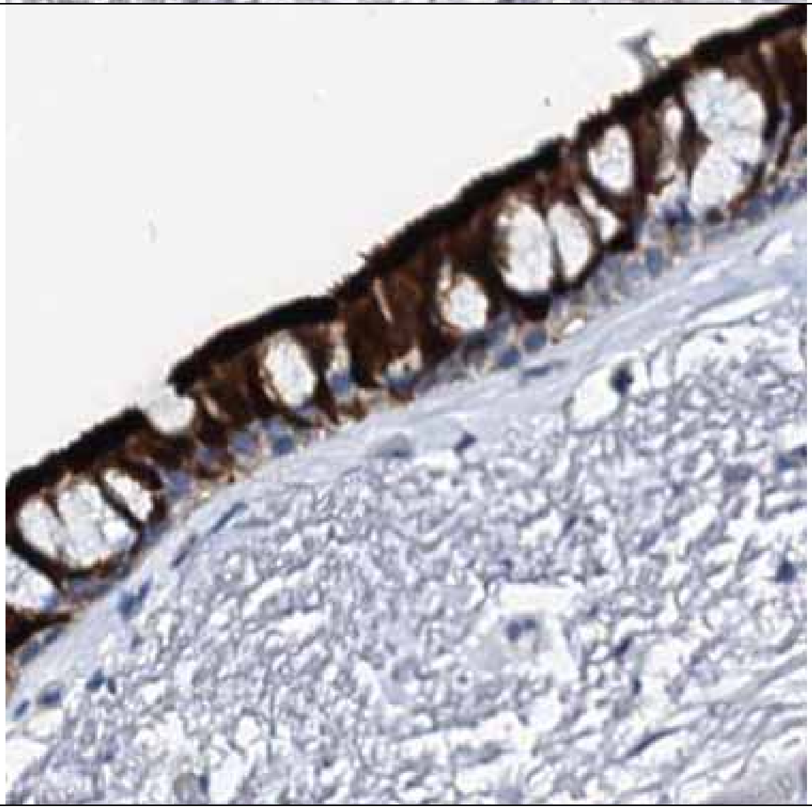	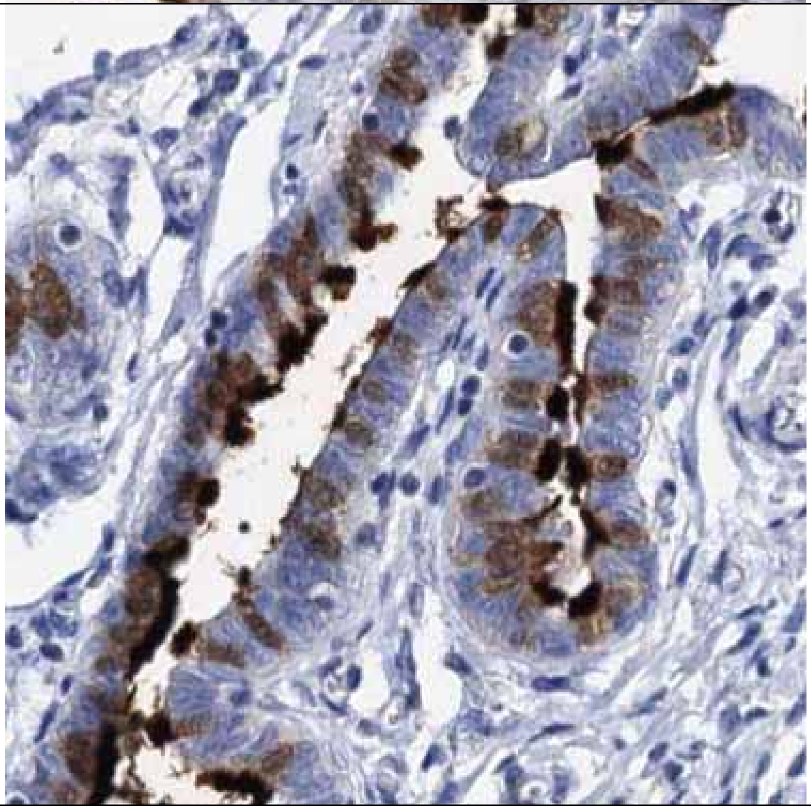	Staining is specific to *cilia*

EFCAB1<br>(antibody<br>HPA023527)<br><br>Category 1	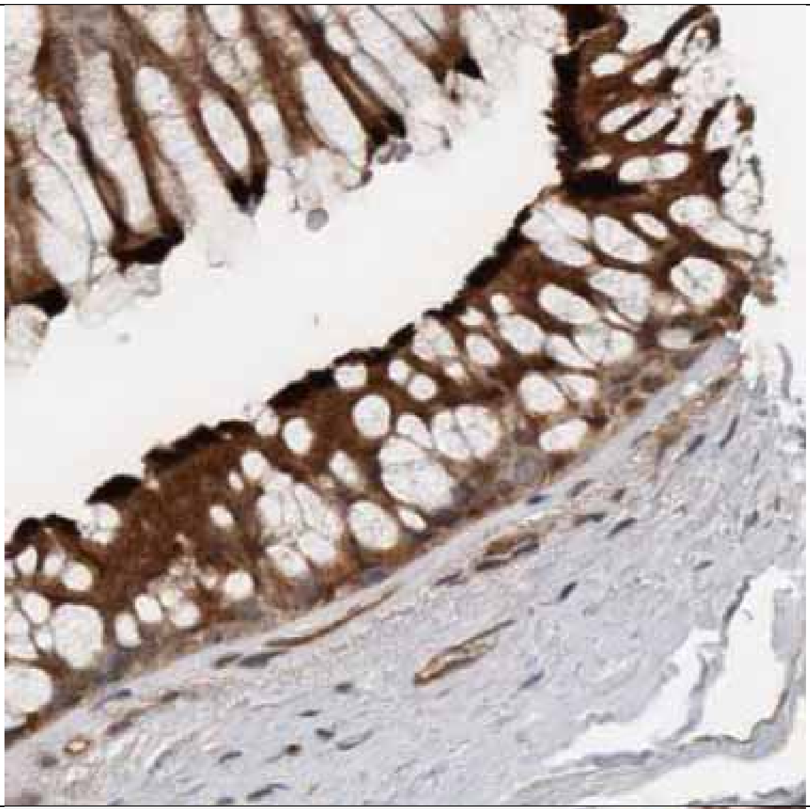	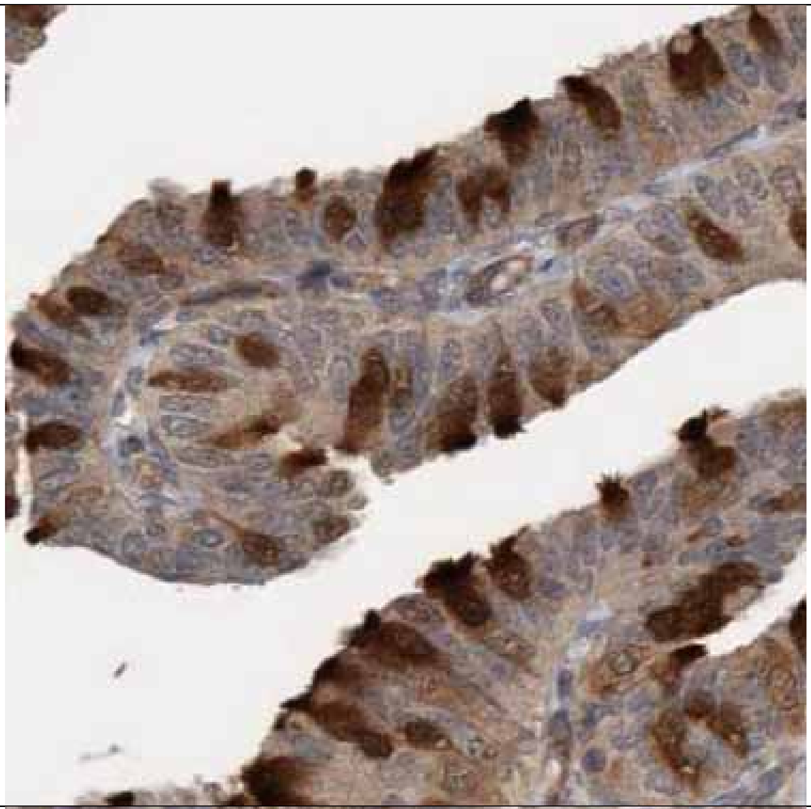	Staining is specific to *cilia*
EFCAB6<br>(antibody<br>HPA003098)<br><br>Category 1	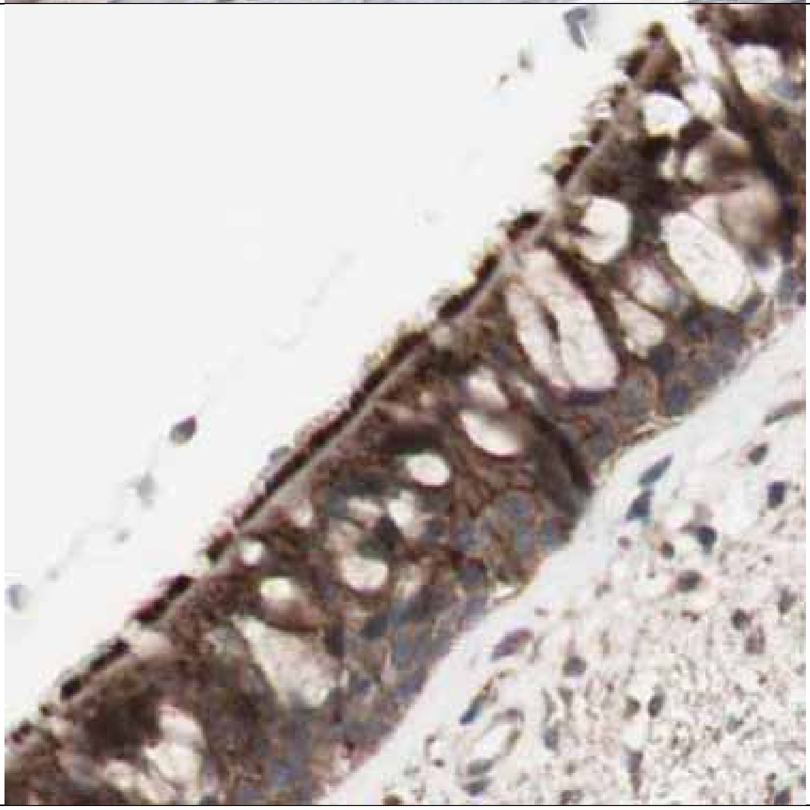	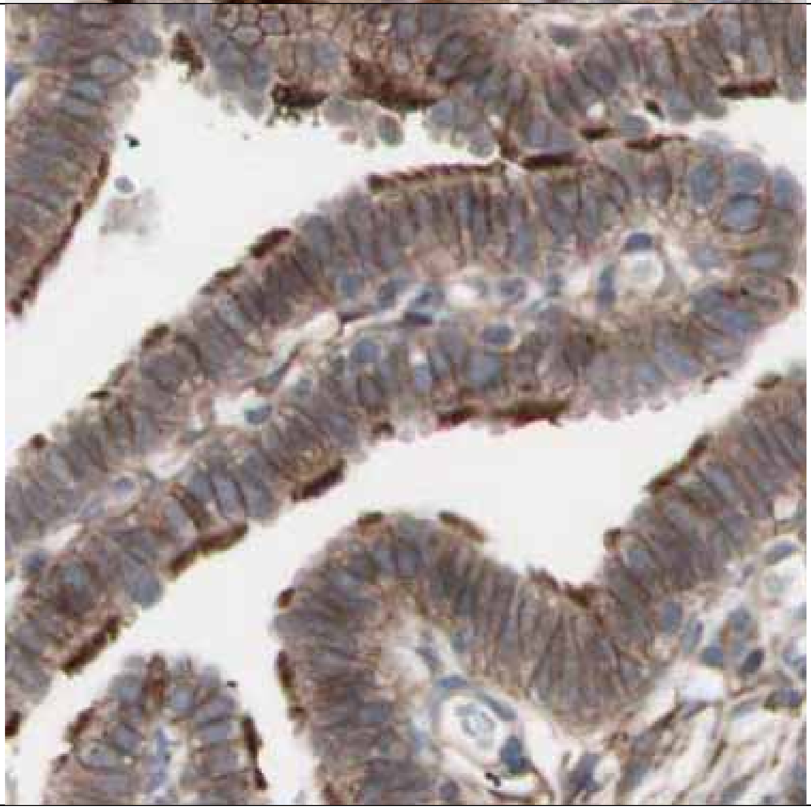	Staining is specific to *cilia*

EFHB (antibody HPA034834)<br><br>Category 1	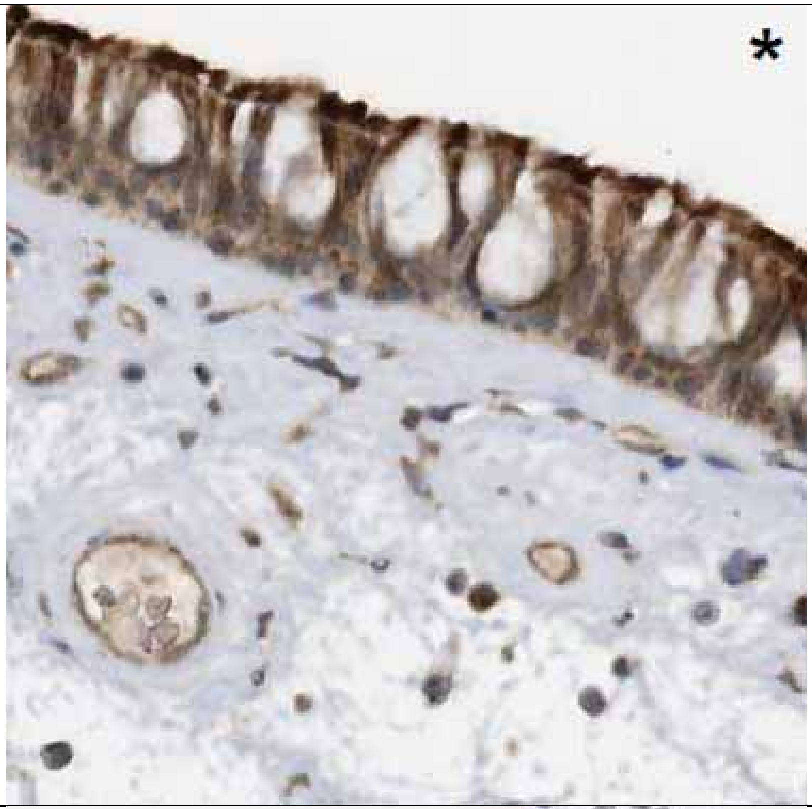	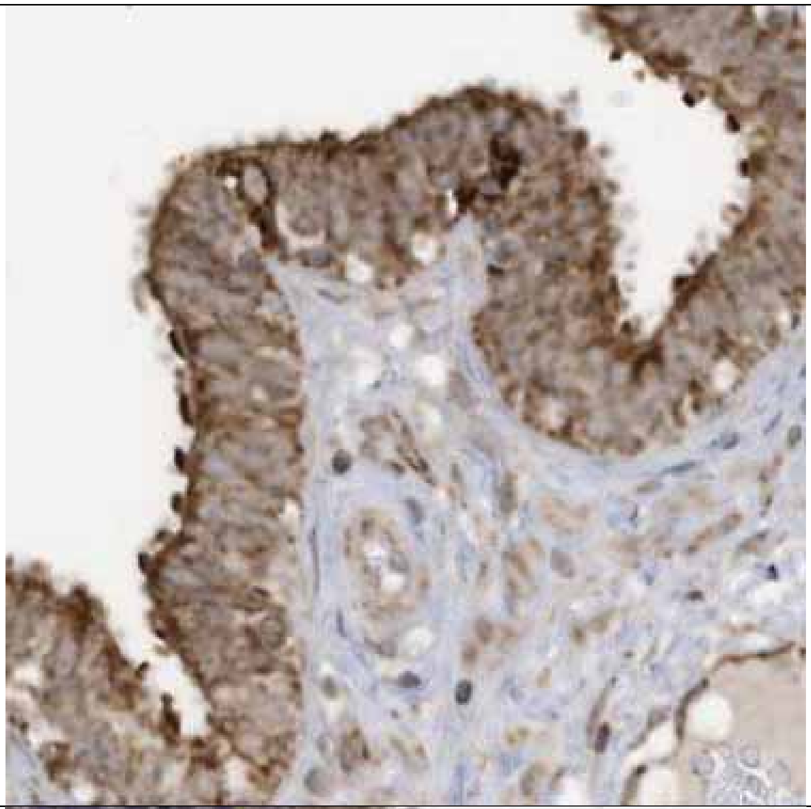	Staining is specific to *cilia*
EFHC1 (antibody CAB020814)<br><br>Category 1	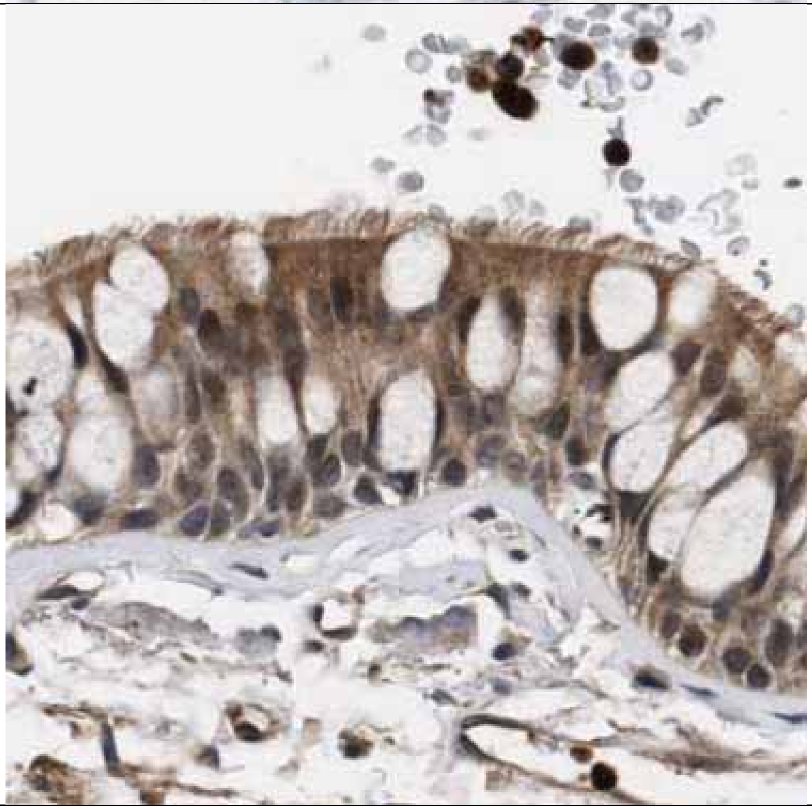	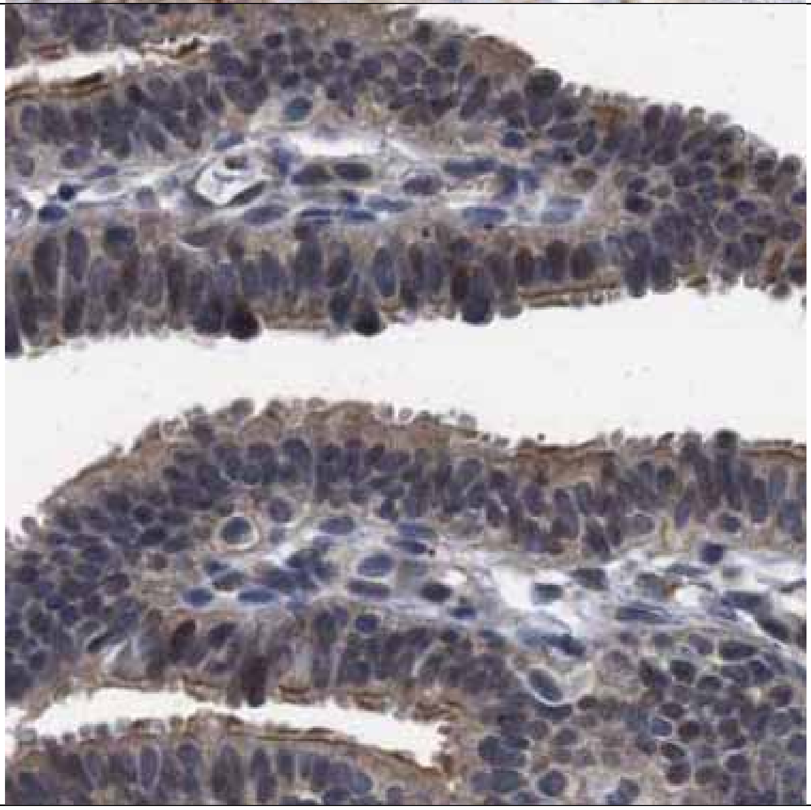	Staining is specific to *cilia*

EFHC2 (antibody HPA034492)<br><br>Category 1	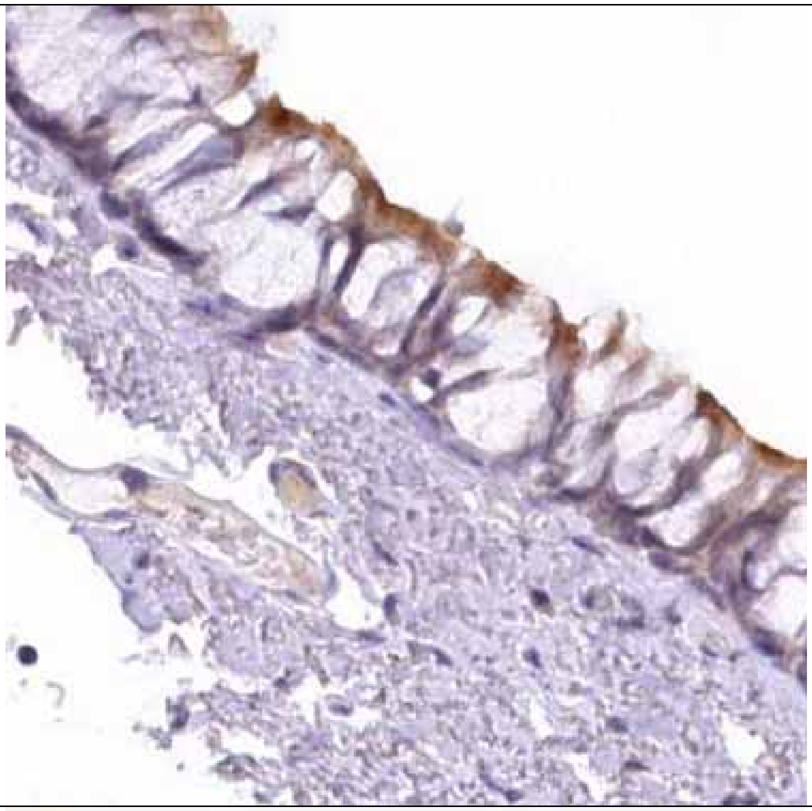	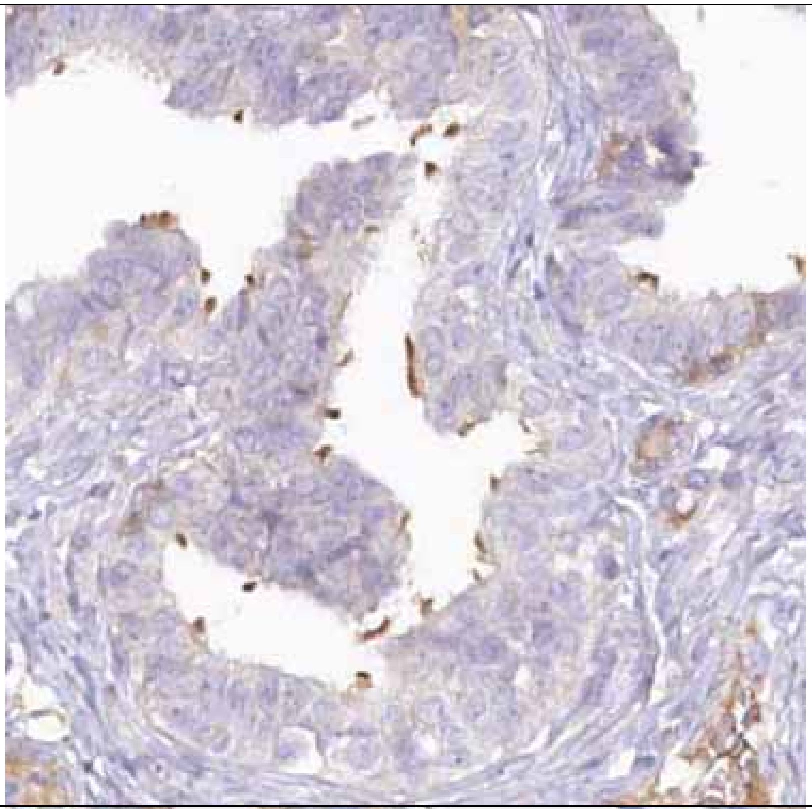	Staining is specific to *cilia*
FLJ23834 (antibody HPA011218)<br><br>Category 1	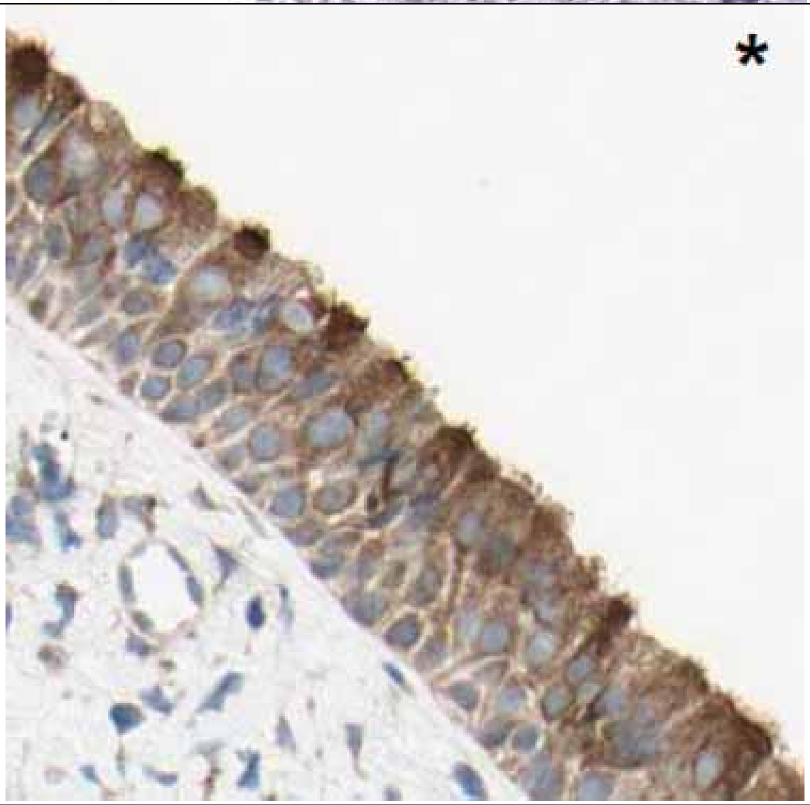 	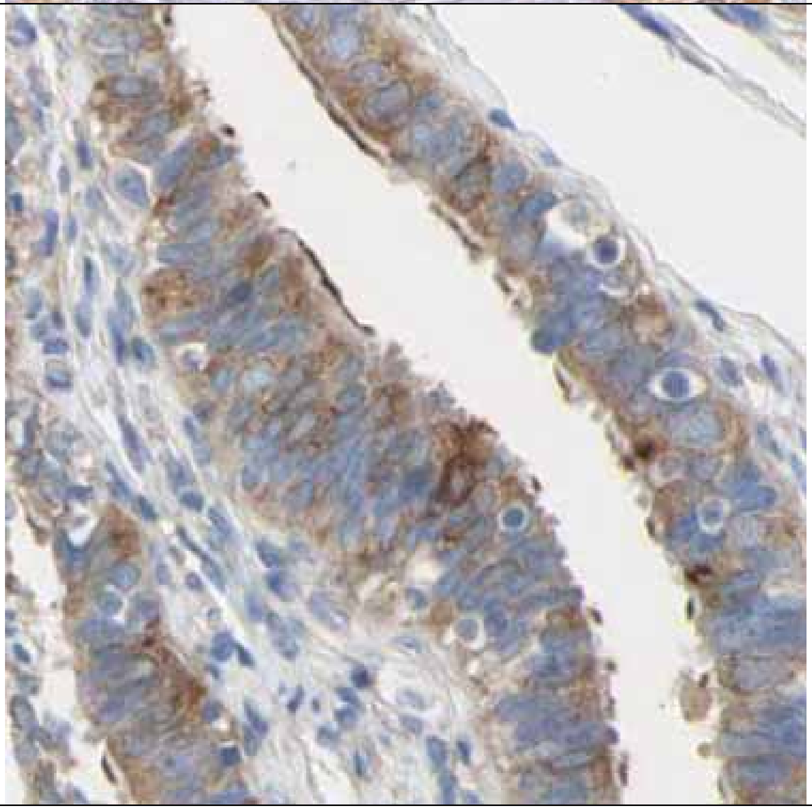	Staining is specific to *cilia*

IQUB (antibody HPA020621)<br><br>Category 1	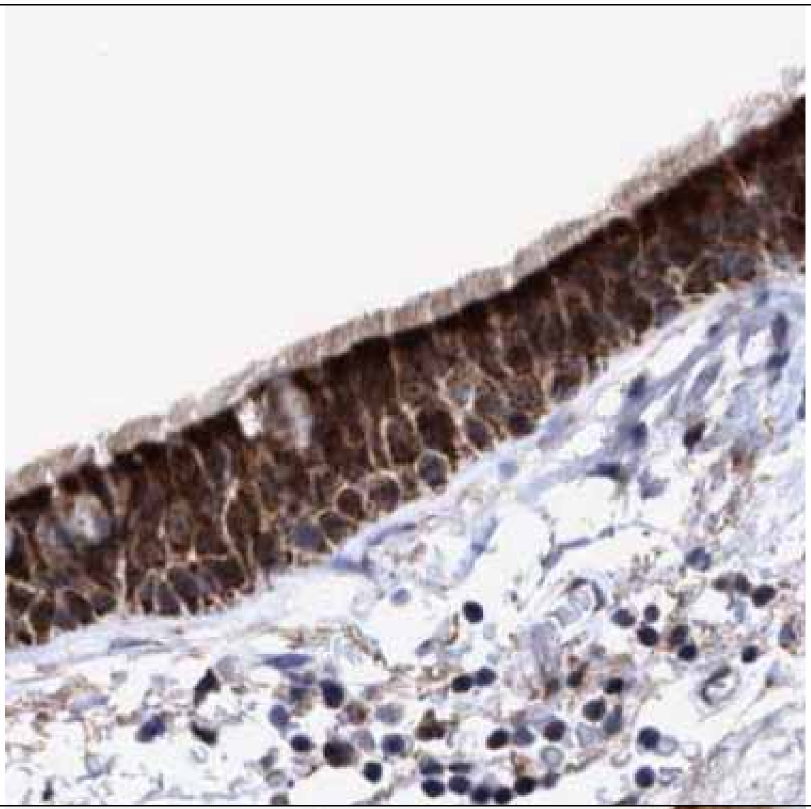	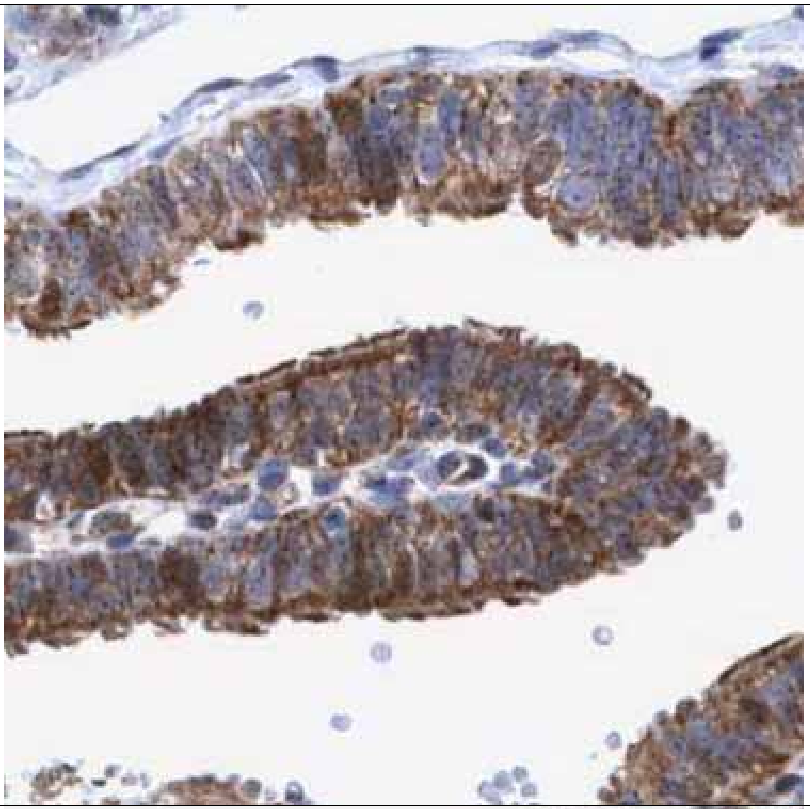	Staining is specific to *cilia*
KIF9 (antibody HPA022031)<br><br>Category 1	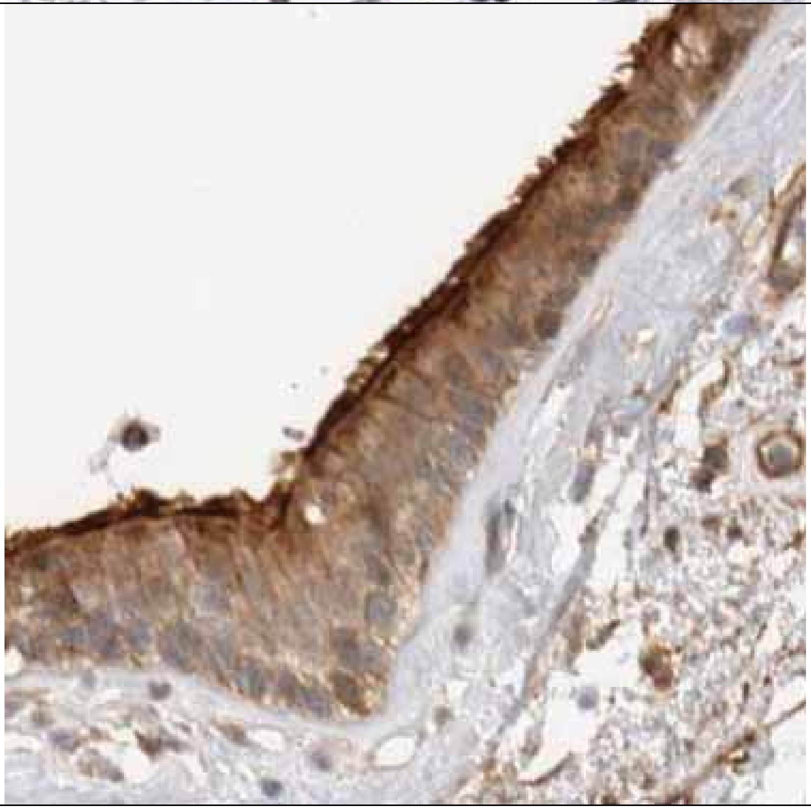	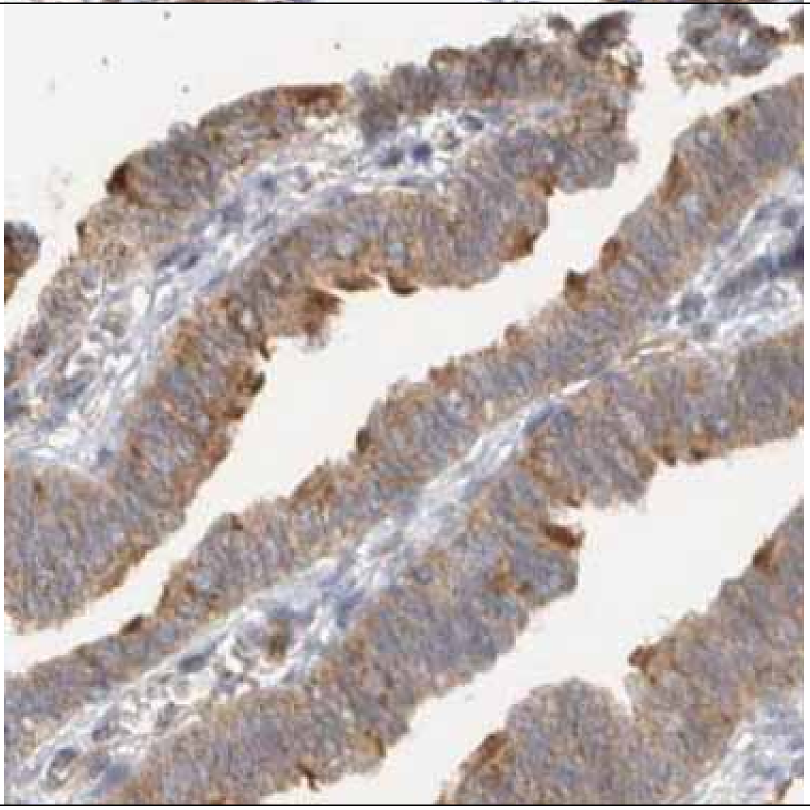	Staining is specific to *cilia*

LCA5<br>(antibody<br>HPA029053)<br><br>Category 1	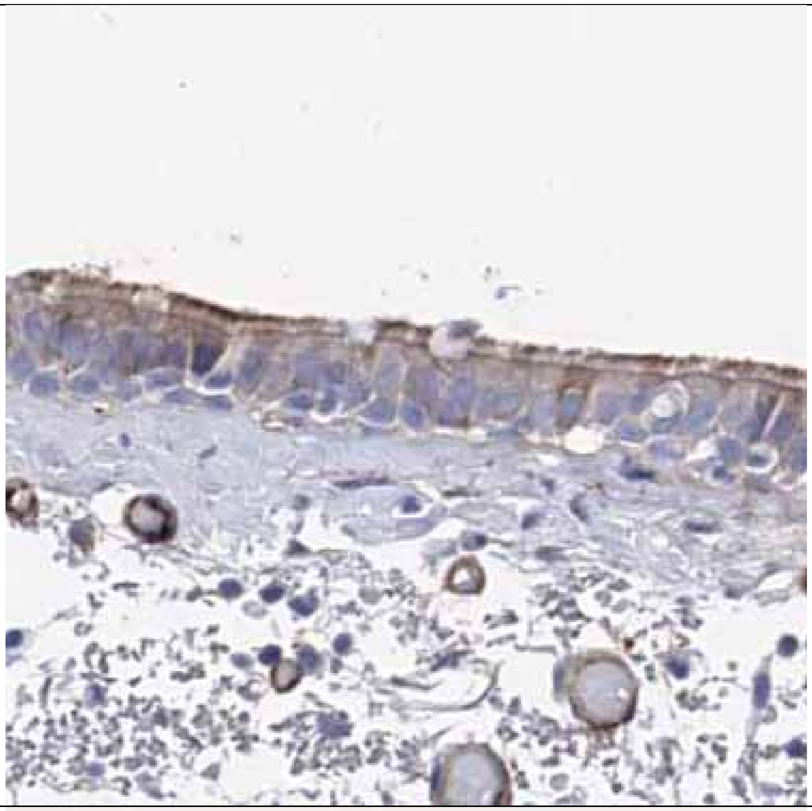	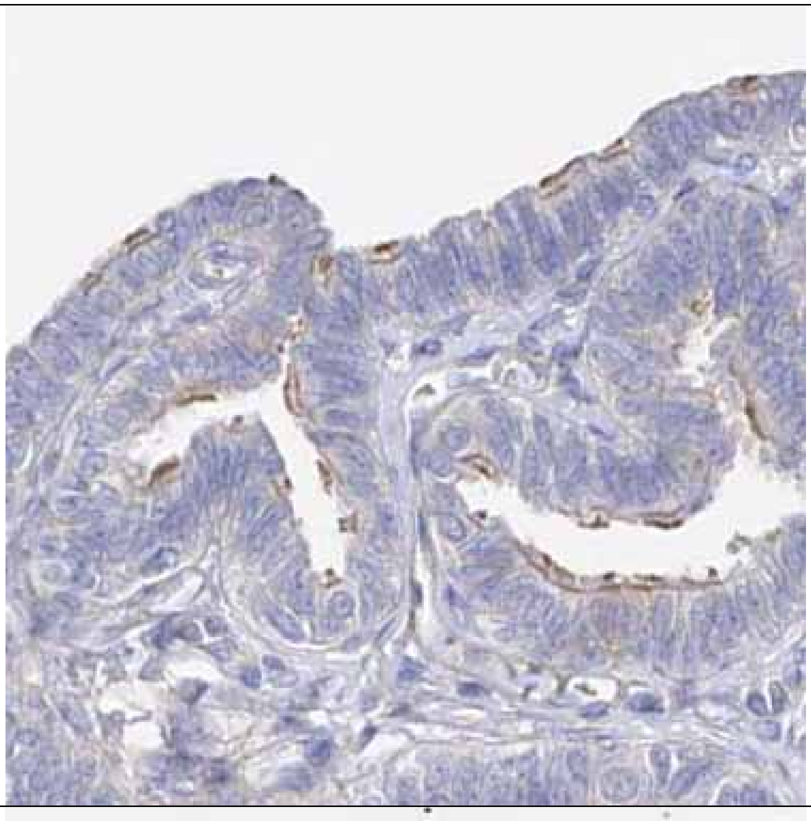	Staining is specific to *cilia*
LRRC23<br>(antibody<br>HPA037766)<br><br>Category 1	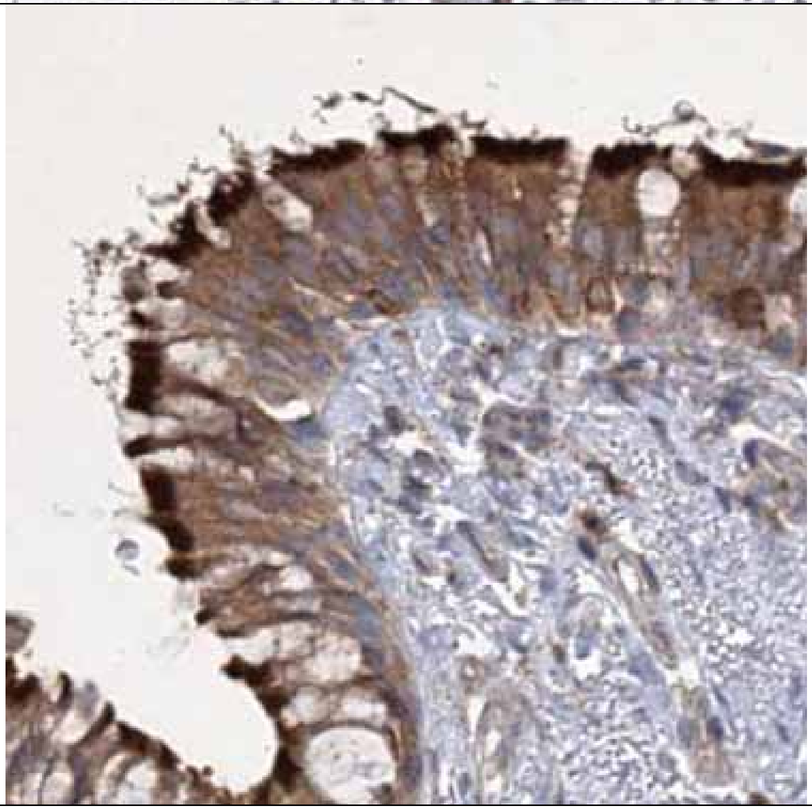	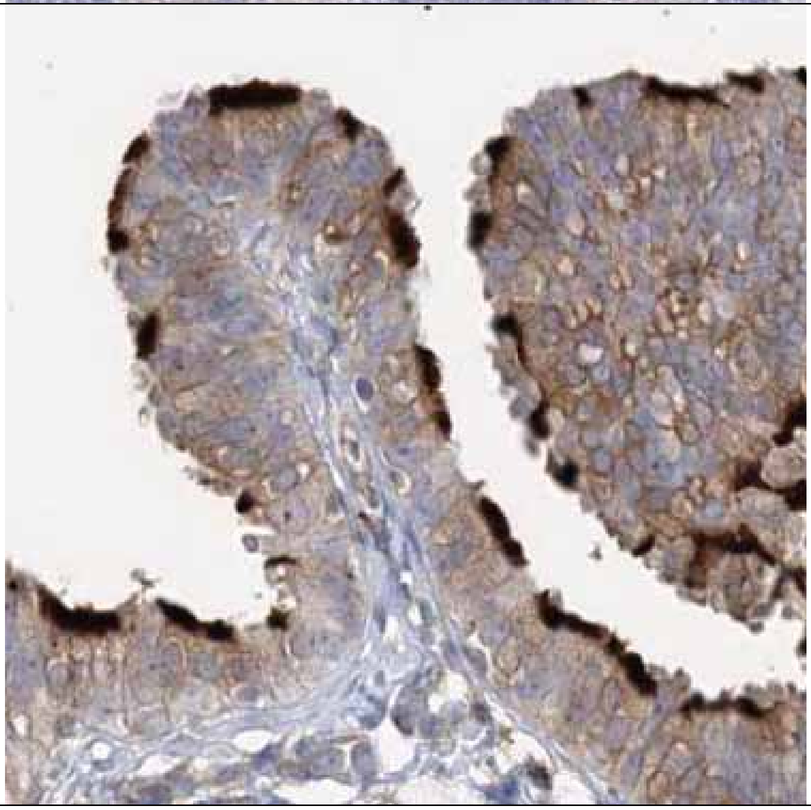	Staining is specific to *cilia*

LRRC34 (antibody HPA035747)<br><br>Category 1	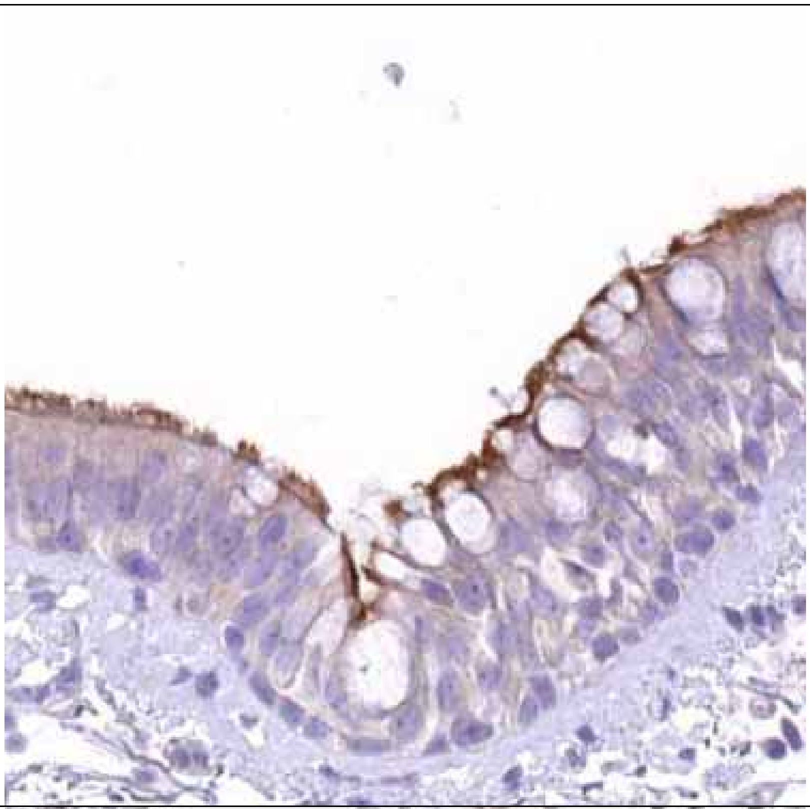	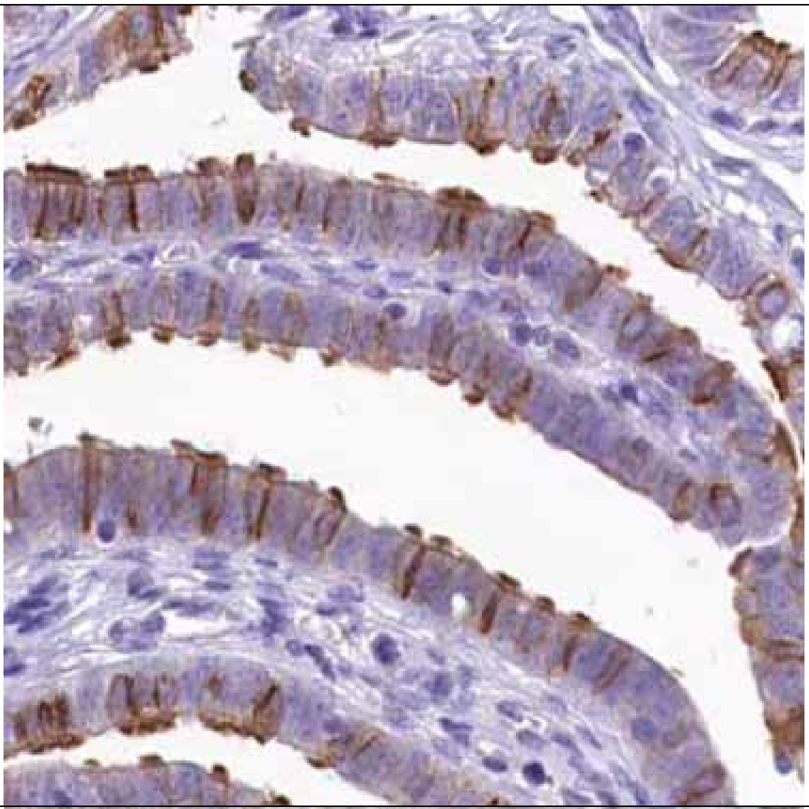	Staining is specific to *cilia*
LRRC48 (antibody HPA036040)<br><br>Category 1	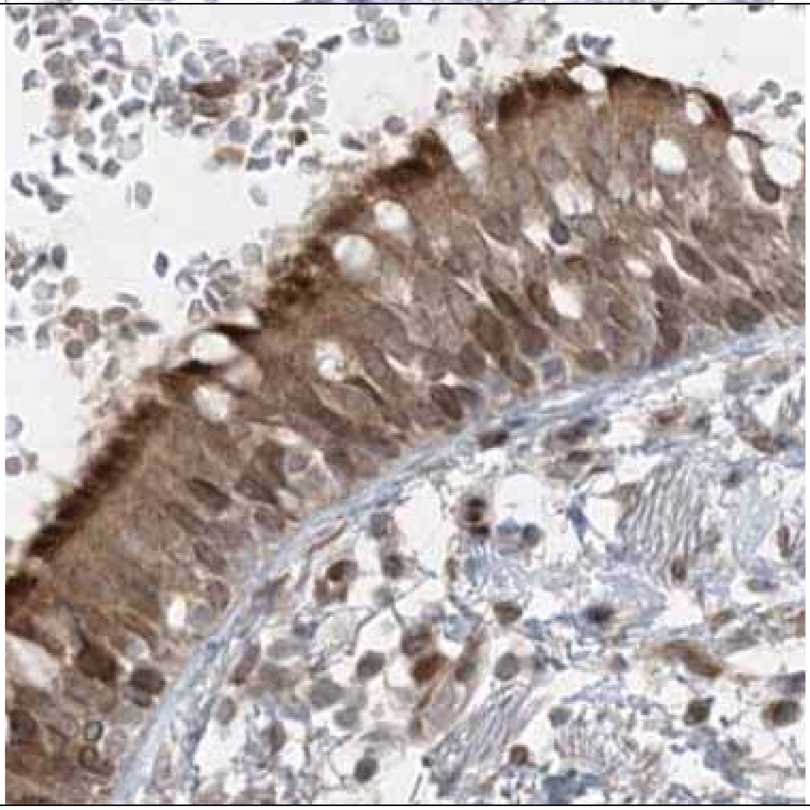	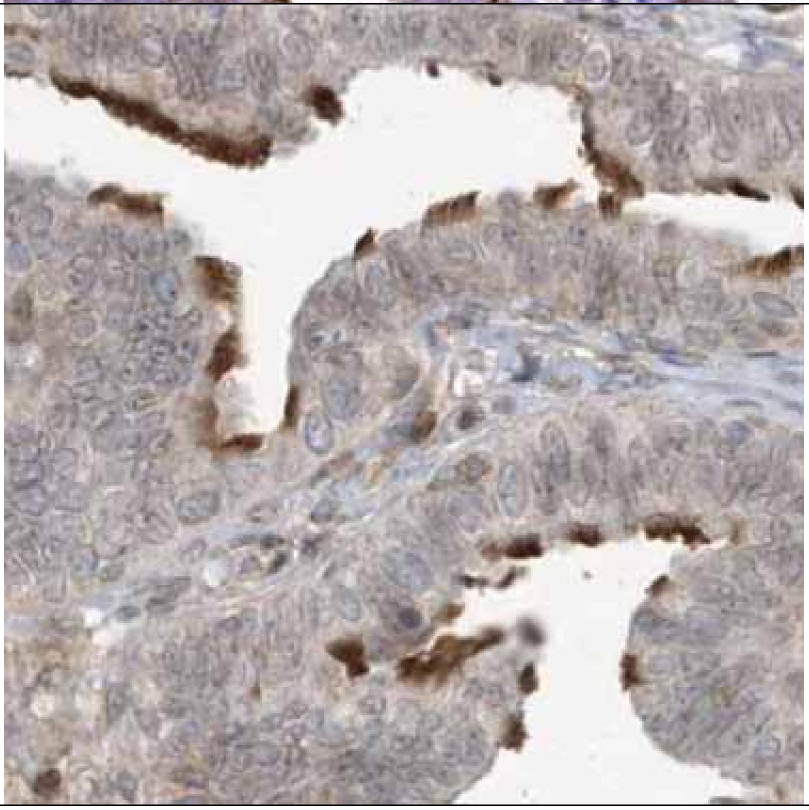	Staining is specific to *cilia*

MAP6 (antibody CAB022600)<br><br>Category 1	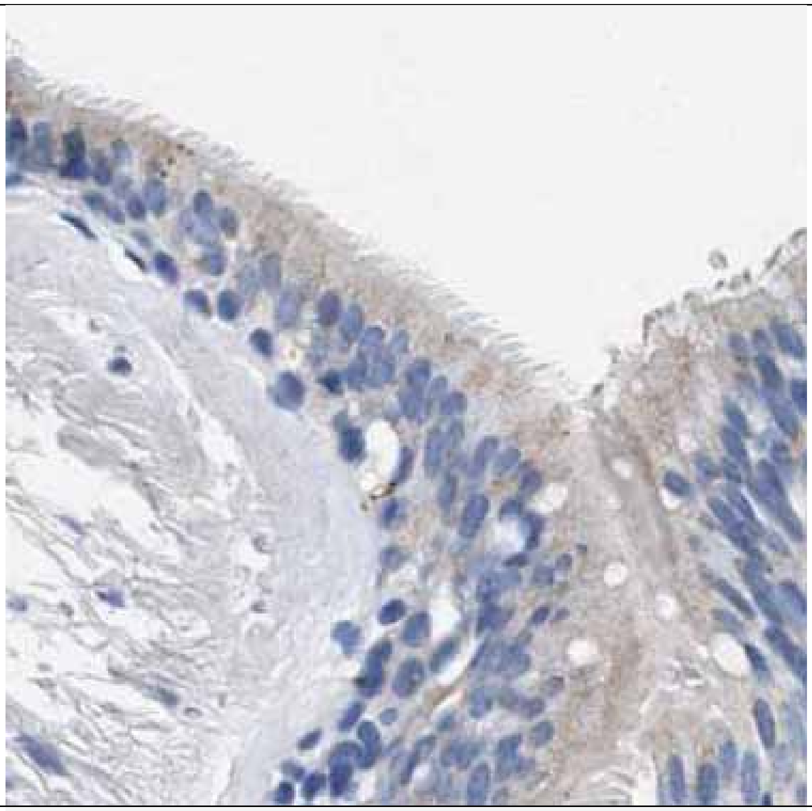	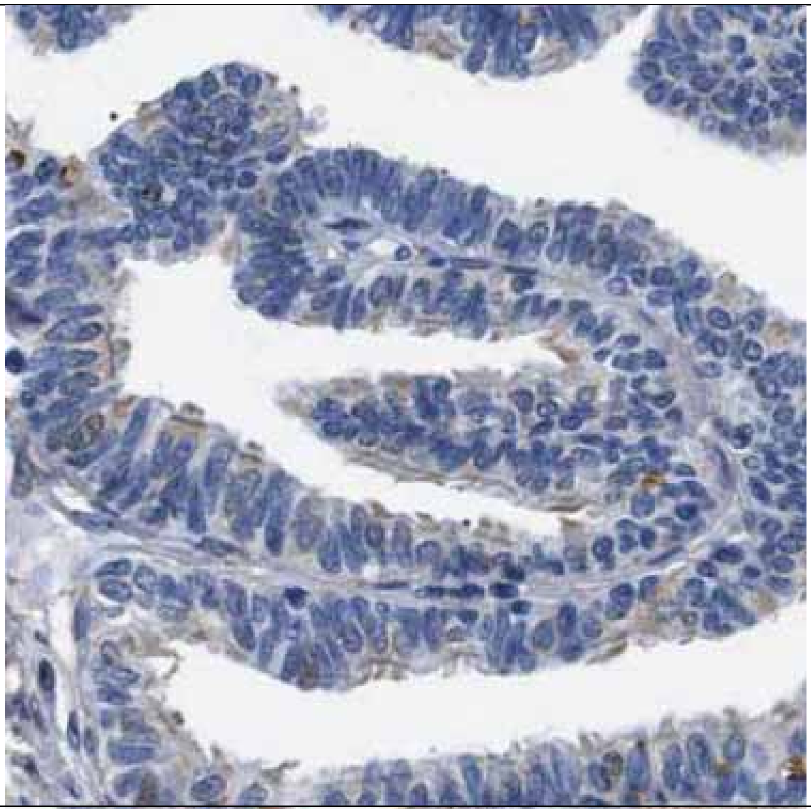	Staining is specific to *cilia*
MDH1B (antibody HPA034741)<br><br>Category 1	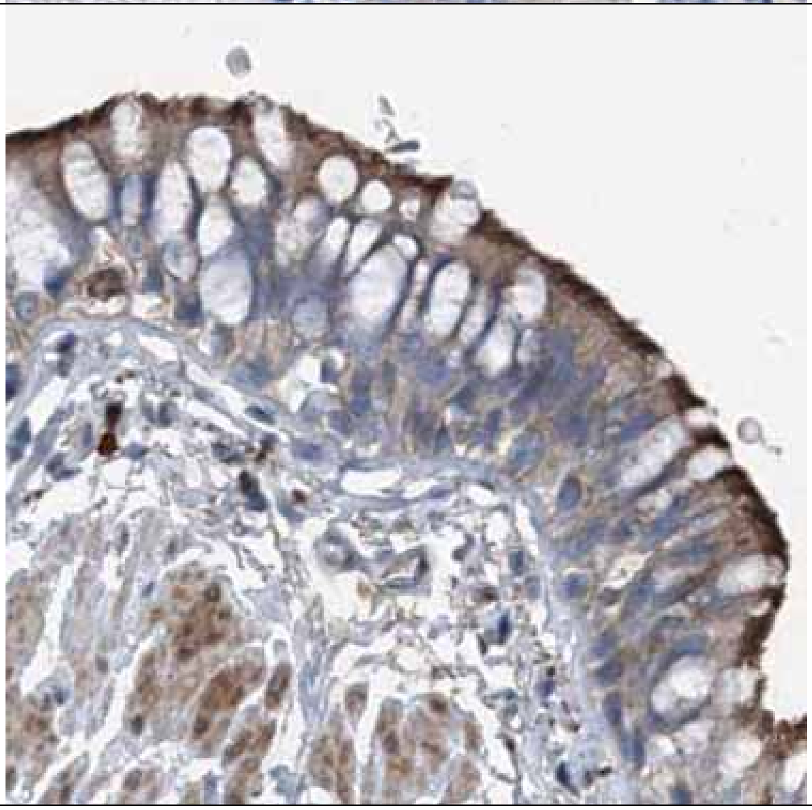	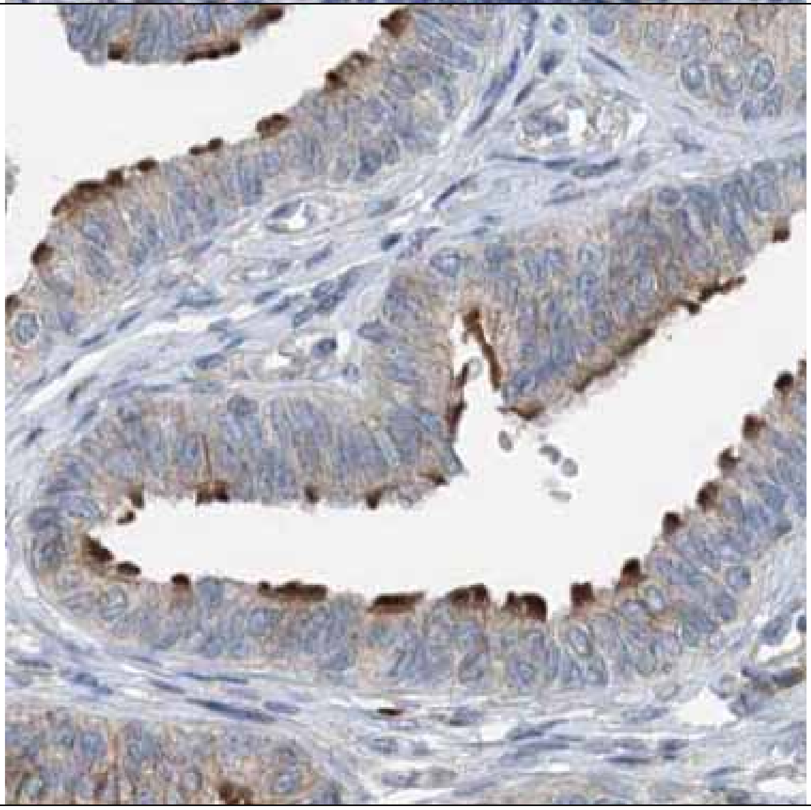	Staining is specific to *cilia*

MLF1<br>(antibody<br>HPA017903)<br><br>Category 1	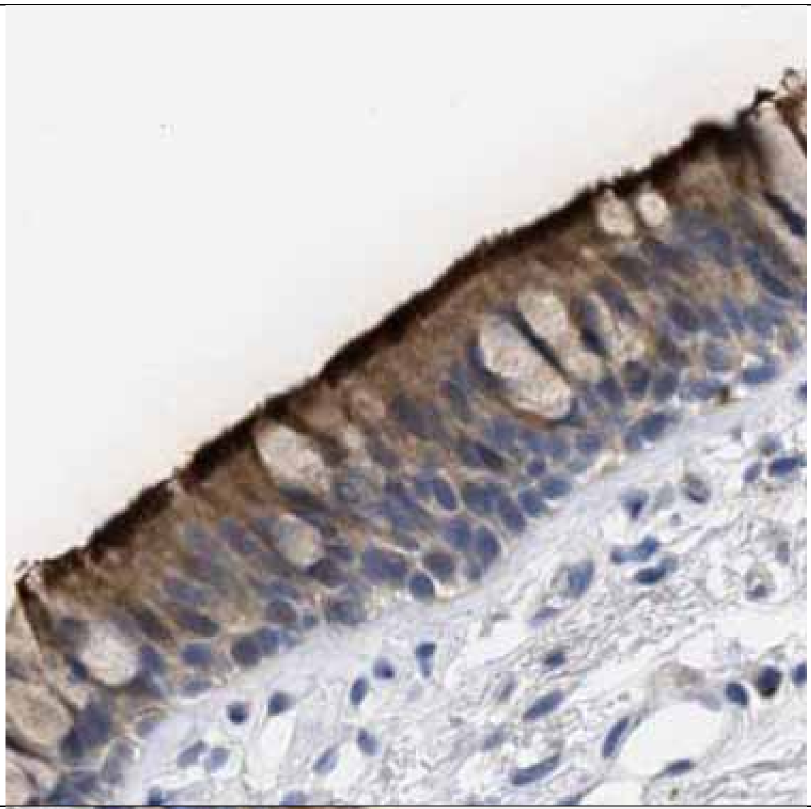	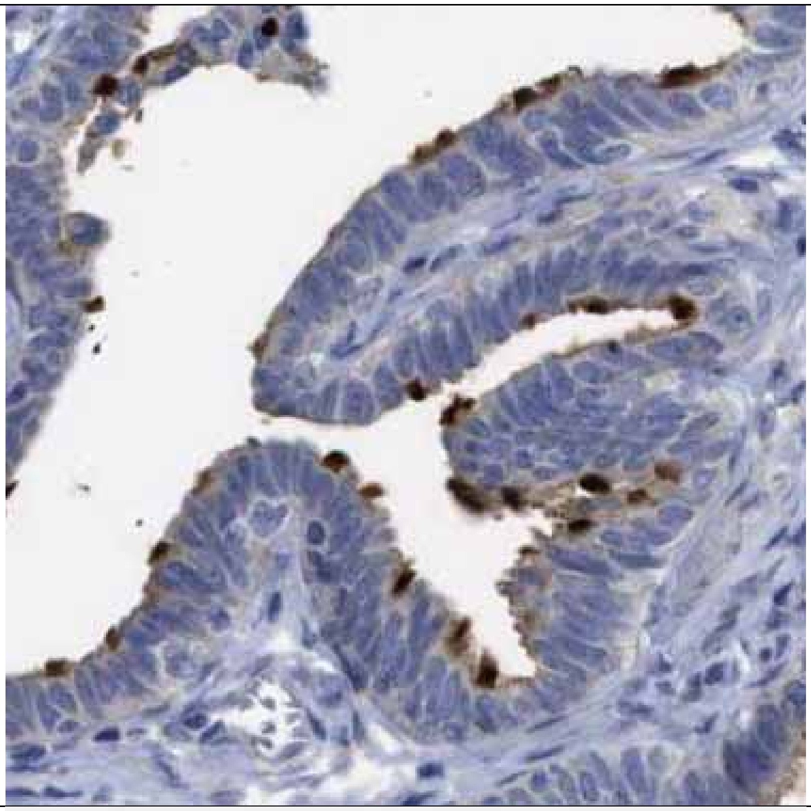	Staining is specific to *cilia*
MORN5<br>(antibody<br>HPA020930)<br><br>Category 1	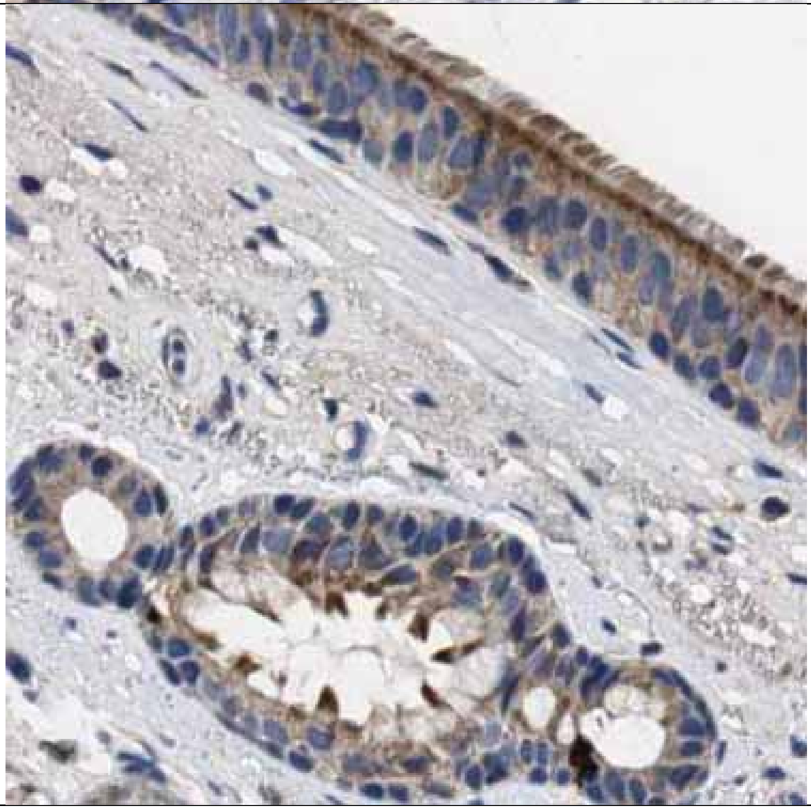	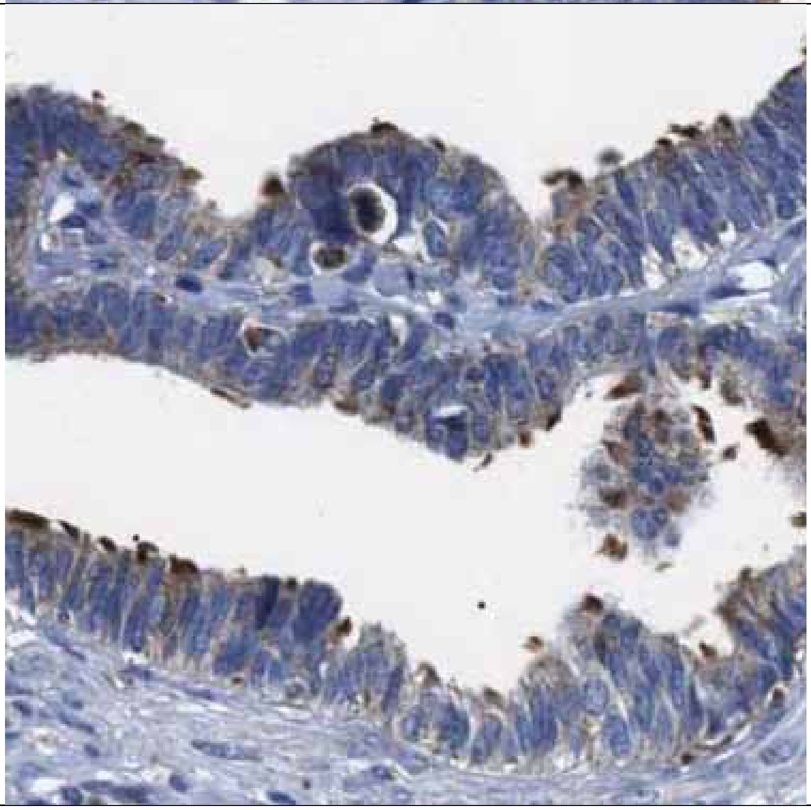	Staining is specific to *cilia*

MS4A8B (antibody HPA007318)<br><br>Category 1	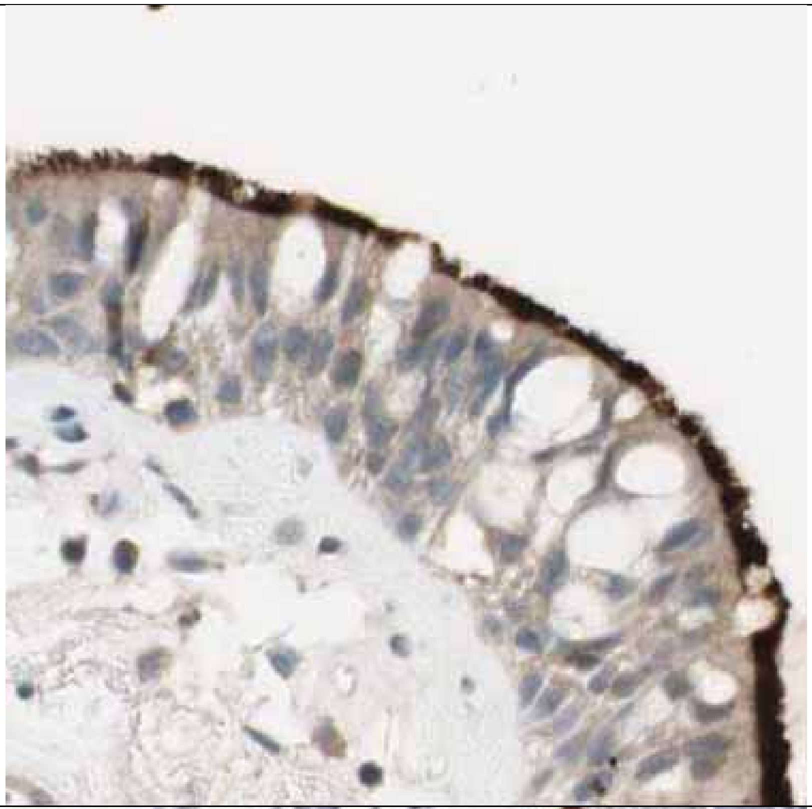	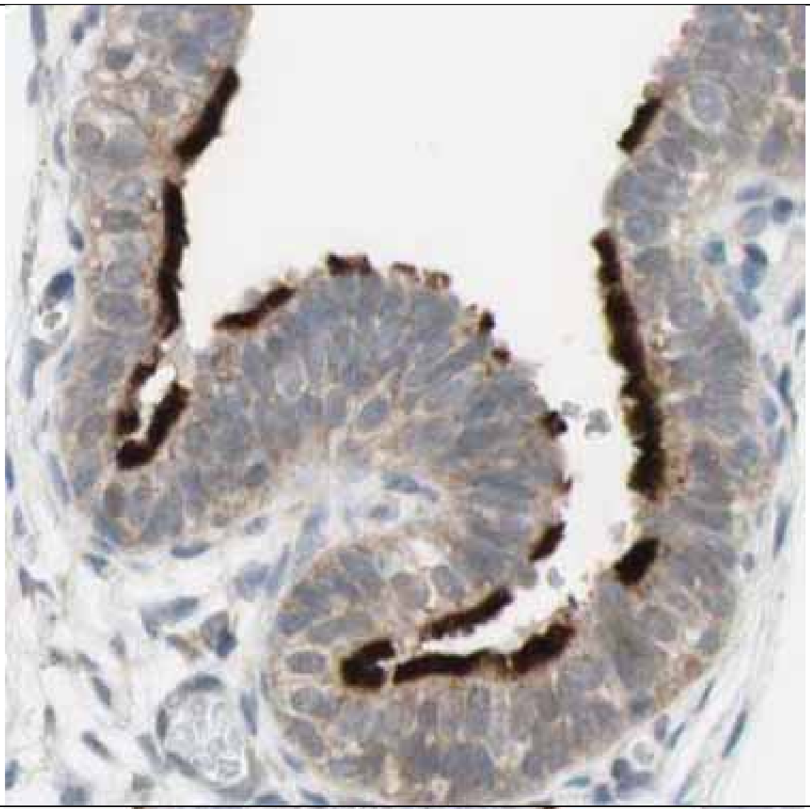	Staining is specific to *cilia*
PPIL6 (antibody HPA036717)<br><br>Category 1	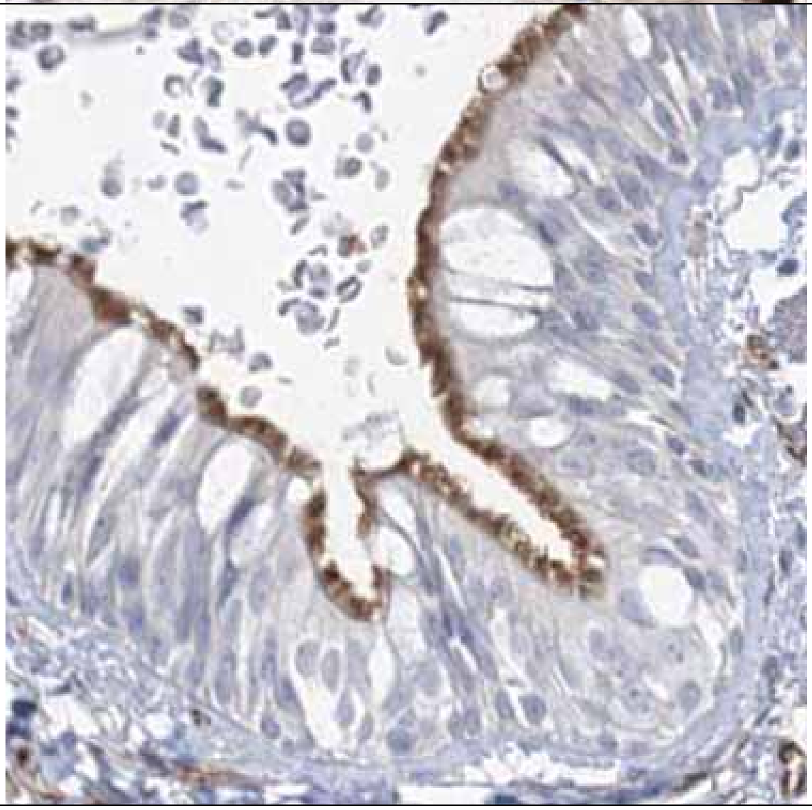	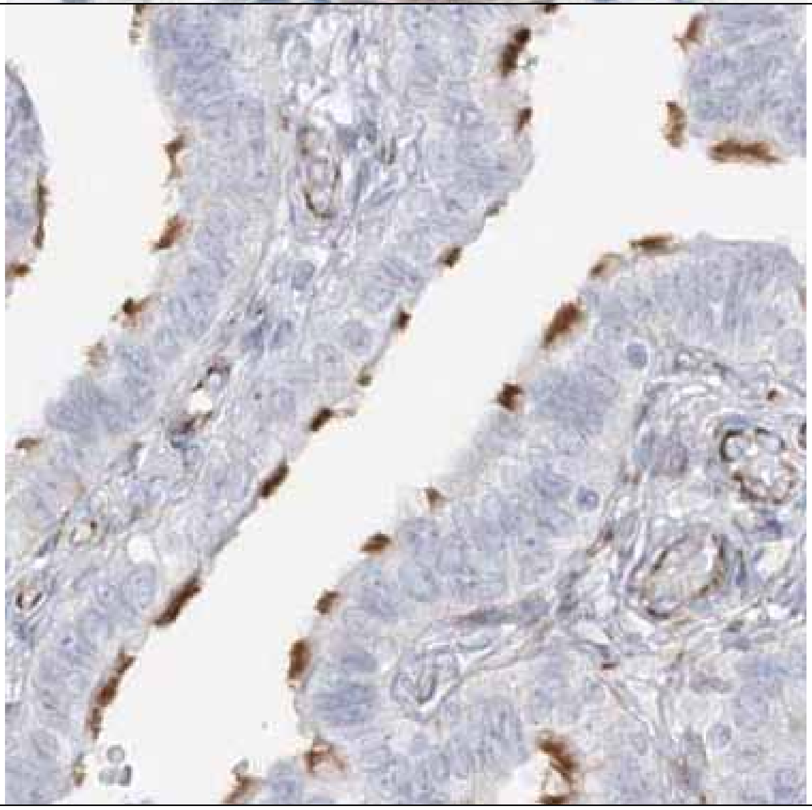	Staining is specific to *cilia*

PROM1 (antibody HPA031053)<br><br>Category 1	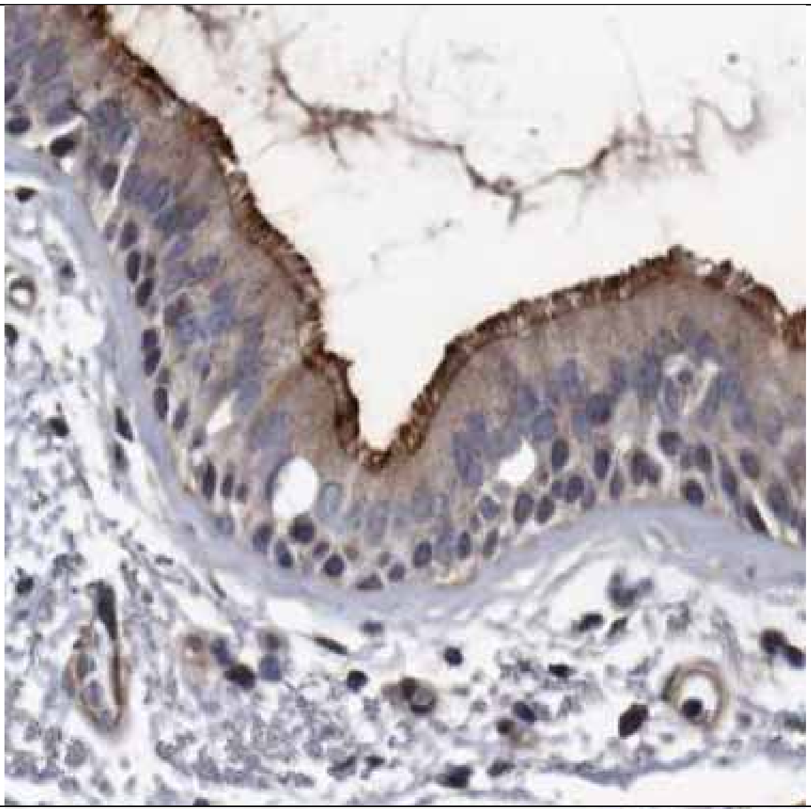	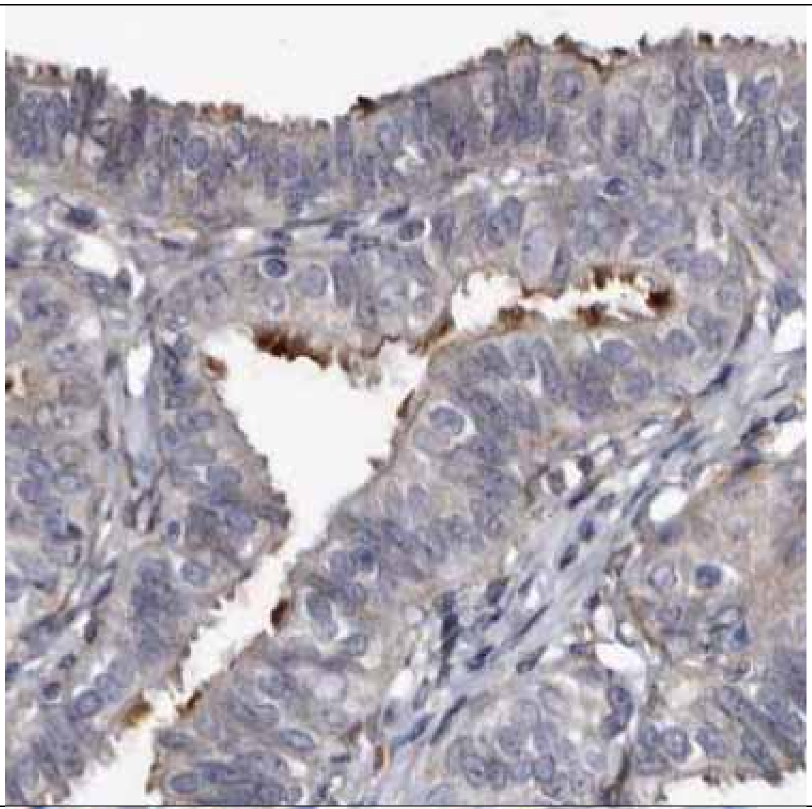	Staining is specific to *cilia*
RIBC2 (antibody HPA003210)<br><br>Category 1	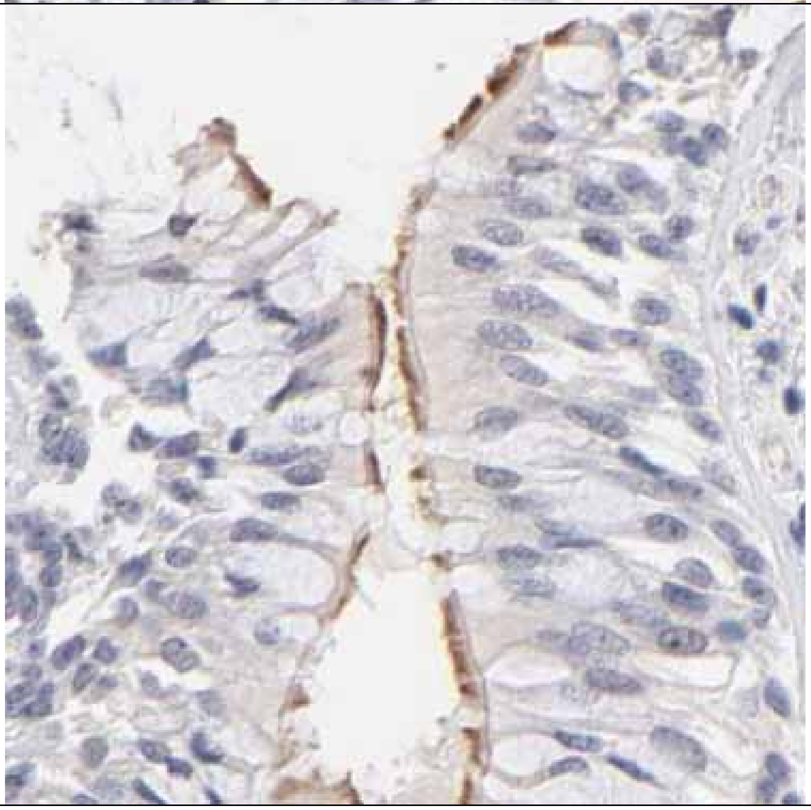	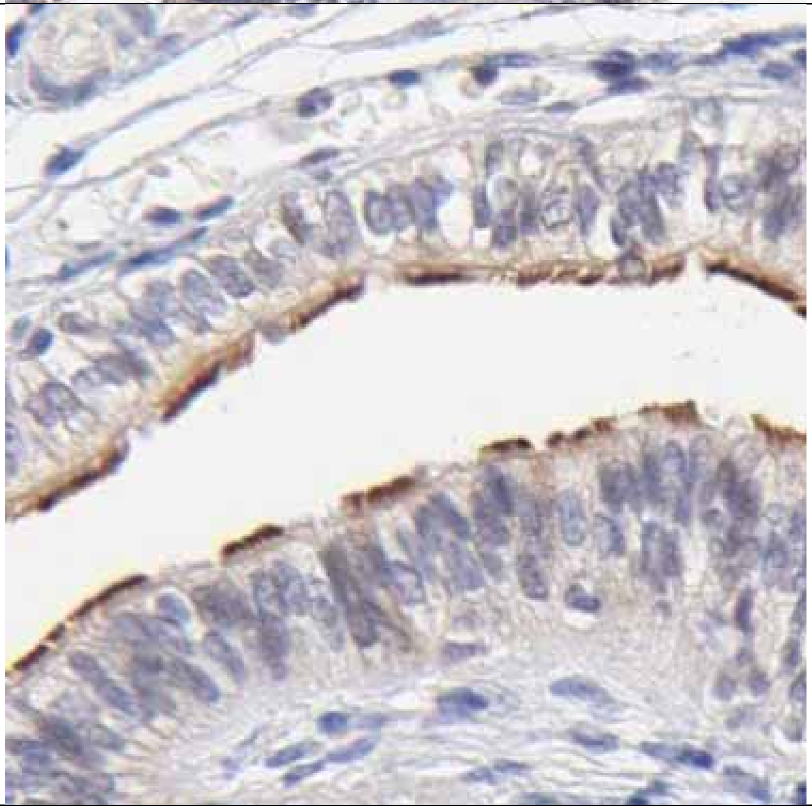	Staining is specific to *cilia*

ROPN1L (antibody HPA039193)<br><br>Category 1	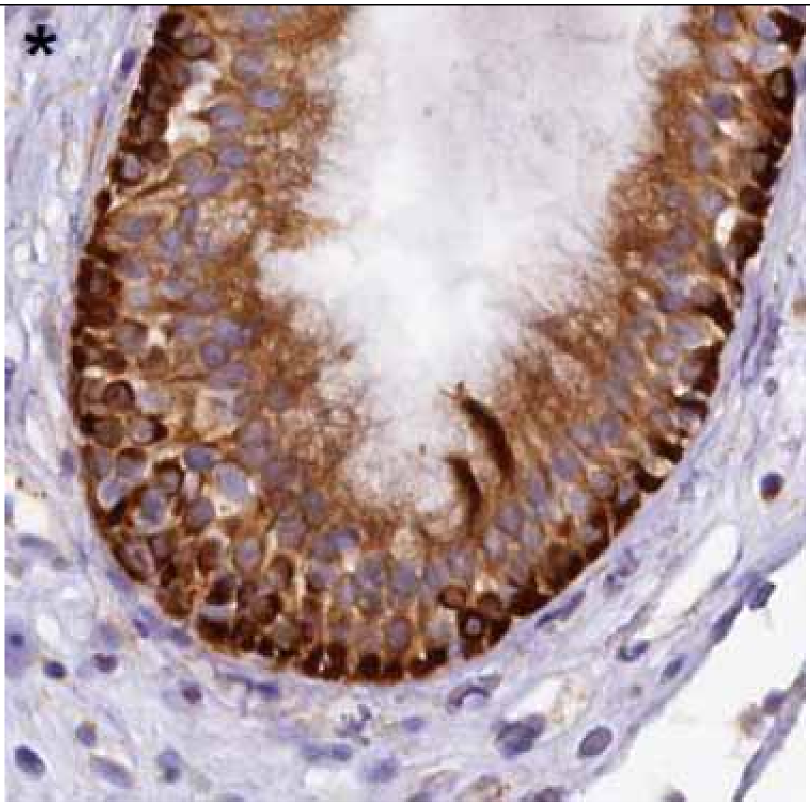	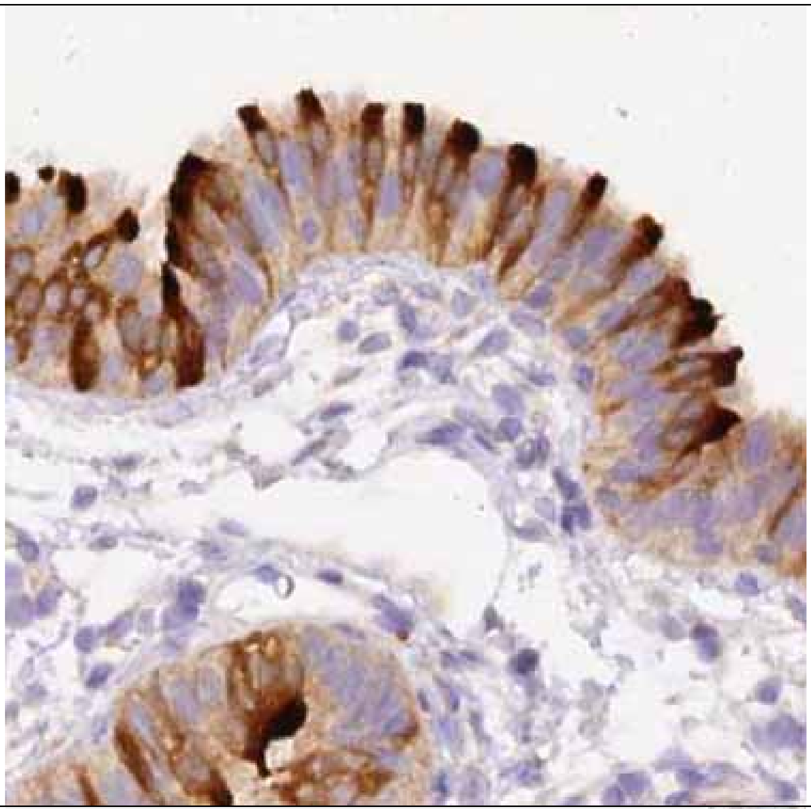	Staining is specific to *cilia*
RPGR (antibody HPA001593)<br><br>Category 1	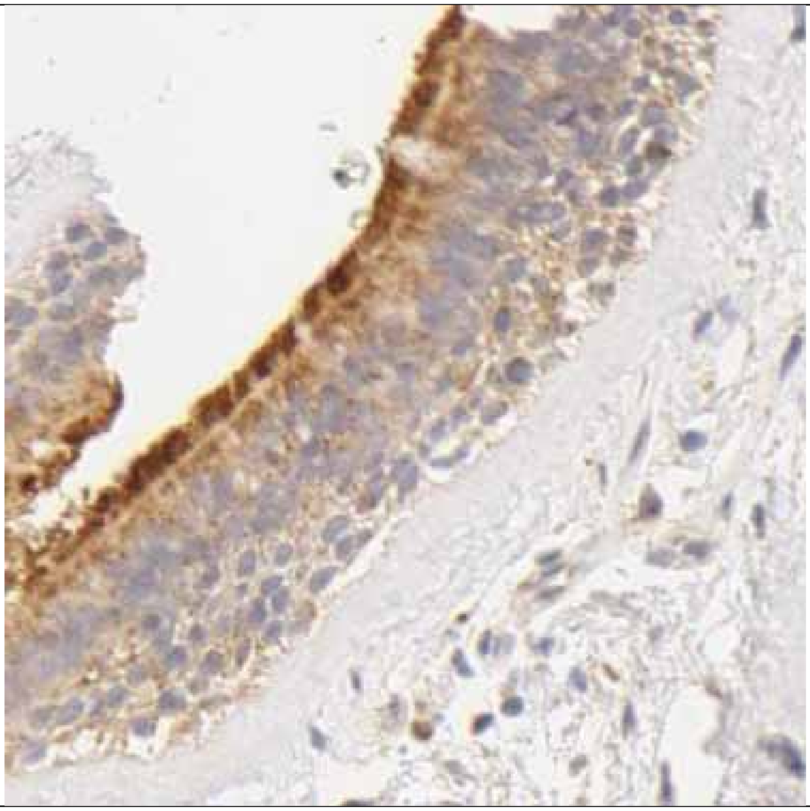	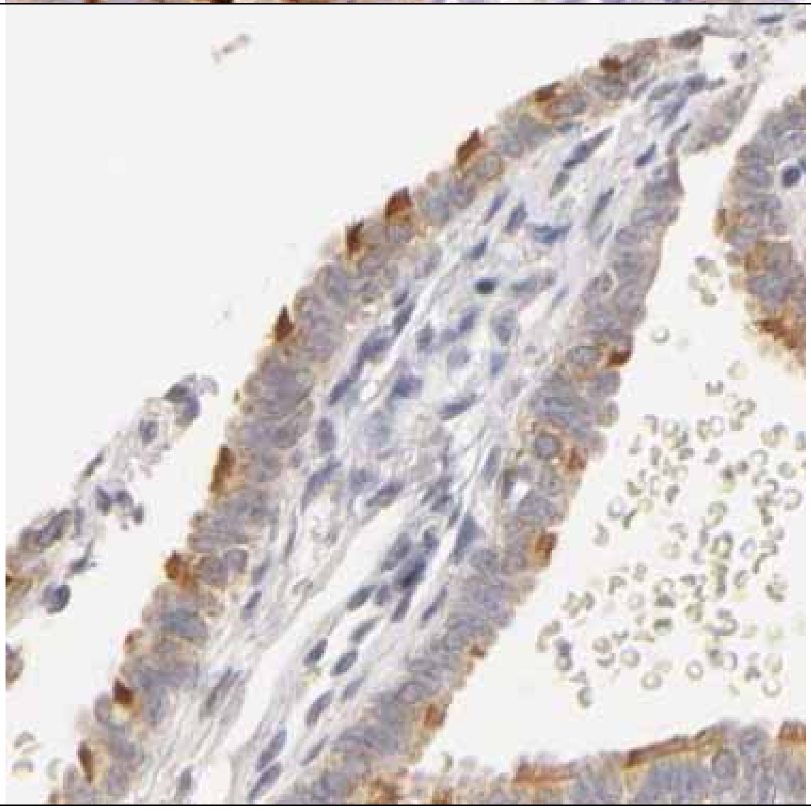	Staining is specific to *cilia*

RSPH1 (antibody HPA016816)<br><br>Category 1	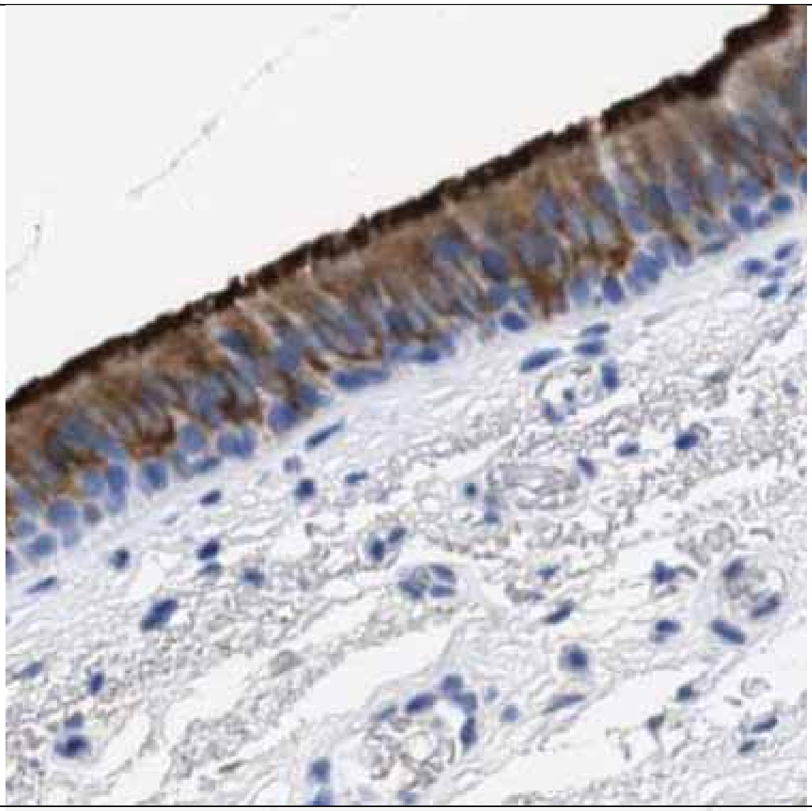	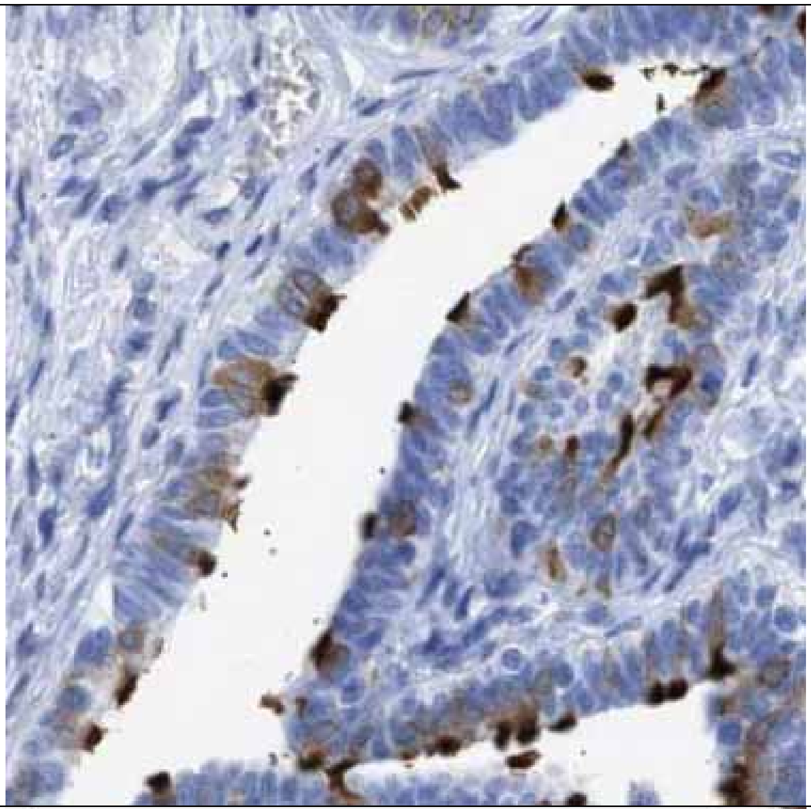	Staining is specific to *cilia*
RSPH4A (antibody HPA031196)<br><br>Category 1	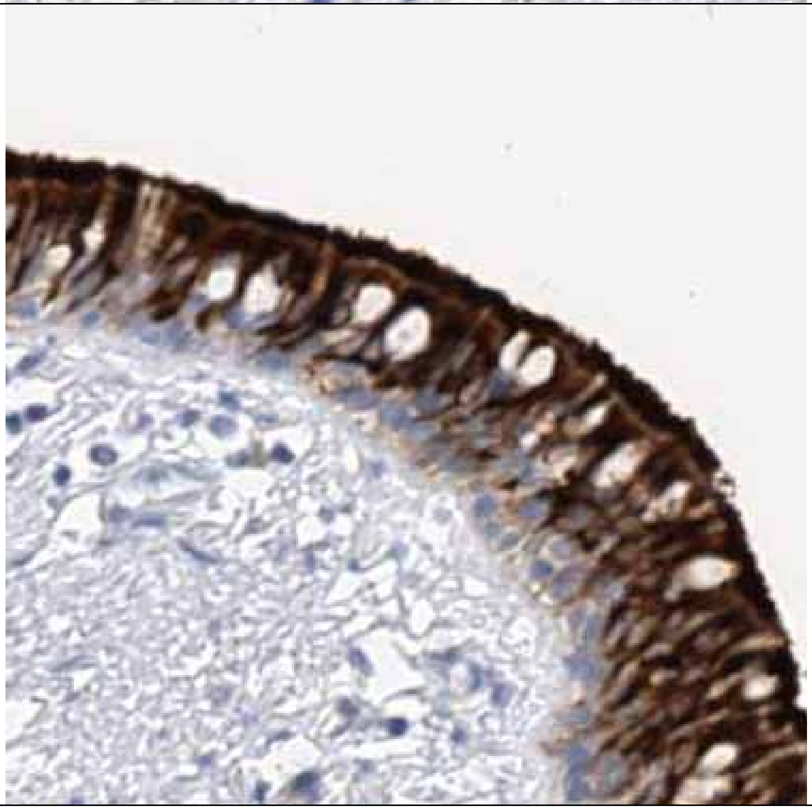	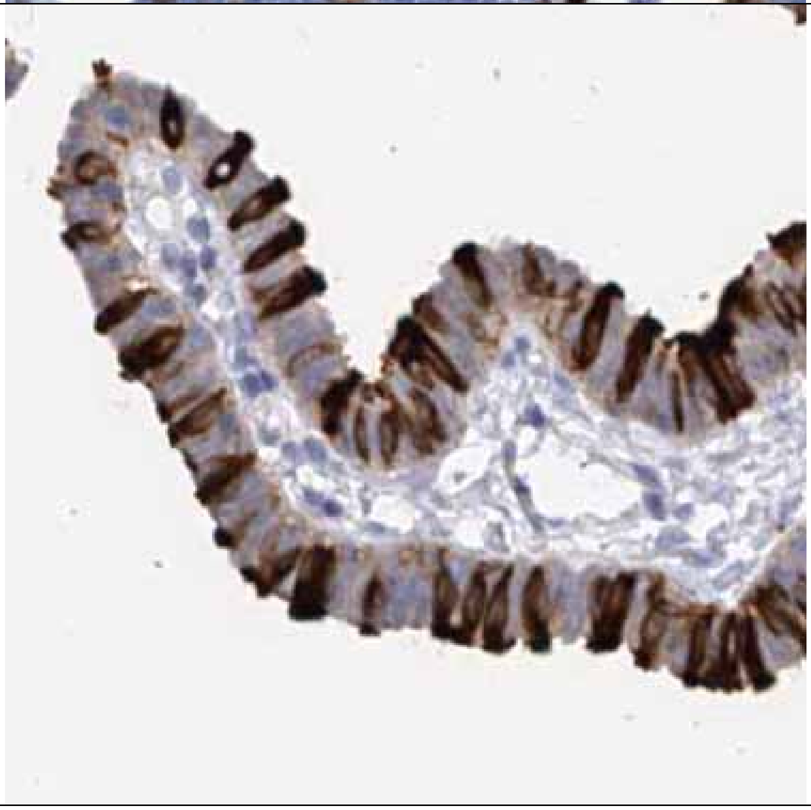	Staining is specific to *cilia*

RSPH9 (antibody HPA031703)<br><br>Category 1	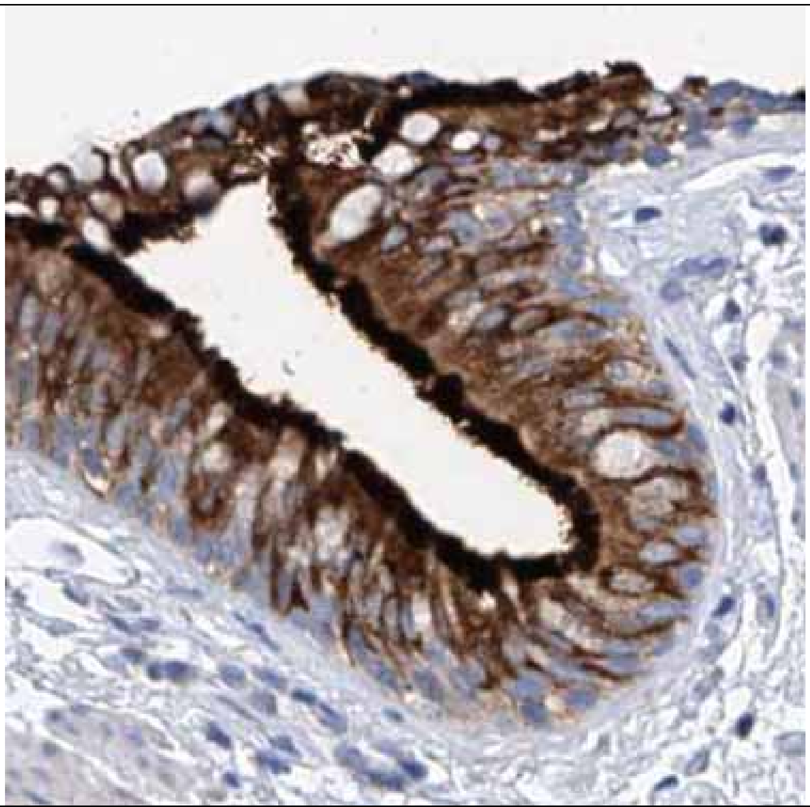	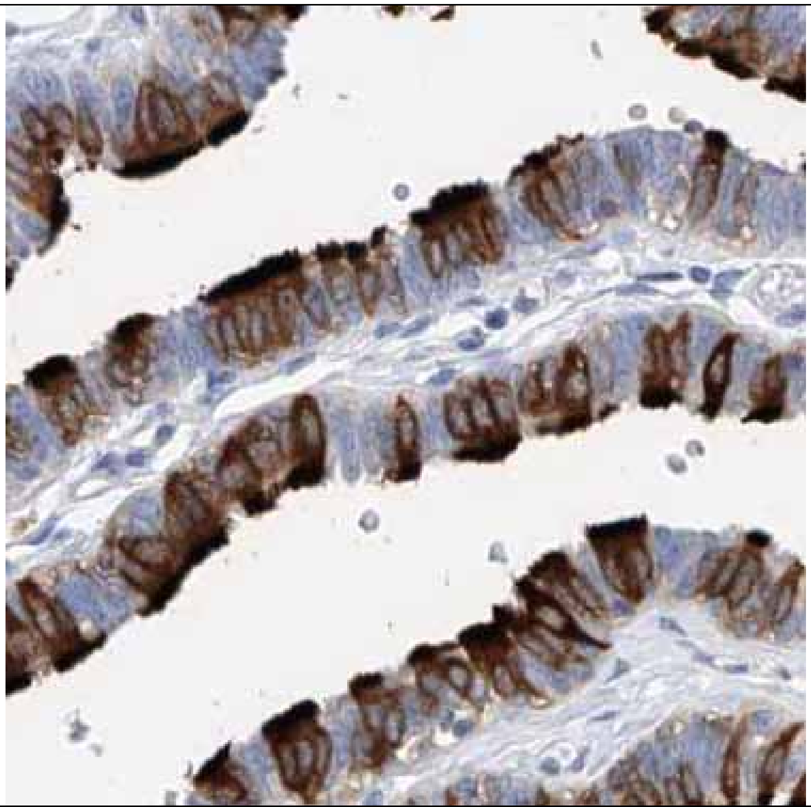	Staining is specific to *cilia*
RTDR1 (antibody HPA018420)<br><br>Category 1	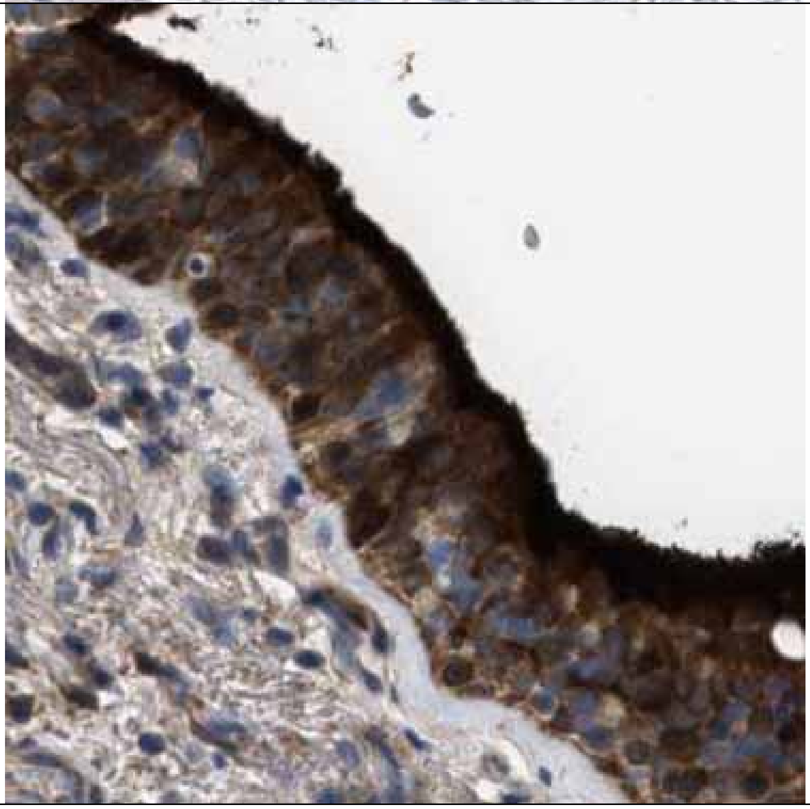	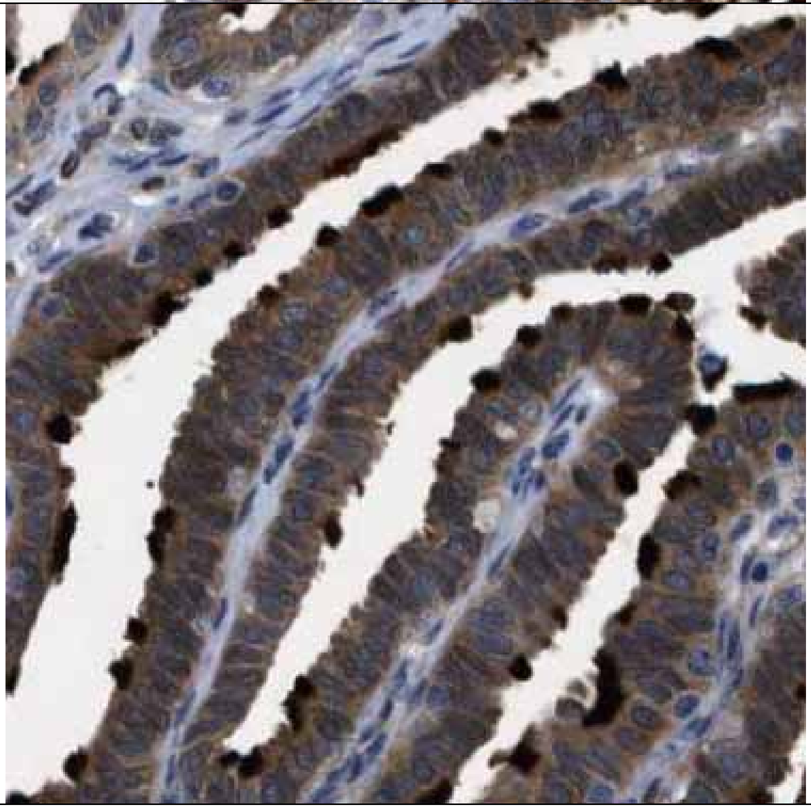	Staining is specific to *cilia*

SPA17 (antibody HPA037568)<br><br>Category 1	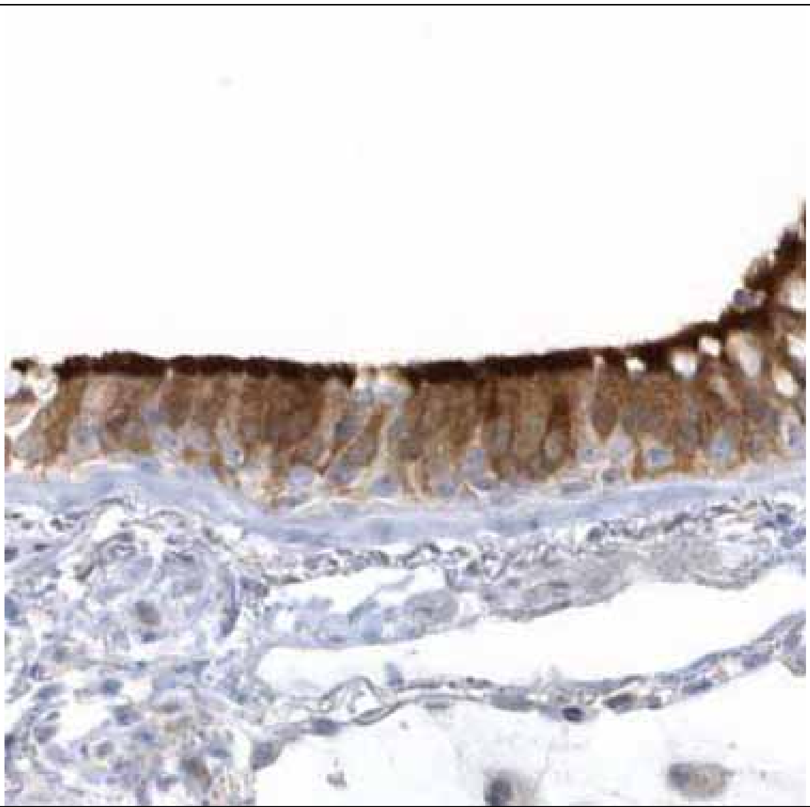	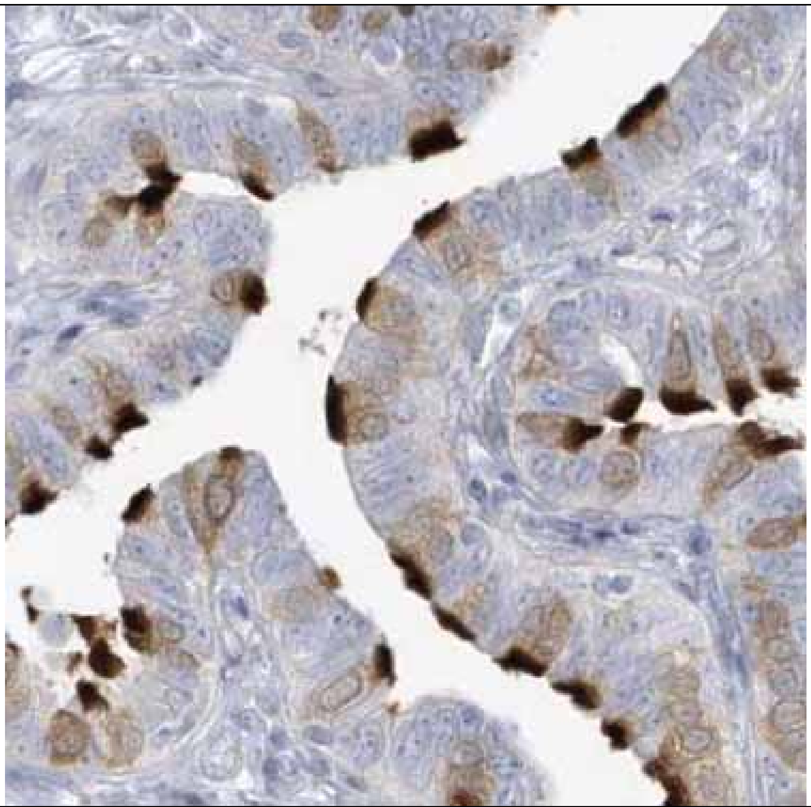	Staining is specific to *cilia*
SPAG6 (antibody HPA038440)<br><br>Category 1	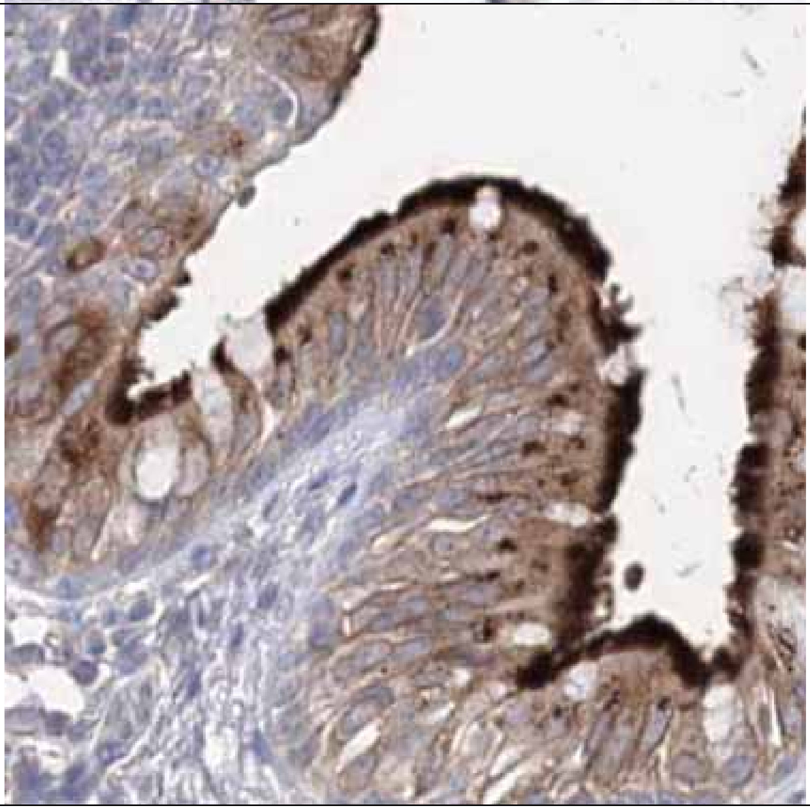	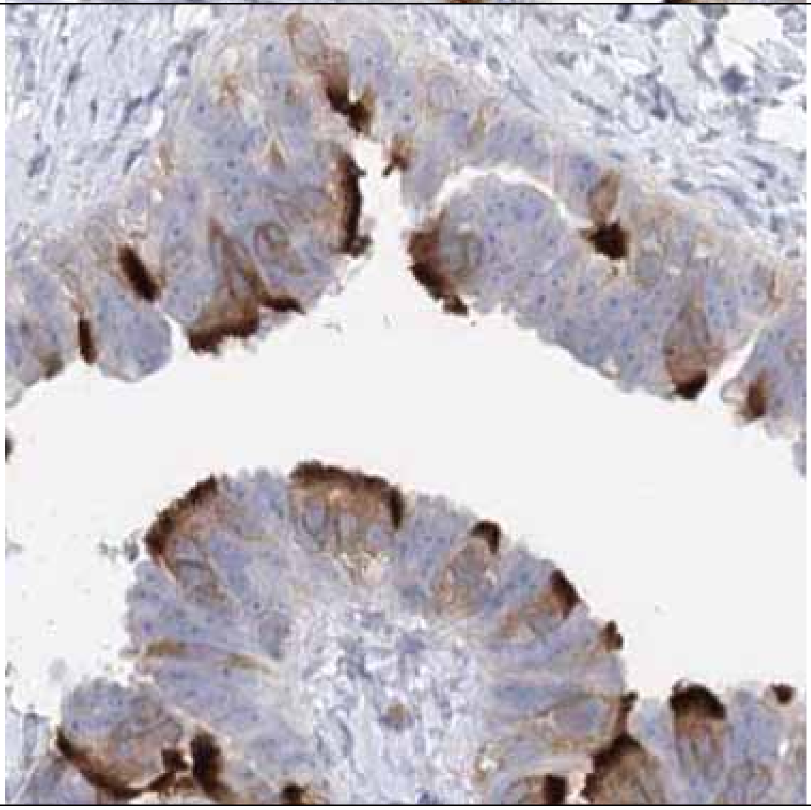	Staining is specific to *cilia*

SPATA17 (antibody HPA030099)<br><br>Category 1	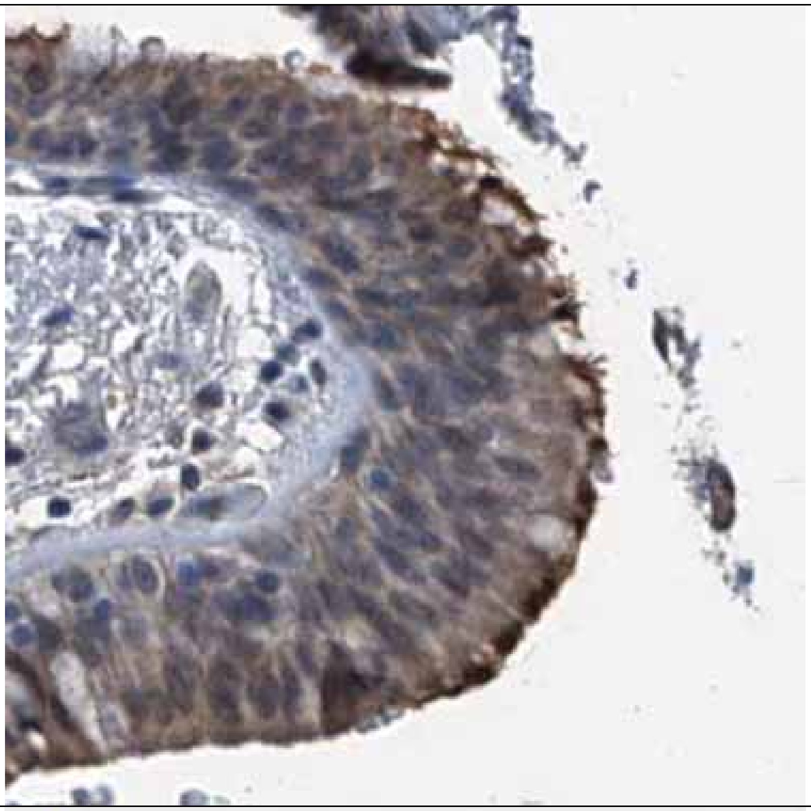	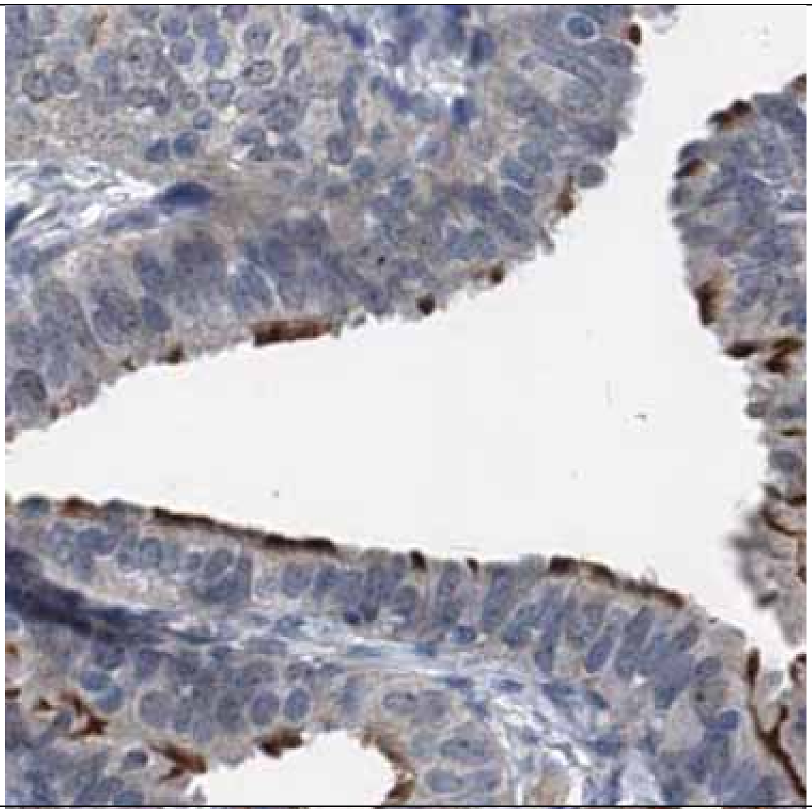	Staining is specific to *cilia*
SPEF2 (antibody HPA039606)<br><br>Category 1	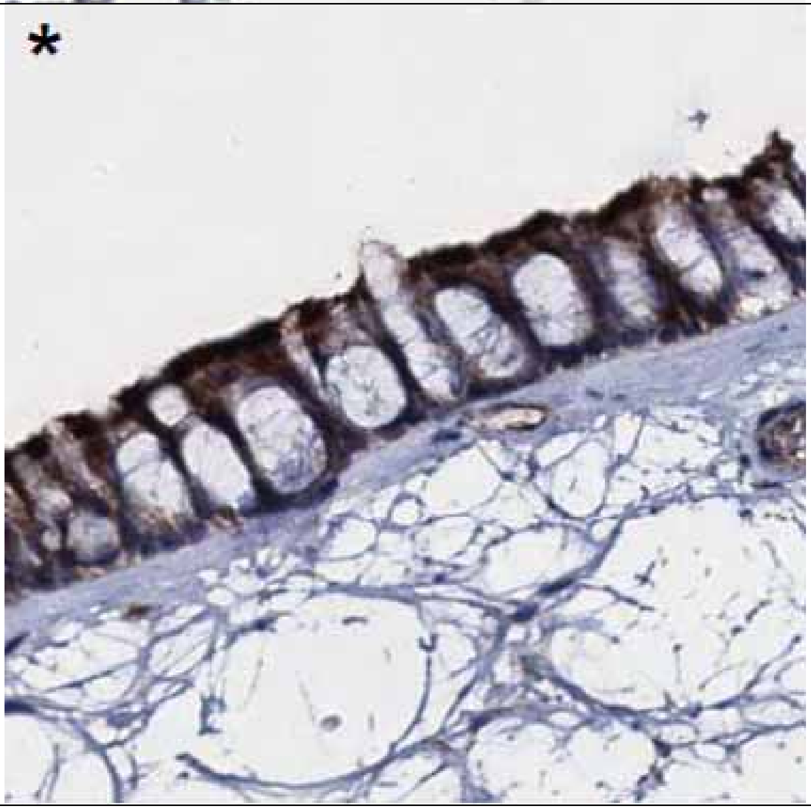	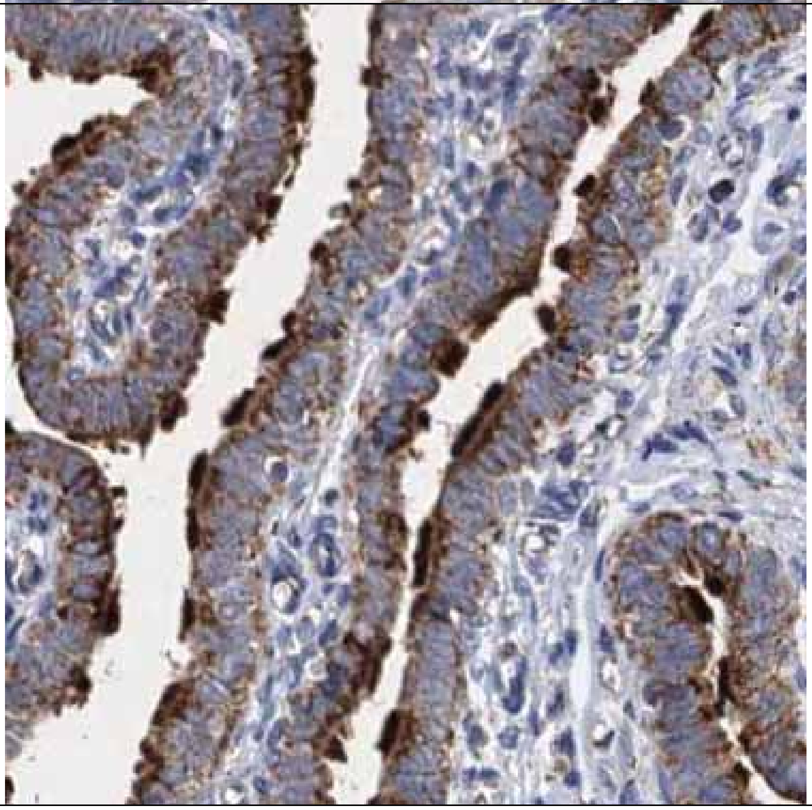	Staining is specific to *cilia*

SRGAP3 (antibody HPA036959)<br><br>Category 1	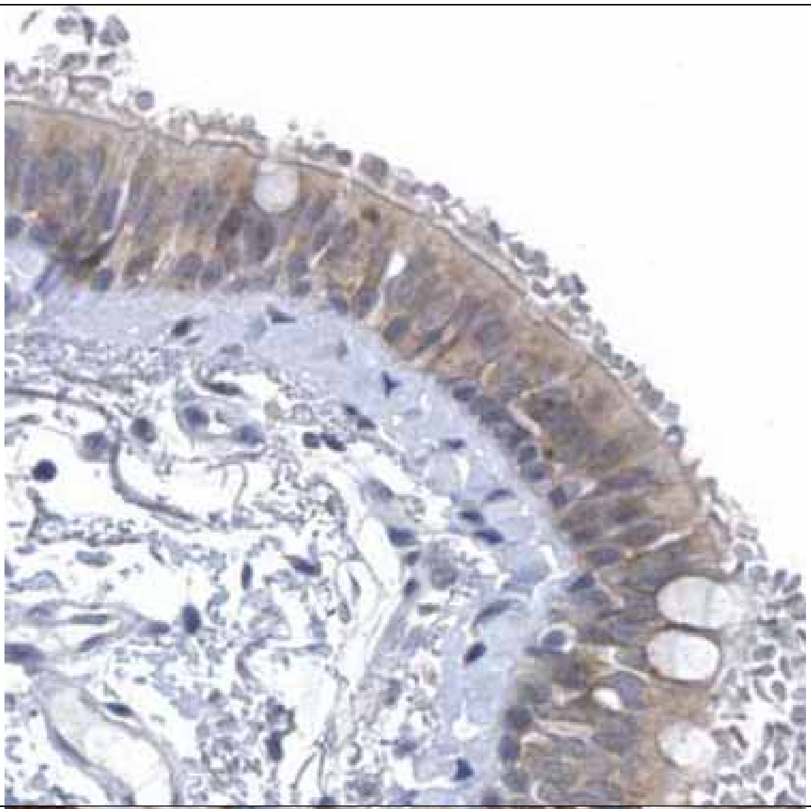	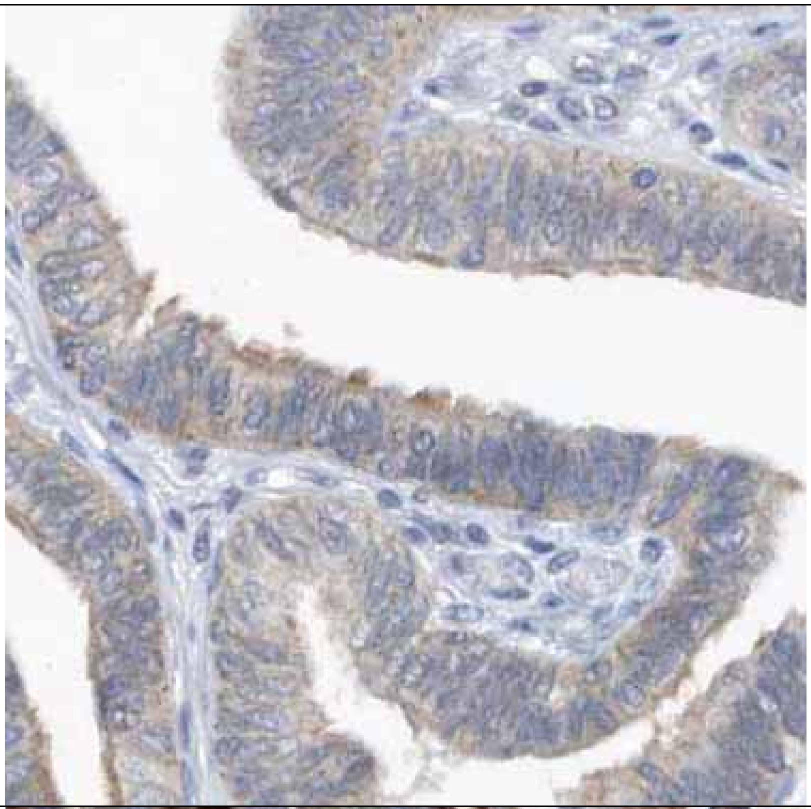	Staining is specific to *cilia*
STOML3 (antibody CAB014880)<br><br>Category 1	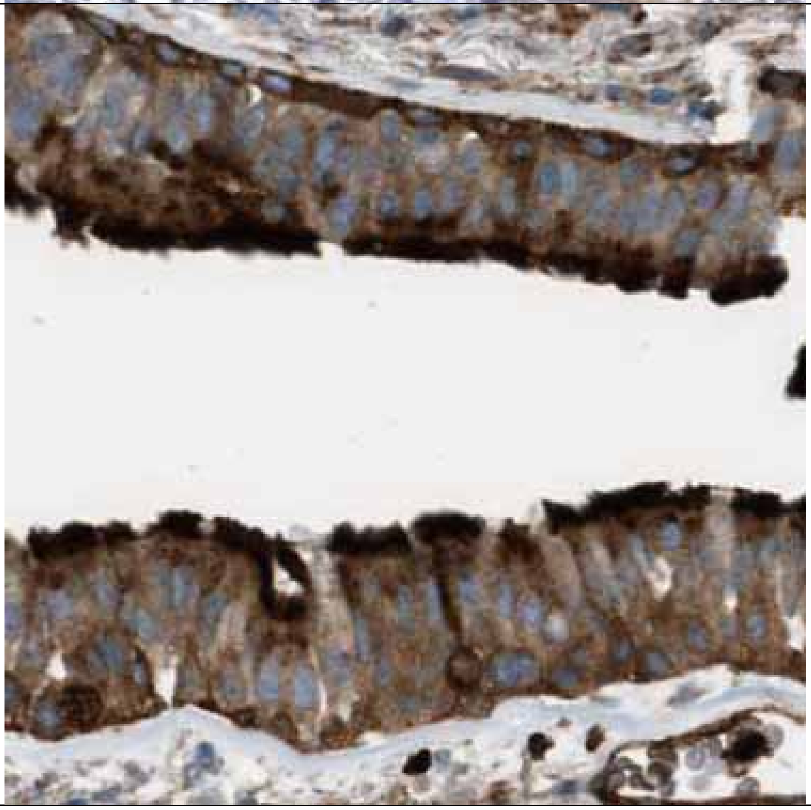	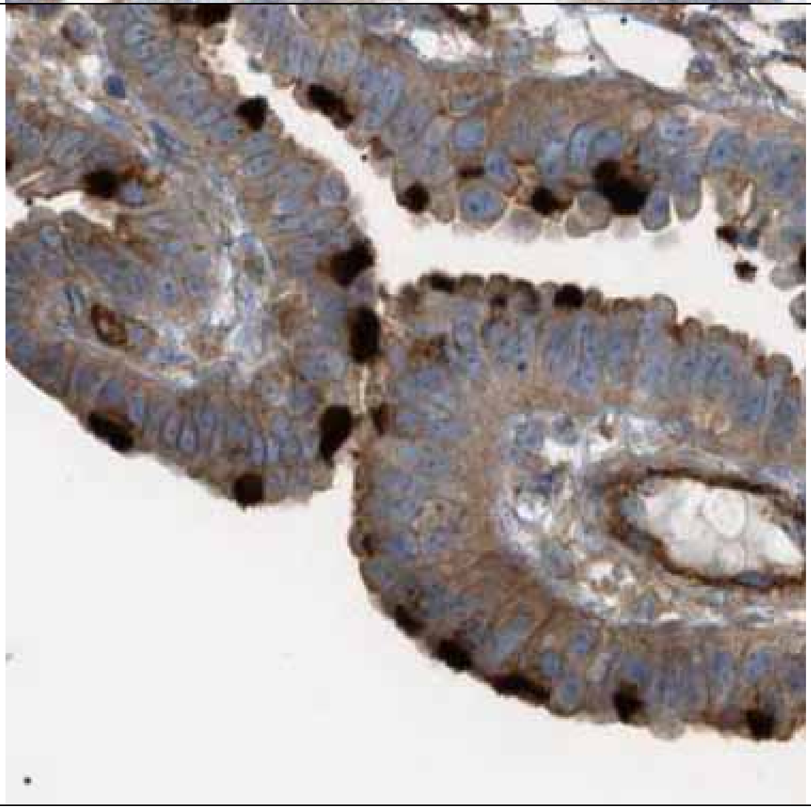	Staining is specific to *cilia*

TCTEX1D1 (antibody HPA028420)<br><br>Category 1	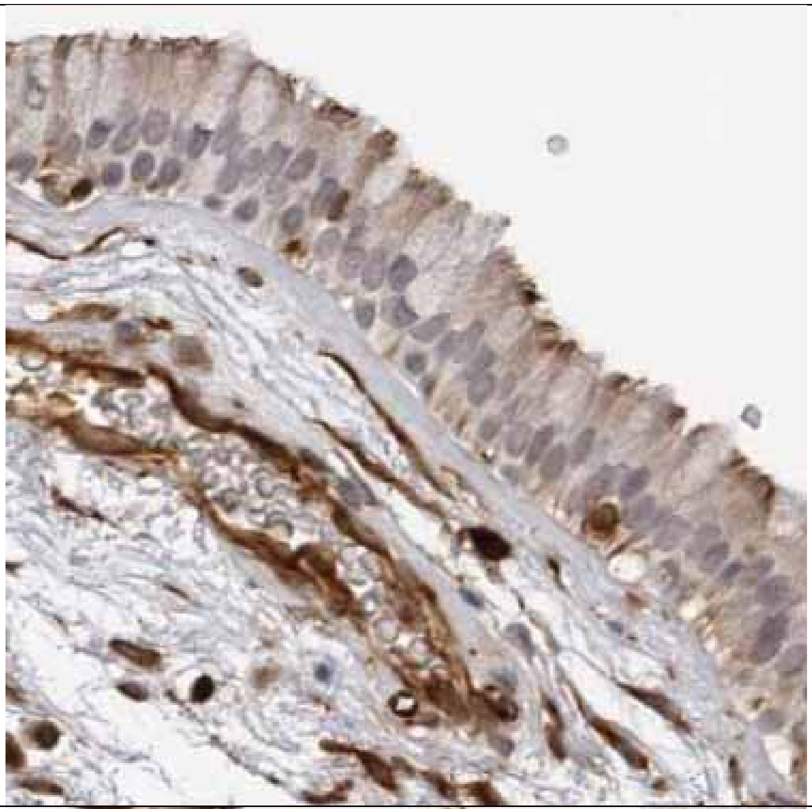	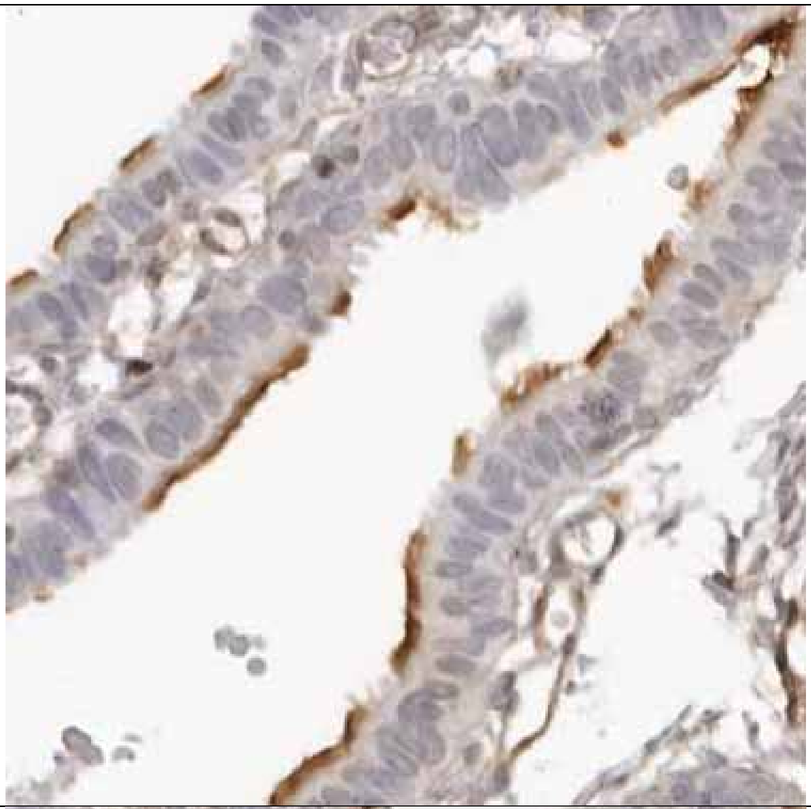	Staining is specific to *cilia*
TEKT2 (antibody HPA027461)<br><br>Category 1	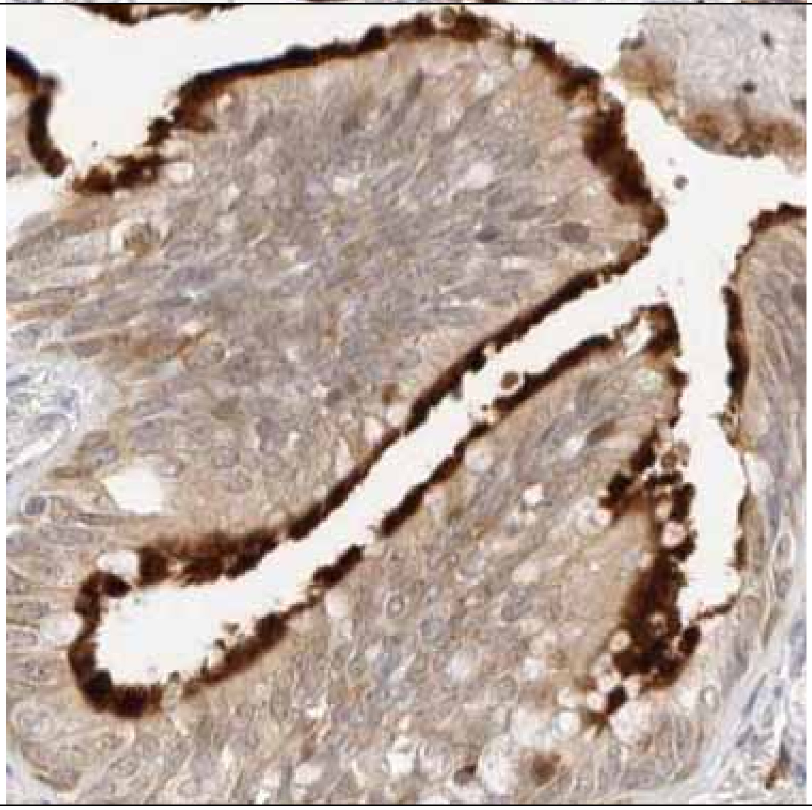	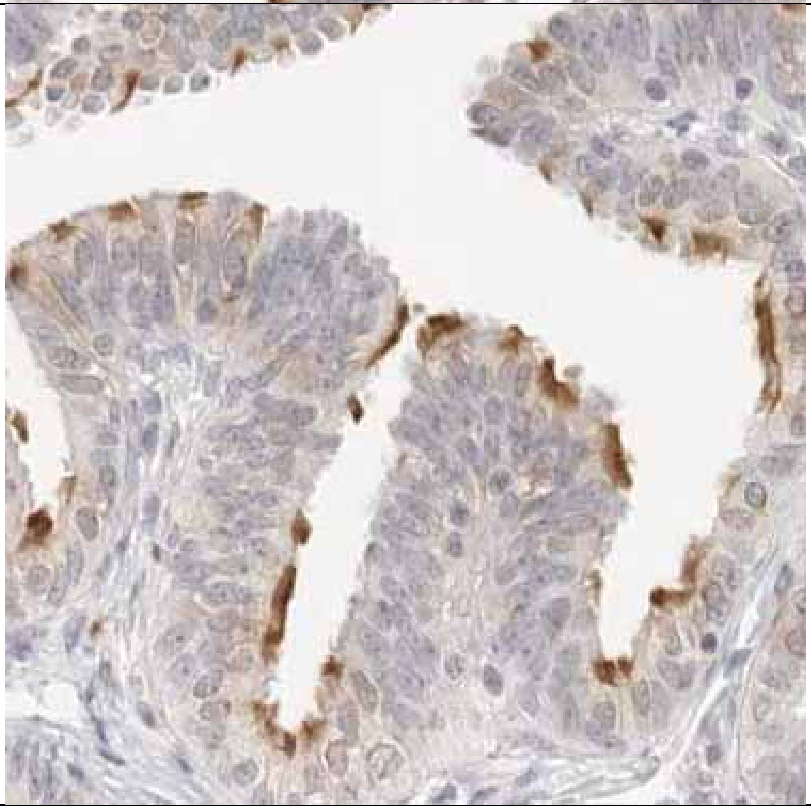	Staining is specific to *cilia*

TEX9 (antibody HPA039415)<br><br>Category 1	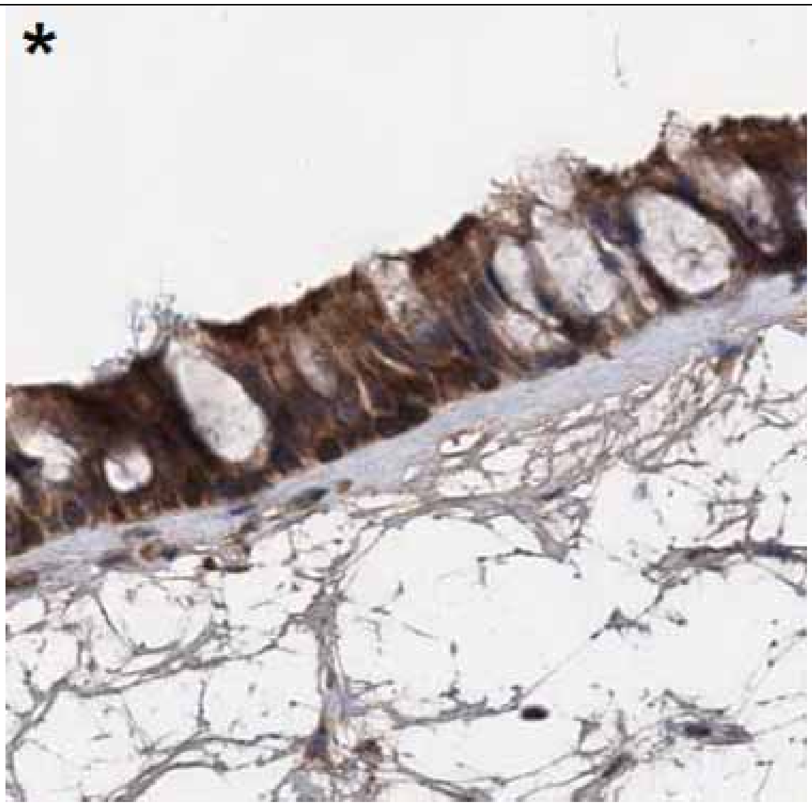	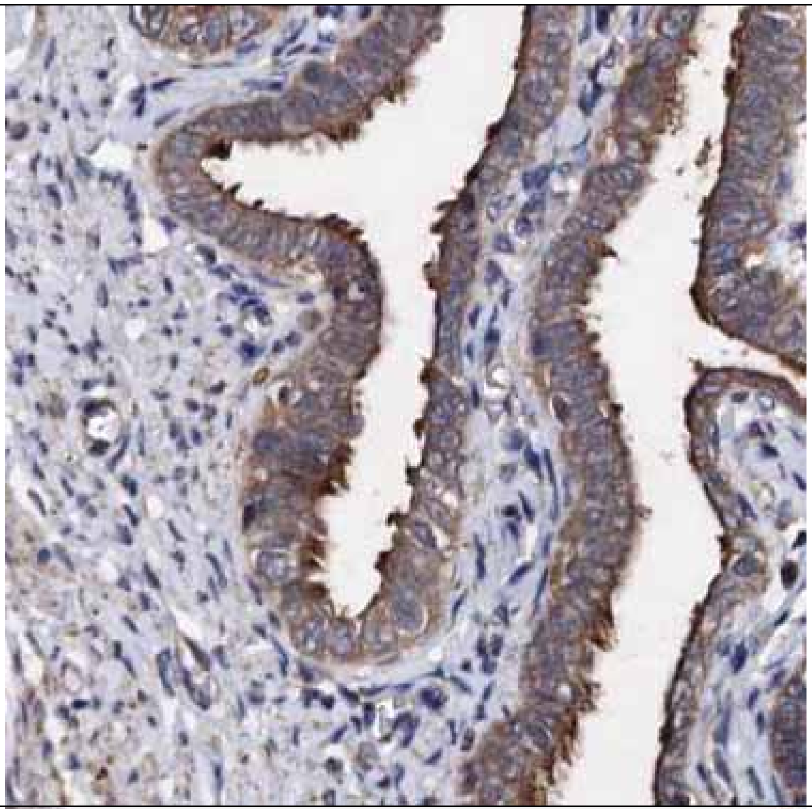	Staining is specific to *cilia*
TTC18 (antibody HPA037581)<br><br>Category 1	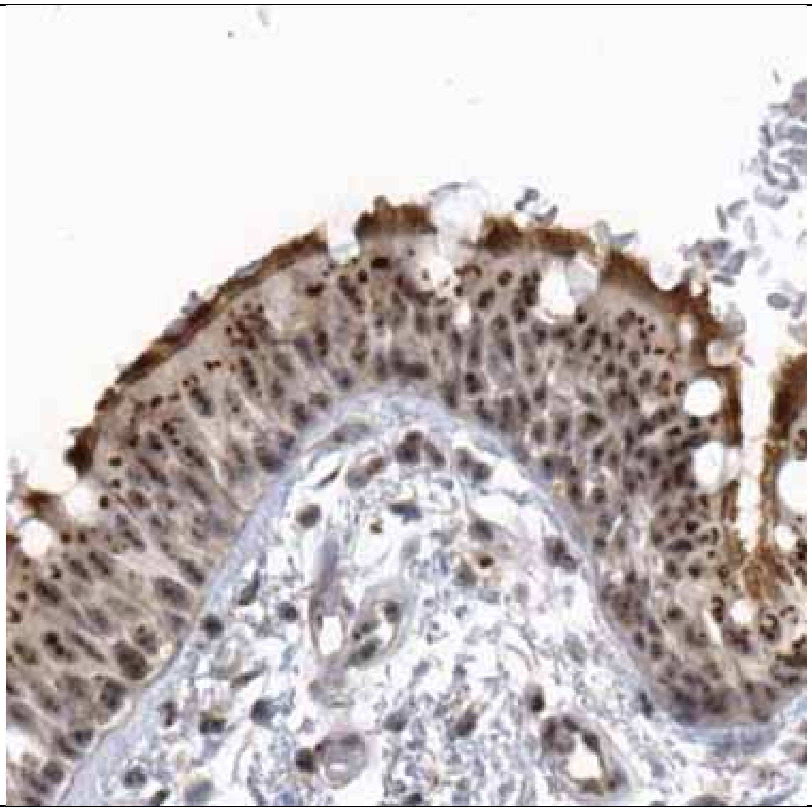	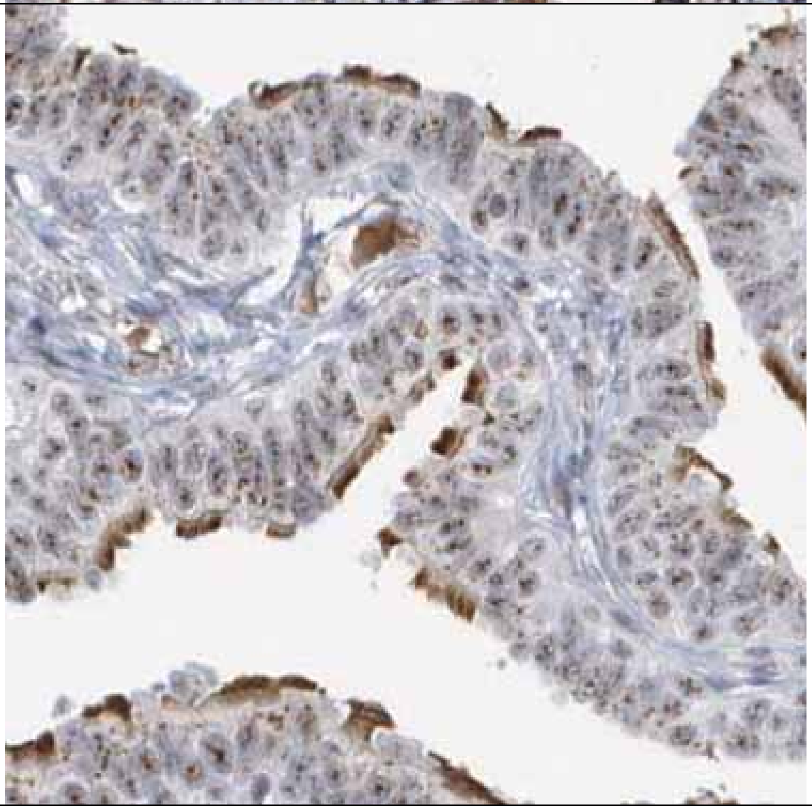	Staining is specific to *cilia*

TTC25 (antibody HPA023908)<br><br>Category 1	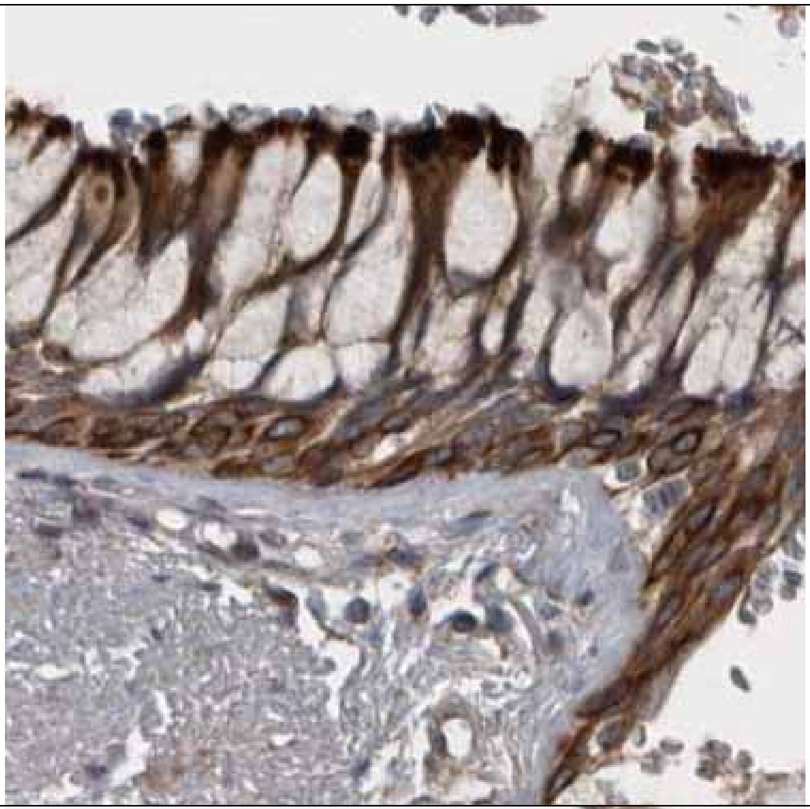	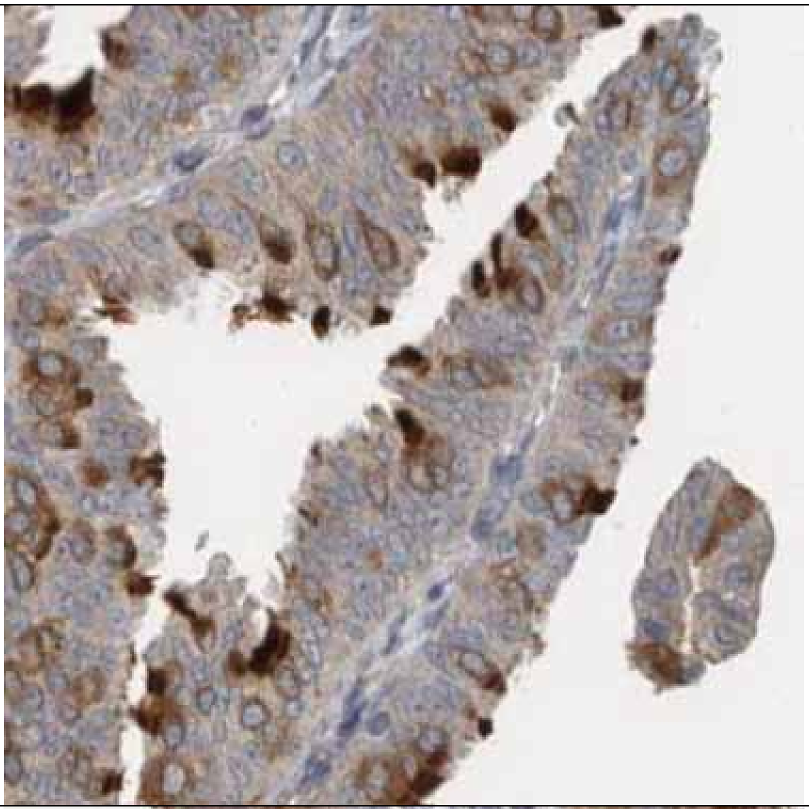	Staining is specific to *cilia*
TTC26 (antibody HPA036338)<br><br>Category 1	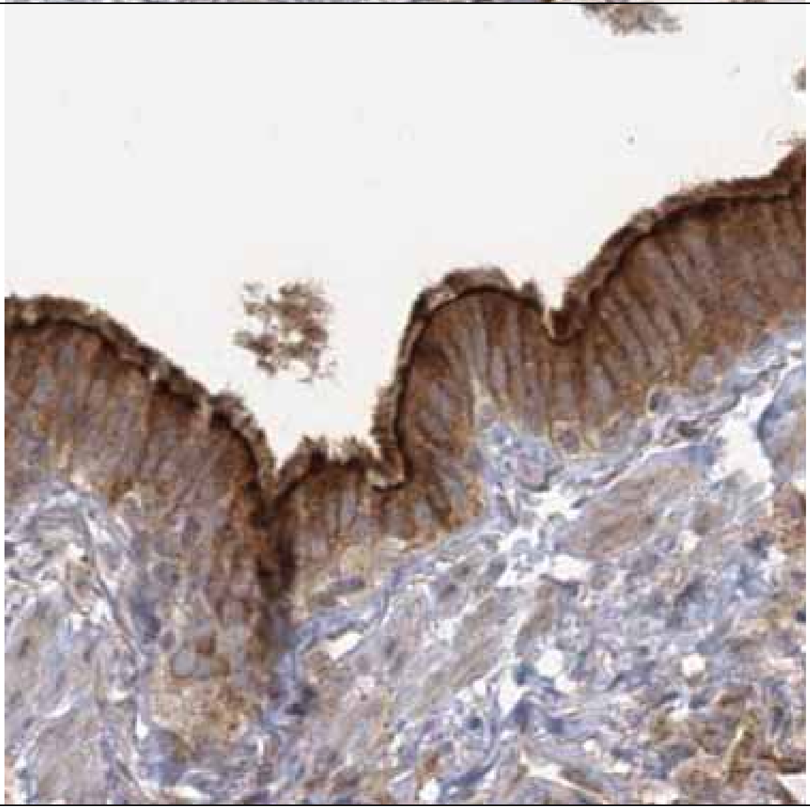	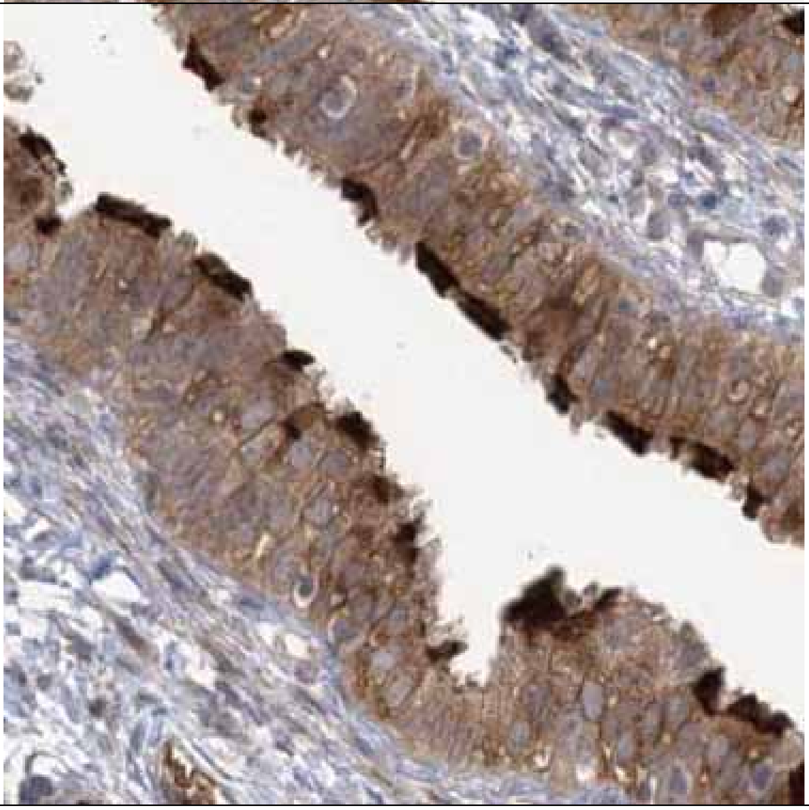	Staining is specific to *cilia*

TTC29 (antibody HPA037006)<br><br>Category 1	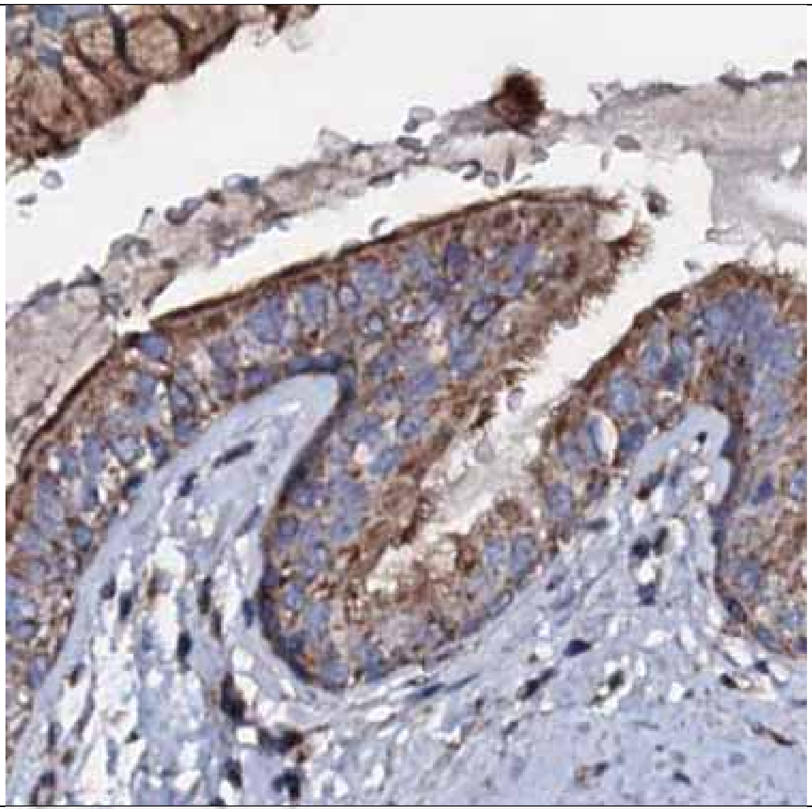	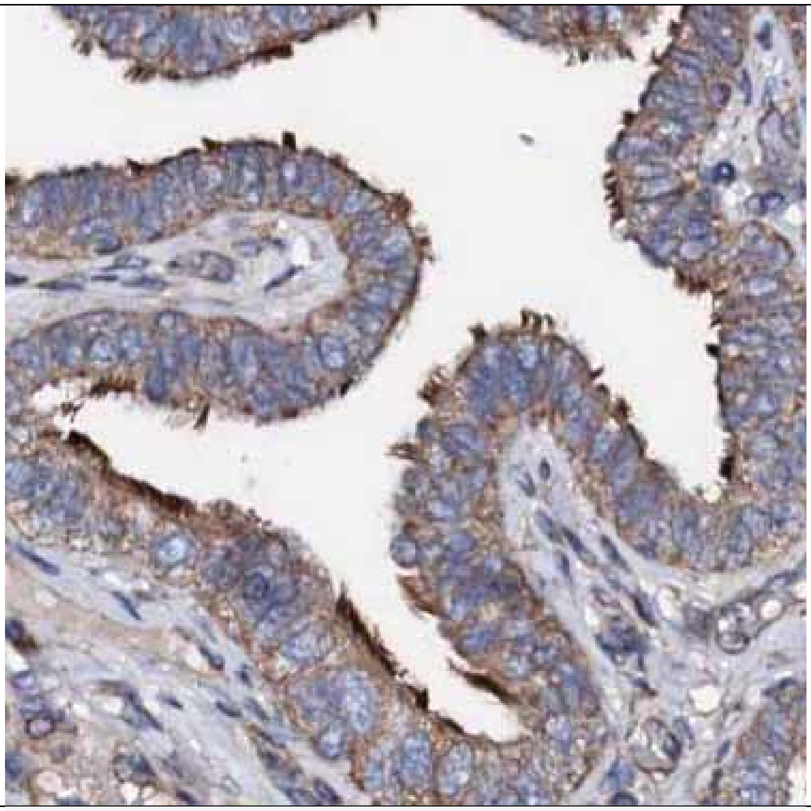	Staining is specific to *cilia*
WDR16 (antibody HPA023247)<br><br>Category 1	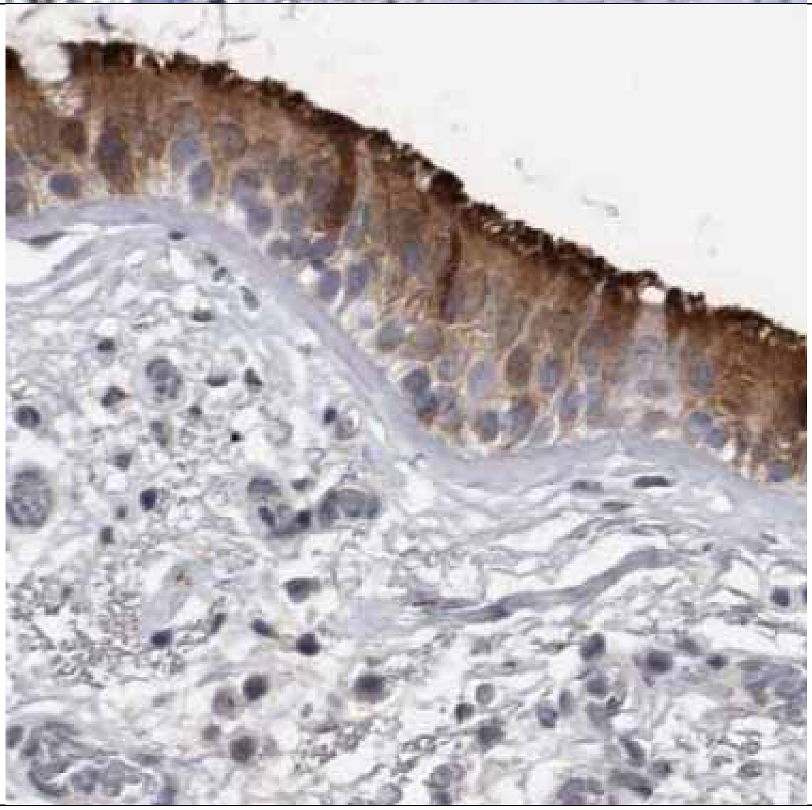	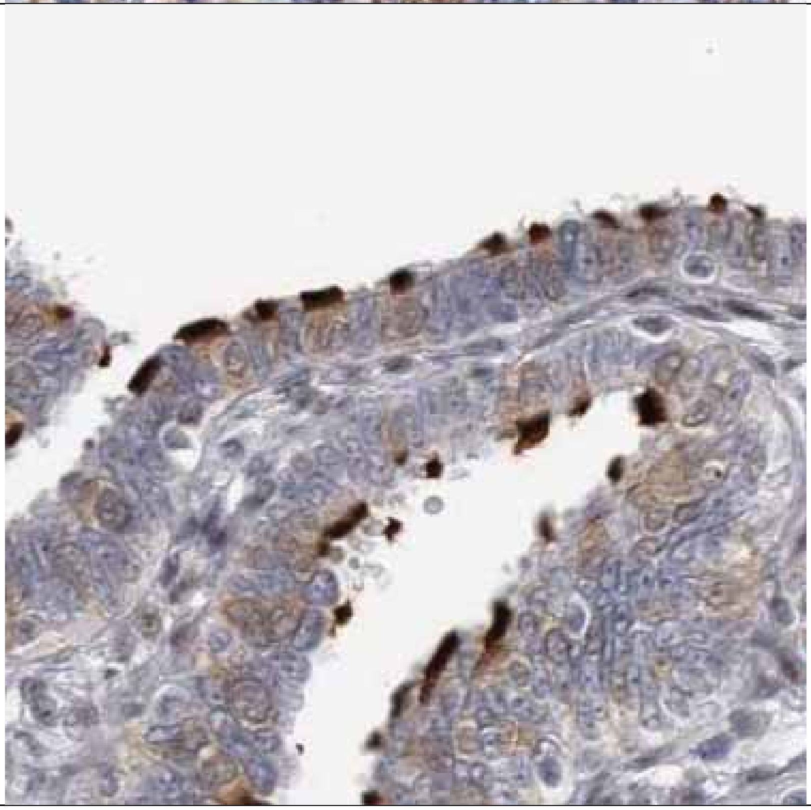	Staining is specific to *cilia*

YSK4 (antibody HPA011388)<br><br>Category 1	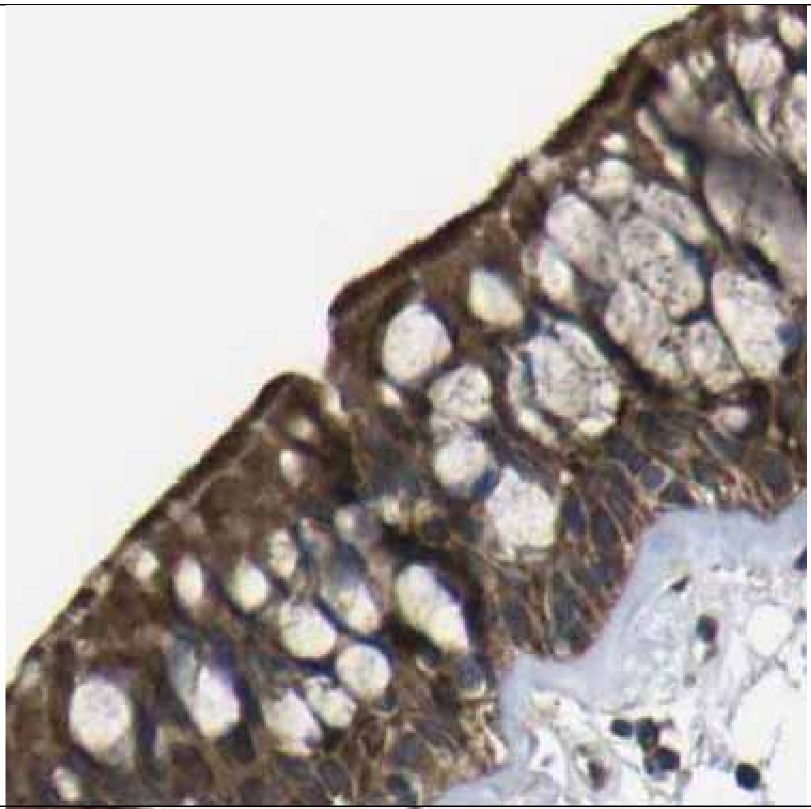	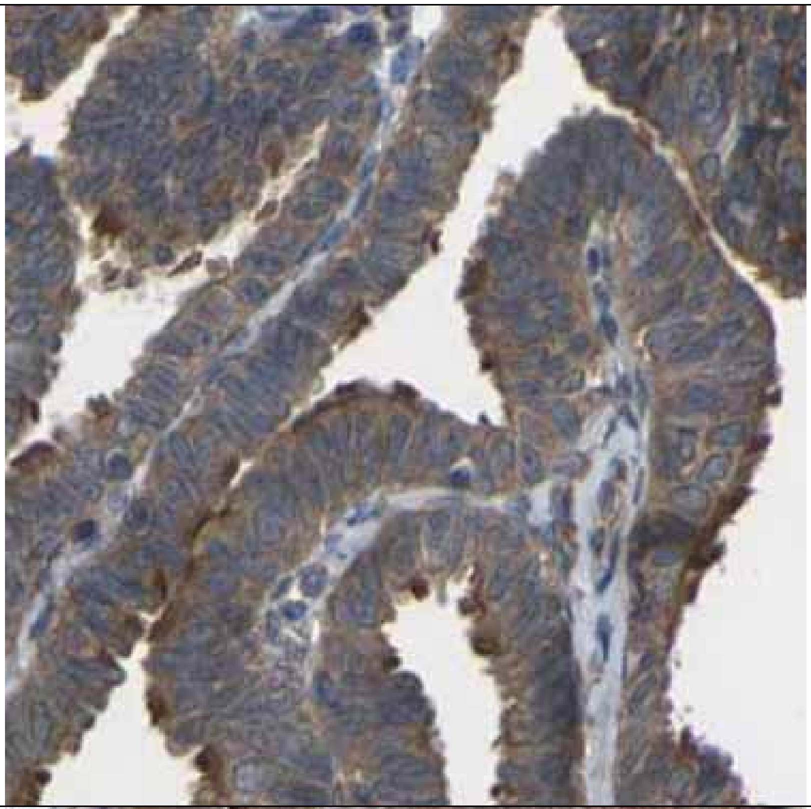	Staining is specific to *cilia*
ZBBX (antibody HPA036327)<br><br>Category 1	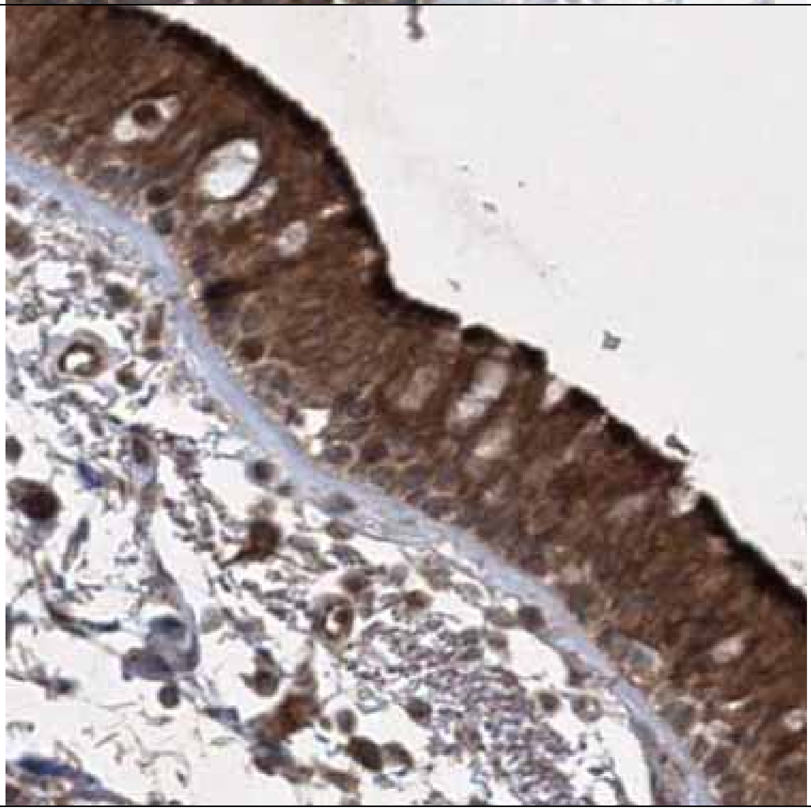	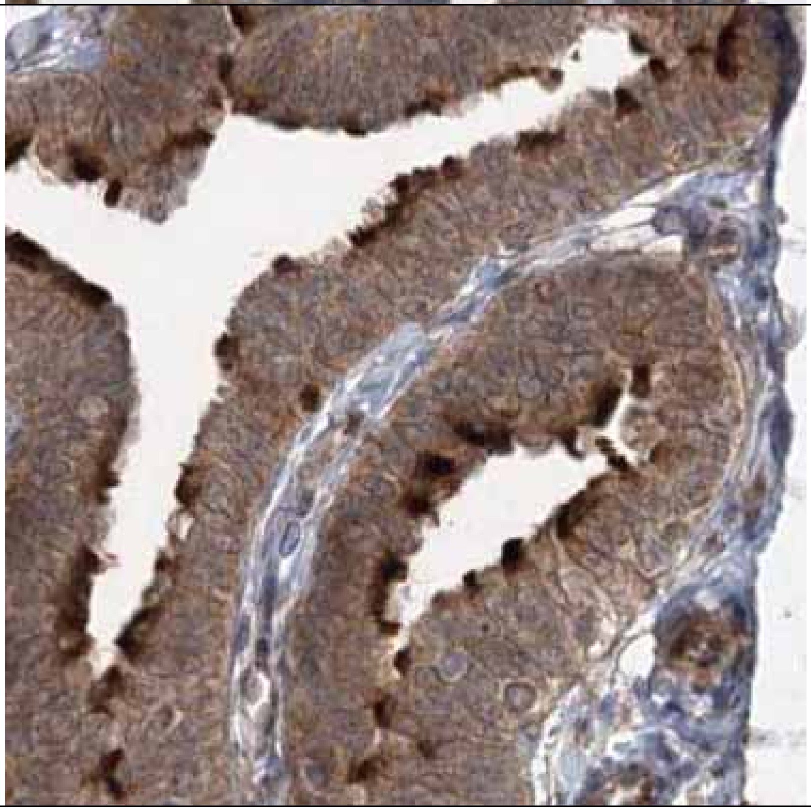	Staining is specific to *cilia*

AK1 (antibody HPA006456)<br><br>Category 1	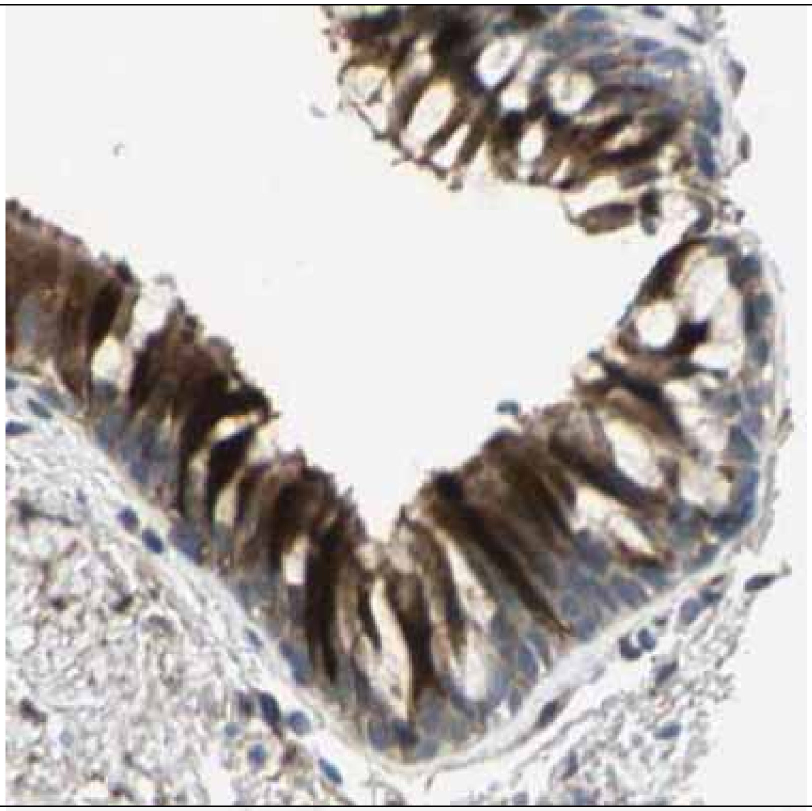	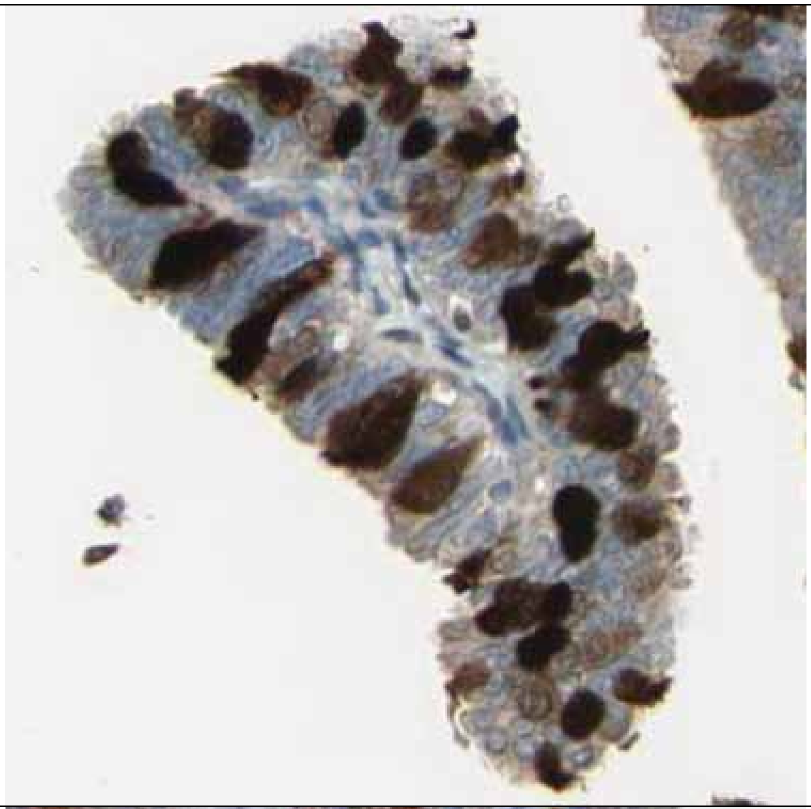	Staining is specific to *ciliated cells* (cilia and cytoplasm)
B9D1 (antibody HPA022957)<br><br>Category 1	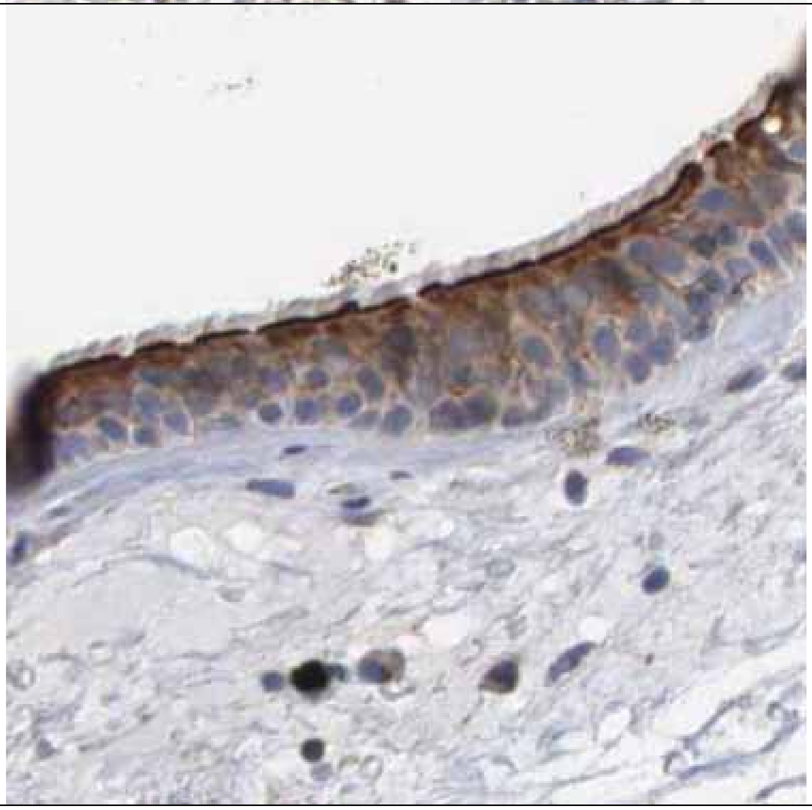	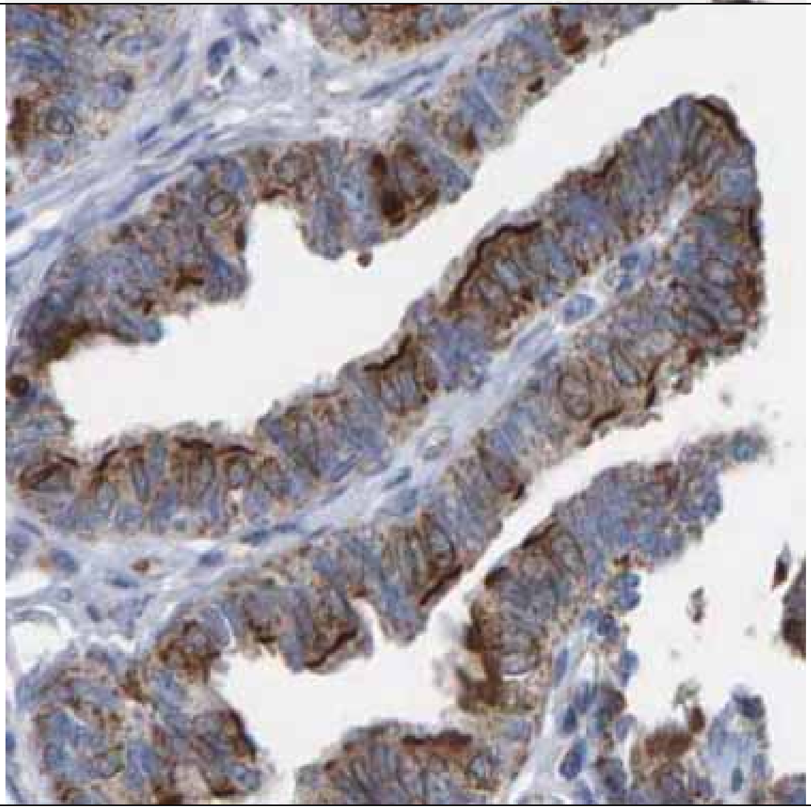	Staining is specific to *ciliated cells* (apical region of cytoplasm)

C1orf102 (antibody HPA028436)<br><br>Category 1	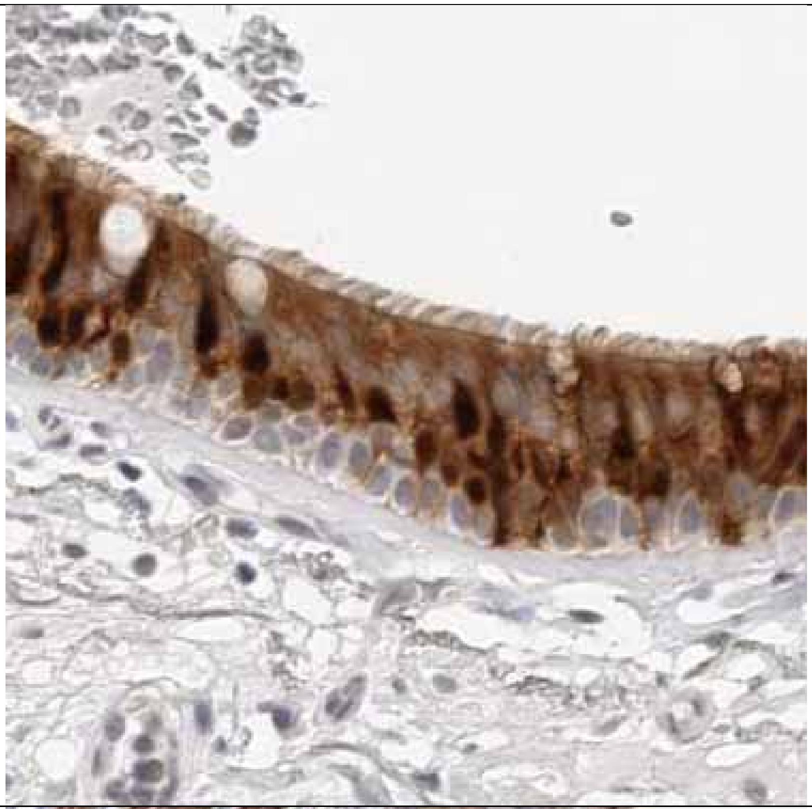	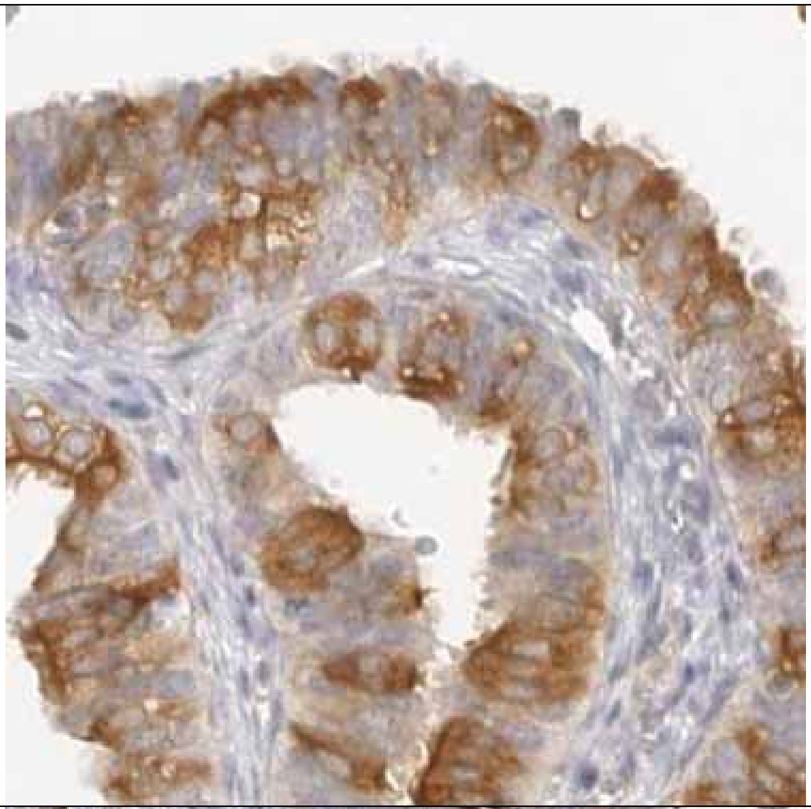	Staining is specific to *ciliated cells* (cytoplasm)
C11orf60 (antibody HPA037909)<br><br>Category 1	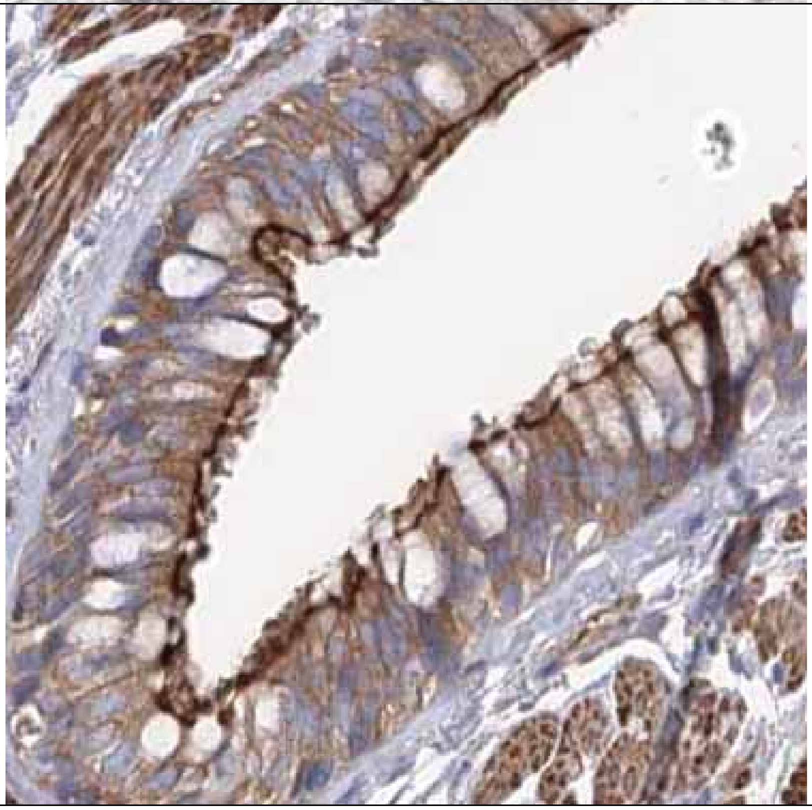	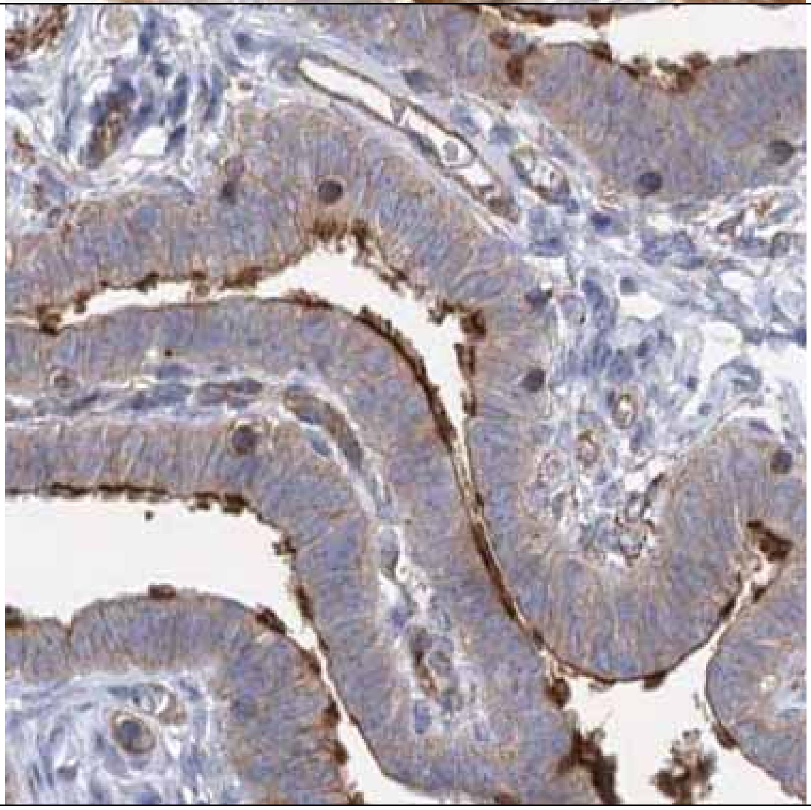	Staining is specific to *ciliated cells* (apical region of cytoplasm)

<table>
<tr><td>C13orf30
(antibody
HPA040118)

Category 1</td><td>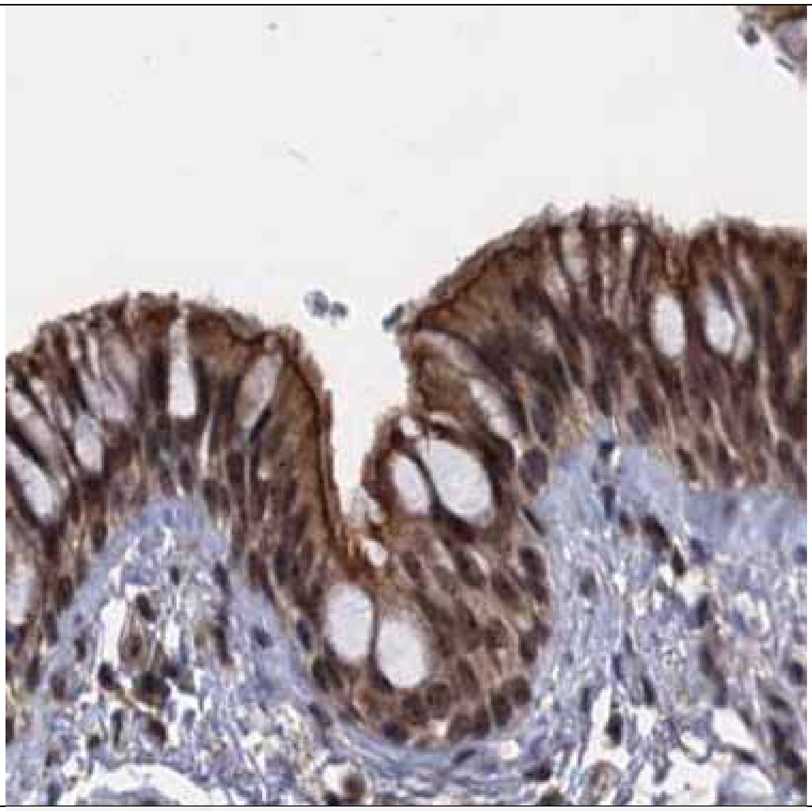</td><td>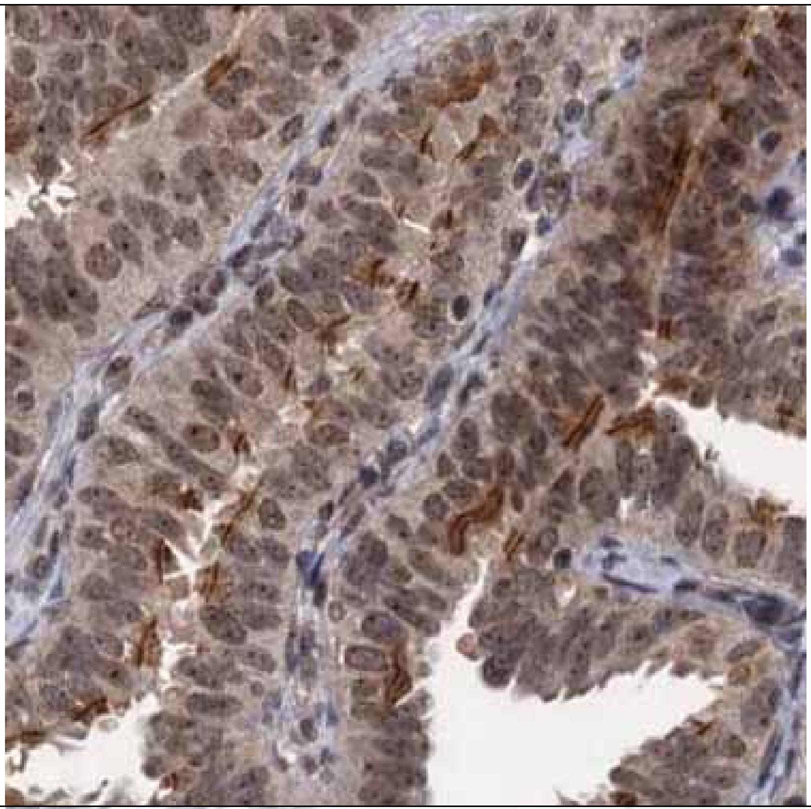</td><td>Staining is specific to ciliated cells (apical region of cytoplasm)</td></tr>
<tr><td>C6orf118
(antibody
HPA029787)

Category 1</td><td>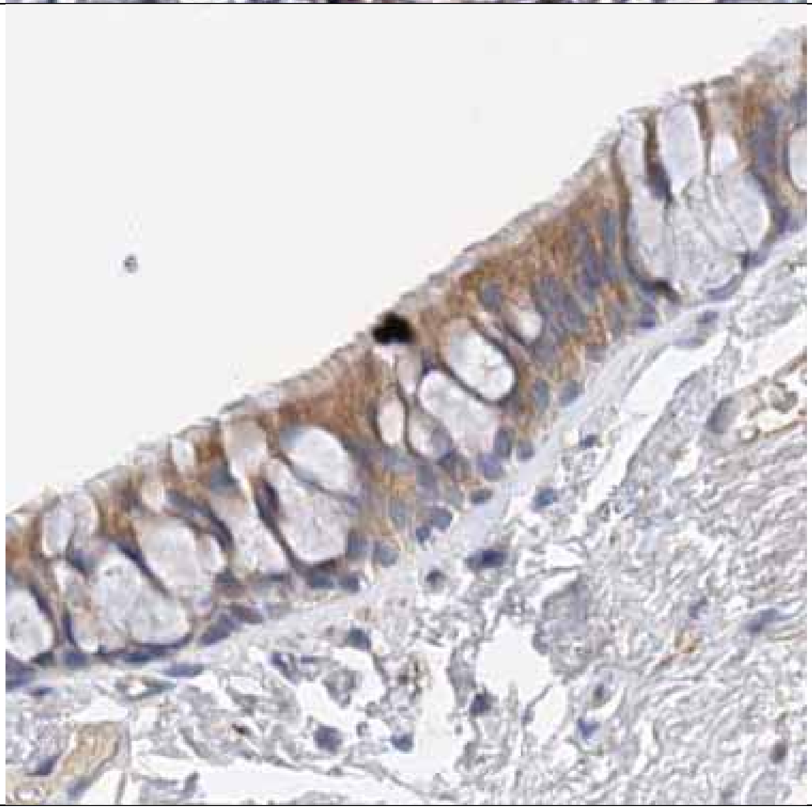</td><td>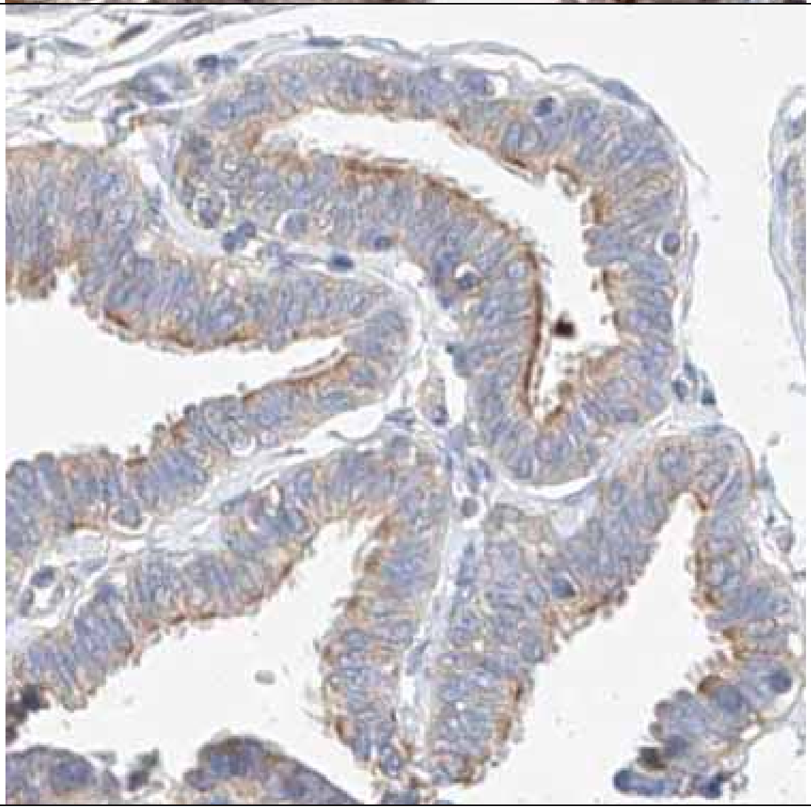</td><td>Staining is specific to ciliated cells (uncertain subcellular localization)</td></tr>
</table>

C9orf135<br>(antibody<br>HPA021325)<br><br>Category 1	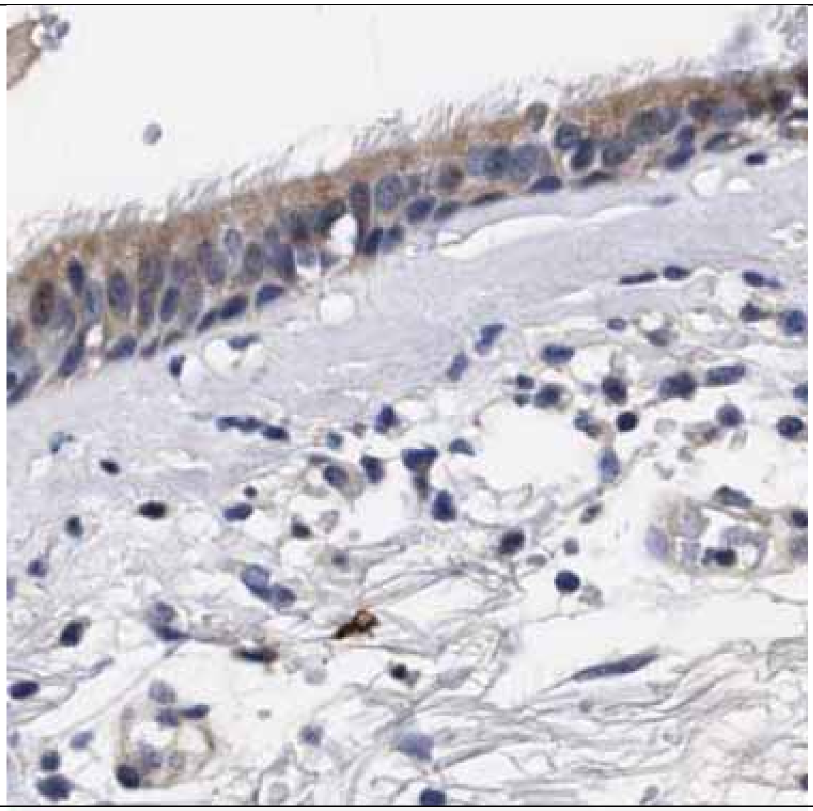	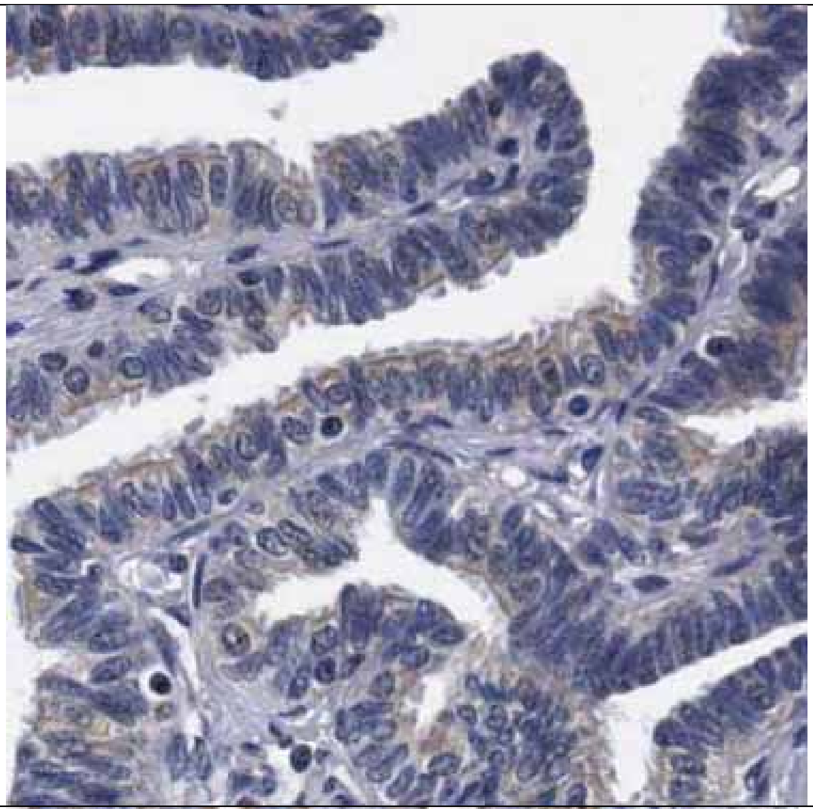	Staining is specific to *ciliated cells* (uncertain subcellular localization)
C2CD3<br>(antibody<br>HPA038552)<br><br>Category 1	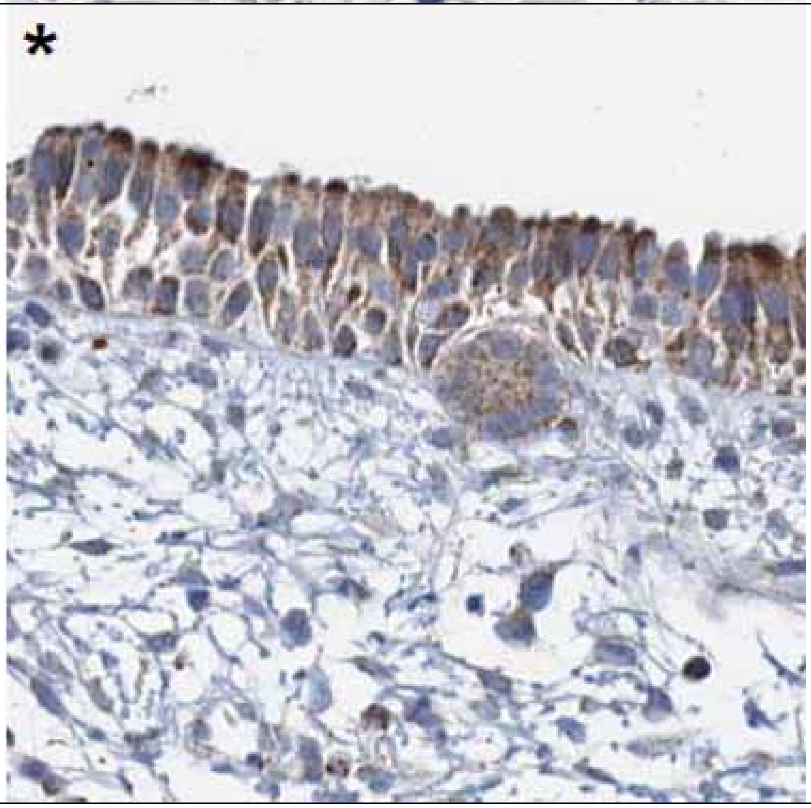	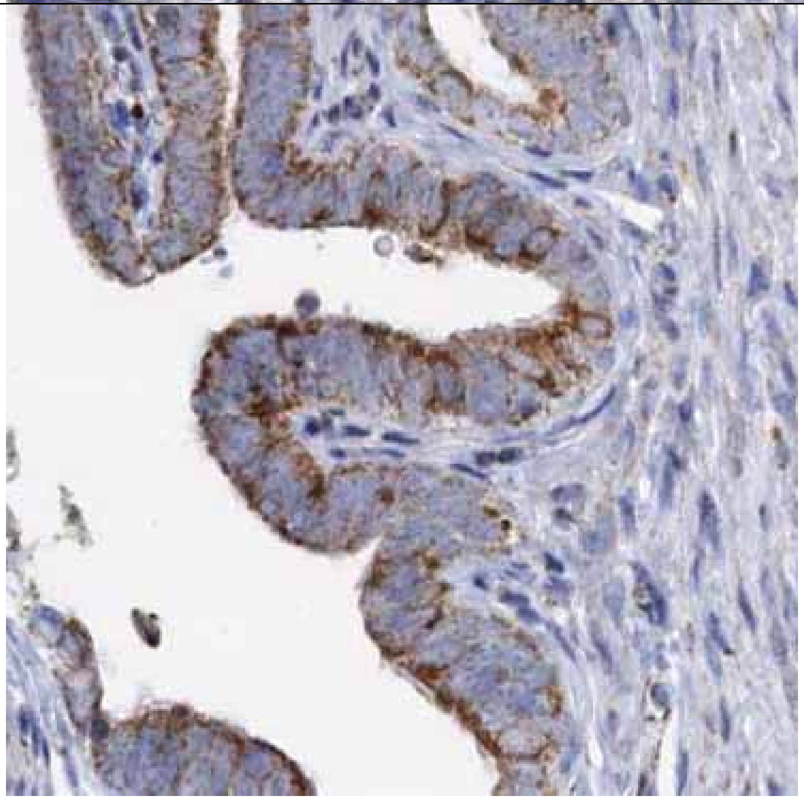	Staining is specific to *ciliated cells* (apical cytoplasm)

CCDC41<br>(antibody<br>HPA038161)<br><br>Category 1	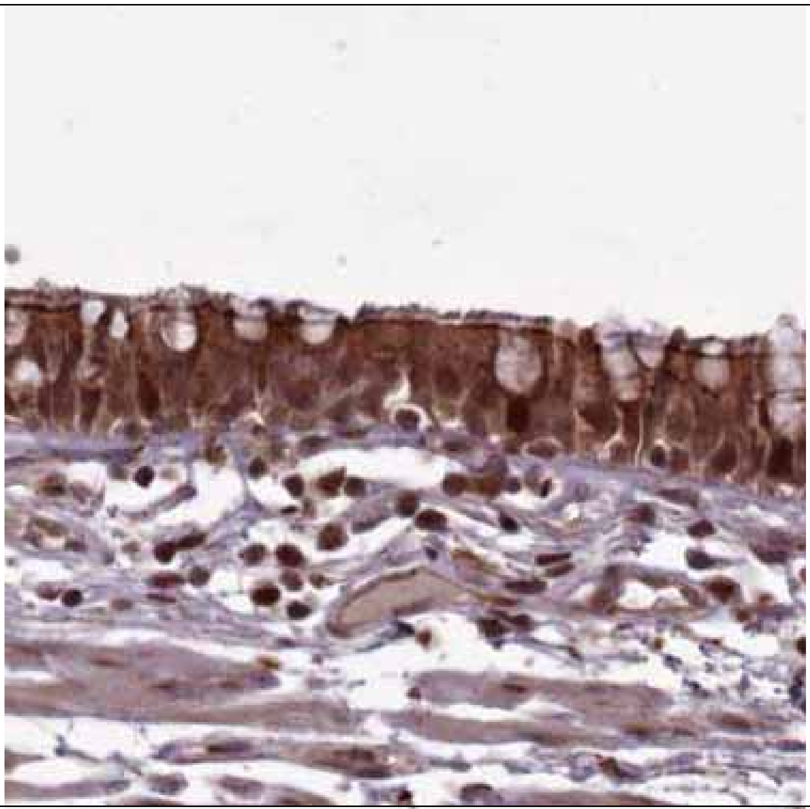	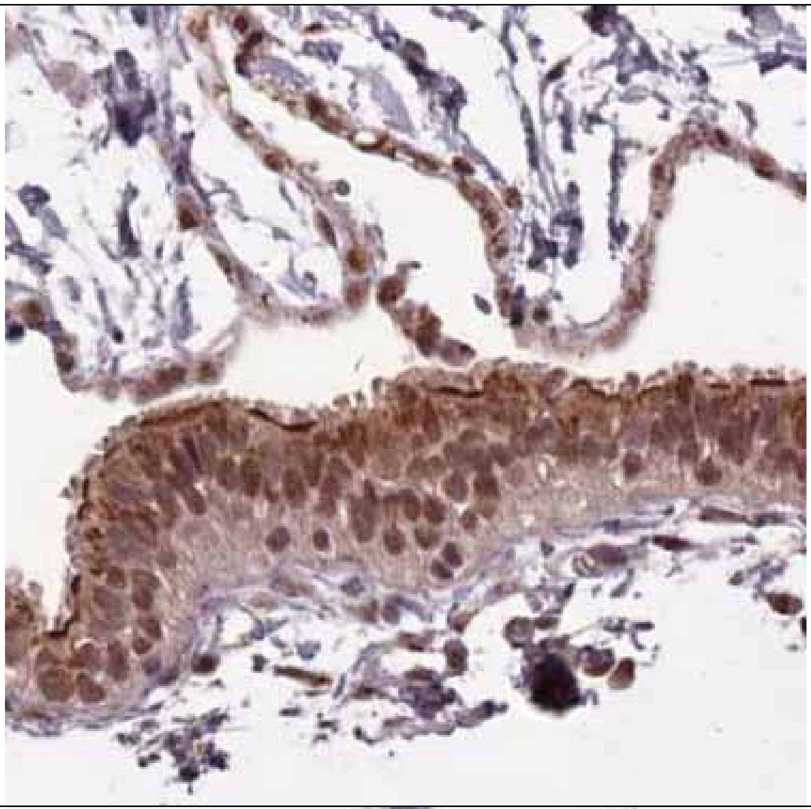	Staining is specific to *ciliated cells* (apical region of cytoplasm)
CDS1<br>(antibody<br>HPA036187)<br><br>Category 1	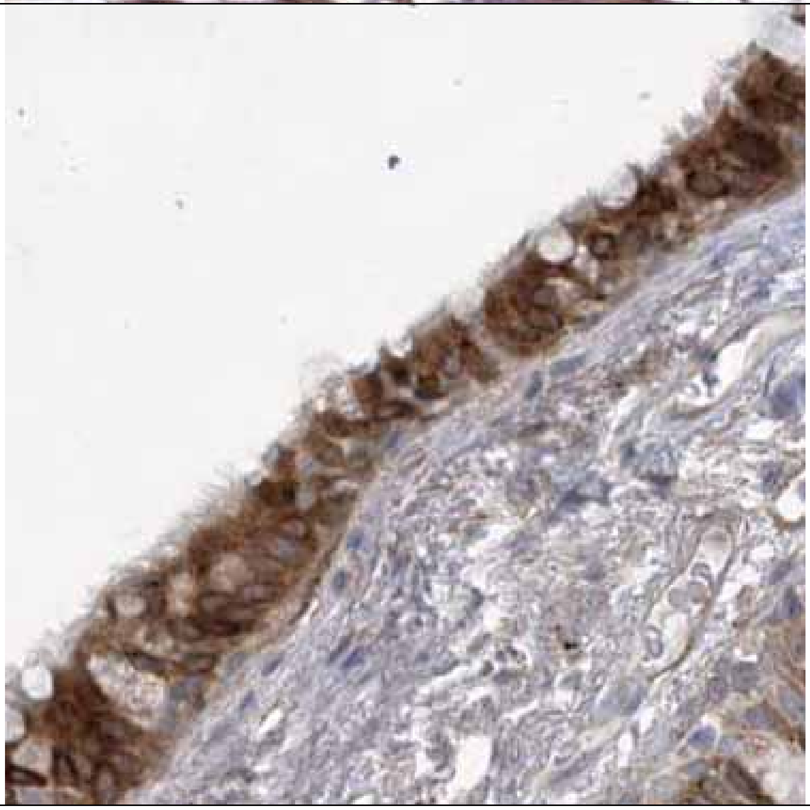	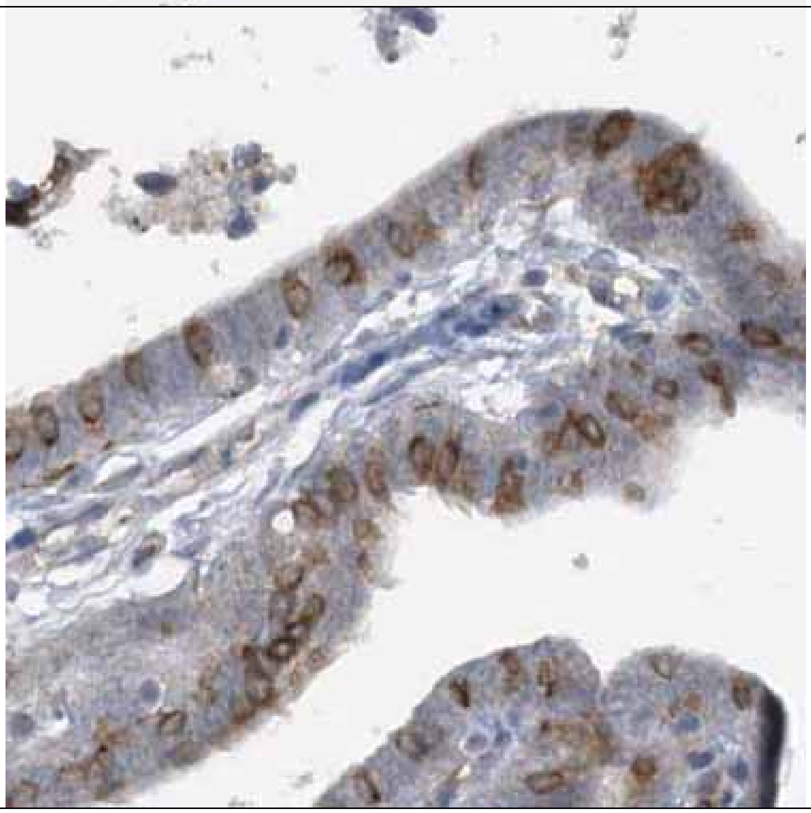	Staining is specific to *ciliated cells* (cytoplasm)

CEP97<br>(antibody<br>HPA002980)<br><br>Category 1	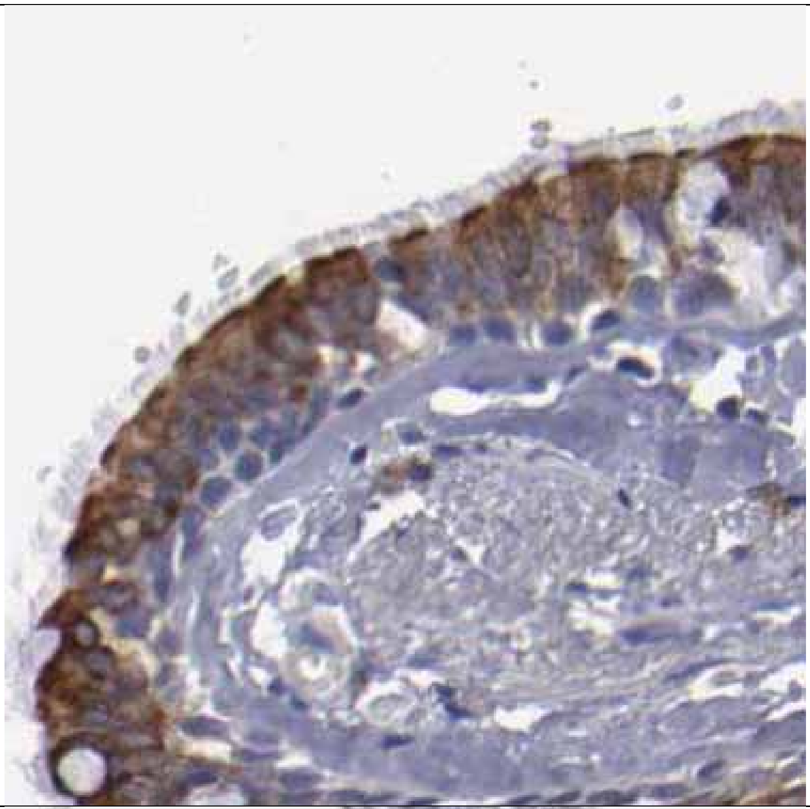	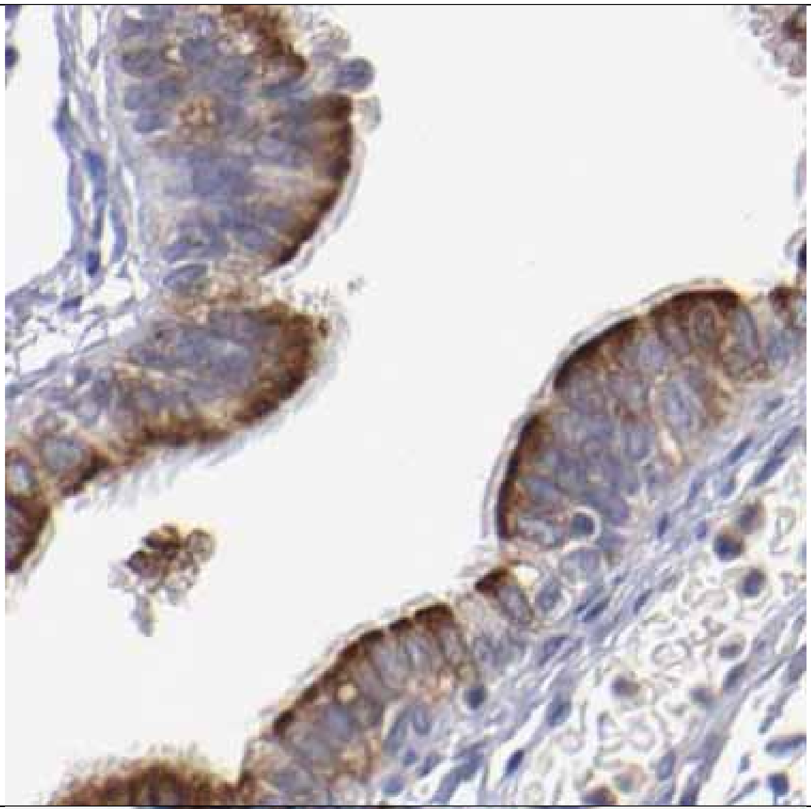	Staining is specific to *ciliated cells* (apical region of cytoplasm)
CLGN<br>(antibody<br>CAB020709)<br><br>Category 1	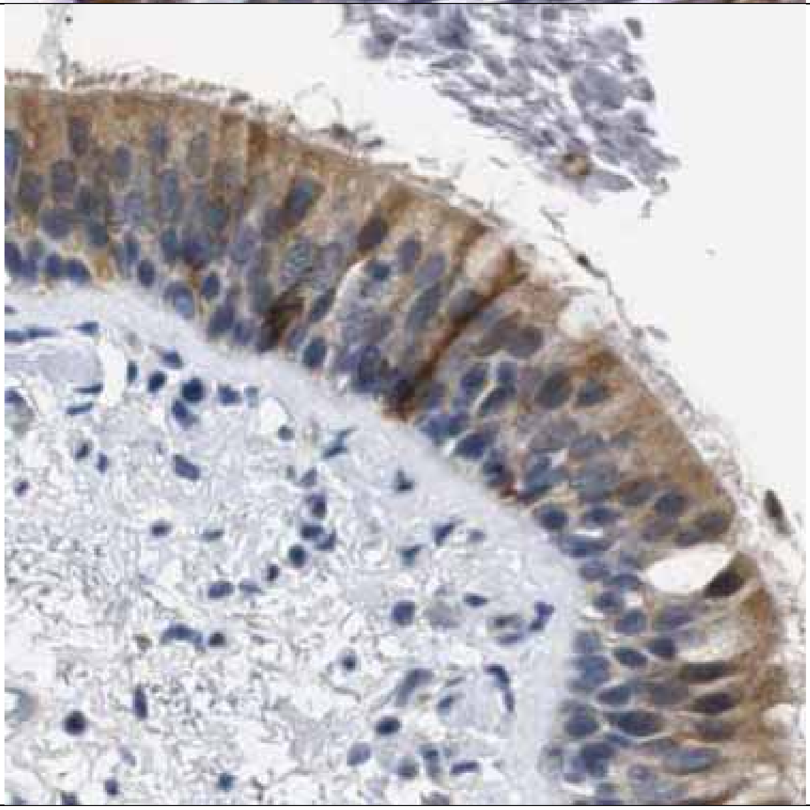	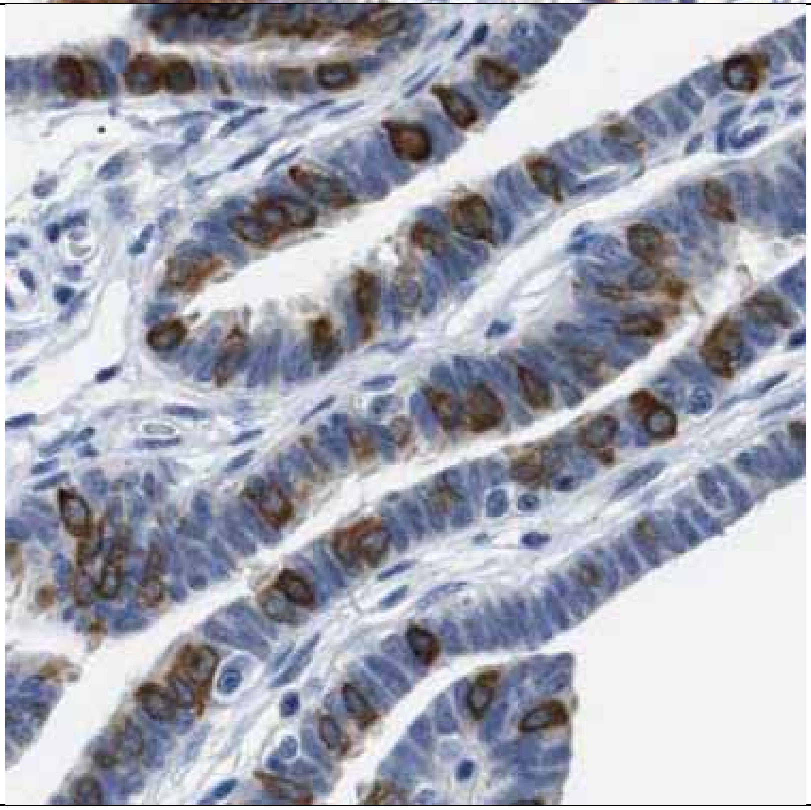	Staining is specific to *ciliated cells* (cytoplasm)

CLUAP1 (antibody HPA036976)<br><br>Category 1	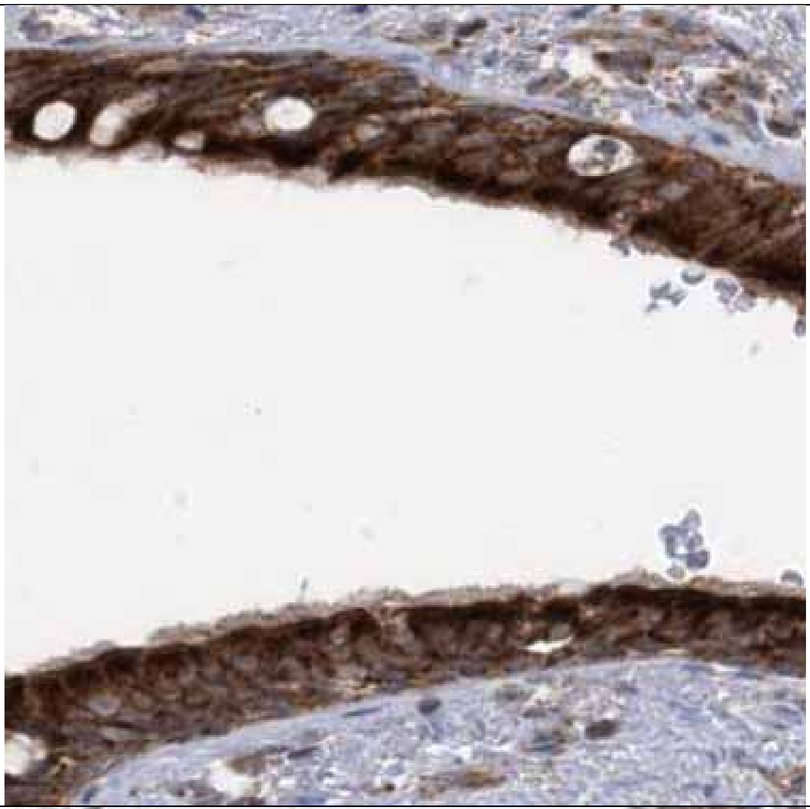	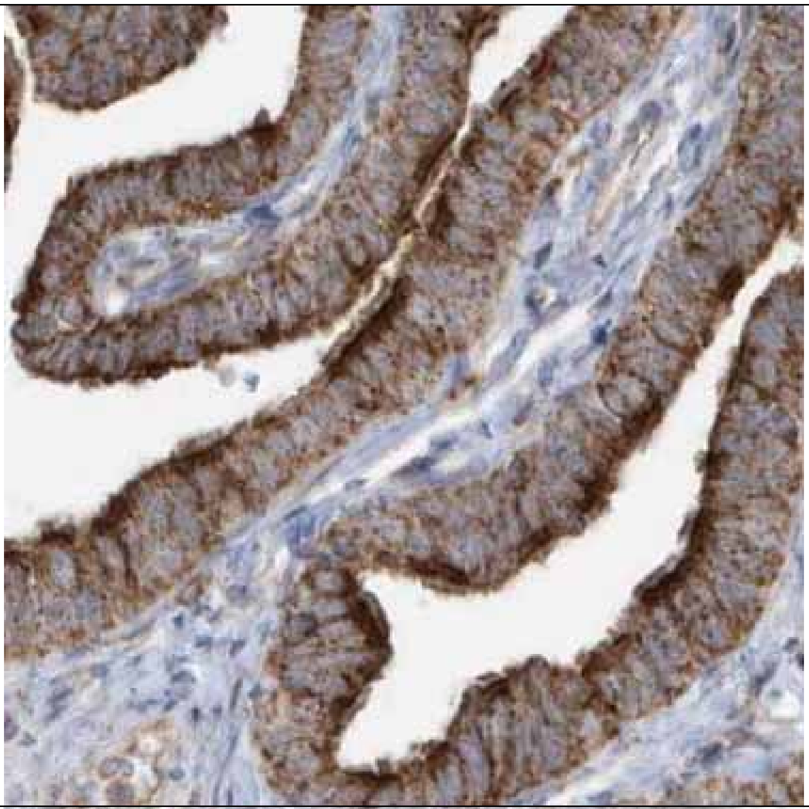	Staining is specific to *ciliated cells* (apical region of cytoplasm)
DPCD (antibody HPA036604)<br><br>Category 1	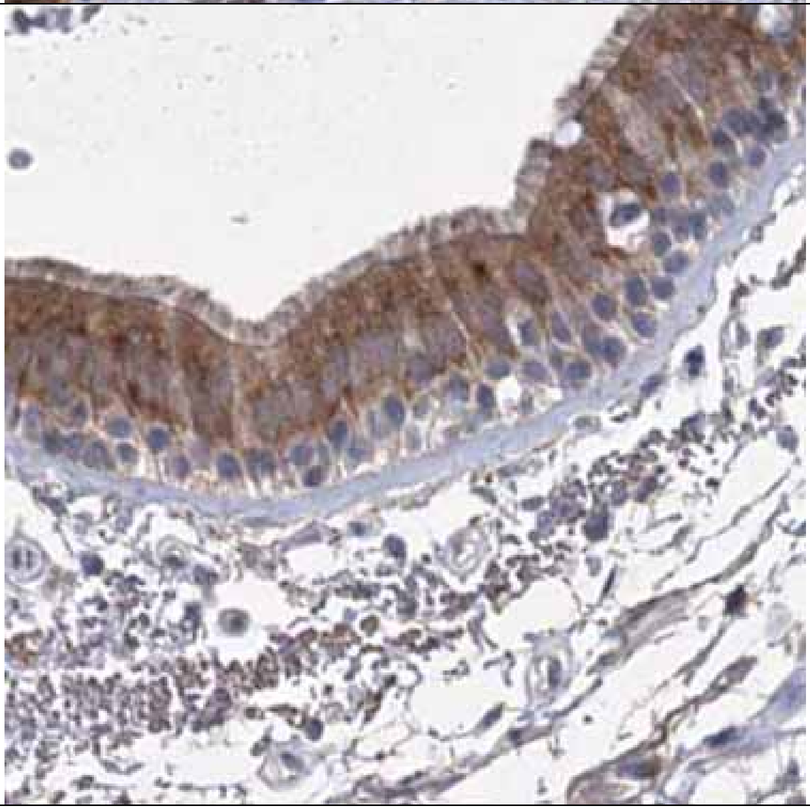	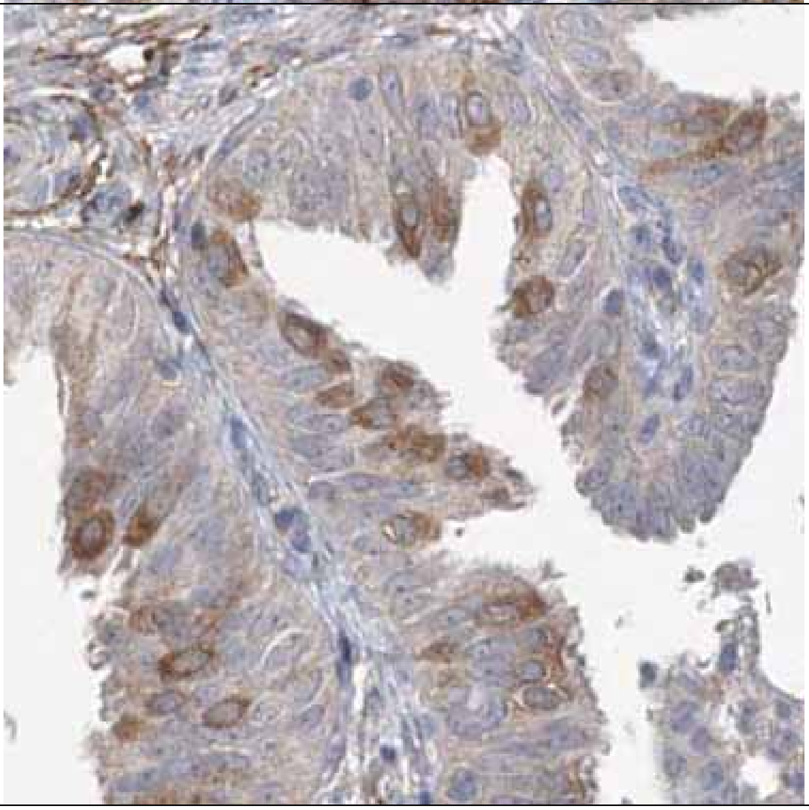	Staining is specific to *ciliated cells* (cytoplasm)

DZIP1L (antibody HPA030404)<br><br>Category 1	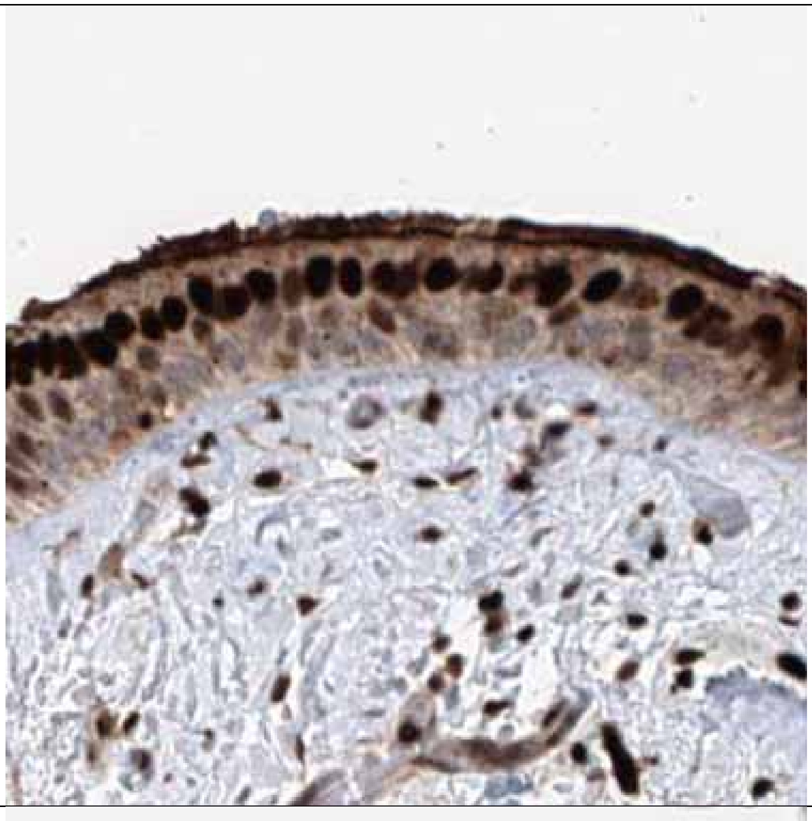	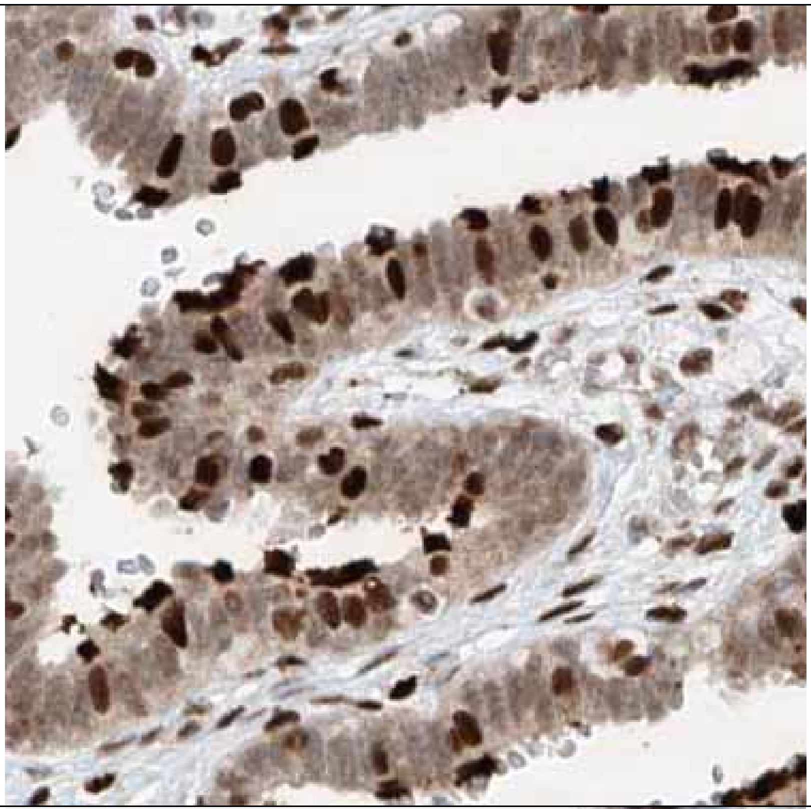	Staining is specific to *ciliated cells* (cilia and nucleus)
FOXJ1 (antibody HPA005714)<br><br>Category 1	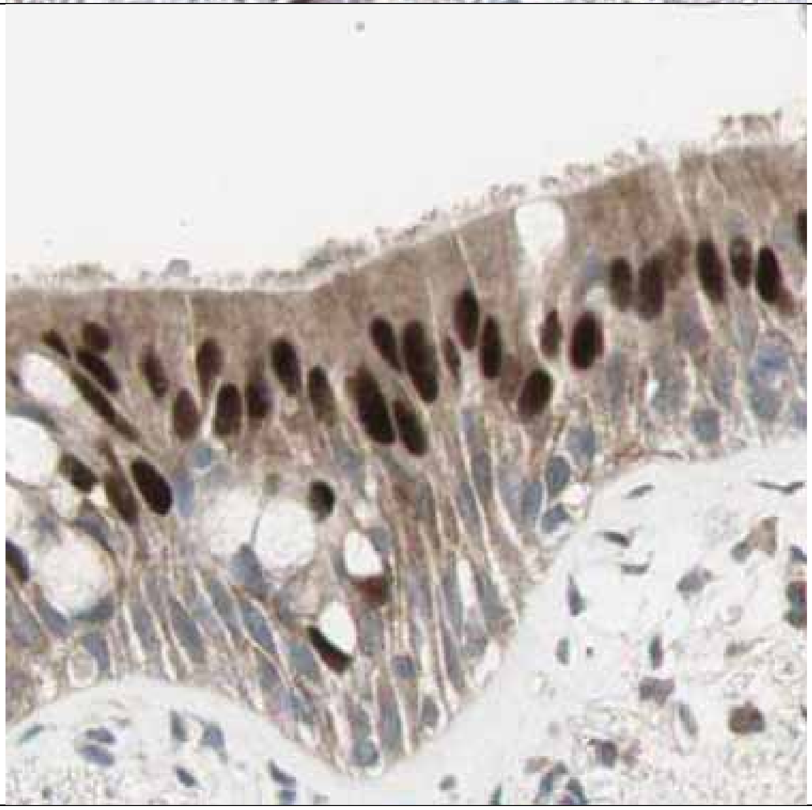	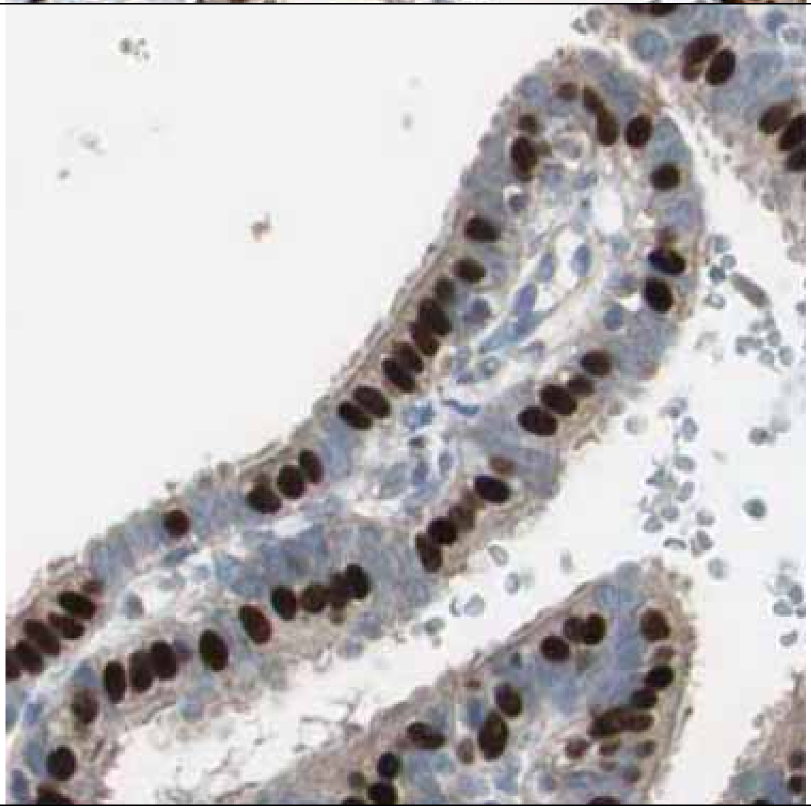	Staining is specific to *ciliated cells* (nucleus)

<table>
<tr><td>HSP90AA1
(antibody
CAB002058)

Category 1</td><td>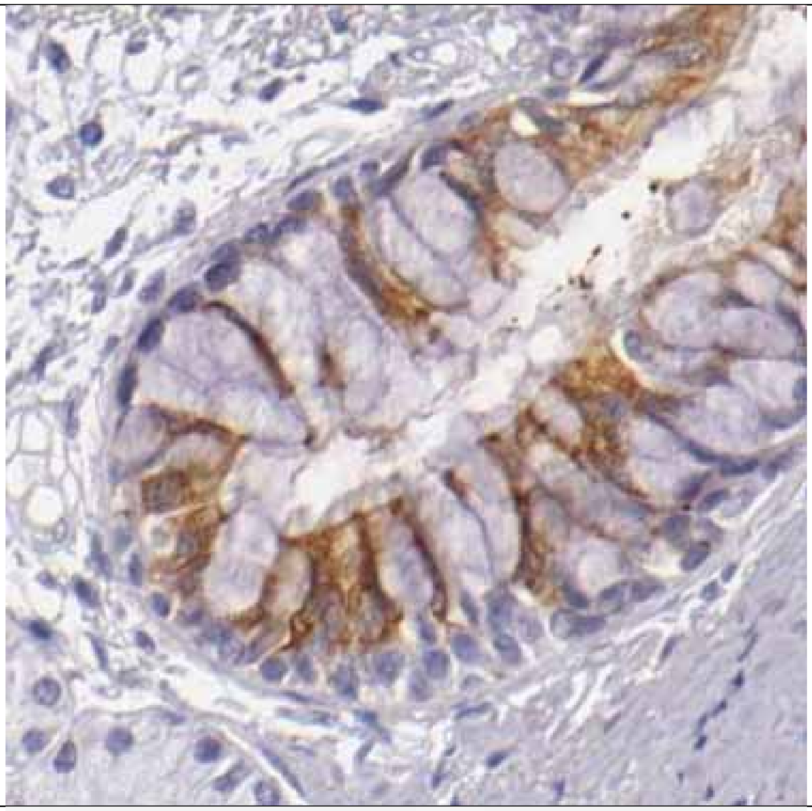</td><td>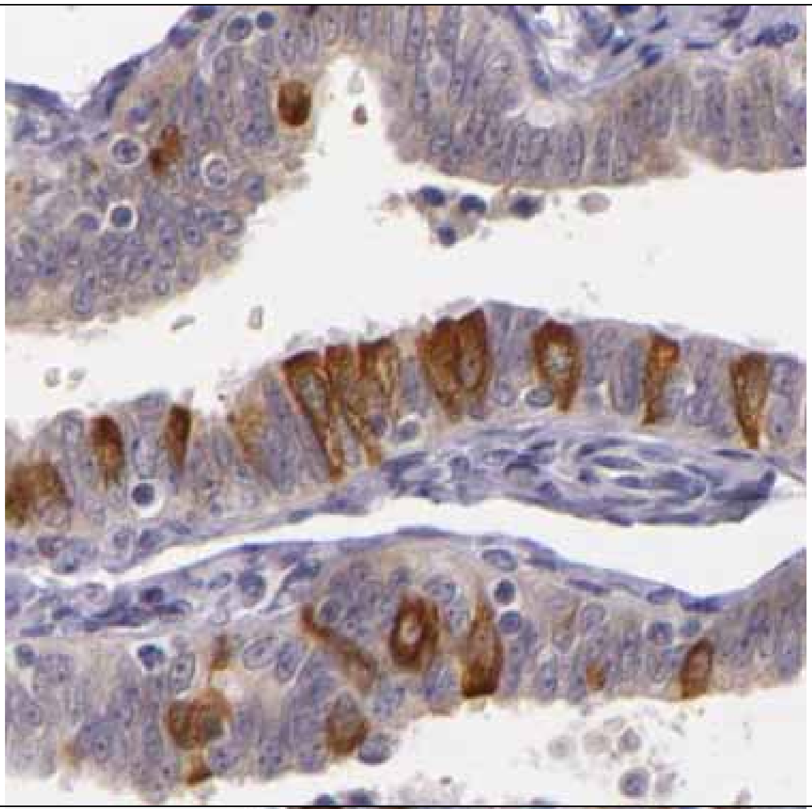</td><td>Staining is specific to ciliated cells (cytoplasm)</td></tr>
<tr><td>HSPB11
(antibody
HPA030140)

Category 1</td><td>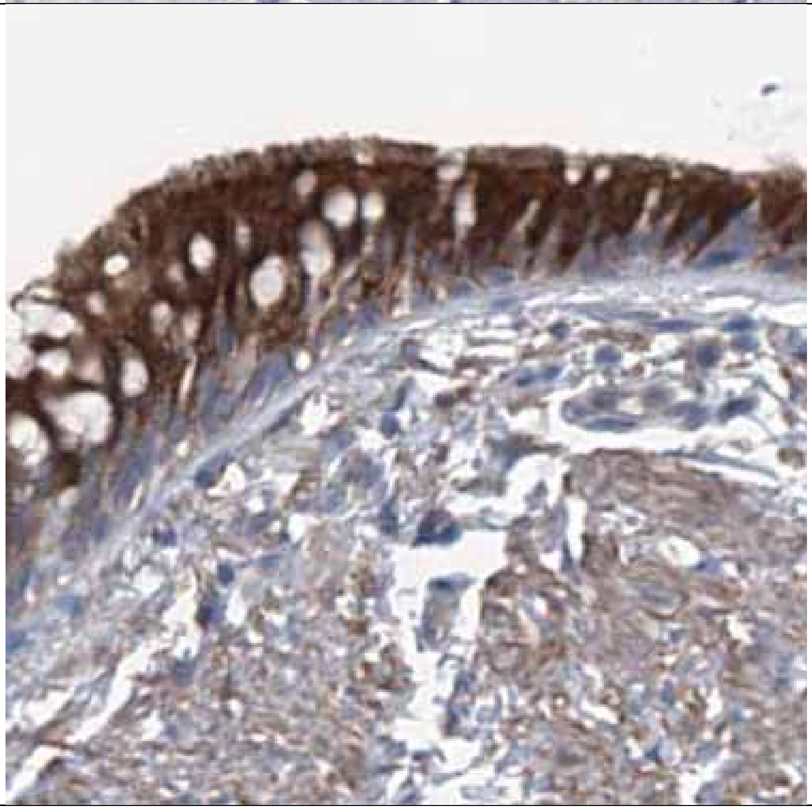</td><td>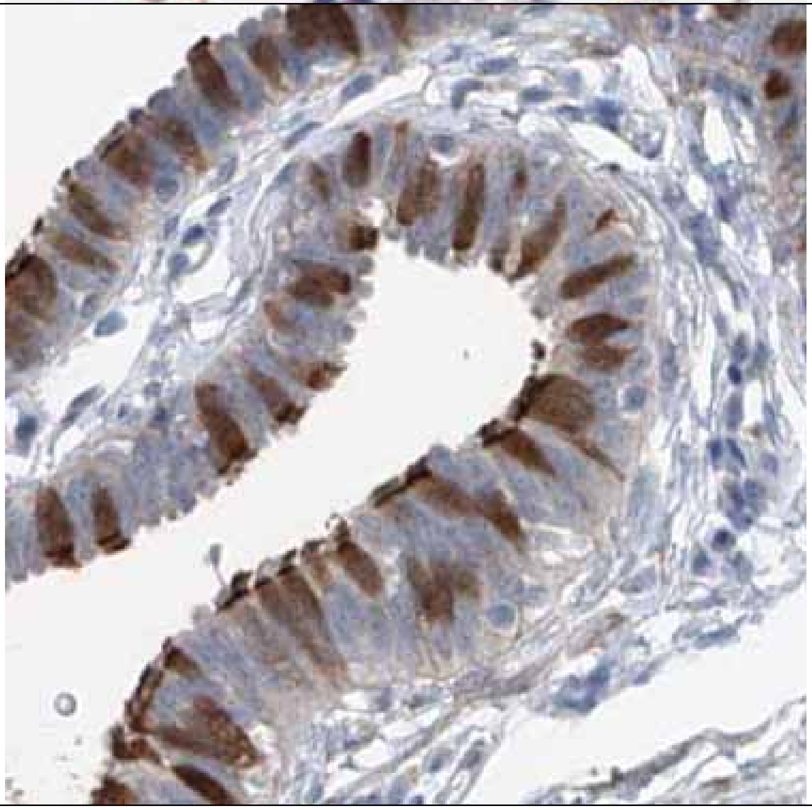</td><td>Staining is specific to ciliated cells (cilia and apical cytoplasm)</td></tr>
</table>

MAK (antibody HPA039092)<br><br>Category 1	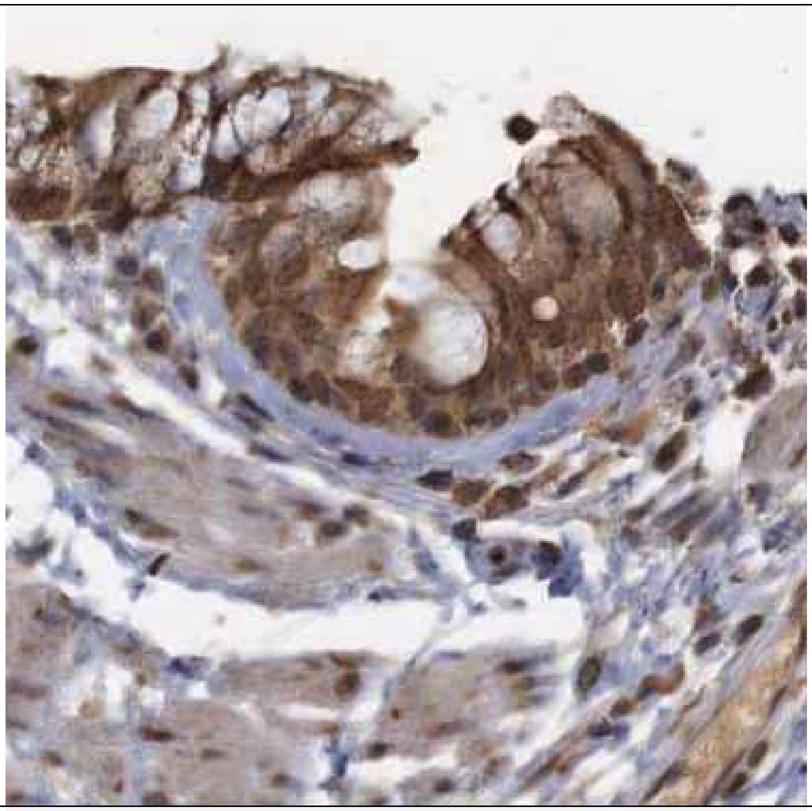	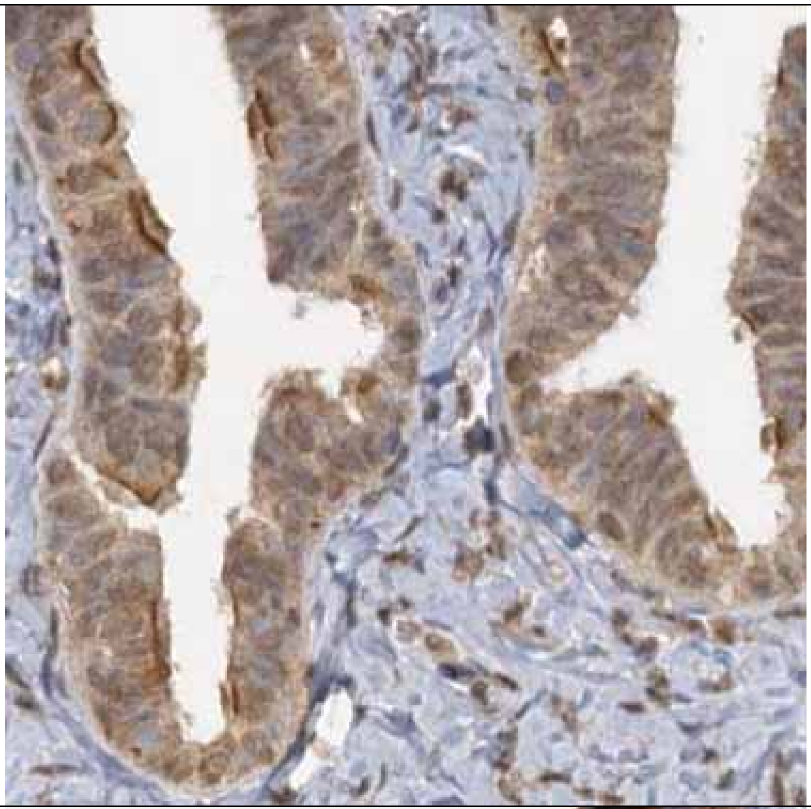	Staining is specific to *ciliated cells* (cilia and cytoplasm)
MAP9 (antibody HPA037864)<br><br>Category 1	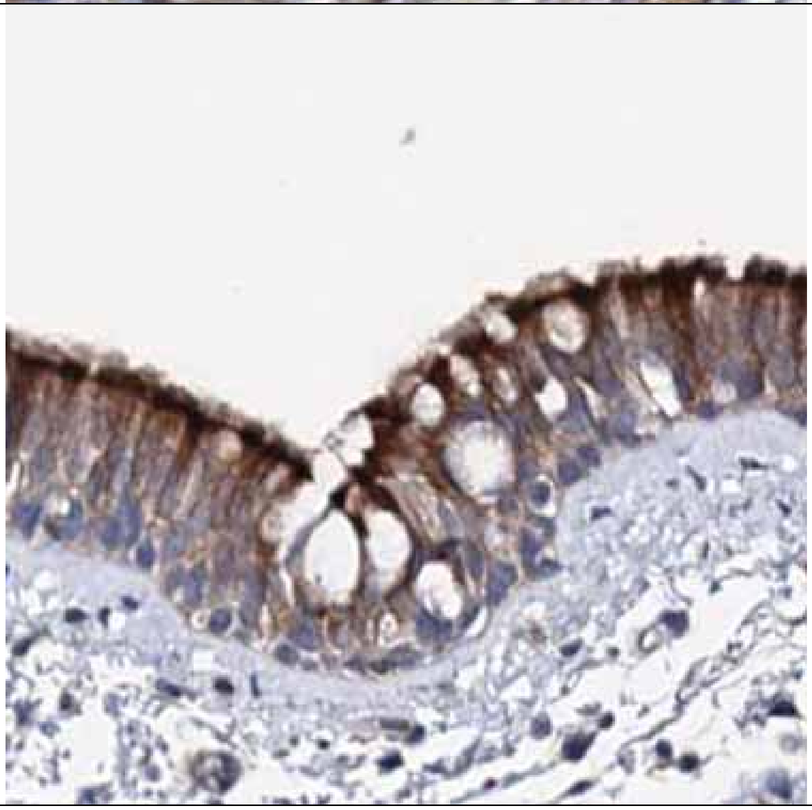	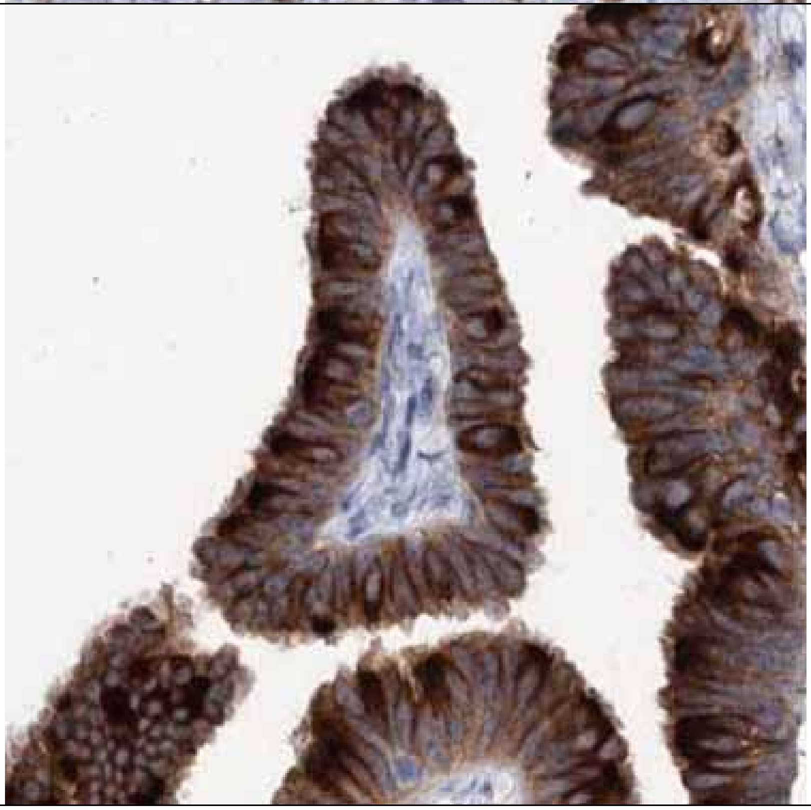	Staining is specific to *ciliated cells* (apical cytoplasm)

PTGES3 (antibody HPA038673)<br><br>Category 1	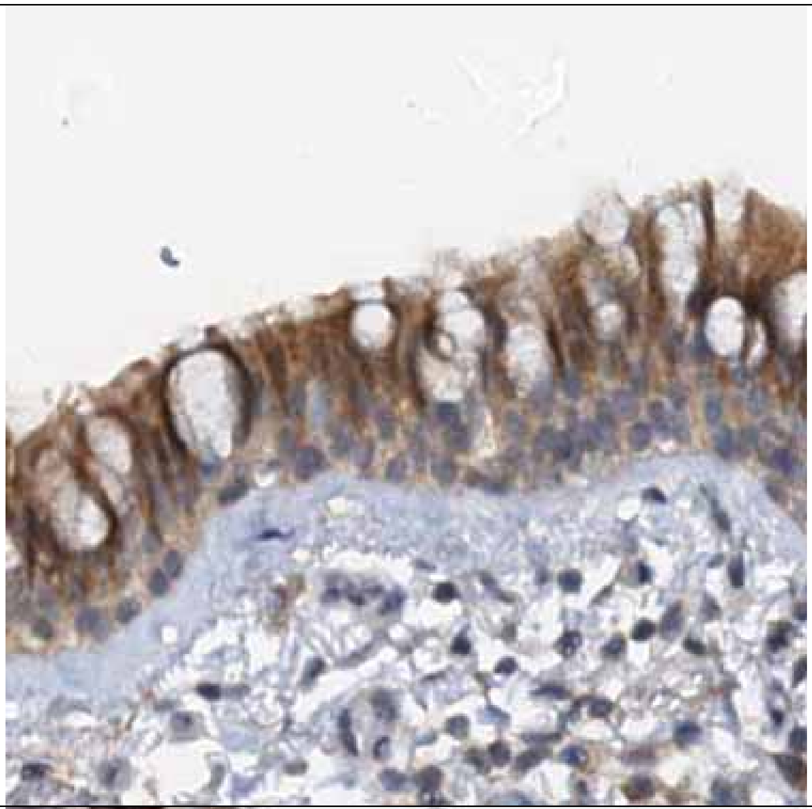	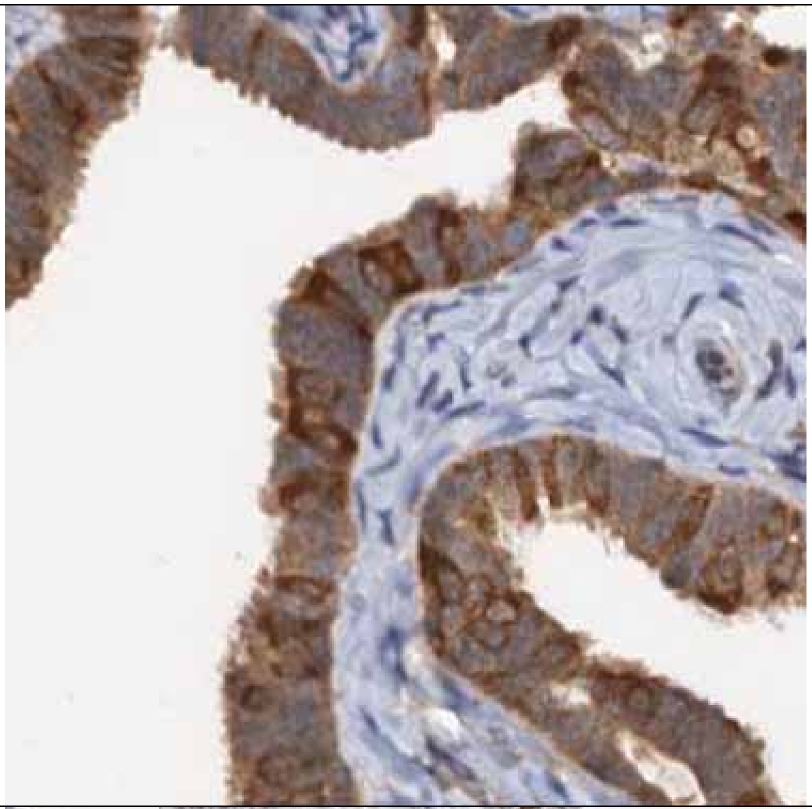	Staining is specific to *ciliated cells* (cytoplasm)
RFX3 (antibody HPA035689)<br><br>Category 1	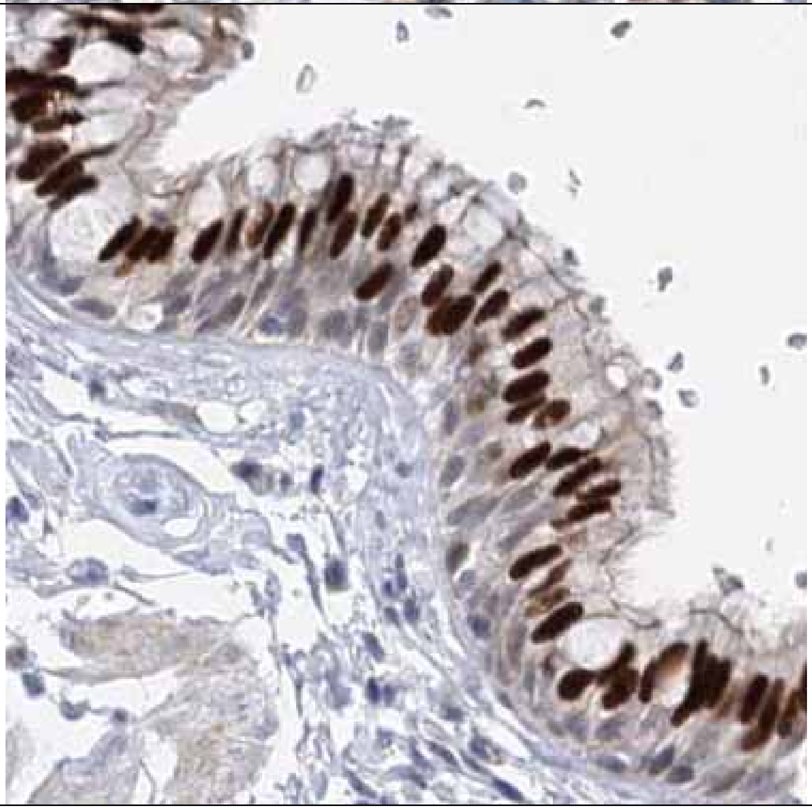	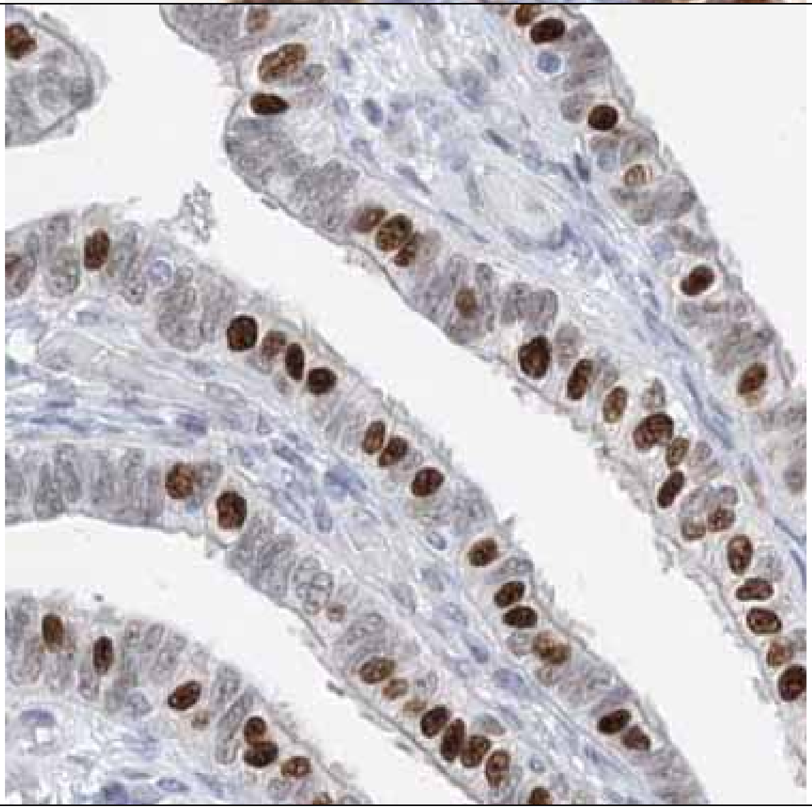	Staining is specific to *ciliated cells* (nucleus)

SCGB2A1 (antibody HPA034584)<br><br>Category 1	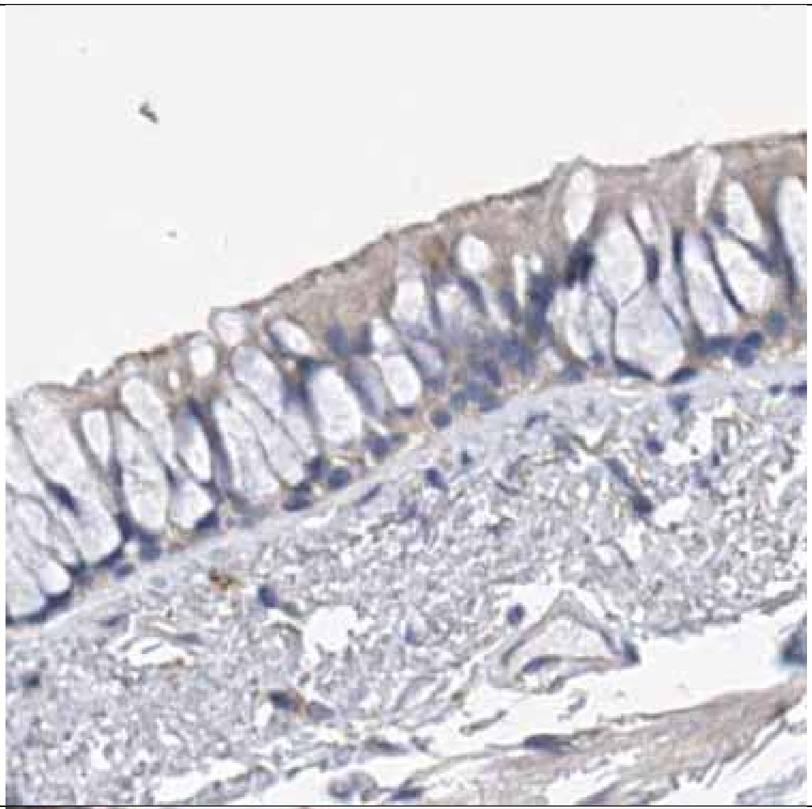	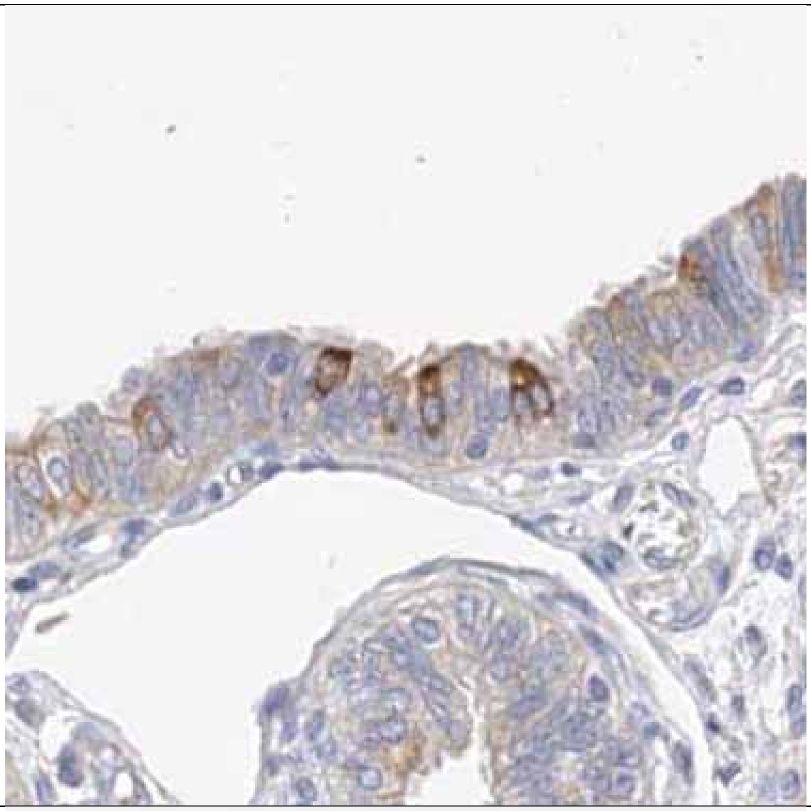	Staining is specific to *ciliated cells* (uncertain subcellular localization)
SLC22A4 (antibody CAB015468)<br><br>Category 1	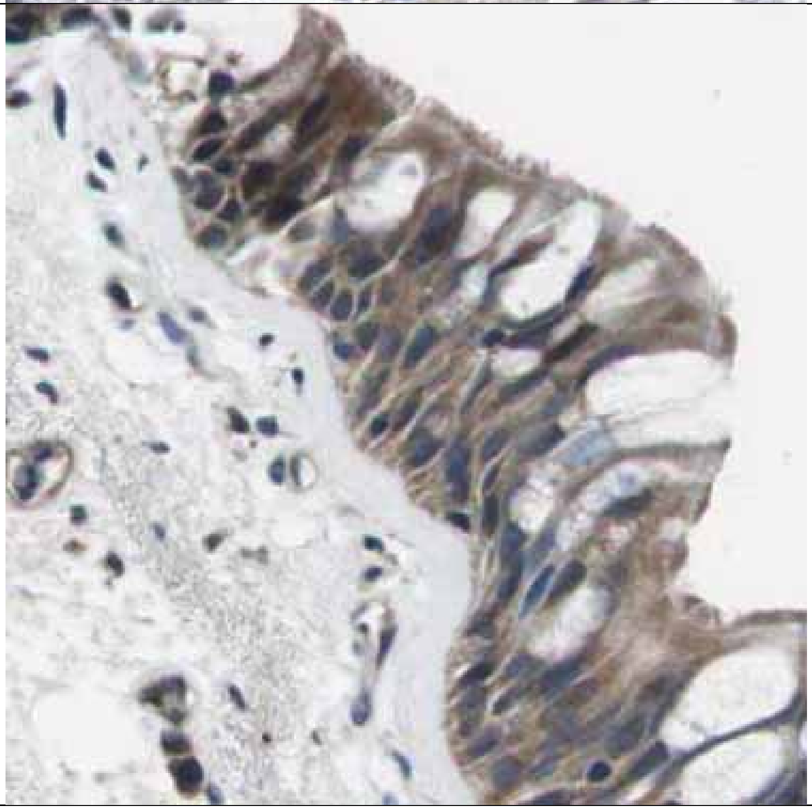	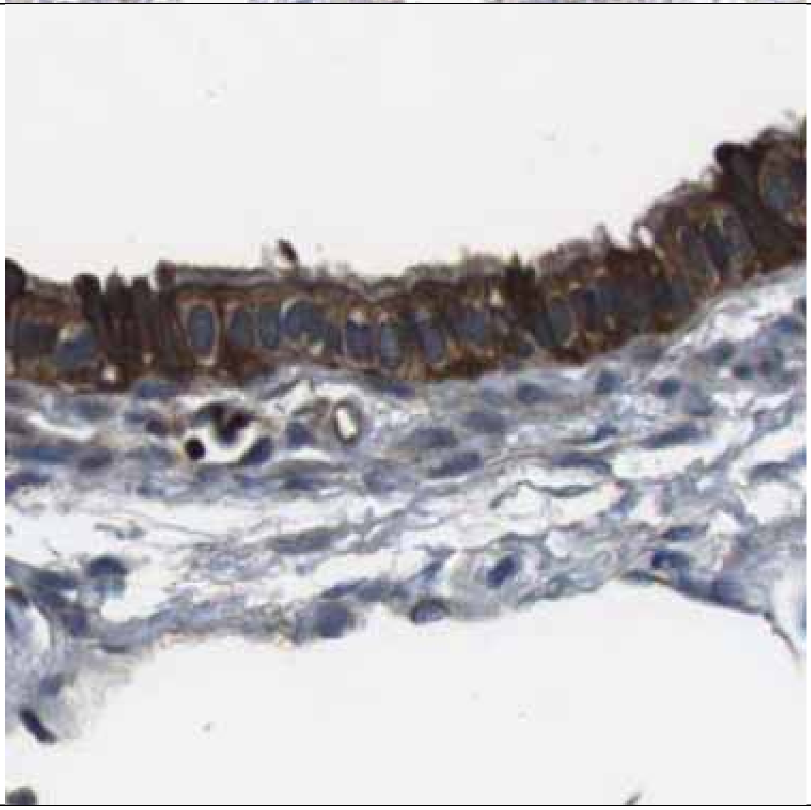	Staining is specific to *ciliated cells* (cilia and cytoplasm)

SLC27A2<br>(antibody<br>HPA026089)<br><br>Category 1	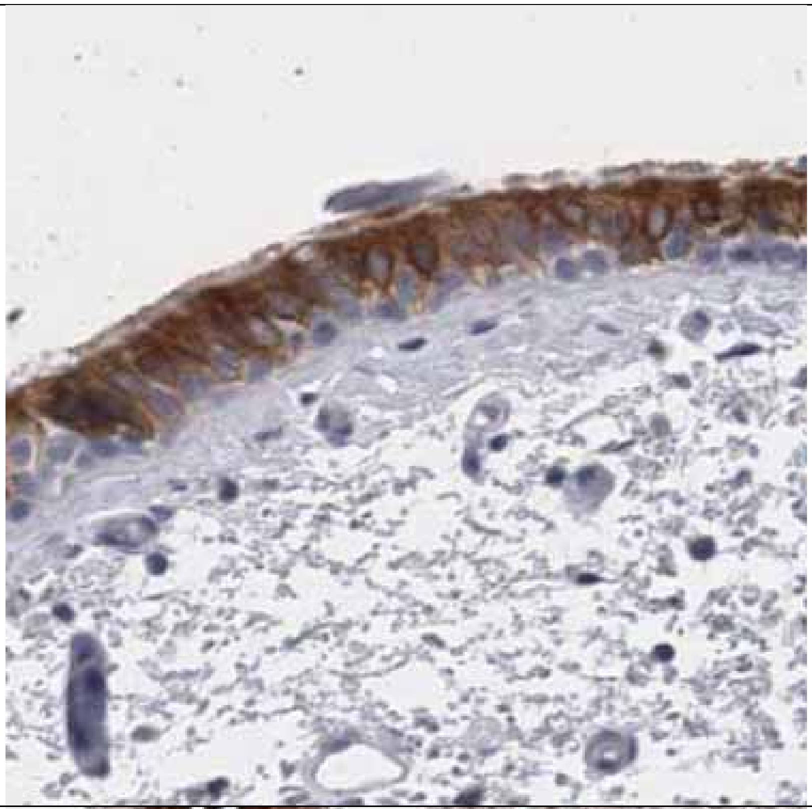	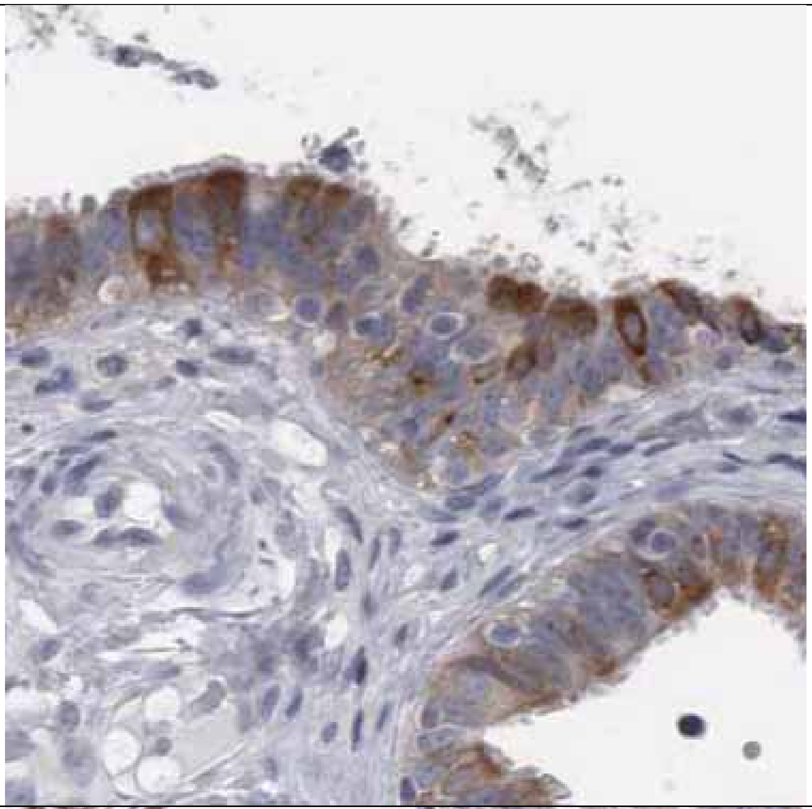	Staining is specific to *ciliated cells* (uncertain subcellular localization)
SPAG16<br>(antibody<br>HPA037542)<br><br>Category 1	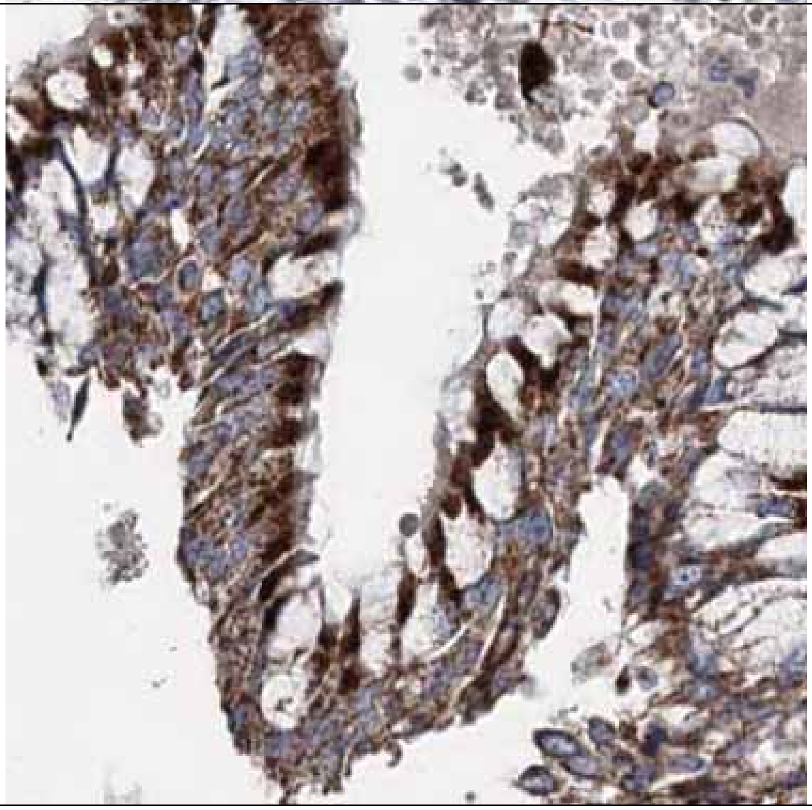	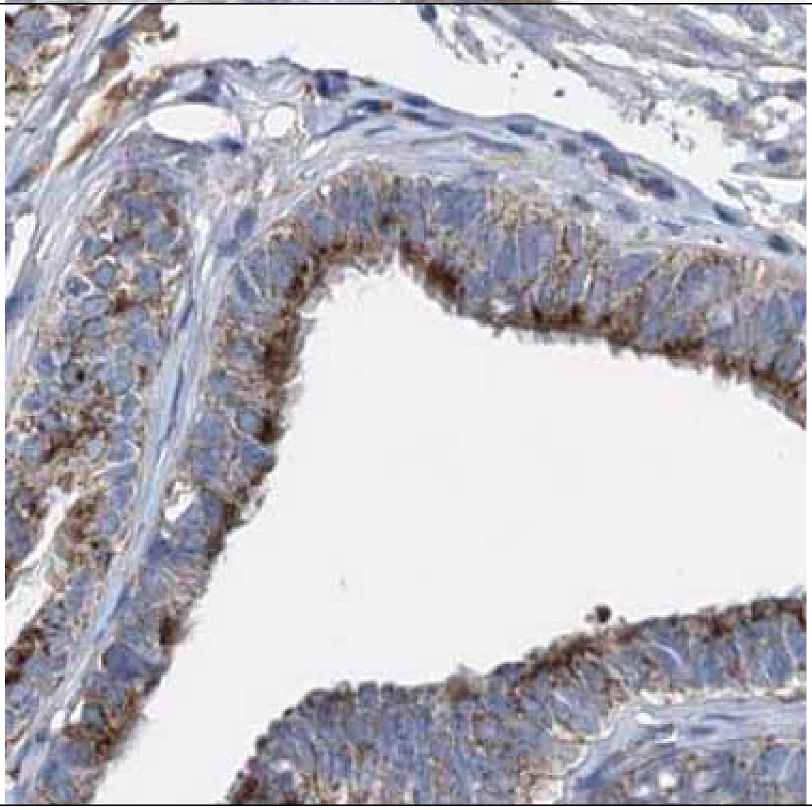	Staining is specific to *ciliated cells* (cytoplasm)

SPATA6 (antibody HPA028326)<br><br>Category 1	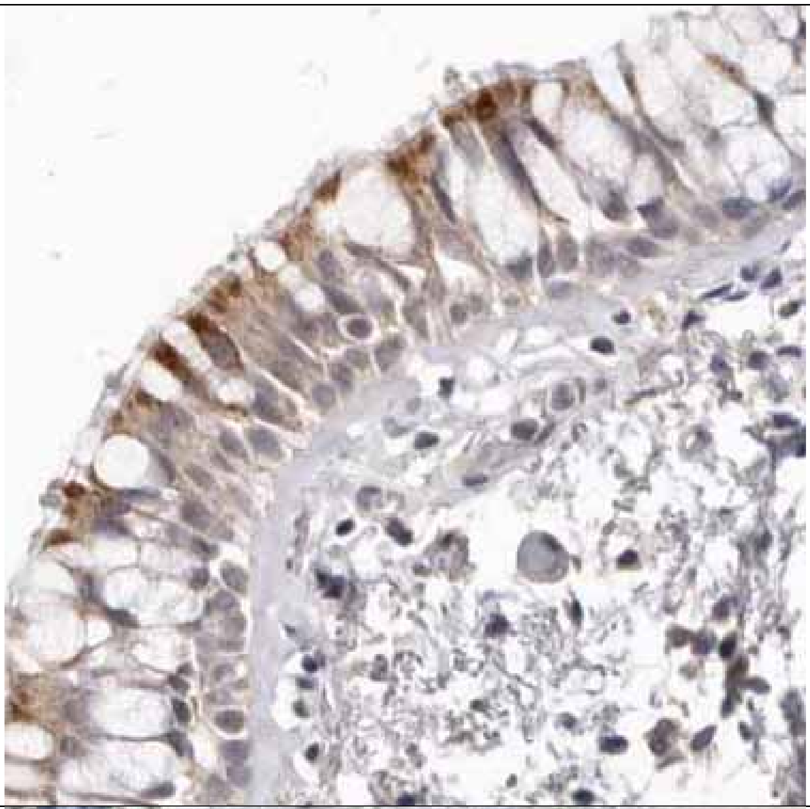	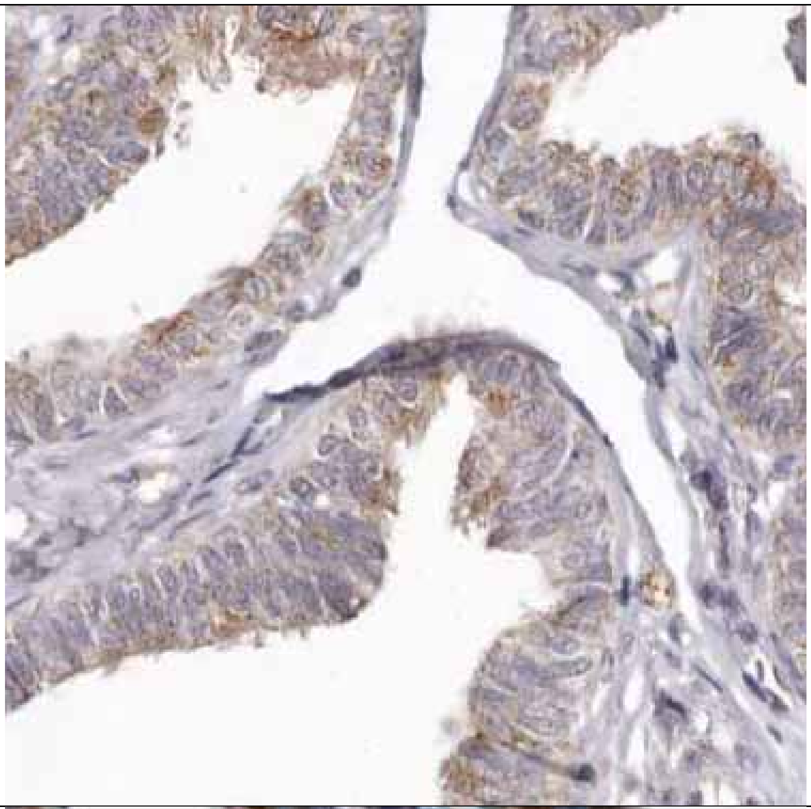	Staining is specific to *ciliated cells* (apical cytoplasm)
STK33 (antibody HPA015742)<br><br>Category 1	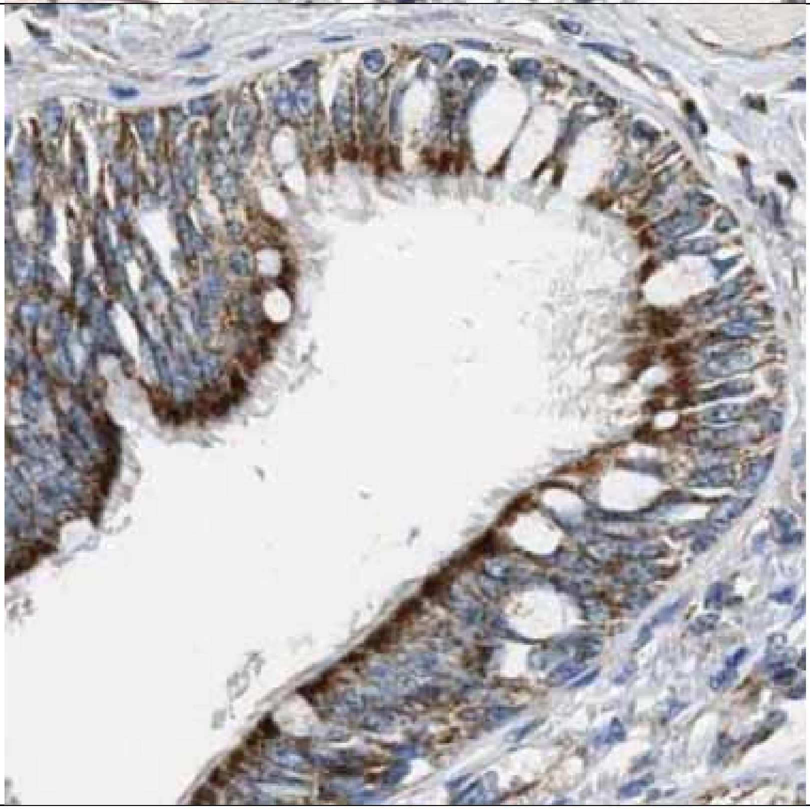	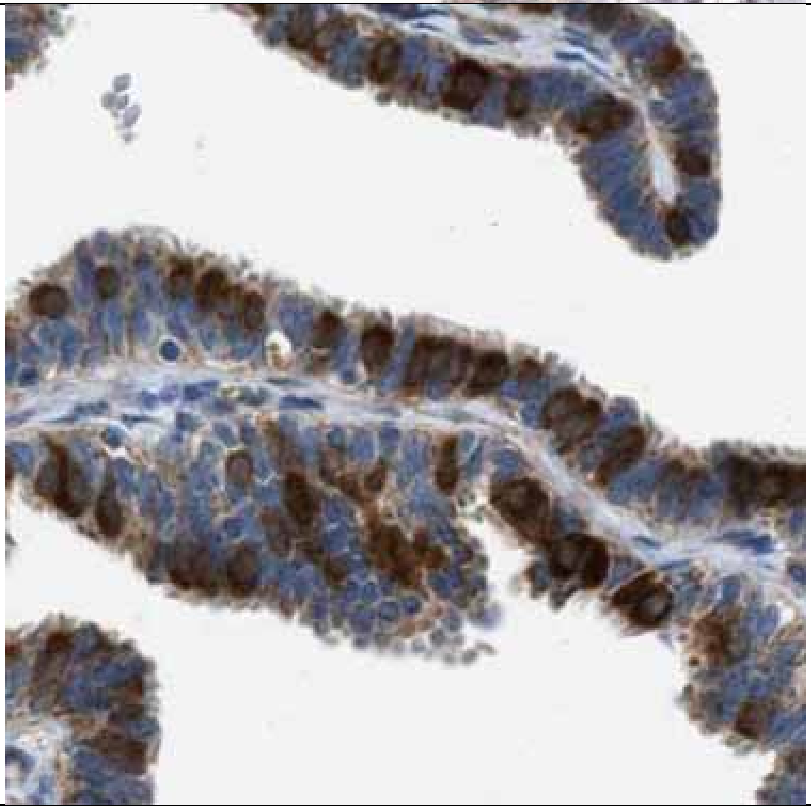	Staining is specific to *ciliated cells* (cytoplasm)

TMEM67 (antibody HPA039940)<br><br>Category 1	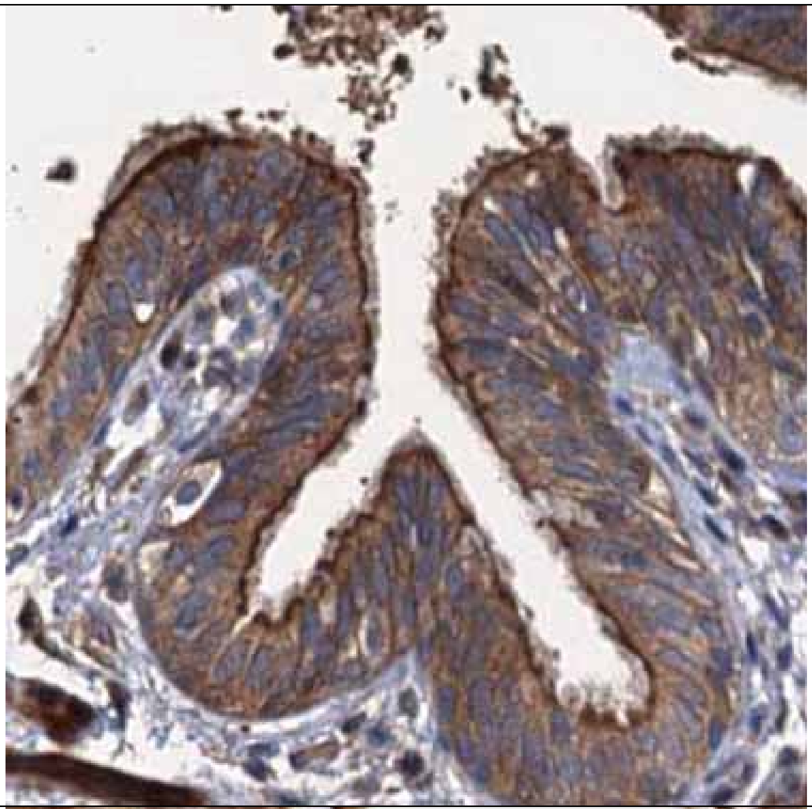	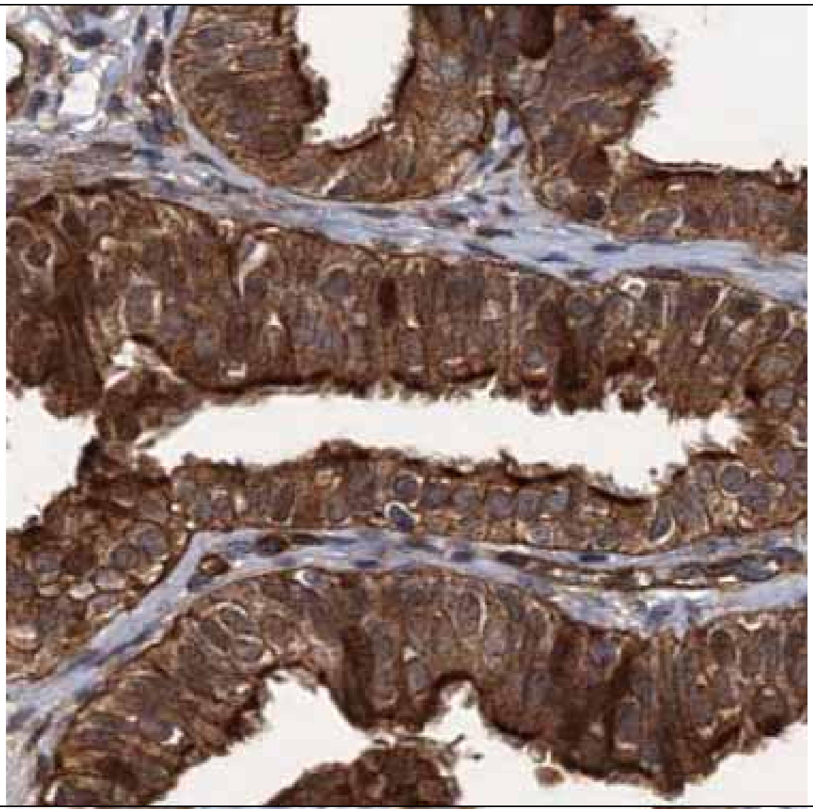	Staining is specific to *ciliated cells* (apical region of cytoplasm)
TRAF3IP1 (antibody HPA037858)<br><br>Category 1	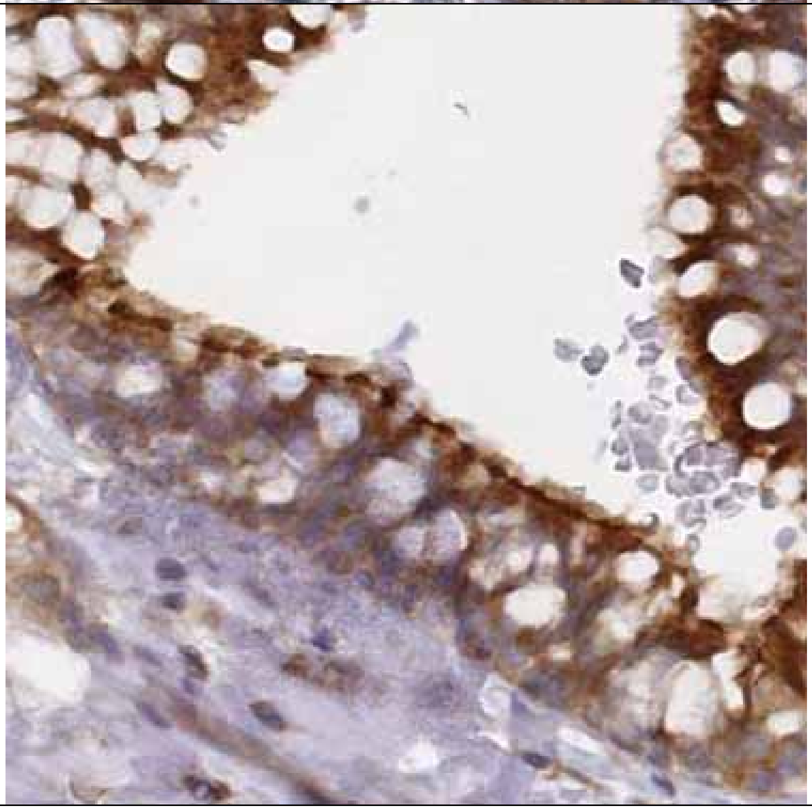	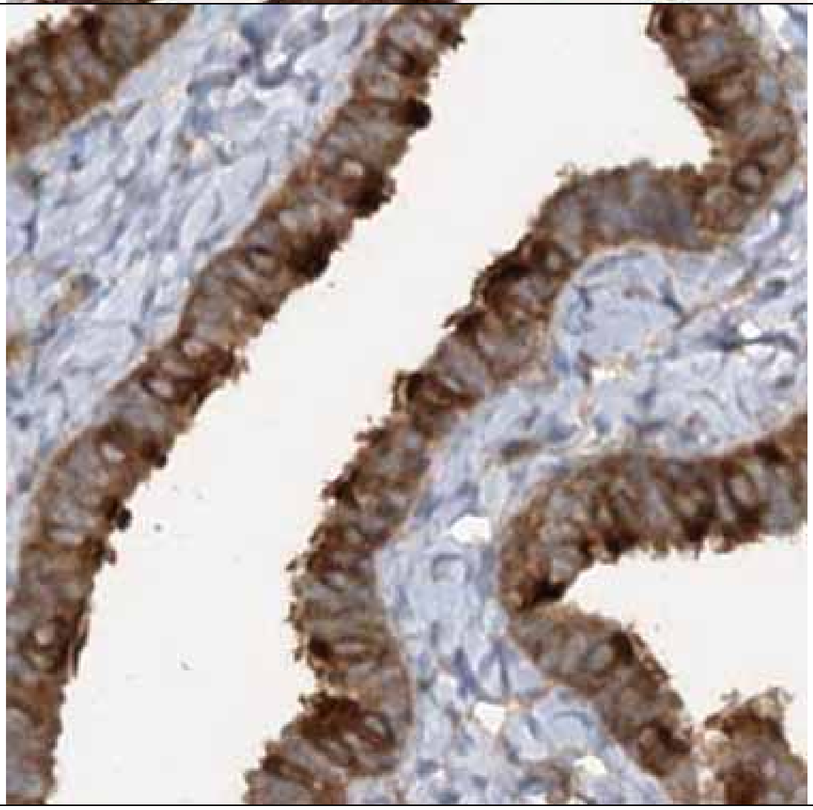	Staining is specific to *ciliated cells* (apical region of cytoplasm)

TSGA10 (antibody HPA036879)<br><br>Category 1	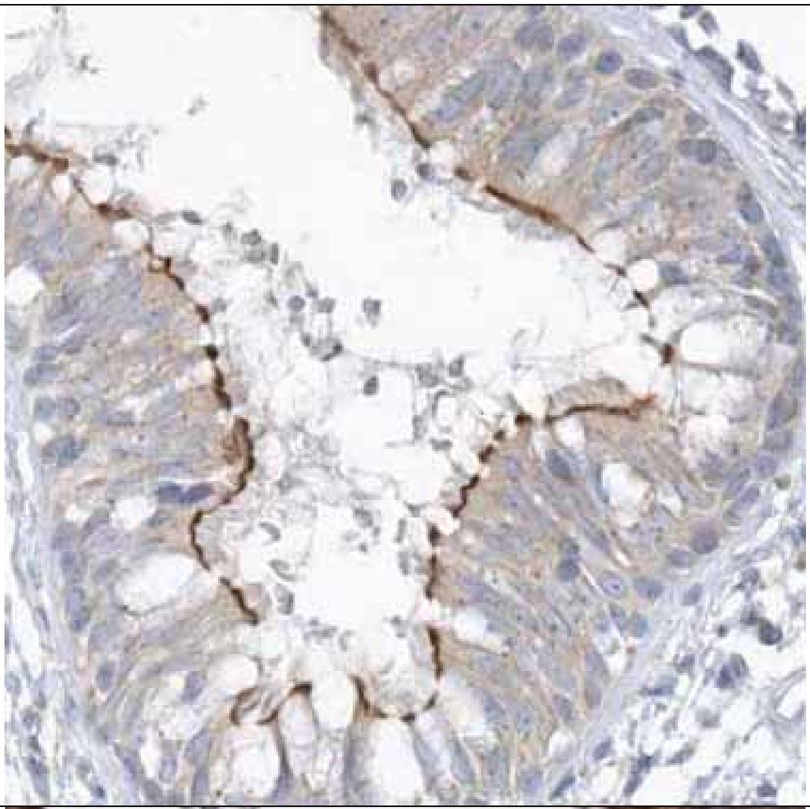	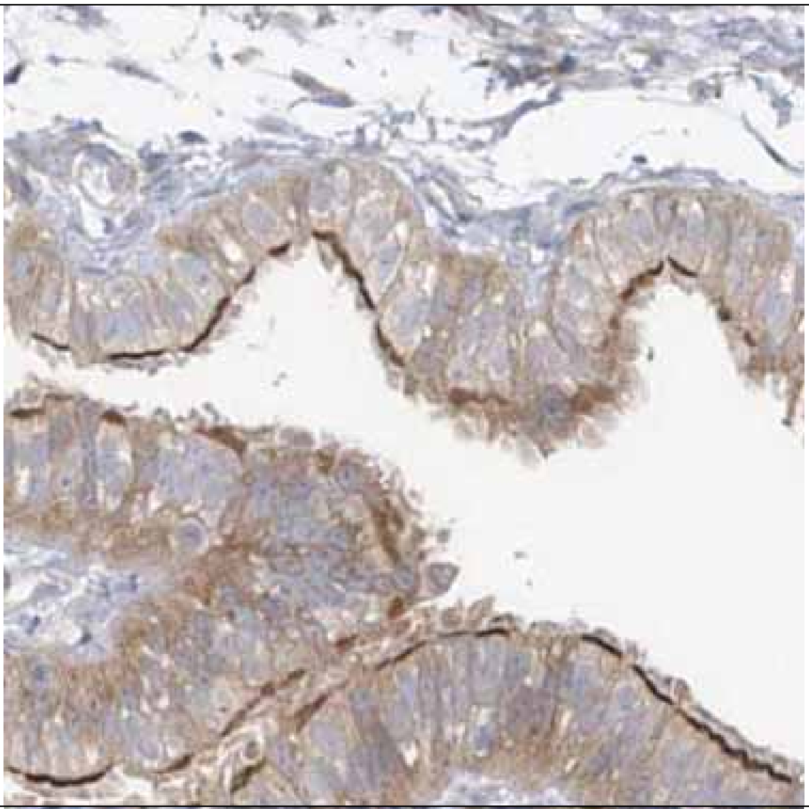	Staining is specific to *ciliated cells* (apical region of cytoplasm)
TTLL6 (antibody HPA023653)<br><br>Category 1	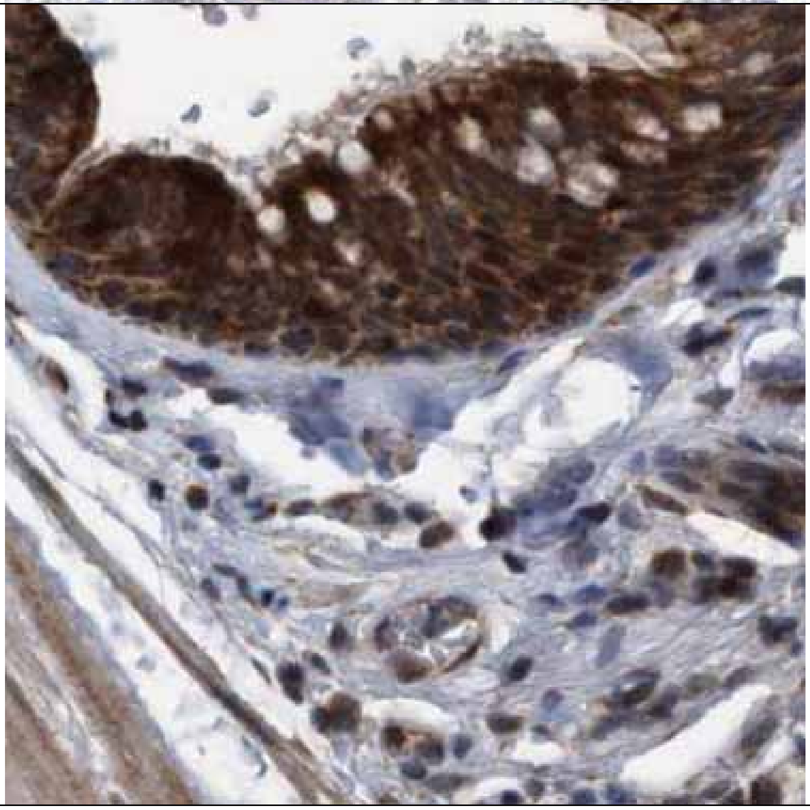	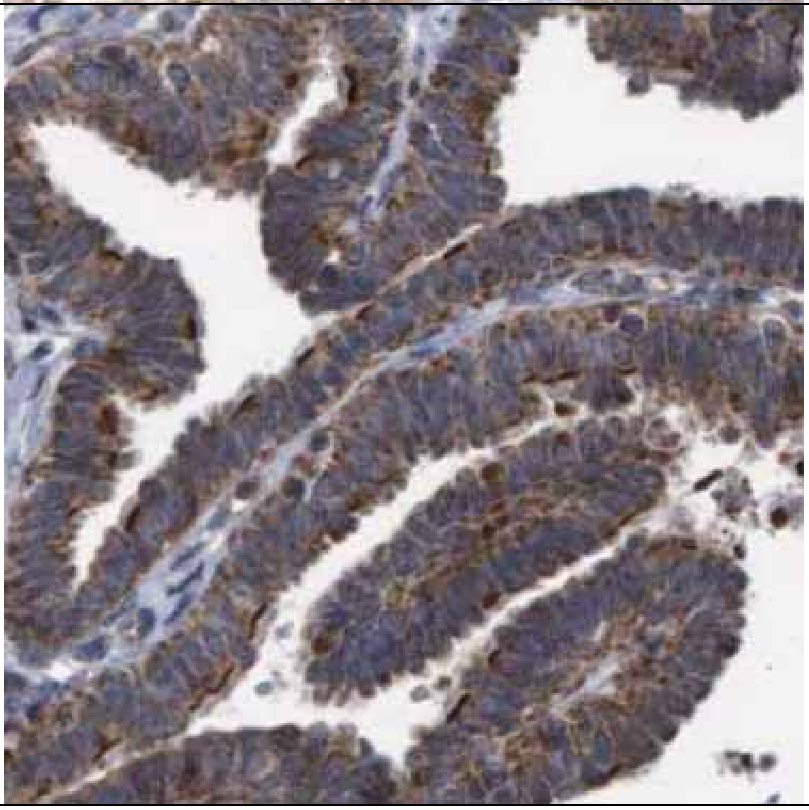	Staining is specific to *ciliated cells* (apical region of cytoplasm)

VWA3B (antibody HPA036701)<br><br>Category 1	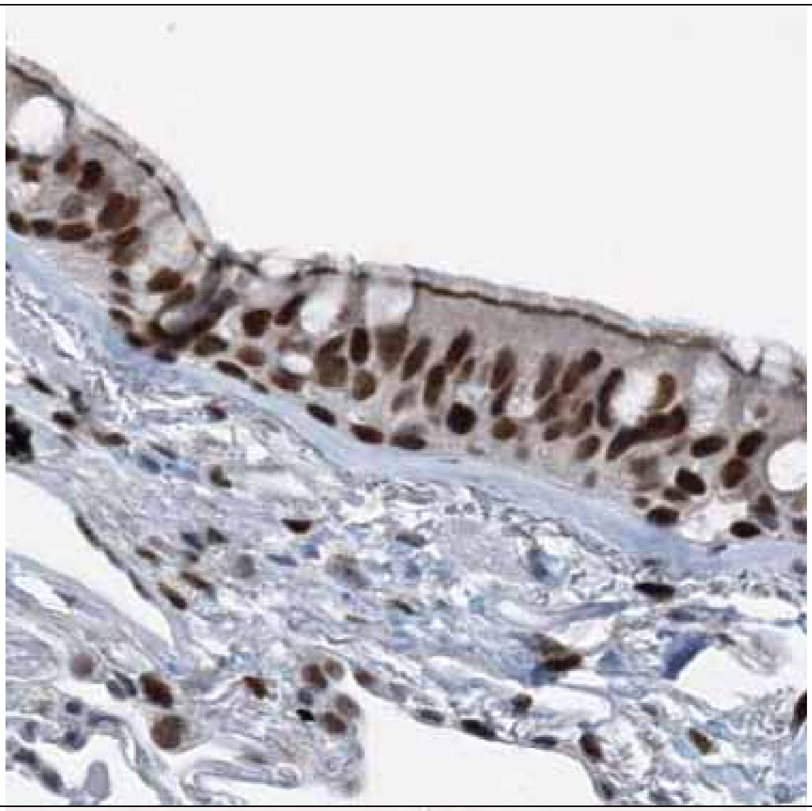	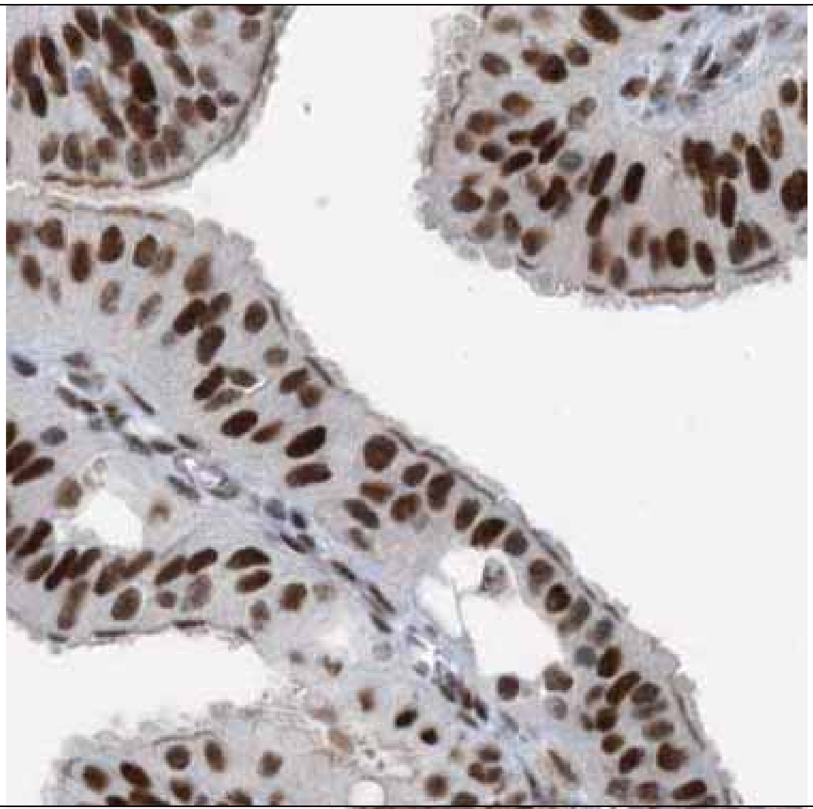	Staining is specific to *ciliated cells* (nucleus & apical region of cytoplasm)
ZNF474 (antibody HPA036595)<br><br>Category 1	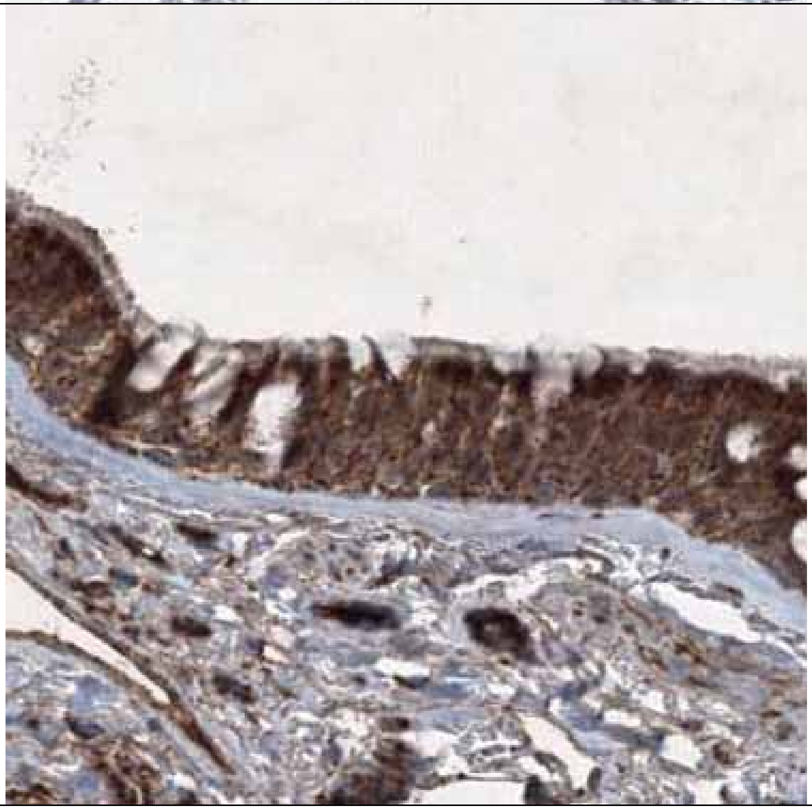	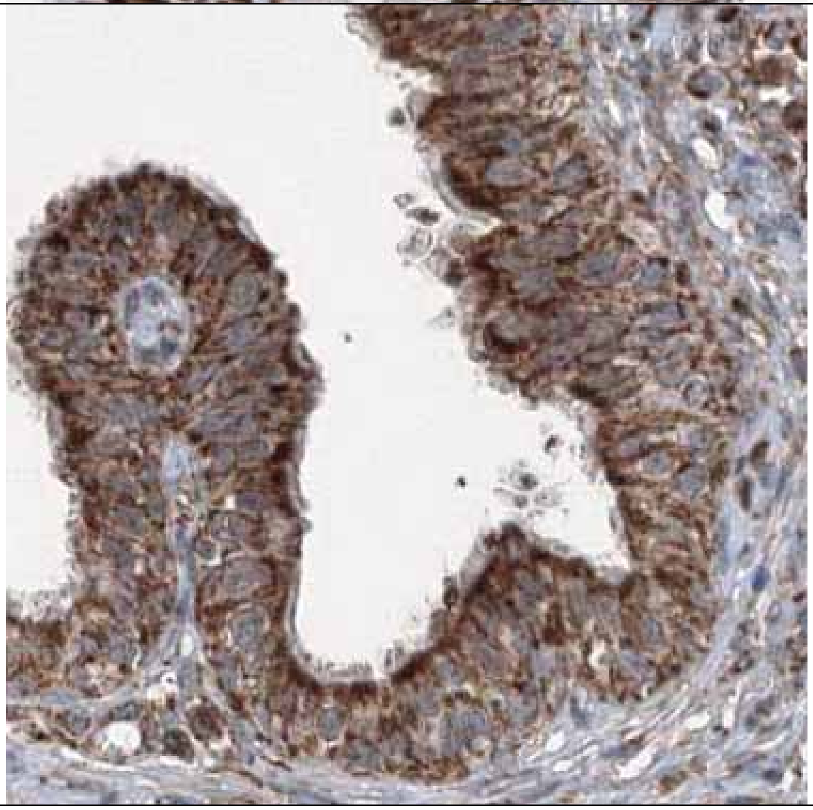	Staining is specific to *ciliated cells* (apical region of cytoplasm)

CAT (antibody CAB001515)<br><br>Category 1	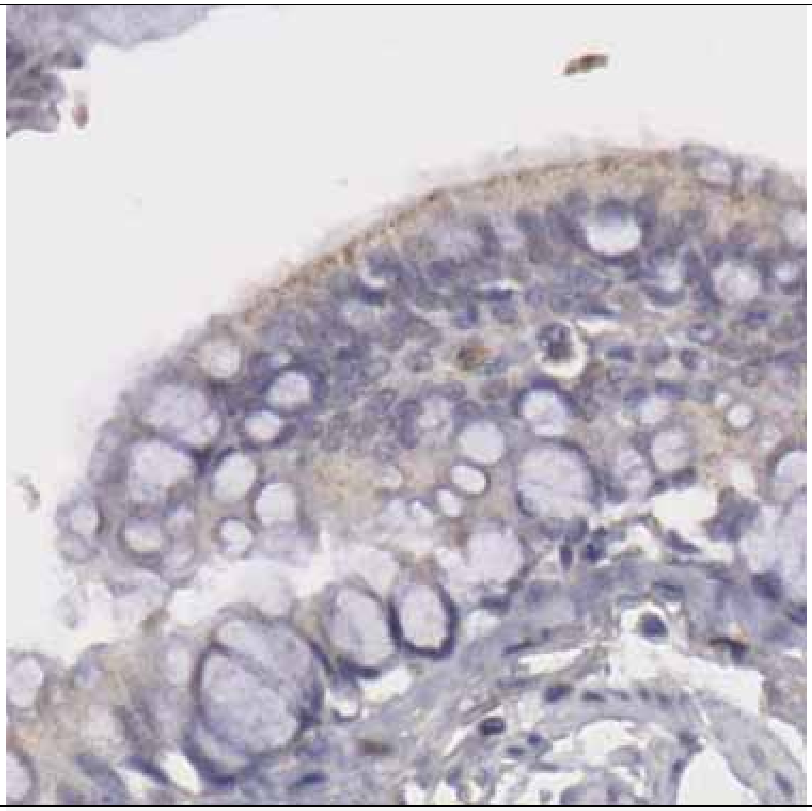	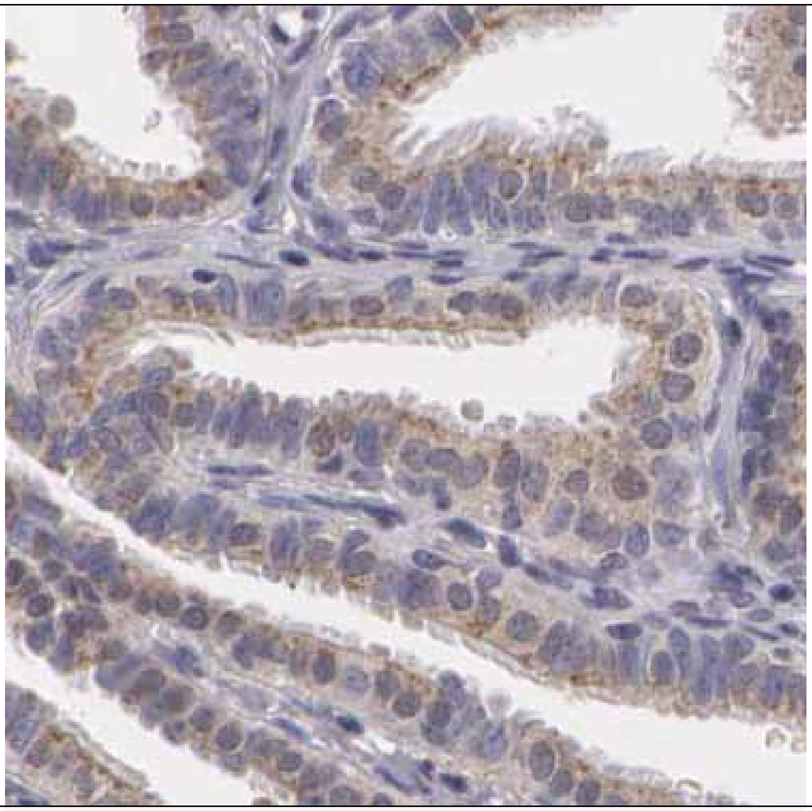	Staining is non-specific to ciliated cells or absent
CCDC17 (antibody HPA028338)<br><br>Category 1	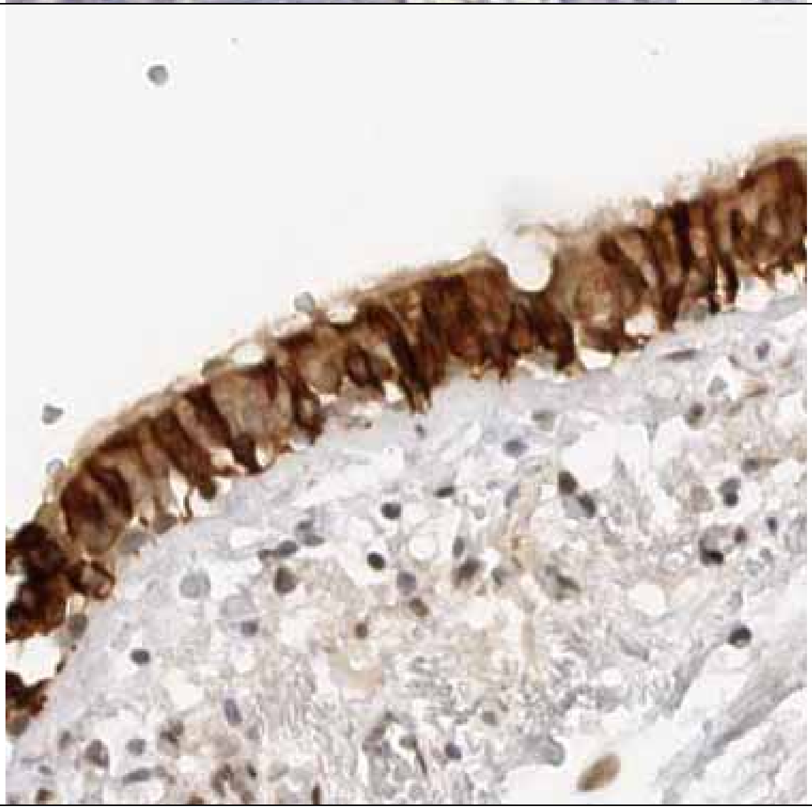	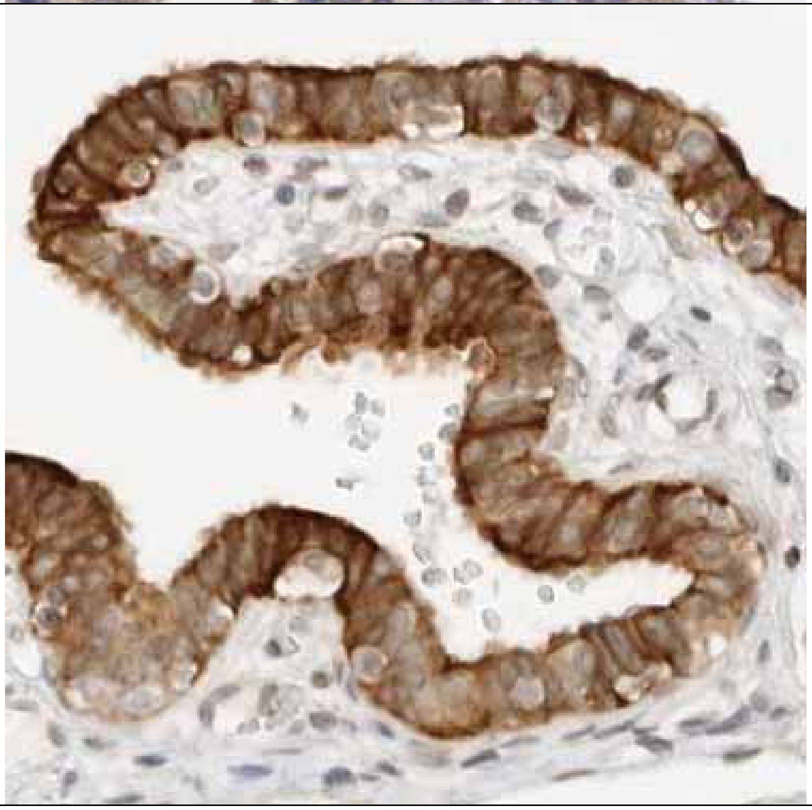	Staining is non-specific to ciliated cells or absent

CCDC65 (antibody HPA038520)<br><br>Category 1	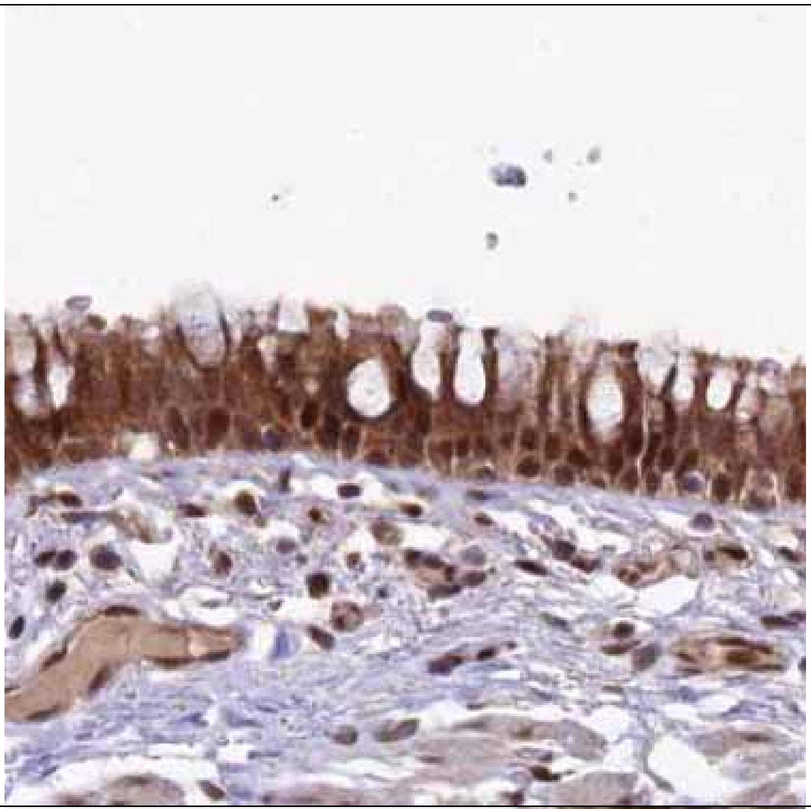	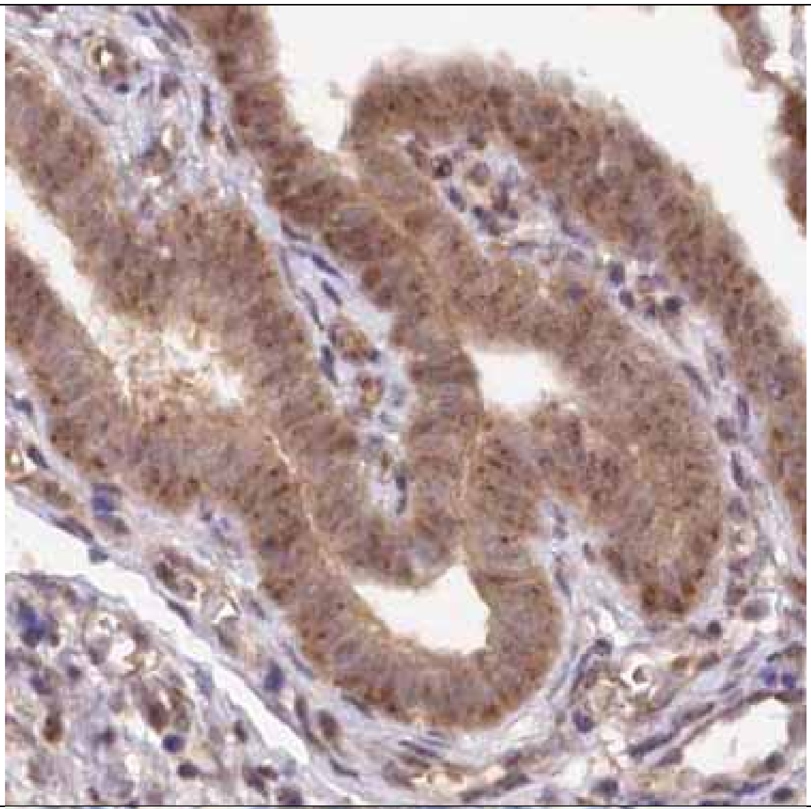	Staining is non-specific to ciliated cells or absent
CHST9 (antibody HPA011393)<br><br>Category 1	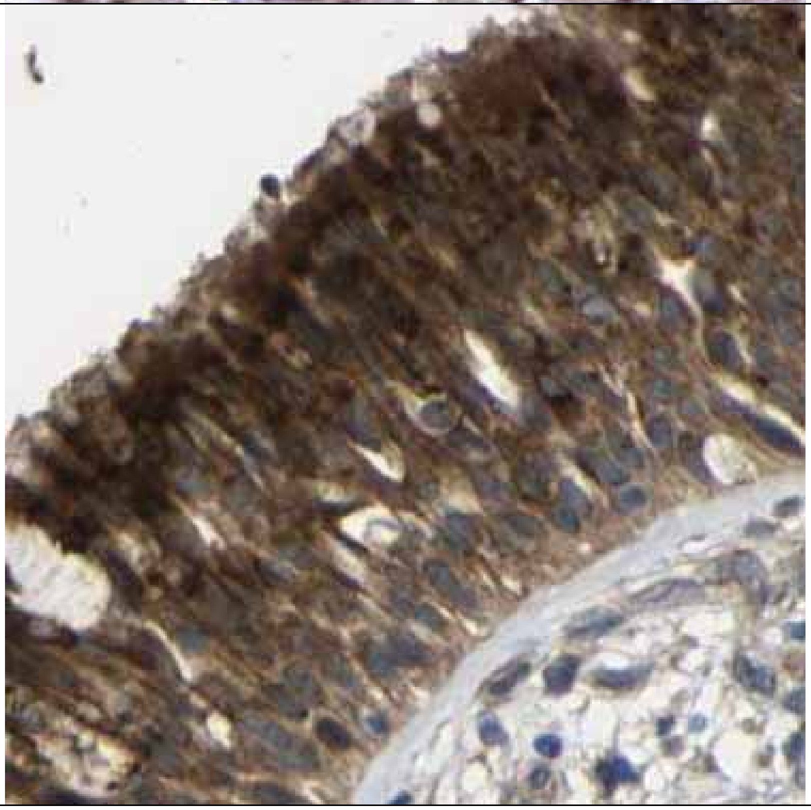	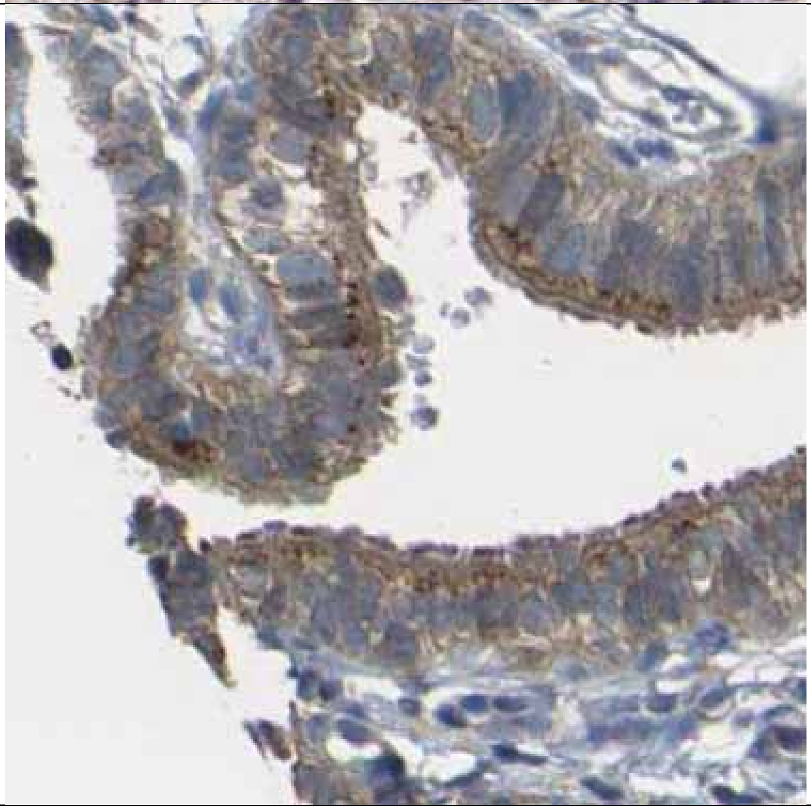	Staining is non-specific to ciliated cells or absent

C14orf45 (antibody HPA003136)<br><br>Category 1	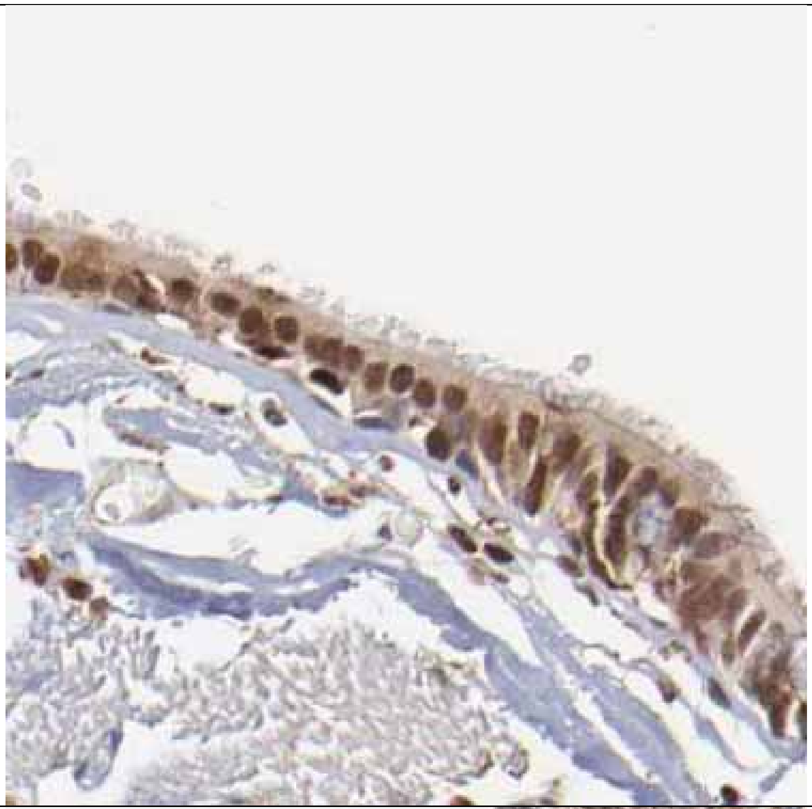	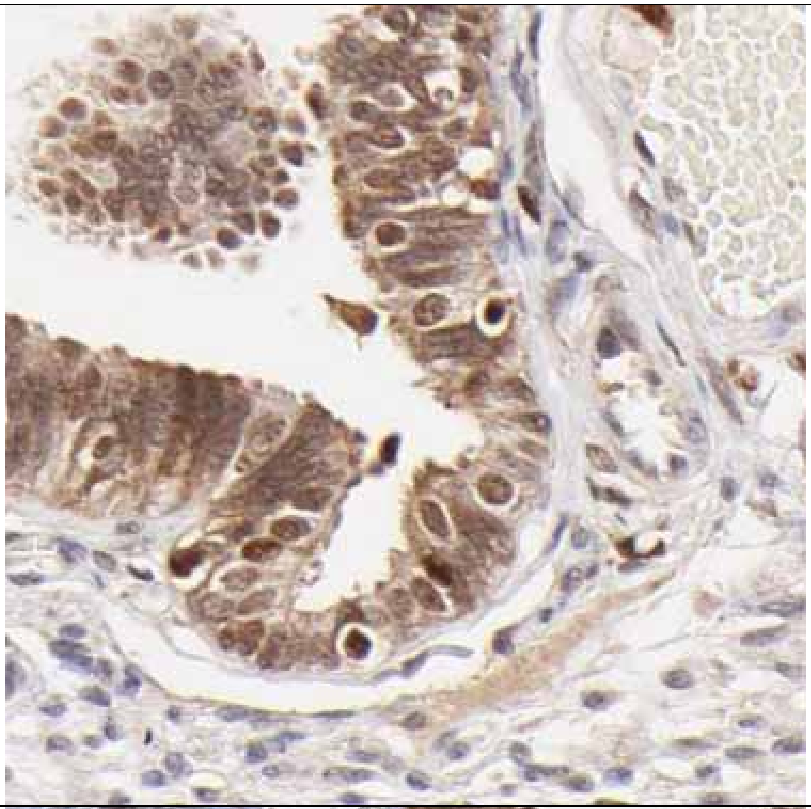	Staining is non-specific to ciliated cells or absent
C14orf50 (antibody HPA036801)<br><br>Category 1	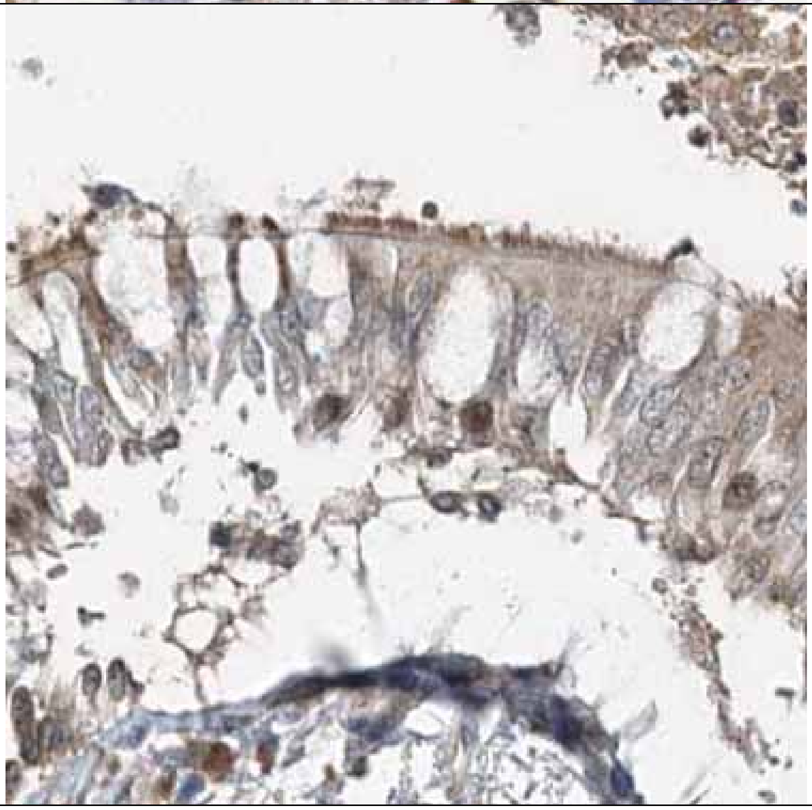	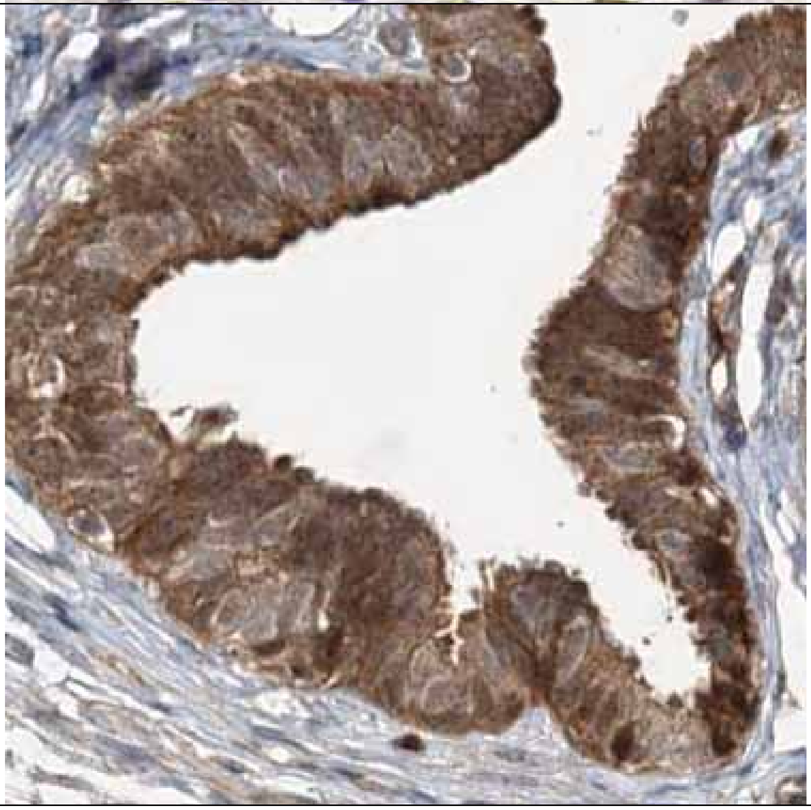	Staining is non-specific to ciliated cells or absent

C2orf40 (antibody HPA008546)<br><br>Category 1	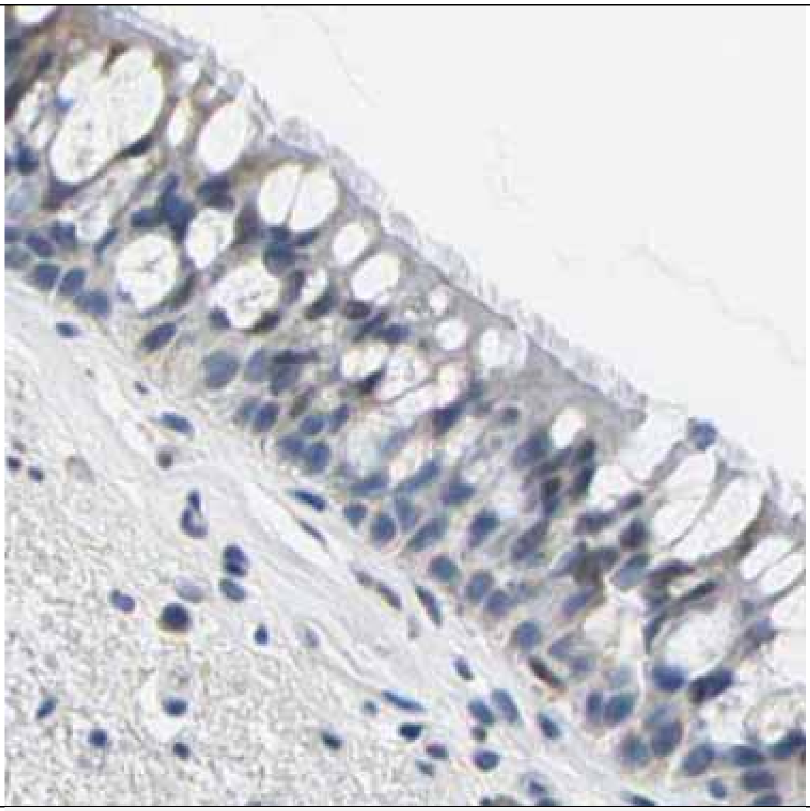	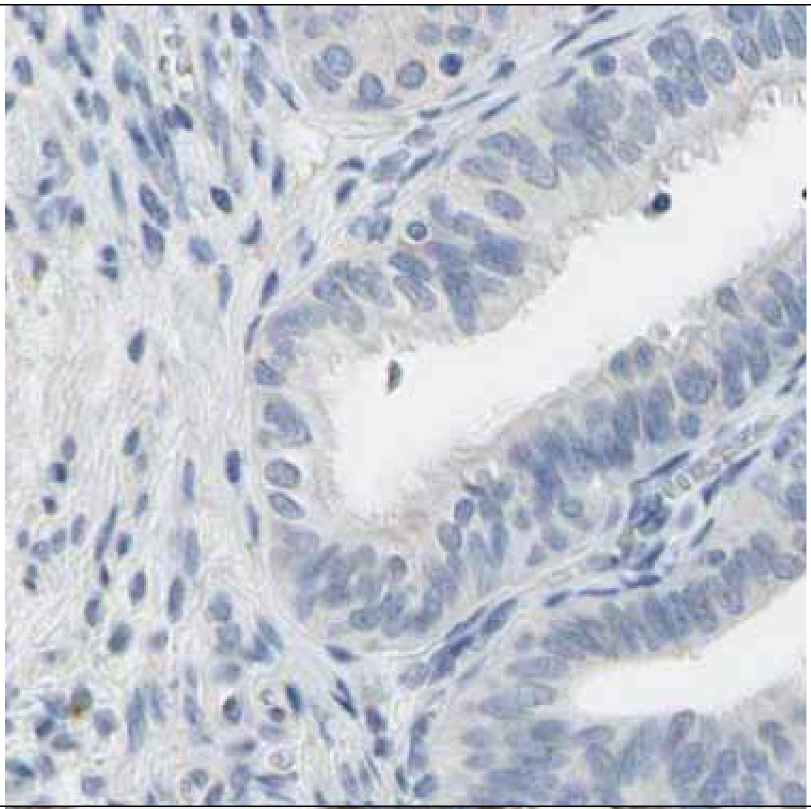	Staining is non-specific to ciliated cells or absent
DNAJA4 (antibody CAB004646)<br><br>Category 1	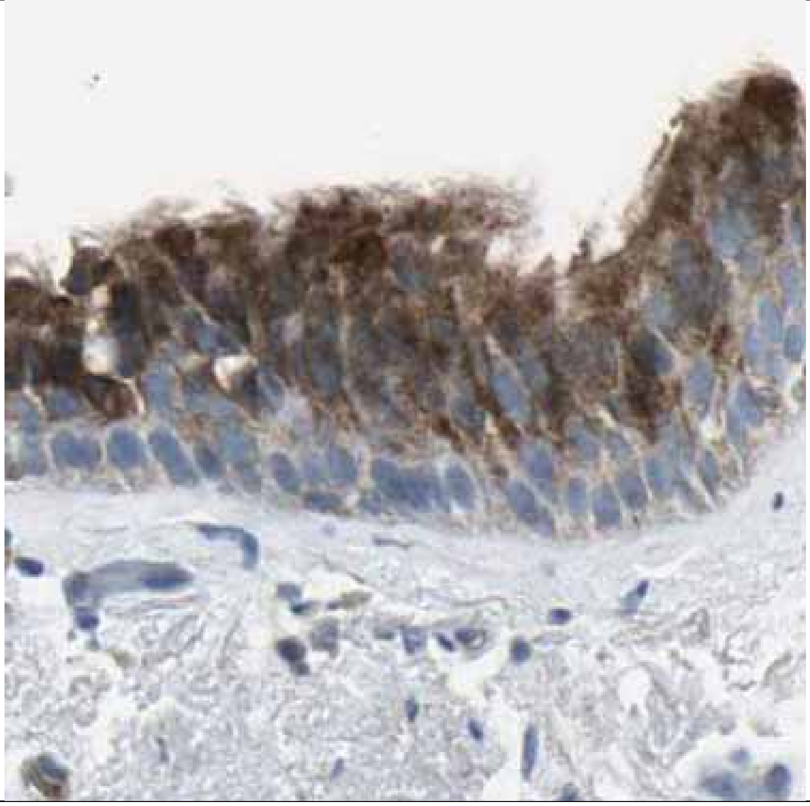	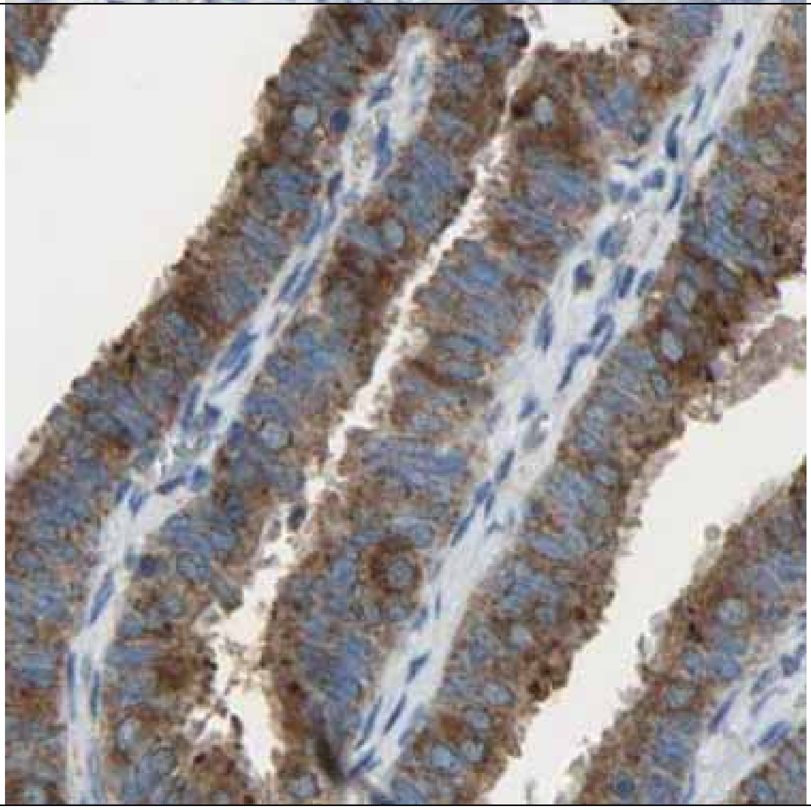	Staining is non-specific to ciliated cells or absent

GMPR (antibody HPA000904)<br><br>Category 1	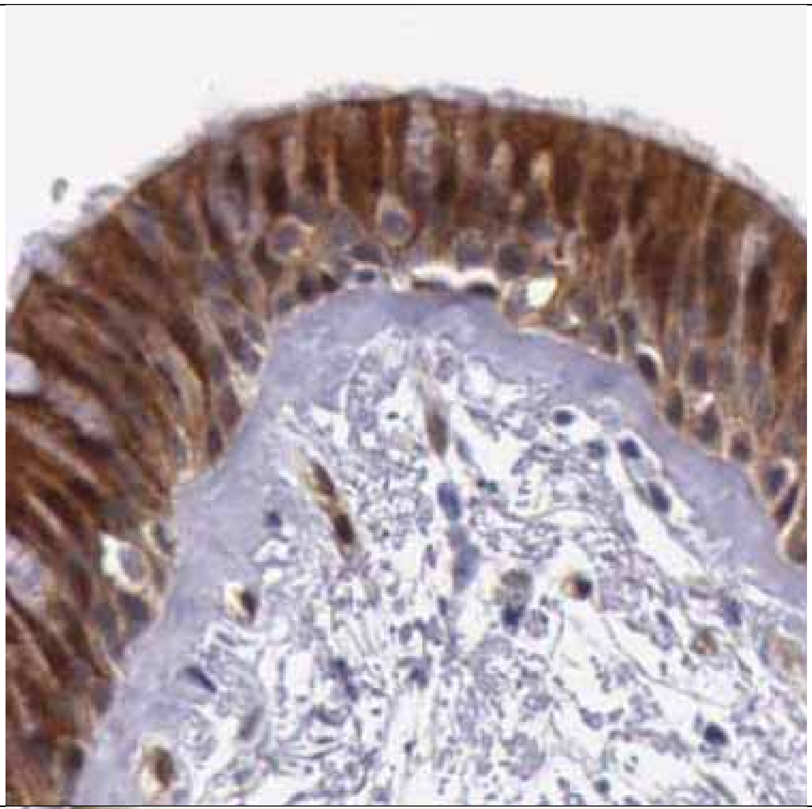	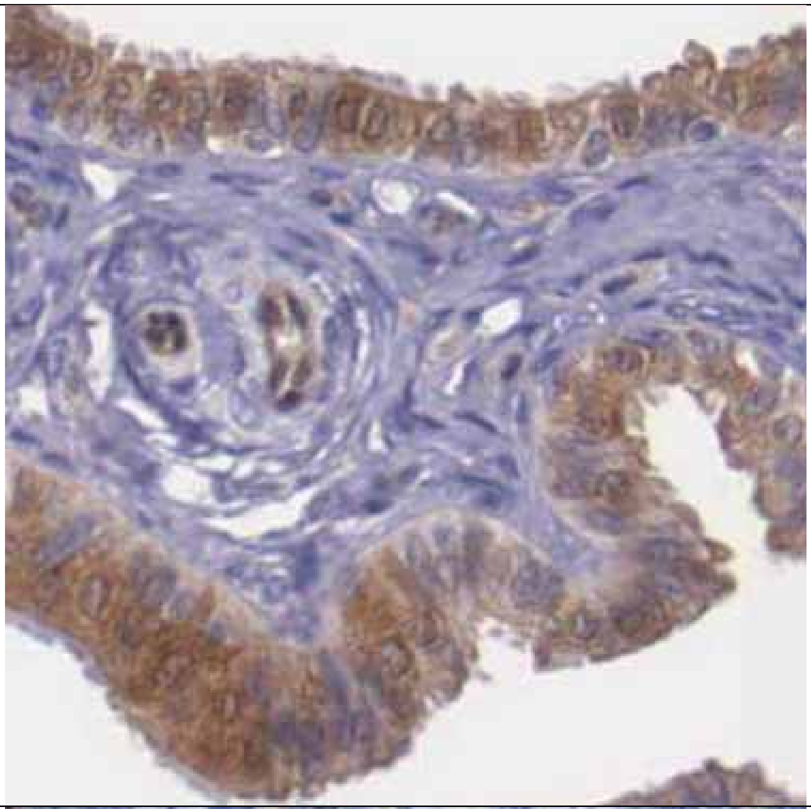	Staining is non-specific to ciliated cells or absent
HKDC1 (antibody HPA011956)<br><br>Category 1	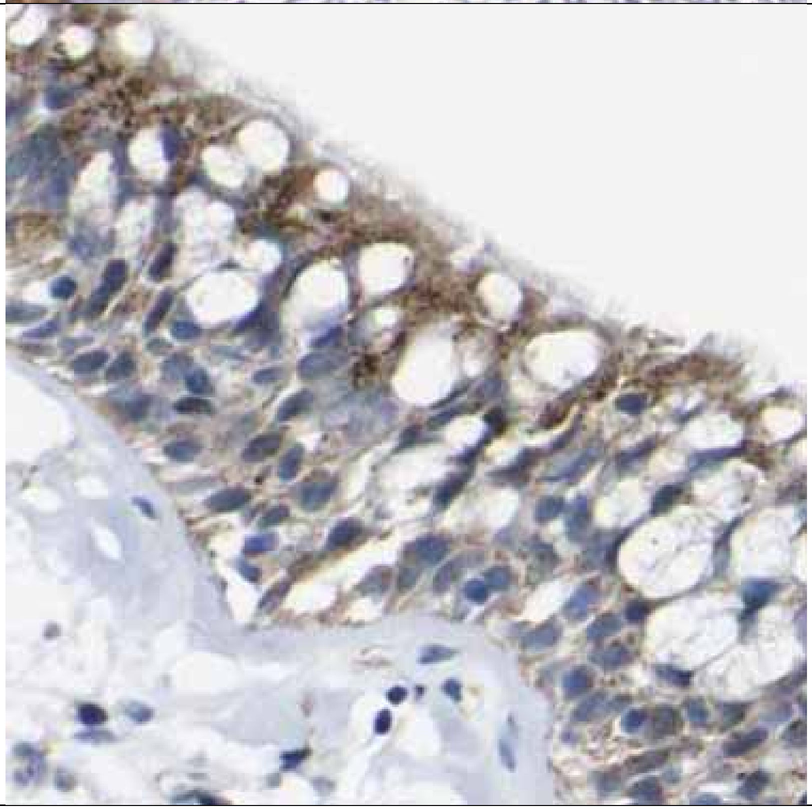	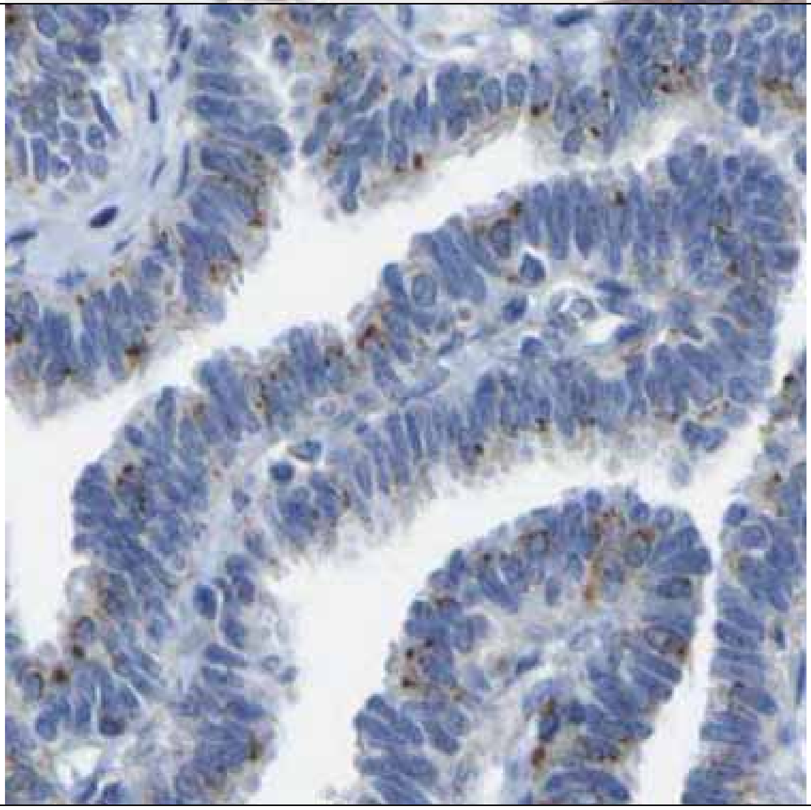	Staining is non-specific to ciliated cells or absent

IK (antibody HPA019754)<br><br>Category 1	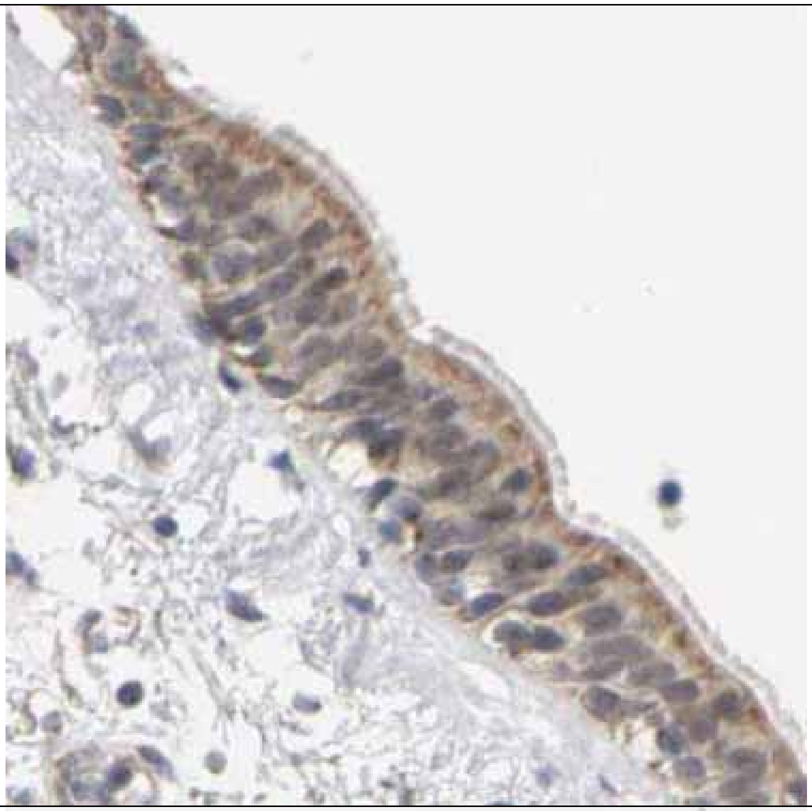	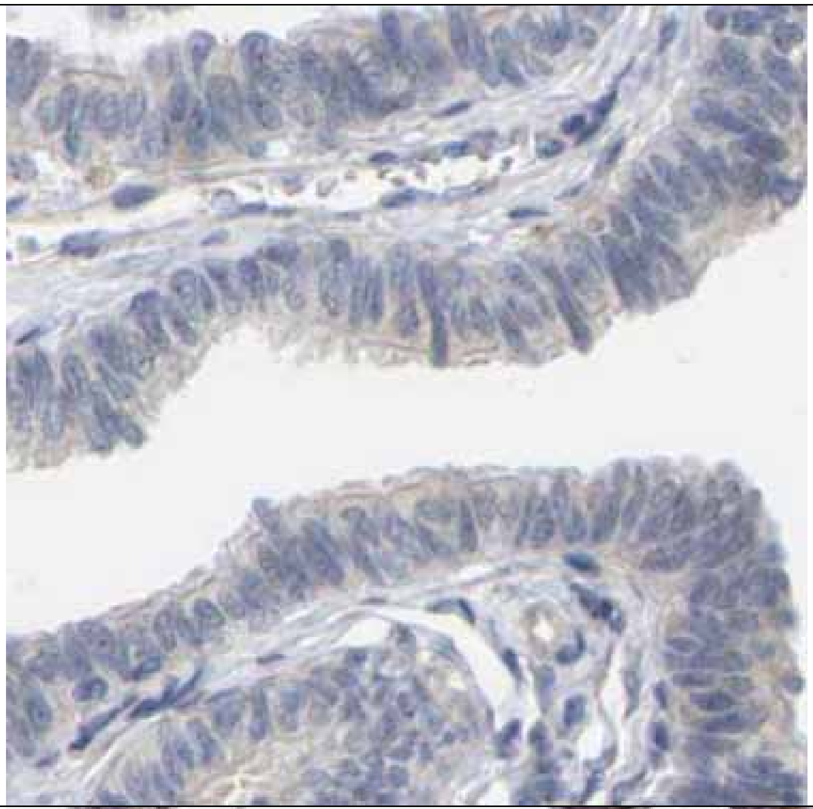	Staining is non-specific to ciliated cells or absent
IQCH (antibody HPA040845)<br><br>Category 1	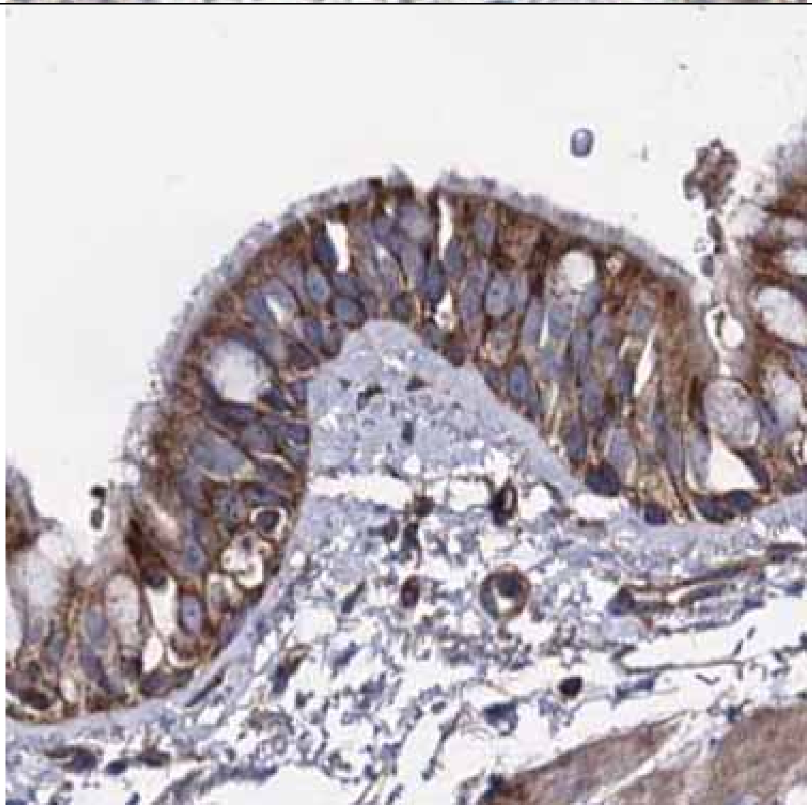	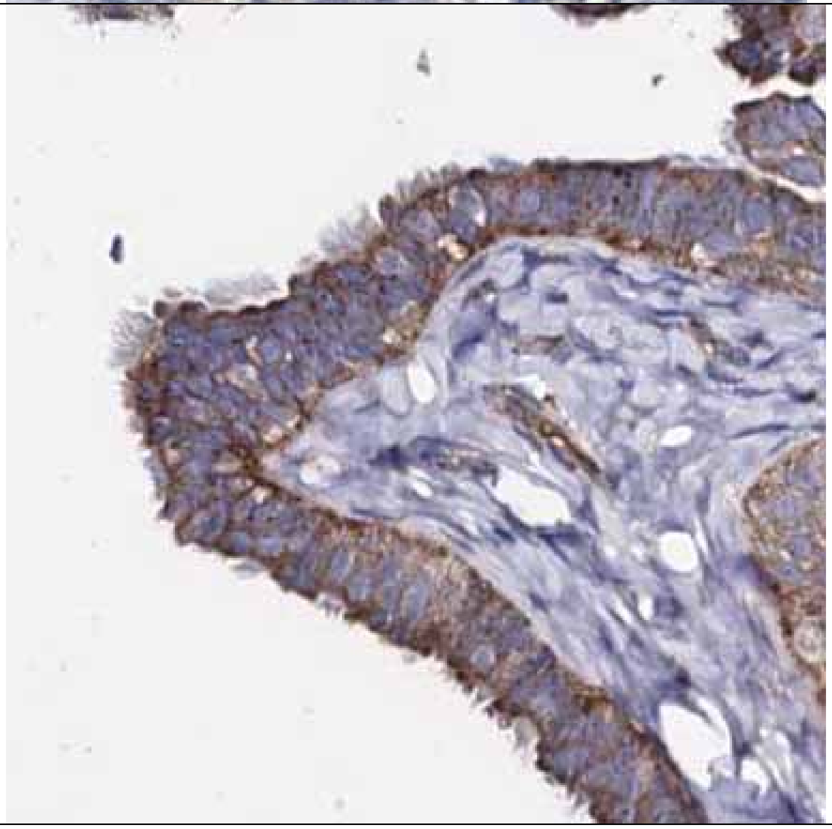	Staining is non-specific to ciliated cells or absent

KIF21A (antibody CAB022079)<br><br>Category 1	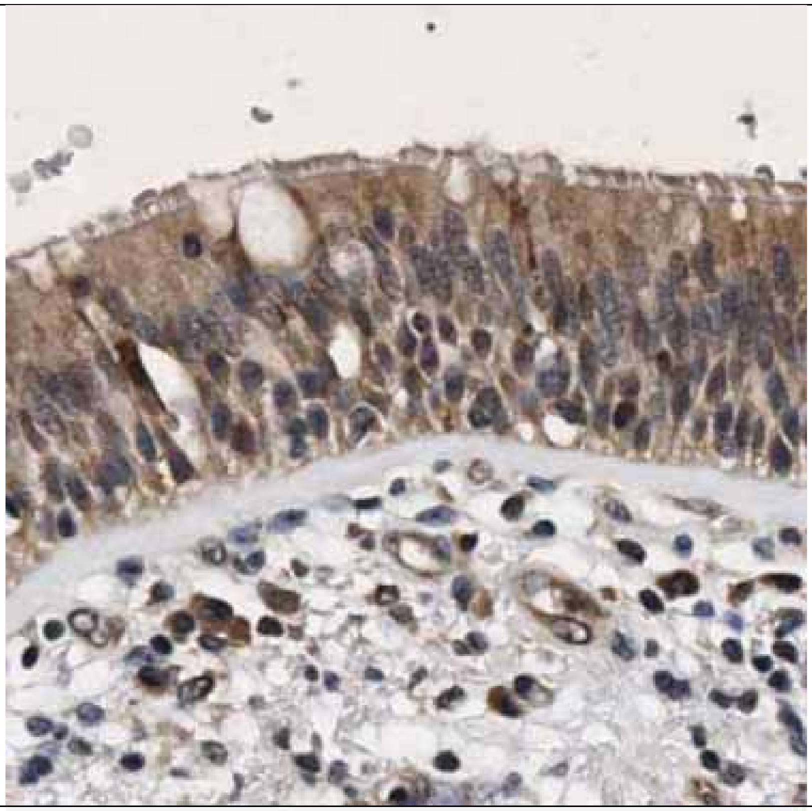	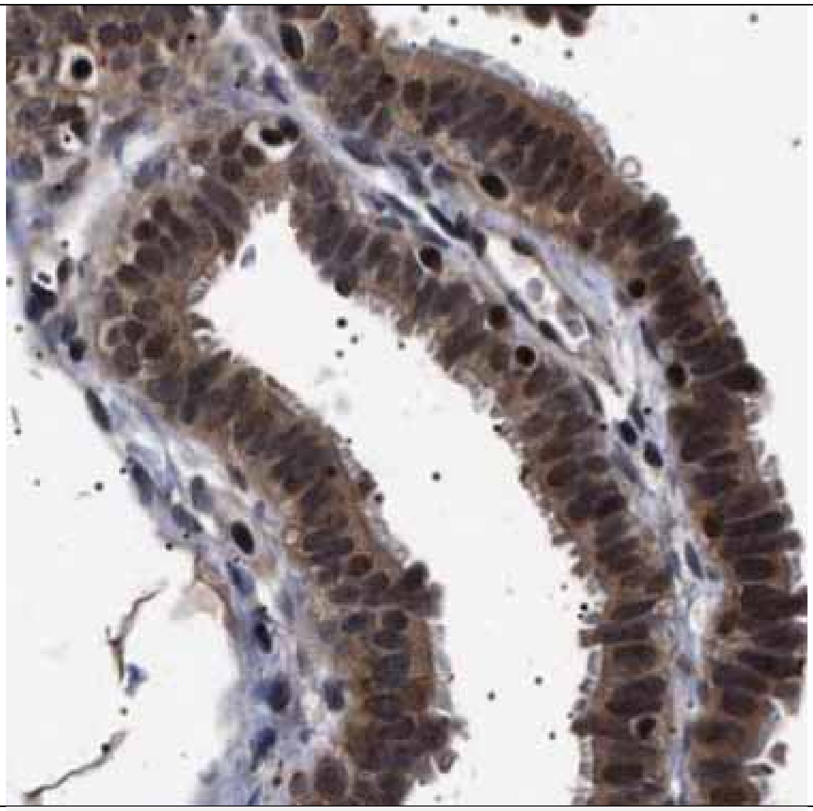	Staining is non-specific to ciliated cells or absent
LRRC46 (antibody HPA021882)<br><br>Category 1	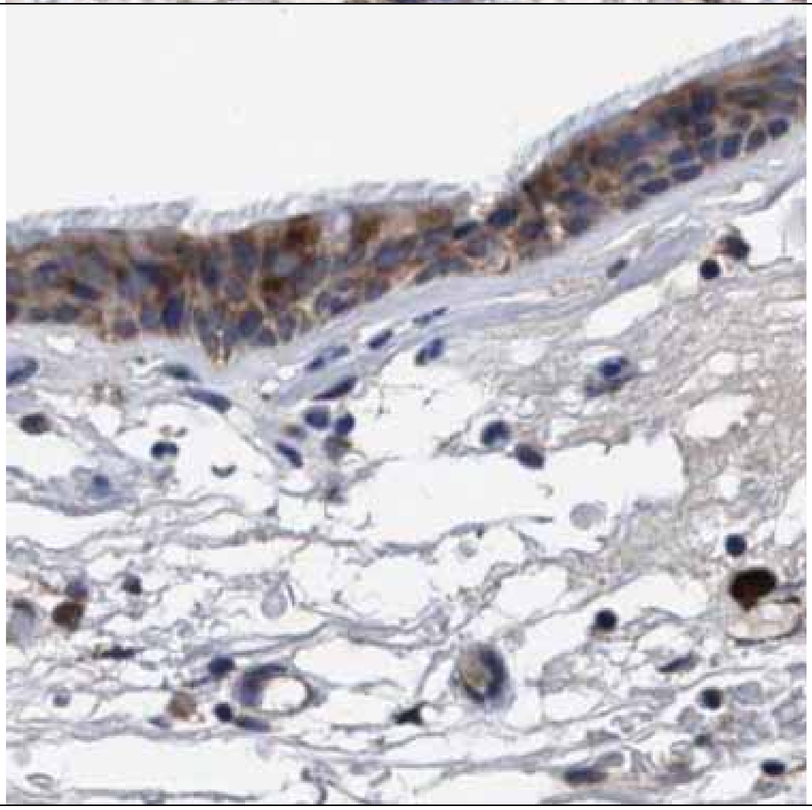	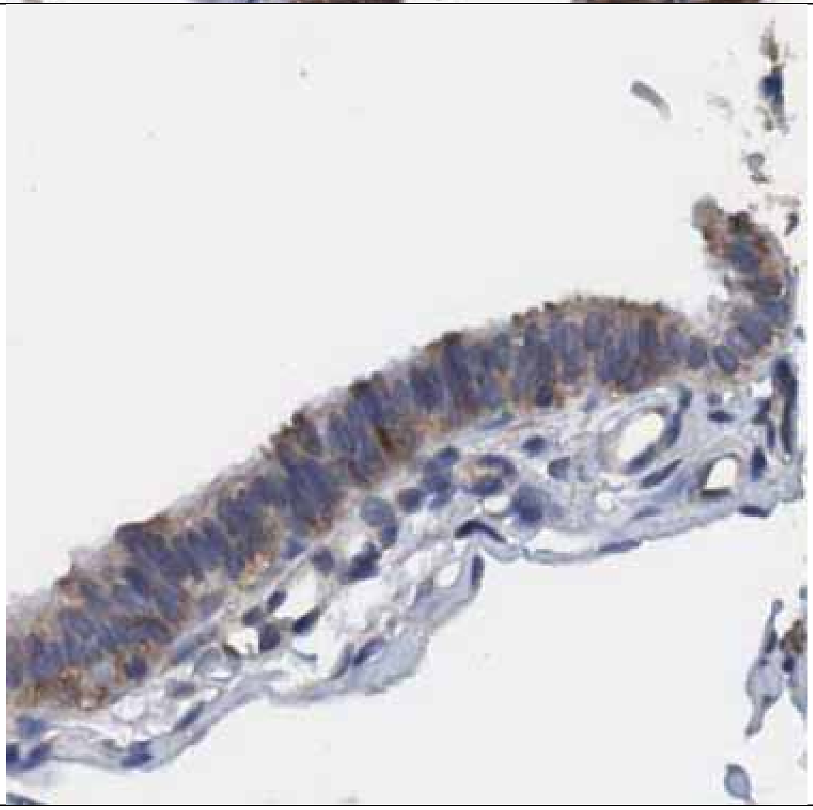	Staining is non-specific to ciliated cells or absent

MYB (antibody CAB017704)<br><br>Category 1	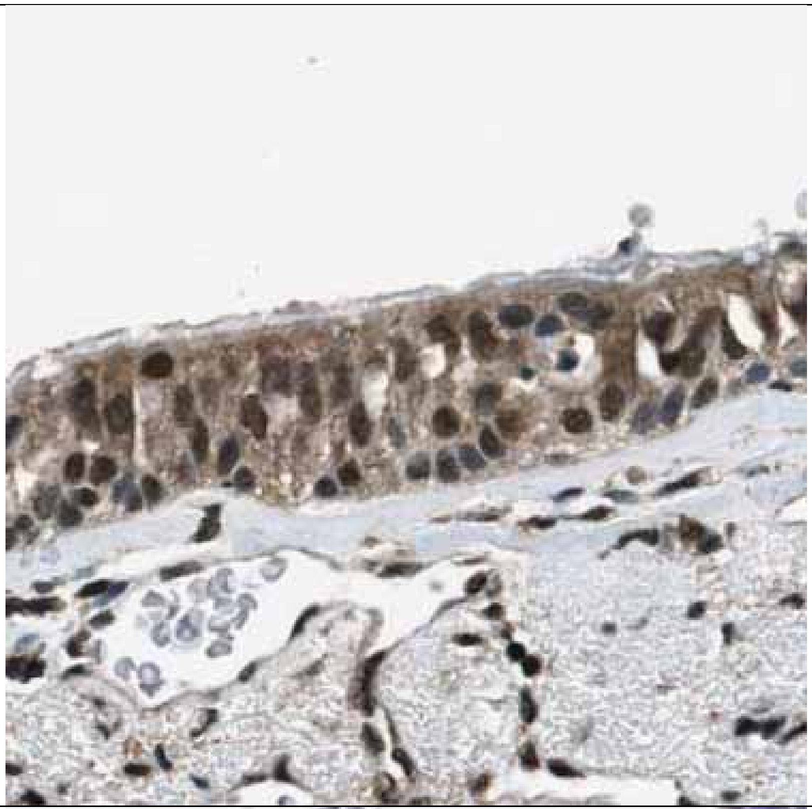	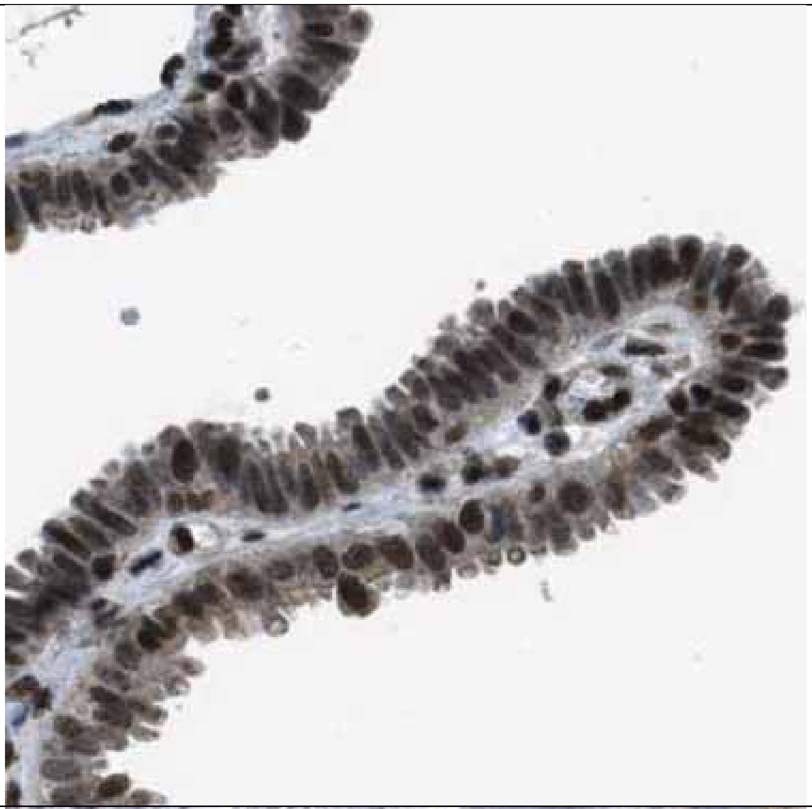	Staining is non-specific to ciliated cells or absent
NME7 (antibody HPA038014)<br><br>Category 1	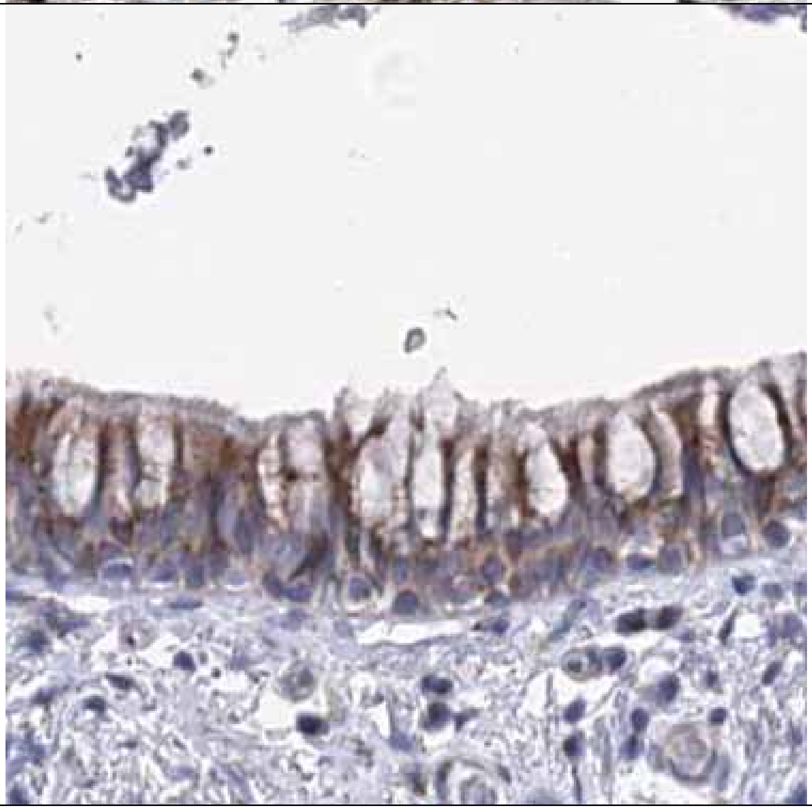	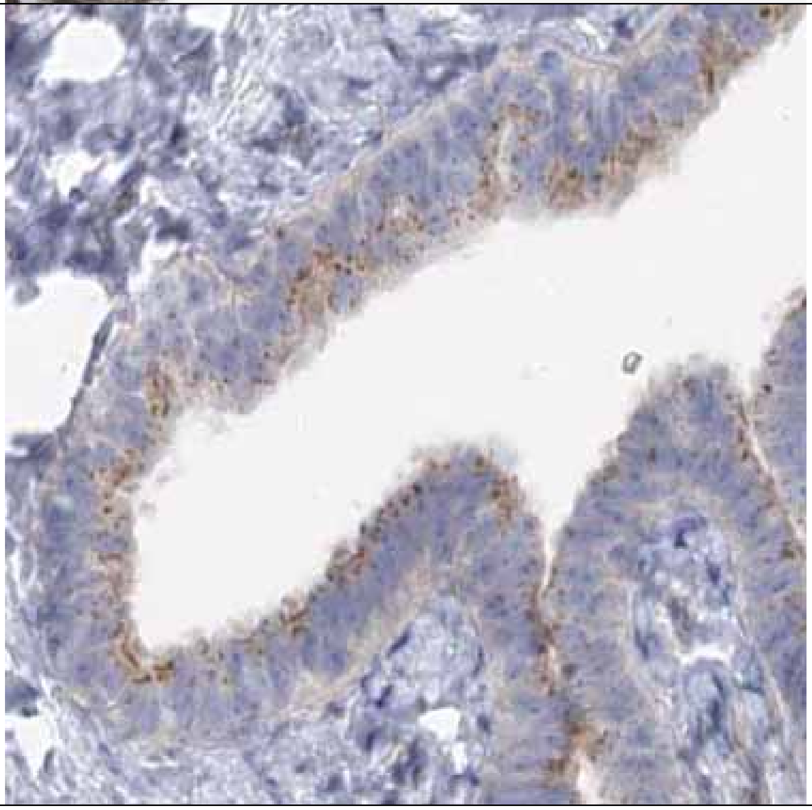	Staining is non-specific to ciliated cells or absent

NSUN7 (antibody HPA020653)<br><br>Category 1	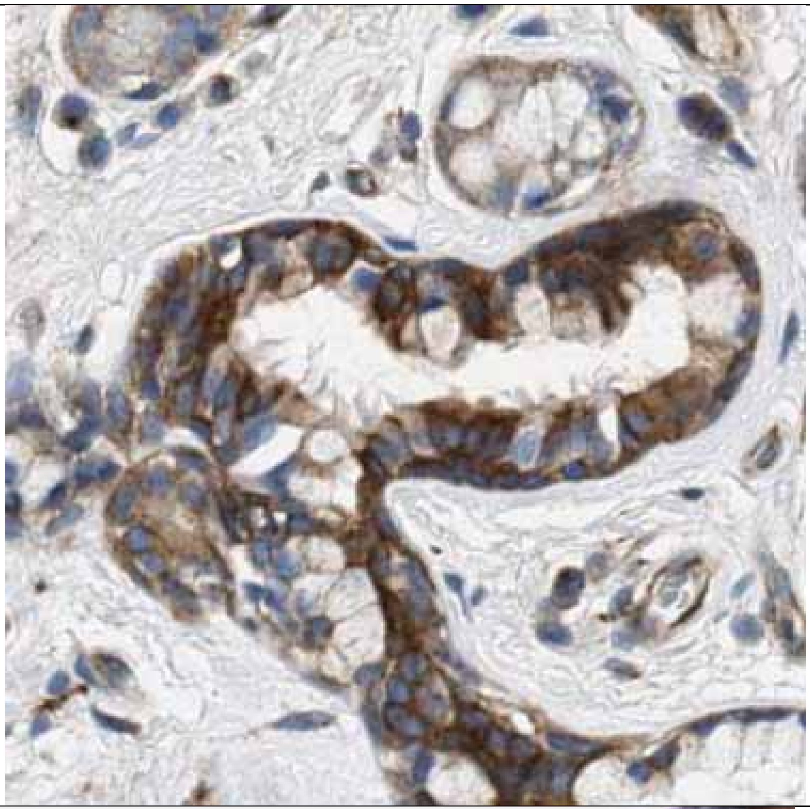	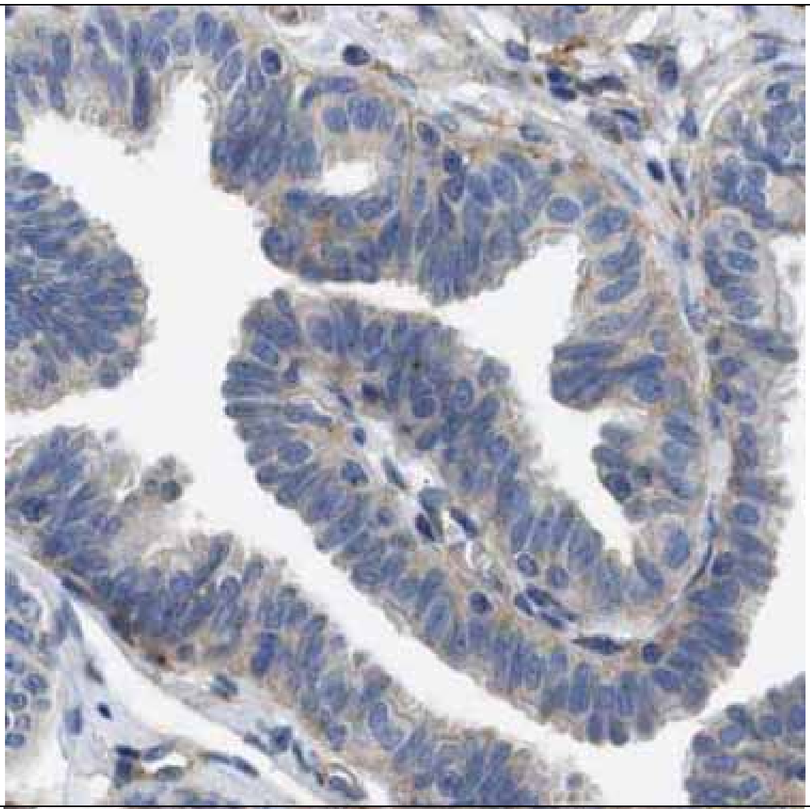	Staining is non-specific to ciliated cells or absent
PECR (antibody HPA021593)<br><br>Category 1	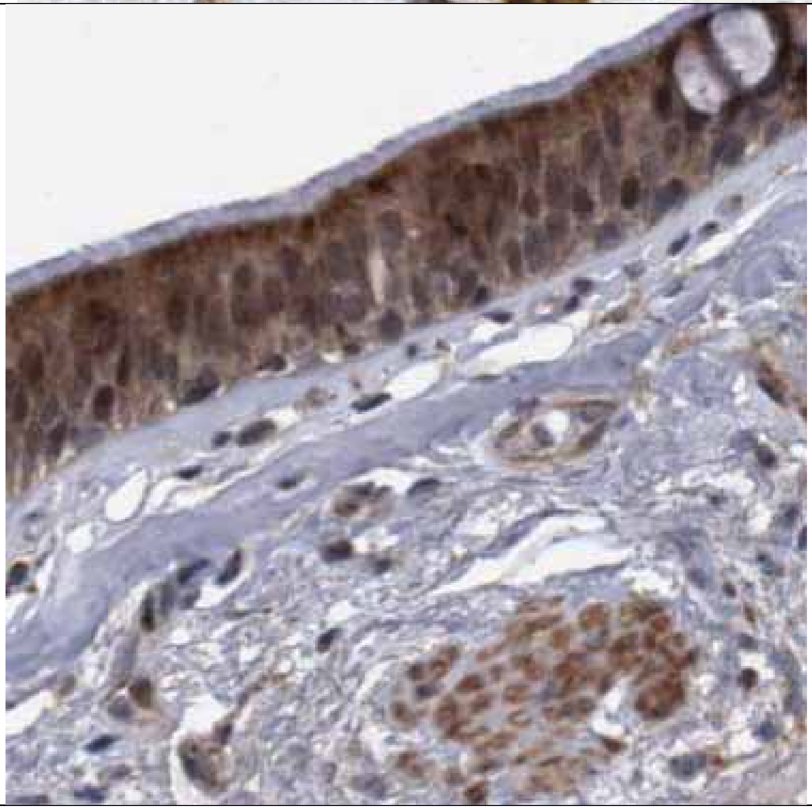	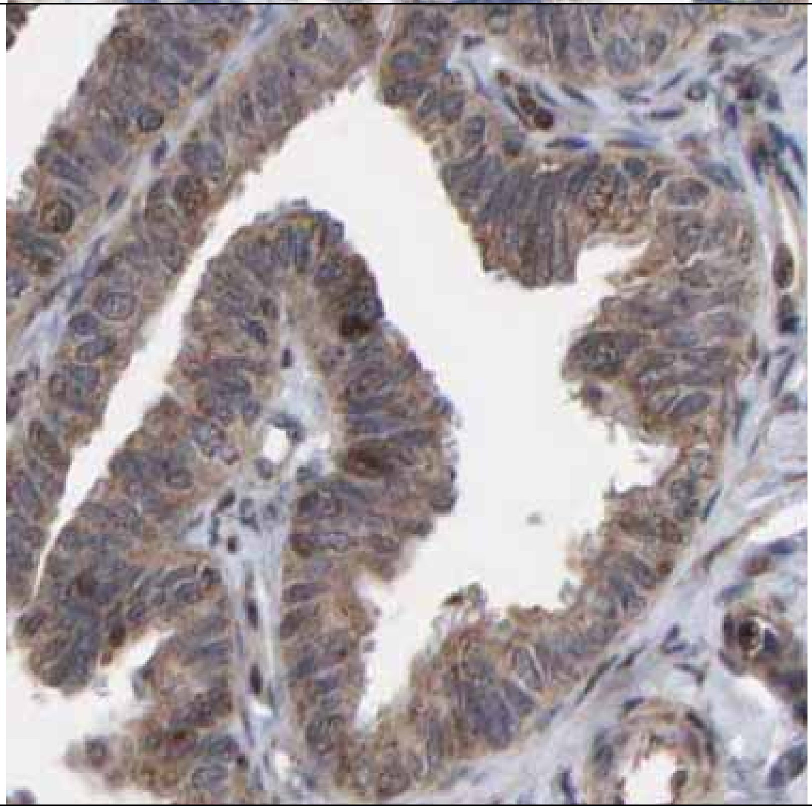	Staining is non-specific to ciliated cells or absent

PTPRT (antibody HPA017336)<br><br>Category 1	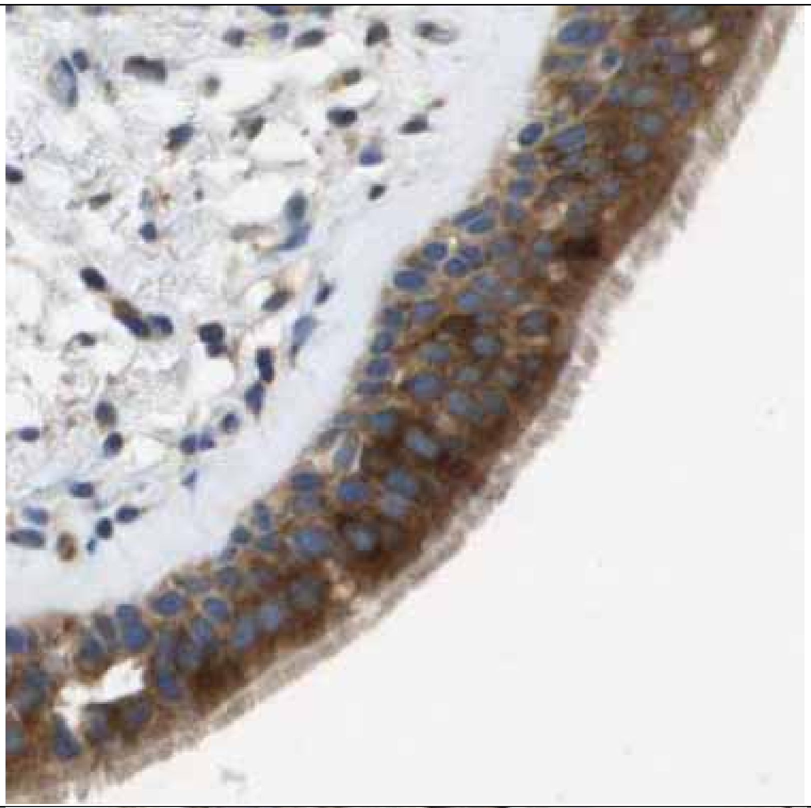	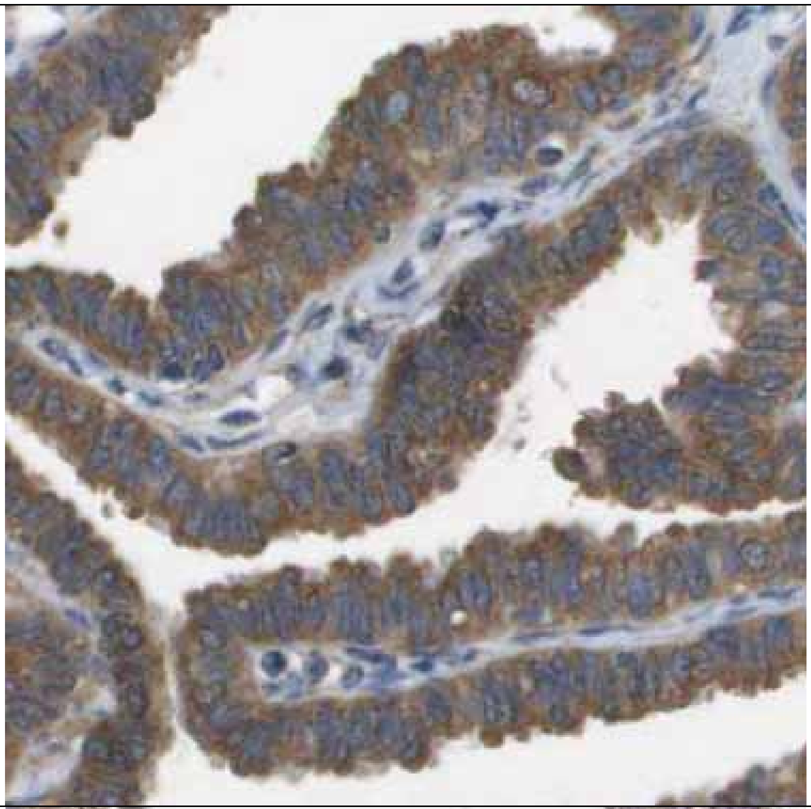	Staining is non-specific to ciliated cells or absent
RAGE (antibody HPA027282)<br><br>Category 1	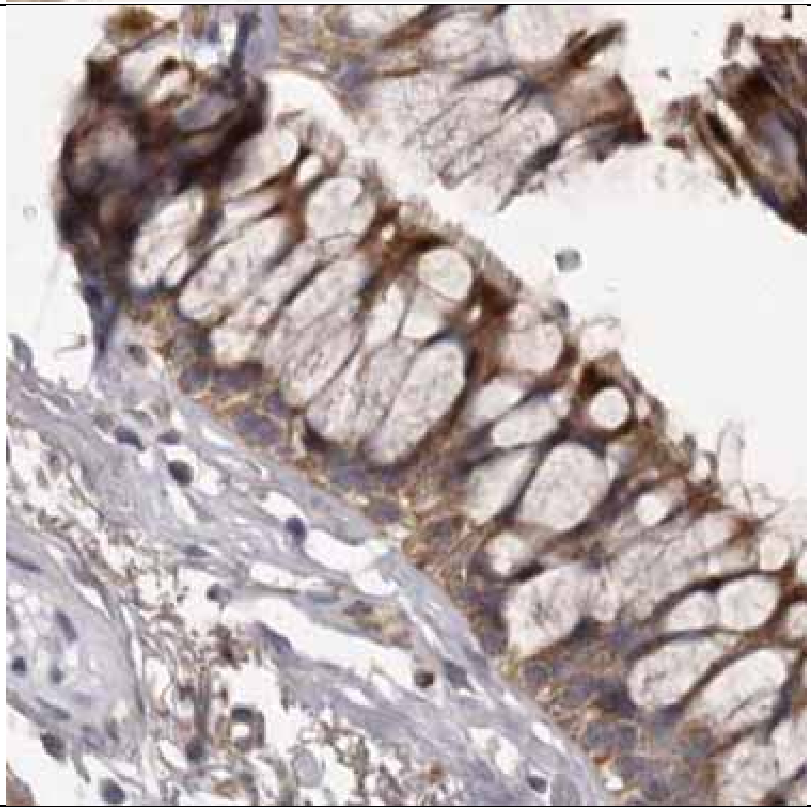	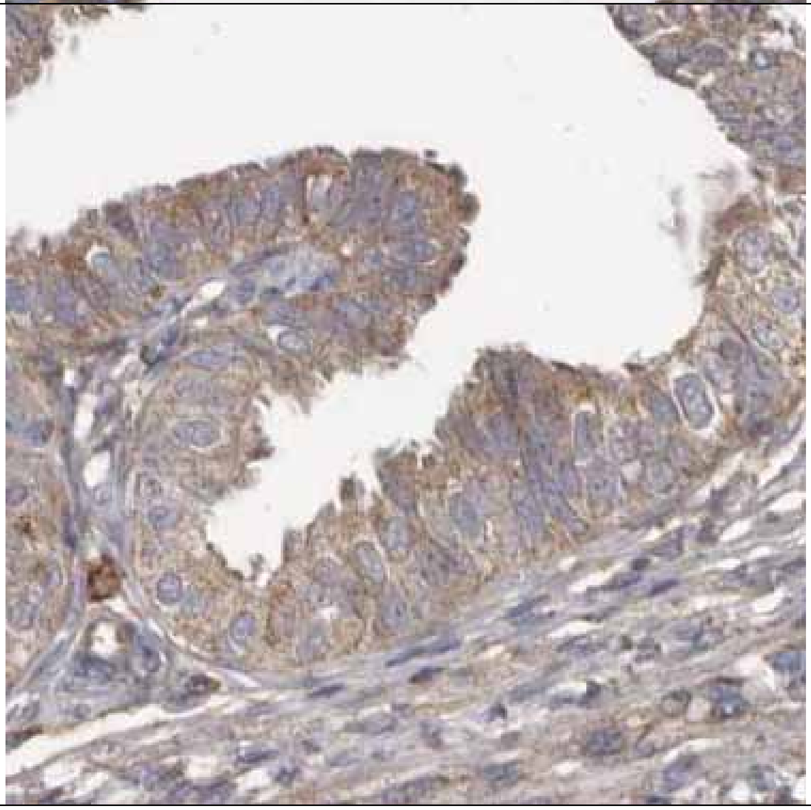	Staining is non-specific to ciliated cells or absent

RIBC1 (antibody HPA021613)<br><br>Category 1	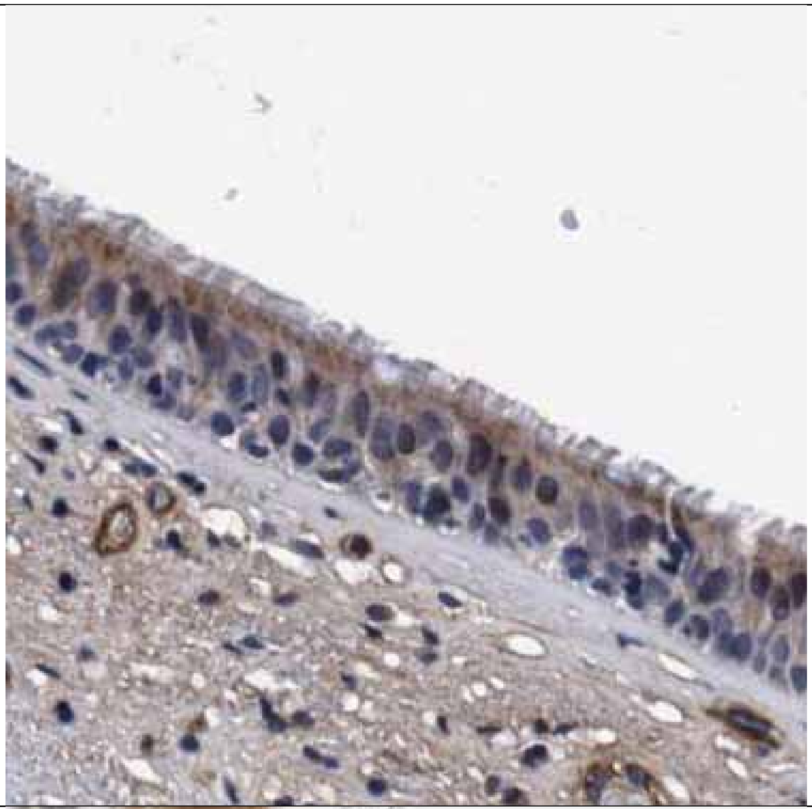	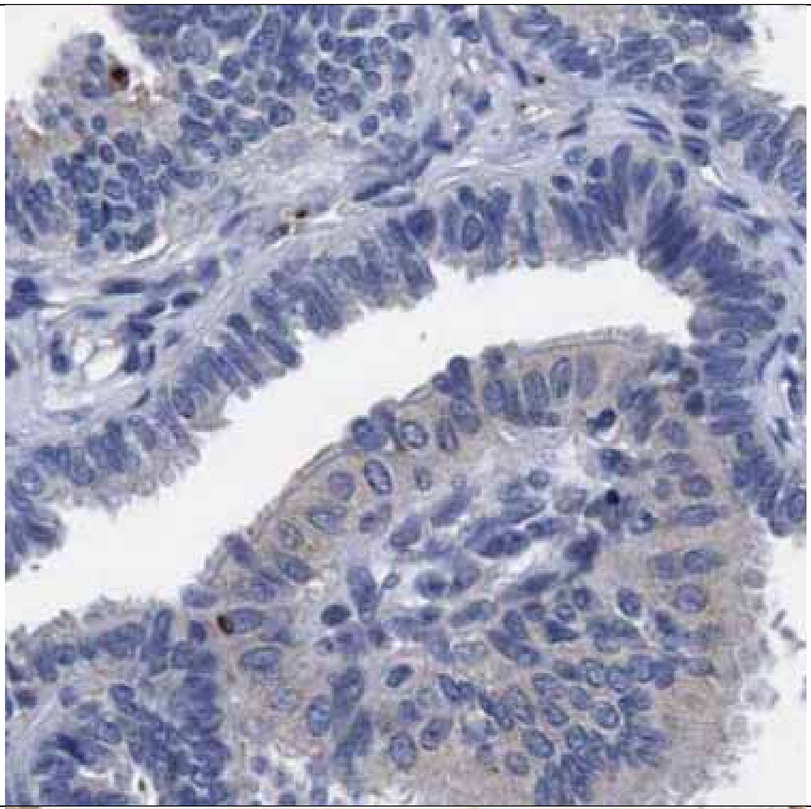	Staining is non-specific to ciliated cells or absent
SERPINI2 (antibody HPA030613)<br><br>Category 1	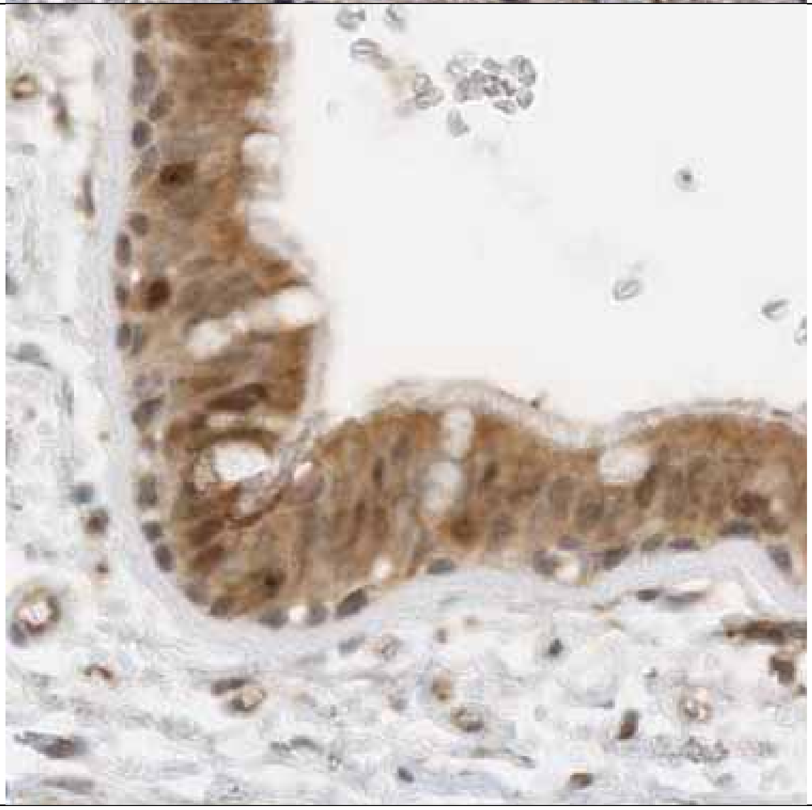	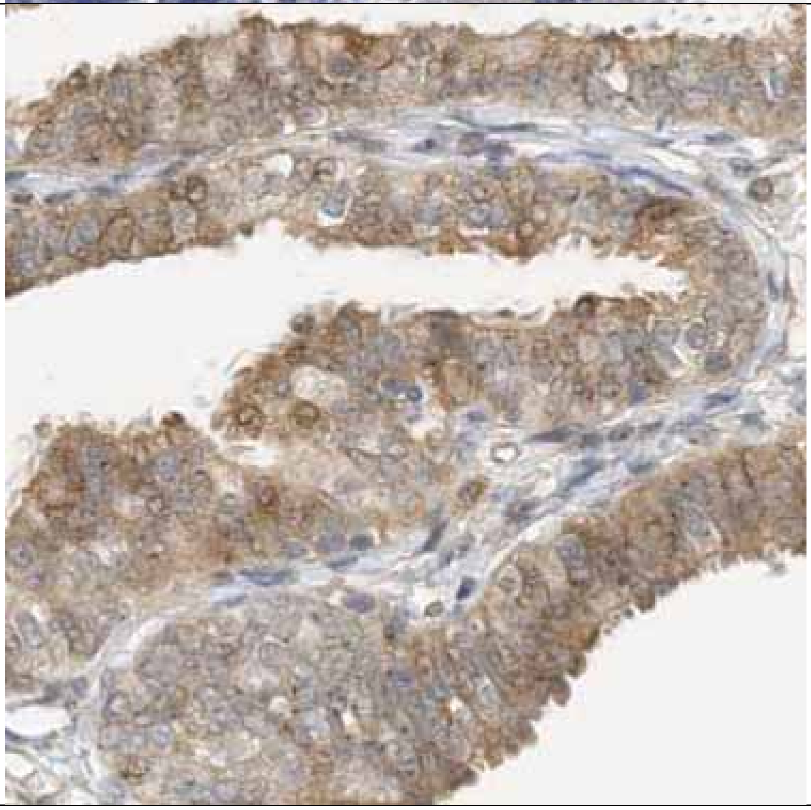	Staining is non-specific to ciliated cells or absent

TMEM190 (antibody HPA026899)<br><br>Category 1	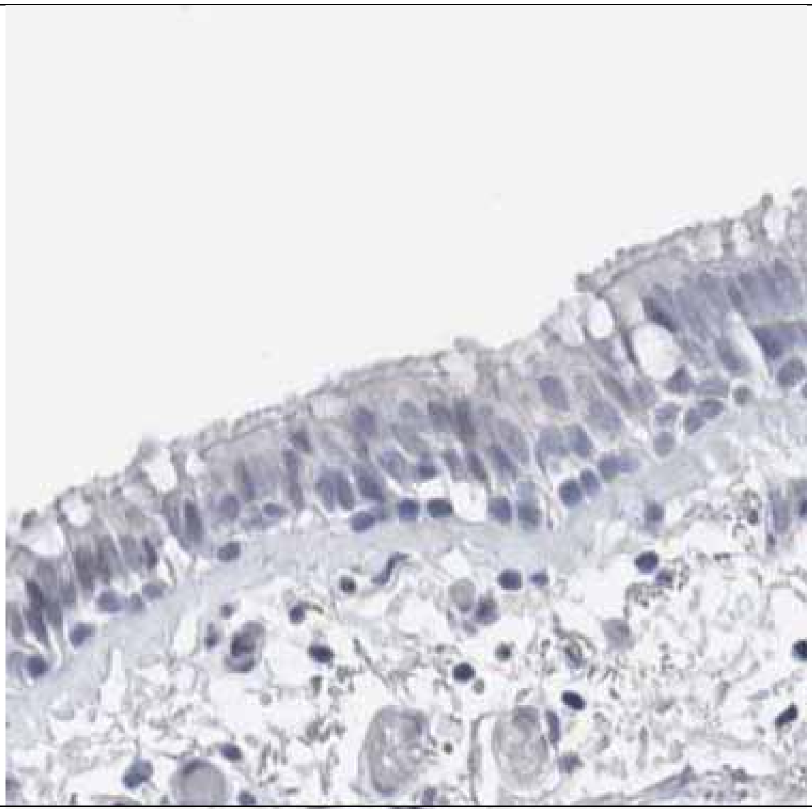	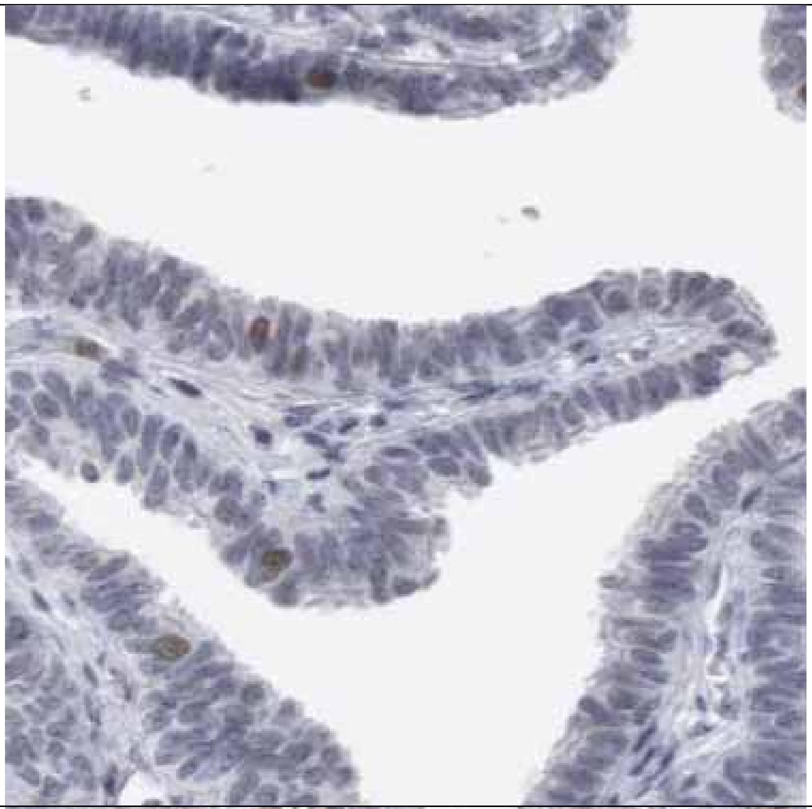	Staining is non-specific to ciliated cells or absent
TTC21A (antibody HPA035511)<br><br>Category 1	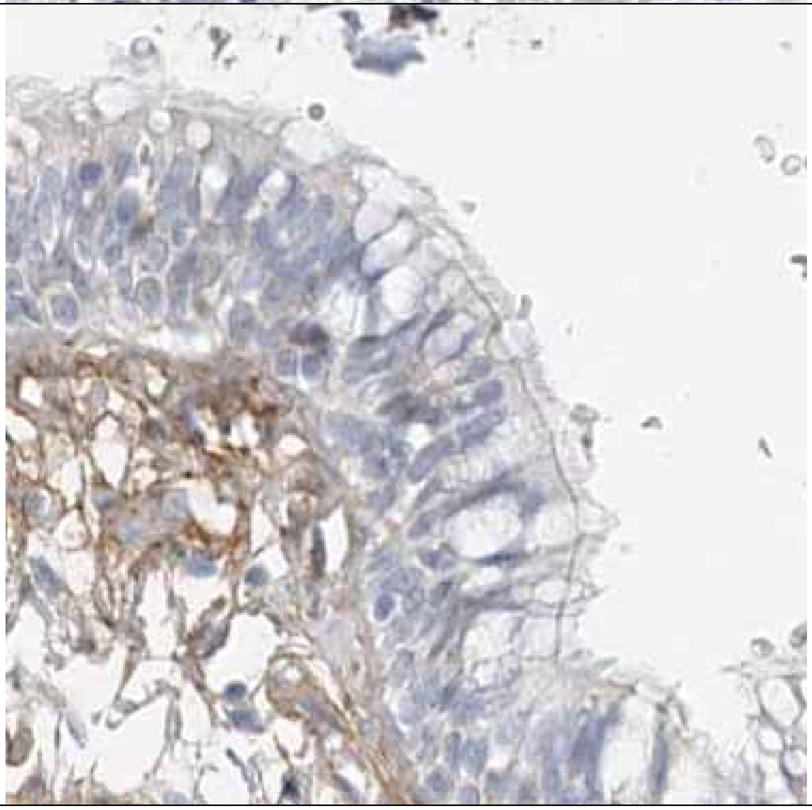	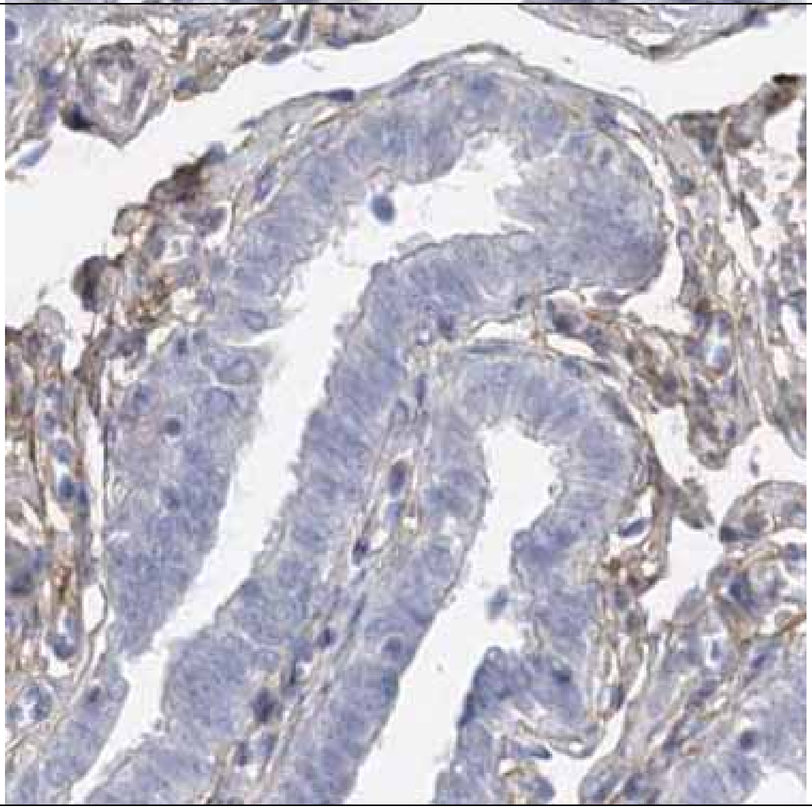	Staining is non-specific to ciliated cells or absent

WDR63 (antibody HPA038066)<br><br>Category 1	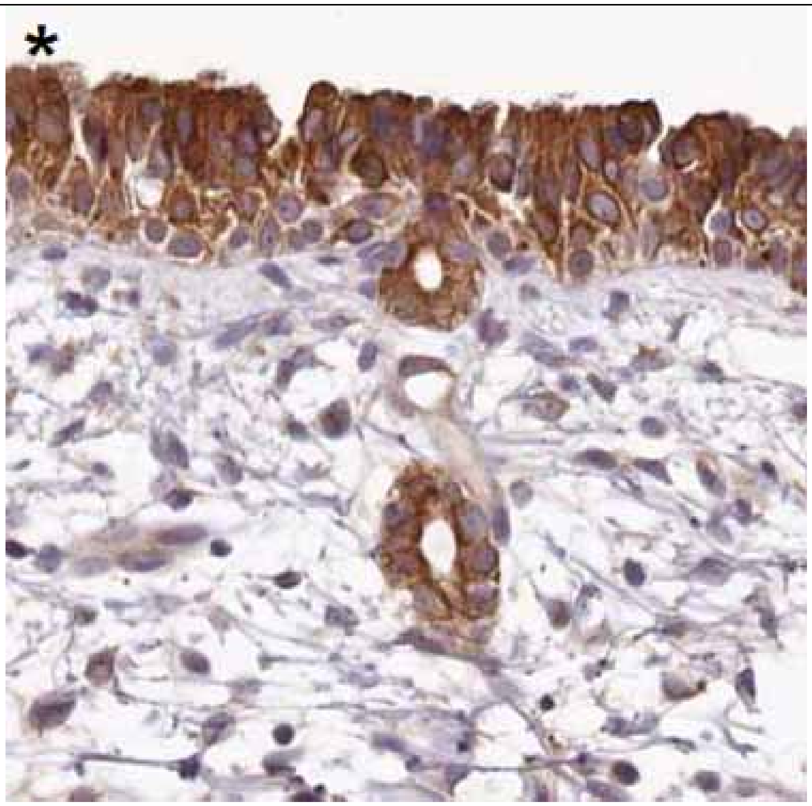	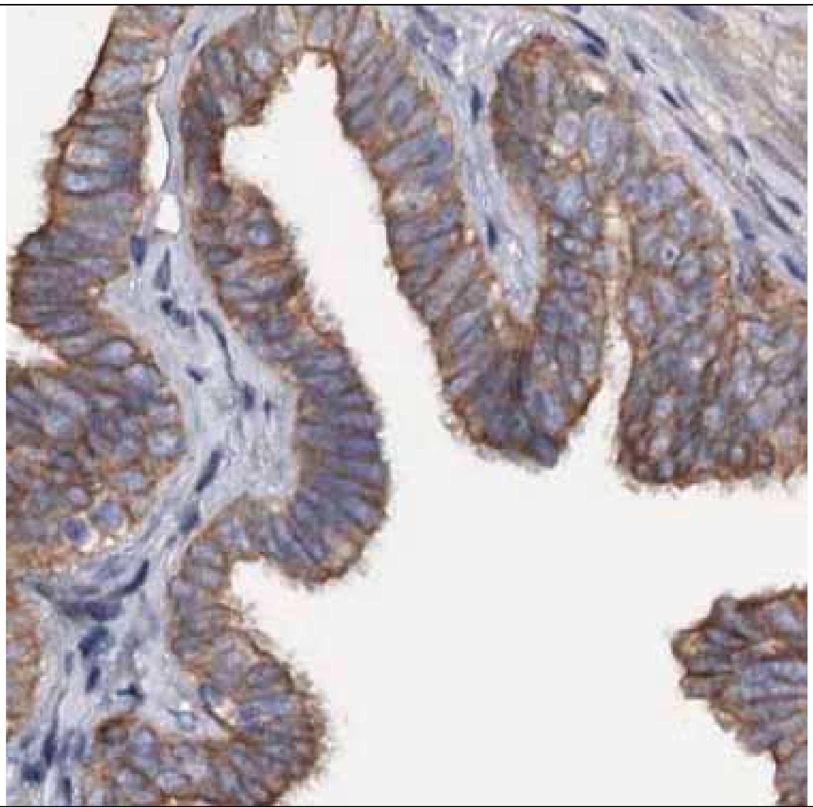	Staining is non-specific to ciliated cells or absent
ZMYND10 (antibody HPA035255)<br><br>Category 1	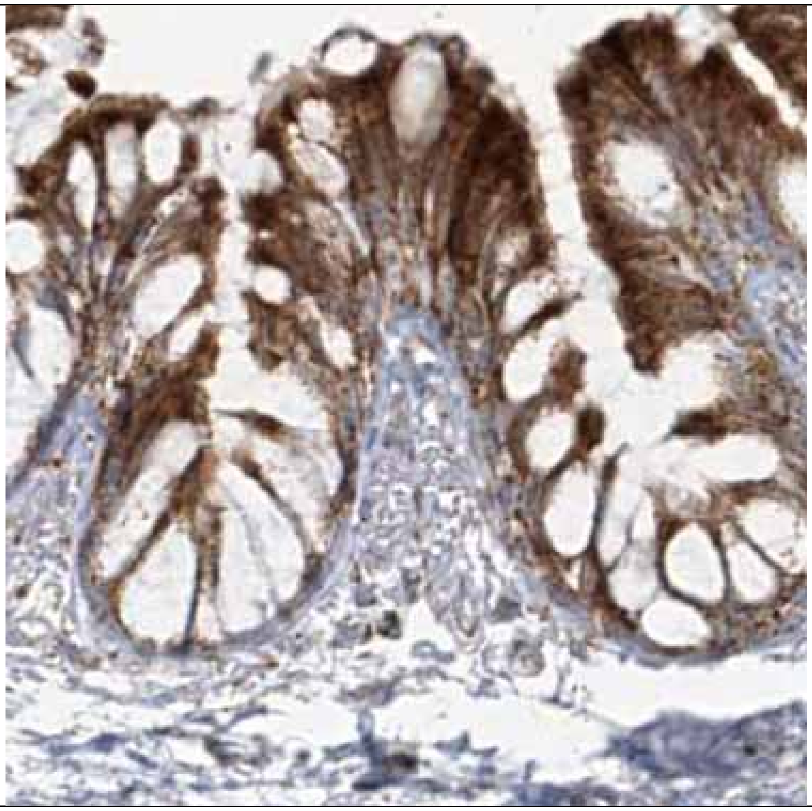	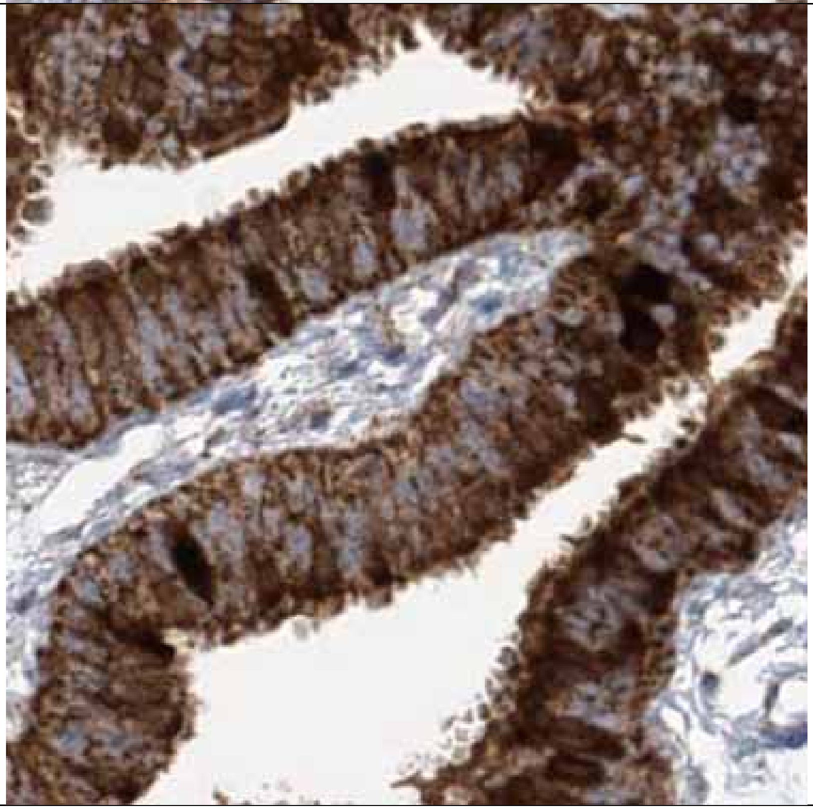	Staining is non-specific to ciliated cells or absent

Category 2

(genes with weak evidence

for ciliary function from the *literature*)

Coverage: 34 proteins were available in Protein Atlas
from the total of 60 proteins in the category

Protein	Airways	Fallopian tubes	Summary
C1orf87 (antibody HPA031368)<br><br>Category 2	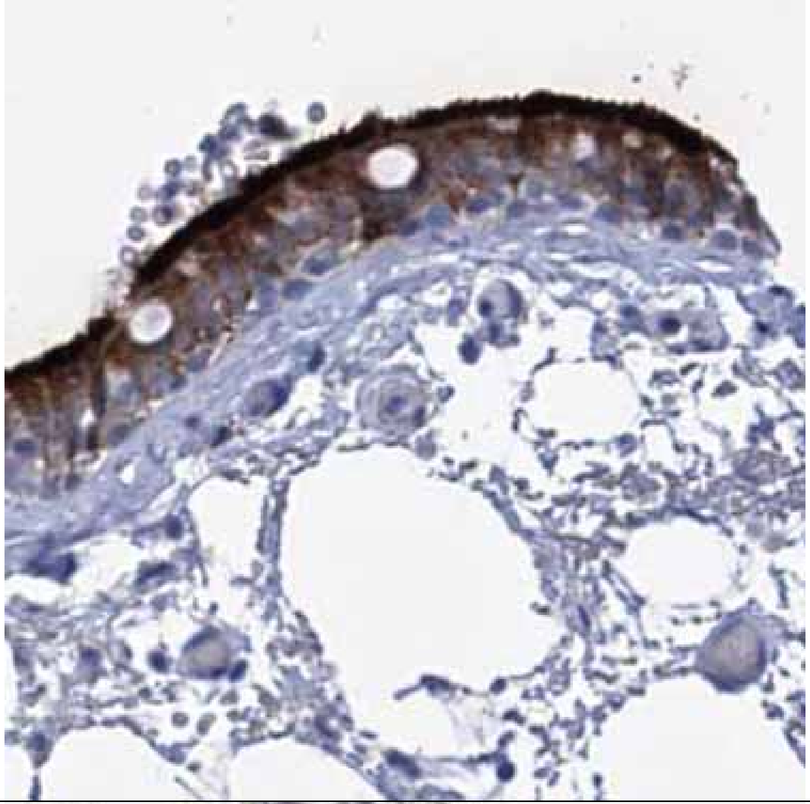	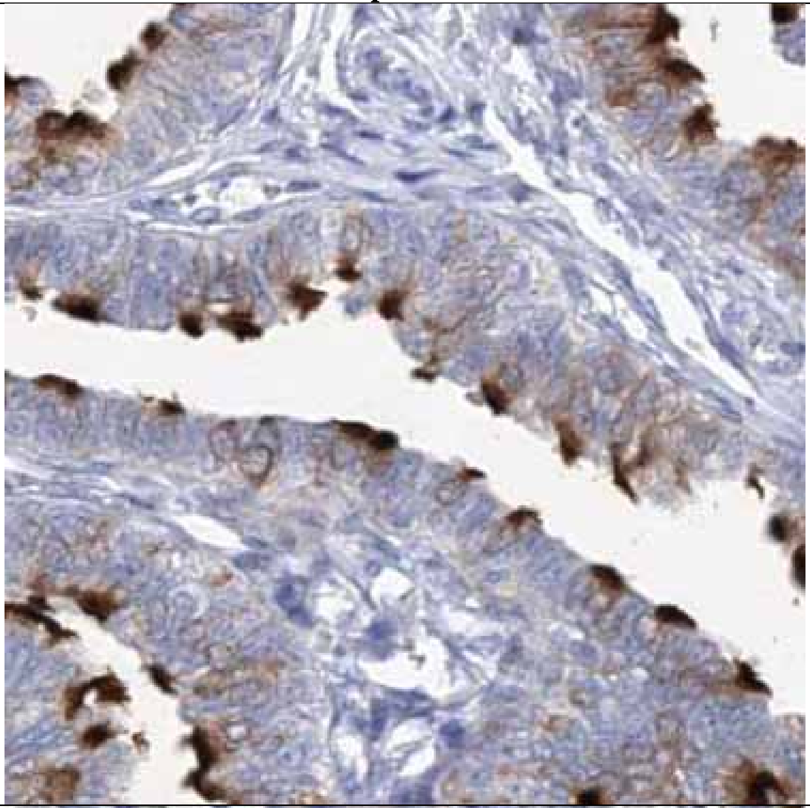	Staining is specific to *cilia*
C1orf222 (antibody HPA029274)<br><br>Category 2	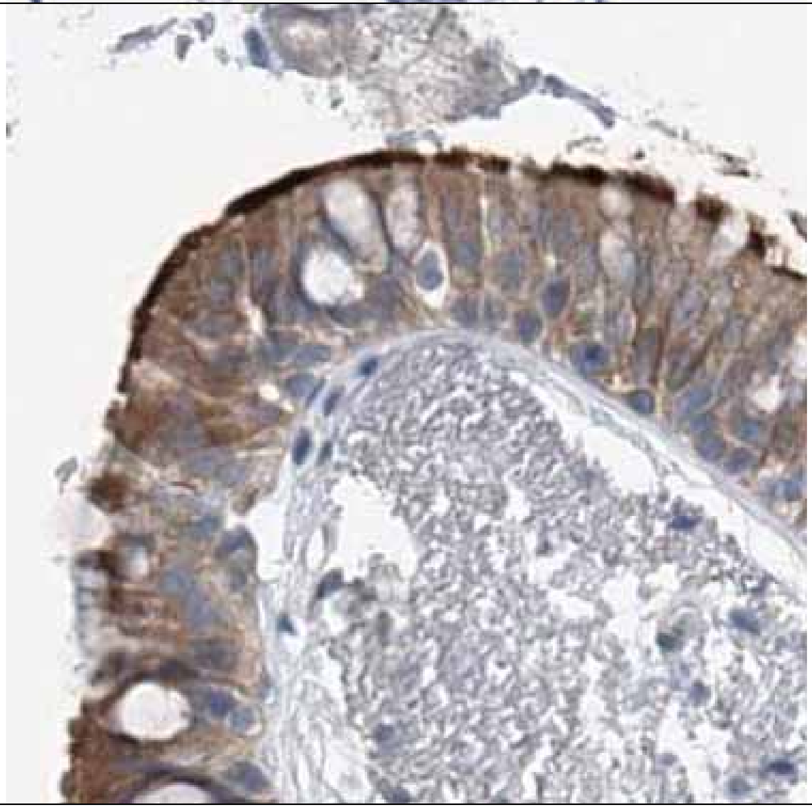	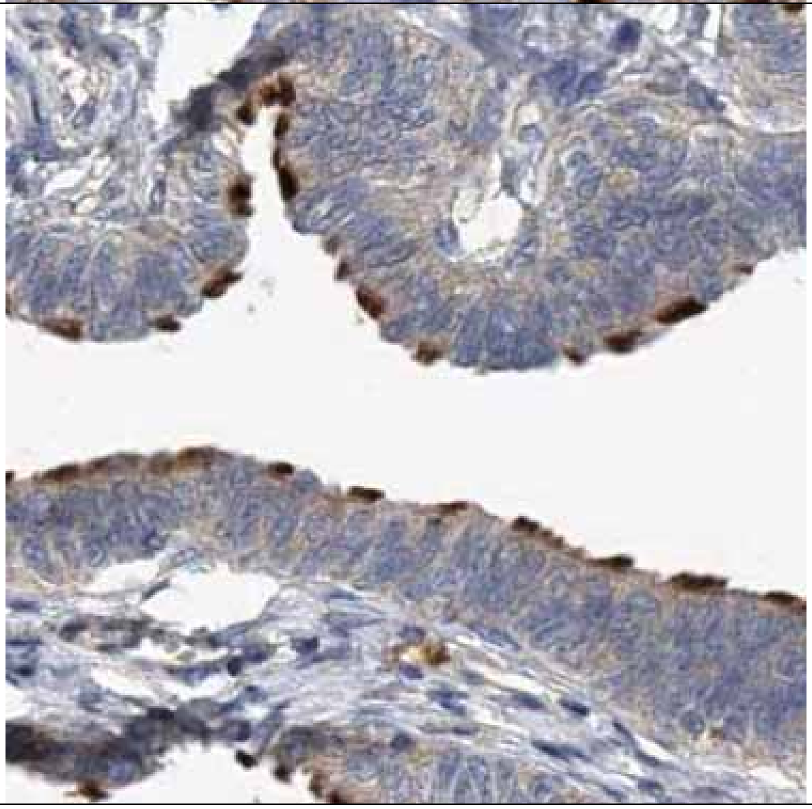	Staining is specific to *cilia*

C11orf66 (antibody HPA039068)<br><br>Category 2	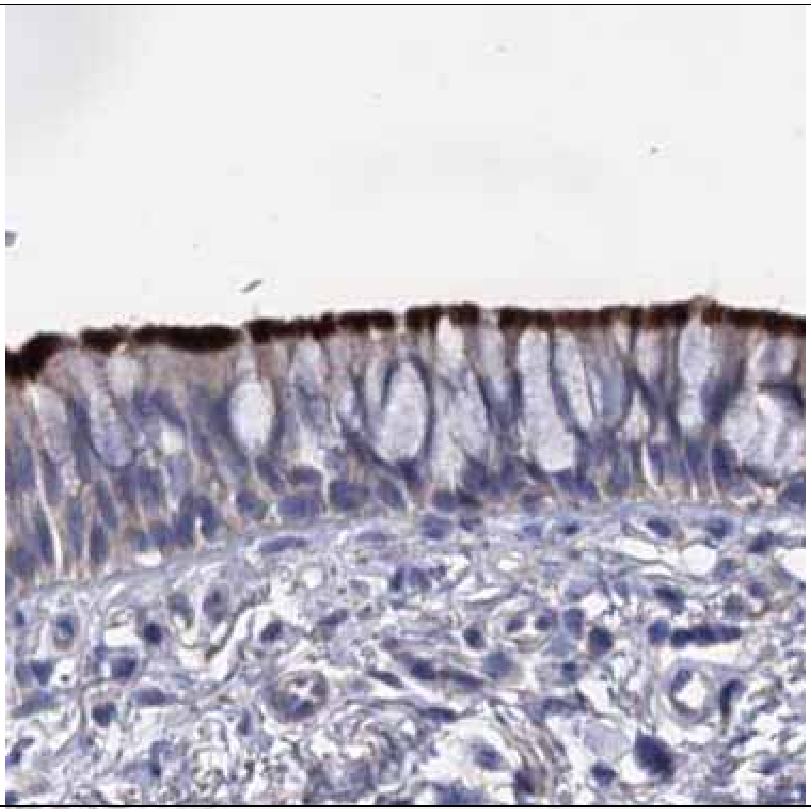	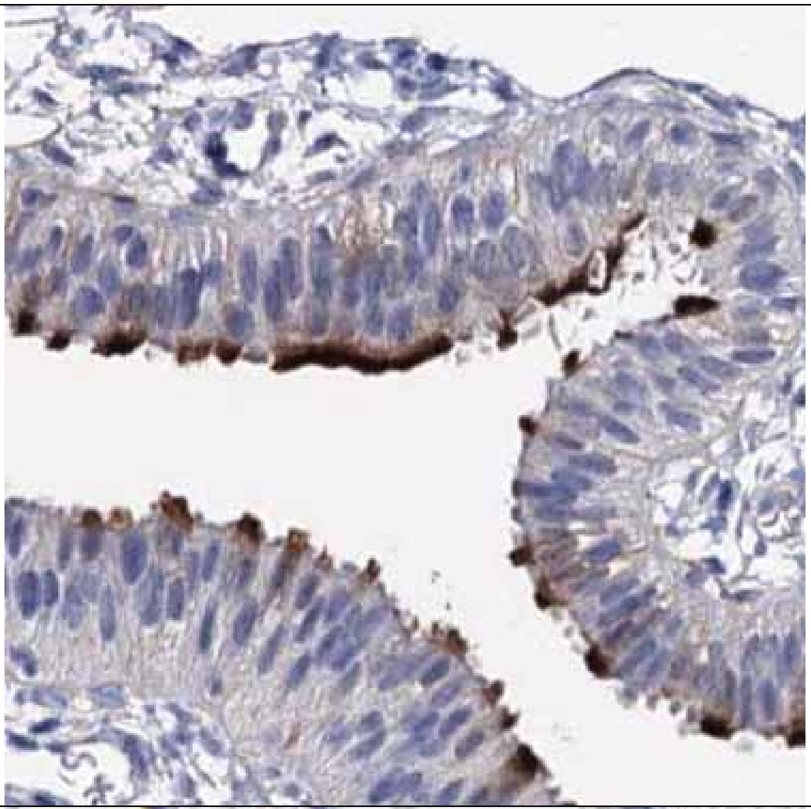	Staining is specific to *cilia*
C14orf179 (antibody HPA003438)<br><br>Category 2	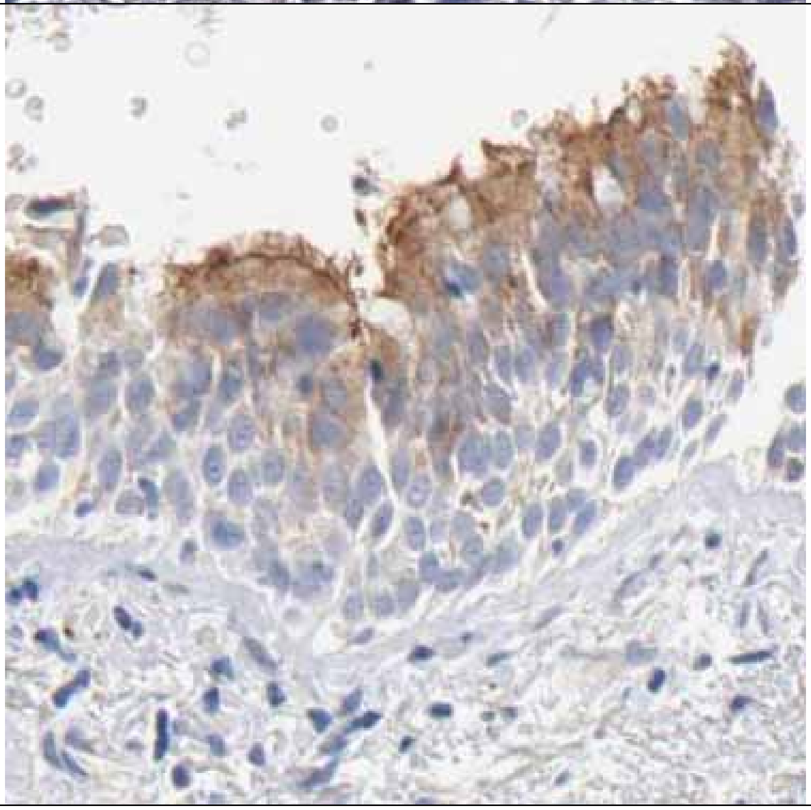	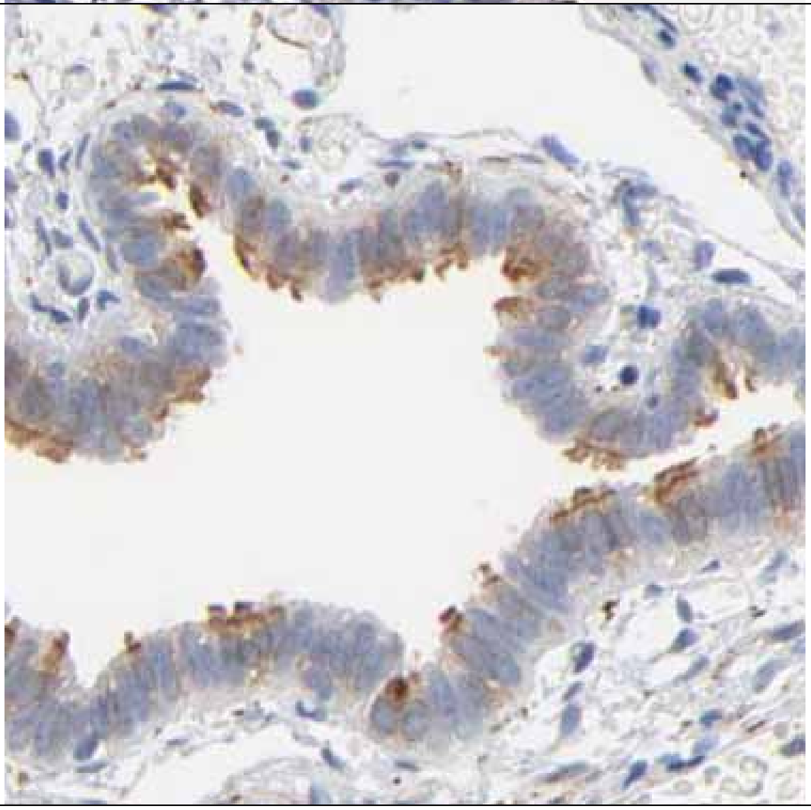	Staining is specific to *cilia*

C9orf9 (antibody HPA022243)<br><br>Category 2	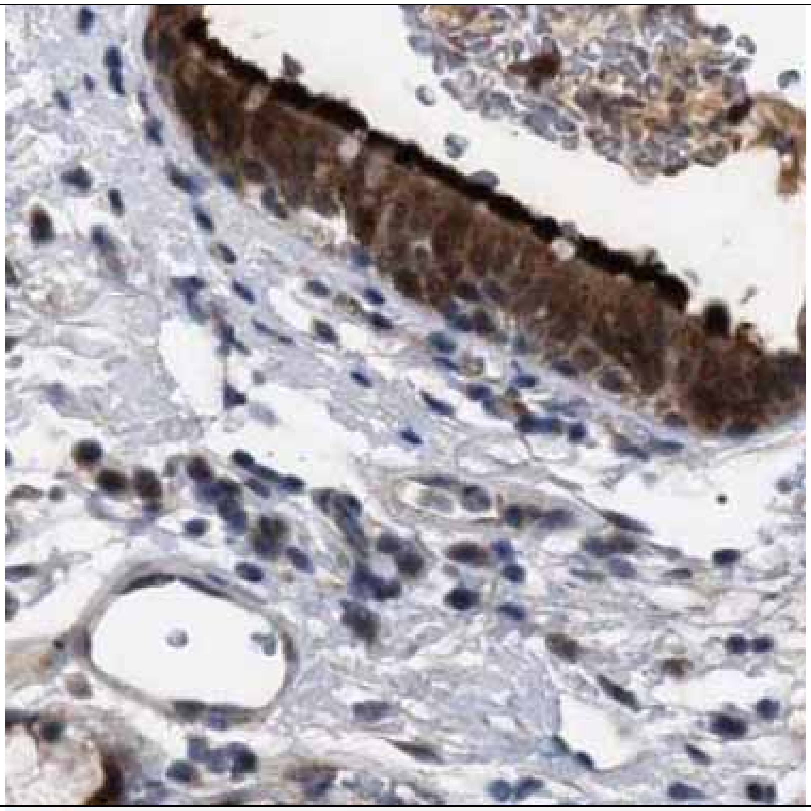	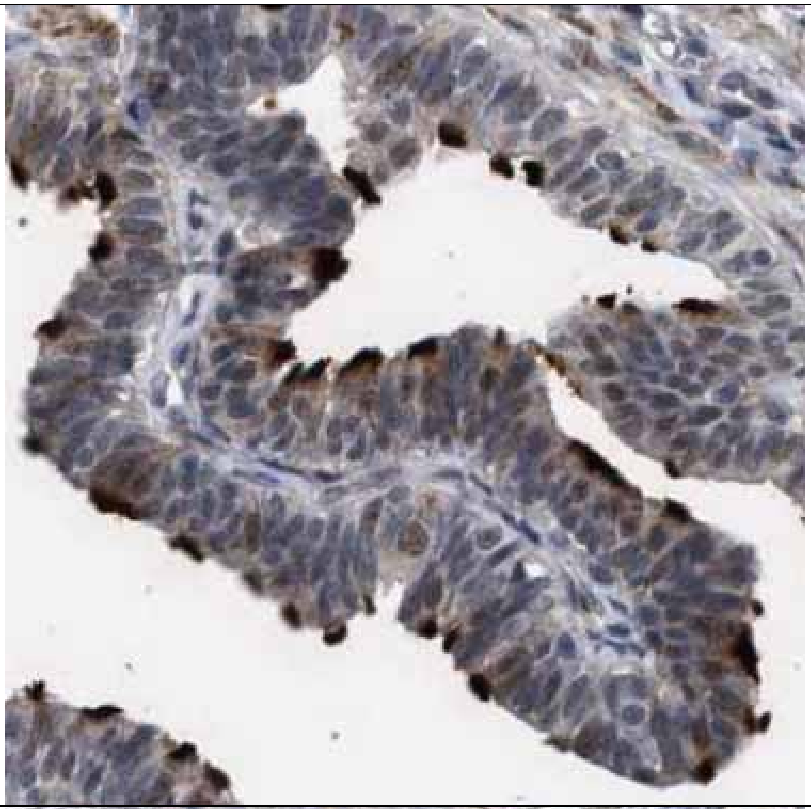	Staining is specific to *cilia*
DZIP3 (antibody HPA035066)<br><br>Category 2	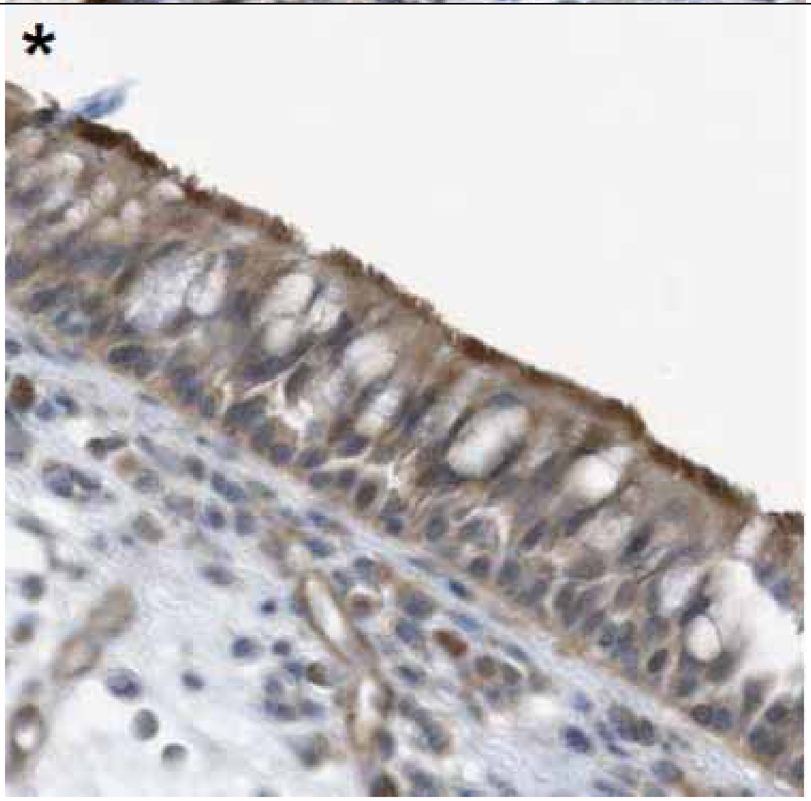	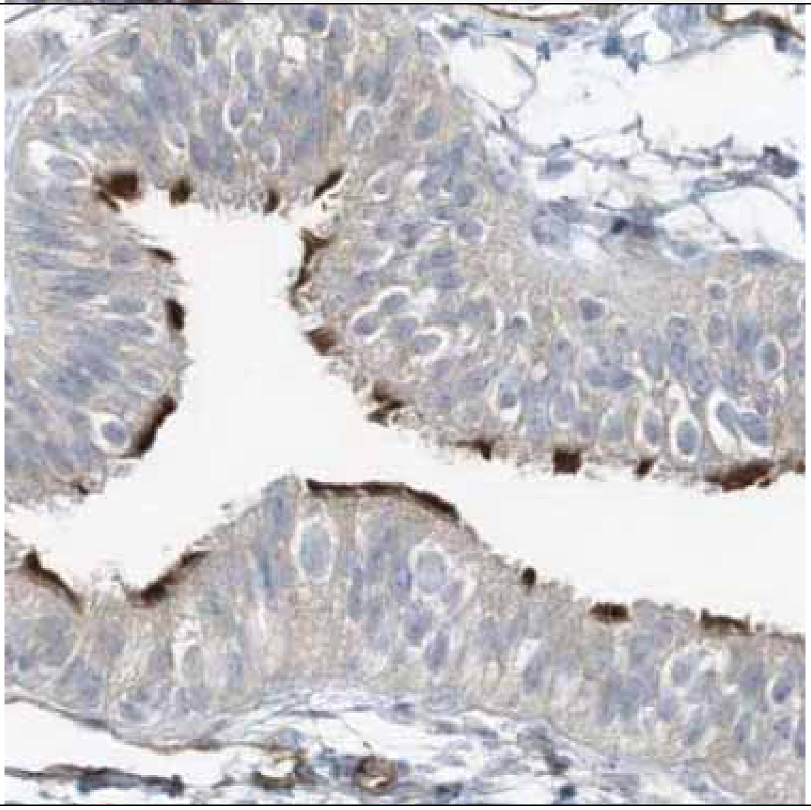	Staining is specific to *cilia*

FSD1L (antibody HPA035138)<br><br>Category 2	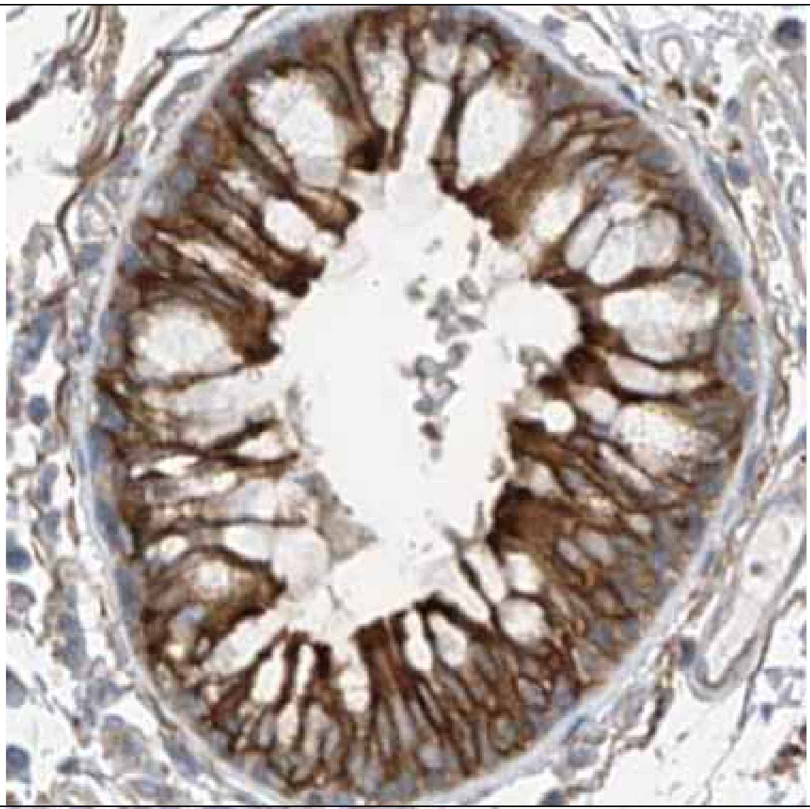	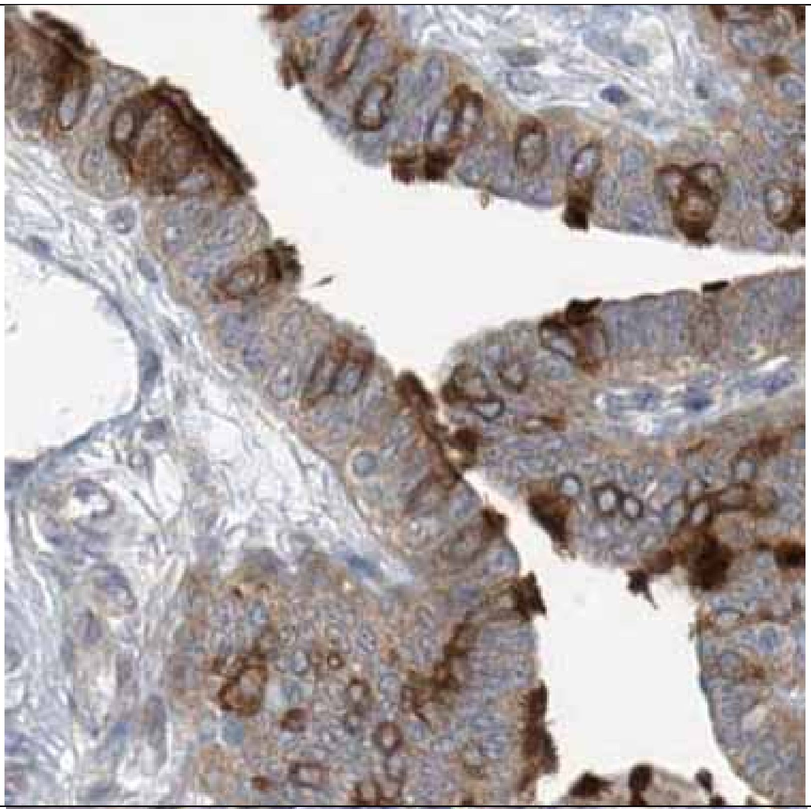	Staining is specific to *cilia*
KCNRG (antibody HPA001741)<br><br>Category 2	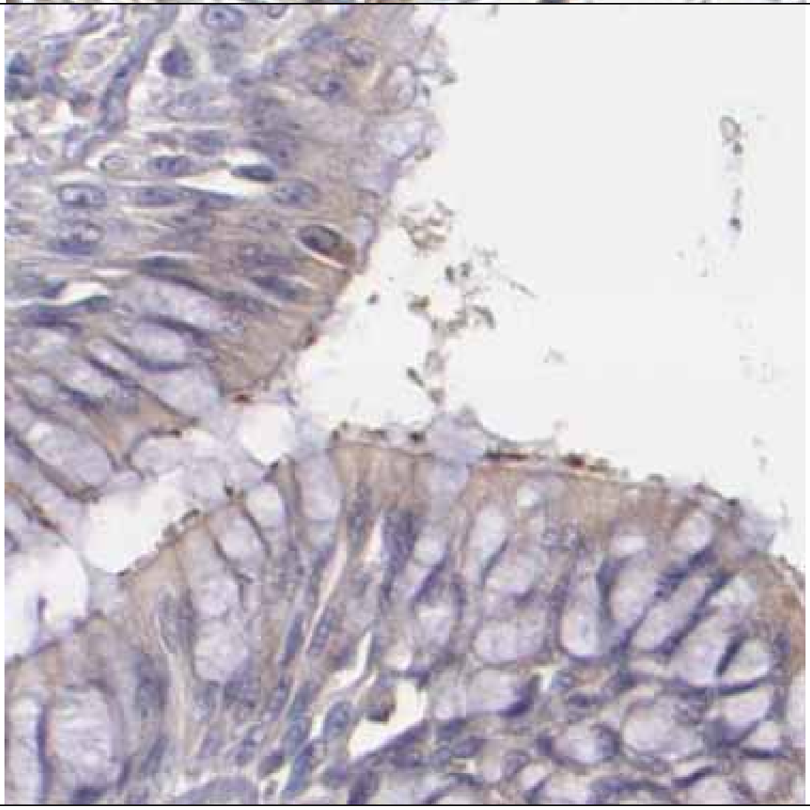	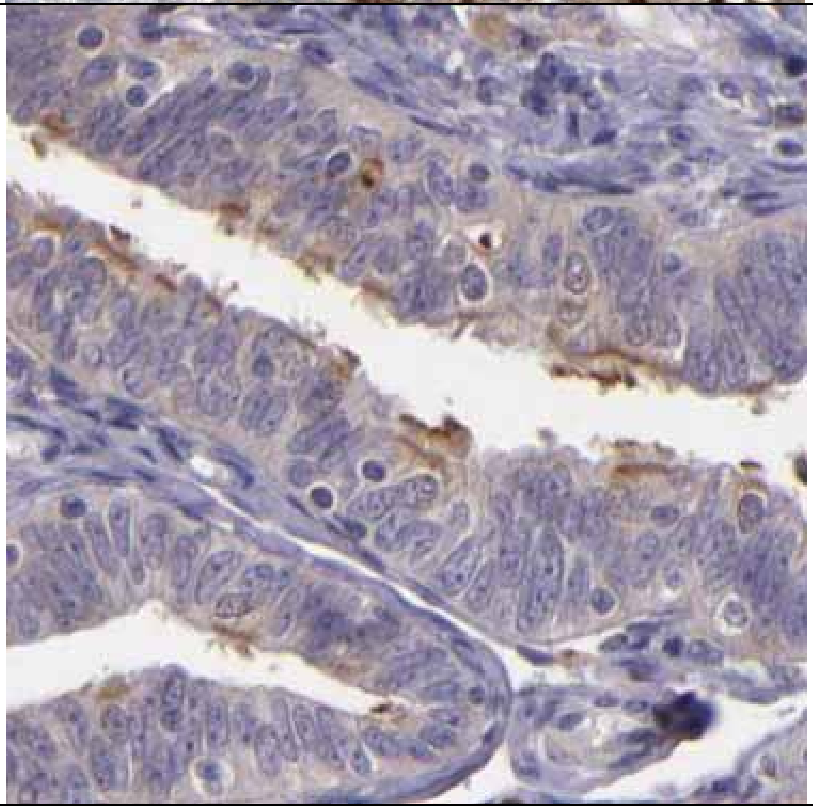	Staining is specific to *cilia*

LRP2BP (antibody HPA036665)<br><br>Category 2	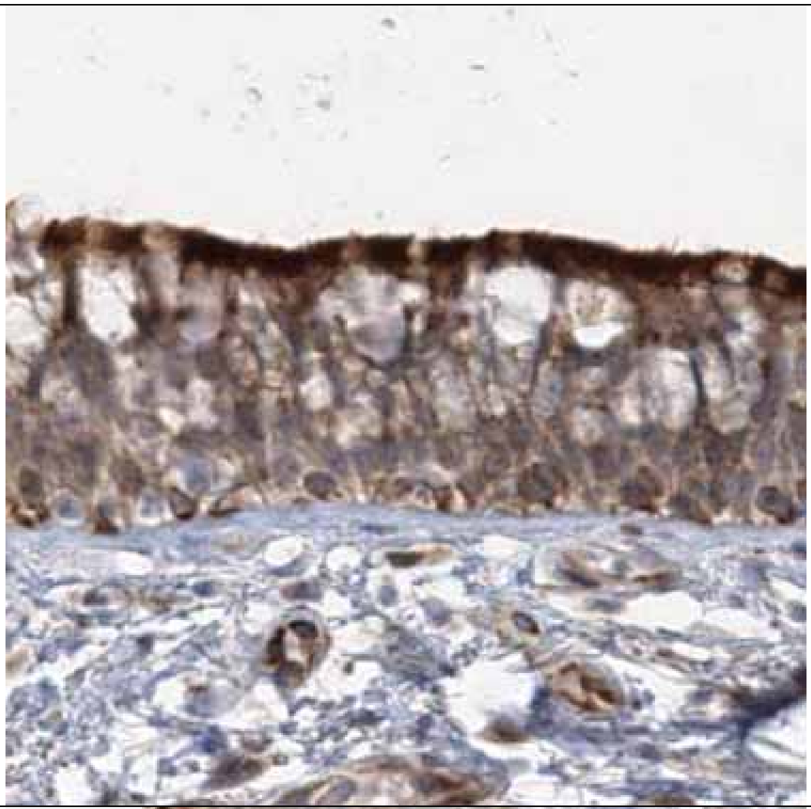	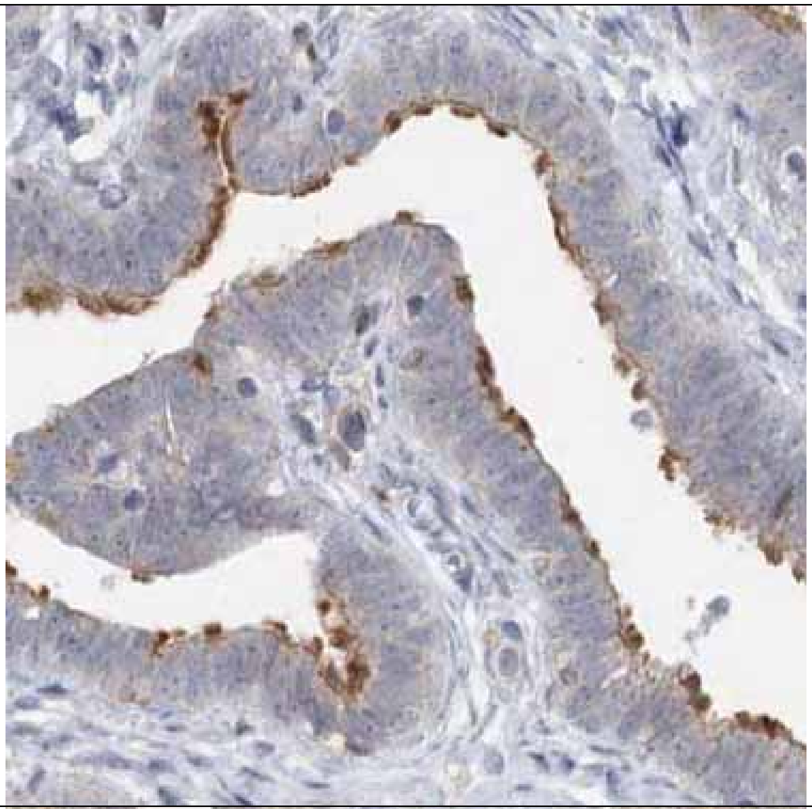	Staining is specific to *cilia*
MIPEP (antibody HPA031669)<br><br>Category 2	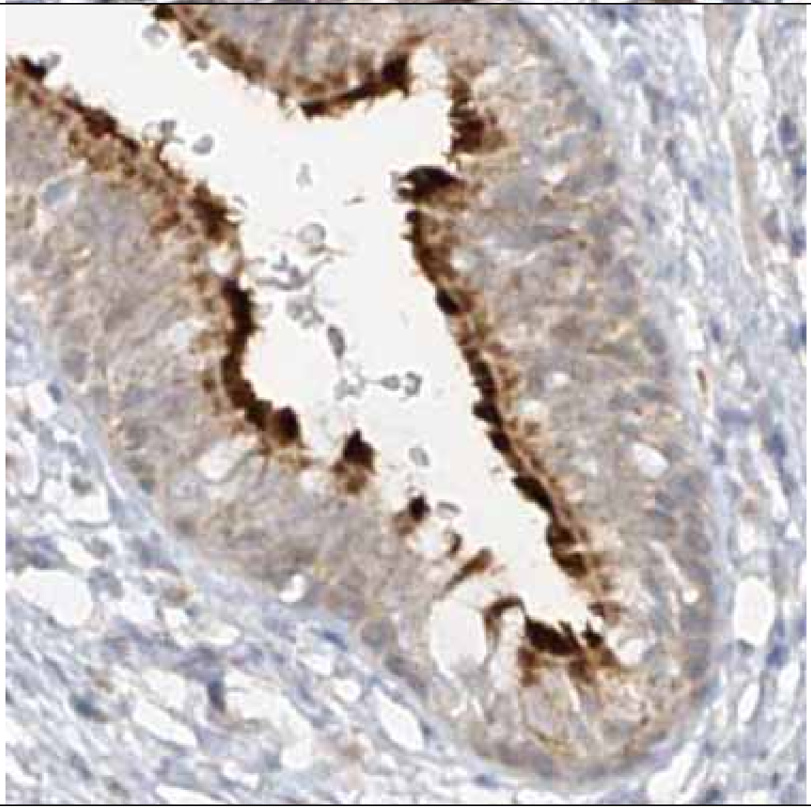	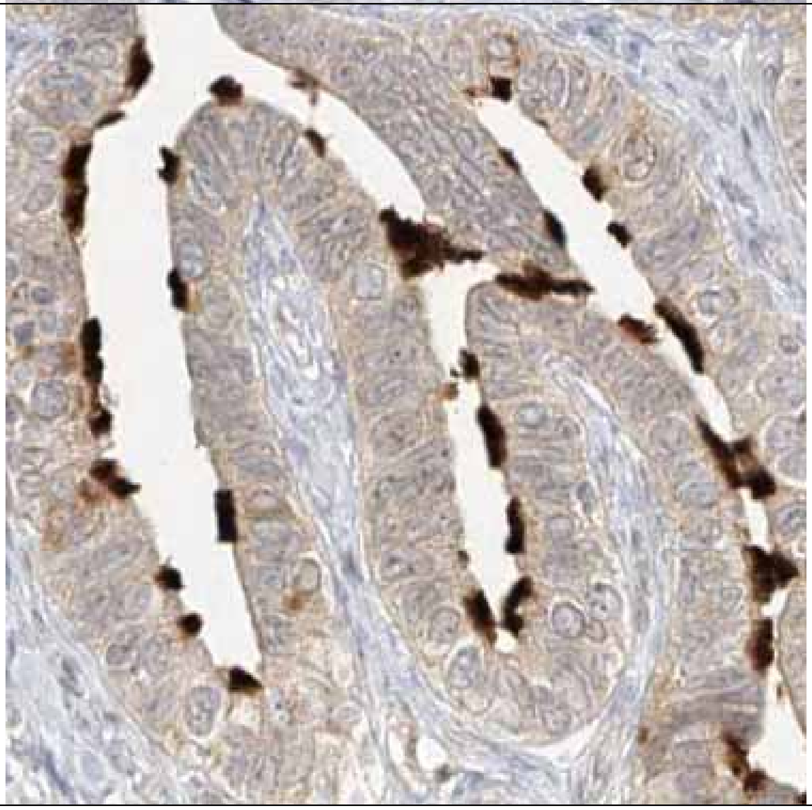	Staining is specific to *cilia*

PLCH1 (antibody HPA036176)<br><br>Category 2	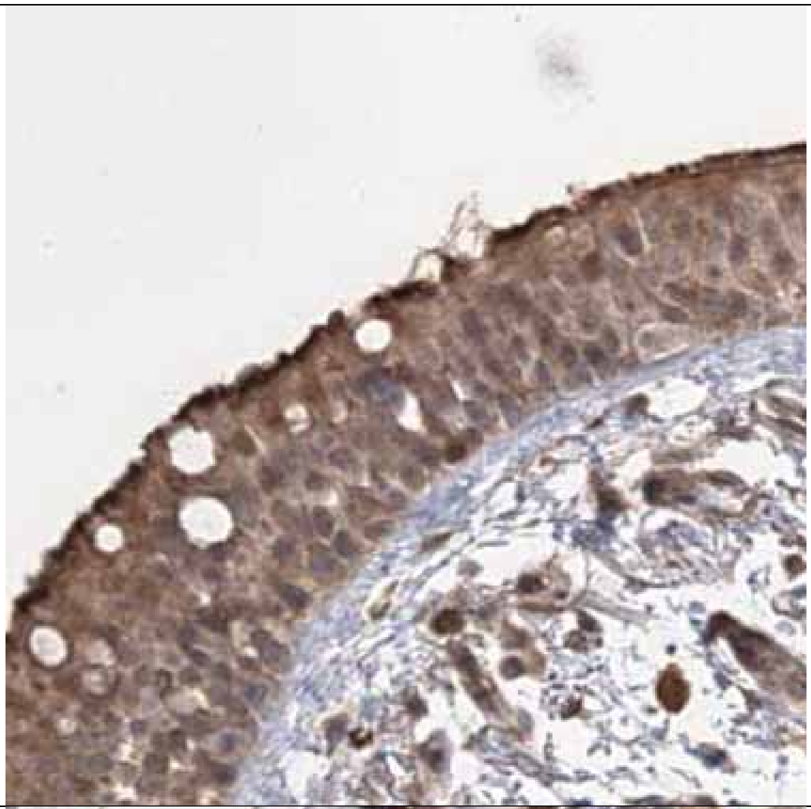	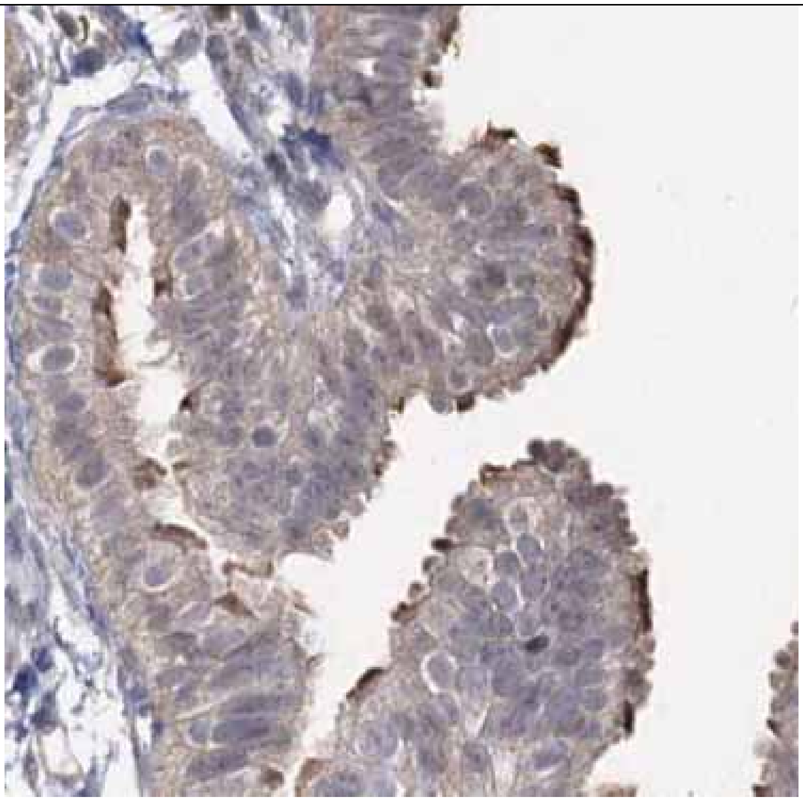	Staining is specific to *cilia*
SPAG17 (antibody HPA028597)<br><br>Category 2	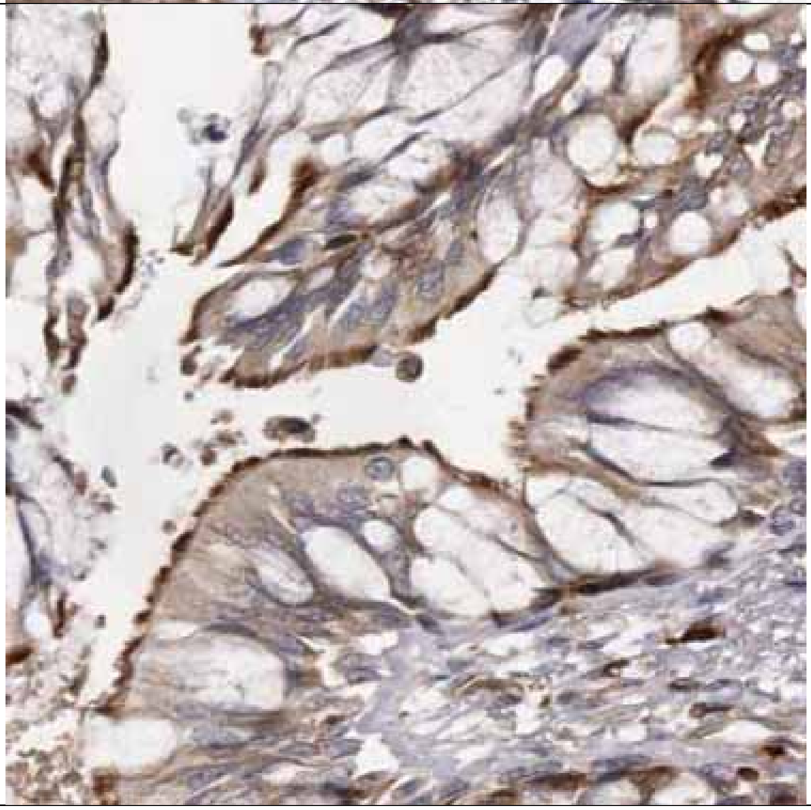	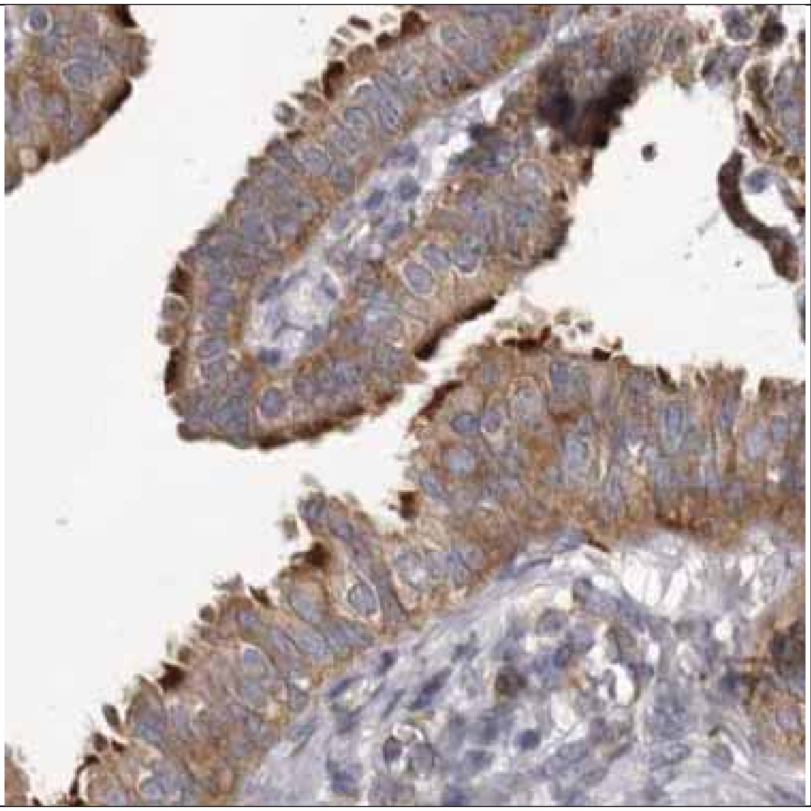	Staining is specific to *cilia*

UBXN10 (antibody HPA028555)<br><br>Category 2	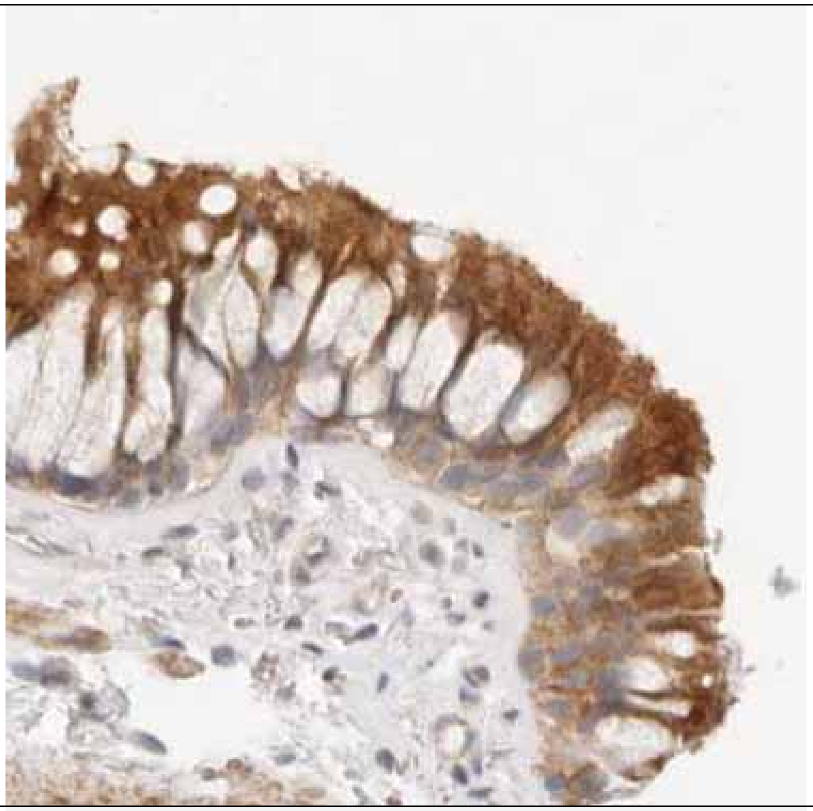	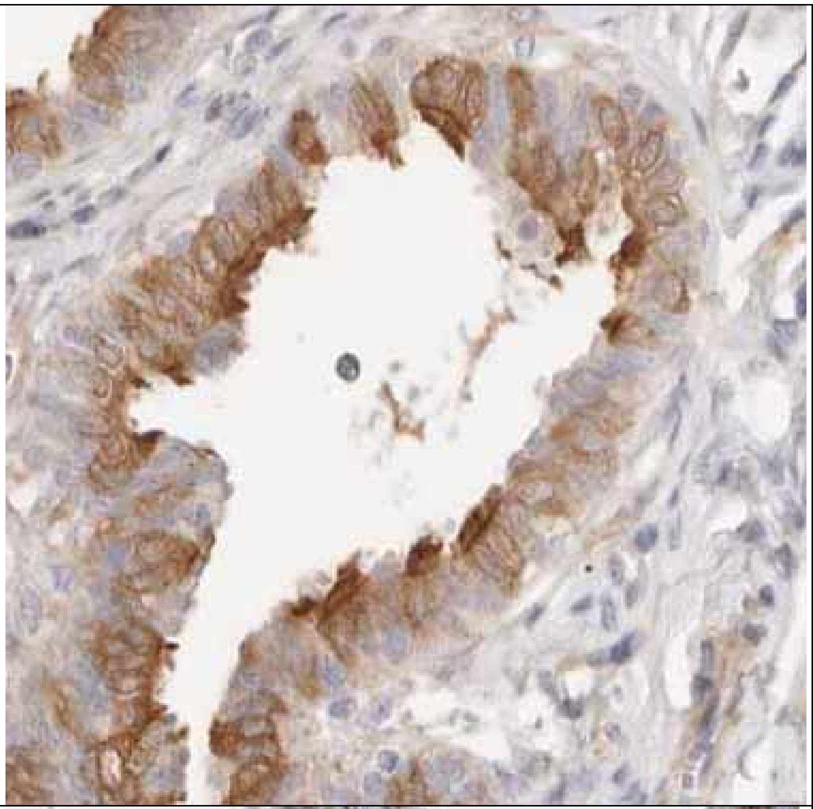	Staining is specific to *cilia*
C6orf103 (antibody HPA036341)<br><br>Category 2	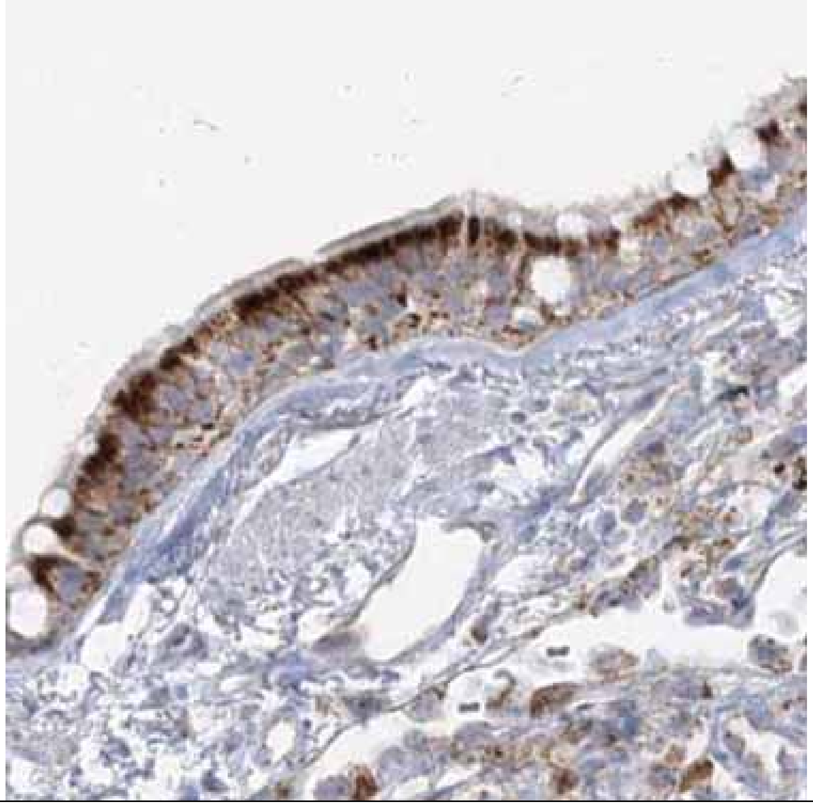	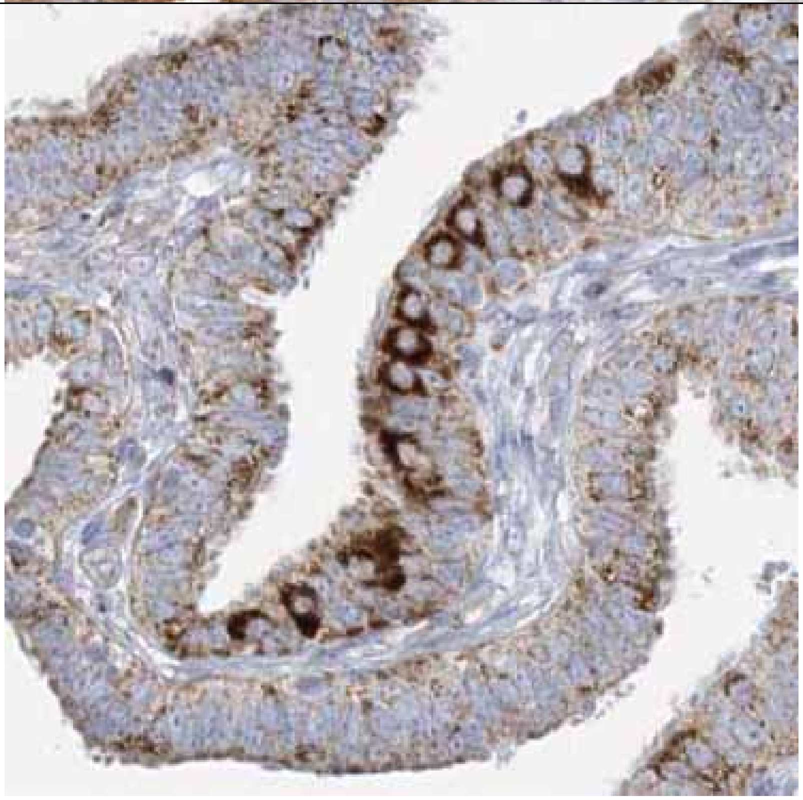	Staining is specific to *ciliated cells* (uncertain subcellular localization)

FANK1 (antibody HPA038413)<br><br>Category 2	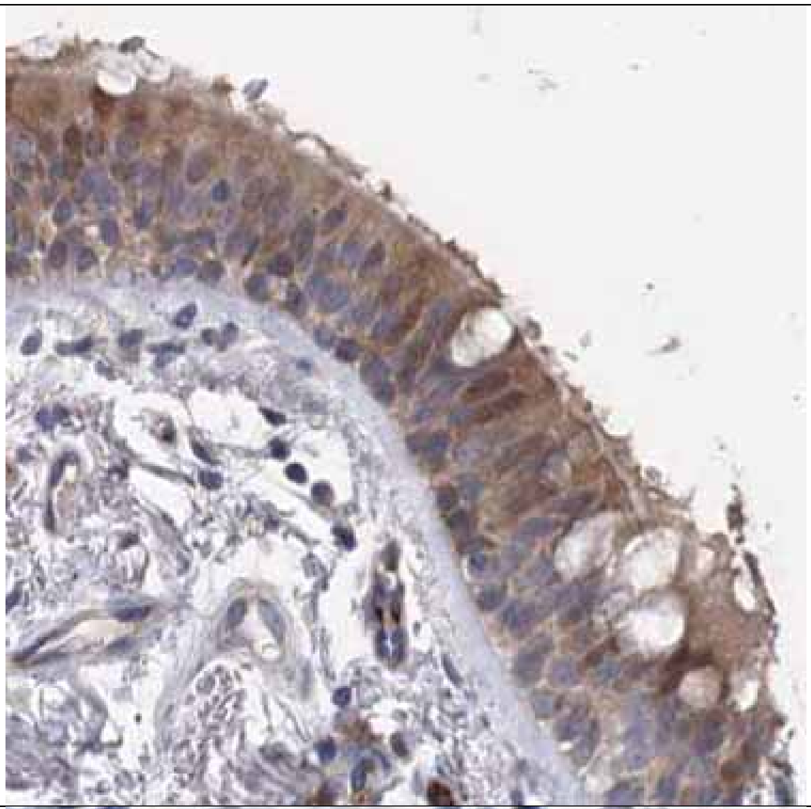	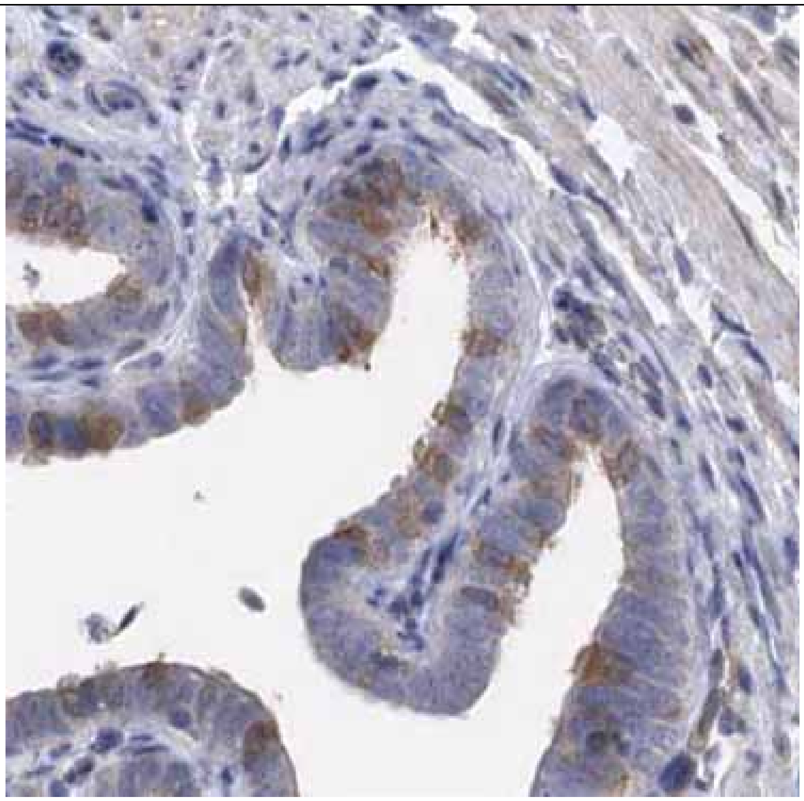	Staining is specific to *ciliated cells* (cilia and cytoplasm)
LRRC6 (antibody HPA028058)<br><br>Category 2	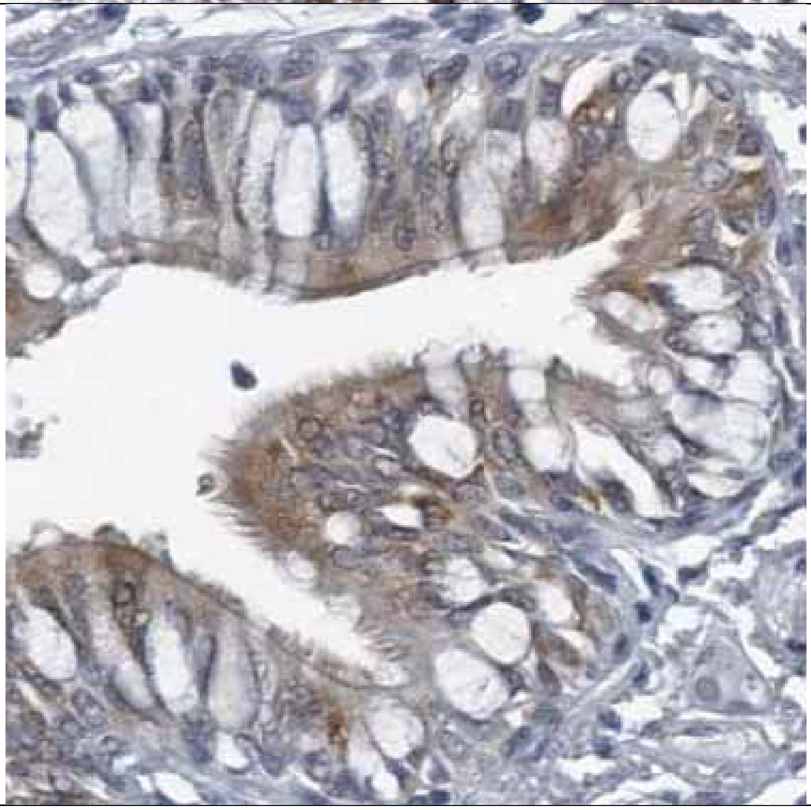	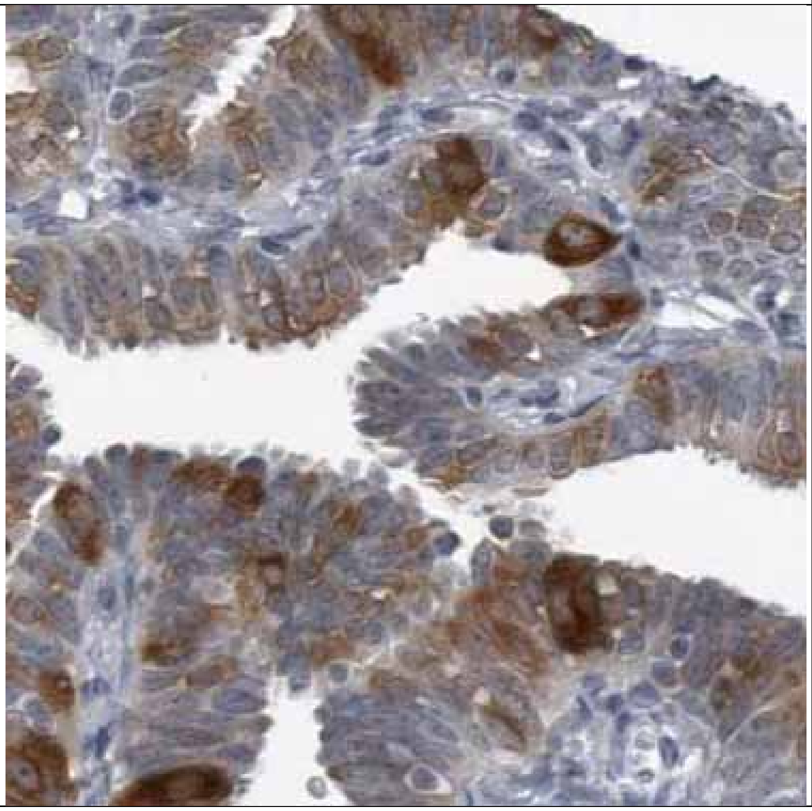	Staining is specific to *ciliated cells* (uncertain subcellular localization)

NEK10 (antibody HPA038941)<br><br>Category 2	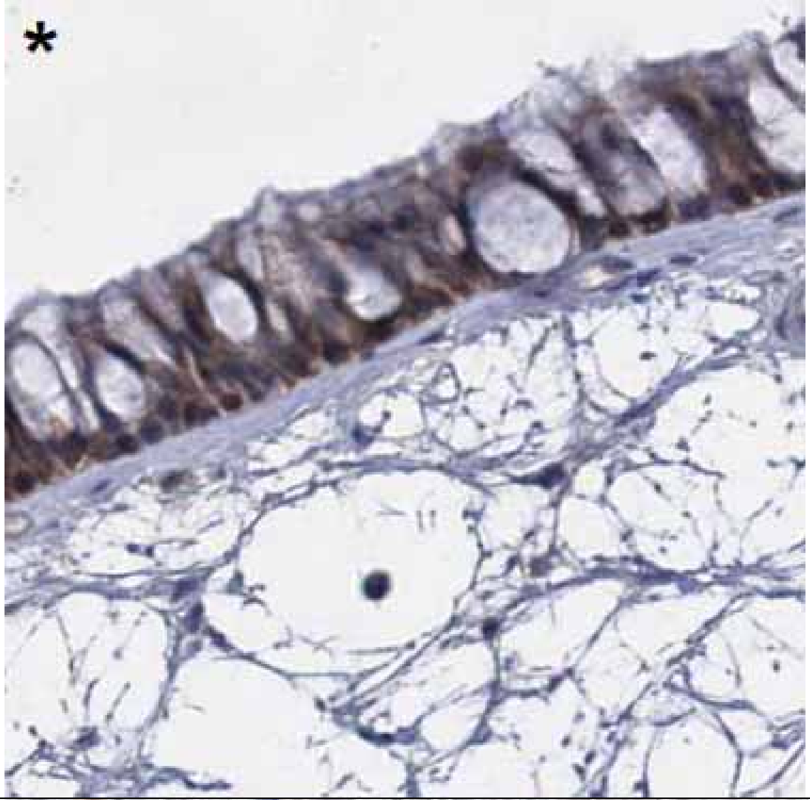	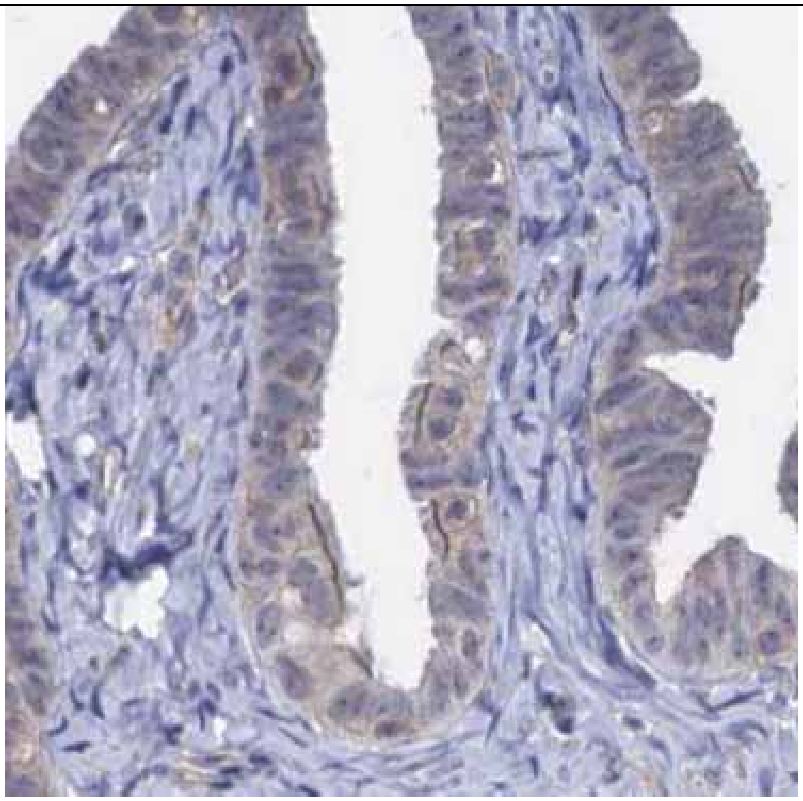	Staining is specific to *ciliated cells* (uncertain subcellular localization)
PPP1R16A (antibody HPA023755)<br><br>Category 2	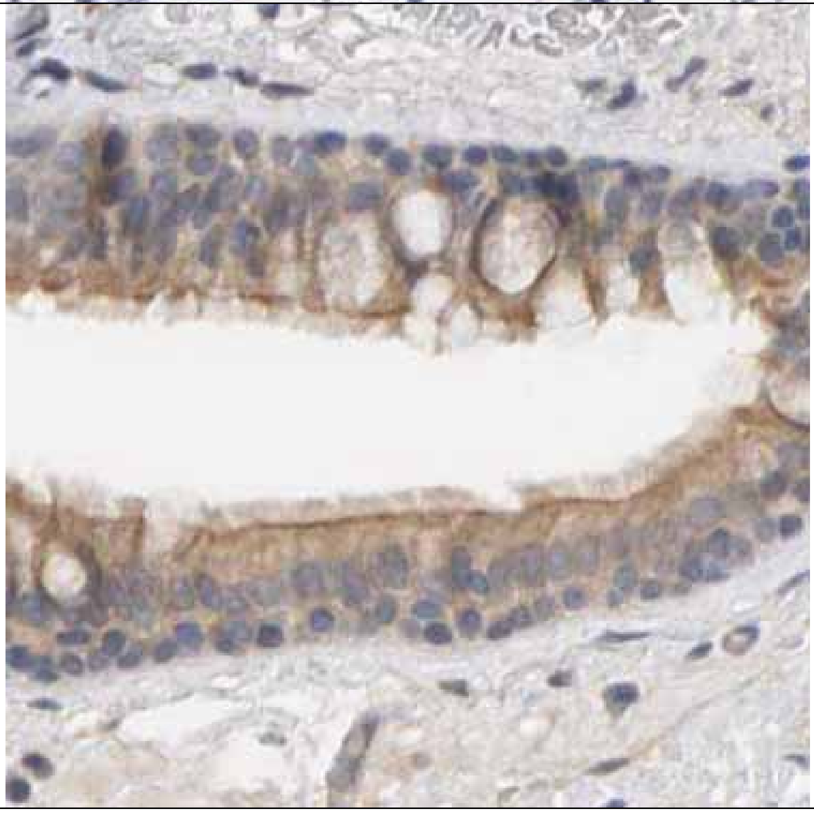	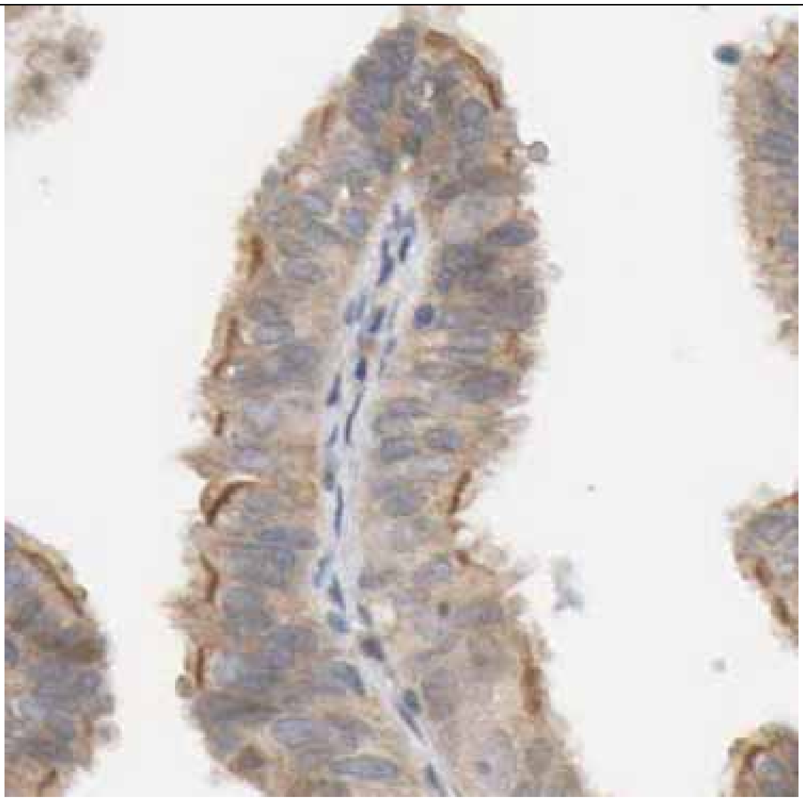	Staining is specific to *ciliated cells* (apical cytoplasm)

RBKS (antibody HPA019725)<br><br>Category 2	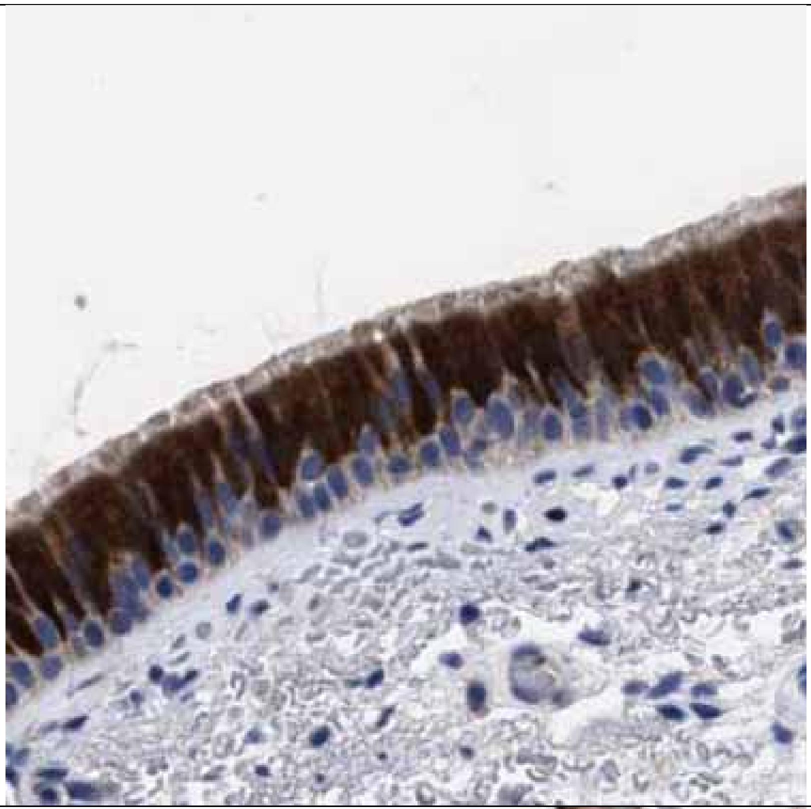	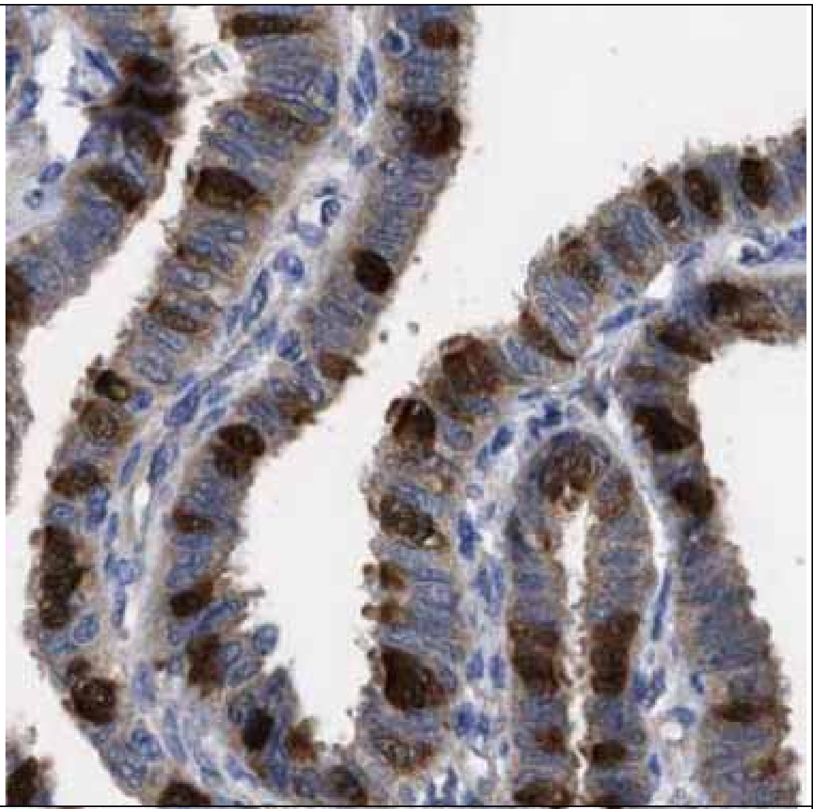	Staining is specific to *ciliated cells* (cilia and cytoplasm)
SPATA18 (antibody HPA036854)<br><br>Category 2	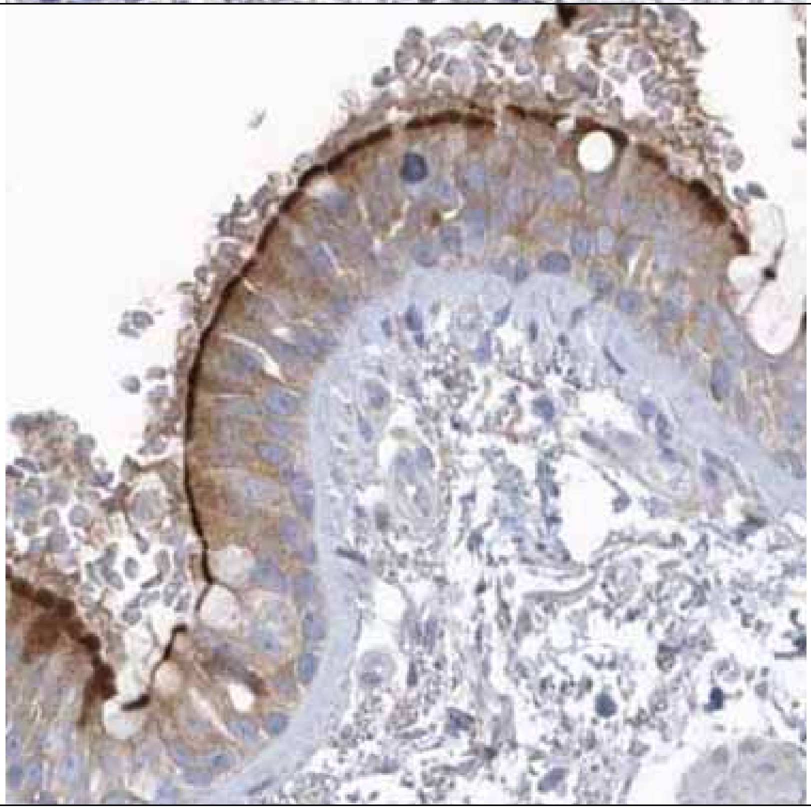	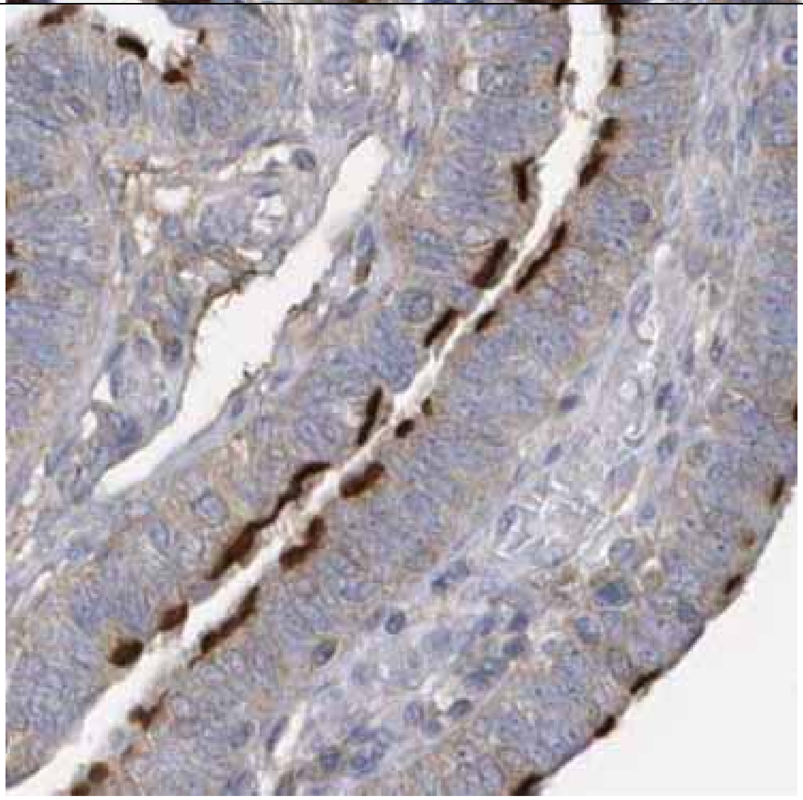	Staining is specific to *ciliated cells* (apical cytoplasm)

WDR49 (antibody HPA036225)<br><br>Category 2	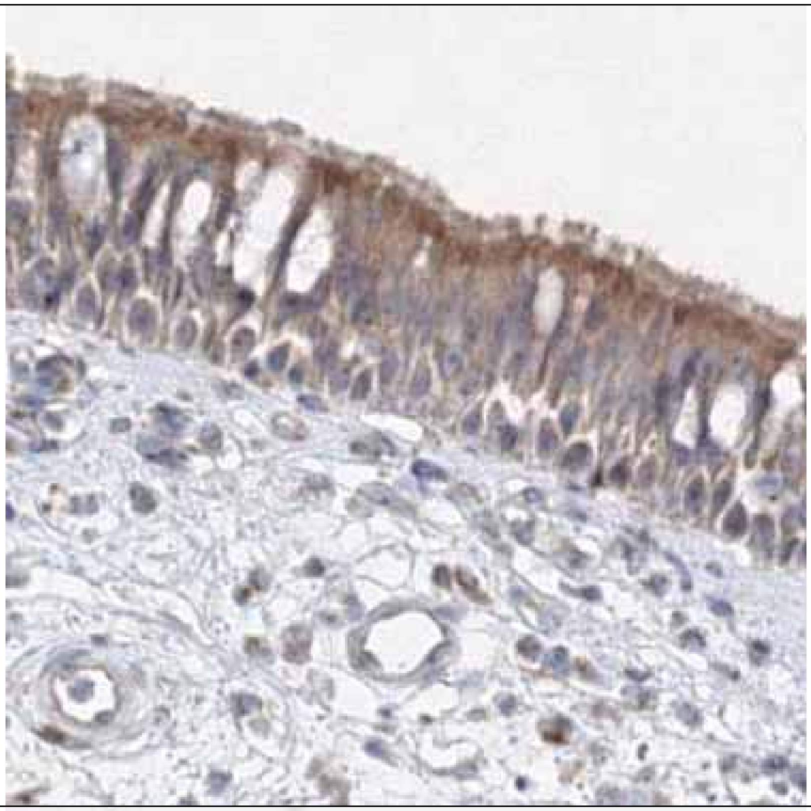	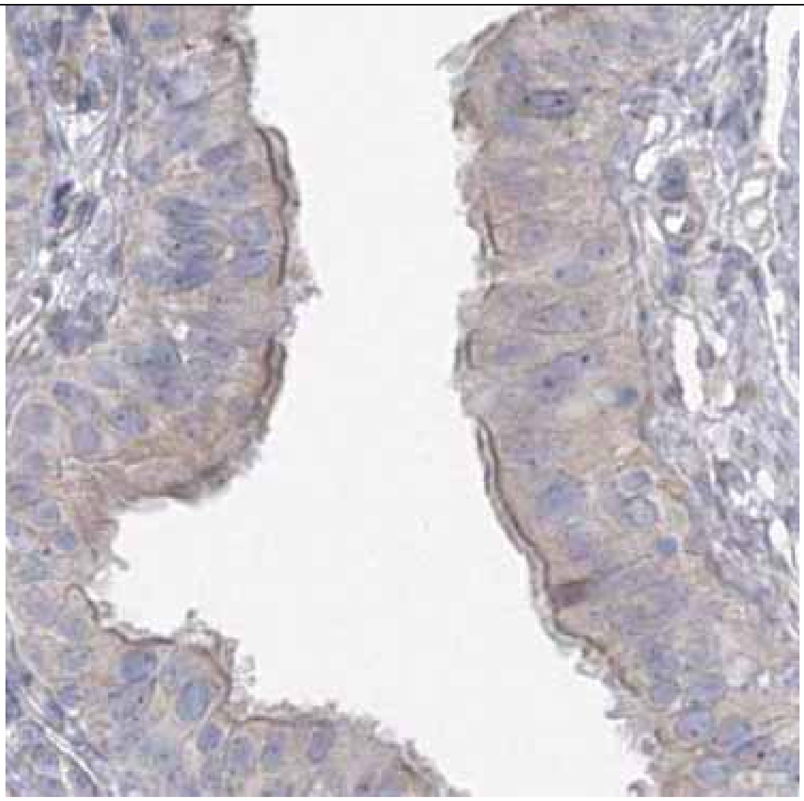	Staining is specific to *ciliated cells* (apical cytoplasm)
APOBEC4 (antibody HPA015637)<br><br>Category 2	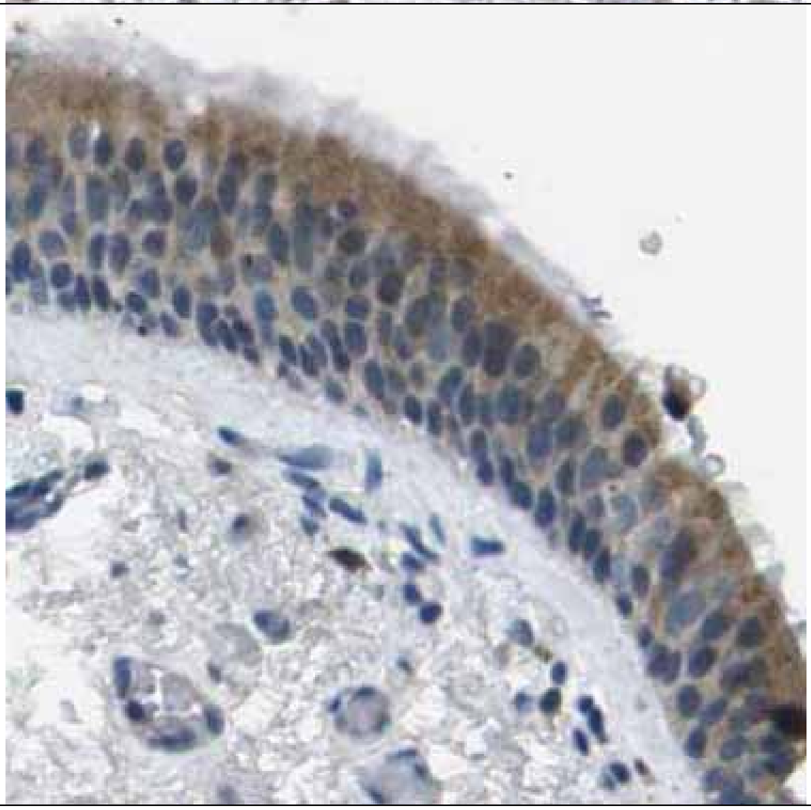	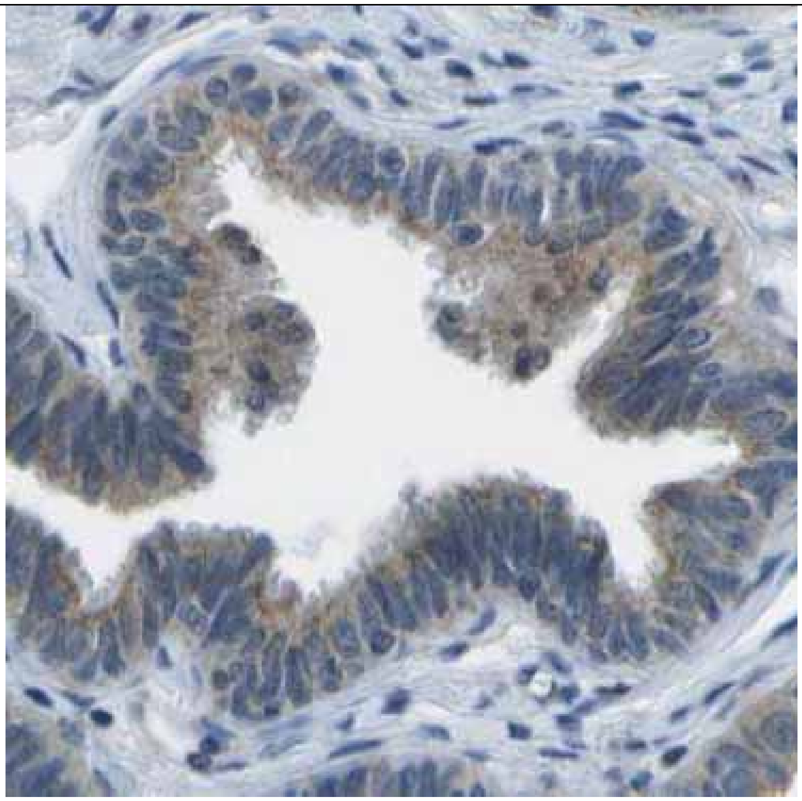	Staining is non-specific to ciliated cells or absent

C3orf25 (antibody HPA037694)<br><br>Category 2	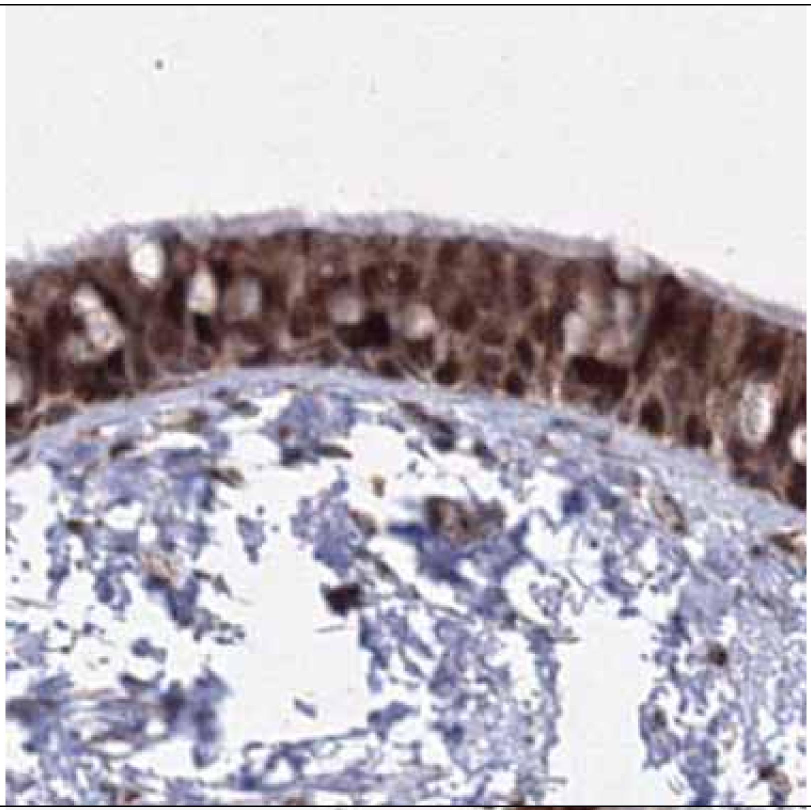	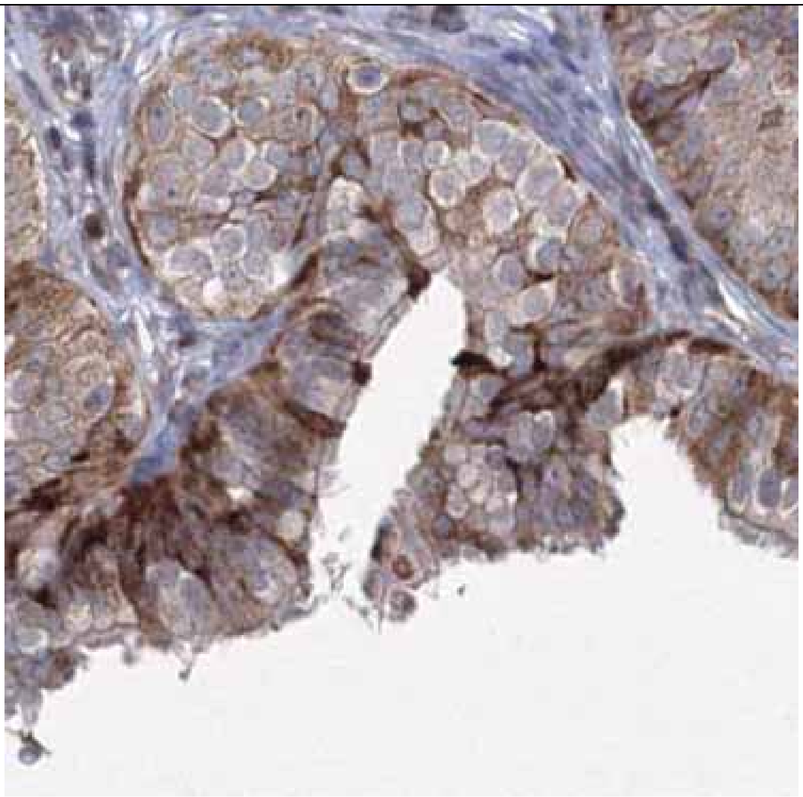	Staining is non-specific to ciliated cells or absent
CCDC60 (antibody HPA039048)<br><br>Category 2	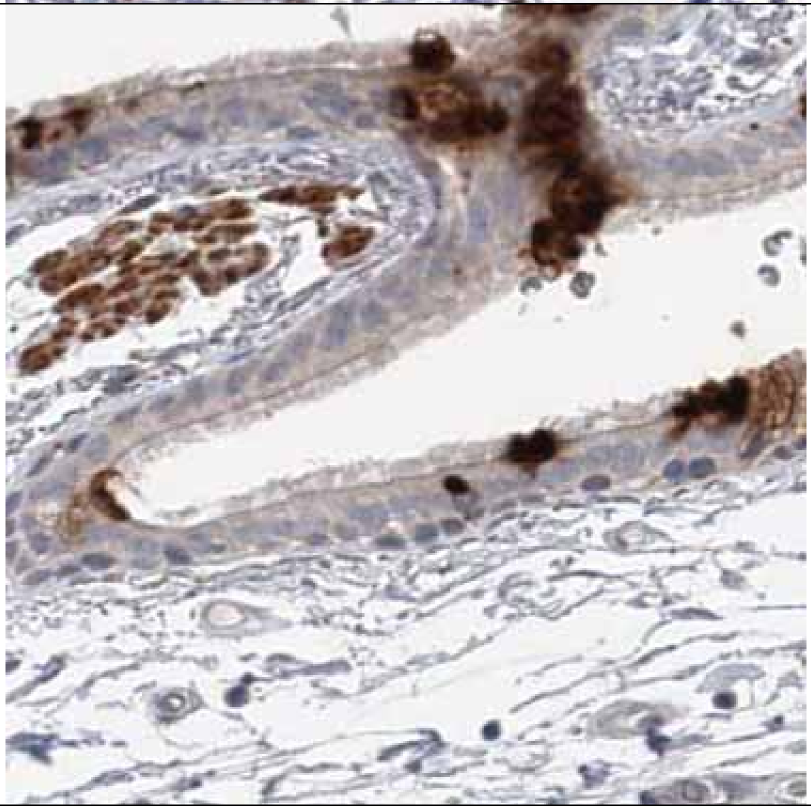	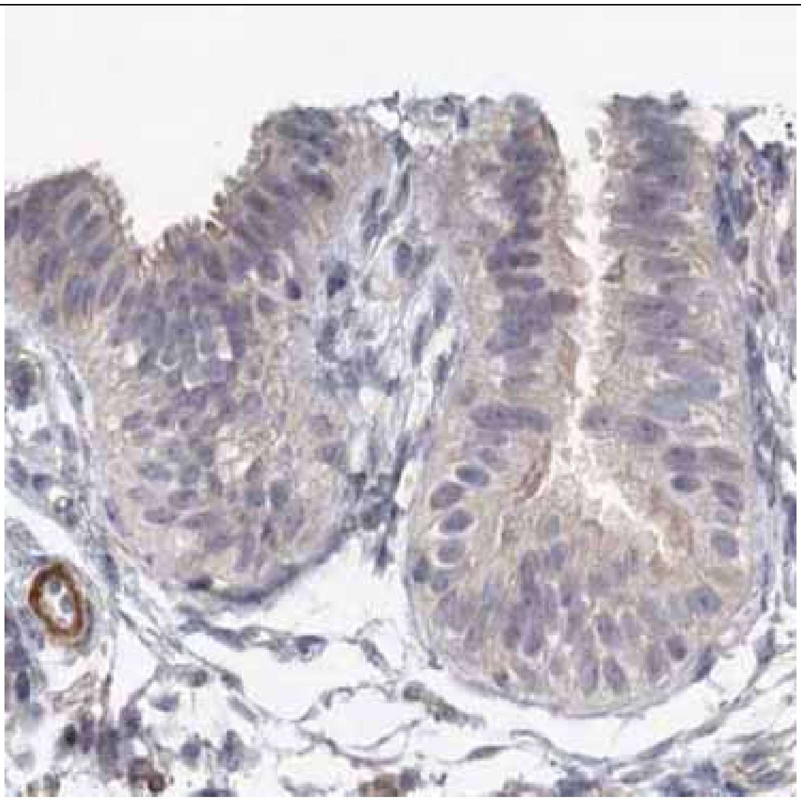	Staining is non-specific to ciliated cells or absent

FAM179A (antibody HPA037561)<br><br>Category 2	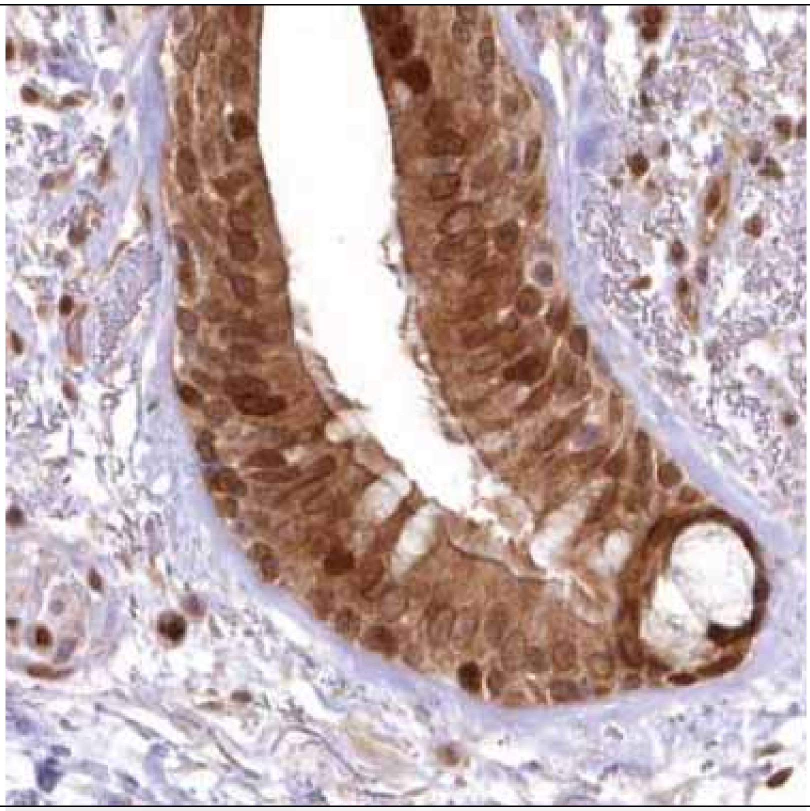	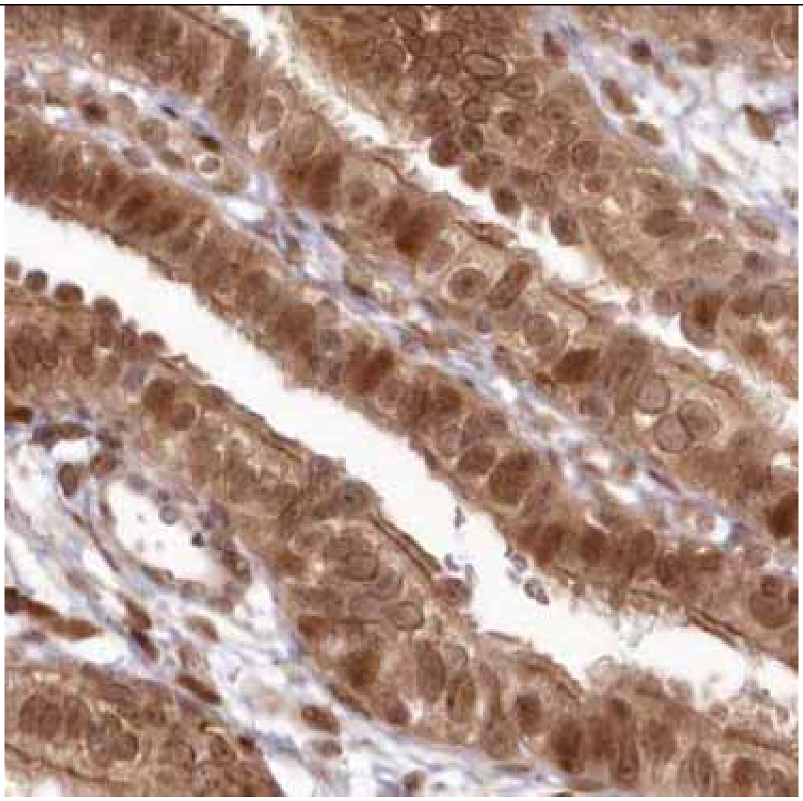	Staining is non-specific to ciliated cells or absent
KIAA1377 (antibody HPA038399)<br><br>Category 2	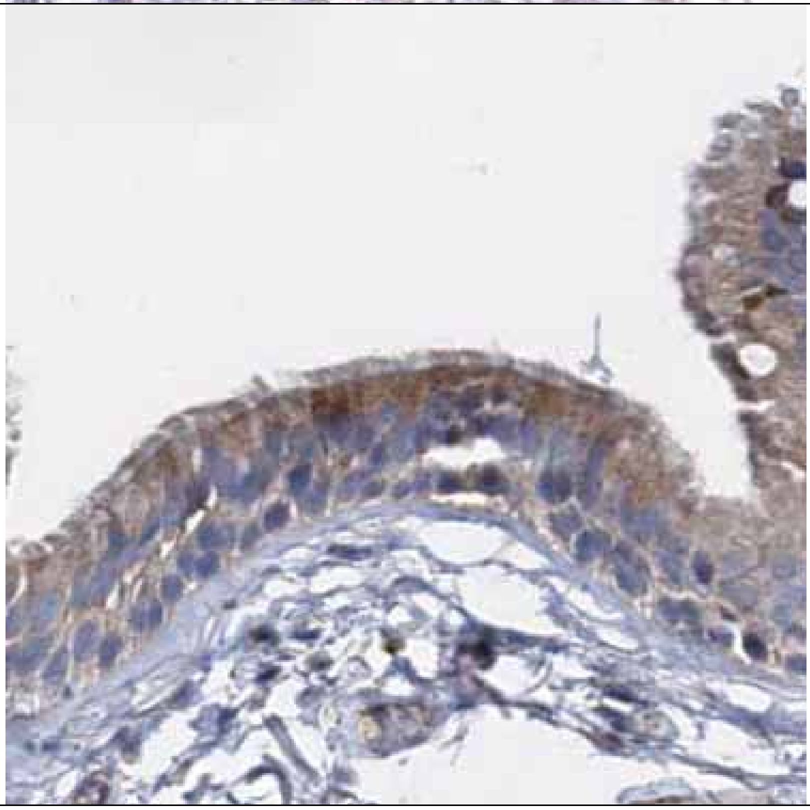	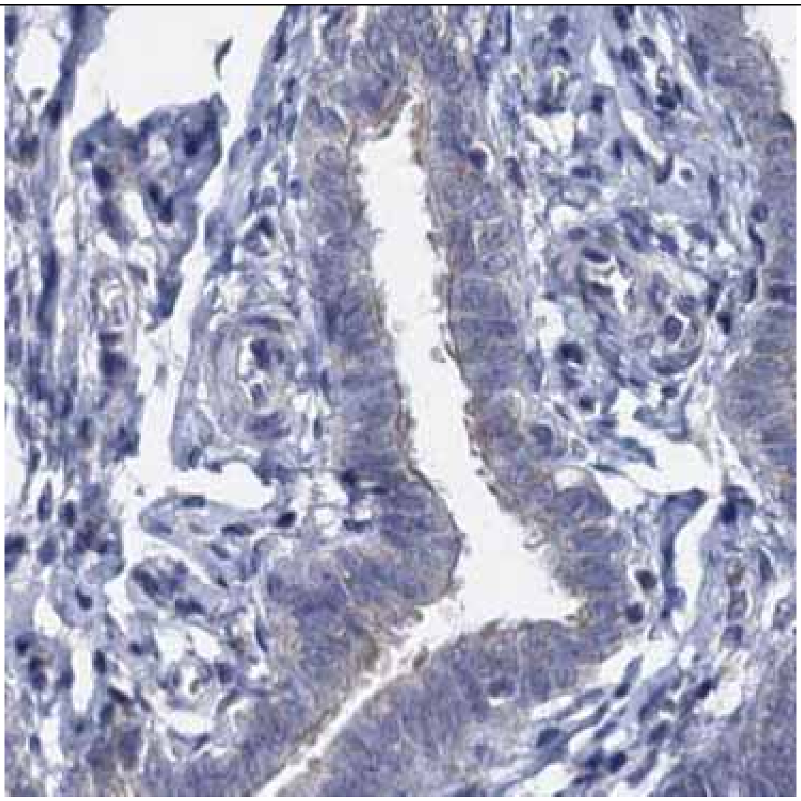	Staining is non-specific to ciliated cells or absent

KLHDC9 (antibody HPA032058)<br><br>Category 2	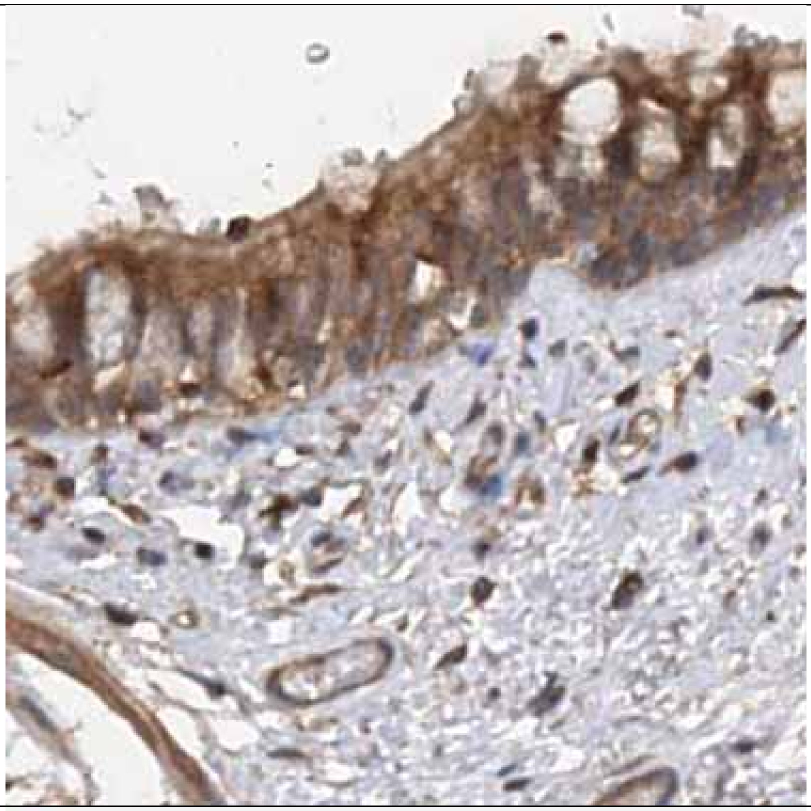	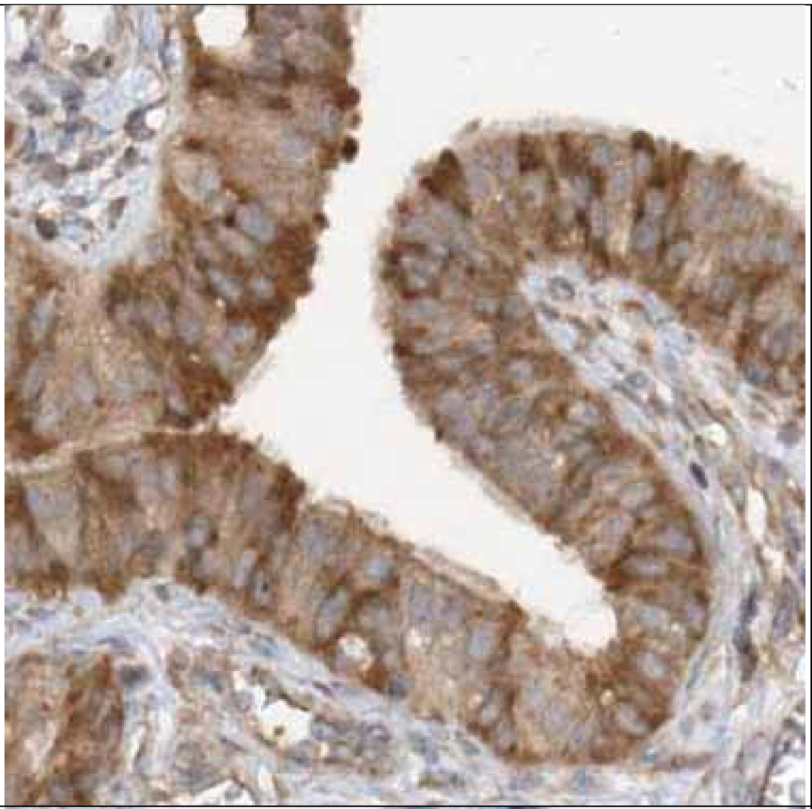	Staining is non-specific to ciliated cells or absent
NEK11 (antibody HPA016908)<br><br>Category 2	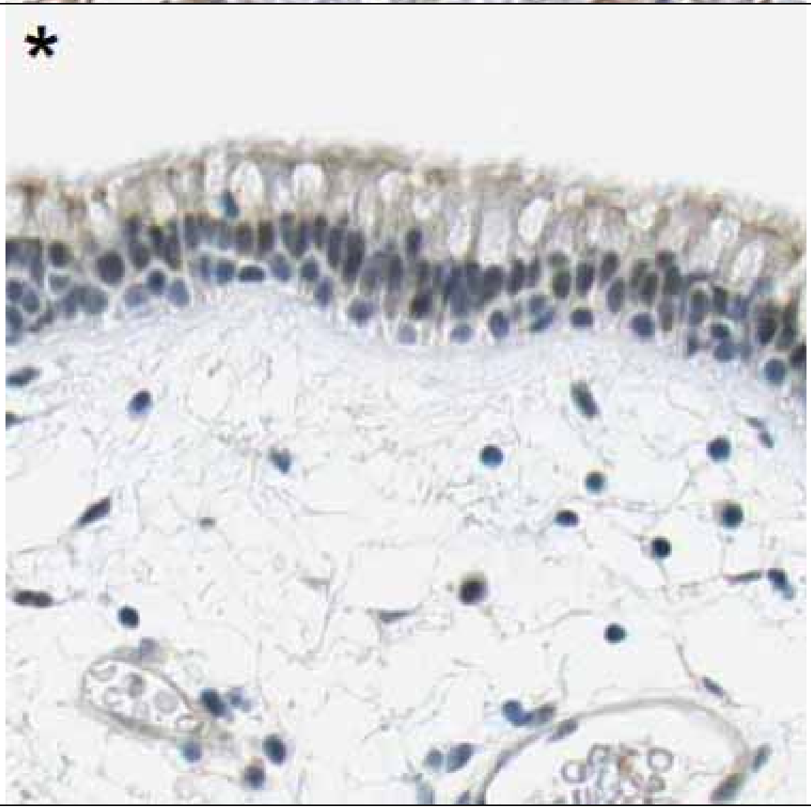	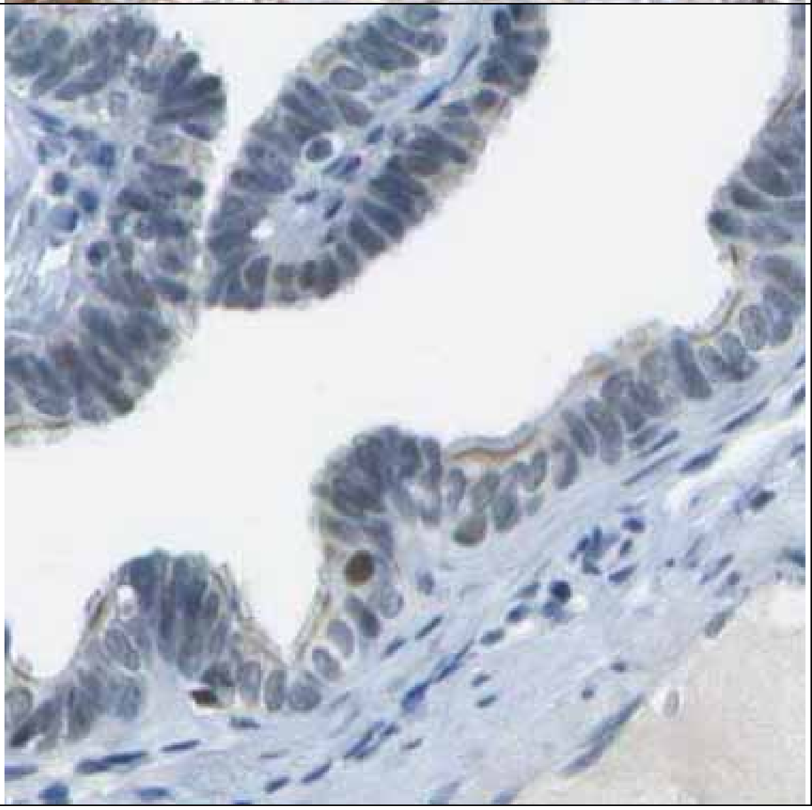	Staining is non-specific to ciliated cells or absent

SLFN13<br>(antibody)<br><br>Category 2	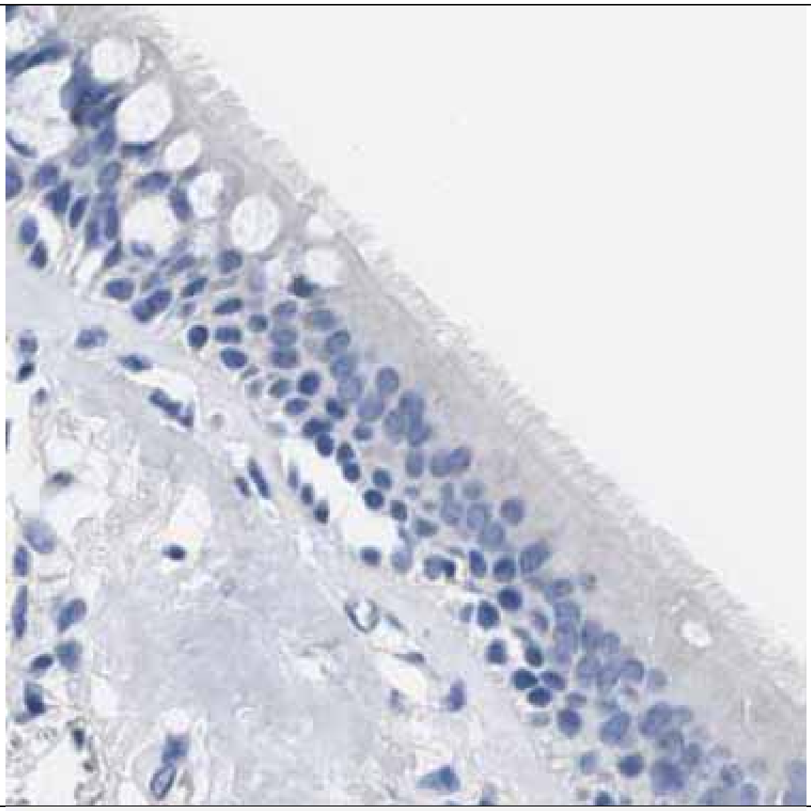	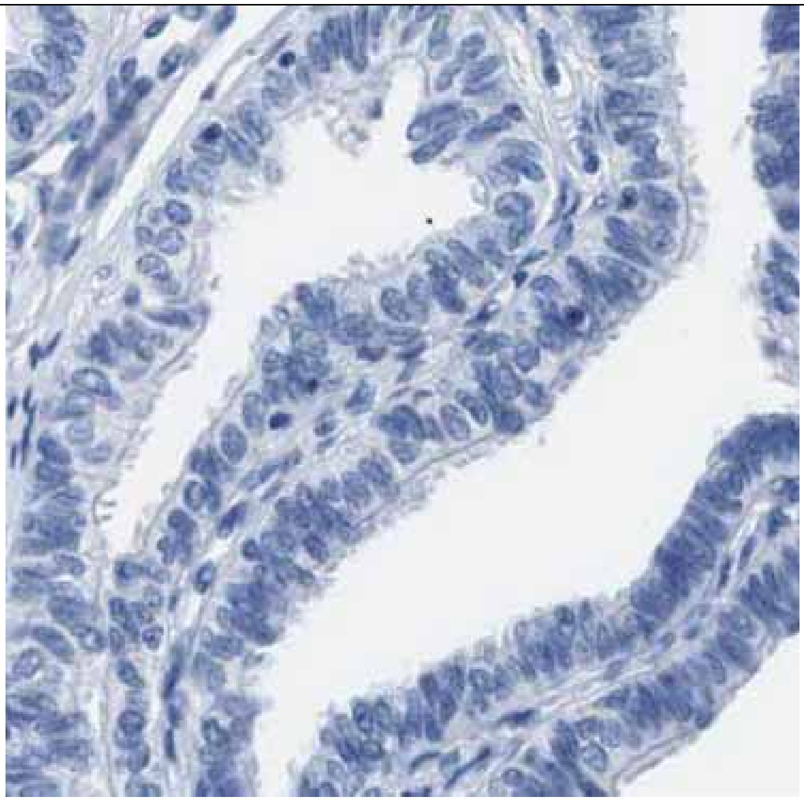	Staining is non-specific to ciliated cells or absent
SPATA4<br>(antibody HPA019220)<br><br>Category 2	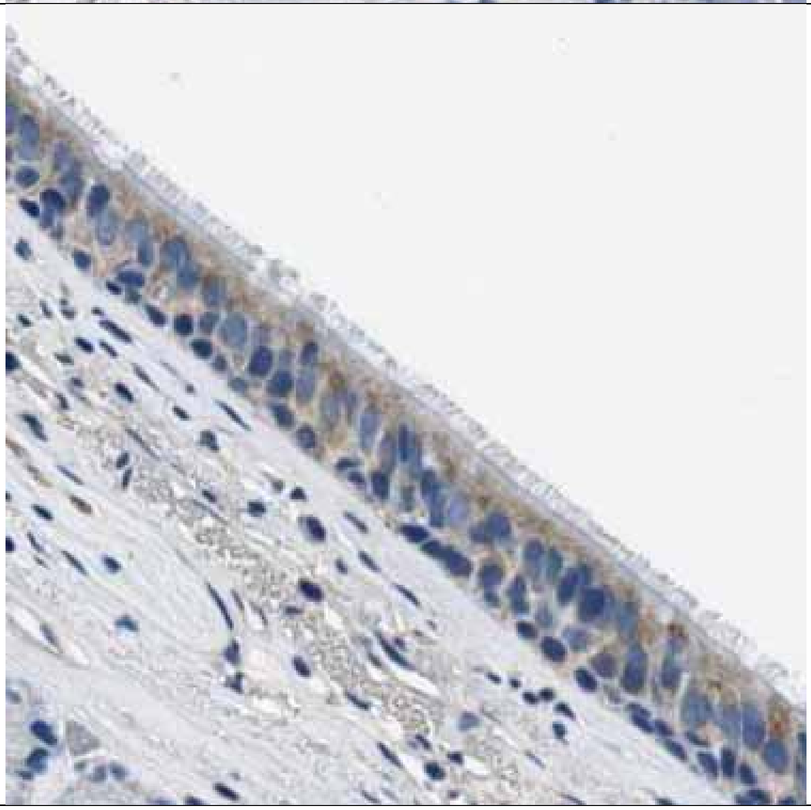	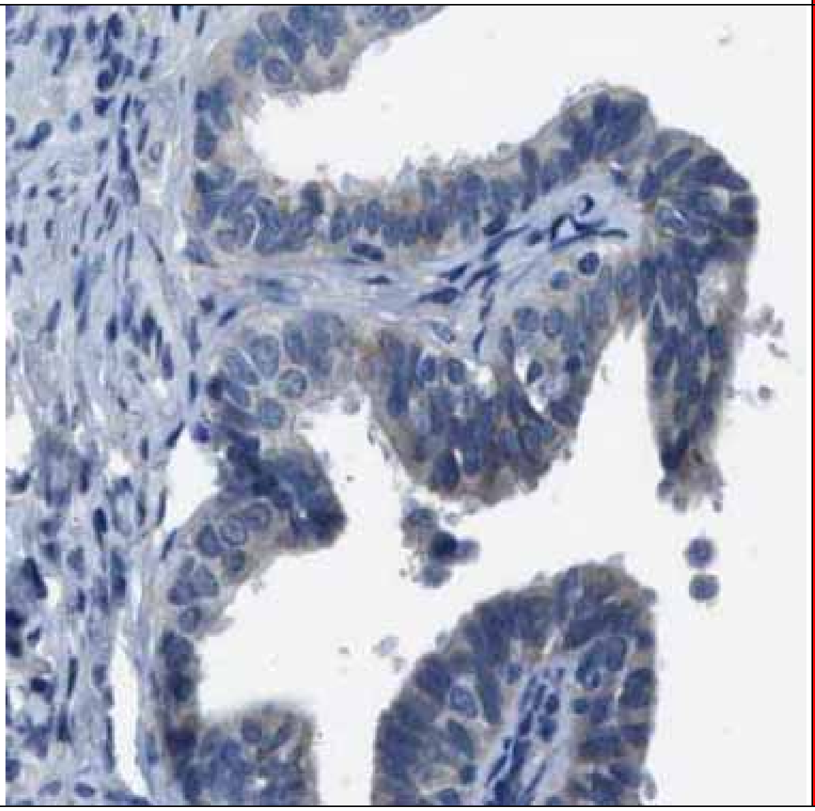	Staining is non-specific to ciliated cells or absent

STOX1 (antibody HPA037845)<br><br>Category 2	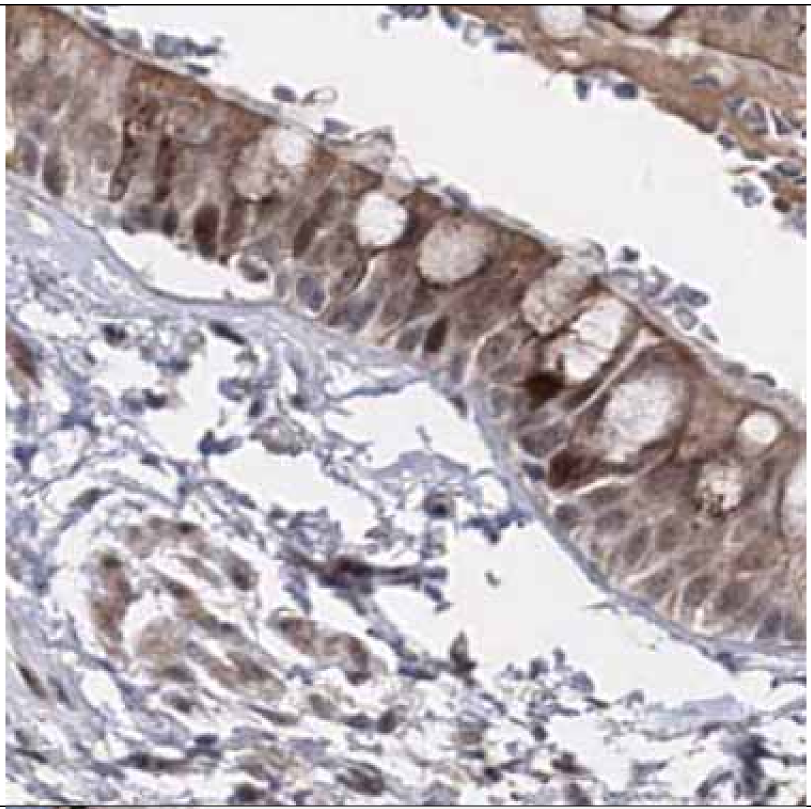	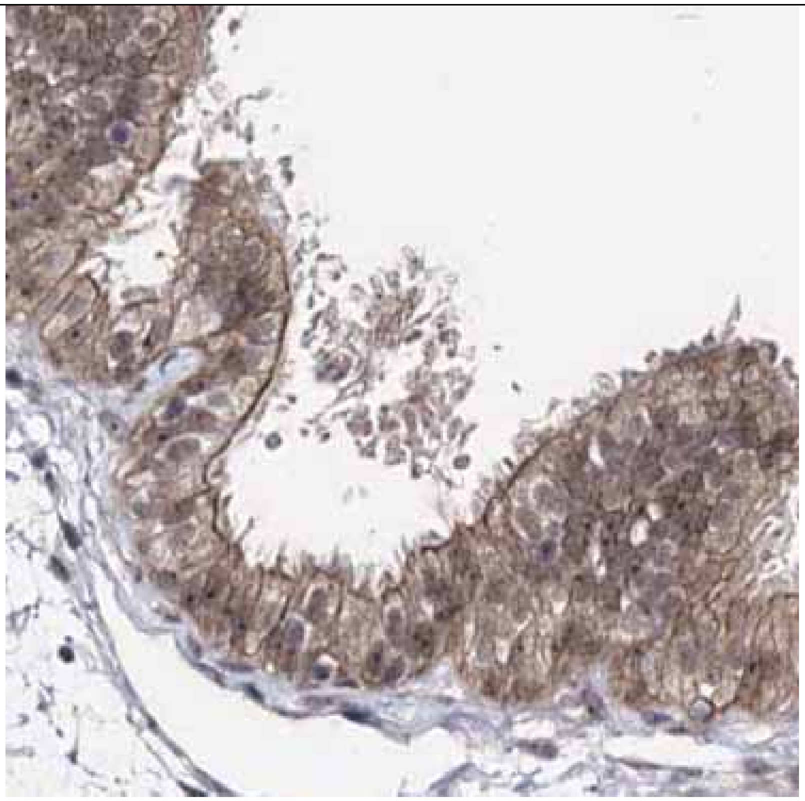	Staining is non-specific to ciliated cells or absent
TMEM17 (antibody HPA018100)<br><br>Category 2	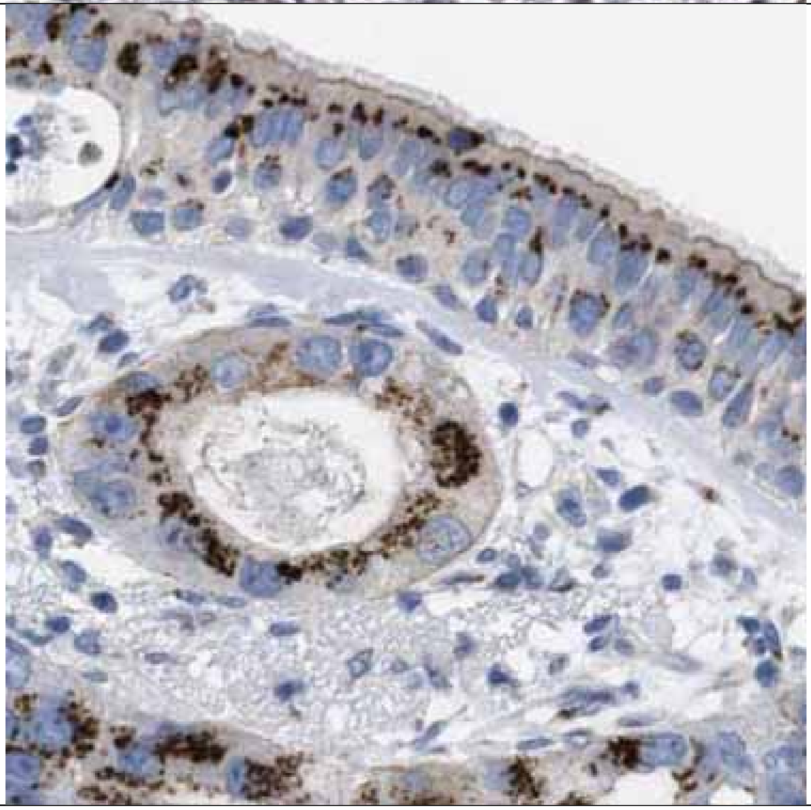	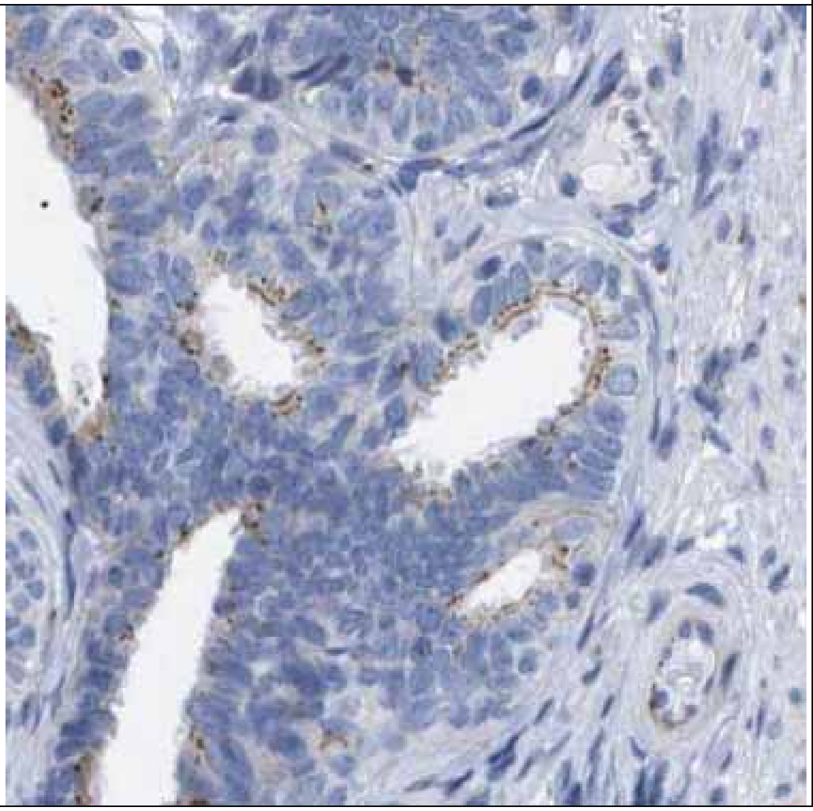	Staining is non-specific to ciliated cells or absent

WDR31 (antibody HPA019347)<br><br>Category 2	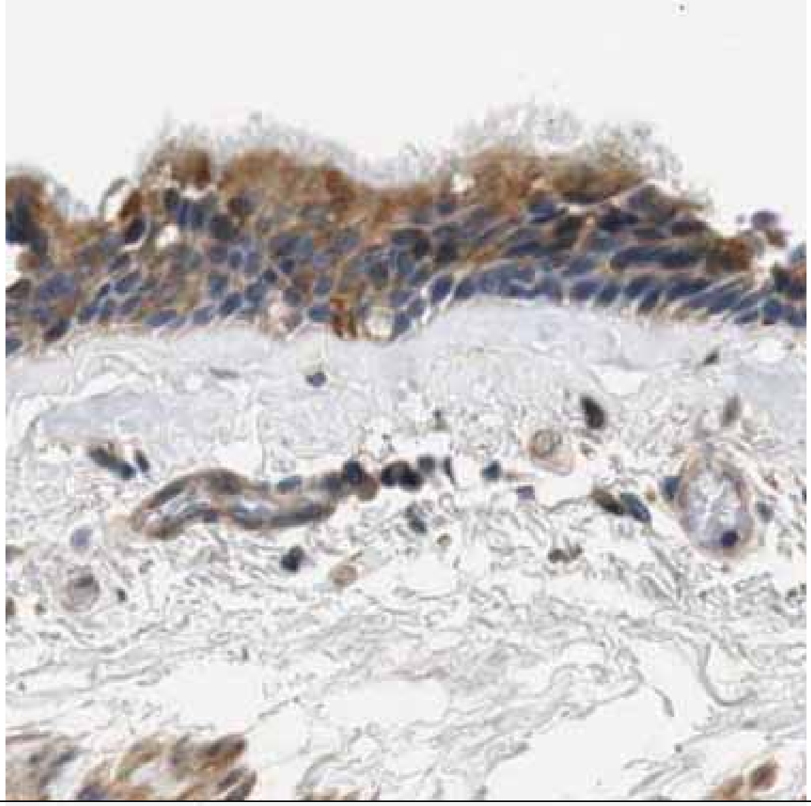	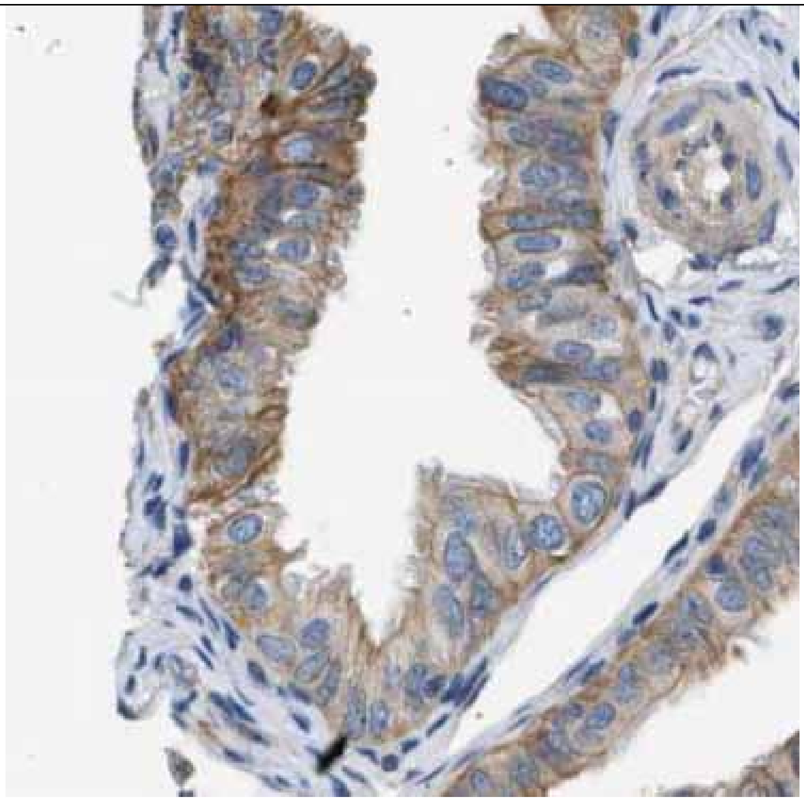	Staining is non-specific to ciliated cells or absent
ZNF20 (antibody HPA020887)<br><br>Category 2	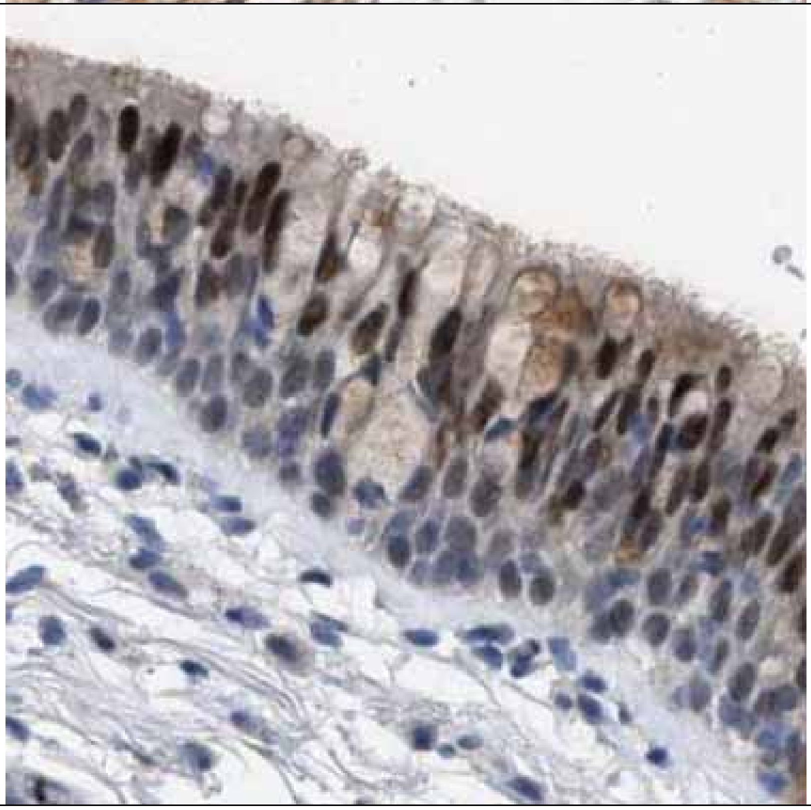	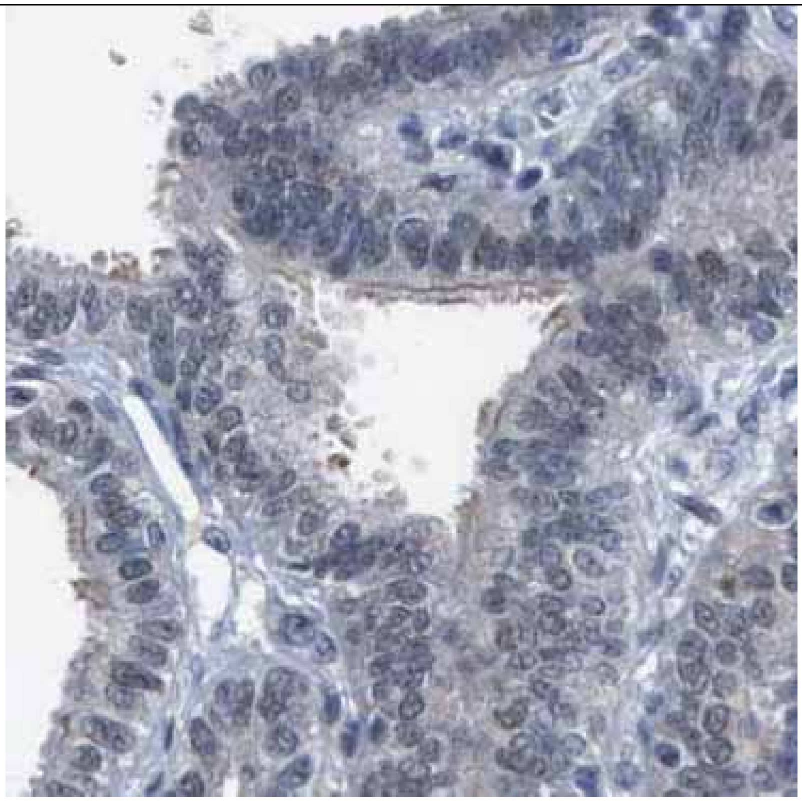	Staining is non-specific to ciliated cells or absent

Category 3

(genes with no evidence

for ciliary function from the *literature*)

<u>Coverage:</u> 48 proteins were available in Protein Atlas
from the total of 74 proteins in the category

Protein	Airways	Fallopian tubes	Summary
ARMC2 (antibody HPA025809)<br><br>Category 3	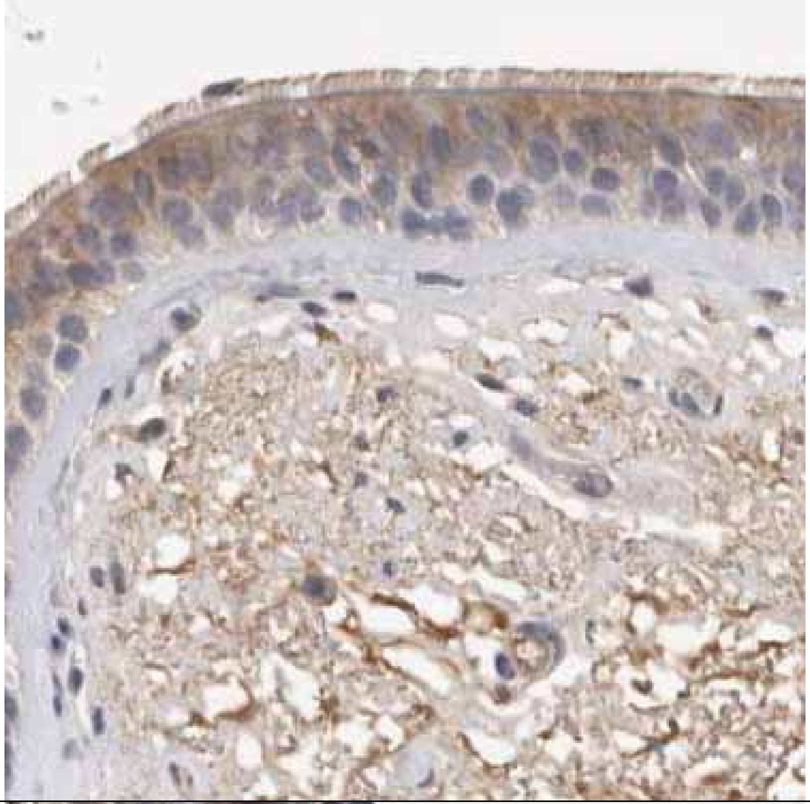	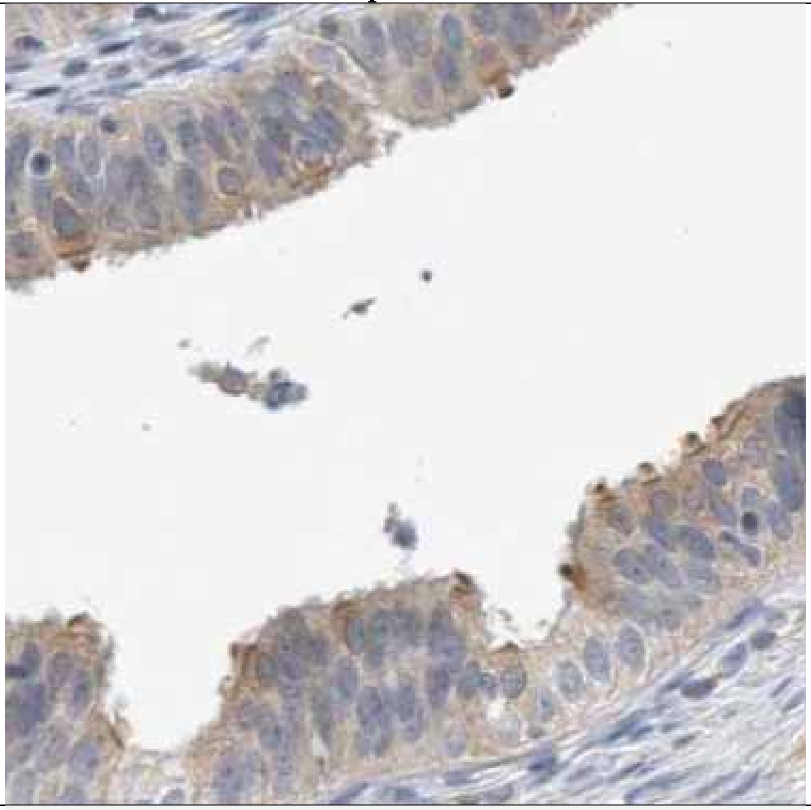	Staining is specific to *cilia*
BAIAP3 (antibody HPA015627)<br><br>Category 3	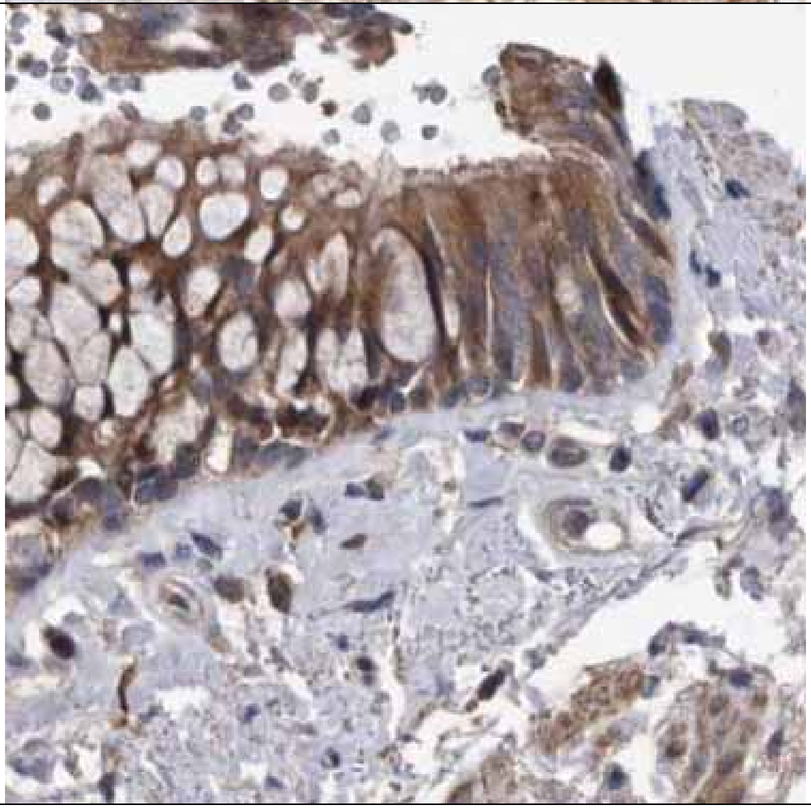	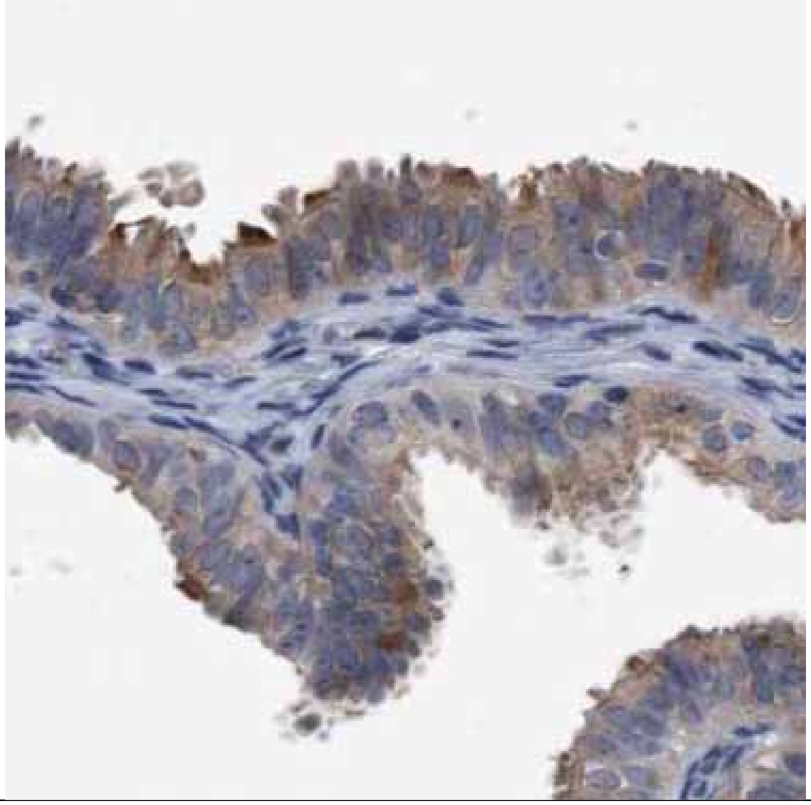	Staining is specific to *cilia*

C1orf92 (antibody HPA028442)<br><br>Category 3	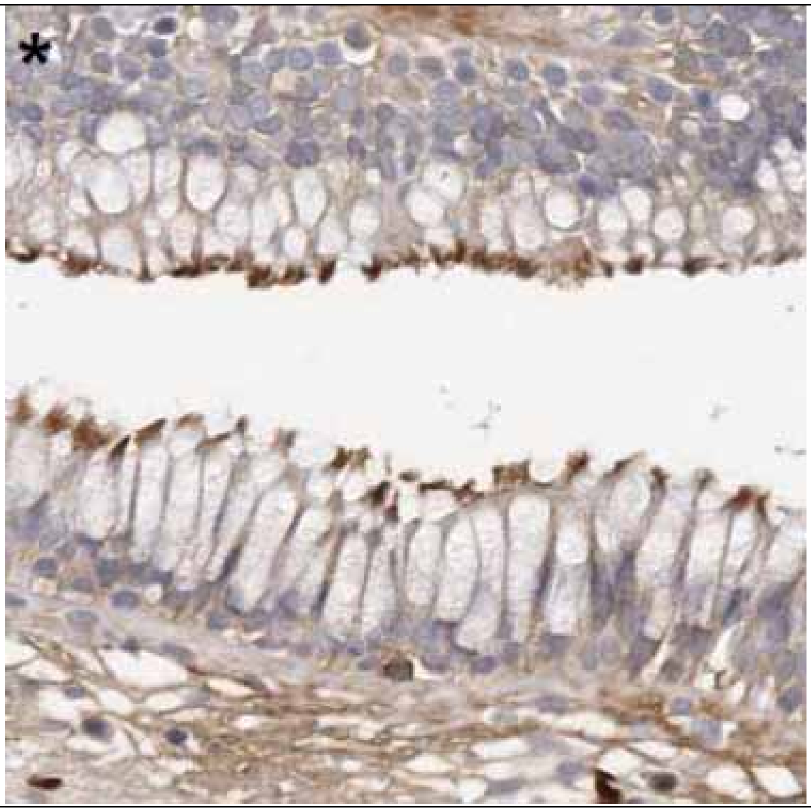	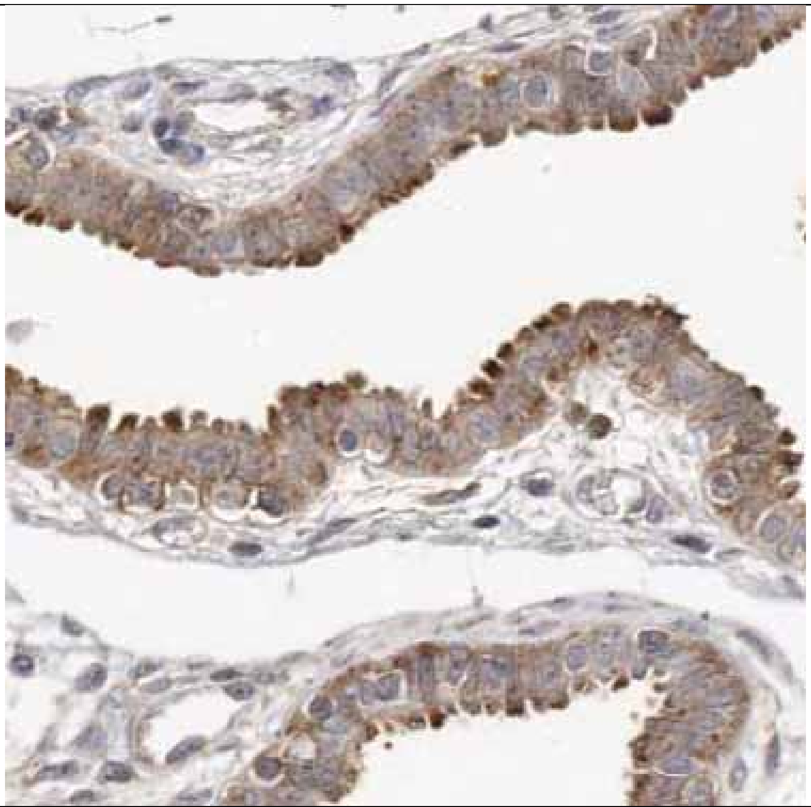	Staining is specific to *cilia*
C10orf92 (antibody HPA037786)<br><br>Category 3	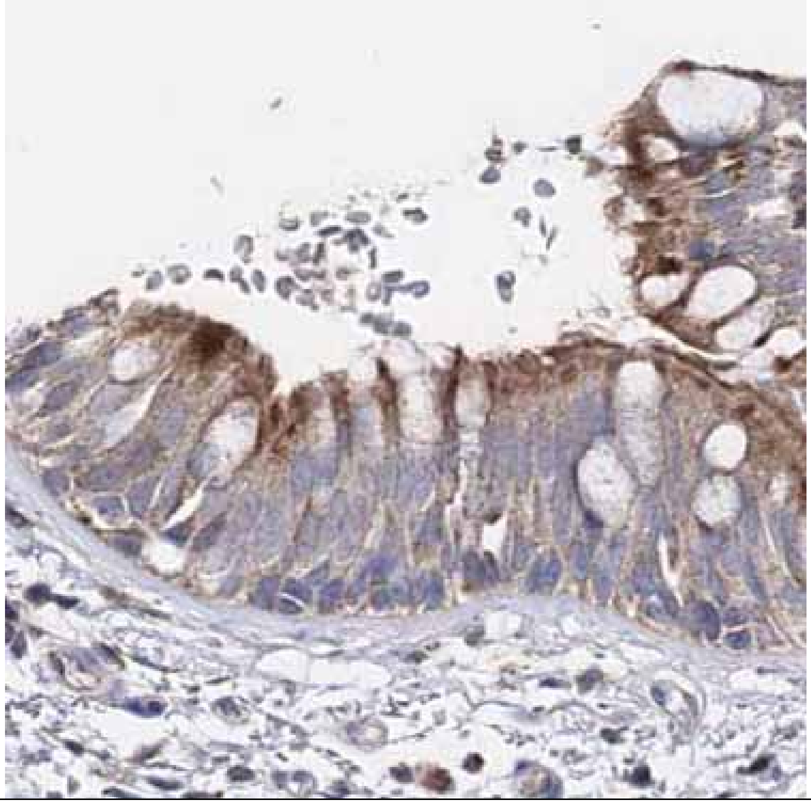	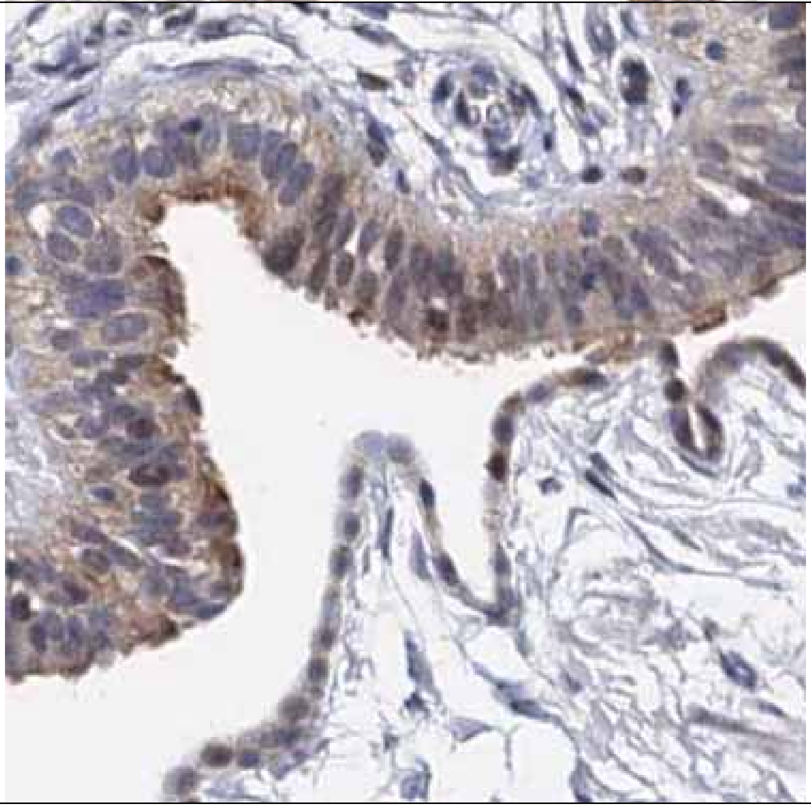	Staining is specific to *cilia*

C11orf63 (antibody HPA039612)<br><br>Category 3	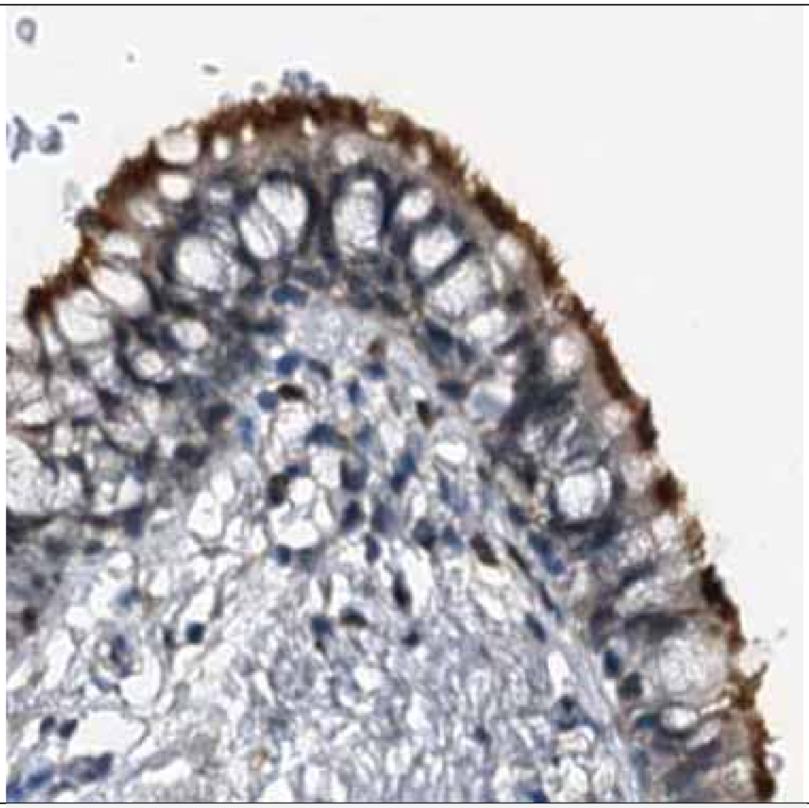	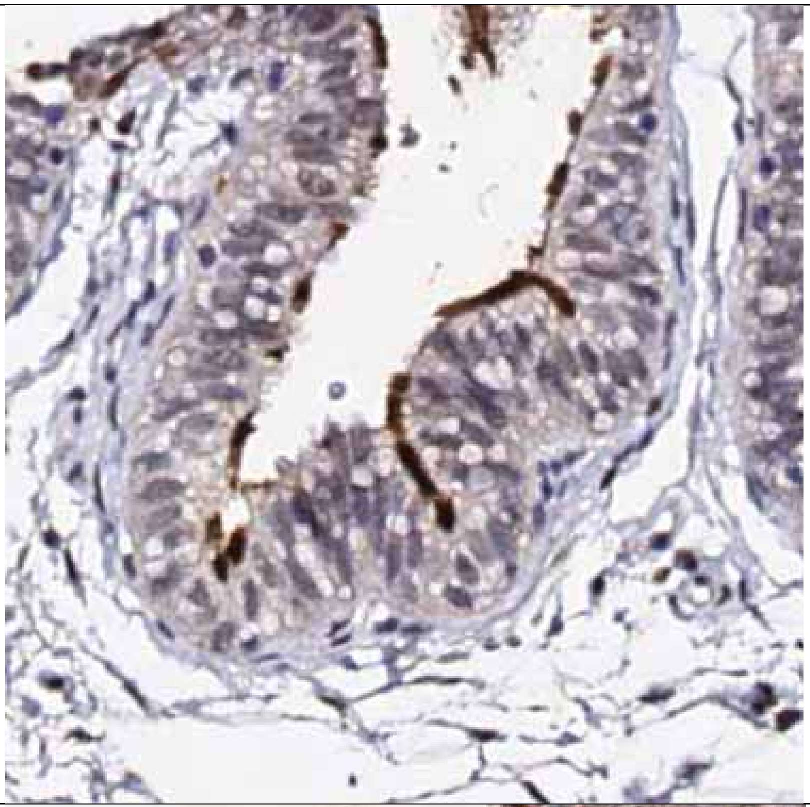	Staining is specific to *cilia*
C21orf58 (antibody HPA035110)<br><br>Category 3	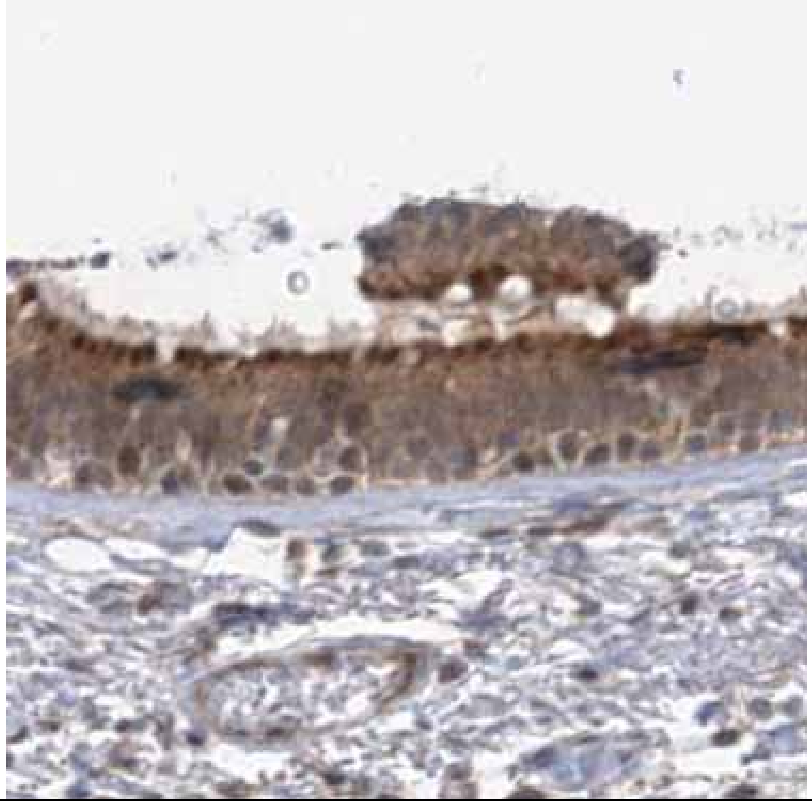	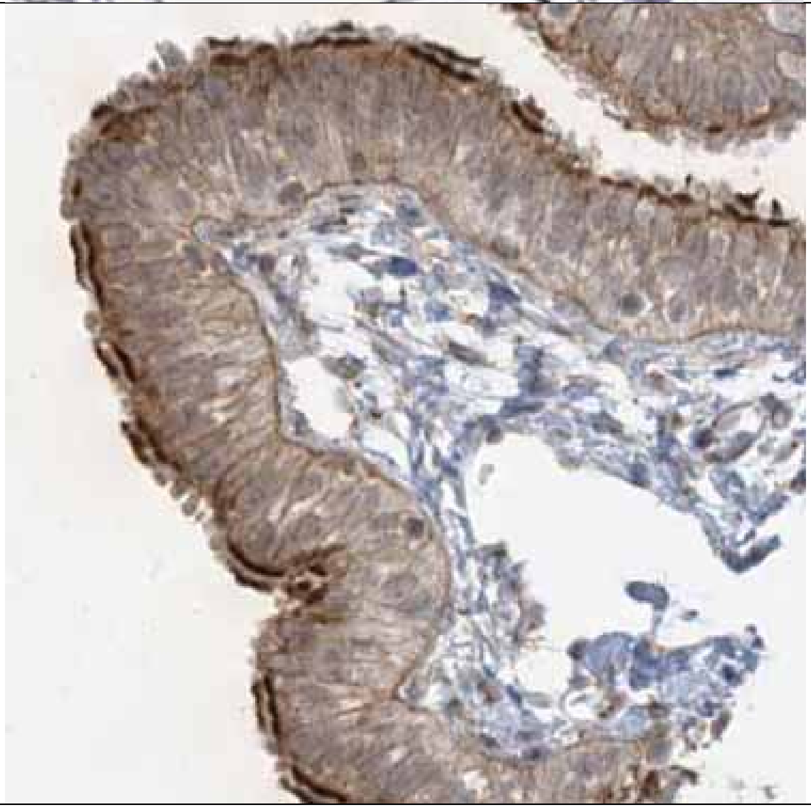	Staining is specific to *cilia*

C22orf23 (antibody HPA001149)<br><br>Category 3	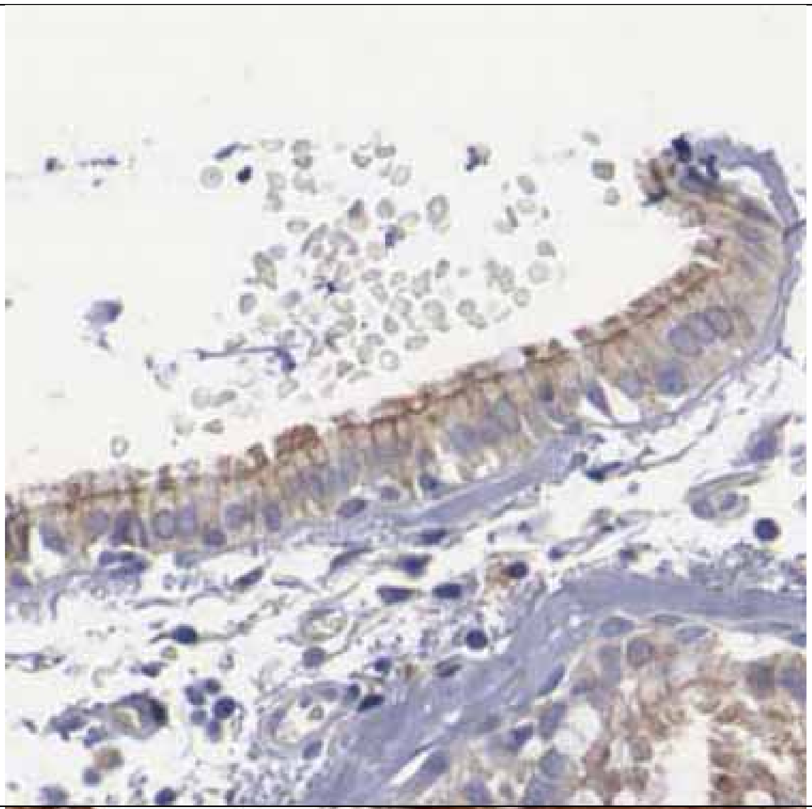	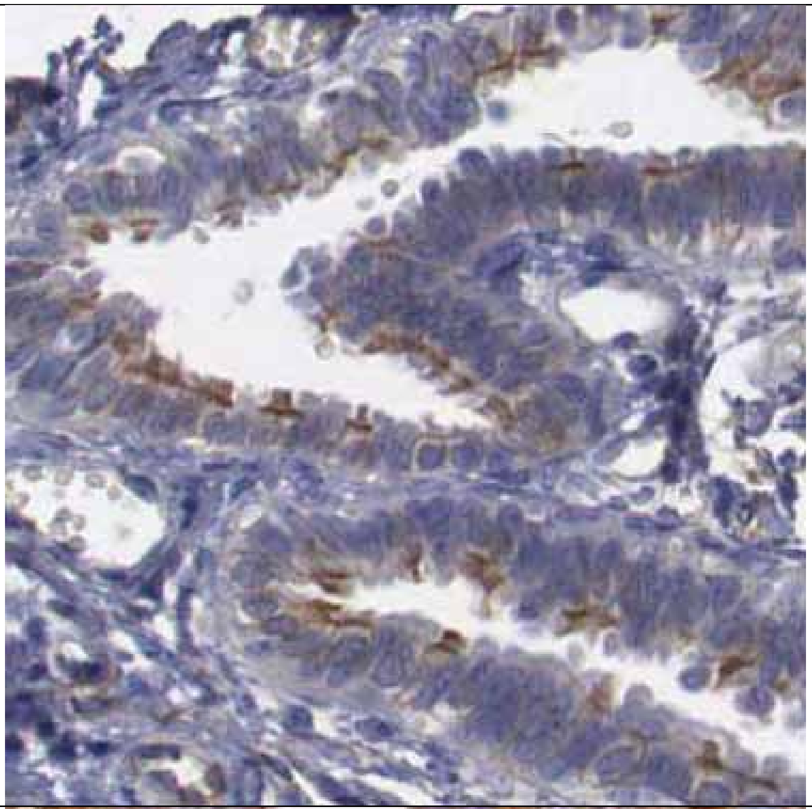	Staining is specific to *cilia*
CCDC89 (antibody HPA039351)<br><br>Category 3	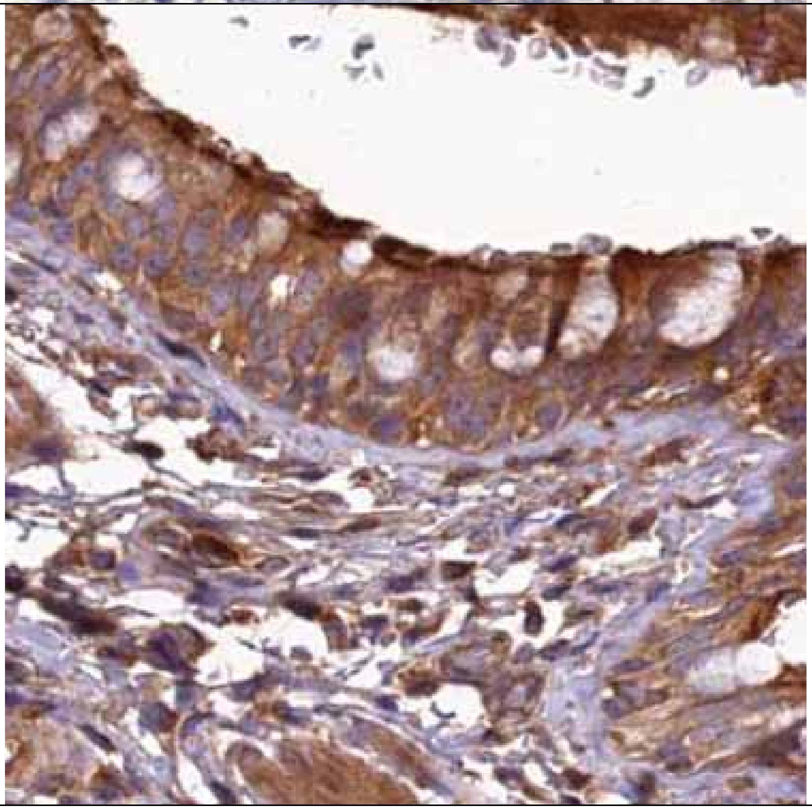	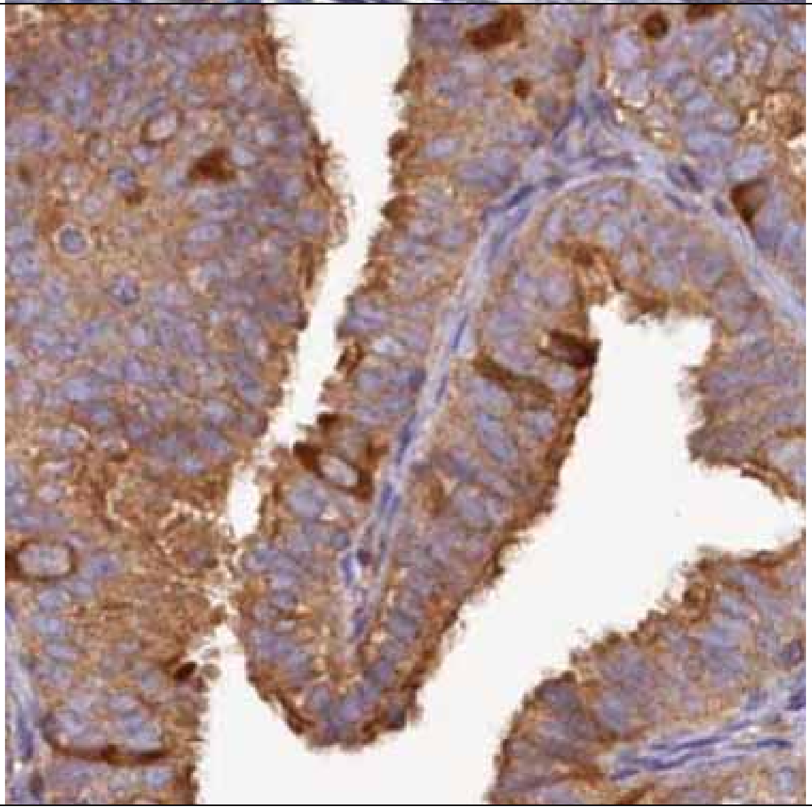	Staining is specific to *cilia*

CIB1 (antibody CAB012991)<br><br>Category 3	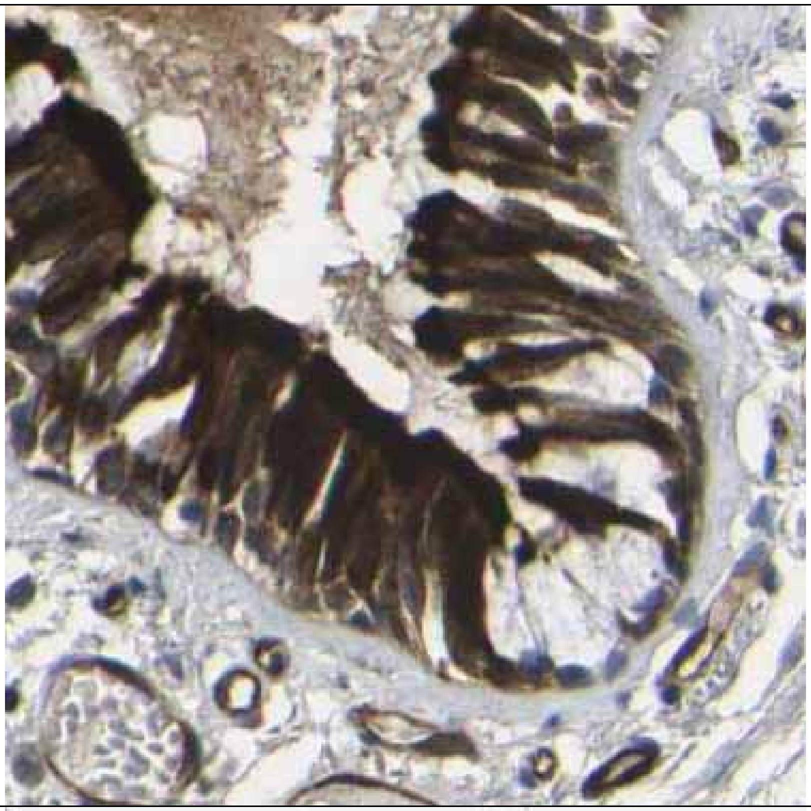	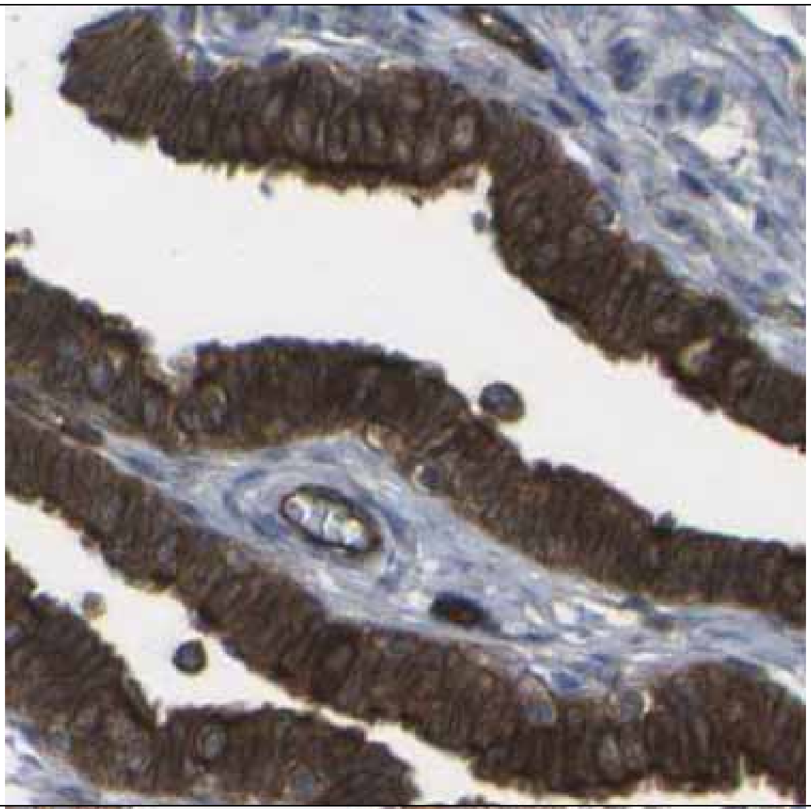	Staining is specific to *cilia*
IQCK (antibody HPA026792)<br><br>Category 3	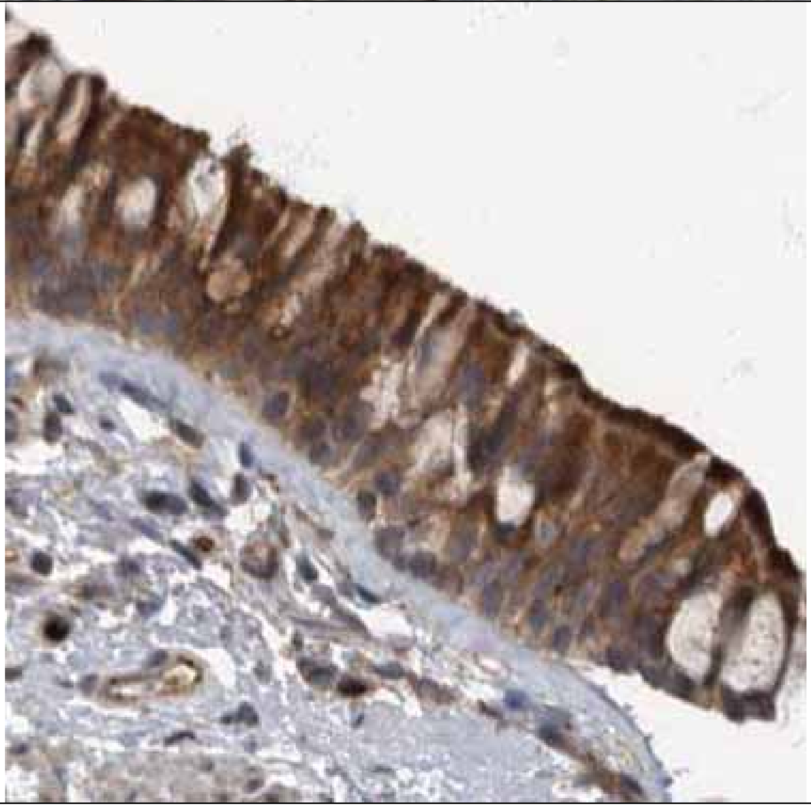	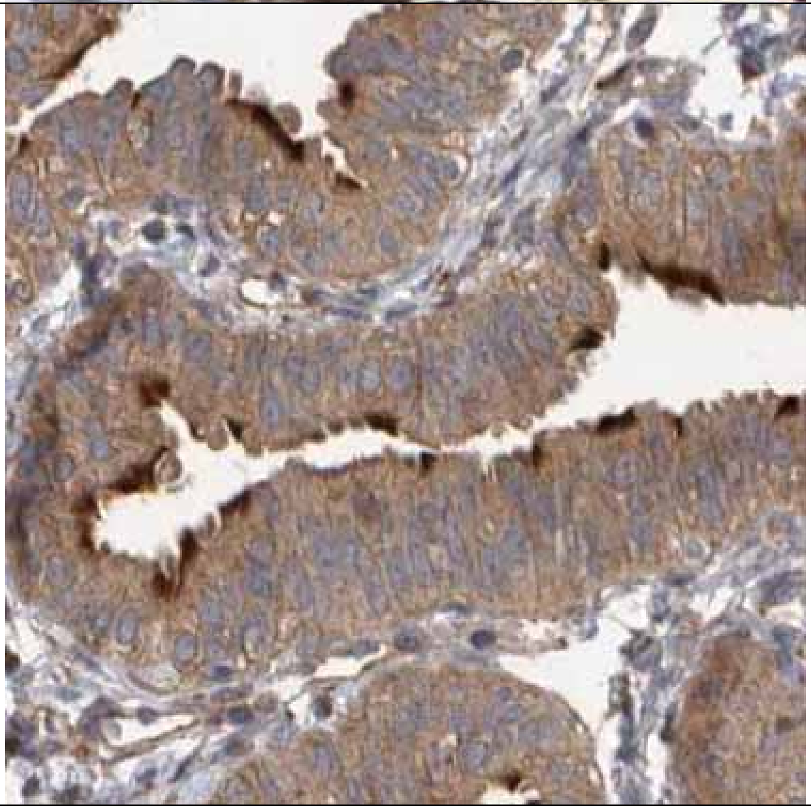	Staining is specific to *cilia*

KIAA0319 (antibody HPA015607)<br><br>Category 3	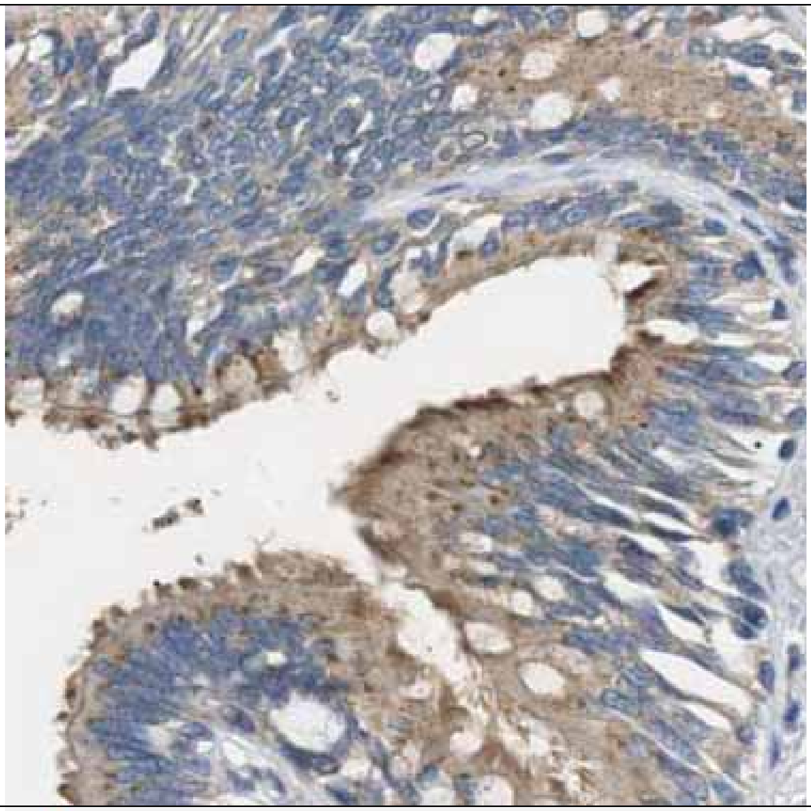	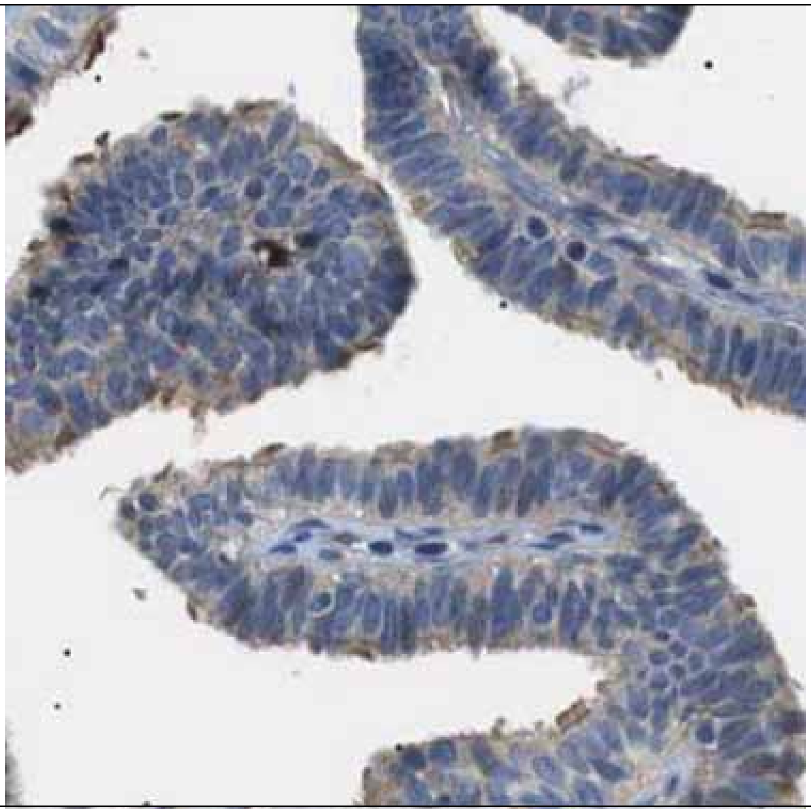	Staining is specific to *cilia*
LPAR3 (antibody HPA013421)<br><br>Category 3	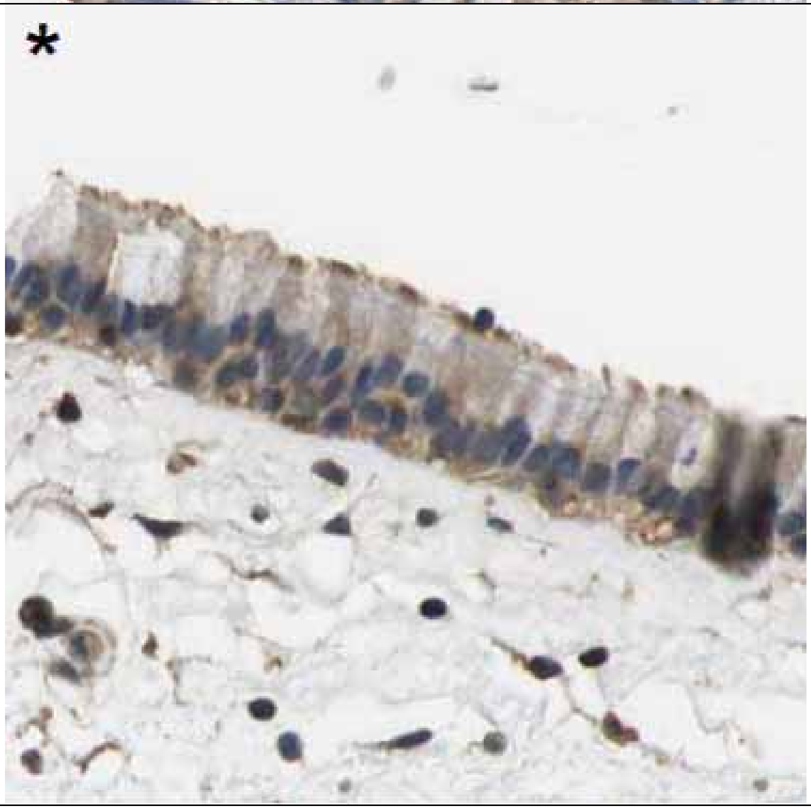	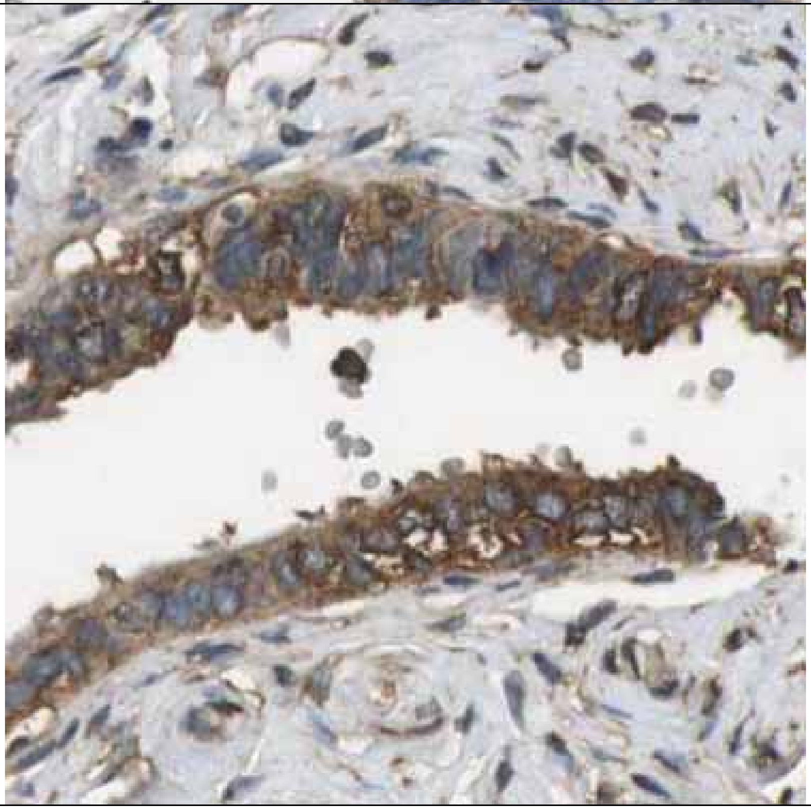	Staining is specific to *cilia*

LRGUK (antibody HPA029506)<br><br>Category 3	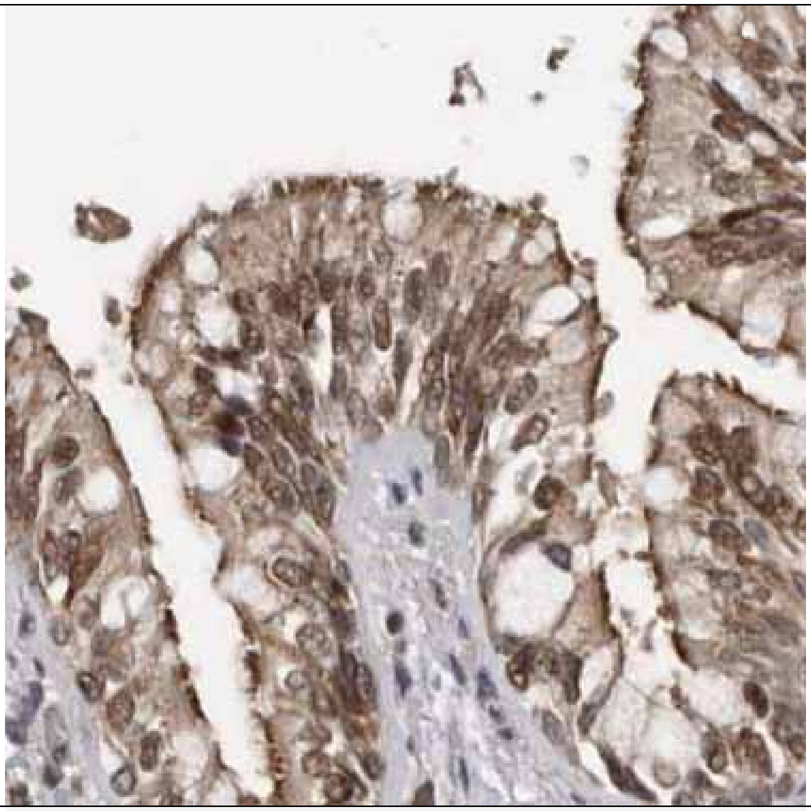	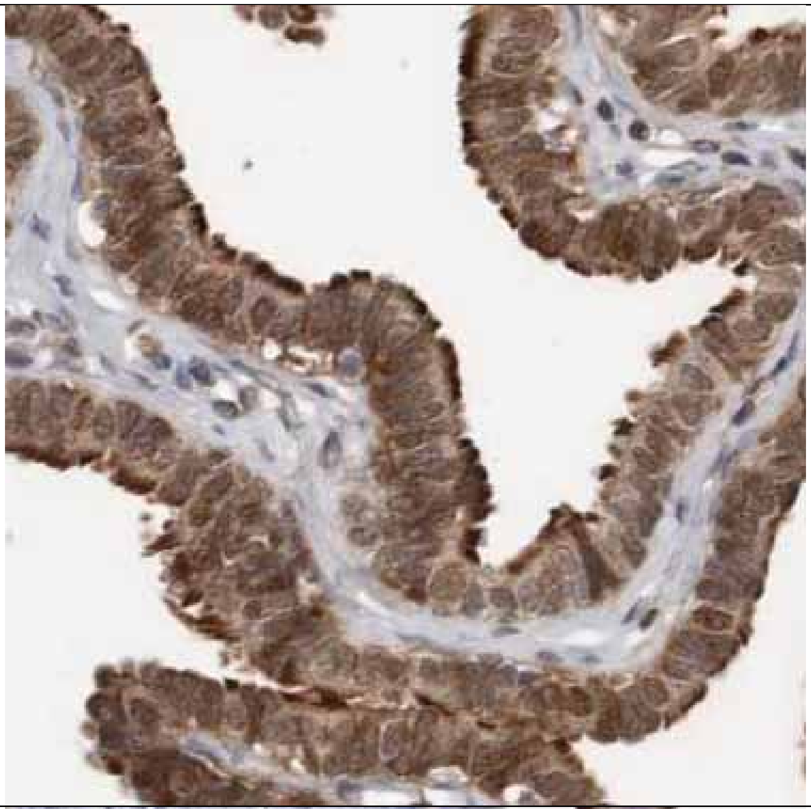	Staining is specific to *cilia*
LRRC18 (antibody HPA039256)<br><br>Category 3	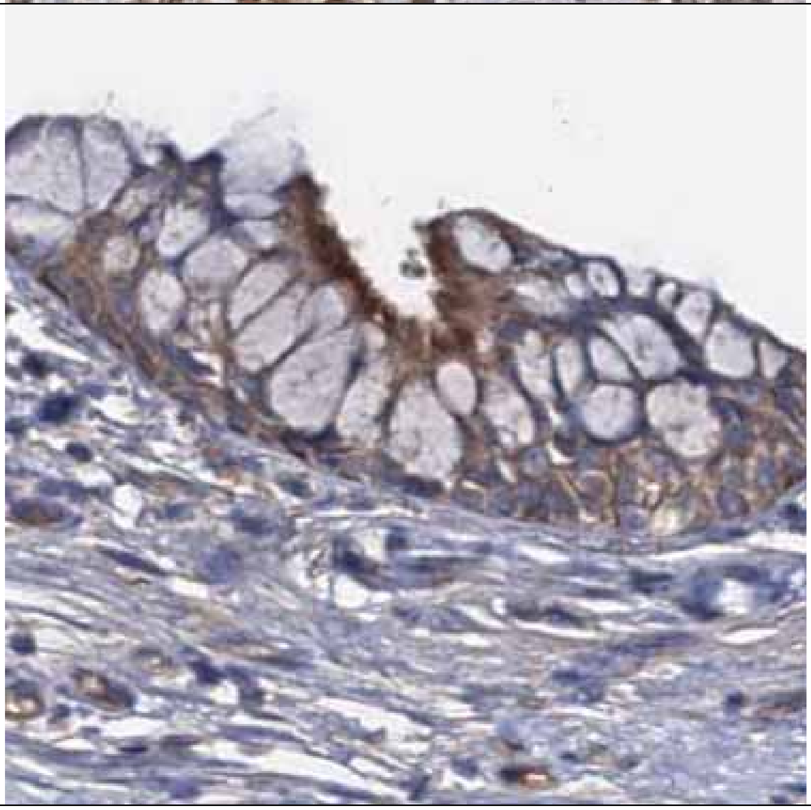	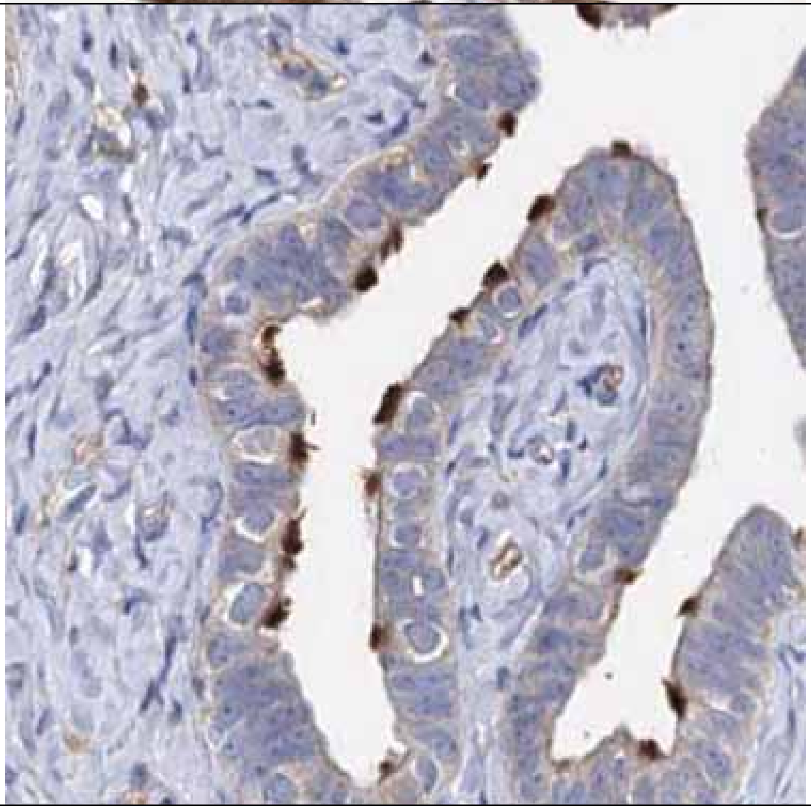	Staining is specific to *cilia*

LRRIQ3<br>(antibody)<br><br>Category 3	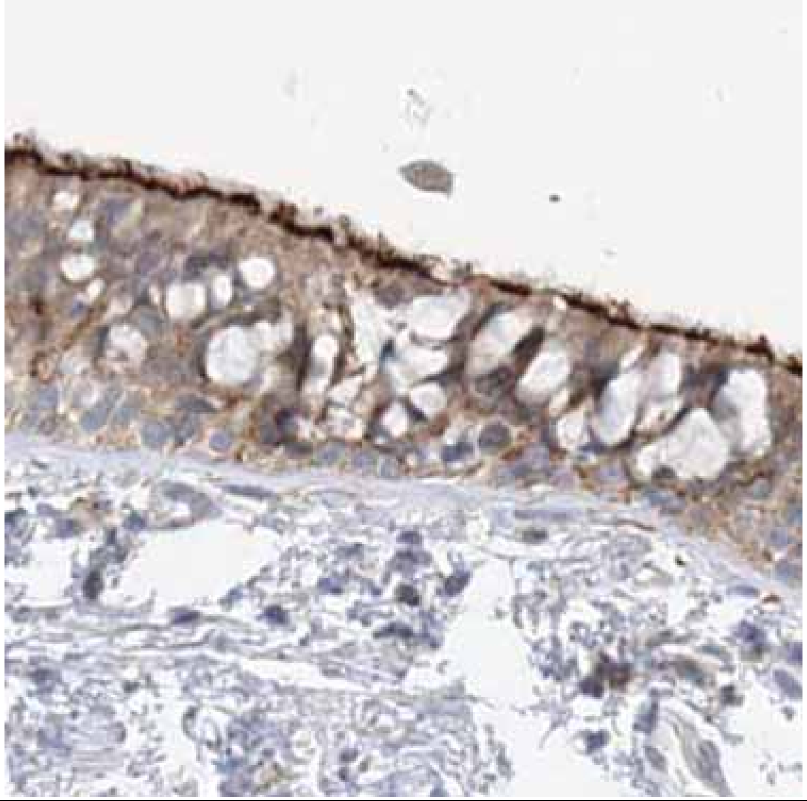	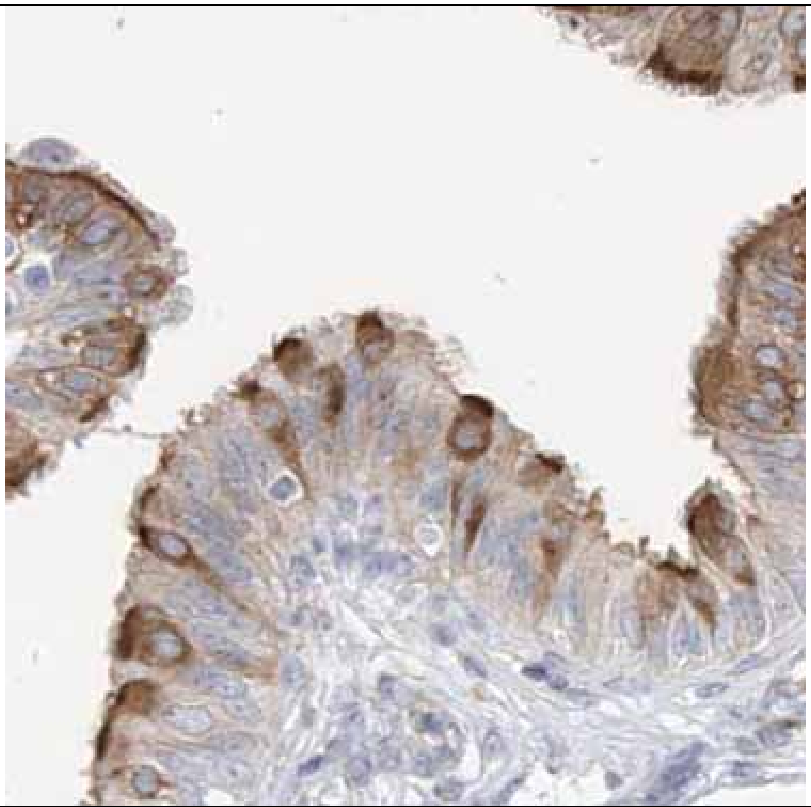	Staining is specific to *cilia*
NUP62CL<br>(antibody HPA001884)<br><br>Category 3	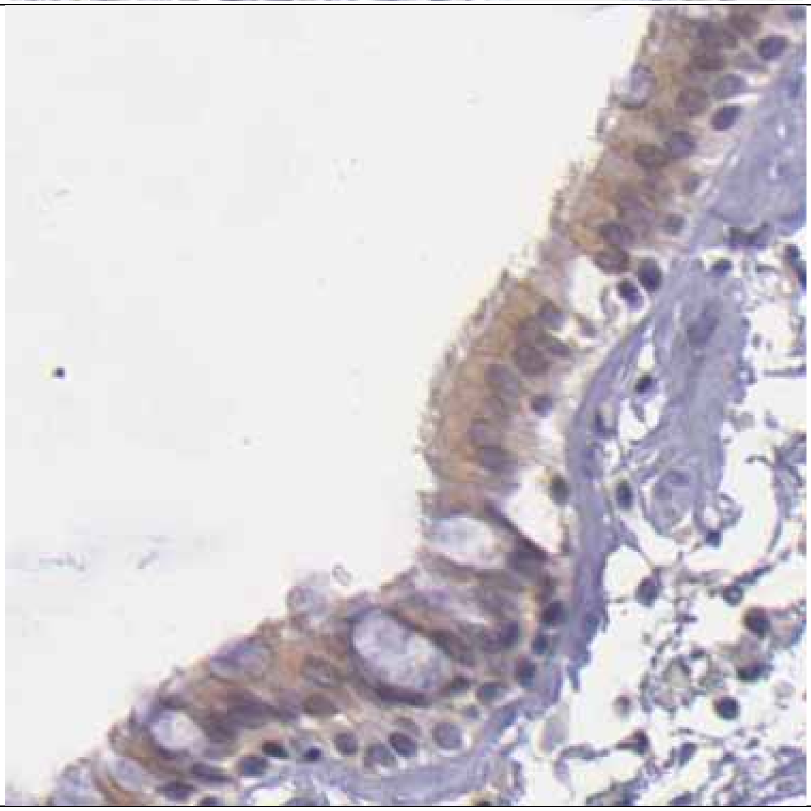	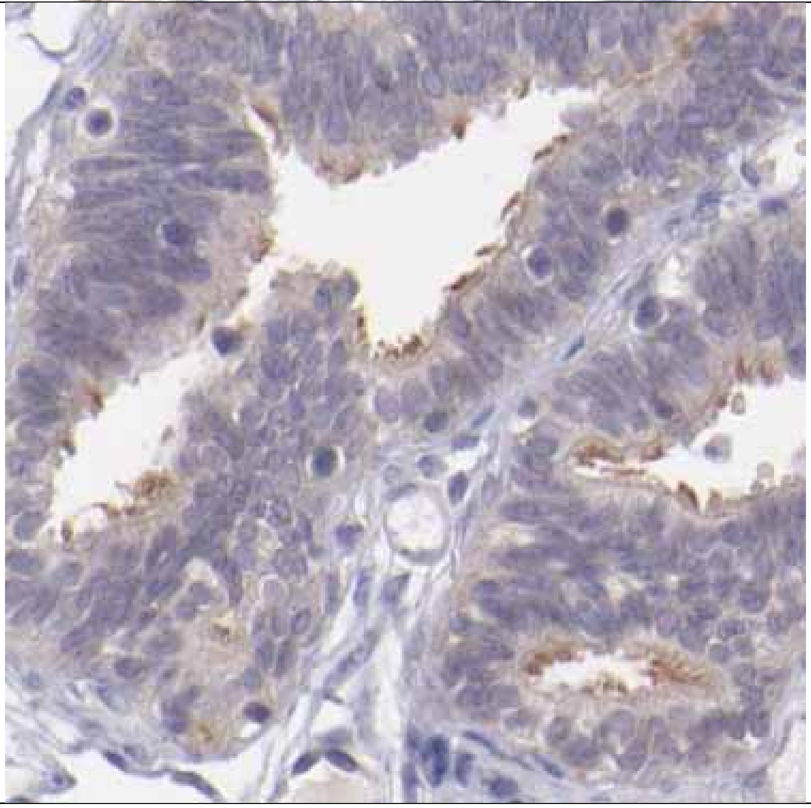	Staining is specific to *cilia*

PPM1E (antibody HPA019263)<br><br>Category 3	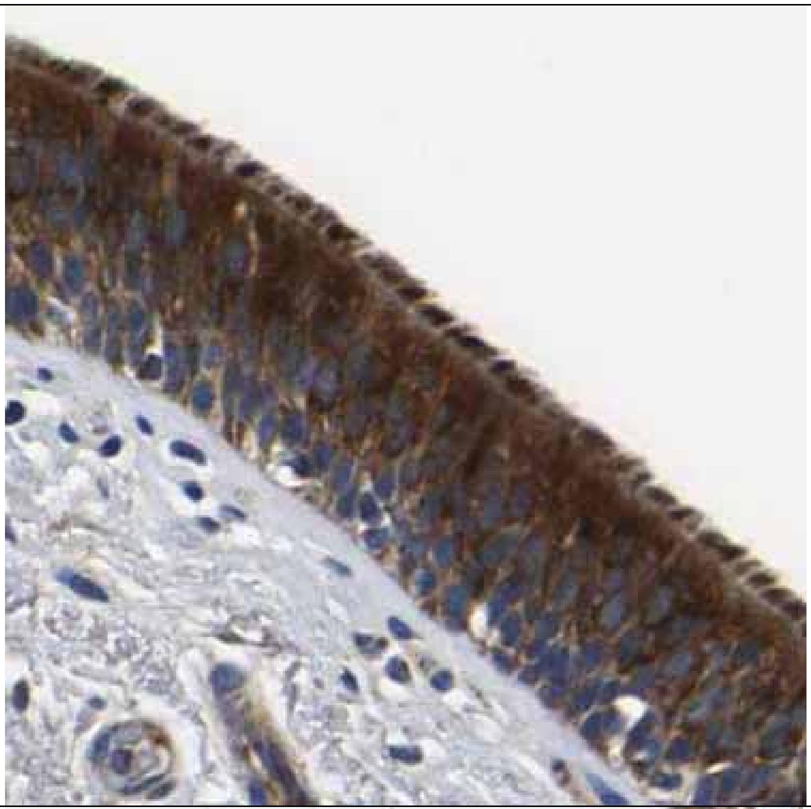	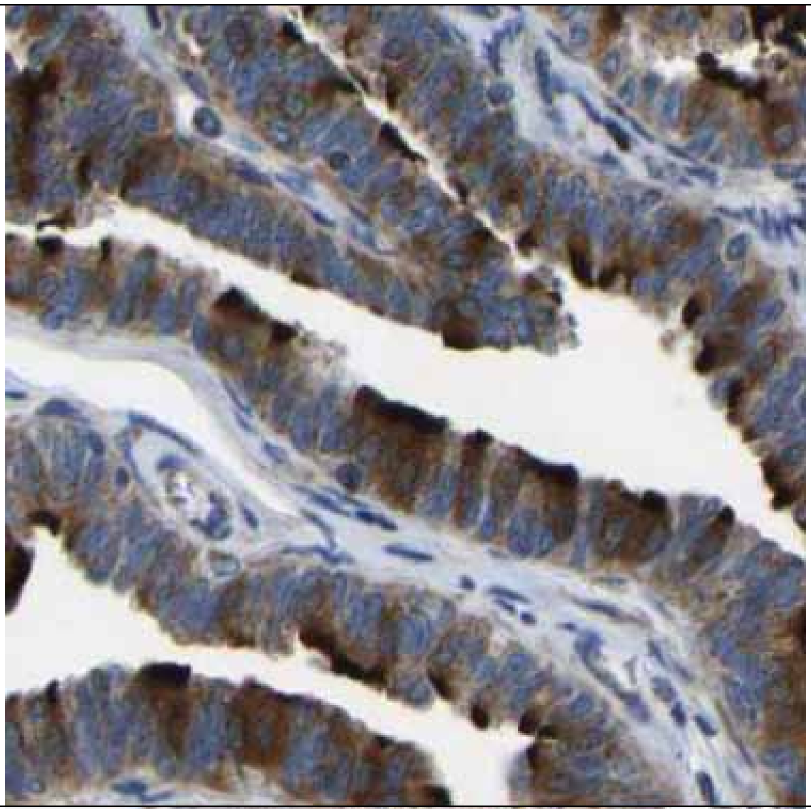	Staining is specific to *cilia*
RBM20 (antibody HPA035806)<br><br>Category 3	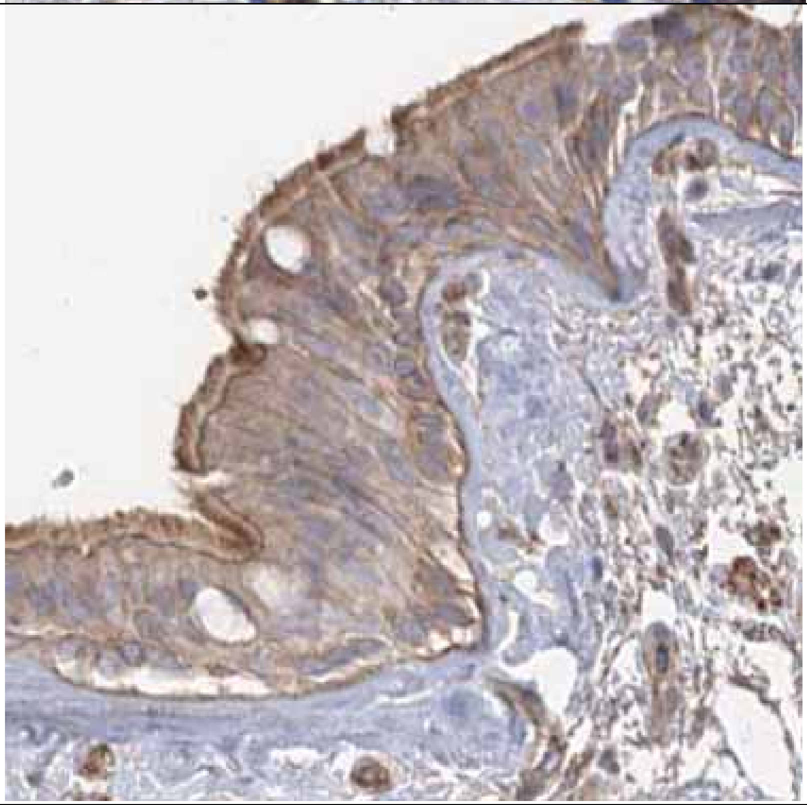	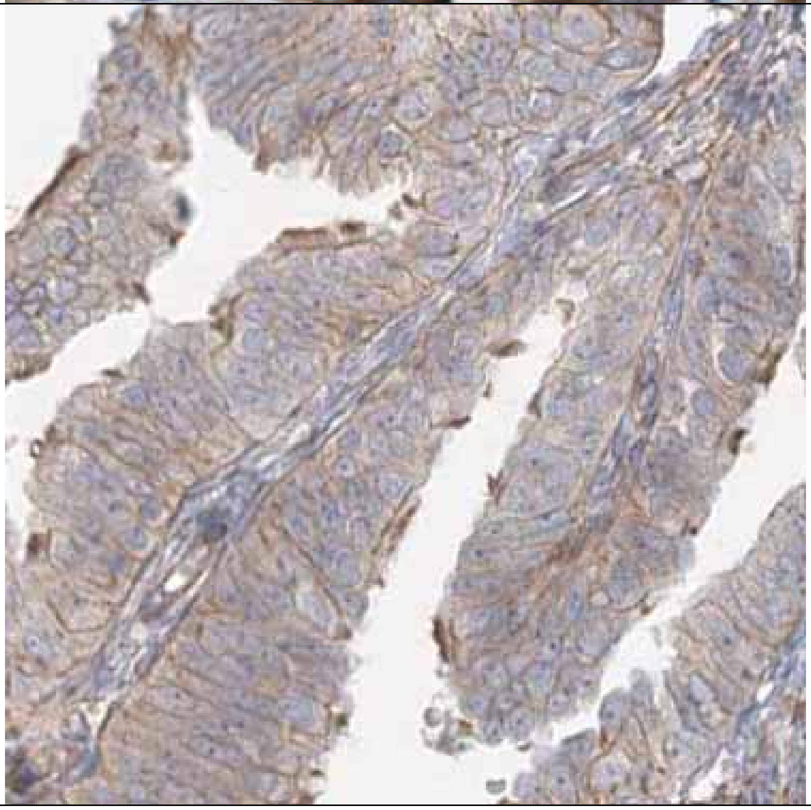	Staining is specific to *cilia*

RGS22 (antibody HPA023118)<br><br>Category 3	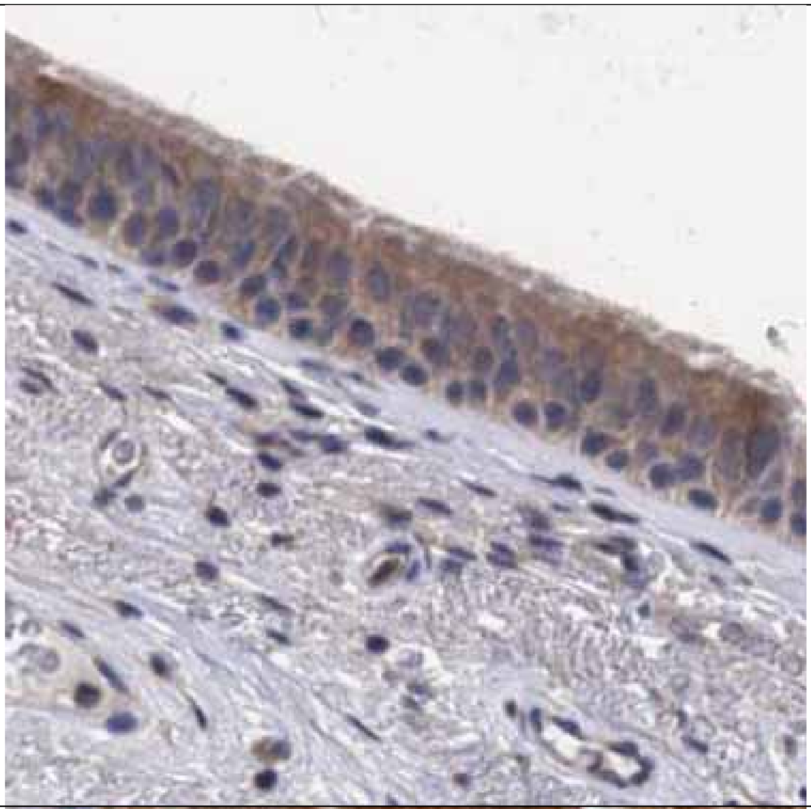	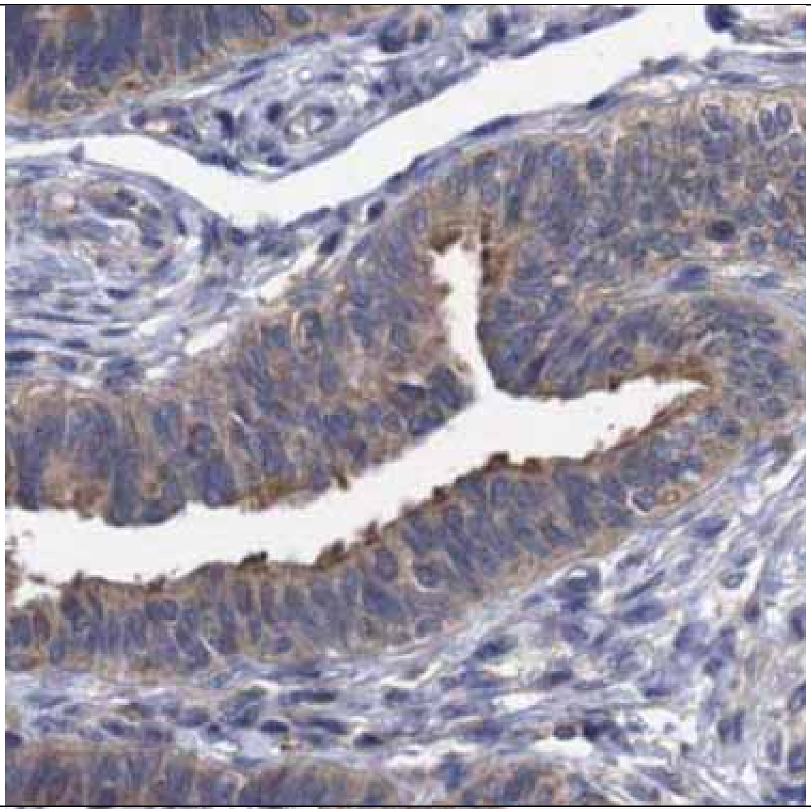	Staining is specific to *cilia*
UFC1 (antibody HPA028722)<br><br>Category 3	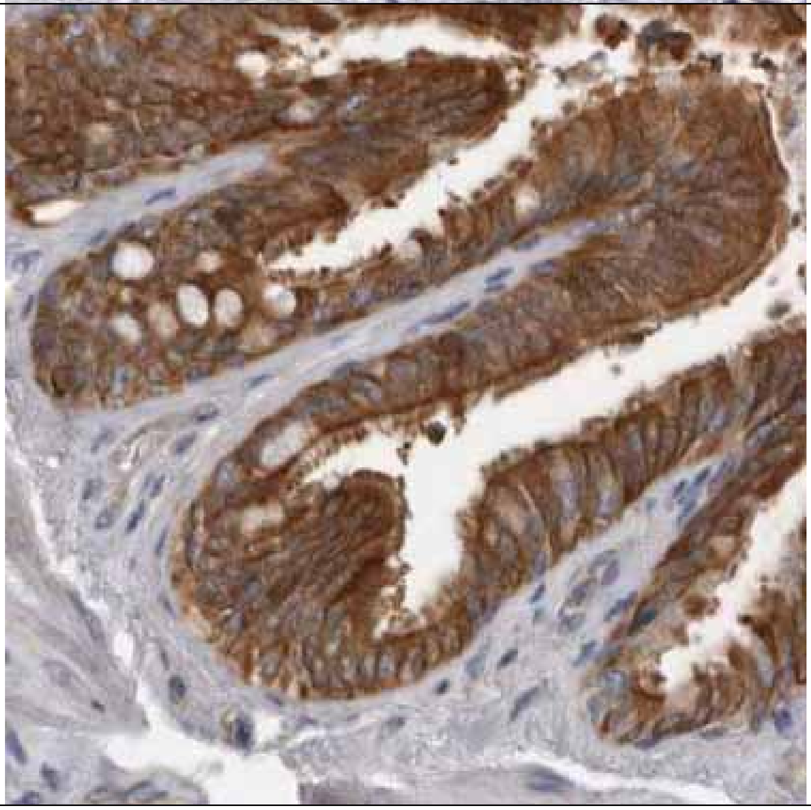	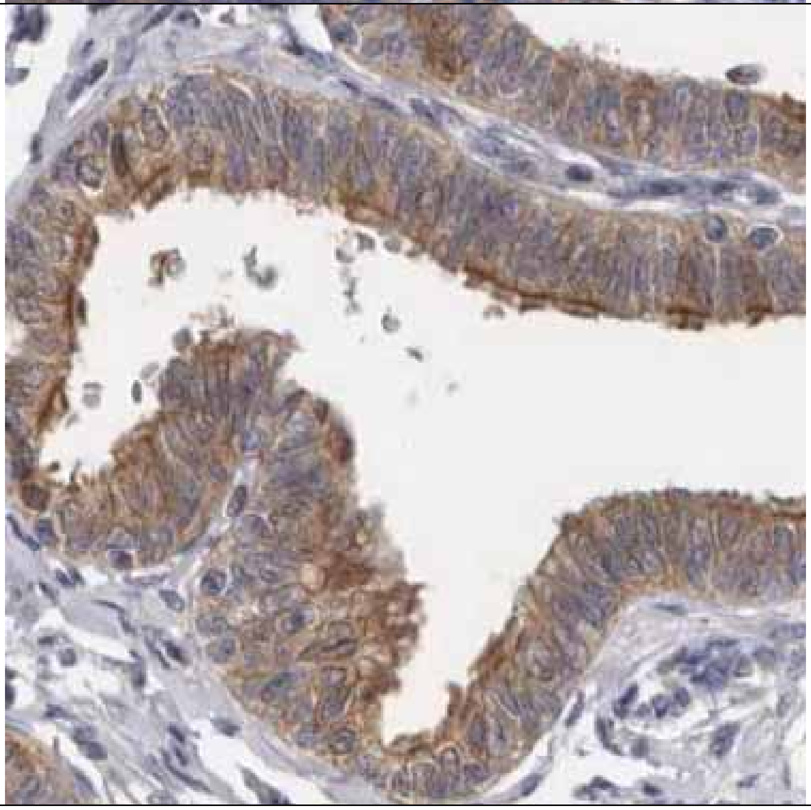	Staining is specific to *cilia*

ACYP1 (antibody HPA034944)<br><br>Category 3	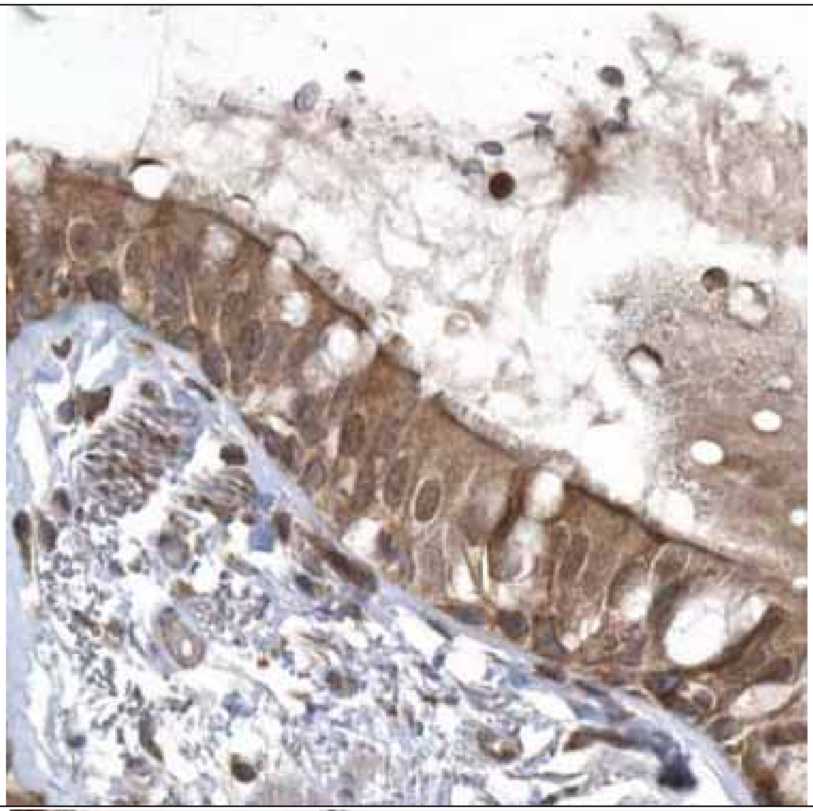	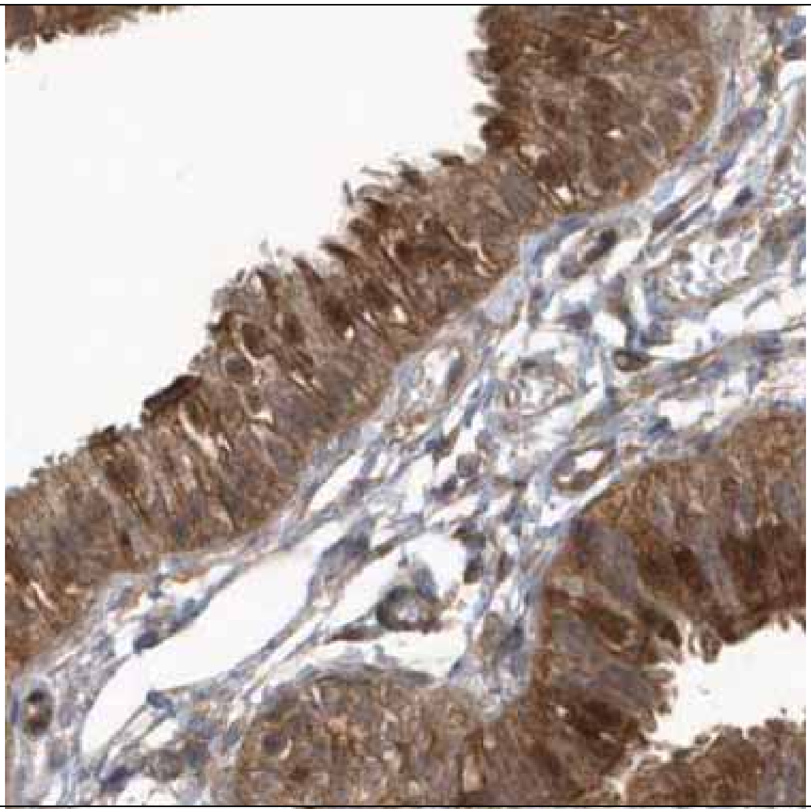	Staining is specific to *ciliated cells* (uncertain subcellular localization)
C1orf129 (antibody HPA028413)<br><br>Category 3	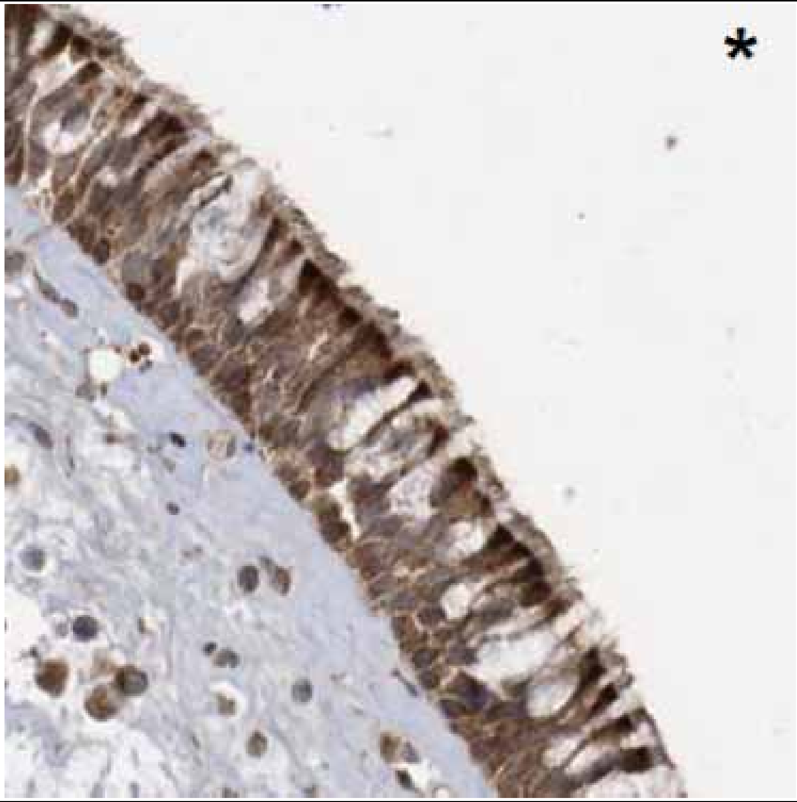	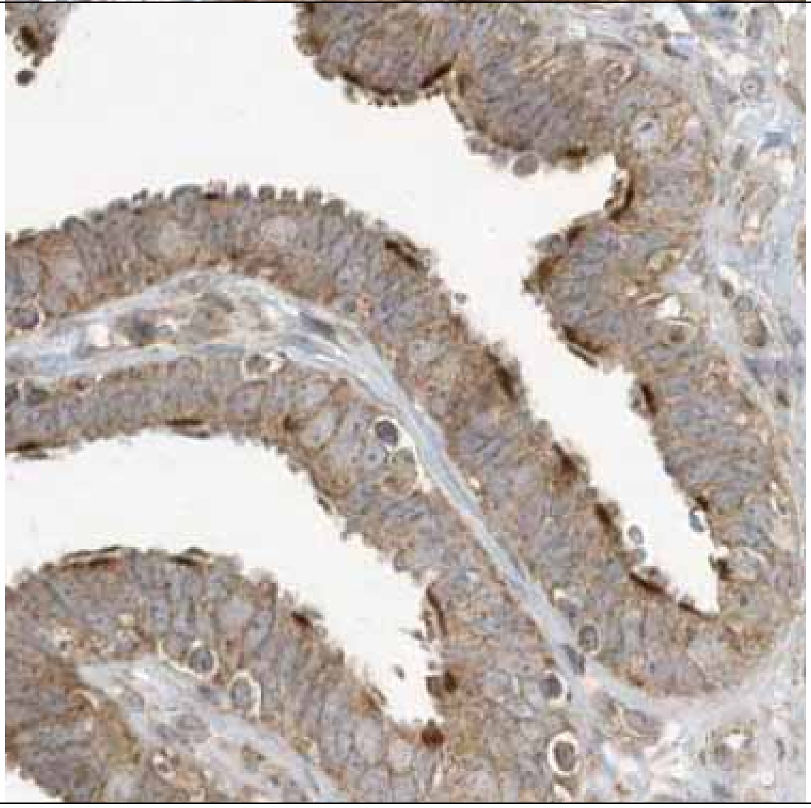	Staining is specific to *ciliated cells* (apical cytoplasm)

DCDC5 (antibody HPA039014)<br><br>Category 3	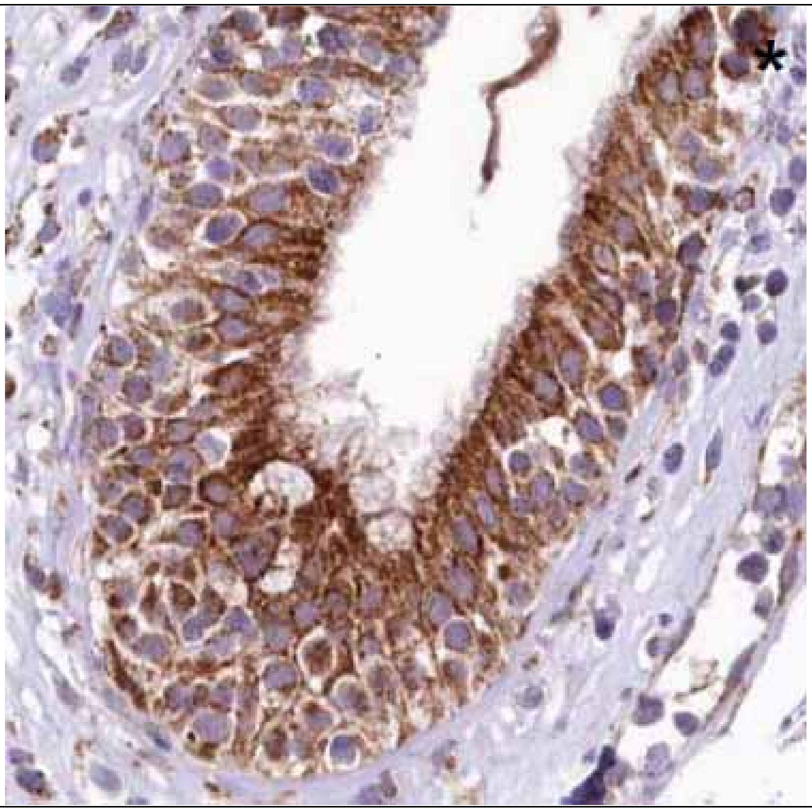	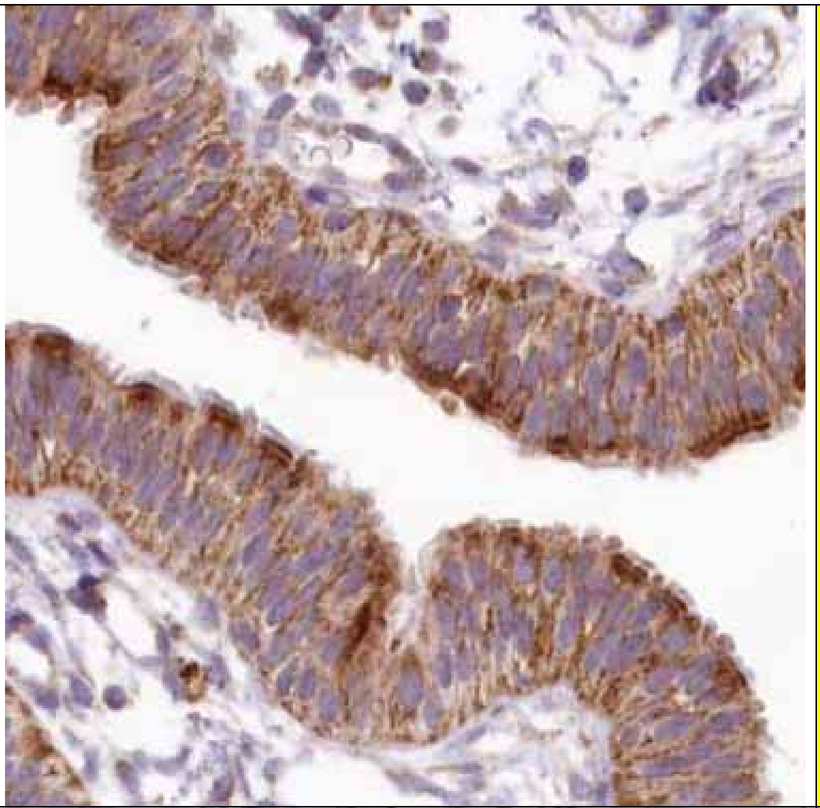	Staining is specific to *ciliated cells* (apical cytoplasm)
FLJ16686 (antibody HPA037761)<br><br>Category 3	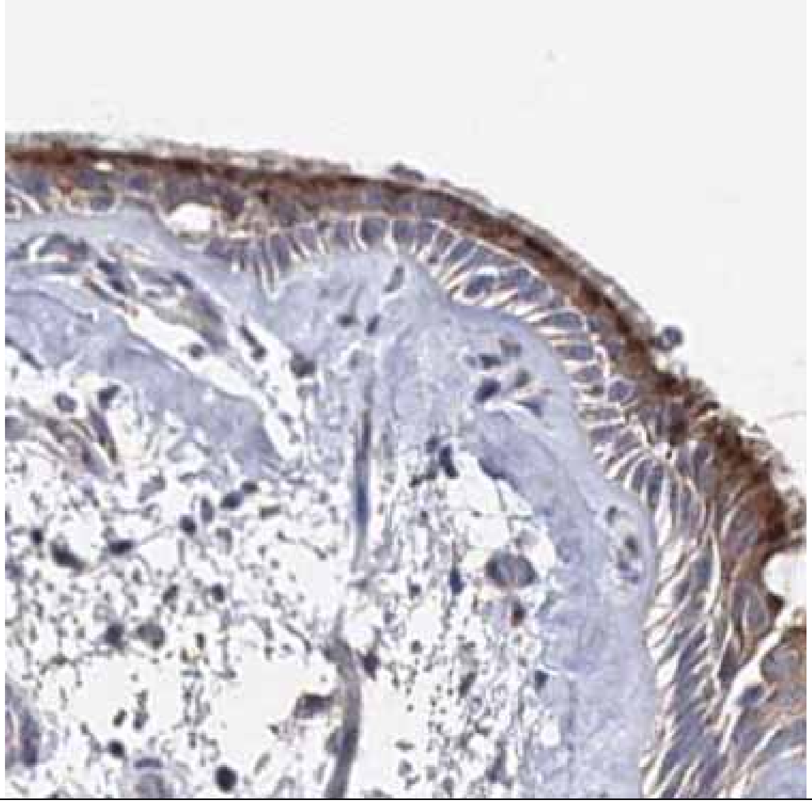	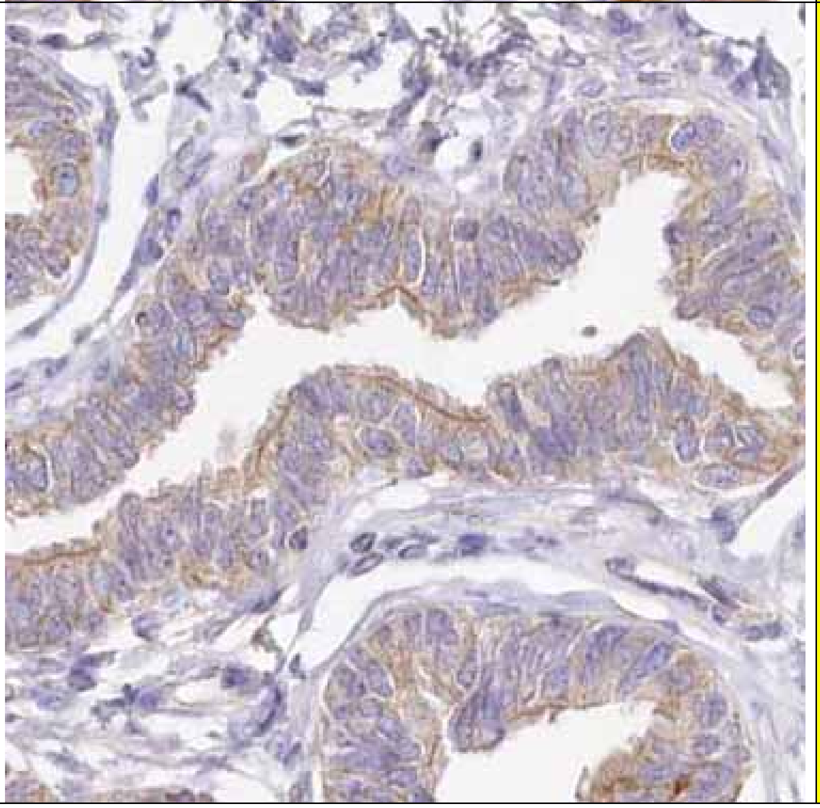	Staining is specific to *ciliated cells* (apical cytoplasm)

SYTL3 (antibody HPA030586)<br><br>Category 3	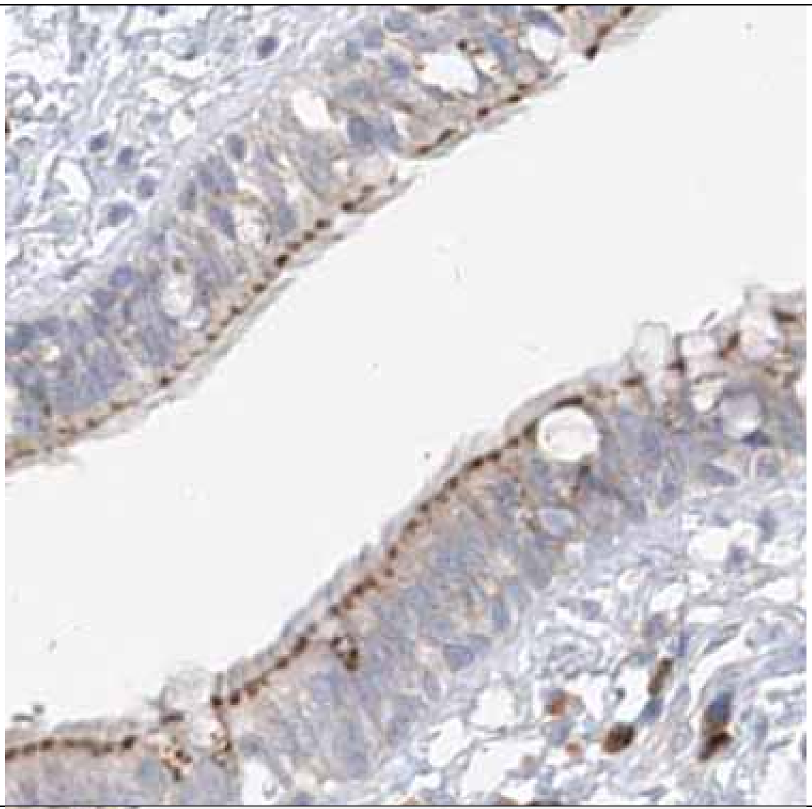	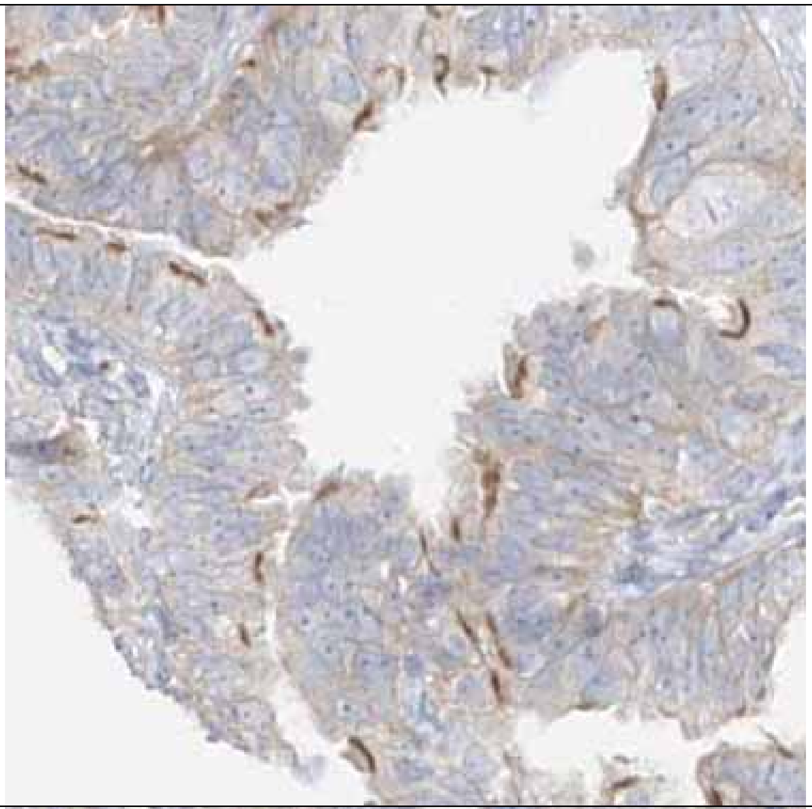	Staining is specific to *ciliated cells* (apical cytoplasm)
ALS2CR12 (antibody HPA035793)<br><br>Category 3	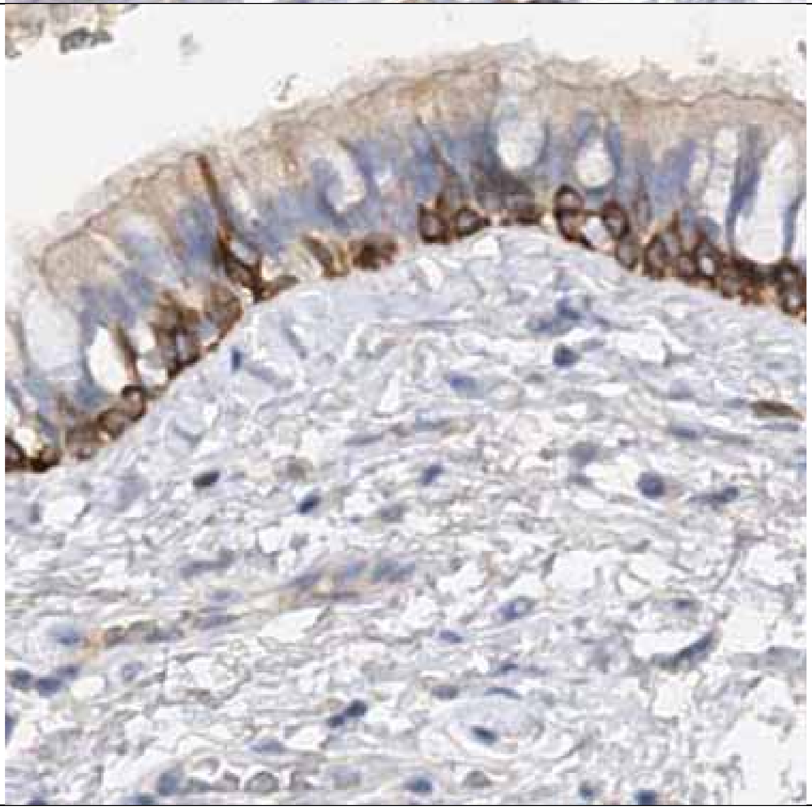	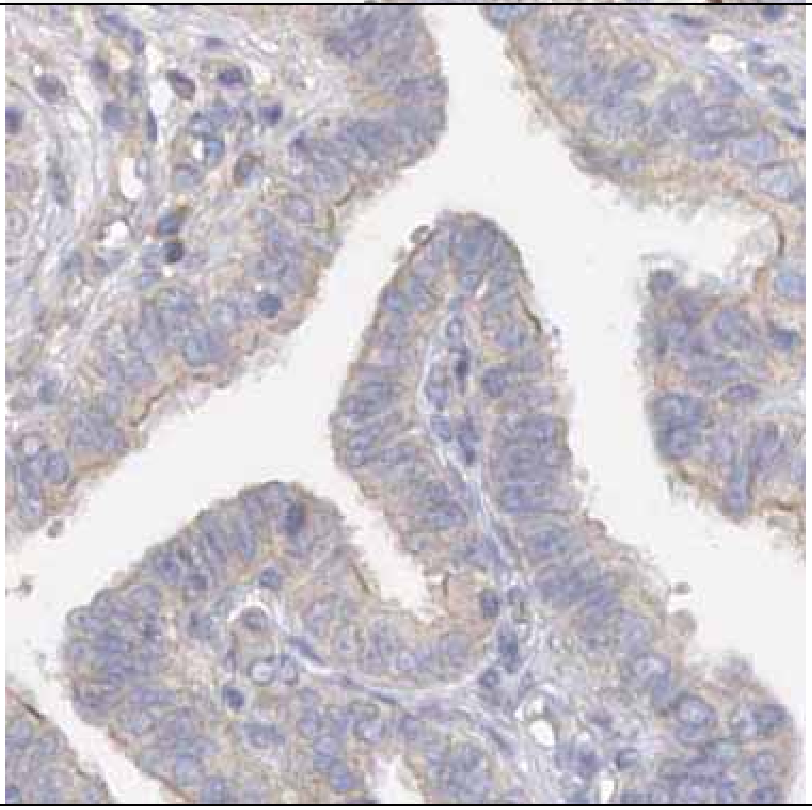	Staining is non-specific to ciliated cells or absent

ANKFN1 (antibody HPA021566)<br><br>Category 3	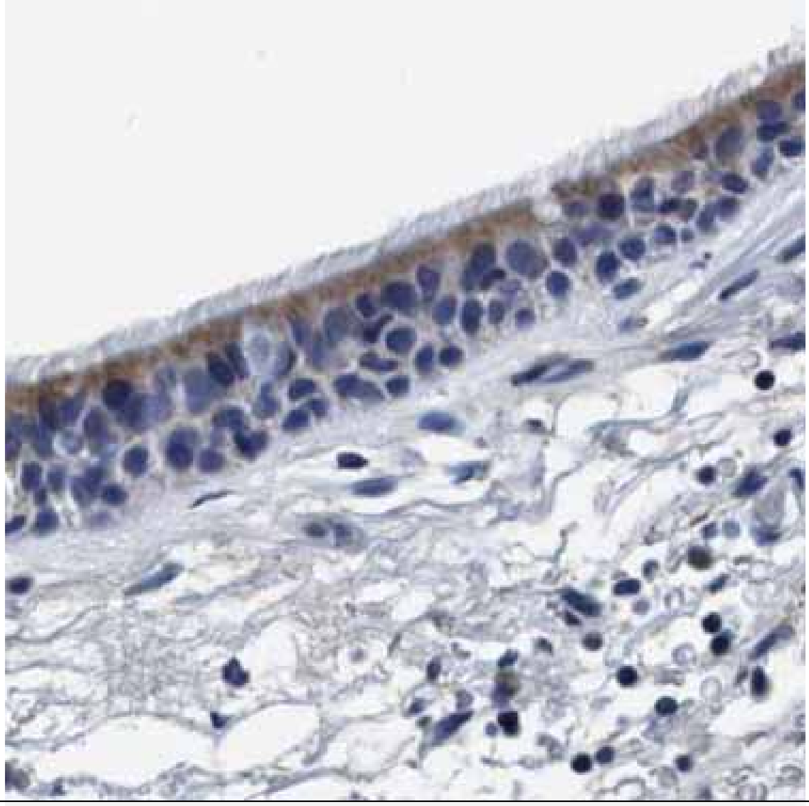	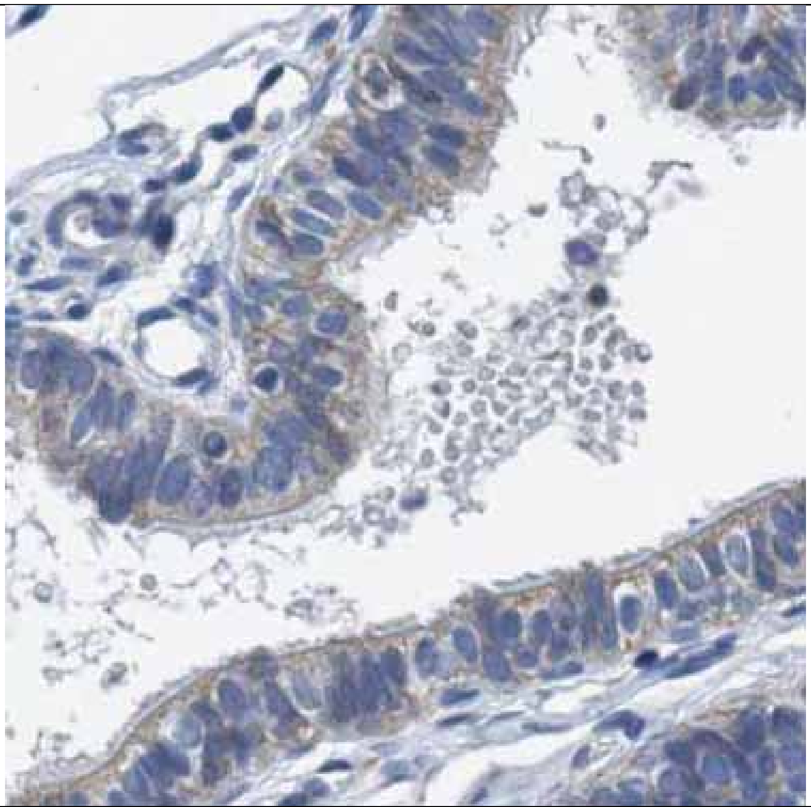	Staining is non-specific to ciliated cells or absent
CCDC67 (antibody HPA010986)<br><br>Category 3	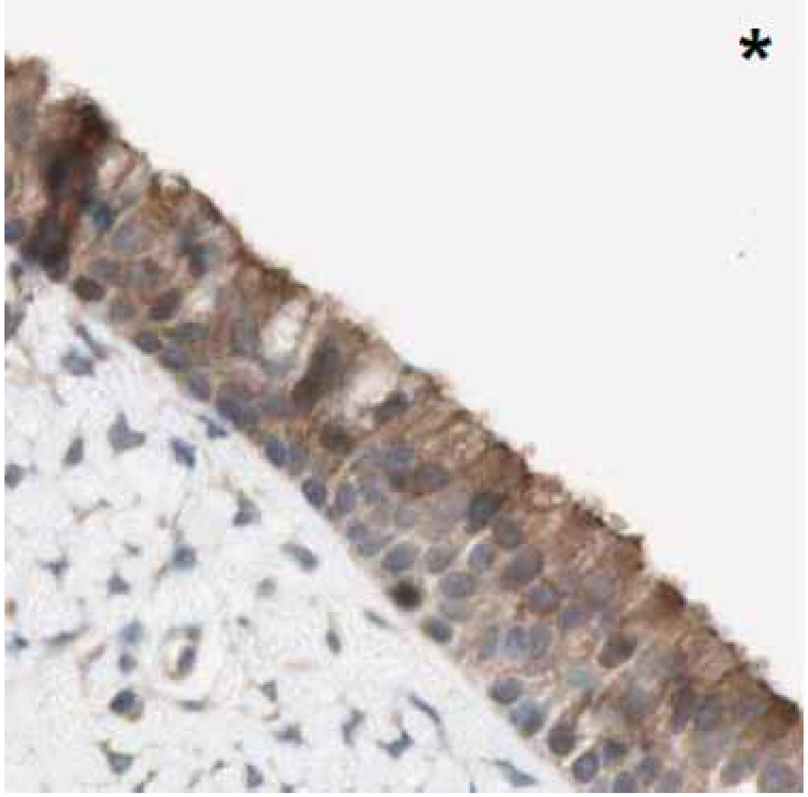	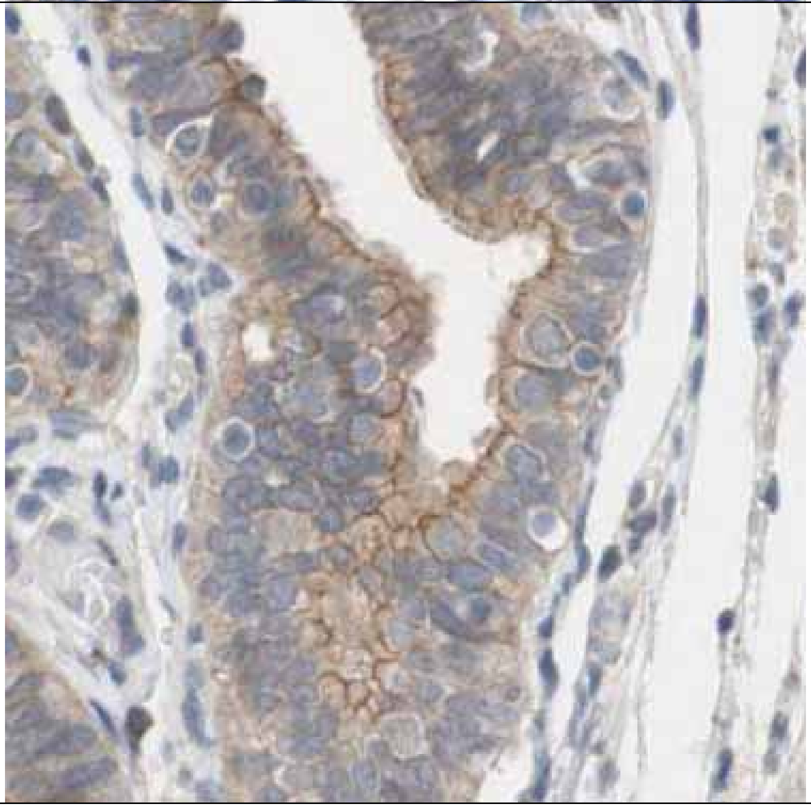	Staining is non-specific to ciliated cells or absent

C9orf103 (antibody HPA020378)<br><br>Category 3	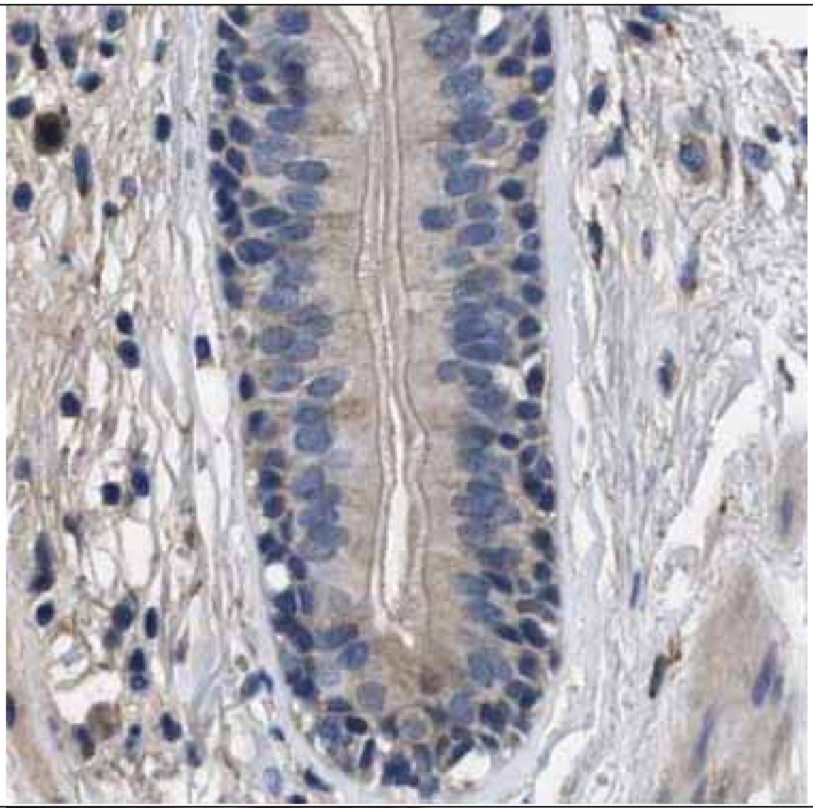	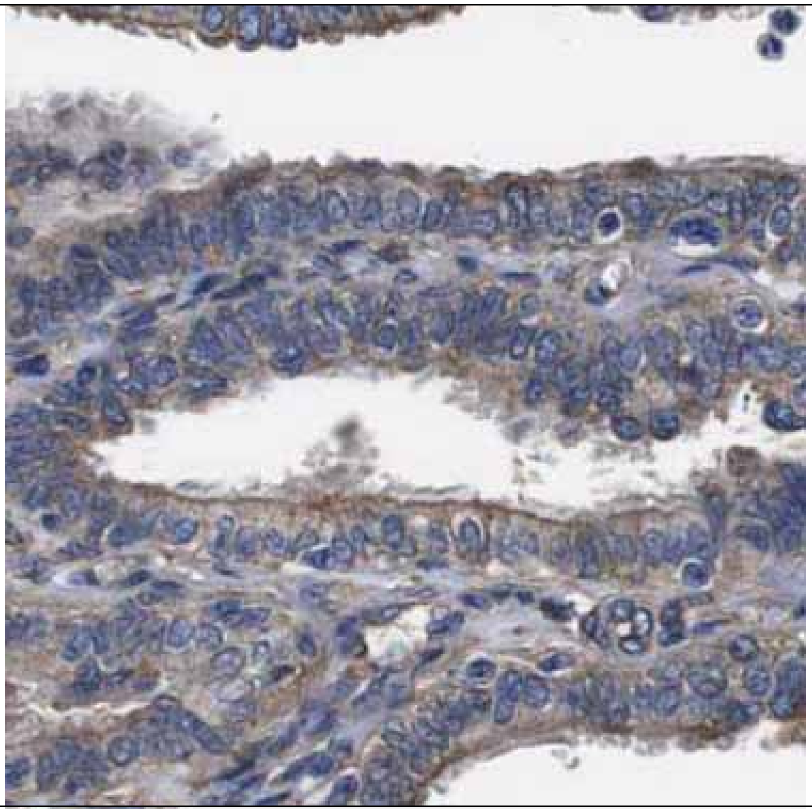	Staining is non-specific to ciliated cells or absent
C10orf57 (antibody HPA009025)<br><br>Category 3	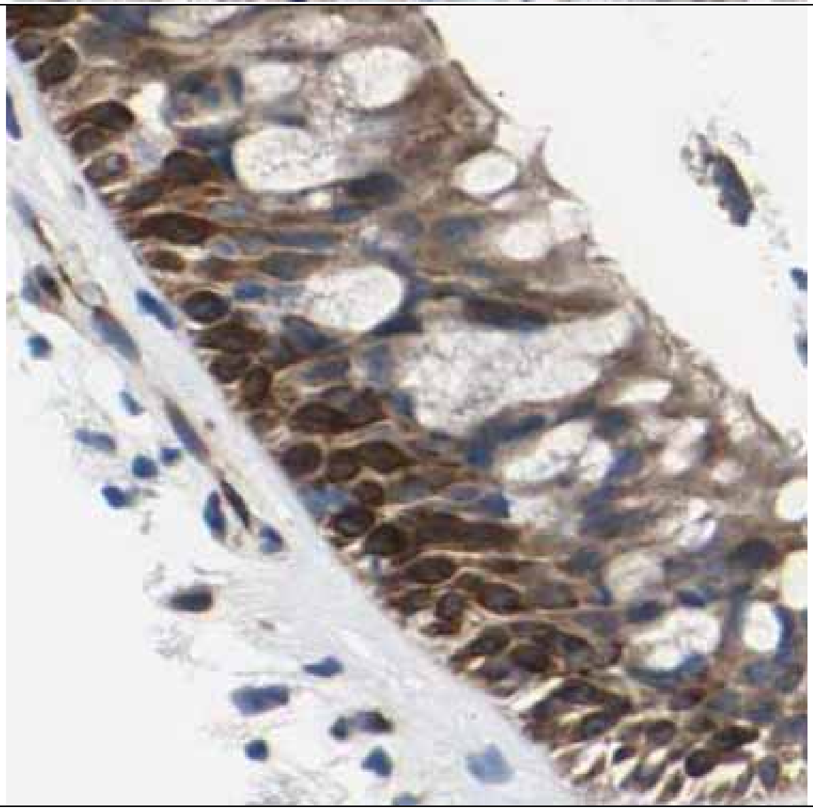	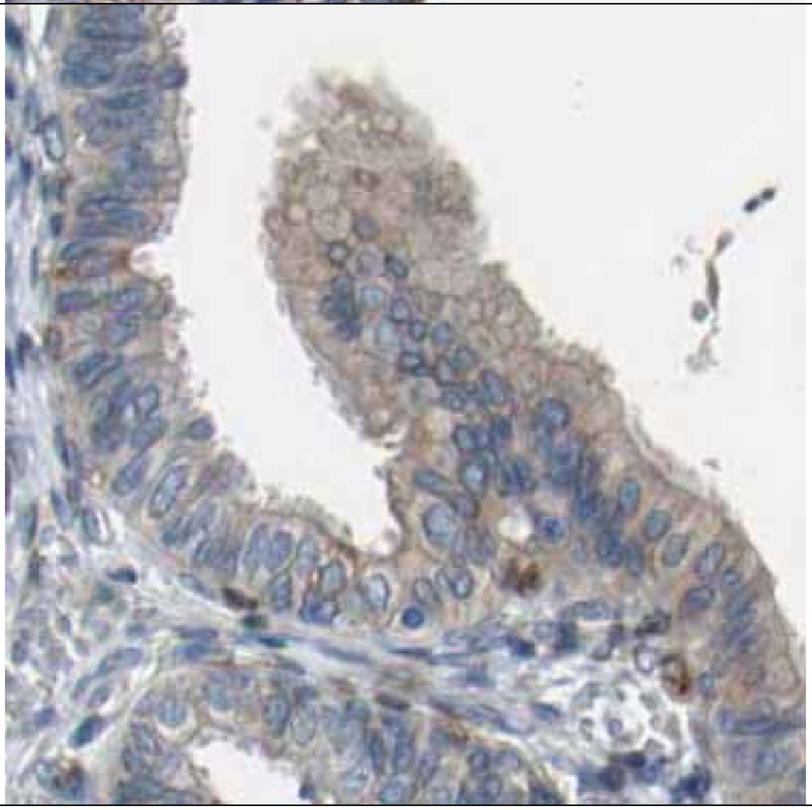	Staining is non-specific to ciliated cells or absent

FABP6 (antibody HPA012601)<br><br>Category 3	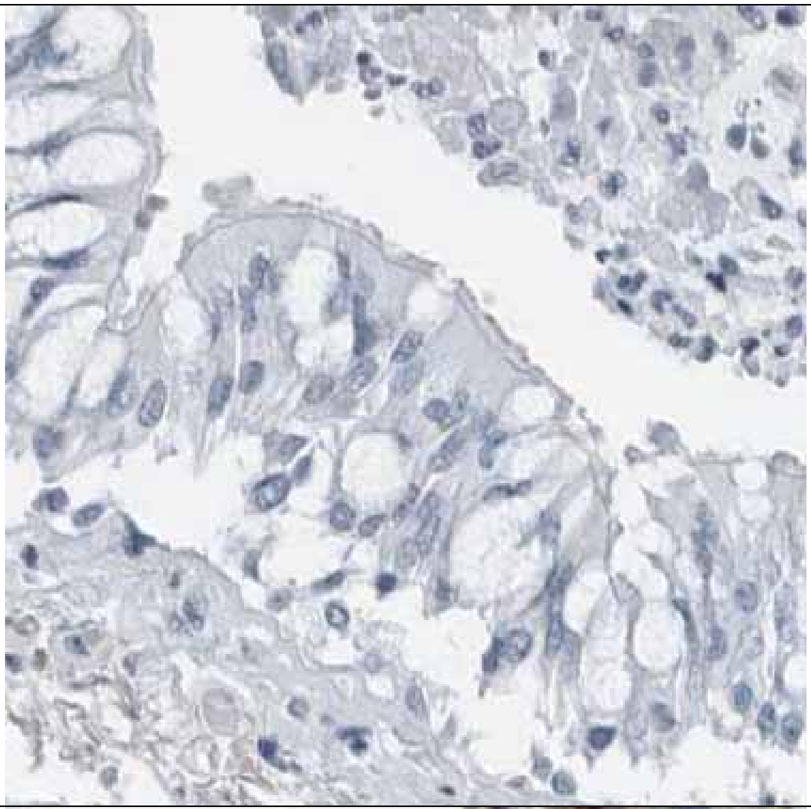	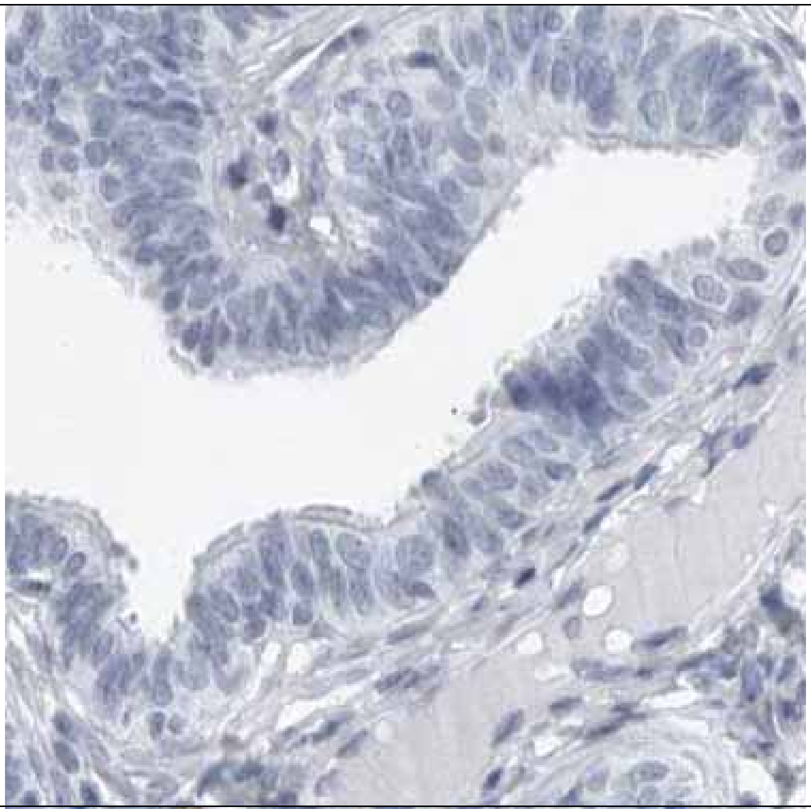	Staining is non-specific to ciliated cells or absent
FAM174A (antibody HPA019539)<br><br>Category 3	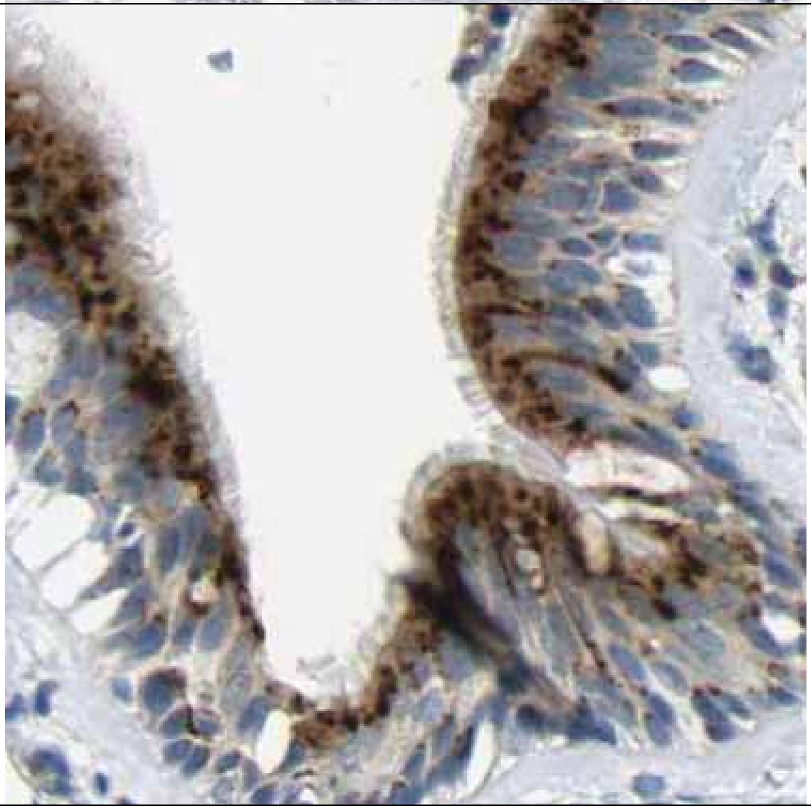	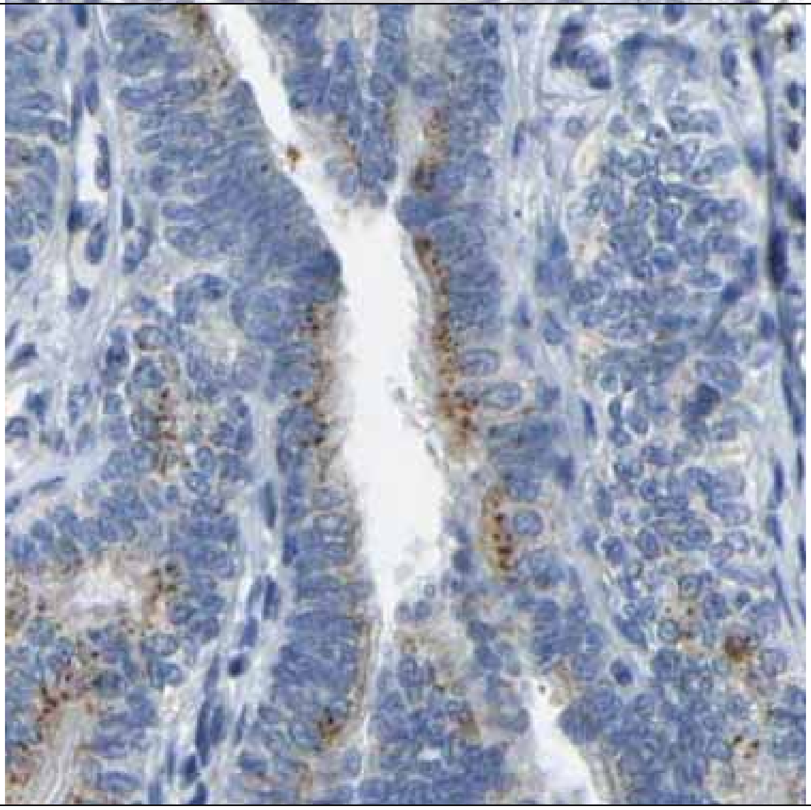	Staining is non-specific to ciliated cells or absent

GLT8D1 (antibody HPA010588)<br><br>Category 3	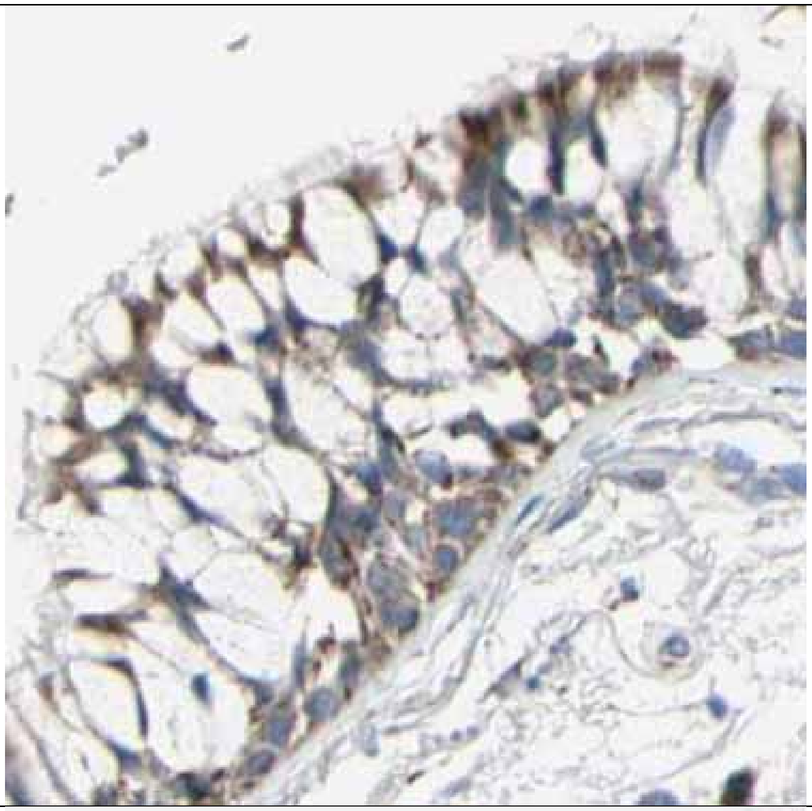	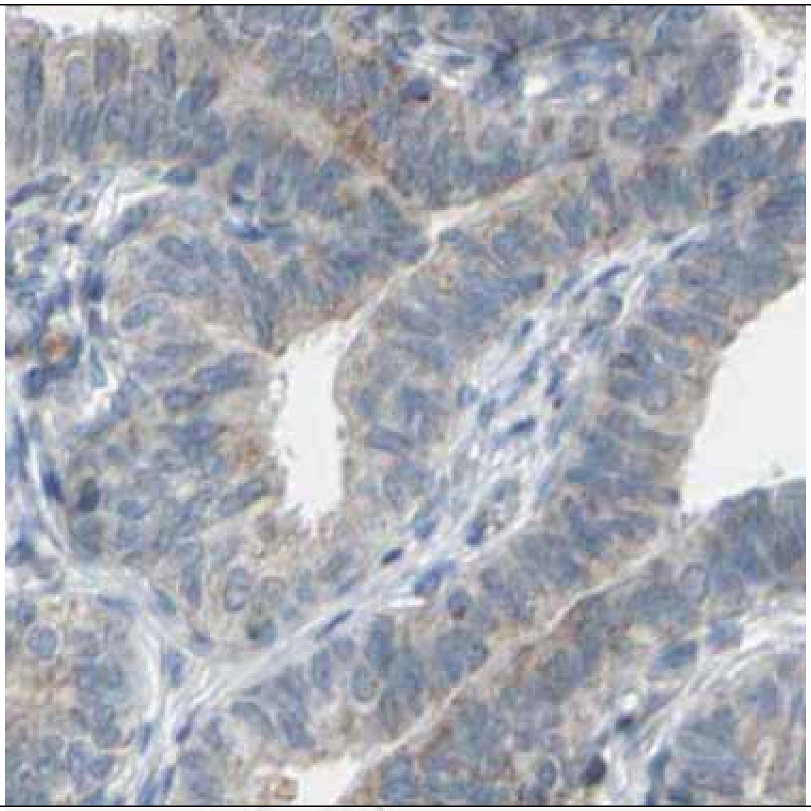	Staining is non-specific to ciliated cells or absent
GRAMD2 (antibody HPA029435)<br><br>Category 3	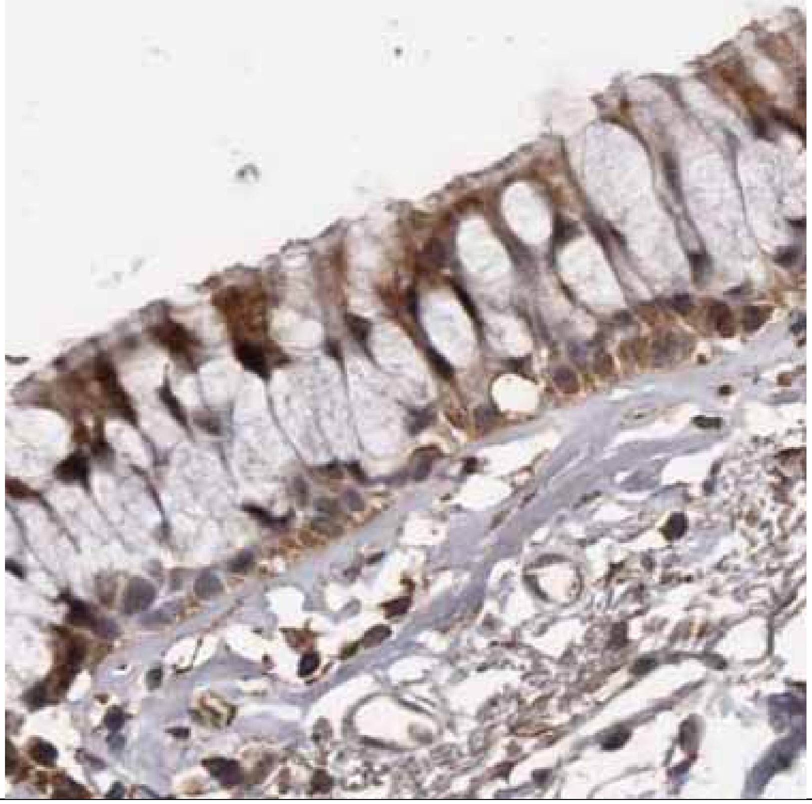	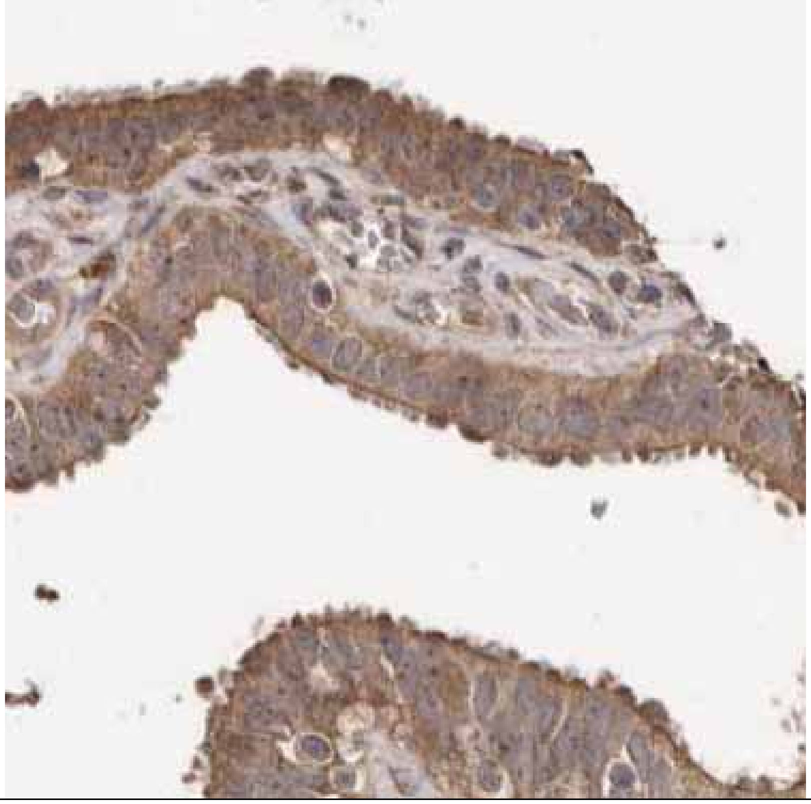	Staining is non-specific to ciliated cells or absent

IL5RA (antibody HPA013196)<br><br>Category 3	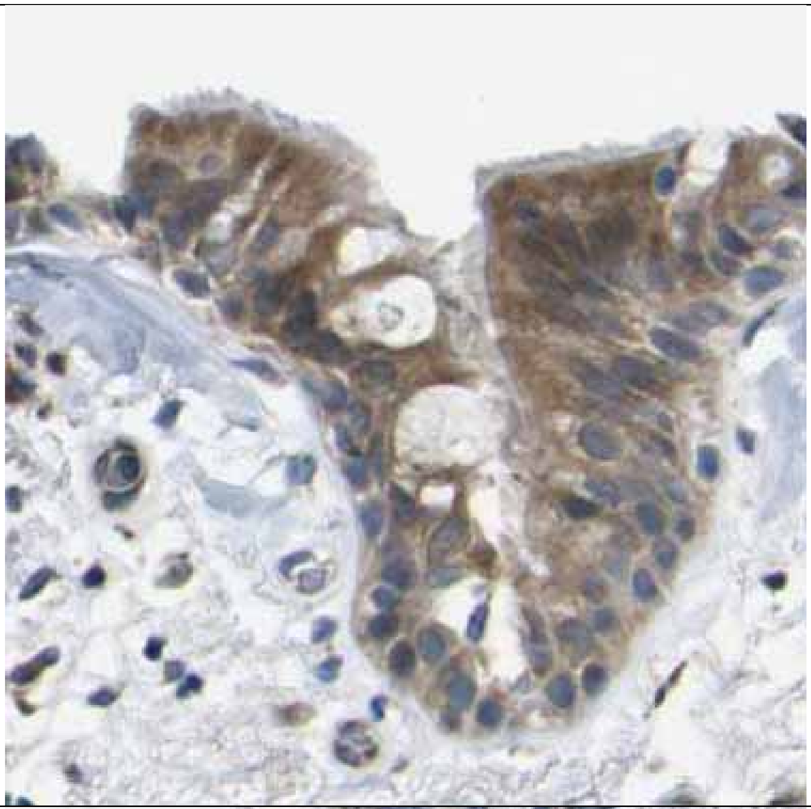	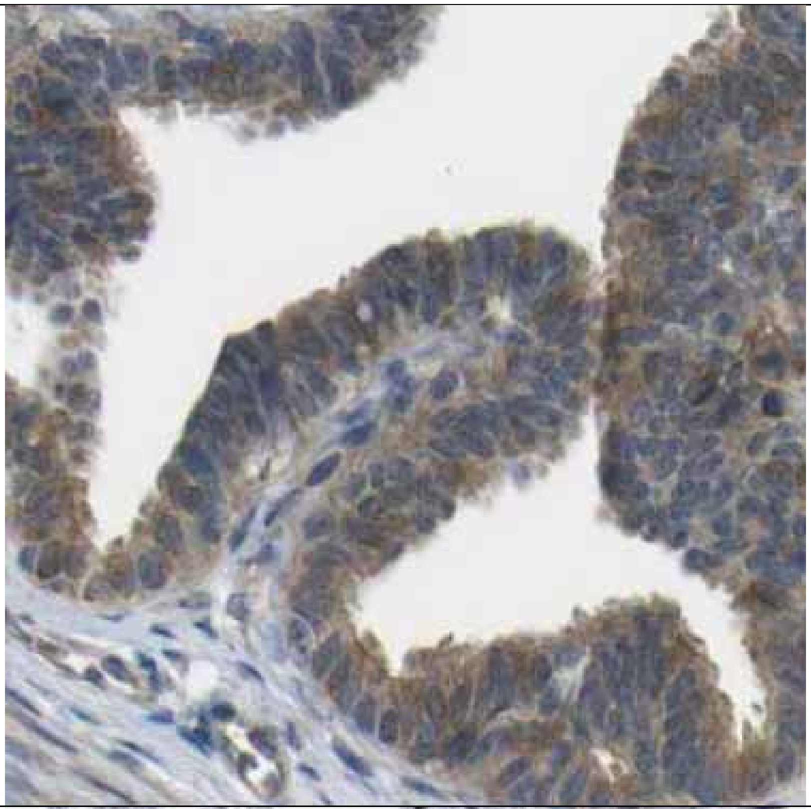	Staining is non-specific to ciliated cells or absent
KCNMB2 (antibody CAB022649)<br><br>Category 3	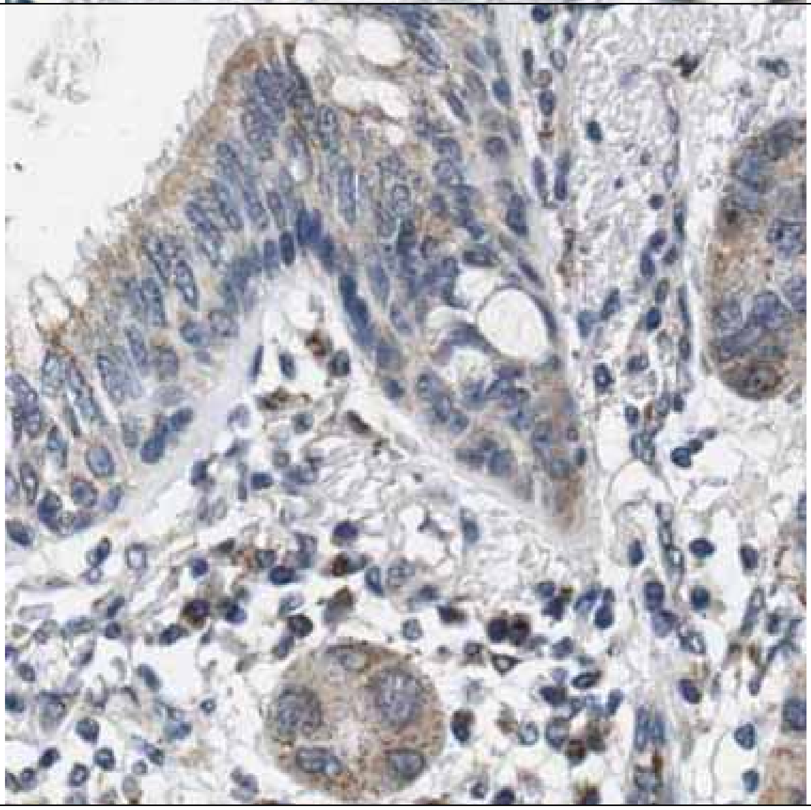	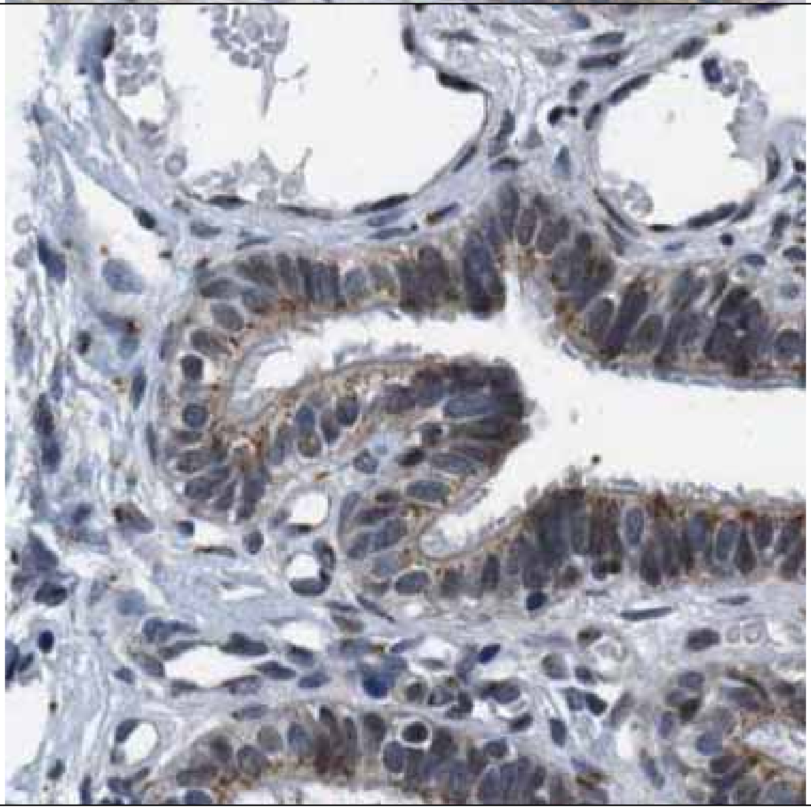	Staining is non-specific to ciliated cells or absent

KIAA0556 (antibody HPA035090)<br><br>Category 3	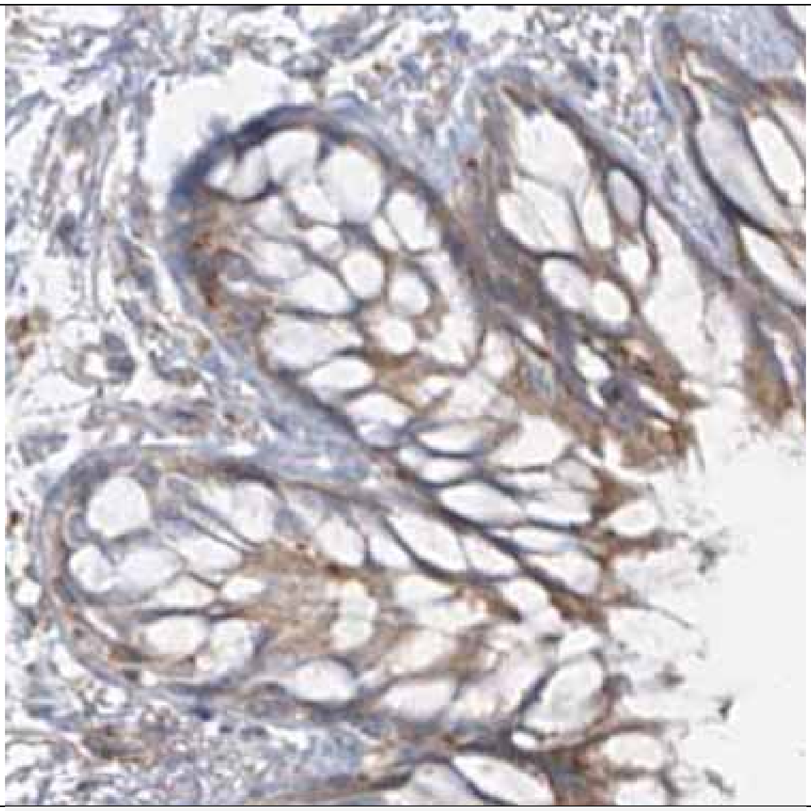	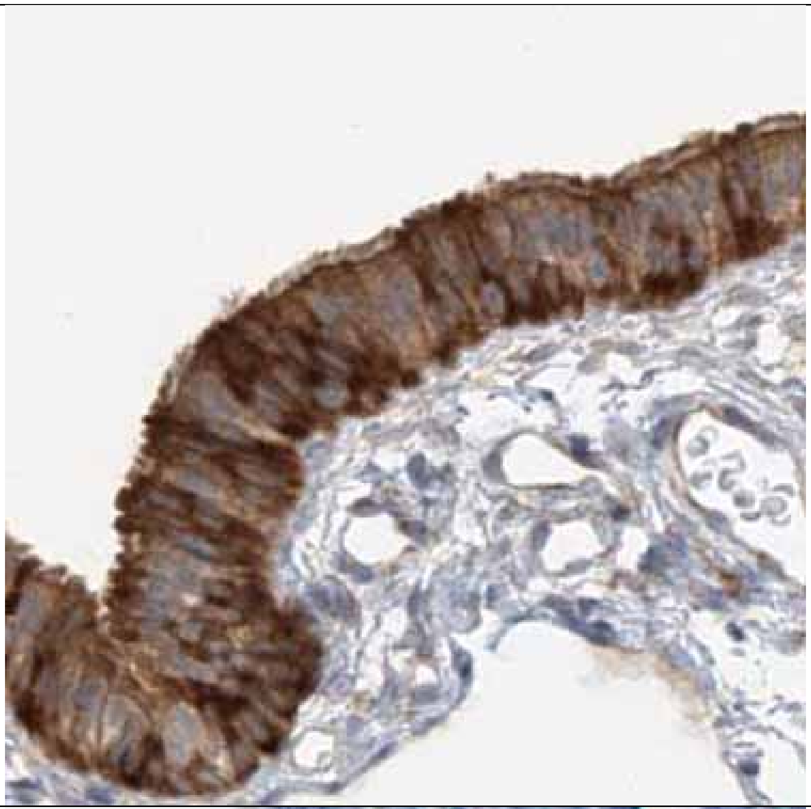	Staining is non-specific to ciliated cells or absent
LCA5L (antibody HPA019790)<br><br>Category 3	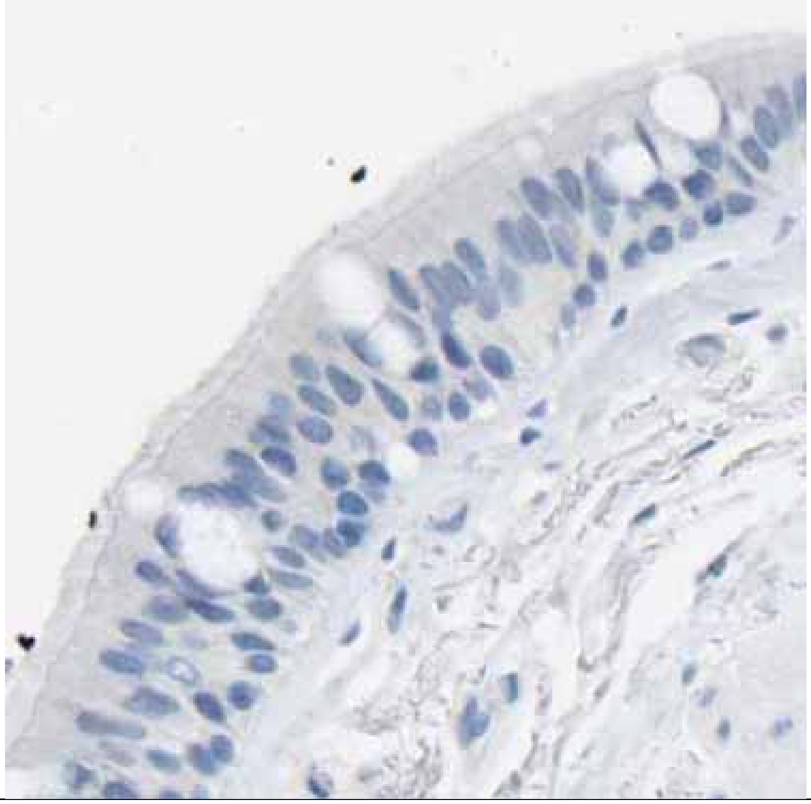	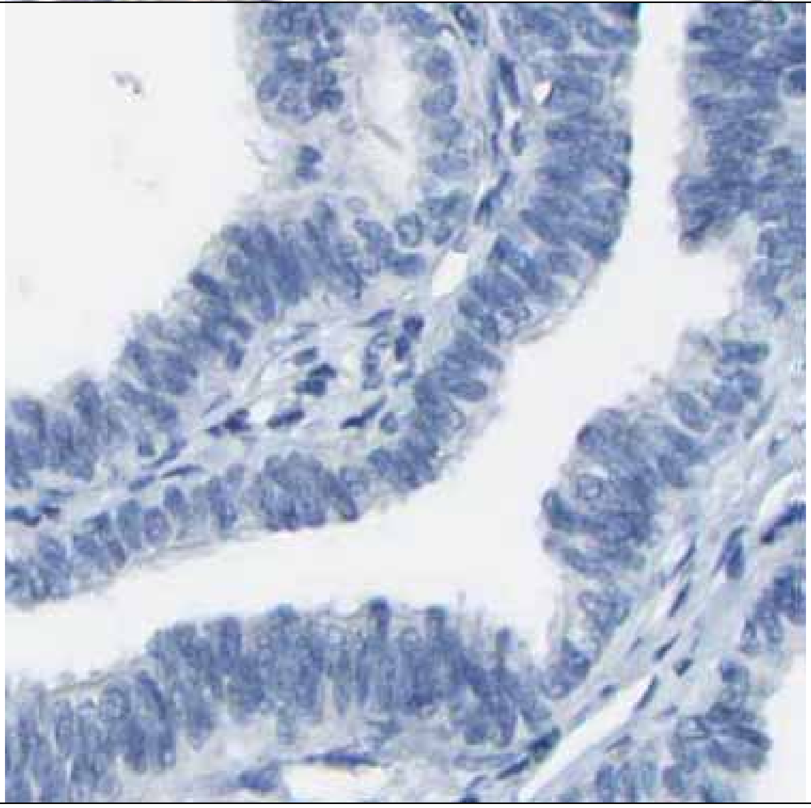	Staining is non-specific to ciliated cells or absent

MARCH10 (antibody HPA023316)<br><br>Category 3	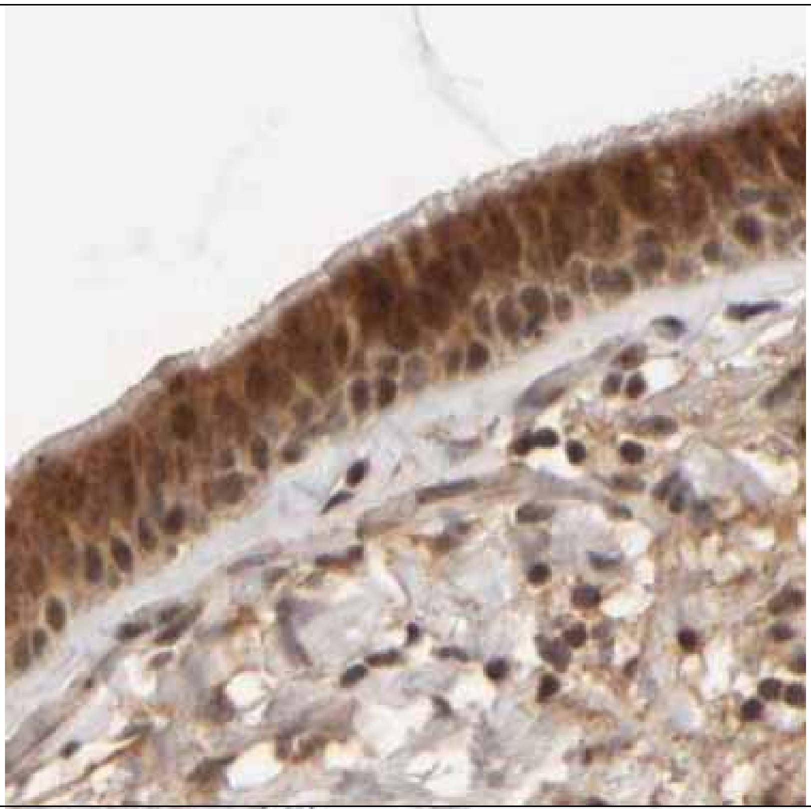	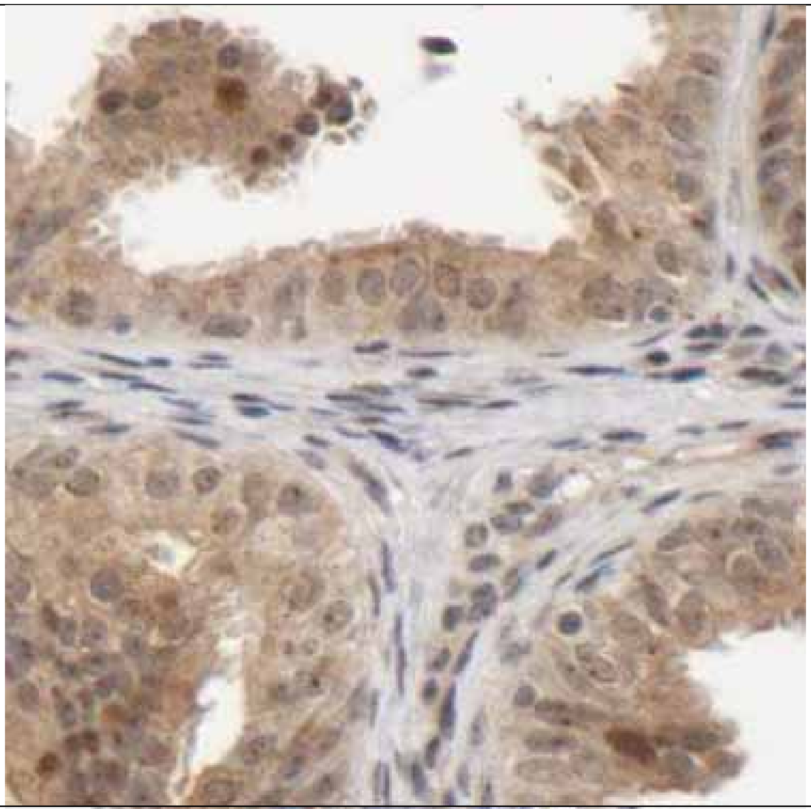	Staining is non-specific to ciliated cells or absent
NELL2 (antibody HPA035715)<br><br>Category 3	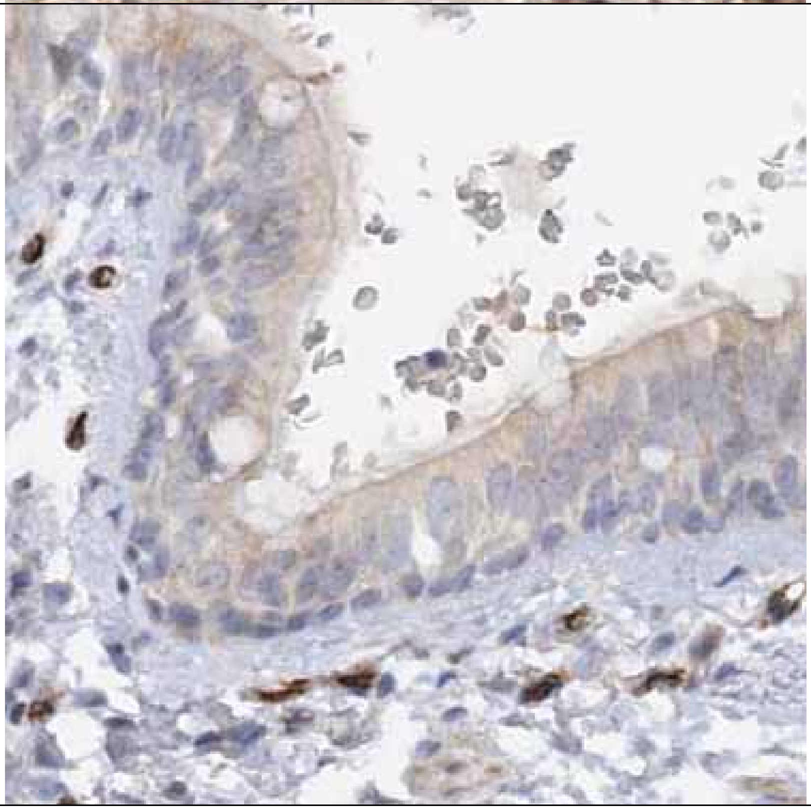	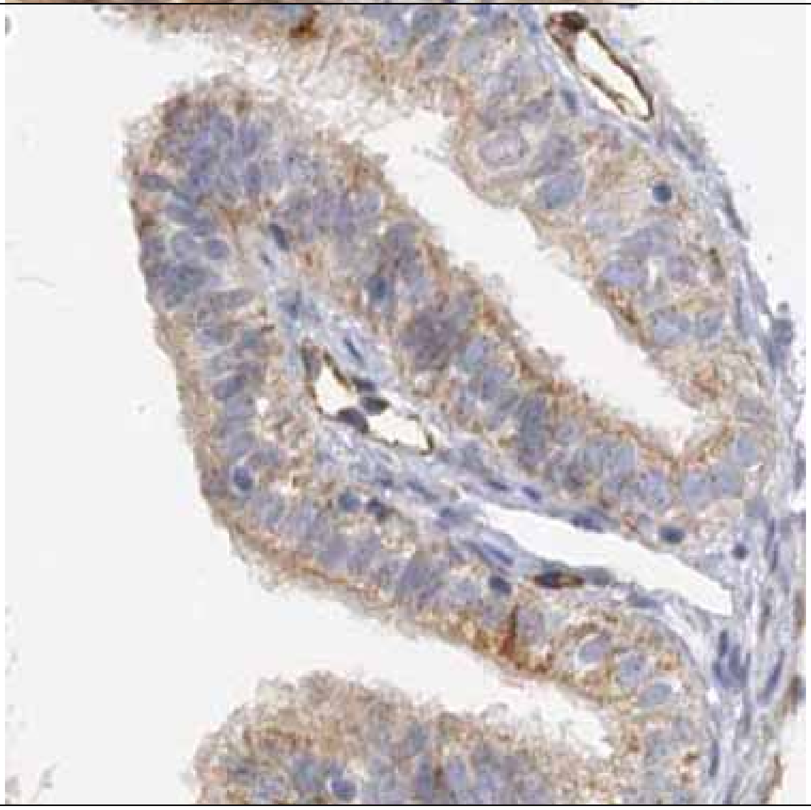	Staining is non-specific to ciliated cells or absent

PERP (antibody HPA022269)<br><br>Category 3	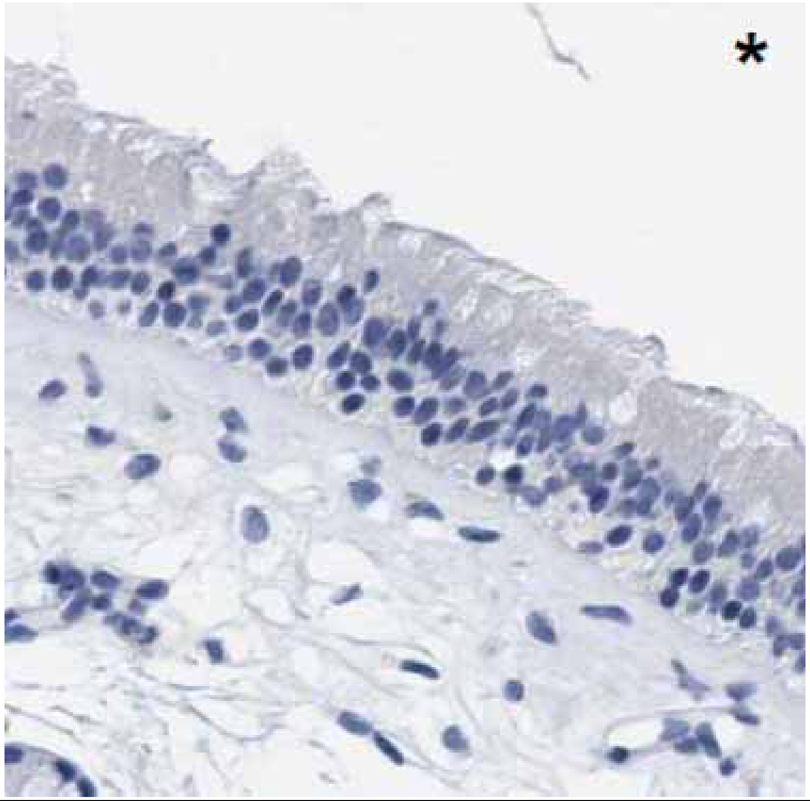	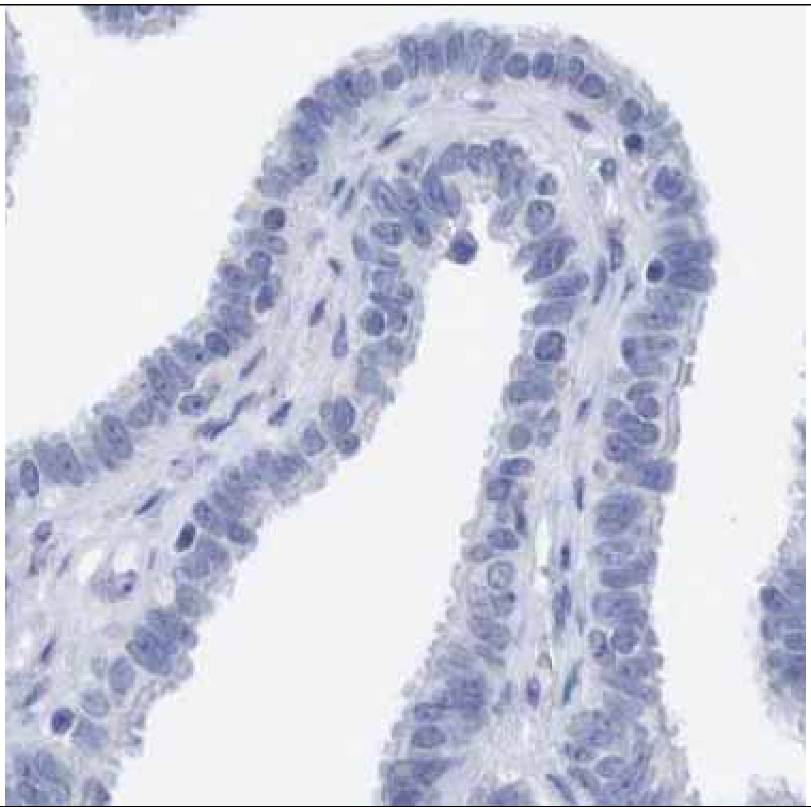	Staining is non-specific to ciliated cells or absent
SNCAIP (antibody HPA003266)<br><br>Category 3	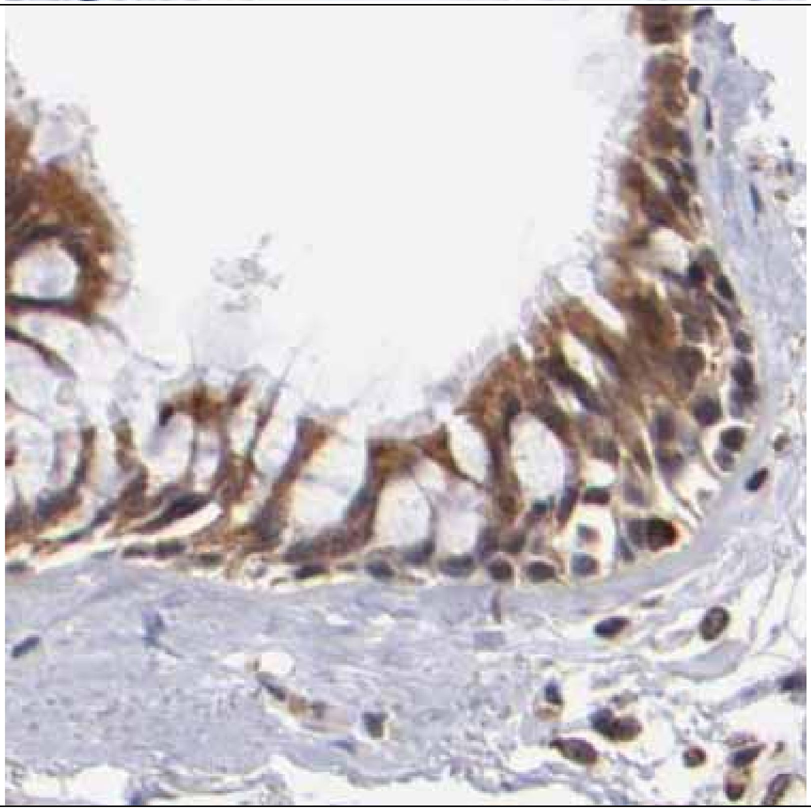	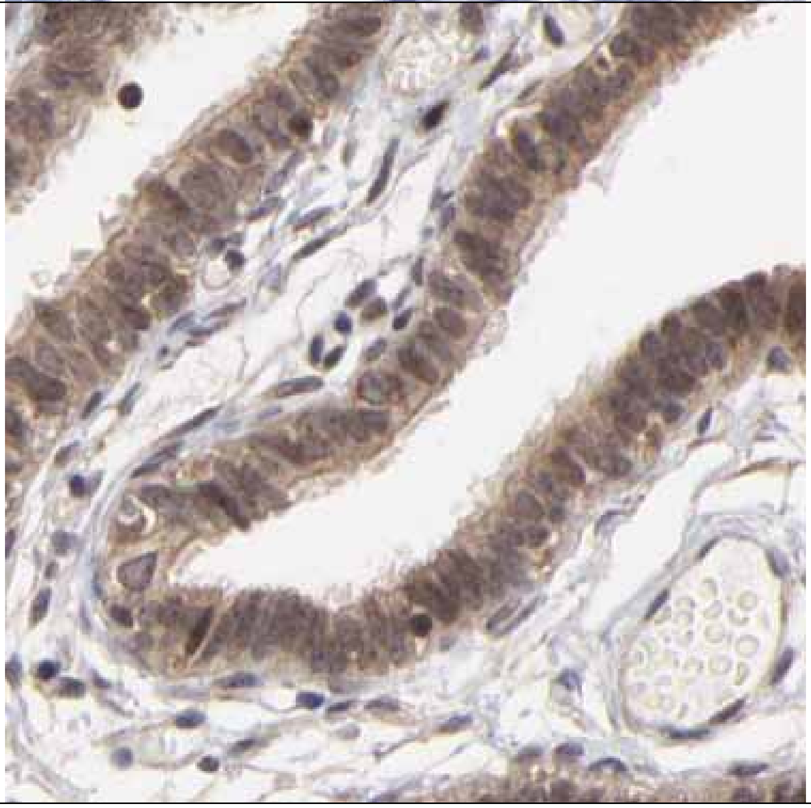	Staining is non-specific to ciliated cells or absent

TIGD4 (antibody HPA035649)<br><br>Category 3	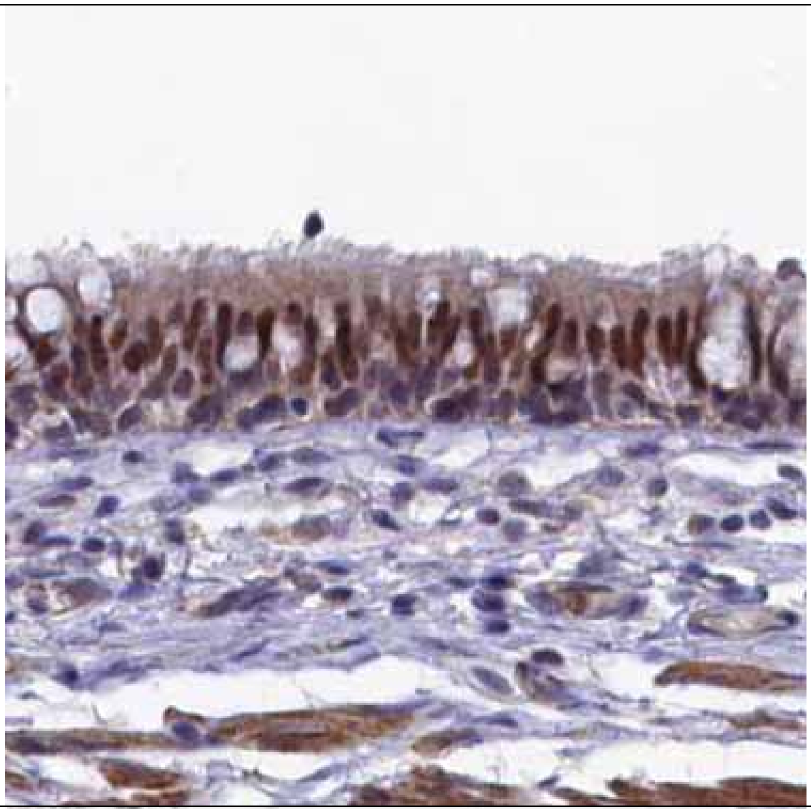	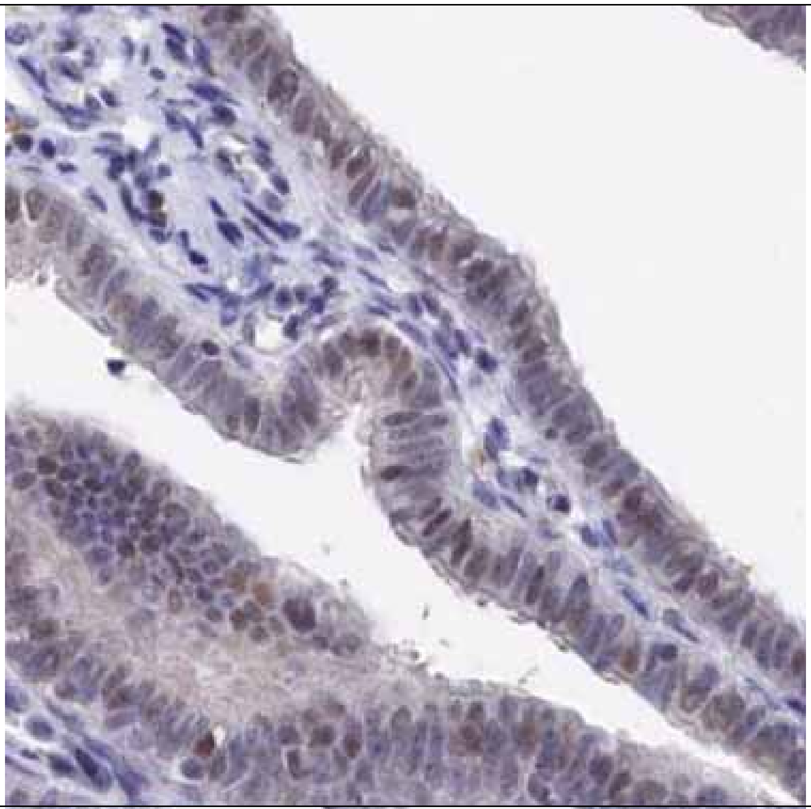	Staining is non-specific to ciliated cells or absent
TMEM146 (antibody HPA024056)<br><br>Category 3	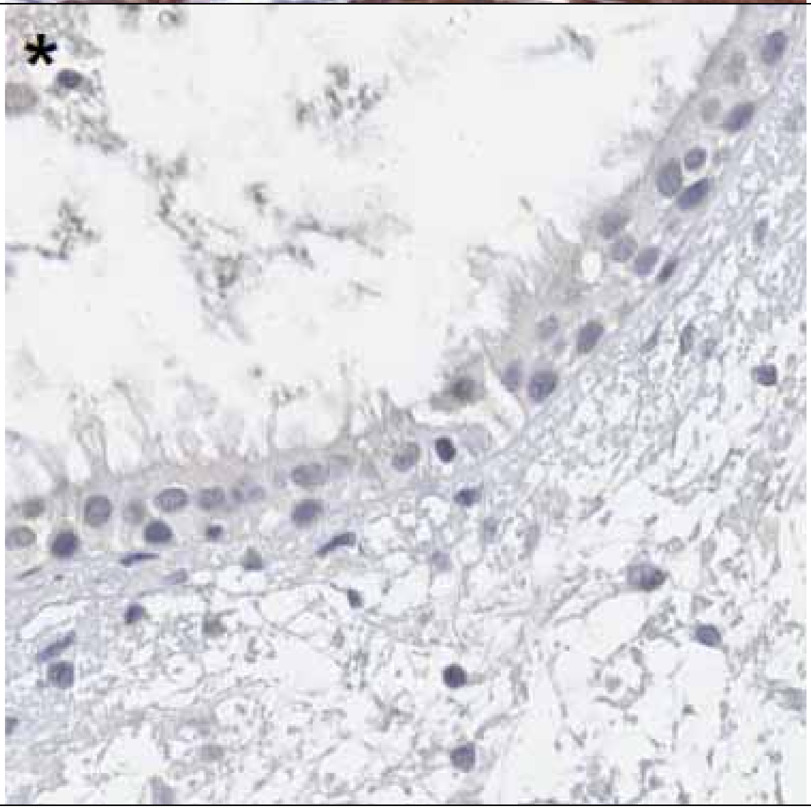	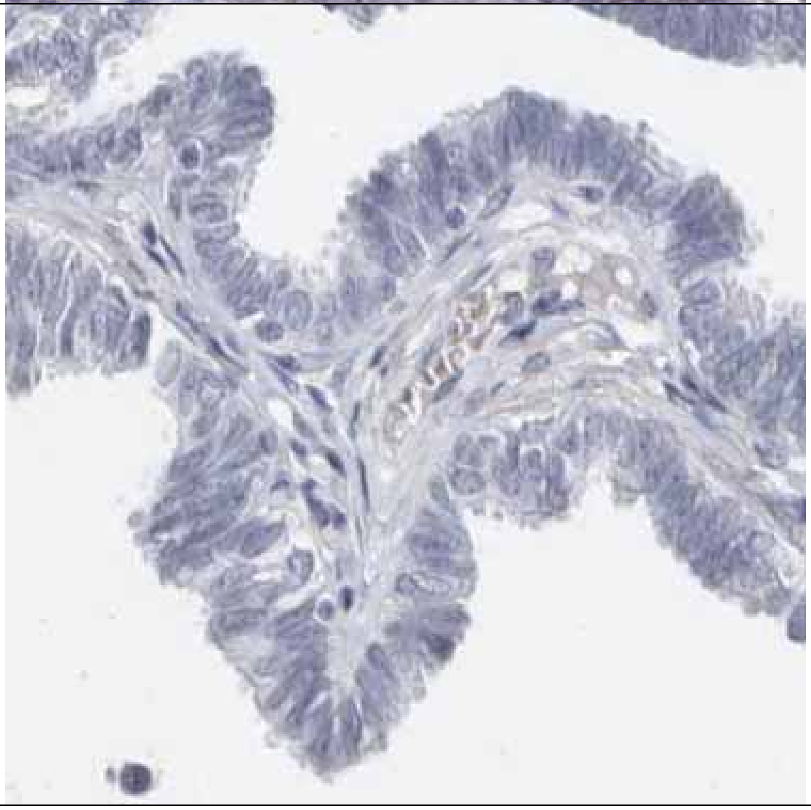	Staining is non-specific to ciliated cells or absent

TSPAN6 (antibody HPA004109)<br><br>Category 3	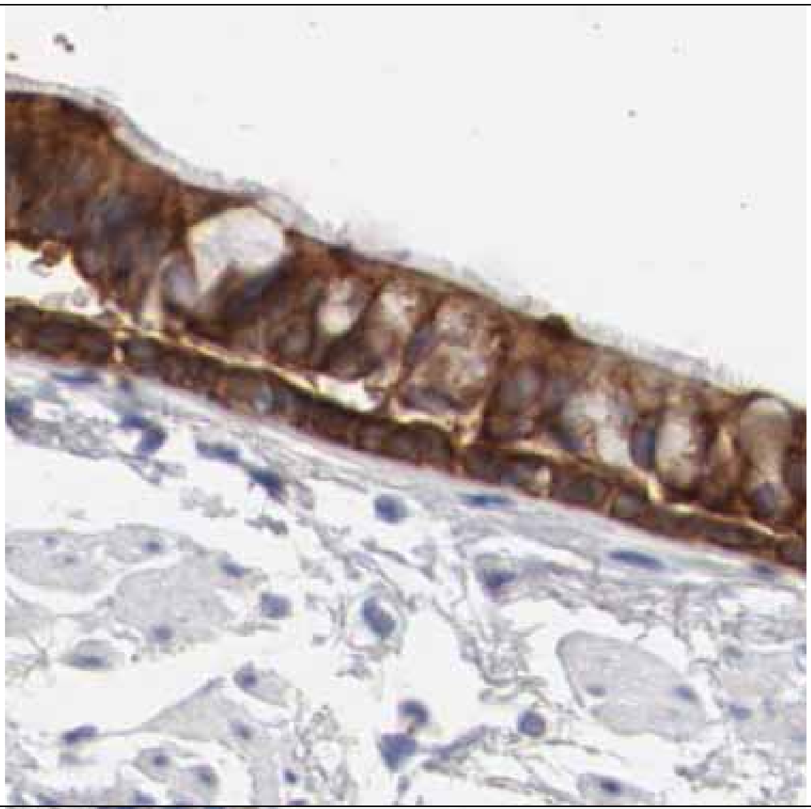	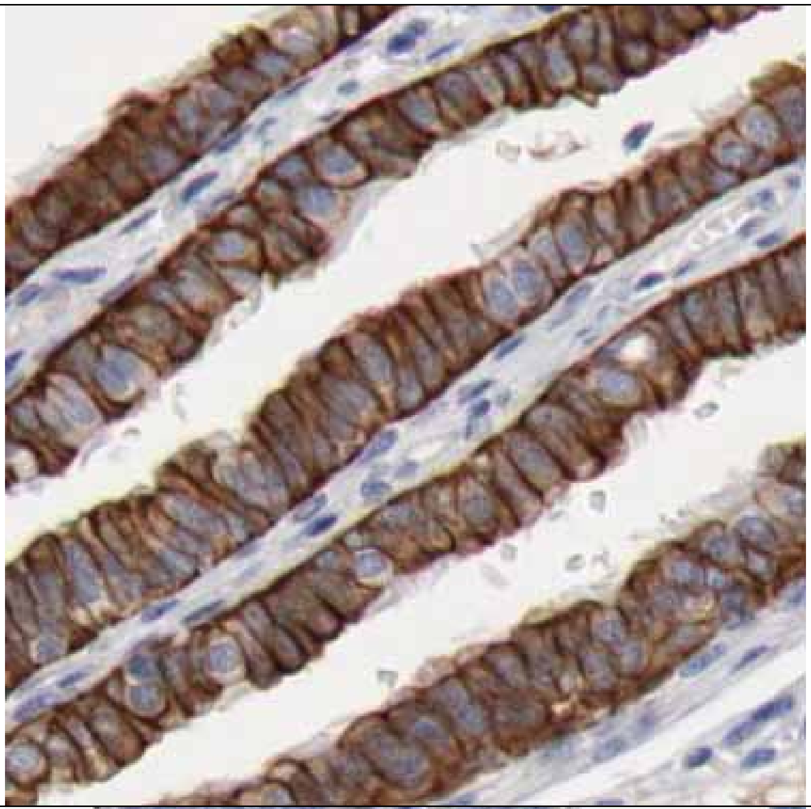	Staining is non-specific to ciliated cells or absent
ULK4 (antibody HPA017930)<br><br>Category	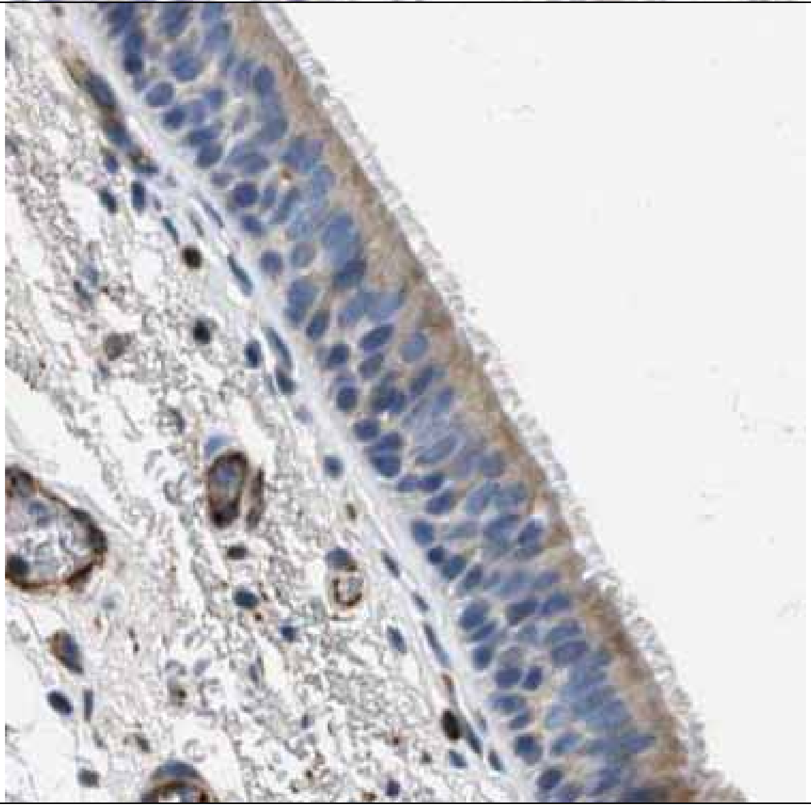	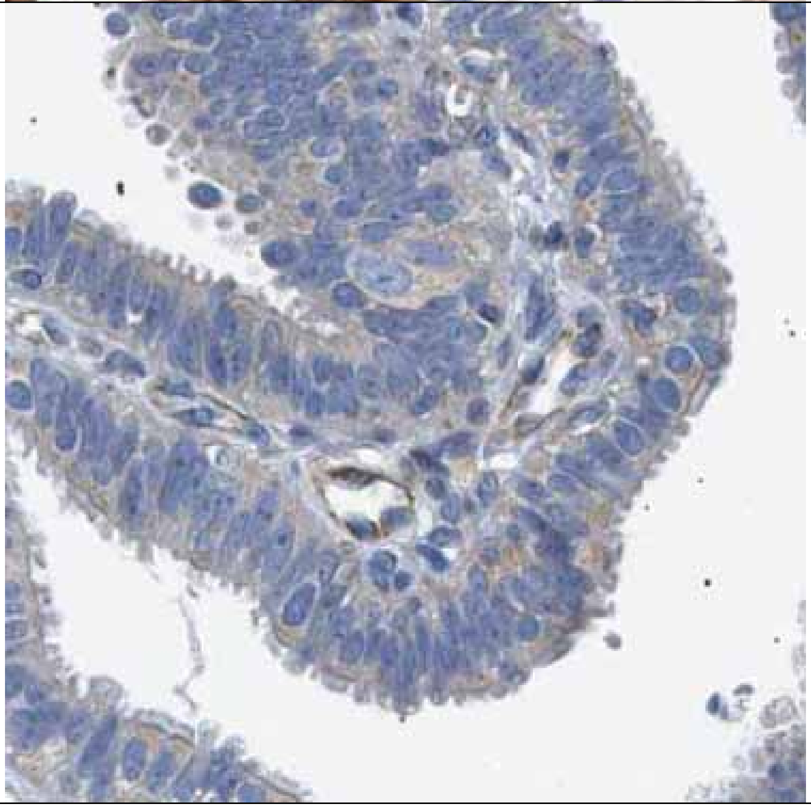	Staining is non-specific to ciliated cells or absent

WRB (antibody HPA018527)<br><br>Category 3	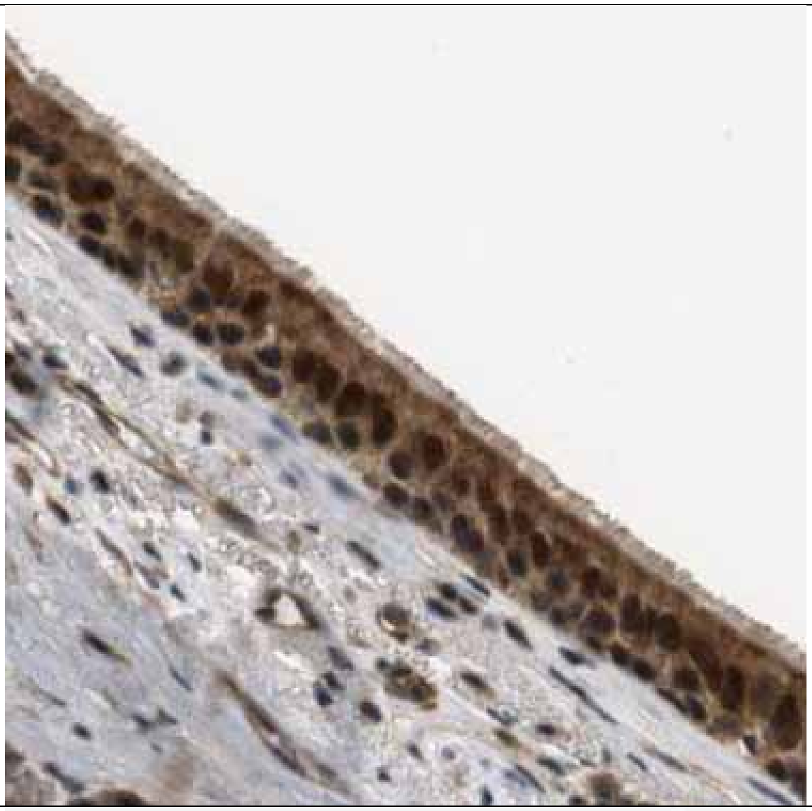	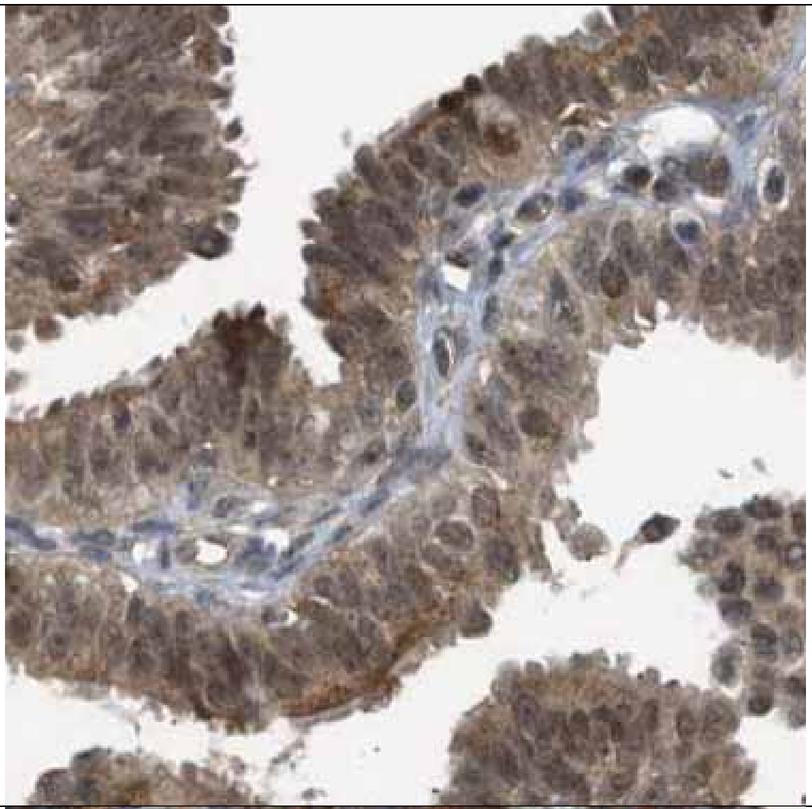	Staining is non-specific to ciliated cells or absent
XK (antibody HPA019036)<br><br>Category 3	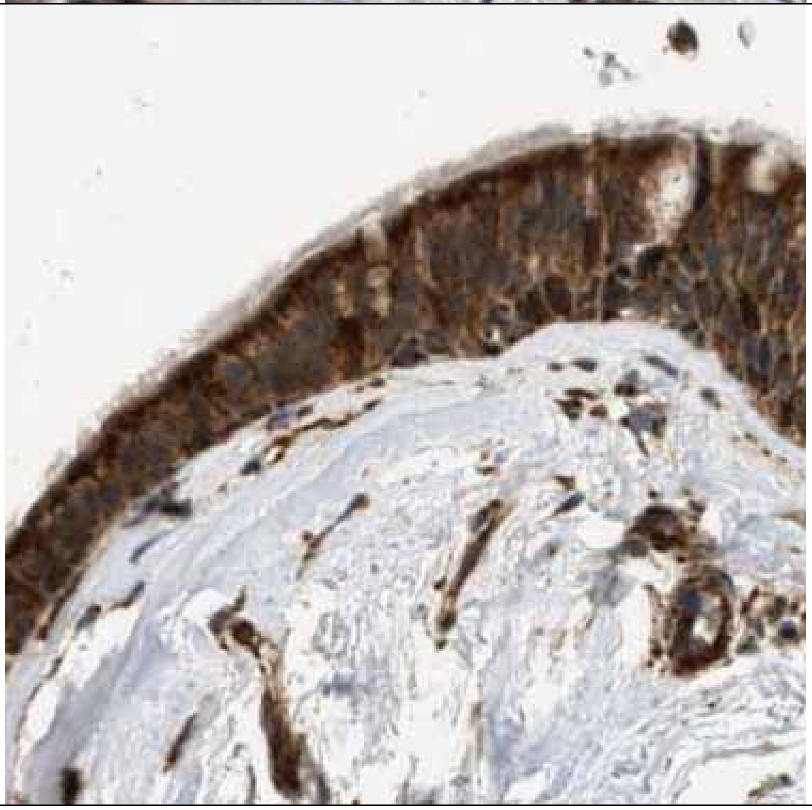	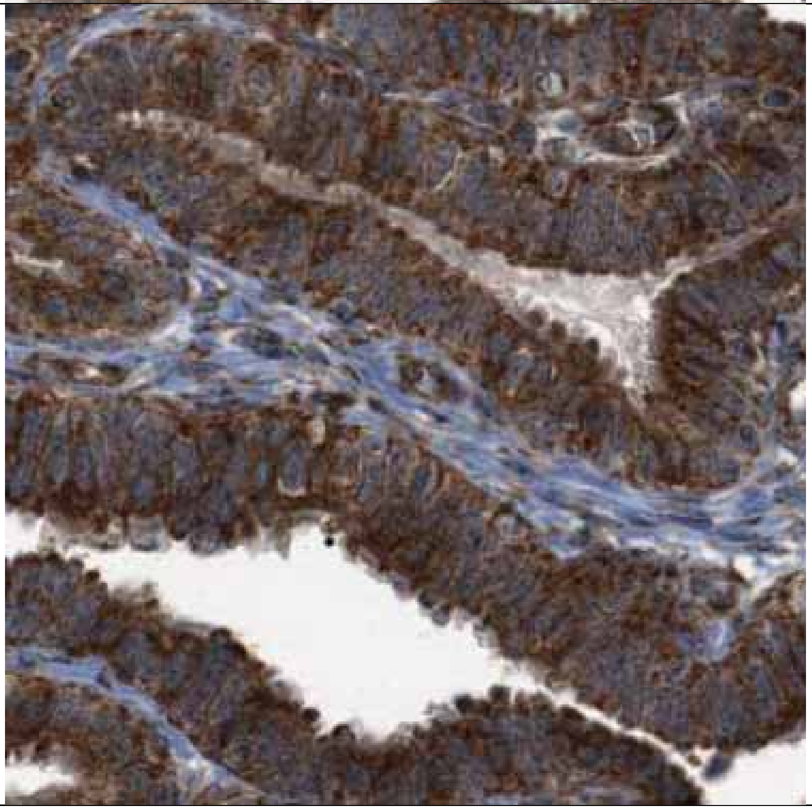	Staining is non-specific to ciliated cells or absent
